# A disease model resource reveals core principles of tissue-specific cancer evolution

**DOI:** 10.1038/s41586-026-10187-2

**Published:** 2026-02-25

**Authors:** Sebastian Mueller, Niklas de Andrade Krätzig, Markus Tschurtschenthaler, Miguel G. Silva, Chiara Thordsen, Riccardo Trozzo, Perrine Simon, Frederic Saab, Thorsten Kaltenbacher, Magdalena Zukowska, Daniele Lucarelli, Rupert Öllinger, Joscha Griger, Nina Groß, Tanja Groll, Jessica Löprich, Antonio E. Zaurito, Linus R. Schömig, Jeroen M. Bugter, Stefanie Bärthel, Chiara Falcomatà, Alexander Strong, Cordelia Brandt, Mulham Najajreh, Aristeidis Papargyriou, Roman Maresch, Katharina A. N. Collins, David Sailer, Christian Schneeweis, Sebastian Burger, Lisa M. Fröhlich, Christine Klement, Alexander Belka, Juan J. Montero, Ute Jungwirth, Maximilian Reichert, Markus Moser, Jens Neumann, George Vassiliou, Juan Cadiñanos, Ignacio Varela, Carsten Marr, Daniel F. Alonso, Pier-Luigi Lollini, Jean Zhao, Louis Chesler, Clare M. Isacke, Angela Riedel, Christian J. Braun, Martin L. Sos, Filippo Beleggia, Hans C. Reinhardt, Monica Musteanu, Mariano Barbacid, Michael Quante, Marc Schmidt-Supprian, Günter Schneider, Simon Clare, Trevor D. Lawley, Gordon Dougan, Katja Steiger, Nathalie Conte, Allan Bradley, Lena Rad, Dieter Saur, Roland Rad

**Affiliations:** 1 Institute of Molecular Oncology and Functional Genomics, School of Medicine and Health, TU Munich, Munich, Germany; 2 Institute for Translational Cancer Research (TranslaTUM), School of Medicine and Health, TU Munich, Munich, Germany; 3 Division of Translational Cancer Research, German Cancer Research Center (DKFZ) and German Cancer Consortium (DKTK), Heidelberg, Germany; 4 Institute of Experimental Cancer Therapy, School of Medicine and Health, TU Munich, Munich, Germany; 5 German Cancer Consortium (DKTK), German Cancer Research Center (DKFZ), Heidelberg, Germany; 6 Institute of Computational Biology, Helmholtz Munich, Munich, Germany; 7 Comparative Experimental Pathology, School of Medicine and Health, TU Munich, Munich, Germany; 8 Institute of Pathology, School of Medicine and Health, TU Munich, Munich, Germany; 9 Klinik für Innere Medizin II, Universitätsklinikum Freiburg, Freiburg, Germany; 10 Genome Campus, Wellcome Trust Sanger Institute, Hinxton, UK; 11 Center for Functional Protein Assemblies, TU Munich, Garching, Germany; 12 Center for Organoid Systems (COS), TU Munich, Garching, Germany; 13 Department of Medicine II, TUM University Hospital, TU Munich, Munich, Germany; 14 Translational Pancreatic Cancer Research Center, Department of Medicine II, TUM University Hospital, TU Munich, Munich, Germany; 15 Institute of Stem Cell Research, Helmholtz Munich, Munich, Germany; 16 Department of Medicine III, LMU University Hospital, LMU Munich, Munich, Germany; 17 Department of Translational Oncology, German Cancer Research Center (DKFZ), LMU Munich, Munich, Germany; 18 Newcastle Drug Discovery Group, Translational and Clinical Research Institute, Newcastle University, Newcastle, UK; 19 Institute of Experimental Hematology, School of Medicine and Health, TU Munich, Munich, Germany; 20 Institute of Pathology, Faculty of Medicine, LMU Munich, Munich, Germany; 21 Department of Haematology, Cambridge University Hospitals NHS Trust, Cambridge, UK; 22 Fundación Centro Médico de Asturias, Oviedo, Spain; 23 Instituto de Biomedicina y Biotecnología de Cantabria (IBBTEC), Universidad de Cantabria-CSIC, Santander, Spain; 24 Institute of AI for Health, Helmholtz Munich, Neuherberg, Germany; 25 Center for Molecular and Translational Oncology (COMTra), National University of Quilmes (UNQ), Buenos Aires, Argentina; 26 National Scientific and Technical Research Council (CONICET), Buenos Aires, Argentina; 27 Department of Medical and Surgical Sciences (DIMEC), University of Bologna, Bologna, Italy; 28 IRCCS Azienda Ospedaliera Universitaria di Bologna, Bologna, Italy; 29 Broad Institute of Harvard and MIT, Cambridge, MA USA; 30 Department of Biological Chemistry and Molecular Pharmacology, Harvard Medical School, Boston, MA USA; 31 Department of Cancer Biology, Dana-Farber Cancer Institute, Boston, MA USA; 32 Laboratory of Systems Pharmacology, Harvard Medical School, Boston, MA USA; 33 Division of Clinical Studies, The Institute of Cancer Research (ICR) and Royal Marsden NHS Trust, Sutton, UK; 34 Breast Cancer Now Toby Robins Research Centre, The Institute of Cancer Research, London, UK; 35 Mildred Scheel Early Career Centre, University Hospital of Würzburg, Würzburg, Germany; 36 Department of Pediatrics, Dr. von Hauner Children’s Hospital, LMU University Hospital, LMU Munich, Munich, Germany; 37 Hopp Children’s Cancer Center Heidelberg (KiTZ), German Cancer Research Center (DKFZ), Heidelberg, Germany; 38 Department of Translational Genomics, Faculty of Medicine and University Hospital Cologne, University of Cologne, Cologne, Germany; 39 Department I of Internal Medicine, Faculty of Medicine and University Hospital Cologne, University of Cologne, Cologne, Germany; 40 Mildred Scheel School of Oncology Cologne, Faculty of Medicine and University Hospital Cologne, University of Cologne, Cologne, Germany; 41 Department of Hematology and Stem Cell Transplantation, West German Cancer Center Essen, University Hospital Essen, Essen, Germany; 42 Centro de Investigación Biomédica en Red de Cáncer, Instituto de Salud Carlos III, Madrid, Spain; 43 Department of Biochemistry and Molecular Biology, Faculty of Pharmacy, Complutense University of Madrid, Madrid, Spain; 44 Experimental Oncology Group, Centro Nacional de Investigaciones Oncológicas, Madrid, Spain; 45 Clinical Research Unit 5002, KFO5002, University Medical Center Göttingen, Göttingen, Germany; 46 Department of General, Visceral and Pediatric Surgery, University Medical Center Göttingen, Göttingen, Germany; 47 Centre for Translational Stem Cell Biology, Hong Kong, China

**Keywords:** Oncogenes, Cancer models, Cancer genetics

## Abstract

Oncogenes such as *KRAS* display marked tissue specificity in their oncogenic potential, genetic interactions and phenotypic effects, but the underlying determinants remain largely unresolved^1–5^. Here, to address these questions, we developed the Mouse Cancer Cell line Atlas, a broad-utility resource of 590 comprehensively characterized models across a wide range of entities (www.mcca.tum.de). Comparative and functional studies using this platform, human cohorts and mice identified core principles underlying tissue-specific evolution of *KRAS*-initiated cancers. First, we show that mutant *KRAS* dosage gain through allelic imbalance exerts cell-type-specific effects, defining its timing across entities, as exemplified by dosage-sensitive developmental reprogramming during pancreatic cancer initiation. Second, we highlight how tissue- and stage-specific evolutionary requirements, such as block of differentiation in the intestine, select for *KRAS*-collaborating alterations. Third, we identified context-dependent epistatic *KRAS*–tumour suppressor interactions and show that reciprocal dosage sensitivities dictate the entity-specific patterns of cancer gene alterations, explaining their frequency, zygosity and acquisition chronology. These findings highlight how intrinsic and acquired determinants instruct cancer evolution in different tissues, with predictable molecular patterns, temporal dynamics and phenotypic outcomes. Our study provides major advances towards a mechanistic understanding of cancer genomes.

## Abstract (web summary)

The Mouse Cancer Cell line Atlas (MCCA) provides major advances towards a mechanistic understanding of cancer genomes.

## Main

Cancer genome sequencing efforts have catalogued genetic alterations for all major human cancer types and revealed considerable differences between tissues^1–3^. However, the causes and evolutionary principles shaping cancer genomic landscapes are only partly understood^4,5^. Cell types differ in their susceptibility to transformation by individual oncogenes, and cancer types initiated by the same oncogene vary in their aggressiveness. Moreover, the same oncogene collaborates with distinct secondary alterations in different tissues, and heterogenous patterns of allelic imbalance at cancer genes further complicate the picture. Mechanistically, these and many other observations in cancer evolution remain largely unexplored.

Comprehensively characterized human cell line collections, such as the Cancer Cell Line Encyclopedia (CCLE)^6,7^, have become indispensable resources and sources of major discoveries in cancer research^8^. Although the mouse is the most important mammalian model organism^9–11^, there is no comparable pan-cancer cell line resource available for this species. The mouse offers some unique opportunities, such as the possibility to engineer defined molecular contexts or to model rare cancer types and assemble required sample sizes. Complementarity to human resources also emerges from the possibility to capture desired timepoints or conditions, such as treatment-naive contexts or defined progression stages. Likewise, the potential transplantability of mouse cell lines into immunocompetent hosts can be decisive in a broad spectrum of research contexts, such as the study of cancer ecosystems or the testing of (immuno)therapies^9,12^.

To address the need for such a mouse resource, we assembled cancer cell lines from a broad spectrum of cancer types. The collection encompasses 590 models, for which we provide multilayered molecular, phenotypic and clinical metadata through an interactive web portal (www.mcca.tum.de). We developed analytical tools to infer immunophenotypes from genomic sequencing data to guide the in vivo use of Mouse Cancer Cell line Atlas (MCCA) lines in immunocompetent settings. By combining MCCA data analyses with functional studies in mice and human investigations, we set out to examine cellular, molecular and temporal parameters in the evolution of cancers initiated by *KRAS*. Through analysis of prototype entities originating from terminally differentiated or stem cells (pancreas, lung and intestine), we describe hallmark events and mechanisms underlying tissue-specific oncogenesis. Overall, our study supports a deterministic model of cancer evolution that explains genomic alteration patterns in different cancer types.

## Development and characterization of the MCCA

**Fig. 1:**
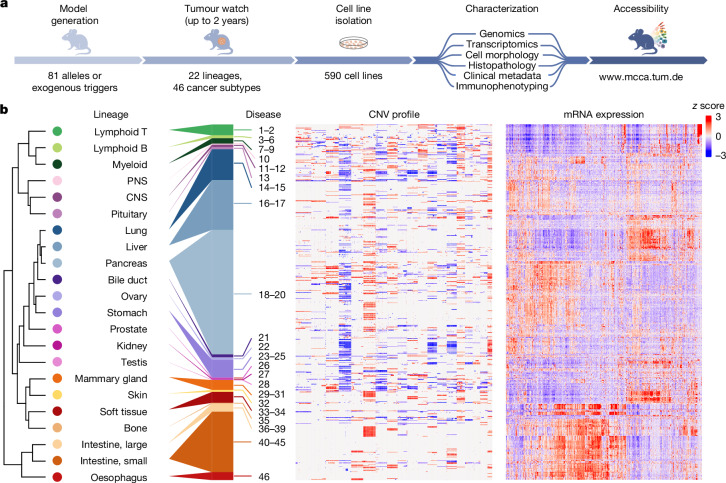
Development and characterization of the MCCA. **a**, Workflow describing the development and characterization of MCCA cell lines. The website providing access to related datasets is indicated. **b**, Overview of selected MCCA datasets derived from the characterization of 590 cell lines, including 36 previously available lines. Cancer types are ordered by unsupervised hierarchical clustering of their transcriptome mean values. Within individual cancer types, each cell line is sorted by transcriptome clustering. Full MCCA annotation is provided in Supplementary Table 1. CNS, central nervous system; PNS, peripheral nervous system.

**Extended Data Fig. 1:**
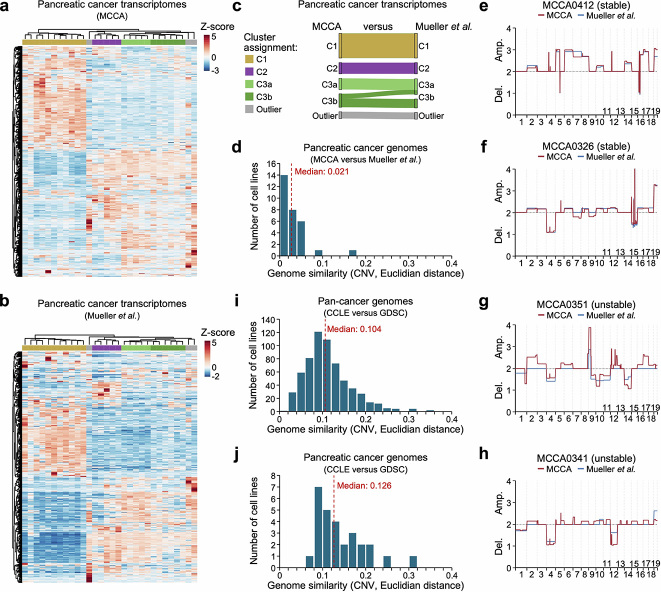
Stability of MCCA cell line transcriptomes and genomes. **a-h**, Stability of transcriptomes and genomes of a set of 30 MCCA pancreatic cancer cell lines characterized at distinct timepoints and passages (Supplementary Table 2). **a,b**, Unbiased hierarchical clustering of transcriptomes from 30 MCCA pancreatic cancer cell lines cultured and profiled by RNA-seq in 2022 (**a**, data from MCCA) or 2016 (**b**, data from^18^, and with up to 13 passages difference. The top 10% most variable expressed genes, as detected by bulk RNA-seq, were used for clustering. Three major transcriptomic clusters (C1, C2, C3) and two subclusters of C3 (C3a and C3b) were identified in both datasets. **c**, Assignment of pancreatic cancer cell line transcriptomes into major transcriptomic clusters (C1, C2, C3) is largely preserved between 2022 and 2016 data sets. Two cell lines were found to switch from C3b to C3a. This observed discordance is however minor, given the small Euclidean distance between both subclusters (**a,b**). **d**, Histogram showing the genomic stability of the same set of 30 pancreatic cancer cell lines cultured and profiled by lcWGS in 2022 (data from MCCA) or WES in 2012 (data from^18^, and with up to 12 passages difference. The conservation of two corresponding copy number profiles was quantified by calculating their Euclidean distance based on the overlap of copy number segment positions as well as on the similarity of corresponding log_2_ copy ratio changes (see Methods). Twenty-eight of 30 cell lines showed relatively stable copy number profiles (Euclidian distance <0.06), while genomes of two cell lines were less conserved (Euclidian distance >0.09). **e,f**, Representative plots of overlayed copy number profiles are shown for cell lines with relatively stable genomes (Euclidean distance <0.06) in (**d**). **g,h**, Plots showing overlayed copy number profiles of both pancreatic cancer cell lines with relatively unstable genomes (Euclidean distance >0.09) in (**d**). Discordance of genomes largely arises from chromosome arm-level gains or losses. **i,j**, Histograms showing the genomic stability of human cancer cell lines cultured and profiled in the Cancer Cell Line Encyclopedia (CCLE) and the Genomics of Drug Sensitivity in Cancer (GDSC) projects. Both projects conducted independent copy number profiling of largely overlapping cell line panels that were propagated over years at different institutions. The conservation of two corresponding copy number profiles was quantified by calculating their Euclidean distance similar to the analysis of mouse pancreatic cancer cell lines shown in (**d**) (see Methods). The conservation of copy number profiles between two human cancer cell lines is shown for all 625 (**i**) or the subset of human pancreatic cancer cell lines ((**j**); for its comparison to mouse pancreatic cancer in (**d**)).

To address the limited availability of non-human cell line resources, we developed the MCCA (Fig. 1a,b). We derived primary cell cultures (hereafter, cell lines) from 81 mouse models of cancer, encompassing tumours induced by engineered oncogene/tumour suppressor alleles or exogenous triggers (Supplementary Table 1). Alongside established genetically engineered mouse models, we also developed models to study genetic, inflammation-associated or irradiation-induced cancers. Examples include genetically engineered cholangiocarcinomas, *Helicobacter*-induced stomach adenocarcinomas or numerous cancer types triggered by γ-irradiation (Supplementary Table 1). Moreover, we characterized 36 publicly available cancer cell lines commonly used in basic and translational research. In total, the MCCA encompasses 590 cell lines, covering 22 lineages and 46 disease types (Supplementary Table 1). To ensure the long-term preservation of cell lines and the high-quality nature of related data, we established rigorous protocols for MCCA handling and characterization (Methods and Extended Data Fig. 1a–j).

**Extended Data Fig. 2:**
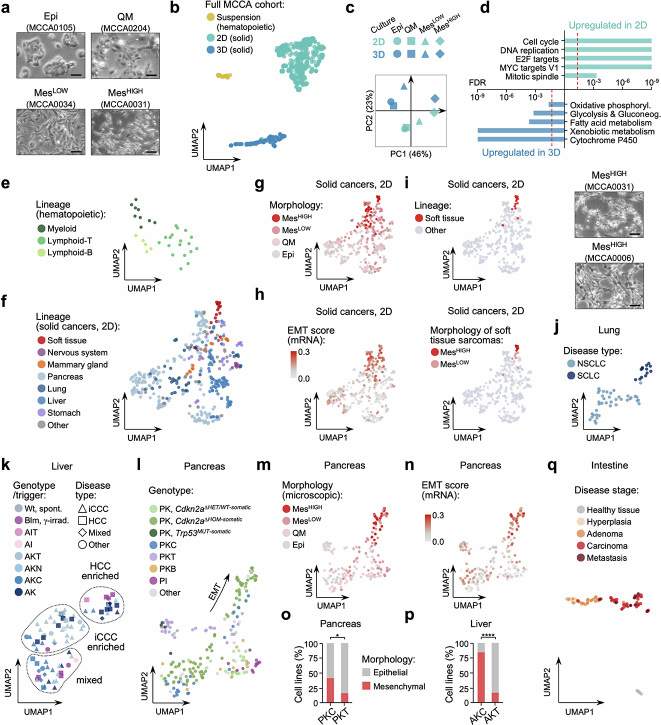
Characterization of the MCCA and integrative analyses of various data layers. **a**, Exemplary microscopic images showing the spectrum of morphologies observed in solid cancer cell lines grown in 2D conditions (n = 439). The panel shows representative images of MCCA lines with epithelial (Epi), quasimesenchymal (QM, hybrid state), weak mesenchymal (Mes^LOW^, low spindle-shaped cell morphology) or strong mesenchymal morphology (Mes^HIGH^, high spindle-shaped cell morphology). Scale bars, 25 µm. **b**, Transcriptome-based UMAP providing an overview on the clustering of cell lines across the MCCA cohort. The culture type of each MCCA line is annotated: 2D (solid cancers, grown as adherent monolayer), 3D (solid cancers, grown as organoid), or suspension (hematopoietic cancers, grown as non-adherent suspension). **c,d**, Assessing the effect of 2D versus 3D culture conditions on global transcriptomes and EMT states of MCCA lines. Four hepatic cancer lines of MCCA covering the full spectrum of EMT states were cultured in either 2D or 3D Matrigel conditions and profiled by RNA-seq (Epi, epithelial; QM, quasi-mesenchymal; Mes^LOW^, Mes^HIGH^, mesenchymal with low/high spindle-shaped cell morphology). Principal component analysis (PCA) shows culture condition-induced transcriptome changes (**c**). Pathways upregulated in 2D versus 3D culture conditions were determined by gene set enrichment analysis and revealed predominant effects on proliferative and metabolic programs, but not EMT (**d**) (Supplementary Table 3). **e-q**, Transcriptome-based UMAPs showing the context-dependence of transcriptome clustering for indicated subsets of MCCA lines, considering distinct phenotypic and molecular contexts. **e,f**, Cellular **lineage** identity, including hematopoietic lines (**e**) or solid cancer lines grown in 2D conditions (excluding 3D organoids) (**f**). **g-i, Morphology**, shown for 2D solid cancer lines of as classified by microscopy into progressive EMT states Epi>QM>Mes^LOW^>Mes^HIGH^ (Epi, epithelial; QM, quasimesenchymal, hybrid state; Mes^LOW/HIGH^, mesenchymal with low/high spindle-shaped cell morphology) (**g**); or classified by transcriptional EMT state based on EMT scores determined by gene set variation analysis using the MSigDB EMT hallmark gene set (**h**). The precise recording of EMT phenotypes in MCCA will allow users to account for this potential ‘confounder’, for example when comparing cell line transcriptomes – a consideration that is likely also relevant to human cell line resources. **i**, Transcriptome-clustering (**top**) and morphology (**bottom**) of MCCA soft tissue sarcoma cell lines (n = 18). UMAP is corresponding to (**f-h**). Representative microscopic images of MES^HIGH^ sarcoma cell lines are shown on the right. Scale bars, 25 µm. **j,k, Disease type**, shown for lung cancers (NSCLC, non-small cell lung cancer; SCLC, small cell lung cancer) (**j**), and hepatic cancer cell lines (Disease type: iCCC, intrahepatic cholangiocellular carcinoma; HCC, hepatocellular carcinoma; Genotype/trigger: Blm, *Bloom*^∆^; A, Alb-Cre; I, *Rosa26*^*PIK3CA-H1047R*^; K, *Kras*^*G12D*^; T, *Trp53*^∆^; N, *Pten*-sgRNA; C, *Cdkn2a*^∆^ or *Cdkn2a*-sgRNA) (**k**). Note: Disease type can be one driver of transcriptome clustering in the liver as indicated by the differential enrichment of iCCC and HCC liver cancer cell lines in two transcriptomic clusters (*P = 0.0437, two-sided Chi-squared test). **l, Genotype** (encompassing engineered and somatic alterations), shown for pancreatic cancers. Co-clustering of PK with PKC or PKT cell lines can be explained by their somatic acquisition of *Cdkn2a* or *Trp53* alterations, respectively. PKB cell lines cluster with a subset of PK cells that are characterized by genetic *Cdkn2a* proficiency (cluster in the lower right) – an alteration profile shared by the PKB model^18^. Vertical spread of some genotypes is linked to the differential EMT/morphology status of individual cell lines (see also **m,n**). Genotype: P, *Panc*^*Cre*^ or *Panc*^*Flp*^; K, *Kras*^*G12D*^; I, *Rosa26*^*PIK3CA-H1047R*^; C, *Cdkn2a*^∆^; T, *Trp53*^∆^; B, *Tgfbr2*^∆^. **m,n**, Transcriptome-based UMAPs showing the clustering of MCCA pancreatic cancer lines, considering their microscopy-based morphology (Epi, epithelial; QM, quasimesenchymal, hybrid state; Mes^LOW/HIGH^, mesenchymal with low/high spindle-shaped cell morphology) (**m**), or transcriptional EMT state (EMT scores based on gene set variation analysis using the MSigDB EMT hallmark gene set) (**n**). **o,p**, Bar plots showing the effect of mouse genotype on cell line morphology as exemplified in *Kras*^*G12D*^-driven pancreatic (**o**) and liver cancer cell lines (**p**) of MCCA with *Cdkn2a* or *Trp53* inactivation (PKC, n = 22; PKT, n = 32; AKC, n = 19; AKT, n = 25). Genotype/trigger: P, *Panc*^*Cre*^; A, Alb-Cre; K, *Kras*^*G12D*^; C, *Cdkn2a*^∆^; T, *Trp53*^∆^. *P = 0.0372, ****P < 0.0001, two-sided Chi-squared test. **q**, Evolutionary **disease stage**, shown for serrated, intestinal cancer evolution.

For each cell line, we provide multiple data layers in the MCCA, including some not systematically captured in human collections (Fig. 1a,b). First, we sequenced MCCA lines and generated genomic and transcriptomic profiles using our computational pipelines specifically tailored to the mouse genome (MoCaSeq)^13,14^. Second, we assembled clinical metadata, such as survival and metastasis. Third, after microscopy-based grading of cellular morphology, we assigned each line to one of four distinct epithelial-to-mesenchymal transition (EMT) states (Extended Data Fig. 2a). Fourth, we performed histopathological classification of tumour tissue related to individual MCCA lines (Supplementary Table 1). MCCA therefore represents a comprehensive cell line (and related data) resource for the mouse—the most widely used experimental model in biomedical research.

## Integrative analyses of MCCA data

When evaluating MCCA’s use in examining the relationships between molecular and phenotypic data, we observed that separation of transcriptomes is driven by various parameters, including cell lineage, cell state, disease type within a lineage/organ, genotype, disease stage and culture conditions (Extended Data Fig. 2a–q). To facilitate such integrative analyses of molecular, cellular, organismal and temporal data layers, we made all MCCA data accessible through a user-friendly mouse-adapted cBioPortal^15–17^ web interface (www.mcca.tum.de). Exemplary data mining is showcased by correlating pancreatic cancer phenotypes, such as survival or metastasis, with molecular data (Supplementary Video 1).

**Extended Data Fig. 3:**
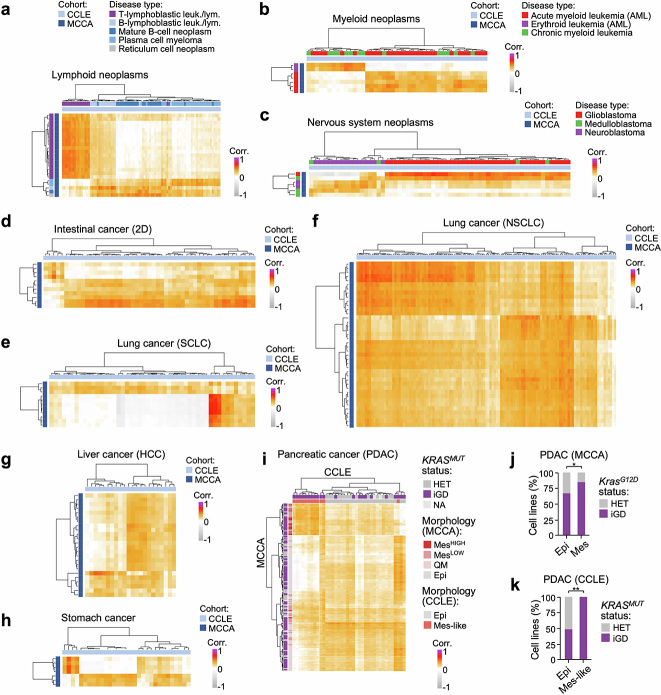
Cross-species comparison of cancer transcriptomes in mice (MCCA) and humans. **a-i**, Transcriptome similarity of MCCA (mouse) and CCLE (human) cancer cell lines. Heatmaps show unbiased hierarchical clustering of Pearson correlation coefficients derived from the pairwise comparison of mouse and human cell line transcriptomes for indicated cancer types. For intestinal cancers, only cell lines cultured as adherent monolayer (2D) were compared. Pearson correlation coefficients were calculated based on the top 10% most variable expressed orthologous genes within each lineage or entity (see Methods and Supplementary Table 4). An extended list of Pearson correlation coefficients for the comparison of MCCA to CCLE lines, and of CCLE to MCCA lines within the respective lineages or disease types are also provided in Supplementary Table 4. Combining the mouse/human transcriptome correlation analyses with the broad molecular and phenotypic annotation of MCCA lines will guide users to select mouse cell lines representing defined human cancer subtypes/models – and vice versa. SCLC, small cell lung cancer; NSCLC, non-small cell lung cancer; HCC, hepatocellular carcinoma; PDAC, pancreatic ductal adenocarcinoma. Corr., Pearson correlation coefficient. **a-c**, Entity-specific clustering of indicated mouse and human neoplasms. Mouse cancer subtypes co-cluster with their respective human counterparts. Leuk., leukaemia; lym., lymphoma. **d-i**, Within disease type comparison of indicated mouse and human cancers. For pancreatic cancer (PDAC) (**i**), mesenchymal mouse and human cancer cell lines co-cluster and are both enriched for increased *KRAS*^*MUT*^ gene dosage (see also **j,k**). Human cell line morphology was classified based on epithelial/mesenchymal marker gene expression (see Methods). PDAC, pancreatic ductal adenocarcinoma; *KRAS*^*MUT*^, *KRAS* G12* or Q61*; HET, heterozygous; iGD, increased gene dosage; NA, unknown; Epi, epithelial; QM, quasimesenchymal; Mes^LOW/HIGH^, mesenchymal with low/high spindle-shaped cell morphology. **j,k**, Comparison of *Kras*^*G12D*^ or *KRAS*^*MUT*^ gene dosage status in epithelial versus mesenchymal pancreatic ductal adenocarcinoma (PDAC) cell lines of MCCA (**j**) or CCLE (**k**), respectively. Acquisition of increased *Kras*^*G12D*^ gene dosage is more frequent in mouse PDAC cell lines with mesenchymal morphology as compared to epithelial lines – as previously reported by us^18^. Similarly, *KRAS*^*MUT*^ (G12* or Q61*) gene dosage increase is enriched in human PDAC cell lines with mesenchymal-like morphology. (Supplementary Table 4; MCCA: 84% versus 67%, *P = 0.0467, Chi-squared test; CCLE: 100% versus 48%, **P = 0.0057, two-sided Chi-squared test). For the analysis of MCCA cell lines in (**j**), *Kras*^*G12D*^-mutant pancreatic cancer with or without engineered genetic alterations in PDAC hallmark genes *Cdkn2a*, *Trp53* or *Tgfbr2* derived from independent cancers were included in the analysis. The morphology of human PDAC cell lines (**k**) was classified based on epithelial/mesenchymal marker gene expression (see Methods).

**Extended Data Fig. 4:**
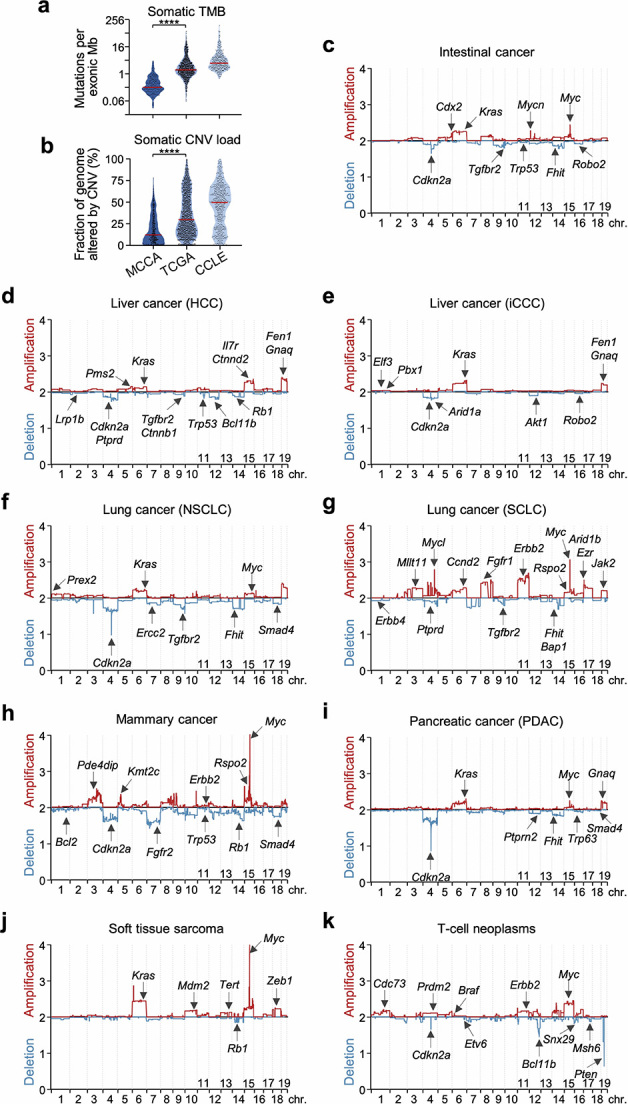
Cross-species comparison of cancer genomes in mice (MCCA) and humans. **a,b**, Tumour mutational burden (TMB, somatic) (**a**) and copy number variation (CNV) load (**b**) across MCCA, TCGA and CCLE cohorts. Stroma cell contributions in TCGA tissue data were corrected using cancer cell purity estimates. Only human cancer cohorts matching to MCCA cancer types were analysed (see Methods). ****P < 0.0001, two-sided Mann-Whitney test; bars, median. **c-k**, Consensus copy number profiles of nine MCCA cancer entities, annotated with the orthologues of genes that are recurrently amplified or deleted in the corresponding human cancer type. MCCA cancer types with sufficiently large numbers of samples and alterations for meaningful detection of recurrent copy number alterations are shown. Consensus profiles were generated by binning the genome and calculating the average copy number change at each genomic bin across all cancers of a given disease type. Y axis shows the averaged copy number value of a genomic region relative to a diploid genome (Amplification, >2; Deletion, <2). For the annotation of human cancer gene orthologues, all genes affected by recurrent copy number alterations in individual human cancer types (CCLE and TCGA datasets) were first assembled and filtered for those reported in the Cancer Gene Census. From these genes, a representative set of cancer genes was then selected for each disease type, based on gene amplification/deletion frequency and literature search. Overall, these profiles highlight that mouse and human cancers share critical disease-specific genomic alterations. Selected explanatory notes: (**i**) In pancreatic cancer, genes frequently affected by copy number alterations in the human disease (e.g. *KRAS*, *MYC*, *CDKN2A*, *TP63*, *SMAD4*) are also altered in mice: while *SMAD4* deletions are less prevalent in mice than in humans, *KRAS* amplifications and *CDKN2A* deletions are frequent in both species. (**c**) Note that in intestinal cancer copy number changes affecting *APC* or *CTNNB1* are infrequent, as both mouse genes are typically altered by mutations (see Fig. 4f), as in humans. (**f,g**) Mouse small cell lung cancers (SCLC) and non-small cell lung cancers (NSCLC) show distinct copy number alterations, as in humans. For example, amplifications of *MYCL* are frequent in mouse and human SCLC, but not in NSCLC. Moreover, both SCLC and NSCLC show amplifications of chromosome 6, but the oncogenic drivers of this event are likely different: amplification of the oncogenic *Kras*^*G12D*^ knock-in allele in NSCLC versus *Ccnd2* gain in SCLC (which are *Kras*-wildtype). HCC, hepatocellular carcinoma; iCCC, intrahepatic cholangiocarcinoma; NSCLC, non-small cell lung cancer; SCLC, small cell lung cancer; PDAC, pancreatic ductal adenocarcinoma.

To examine cross-species relationships, we compared transcriptomes of MCCA and the human CCLE using a correlation-based approach (Methods and Extended Data Fig. 3a–i). For example, among lymphoid cancers, MCCA T cell neoplasms cluster with human T cell leukaemia/lymphoma, while mouse B cell neoplasms align with their human counterparts, including B lymphoblastic leukaemia/lymphoma, multiple myeloma or mature B cell neoplasms. Equivalent analyses within an entity are shown for pancreatic cancer, where mouse and human cell lines with a mesenchymal phenotype and increased dosage of mutant *KRAS* co-cluster—consistent with oncogenic dosage increase promoting EMT and a basal-like differentiation with poor prognosis^18,19^. These and similar data for other cancer types (Extended Data Fig. 3a–k) show the broad spectrum of human disease phenotypes and molecular contexts covered by the MCCA. To support the identification of MCCA counterparts of human disease subtypes or CCLE models, we provide Pearson correlation coefficients for all mouse–human cell line pairs (Supplementary Table 4) along with broad molecular and phenotypic annotations (Supplementary Table 1) and further cross-species comparisons at the genomic level (Extended Data Fig. 4a–k).

## MCCA immunophenotyping

Immunocompetent transplantation models are of growing importance for biomedical research and preclinical drug testing, especially as immunotherapy landscapes expand at an rapid pace. As cancer cell lines often originate from models with mixed genetic backgrounds, matching donor–recipient immunocompatibility requires immunophenotyping. In principle, relevant information (MHC haplotypes, genetic background, sex) can be obtained from genomic sequencing data, but analytical tools are lacking. We therefore developed methodological approaches and computational tools addressing this need (Methods).

**Fig. 2:**
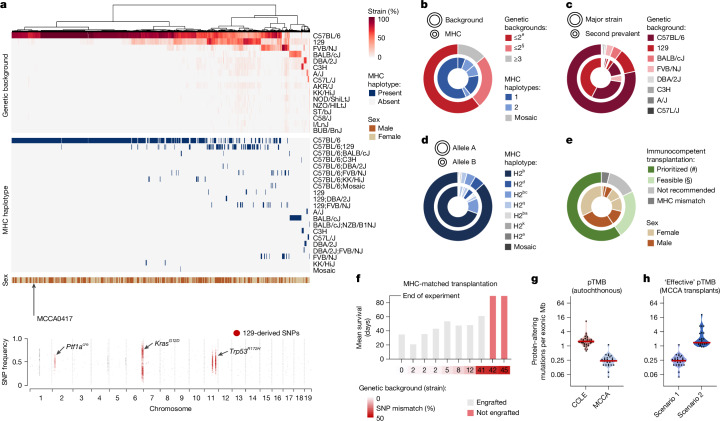
Immunophenotyping of MCCA and application for immunocompetent transplantation studies. **a**, Inference of strain background composition, MHC haplotypes and sex for MCCA lines from genome sequencing data. Top, overview of MCCA immunophenotypes. Cell lines are ordered by unsupervised hierarchical clustering of genetic background composition. Bottom, genome-wide SNP plot of a C57BL/6-backcrossed cell line (MCCA0417). Note the persistence of the 129-mouse signature SNPs in proximity to genetically engineered alleles. **b**–**e**, Immunophenotype characteristics across the MCCA. **b**, The distribution of genetic backgrounds and corresponding MHC haplotypes. Prioritized (#) and feasible (§) lines are defined in the caption for **e**. **c**, Genetic background (strain composition) per cell line, considering the two highest-ranking strains only. **d**, MHC haplotype distribution for both alleles (A,B) per cell line. **e**, Choice of MCCA transplant recipients: recommendations are indicated for each MCCA line based on their genetic background(s), MHC haplotype(s) and sex information. Prioritized (#), MCCA lines with one or two genetic backgrounds (in a few cases, with a third genetic background contributing <1%; exemplary transplant scenarios are shown in **h**). Feasible (§), MCCA lines with two dominant genetic backgrounds and a third background contributing 1–9% of SNPs (exemplary transplant scenarios are shown in Extended Data Fig. 6f). Not recommended, MCCA lines with three dominant backgrounds. **f**, Survival of immunocompetent mice transplanted with MHC-matched mPACA lines with various degrees of strain SNP mismatch (*n* = 63 transplantations). Only mPACA lines with incomplete *Cdkn2a* inactivation were compared. **g**,**h**, TMB (considering protein-changing alterations, pTMB) of MCCA lines in different experimental contexts, shown for PACA lines with C57BL/6-derived MHC and C57BL/6;129-SNP contributions (*n* = 27). **g**, In autochthonous tumours, somatic protein-altering mutations define pTMB. Human PACA lines are displayed for comparison (CCLE, *n* = 41). **h**, In MHC-matched transplantations using the corresponding cell lines, the ‘effective’ pTMB is recipient dependent. Scenario 1 (C57BL/6;129-F1 recipients): only somatic mutations contribute to ‘effective’ pTMB. Scenario 2 (C57BL/6 recipients): somatic mutations and 129-strain-specific germline variants can be immunogenic (increased ‘effective’ pTMB).

**Extended Data Fig. 5:**
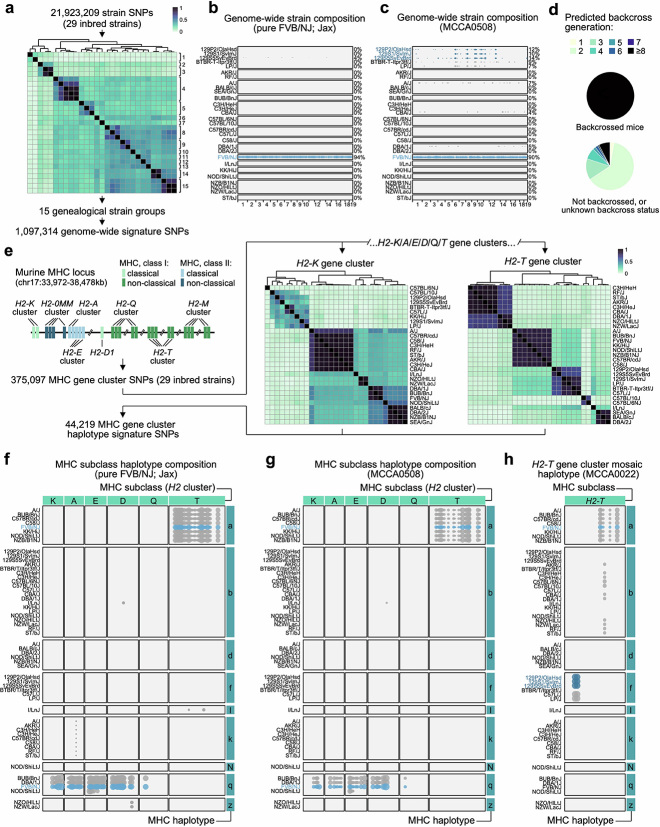
Analytical tools for immunophenotyping of MCCA lines using genomics data. **a**, Schematic workflow illustrating the identification of mouse strain-specific signature SNPs for genome-wide detection of genetic background composition from genomic sequencing data. First, a total of 21,923,209 SNPs were extracted from the whole genome sequencing catalogue of the Mouse Genomes Project comprising 29 inbred mouse strains^20^. Next, these SNPs were used to compute the Pearson correlation of SNP patterns across mouse strains (heatmap). Based on the hierarchical clustering of these 29 inbred strains, 15 genealogically-related strains groups were defined. Finally, a total of 1,097,314 strain signature SNPs were determined based on variants that are mutually exclusive between strain groups (while signature SNPs are allowed to be shared within the same strain group). The enrichment or absence of strain-specific signature SNPs was subsequently used for precise analysis of strain composition across the entire genome. **b,c**, Bubble plots showing the genome-wide strain composition for a given sample. Percent genomic enrichments of signature-SNPs for each of the 29 inbred mouse strain is annotated on the right. Boxes indicate the 15 groups of genealogically-related strains. **b**, Strain composition in a pure FVB/NJ mouse as determined by whole exome sequencing (MMR-4805, sequencing data obtained from the Jackson Laboratory). The FVB/NJ strain is highlighted in blue. **c**, Analysis of strain composition for cell line MCCA0508 of the MCCA resource which was isolated from a mouse with unknown genetic background. An enrichment of FVB/NJ (90%) and 129-related strains (10–14%) was found, based on whole exome sequencing. The FVB/NJ strain and 129-related strains are highlighted in blue. **d**, Computational backcross generation for MCCA pancreatic cancer cell lines derived PK mice with distinct backcross status. Backcross generation was inferred from the strain composition of a given cell line as detected in genomic sequencing data (see Methods). All cell lines derived from backcrossed pancreatic cancer mouse colonies were identified as ‘highly backcrossed’ (≥ 8 generations, n = 11), which corresponds to less than 1% non-dominant genetic background contribution. In contrast, cell lines with unknown backcross status (n = 105) showed a mixed level of backcrossing. Corresponding information across the MCCA cohort is provided in Supplementary Table 5. **e**, Schematic workflow visualizing the identification of major histocompatibility complex (MHC) signature SNPs for MHC haplotype classification from genomic sequencing data. For this, the murine MHC locus was first divided into 6 gene clusters (*H2-K*, *-A*, *-E*, *-D*, *-Q* and *-T*) based on their MHC subclass assignment (class I or II, classical or non-classical). Data from the whole genome sequencing catalogue of the Mouse Genomes Project comprising 29 inbred mouse strains^20^ was used to examine the SNP composition at MHC gene clusters and to derive a total of 375,097 SNPs. Next, these MHC-related SNPs were used to compute Pearson correlations and to perform hierarchical clustering of SNP patterns for each MHC gene cluster across the 29 inbred strains. MHC haplotype assignment and signature SNP identification were conducted for each MHC gene cluster individually, as the combination of MHC gene cluster haplotypes do not need to be conserved across strains within the same genealogical strain group. These analyses resulted in a set of 44,219 mutually exclusive MHC signature SNPs. Ultimately, MHC-specific signature SNPs were utilized to infer the haplotype for each of the 6 MHC gene clusters – which in combination defines the full MHC haplotype. **f-h**, Bubble plots showing the enrichment of MHC-specific signature SNPs across MHC gene clusters (*H2-K*,*-A*,*-E*,*-D*,*-Q*,*-T*) and for distinct MHC haplotypes (a,b,d,f,I,k,N,q,z). The MHC haplotypes follow the lowercase letter nomenclature, except ‘I’ and ‘N’ which were specifically defined in this study for strains I/LnJ and NOD/ShiLtJ, respectively. Horizontal boxes represent groups of inbred mouse strains with identical haplotypes in at least one MHC gene cluster. Of note, individual strains can be annotated multiple times in the MHC haplotype plot. This is because the assignment of inbred strains into MHC haplotype groups does vary across the 6 defined MHC gene clusters (as explained in (**e**)). **f**, MHC haplotype composition of each MHC gene cluster is shown for a pure FVB/NJ mouse as determined by whole exome sequencing (MMR-4805, sequencing data obtained from the Jackson Laboratory; same sample as shown in (**b**)). The MHC haplotype combination for this sample was predicted to be ‘q-q-q-q-q-a’, which is line with to the available MHC annotations for the FVB/NJ strain. Bubbles corresponding to FVB/NJ-specific MHC gene cluster haplotypes are highlighted in blue. **g**, Analysis of MHC haplotype composition for cell line MCCA0508 of the MCCA resource which was isolated from a mouse with unknown genetic background. The MHC haplotype composition was determined to be ‘q-q-q-q-q-a’ as inferred from whole exome sequencing data. This MHC locus haplotype is consistent with the high enrichment of FVB/NJ-specific strain SNPs in this sample (**c**). Bubbles corresponding to FVB/NJ-specific MHC gene cluster haplotypes are highlighted in blue. **h**, MHC haplotype composition at the MHC gene cluster *H2-T* in MCCA line MCCA0022 based on low coverage whole genome sequencing (lcWGS). The bubble plot shows a mosaic MHC haplotype at *H2-T* with enrichment for 129- (‘f’ haplotype at the 5′-end) and FVB/NJ-specific (‘a’ haplotype at the 3′-end) signature SNPs. Such mosaic MHC haplotypes are rarely observed in MCCA (0.5%) and are generated de novo through meiotic crossover events at the MHC locus in mouse cohorts maintained on a mixed genetic background. 129- and FVB/NJ-related SNP enrichment is highlighted in blue.

First, for strain background detection, we extracted single-nucleotide polymorphisms (SNPs) for 29 inbred mouse strains using Mouse Genomes Project^20^ data. By correlating SNP patterns between strains, we identified 15 clusters, which we defined as genealogically related strain groups (Extended Data Fig. 5a). This assignment was critical for delineating strain-specific signature SNPs (SNPs unique for each of the 15 strain groups but allowed to be shared within the same group). These signature SNPs (*n* = 1,097,314) enabled highly accurate detection of strain composition (Fig. 2a–c and Extended Data Fig. 5b,c). Supplementary Table 5 lists corresponding data for all of the MCCA lines. Notably, non-dominant genetic backgrounds can remain detectable despite extensive backcrossing, as exemplified for cell line MCCA0417, which was derived from a C57BL/6-backcrossed mouse carrying the *Ptf1a*^*cre*^, *Kras*^*LSL-G12D*^ and *Trp53*^*LSL-R172H*^ alleles originally engineered in 129-related stem cells. Owing to genetic linkage, 129 signature SNPs in close genomic proximity to engineered alleles ‘withstand’ backcrossing to C57BL/6 mice (Fig. 2a), thereby contributing around 4% 129 background. Such effects are critical for estimating backcrossing status from genomic data (Methods, Extended Data Fig. 5d and Supplementary Table 5).

Second, for MHC haplotype detection, we divided the MHC locus into six gene clusters (*H2-K, -A*, *-E*, *-D*, *-Q* and *-T*) on the basis of MHC subclasses. Precise classification of MHC clusters is crucial for preventing T cell-mediated transplant rejection. We correlated SNP data for each gene cluster to assign 29 inbred strains into genetically conserved MHC subclass haplotypes, defined by 44,219 signature SNPs (Extended Data Fig. 5e). These MHC signature SNPs were used to determine MHC-subclass-specific haplotypes, which enabled us to define the combined/full MHC haplotype (Extended Data Fig. 5f,g). Overall, 83% of cell lines possess MHC alleles from C57BL/6-related (H2^b^) and/or 129-related (H2^bc^) strains (Fig. 2d). In rare cases, additional complexity can arise from meiotic crossover events in mouse cohorts with mixed genetic backgrounds. For example, we detected mosaic MHC haplotypes generated through recombination of 129- and FVB-derived MHC *H2-T* gene clusters (Fig. 2d and Extended Data Fig. 5h).

**Extended Data Fig. 6:**
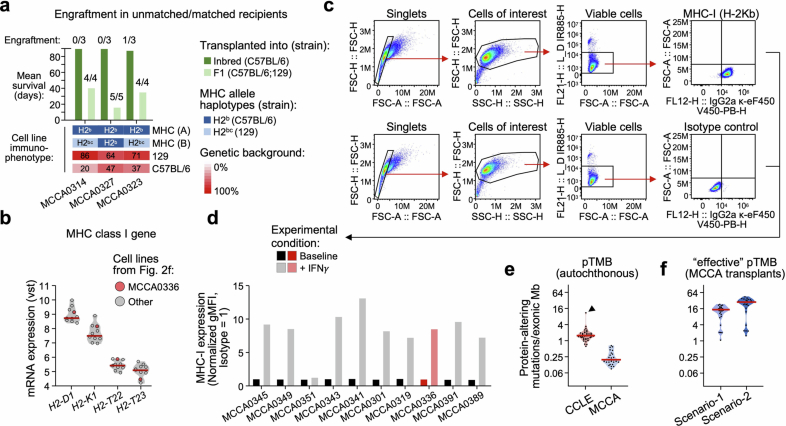
Relevance of genetic background, MHC-I haplotype and ‘effective’ pTMB of MCCA lines in immunocompetent transplantations. **a**, Engraftment of mouse pancreatic cancer cell lines with indicated MHC haplotypes and strain contributions from C57BL/6 and 129 backgrounds when transplanted into immunocompetent C57BL/6 or C57BL/6;129-F1 hybrid mice. After their transplantation into C57BL/6 recipients, engraftment was observed in only one out of nine cases and occurred with long latency (66 days, MCCA0323). In contrast, all three cell lines engrafted when transplanted into immunophenotype-matched C57BL/6;129-F1 hybrid mice (13/13 transplantations) and formed pancreatic cancers within 16–42 days. These exemplary data show that MCCA lines carrying MHC haplotypes and/or high strain contributions from two genetic backgrounds can engraft in corresponding F1 hybrid recipients. Note that MCCA lines might occasionally not engraft in fully immunophenotype-matched hosts due to non-immunological reasons, such as the lack of specific niche factors. These effects are especially relevant when transplanting low cell numbers (< 10,000), which can however be rescued by increasing the number of injected cells. **b**, mRNA expression of MHC class I genes (*H2-D1*, *H2-K1*, *H2-T22*, *H2-T23*) in transplanted pancreatic cancer cell lines from Fig. 2f. MCCA0336 (highlighted in red), which engrafted despite high strain mismatch (Fig. 2f), does not show down-regulation of MHC class I gene expression in comparison to all other pancreatic cancer cells lines. Only MHC class I genes robustly expressed in this set of cell lines are shown (median vst expression >5). Batch-corrected, variance-stabilizing transformed (vst) gene expression values are shown because transcriptomes originate from distinct RNA-seq batches of MCCA. **c**, Flow cytometry gating strategy for the quantification of cell surface MHC class I protein expression. Sequential gating illustrates the selection of singlets, cells of interest, and viable cells. **Top:** Staining with anti-MHC class I antibody (H-2Kb). **Bottom:** Isotype control (IgG2a κ). **d**, Bar plot depicting cell surface MHC class I protein levels for transplanted pancreatic cancer cell lines from Fig. 2f, either untreated (baseline) or stimulated with IFN-γ (100 ng/mL, 3 days). Neither baseline nor IFN-γ-stimulated MHC levels of MCCA0336 were reduced in comparison to matched/engrafted or mismatched/non-engrafted pancreatic cancer cell lines of Fig. 2f. Order of cell lines is identical to Fig. 2f. **e,f**, Tumour mutational burden of non-synonymous (protein-altering) mutations (pTMB) in human (CCLE, n = 41) and mouse pancreatic cancer cell lines (MCCA, n = 27). MCCA lines with homozygous C57BL/6 MHC haplotype, C57BL/6;129 dominant genetic background and a 3^rd^ genetic background contributing 1–9% of SNPs (which typically display higher germline SNP burdens) were selected to illustrate how these cell lines can be used to model high mutational burden. Compare to Fig. 2g,h where MCCA pancreatic cancer cell lines with only C57BL/6;129 strain contribution are shown. In the human or mouse autochthonous setting, somatic protein-altering mutations define the pTMB (**e**). Arrowhead indicates human pancreatic cancer cell line SNU324 which shows a high pTMB of 11.1 due to micro-satellite instability caused by a homozygous *MSH2* missense mutation. In MHC-matched transplantations, the ‘effective’ pTMB of the selected MCCA lines is recipient-dependent (**f**). Scenario-1: transplantation into C57BL/6;129-F1 hybrid mice, somatic mutations and strain-specific germline variants of the 3^rd^, non-dominant strain contribute to the ‘effective’ pTMB. Scenario-2: transplantation into C57BL/6 mice, somatic mutations and strain-specific germline variants of the 3^rd^ as well as the 129 genetic background can be immunogenic, thereby further elevating the ‘effective’ pTMB.

Third, we determined immunophenotypes for all cell lines by combining genetic background (SNP composition), MHC haplotype and sex information (Supplementary Table 5). We found that 60% of cell lines possess immunophenotypes from one or two inbred strains, allowing transplantation into one strain or matched F_1_ hybrid mice (most commonly 129;C57BL/6; Fig. 2c,e and Extended Data Fig. 6a). Importantly, most entities are represented in this group. The remaining lines had immunophenotype contributions from more than two strains. Although donor–recipient matching is still possible at the MHC level, SNP mismatching could affect transplantability. However, in 56% of cases (23% of the MCCA), the third-background SNP contribution is less than 10%, which is often tolerated in transplantation experiments. To exemplify this, we performed MHC-matched transplantations (Fig. 2f). As expected, cell lines with the highest SNP mismatch to recipients (42% and 45%) did not engraft, whereas lines with ≤12% mismatch engrafted robustly. Notably, one line with 41% mismatch and intact MHC expression/competence (Extended Data Fig. 6b–d) formed cancers, albeit with long survival.

Overall, these results highlight the importance of annotating MCCA immunophenotypes, which will guide precise recipient selection in future studies (Supplementary Table 5).

## Somatic and germline variants in MCCA

In transplantation experiments, not only somatic mutations in cell lines, but also strain-specific germline variants, can contribute to tumour mutational burden (TMB) and immunogenicity, depending on the recipient. This is illustrated by transplanting pancreatic cancer cell line MCCA0349 into distinct MHC-matched recipients. MCCA0349 was derived from a C57BL/6-backcrossed mouse with *Ptf1a*^*cre*^ and *Kras*^*LSL-G12D*^ alleles originally engineered in the 129 background. Its MHC haplotype is therefore C57BL/6, but 129-associated SNPs in proximity to *Ptf1a*^*cre*^ and *Kras*^*LSL-G12D*^ ‘withstood’ backcrossing.

In the first scenario, MCCA0349 is transplanted into C57BL/6;129-F1 hybrid mice. Here, only somatic mutations (*n* = 26 protein-altering mutations, yielding a protein-altering TMB (pTMB) of 0.4 per Mb exome), but not strain-specific germline variants, contribute to immunogenicity and pTMB. Equivalent data for other pancreatic cancer cell lines with similar MHC and strain characteristics (Fig. 2g,h) show that this transplantation scenario exhibits a lower pTMB compared with human cancers.

In the second scenario, MCCA0349 is transplanted into C57BL/6 mice. Here, the ‘effective’ pTMB amounts to 103 protein-altering mutations (1.4 per Mb exome; 26 somatic mutations plus 77 129-related SNPs). Modelling this scenario for all MCCA pancreatic cancer lines with similar MHC and strain contributions (Fig. 2h) revealed that the majority possesses an ‘effective’ pTMB comparable to human cancers (Fig. 2g). These results corroborate previous observations that syngeneic transplant models often respond better to immunotherapies compared with their autochthonous counterparts^12^.

Notably, in scenario 2, the MCCA lines with the highest 129-strain contributions displayed ‘effective’ pTMB levels similar to the *MSH2*-mutant human line SNU324. Moreover, considering MCCA lines with over two genetic backgrounds for MHC-matched transplantations gives further flexibility to create experimental settings with high mutational burden (Extended Data Fig. 6e,f). Thus, strain-specific germline variants can be exploited to design immunocompetent transplantation experiments with desired levels of ‘effective’ pTMB (Supplementary Table 6).

## *KRAS* gene dosage variation across entities

**Fig. 3:**
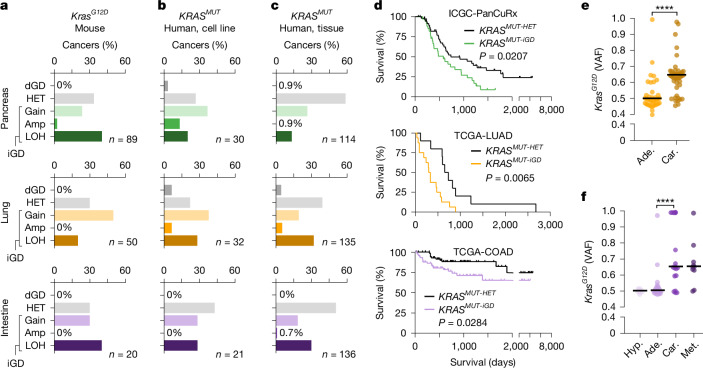
Frequency, clinical relevance and timing of *KRAS*^*M*^^*UT*^ allelic imbalance across tissues. **a**–**c**, Inference of *KRAS*^*MUT*^ allelic status in mouse and human pancreatic, lung and intestinal cancer cohorts using genomics data. Three main *KRAS*^*MUT*^ allelic states were defined as dGD (decreased gene dosage, allelic imbalance favouring the WT allele), HET (heterozygous *KRAS*^*MUT*^) and iGD (increased *KRAS*^*MUT*^ dosage) (Methods; examples are provided in Extended Data Fig. 7a). Enrichment of *KRAS*^*MUT-iGD*^, but not *KRAS*^*MUT-dGD*^, across entities and species demonstrates positive selection of increased gene dosage (*P *≤ 0.0001, two-sided Mann–Whitney *U*-test). Amp, high-level amplification. **a**, MCCA lines and tissues derived from genetically engineered mouse models: *Ptf1a*^*cre/+*^*;Kras*^*LSL-G12D/+*^ (pancreas), *Ela-creER*^*TM*^*;Kras*^*LSL-G12D/+*^ (lung) and *Vil-cre;Kras*^*LSL-G12D/+*^ (intestine). **b**, Human cancer cell lines from pancreas (CCLE-PAAD), lung (CCLE-NSCLC) and intestine (CCLE-COAD). **c**, Human cancer tissues from pancreas (ICGC-PanCuRx), lung (TCGA-LUAD) and intestine (TCGA-COAD). **d**, Disease-specific survival of patients with *KRAS*^*MUT-HET*^ versus *KRAS*^*MUT-iGD*^ cancers (top, pancreatic cancers; middle, lung cancers; bottom, intestinal cancers). TCGA-LUAD survival including event censoring is shown in Extended Data Fig. 7b. *P* values were calculated using two-sided log-rank tests. **e**, *Kras*^*G12D*^ allelic imbalance at defined stages of lung cancer evolution in *Ela-creER*^*TM*^*;Kras*^*LSL-G12D/+*^ mice. Purity-corrected *Kras*^*G12D*^ allele frequencies of adenomas (ade; *n* = 40) and carcinomas (car; *n* = 44) microdissected from MCCA tissues. Statistical analysis was performed using two-sided Mann–Whitney *U*-tests; ****P < 0.0001. **f**, *Kras*^*G12D*^ allelic imbalance in MCCA organoids isolated at defined stages of serrated, intestinal cancer evolution from *Vil-cre;Kras*^*LSL-G12D/+*^ mice: hyperplasia (hyp; *n* = 9), adenoma (*n* = 24), carcinoma (*n* = 20) and metastasis (met;* n* = 10). For **e** and **f**, statistical analysis was performed using two-sided Mann–Whitney *U*-tests; ****P < 0.0001. VAF, variant allele frequency.

**Extended Data Fig. 7:**
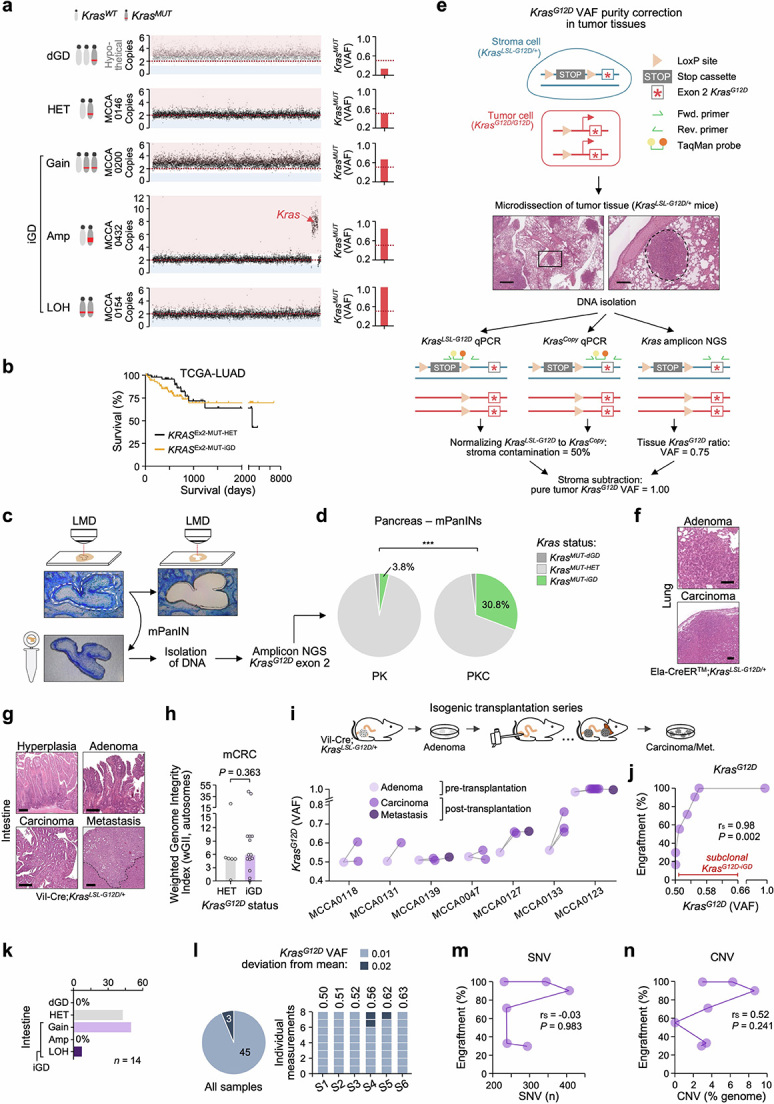
Allelic states of *KRAS*^*MUT*^ dosage variation, their detection in mouse tissues and their role for driving cancer evolution. **a**, Overview of *Kras*^*G12D*^ allelic ‘states’ as defined in this study. For each individual *Kras*^*G12D*^ allelic status, exemplary CNV plots and corresponding *Kras*^*G12D*^ variant allele frequencies (VAF) are shown for representative MCCA lines. The hypothetical dGD example (in grey) was not found in MCCA lines and is rarely detected in human cancer cohorts. dGD, decreased *Kras*^*G12D*^ gene dosage, allelic imbalance favouring the wildtype allele; HET, heterozygous *Kras*^*G12D*^; iGD, increased *Kras*^*G12D*^ dosage; gain, low-level amplification with tumour/normal copy ratio <2.8; Amp, high-level amplification; LOH, loss of heterozygosity. **b**, Disease-specific patient survival related to lung adenocarcinomas (TCGA-LUAD) with heterozygous or increased *KRAS*^*MUT*^ gene dosage (related to Fig. 3d). Survival curves (including censored events) show early separation but intercross at later timepoints due to event censoring. **c**, Experimental workflow illustrating the analysis of *Kras*^*G12D*^ allelic imbalance in single mouse PanIN lesions. Individual PanINs were excised by laser microdissection (LMD), followed by DNA isolation and amplicon-based next-generation sequencing of *Kras* exon 2 using a customized nested PCR protocol (see Methods). Developing a customized protocol for amplicon-based *Kras* exon 2 sequencing was necessary to enable the quantification of *Kras*^*G12D*^ allelic imbalance in single mouse PanINs, which are up to ten times smaller and contain fewer cells than their human counterparts. **d**, Quantification of *Kras*^*G12D*^ allelic states in laser-microdissected PanINs from *Ptf1a*^*Cre/+*^;*Kras*^*LSL-G12D/+*^ (PK) or *Ptf1a*^*Cre/+*^;*Kras*^*LSL-G12D/+*^;*Cdkn2a*^*FL/FL*^ (PKC) mice. *Kras*^*G12D*^ allelic status was assigned based on *Kras*^*G12D*^ variant allele frequencies (VAF) using identical thresholds as for all other mouse samples. In the PK model, mouse PanINs acquired *Kras*^*G12D-iGD*^, as observed earlier in humans^18^. However, the proportion of PanINs with *Kras*^*G12D-iGD*^ was low (2 of 52 mPanINs, 3.8%), consistent with the fact that most PanINs of this mouse model do not progress to invasive carcinoma. By contrast, in the PKC model 16 of 52 PanINs (30.8%) acquired *Kras*^*G12D-iGD*^, in line with earlier findings (i) that tumour progression in PK mice following acquisition of *Kras*^*G12D-iGD*^ is contingent on loss of *Cdkn2a* and (ii) that *KRAS*^*MUT-iGD*^ and early loss of *CDKN2A* occur frequent in human PanINs^18,102^. ***P = 0.0002, two-sided Chi-squared test. **e**, Scheme illustrating the inference of ‘pure’ *Kras*^*G12D*^ variant allele frequencies (VAF) in microdissected tumour tissues of MCCA. After microdissection of tumour tissues and subsequent DNA isolation, stroma cell contamination is measured by quantifying non-recombined *Kras*^*LSL-G12D*^ alleles using a custom-designed TaqMan qPCR assay. Non-recombined LSL-cassettes at the *Kras* locus are specific to stroma cells but cannot be found in tumour cells, where the LSL-cassette is recombined. To account for artifacts arising from (potential) *Kras* copy number changes in tumour cells, a second TaqMan qPCR assay for quantifying total *Kras* copy number was used to normalize *Kras*^*LSL-G12D*^ quantities. Normalized *Kras*^*LSL-G12D*^ values directly reflect stroma cell contamination of a given tumour tissue sample and were finally utilized for mathematical ‘purity’-correction of its *Kras*^*G12D*^ VAF (as determined by amplicon-based next generation sequencing of the tumour tissue sample). HE stains show an example for the microdissection of lung carcinoma tissue. NGS, next generation sequencing. Scale bars, left: 1 mm, right: 200 µm. **f**, Representative HE stains depict lung adenoma and carcinoma disease stages in Ela-CreER^TM^;*Kras*^*LSL-G12D/+*^ mice and correspond to the analysis of *Kras*^*G12D*^ allelic imbalance shown in Fig. 3e. Scale bars, 100 µm. **g**, Representative HE stains show hyperplasia, adenoma, carcinoma and metastasis disease stages of serrated, intestinal cancer evolution in Vil-Cre;*Kras*^*LSL-G12D/+*^ mice and correspond to the analysis of *Kras*^*G12D*^ allelic imbalance shown in Fig. 3f. Scale bars, 200 µm. **h**, Genomic copy number instability of intestinal carcinoma organoid lines from Vil-Cre;*Kras*^*LSL-G12D/+*^ mice. The weighted Genome Instability Index (wGII) indicates the proportion of the genome with aberrant copy number, weighted on a per chromosome basis^70^. wGII correlates with copy number instability in cancer cell lines^70^. No significant difference of wGII was observed between intestinal carcinomas with one copy of *Kras*^*G12D*^ or more than one copy of *Kras*^*G12D*^. *P*, two-sided Mann-Whitney test. **i,j**, Functional interrogation of the importance of *Kras*^*G12D*^ allelic imbalance for intestinal adenoma-to-carcinoma progression. MCCA adenoma organoids from Vil-Cre;*Kras*^*LSL-G12D/+*^ mice with varying levels of subclonal *Kras*^*G12D*^ allelic imbalance were selected for colorectal transplantations using mouse endoscopy. Adenoma organoid with highest *Kras*^*G12D*^ variant allele frequency (VAF) was used as control. **i**, *Kras*^*G12D*^ VAF in MCCA adenoma organoids (pre-transplantation) and corresponding carcinomas/metastases (post-transplantation, n = 21 organoids). Cartoon depicts experimental procedure. **j**, Correlation analysis of pre-transplantation *Kras*^*G12D*^ allelic imbalance and engraftment rates for MCCA adenoma organoid lines (n = 81 transplantations). Dots correspond to adenoma lines shown in (**i**). r_s_, Spearman correlation coefficient; *P*, two-sided *t*-test. **k**, *Kras*^*G12D*^ allelic states in carcinomas derived from transplanted intestinal adenomas with subclonal *Kras*^*G12D*^ allelic imbalance. *Kras* variant allele frequencies for individual adenomas are shown in (**i**). High-level amplifications of *Kras*^*G12D*^ were not detected (*Kras*^*G12D-Amp*^, tumour/normal copy ratio ≥2.8). Instead, all carcinomas exhibited low-level *Kras*^*G12D*^ amplification, with trisomy of *Kras*^*G12D*^ being the most frequent state (*Kras*^*G12D-Gain*^, 7 of 8 cases). Thus, subclonal *Kras*^*G12D*^ dosage increase observed in intestinal adenomas in (**i**) is based on low level amplification of *Kras*^*G12D*^. **l**, Accuracy of *Kras*^*G12D*^ variant allele frequency (VAF) quantification by amplicon-based next generation sequencing. Six samples with *Kras*^*G12D*^ VAFs between 0.50 and 0.63 were each examined by eight independent library preparations and sequencing experiments. Of 48 total measurements, 45 deviated by less than 0.01 (1%) from the expected mean. The remaining 3 quantifications deviated by <2%. Expected mean is indicated on top of the bar for each sample. This demonstrates that *Kras*^*G12D*^ -VAF quantification by amplicon-based next generation sequencing is highly accurate and suitable for determining subclonal *Kras*^*G12D*^ dosage increase during early cancer evolution. **m,n**, Correlation analysis of pre-transplantation SNV (**m**) or CNV load (**n**) with engraftment rates for MCCA adenoma organoid lines (n = 81 transplantations). Neither SNV nor CNV load of transplanted adenomas correlate with their engraftment rate in vivo. Instead, subclonal *Kras*^*G12D*^ -VAF displays marked correlation with engraftment (**j**). Dots correspond to adenoma lines shown in (**i**). SNV, single-nucleotide variant; CNV, copy number variation. r_S_, Spearman correlation coefficient; *P*, two-sided *t*-test.

*KRAS* is the most frequently mutated human oncogene^21^. While allelic imbalance at mutated *KRAS* has been shown to exacerbate oncogenic signalling^18,19,22–24^, its timing, biological consequences and genetic interaction partners in different tissues are mostly unclear. To address these questions, we analyse *Kras* allelic imbalance in MCCA lines from pancreatic (mPACA), lung (mLUCA) and intestinal (mINCA) carcinomas initiated in *Kras*^*LSL-G12D*^ mice^25^. By integrating single-nucleotide variant (SNV) and copy-number variation (CNV) data, we defined three *Kras*^*G12D*^-allelic states: decreased gene dosage (dGD), heterozygous (HET) and increased gene dosage (iGD) (Methods and Extended Data Fig. 7a). Whereas *Kras*^*G12D-iGD*^ was common across entities (Fig. 3a and Supplementary Table 7), *Kras*^*G12D-dGD*^ was not detected. To perform equivalent analyses in humans, we used cell lines^6^ and tissues from The Cancer Genome Atlas (TCGA) and the International Cancer Genome Consortium (ICGC) with *KRAS* G12* or G13* mutations (hereafter, *KRAS*^*MUT*^). For cancer tissues, we inferred pure *KRAS*^*MUT*^ gene dosage states using purity-corrected SNV and CNV values (Methods). As in mice, *KRAS*^*MUT-iGD*^ was frequent across cancer types, whereas *KRAS*^*MUT-dGD*^ was very rare (Fig. 3b,c and Supplementary Tables 8 and 9), consistent with *KRAS*^*MUT*^ increase in gene dosage being under positive selection rather than a neutral event in cancer evolution. Moreover, we found that *KRAS*^*MUT-iGD*^ was associated with reduced patient survival (Fig. 3d and Extended Data Fig. 7b). Thus, *KRAS*^*MUT-iGD*^ is of clinical relevance and is positively selected in pancreatic, lung and intestinal cancer.

## Tissue-specific timing of *KRAS*^*MUT-iGD*^

We next investigated the evolutionary timing of *KRAS*^*MUT-iGD*^ in different cancer types. For the pancreas, we found that *KRAS*^*MUT*^ gene dosage increase in mouse (Extended Data Fig. 7c,d) and human^18^ pancreatic intraepithelial neoplasia (PanIN), highlighting its acquisition during the earliest stages of PACA evolution. By contrast, acquisition of *Kras*^*G12D*^ allelic imbalance in the lung was linked to carcinomas in *Kras/Trp53* compound mutant mice^22^. As *Trp53* barrier loss can facilitate *Kras*^*G12D*^ allelic imbalance^18^, we studied macrodissected lung adenomas and carcinomas from *Trp53*-wild-type (WT) *Ela-creER*^*TM*^*;Kras*^*LSL-G12D/+*^ mice (Methods and Extended Data Fig. 7e,f). We found that *Kras*^*G12D-iGD*^ is frequently acquired in carcinomas, but not adenomas (Fig. 3e), confirming the later acquisition of *Kras*^*G12D-iGD*^ in the lung.

To study the timing of *Kras*^*MUT-iGD*^ in RAS-initiated (serrated) intestinal tumorigenesis, we analysed *Kras*^*G12D*^ allelic status in 63 MCCA organoid lines isolated at distinct disease stages from *Vil-cre;Kras*^*LSL-G12D/+*^ mice. We found that *Kras*^*G12D-iGD*^ is rare in hyperplasias and adenomas but frequent in carcinomas and metastases (Fig. 3f and Extended Data Fig. 7g). These data suggest positive selection of *Kras*^*G12D-iGD*^ during intestinal adenoma to carcinoma progression, particularly given the lack of *Kras*^*WT*^ gain (Fig. 3f) or of increased genomic instability in *Kras*^*G12D-iGD*^ carcinomas (Extended Data Fig. 7h).

To functionally interrogate the contribution of *Kras*^*MUT-iGD*^ to intestinal cancer progression, we transplanted *Kras*^*G12D-HET*^ adenoma organoids of MCCA with or without subclonal *Kras*^*G12D-iGD*^ into mice. All engrafted adenomas progressed to carcinomas with frequent acquisition or clonal expansion of *Kras*^*G12D-iGD*^ (Extended Data Fig. 7i). Notably, individual MCCA adenoma lines displayed markedly different engraftment rates that strongly correlated with the extent of subclonal *Kras*^*G12D-iGD*^ at transplantation, but not with SNV or CNV load (Extended Data Fig. 7j–n). Thus, *Kras*^*MUT*^ allelic imbalance is functionally relevant and selected for during adenoma-to-carcinoma progression. Overall, these results highlight tissue-specific differences in the evolutionary timing of *Kras*^*MUT-iGD*^ acquisition.

## *KRAS* effects are dosage and tissue dependent

**Extended Data Fig. 8:**
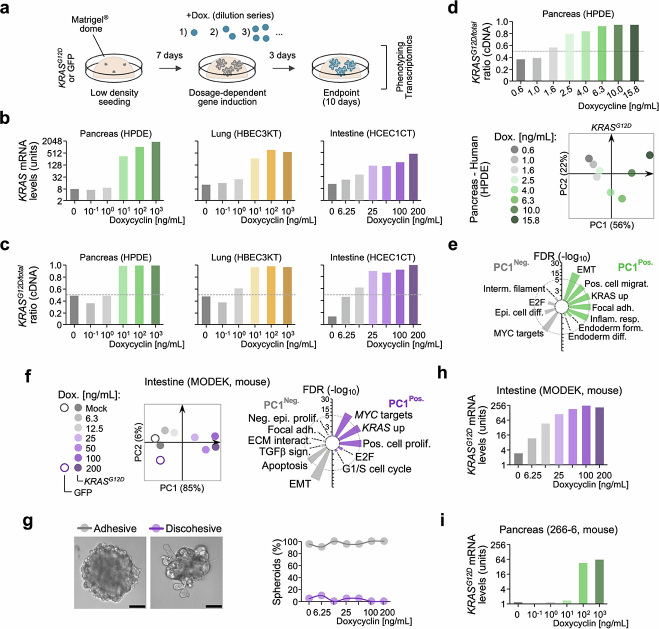
Titratable induction of *KRAS*^*MUT*^ expression for modelling the dosage-dependency of its molecular and cellular effects. **a**, Experimental outline for doxycycline-titratable expression of *KRAS*^*G12D*^ in pancreatic, lung and intestinal cell lines. To model early cancer evolution, non-transformed/immortal cells were used instead of full-blown cancer cell lines. Further, cells were cultured in optimized 3D conditions to model processes related to initiation of carcinogenesis, such as epithelial de-differentiation or invasion. To this end, cells were first seeded at very low density in Matrigel domes and allowed to form clonal spheroids. After 7 days of initial growth, distinct levels of *KRAS*^*G12D*^ expression were induced by using defined doxycycline concentrations. *KRAS*^*G12D*^ expression was continued for the following 3 days before spheroid phenotypes (at least 20 spheroids imaged per condition), and mRNA transcriptomes were assessed. GFP was used as control for doxycycline-mediated effects. **b**, Total *KRAS* mRNA levels induced by increasing concentrations of doxycycline in non-transformed human cell lines. *KRAS* mRNA levels were normalized to *GAPDH*. Both targets were quantified by TaqMan qPCR. **c**, Relative ratio of *KRAS*^*G12D*^ to total *KRAS* mRNA transcripts induced by increasing doxycycline concentrations in non-transformed human cell lines, and as detected by amplicon-based cDNA sequencing. The dotted horizontal line at 0.5 ratio indicates the threshold at which *KRAS*^*G12D*^ levels start to increase over *KRAS*^*WT*^ – a point which might resemble the transition from heterozygous to increased *KRAS*^*G12D*^ dosage. **d,e**, Modelling low-level *KRAS*^*G12D*^ dosage increase in non-transformed, human pancreatic ductal epithelial (HPDE) cells using a fine-graded titration series of doxycycline concentrations between 0.6 and 15.8 ng/mL. **d**, *KRAS*^*G12D*^ to total *KRAS* transcript ratios steadily increase with doxycycline concentration as detected by amplicon-based deep cDNA sequencing (**top**). PCA of *KRAS*^*G12D*^-induced transcriptome changes at different doxycycline concentrations (**bottom**). Progressive transcriptomic changes along PC1 are driven by *KRAS*^*G12D*^ in a dosage-dependent manner. Of note, the tipping point at which strong transcriptomic shifts in the PCA start to emerge is at a *KRAS*^*G12D*^ to total *KRAS* transcript ratio of ~80%. **e**, Gene set enrichment analysis of the top250 genes driving transcriptome separation on PC1 (negative, PC1^Neg.^; positive, PC1^Pos.^). Circular bar plot shows selected gene sets enriched on each principal component, which mirror the transcriptomic changes observed in the wider-range titration experiment (Fig. 4b). A full list of enriched gene sets is provided in Supplementary Table 10. FDR, false discovery rate. **f-h**, Doxycycline-titratable induction of *KRAS*^*G12D*^ expression in a non-transformed, murine intestinal cell line from MCCA. GFP served as control for doxycycline-mediated effects. **f**, PCA of *KRAS*^*G12D*^-induced transcriptome changes at defined doxycycline concentrations (**left**). Circular bar plot (**right**) depicts gene set enrichment analyses of top250 genes driving transcriptome separation on PC1 (negative, PC1^Neg.^; positive, PC1^Pos.^). Selected gene sets are shown (a full list of enriched gene sets is provided in Supplementary Table 11). FDR, false discovery rate. **g**, *KRAS*^*G12D*^ dosage-dependent induction of cellular phenotypes as detected by microscopy (n = 21 spheroids per doxycycline concentration). Graphs depict the frequency of adhesive/discohesive phenotypes at each doxycycline concentration. Representative images exemplify each phenotype for this cell line. **h**, *KRAS*^*G12D*^-specific mRNA expression levels normalized to *Gapdh*, both determined by TaqMan qPCR. **i**, Doxycycline-titratable induction of *KRAS*^*G12D*^ mRNA expression in murine pancreatic acinar carcinoma cells from MCCA. *KRAS*^*G12D*^-specific mRNA levels were normalized to *Gapdh*. Both targets were quantified by TaqMan qPCR.

To identify the biological basis of these observations, we next examined the molecular and cellular effects exerted by different *KRAS*^*MUT*^ dosages across tissues. We modelled early tumour evolution by developing non-transformed human cell lines with doxycycline-titratable *KRAS*^*G12D*^ expression. Growing them under three-dimensional (3D) (instead of two-dimensional (2D)) conditions enabled accurate examination of related processes like de-differentiation or invasion (Extended Data Fig. 8a). We optimized doxycycline concentration ranges to achieve *KRAS*^*G12D*^ mRNA induction over a wide dynamic range for each cell model (Extended Data Fig. 8b) and performed RNA-sequencing (RNA-seq) to capture transcriptome changes at each condition.

**Fig. 4:**
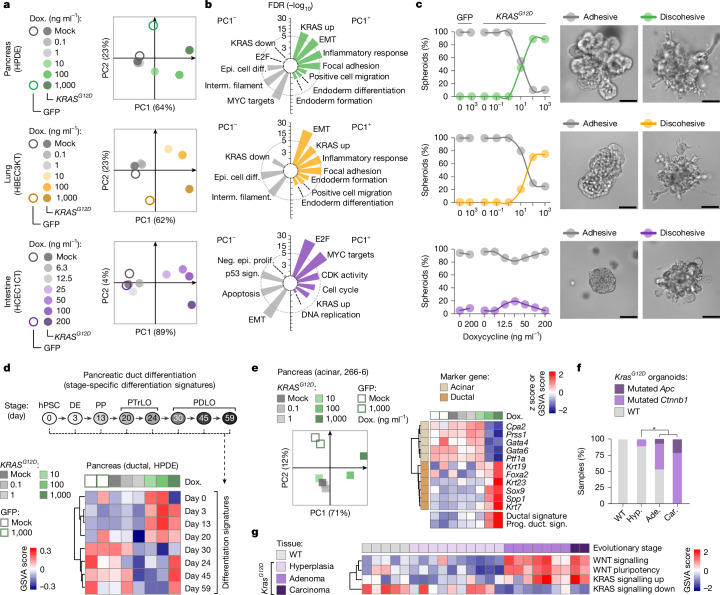
*KRAS*^*MUT*^ effects are dosage dependent and shaped by cellular context. **a**–**c**, Doxycycline (dox)-titratable induction of *KRAS*^*G12D*^ or GFP in the indicated non-transformed human cellular models (Methods and Extended Data Fig. 8a). **a**, PCA of *KRAS*^*G12D*^-induced transcriptome changes and GFP controls. **b**, Gene set enrichment analyses of the top 250 genes driving transcriptome separation on PC1 (negative, PC1^−^; positive, PC1^+^). The circular bar plots show selected gene sets. FDR, false-discovery rate. Epi. cell diff., epithelial cell differentiation; interm. filament, intermediate filament. **c**, Microscopy-based analysis of *KRAS*^*G12D*^-induced cellular phenotypes and GFP controls. Left, the frequency of adhesive/discohesive phenotypes at each doxycycline concentration (*n* ≥ 20 spheroids per condition; Methods). Right, representative images exemplify each phenotype (*n*_total_ ≥ 160 spheroids imaged per model). Scale bars, 60 µm. **d**, Inference of developmental programs induced by *KRAS*^*G12D*^. Upregulation/downregulation of eight developmental signatures in HPDE cells after induction of different *KRAS*^*G12D*^ levels (data are from **a** and **b**). The heat map shows gene set variation scores. The eight signatures signify developmental stages during in vitro differentiation of human embryonic stem cells into ductal cells^26^. DE, definitive endoderm; hPSC, human pluripotent stem cell; PDLO, pancreatic duct-like organoid; PP, pancreatic progenitor; PTrLO, pancreatic-trunk-like organoid. **e**, Doxycycline-titratable induction of *KRAS*^*G12D*^ or GFP in a pancreatic acinar cell model (266-6). Left, PCA of *KRAS*^*G12D*^-induced transcriptome changes, and GFP controls. Right, clustered heat map showing expression changes for acinar (top cluster) or ductal transcription factors/marker genes (bottom cluster) as well as of ductal^55^ (duct.) and progenitor^56^ (prog.) duct signatures (sign.). Genes, *z*-transformed expression values; signatures, gene set variation scores. **f**, Somatic WNT pathway mutations in organoids isolated from defined disease stages of *Vil-cre;Kras*^*LSL-G12D/+*^ mice or WT controls. *n* = 4 (WT), *n* = 9 (hyperplasia), *n* = 27 (adenoma), *n* = 5 (carcinoma). *P = 0.0164, two-sided *χ*^2^ test. **g**, The transcriptomic gene set variation scores of the indicated signatures in MCCA tissues isolated from defined diseases stages of *Vil-cre;Kras*^*LSL-G12D/+*^ mice or WT controls. Source data

Principal component analyses (PCA) revealed that *KRAS*^*G12D*^ is driving transcriptomic changes in a dosage-dependent manner (mainly along PC1; Fig. 4a). These effects started emerging at doxycycline concentrations at which *KRAS*^*G12D*^ exceeds *KRAS*^*WT*^ expression (Extended Data Fig. 8c,d). To examine the nature of *KRAS*^*G12D*^-driven transcriptional responses in PC1, we conducted gene set enrichment analyses (Methods). While all of the models displayed dosage-dependent *KRAS*^*G12D*^ effects, the induced molecular processes differed between entities (Fig. 4b, Extended Data Fig. 8e and Supplementary Table 10). Characteristic for pancreas and lung were signatures related to invasion (EMT, focal adhesion) and reactivation of fetal programs (endoderm differentiation/formation). The intestine instead displayed prominent dosage-dependent proliferation signatures, as also confirmed in a mouse model (Extended Data Fig. 8f–h and Supplementary Table 11).

To examine whether *KRAS*^*G12D*^ dosage-dependent signatures relate to specific cellular phenotypes, we imaged spheroids at each doxycycline dilution and classified them as either adhesive or discohesive, based on protrusion formation, cellular invasion and loss of epithelial organization (Methods). Discohesive growth appeared in the pancreas and lung, but not in the intestine (Fig. 4c and Extended Data Fig. 8g)—mirroring the tissue specificity of invasion-related transcriptional signatures (Fig. 4b). Thus, the biological effects of *KRAS* are not only dosage dependent but also profoundly shaped by tissue context: invasion and developmental programs in pancreas/lung versus proliferation in the intestine.

## Pancreatic de-differentiation is *KRAS* dosage-sensitive

*KRAS*^*MUT*^ allelic imbalance is detectable in early pancreatic cancer precursors. These lesions arise through cellular de-differentiation, which might therefore be *KRAS*-dosage sensitive. To test this possibility, we overlayed the transcriptomic effects exerted by different *KRAS*^*G12D*^ levels in human pancreatic ductal epithelial (HPDE) cells with cell-type-specific signatures representing the consecutive stages of pancreatic duct cell differentiation (Fig. 4d). For the latter, we used stage-specific transcriptomic signatures from an in vitro human stem-to-ductal cell differentiation series^26^ and performed gene set variation analysis (GSVA) to determine the upregulation/downregulation of each developmental signature in the transcriptomes of HPDE cells. We found that high—but not low—levels of *KRAS*^*G12D*^ induce a switch from differentiated duct-like (days 30, 45, 59) to early pancreatic progenitor-like (days 3, 13, 20) signatures in HPDE cells (Fig. 4d).

As PACA can originate not only from ductal cells but also from acinar cells, we studied a pancreatic acinar cell model after equipping it with doxycycline-inducible *KRAS*^*G12D*^ (Extended Data Fig. 8i). We found that *KRAS*^*G12D*^ induces de-differentiation of acinar cells in a dosage-dependent manner (Fig. 4e): while markers of acinar cell differentiation (such as *Cpa2*, *Prss1* and* Ptf1a*) were progressively lost, duct-like precursor cell markers (such as *Sox9*) increased with doxycycline concentrations.

**Extended Data Fig. 9:**
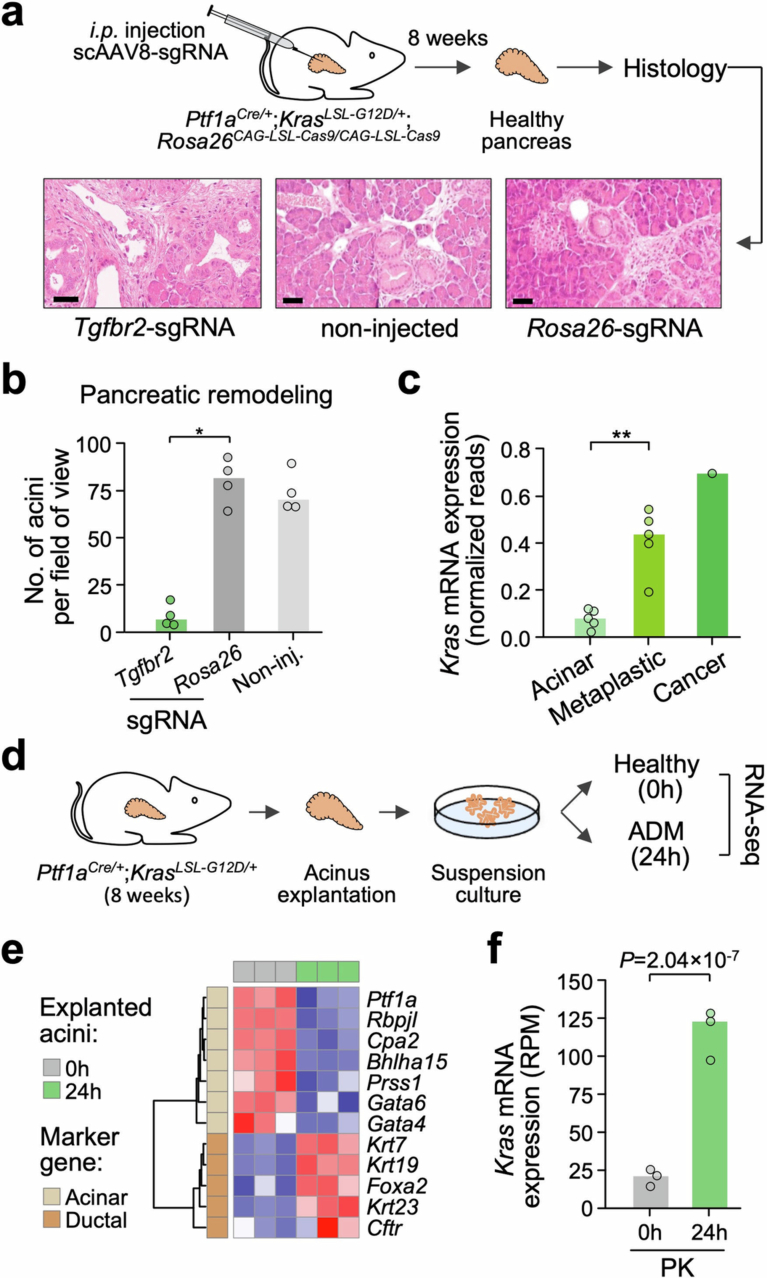
Drivers of early de-differentiation in the pancreas beyond *KRAS*^*MUT*^ gene dosage increase. **a,b**, Loss of *Tgfbr2* enhances *Kras*^*G12D*^-driven de-differentiation of acinar cells during early stages of pancreatic cancer evolution in vivo. **a, Top:** Overview of the experimental procedure. Single-stranded AAV8 (scAAV8) carrying *Tgfbr2* or *Rosa26* control sgRNA was injected into *Ptf1a*^*Cre/+*^*;Kras*^*LSL-G12D/+*^*,Rosa26*^*CAG-LSL-Cas9/CAG-LSL-Cas9*^ (PKR) mice for CRISPR/Cas9-mediated somatic gene inactivation in adult pancreatic acinar cells. Non-injected PKR mice served as control for the delivery of scAAV8. **Bottom:** Representative hematoxylin and eosin-stained pancreas sections of PKR mice eight weeks post-sgRNA delivery or of age-matched non-injected controls. Scale bar: 50 µm. **b**, Quantification of acini per field of view in the pancreas of *Tgfbr2*-sgRNA-, *sgRosa26*-sgRNA- and non-injected PKR mice. Dots represent independent mice. For each mouse, the mean acinus count from at least five hematoxylin and eosin-stained images is shown. Loss of *Tgfbr2* dramatically enhances *Kras*^*G12D*^-driven acinar cell de-differentiation, consistent with essential role of TGFβ in maintaining acinar cell identity. The results are also in line with the reduced selective pressure for the acquisition of *Kras*^*G12D*^ allelic imbalance during early pancreatic tumorigenesis in *Ptf1a*^*Cre/+*^;*Kras*^*LSL-G12D/+*^;*Tgfbr2*^∆^ mice^18^. *P = 0.0286, two-sided Mann–Whitney test; bars, median. **c**, *Kras* mRNA expression from its endogenous locus during *Kras*^*G12D*^-driven pancreatic cancer evolution in vivo. Induction of *Kras* mRNA levels was measured in acinar, metaplastic/PanIN and cancer cells from *Ptf1a*^*Cre-ERTM/+*^;*Kras*^*LSL-G12D/+*^*,Rosa26*^*LSL-CAG-tdTomato/+*^ mice as compared to healthy, *Kras* wildtype acinar cells (data from^29^. Endogenous *Kras* expression increases 5.5-fold during acinar cell de-differentiation (and is further enhanced in the cancer cell state). Normalized reads, Mean of normalized reads per cell. *P* = 0.0079, two-sided Mann–Whitney test; bars, median. **d-f**, *Kras* mRNA expression from its endogenous locus during de-differentiation of *Kras*^*G12D*^-mutant acinar cells in vitro. **d**, Overview on the experimental procedure of the ex vivo acinar-to-ductal metaplasia (ADM) assay. Healthy acini were explanted from 8 weeks-old *Ptf1a*^*Cre/+*^;*Kras*^*LSL-G12D/+*^ mice and cultured under ultra-low attachment conditions in vitro, where they spontaneously transdifferentiate into duct-like precursor cells within 24 h. **e**, Clustered heatmap illustrates the downregulation of acinar and upregulation of ductal marker gene expression in transdifferentiated (24 h) versus freshly isolated acini (0 h), as determined by RNA-seq. **f**, Bar plot showing *Kras* mRNA expression from its endogenous locus in de-differentiated metaplastic (24 h) versus freshly isolated acini (0 h), as quantified by RNA-seq. A 5.7-fold upregulation of *Kras* expression from its endogenous locus is observed during acinar cell de-differentiation, as observed during de novo tumorigenesis in mice (see (**c**) above). *P* = 2.04 × 10^−7^, two-sided Wald test in DESeq2; bars, median.

The dependence of transcriptional reprogramming (the first step in PACA initiation) on increased *KRAS*^*MUT*^ levels rationalizes the selective pressure to amplify oncogenic signalling early in oncogenesis. We therefore hypothesized that other triggers causing de-differentiation would release the selective pressure to amplify *Kras*^*MUT*^ during early evolution. Indeed, we previously found that cancers in *Ptf1a*^*cre/+*^*;Kras*^*LSL-G12D/+*^*;Tgfbr2*^*fl/fl*^ mice are largely *Kras*^*G12D-HET*^ (ref. ^18^), which might reflect the role of TGFβ in maintaining the acinar cell identity^27^. To test this, we somatically inactivated *Tgfbr2* in fully differentiated acinar cells of adult *Ptf1a*^*cre/+*^*;Kras*^*LSL-G12D/+*^*,Rosa26*^*CAG-LSL-Cas9/CAG-LSL-Cas9*^ mice using scAAV8-based sgRNA delivery, as described previously^28^. We found that *Tgfbr2* targeting substantially increased acinar de-differentiation 8 weeks after mutagenesis compared with control mice (Extended Data Fig. 9a,b). *Tgfbr2* inactivation therefore promotes loss of acinar cell identity, thereby reducing the selective pressure for early acquisition of *Kras*^*MUT-iGD*^.

Together, these results highlight the importance of *Kras*^*MUT-iGD*^ for early pancreatic de-differentiation. As genetic duplication of *KRAS*^*MUT*^ seems sufficient to trigger reprogramming (high-copy amplification is uncommon), we examined whether non-genetic mechanisms further enhance transcriptional output at the locus during de-differentiation. To this end, we first used single-cell RNA-seq (scRNA-seq) data from pancreata of *Ptf1a*^*cre-ERTM/+*^*;Kras*^*LSL-G12D/+*^*,Rosa26*^*LSL-CAG-tdTomato/+*^ mice^29^. In this model, tdTomato marks acinar cells (*Cpa1*^+^tdTomato^+^*Krt19*^−^) and their de-differentiated duct-like progeny (*Cpa1*^−^tdTomato^+^*Krt19*^+^, acinar-to-ductal metaplasia (ADM)/PanIN cells), while adult ductal cells are *Cpa1*^−^tdTomato^−^*Krt19*^+^. Comparative analyses revealed that endogenous *Kras* expression increases 5.5-fold during acinar cell de-differentiation and is further enhanced in the cancer cell state (Extended Data Fig. 9c). To verify this effect, we performed an ex vivo ADM assay, in which healthy acini from *Ptf1a*^*cre/+*^*;Kras*^*LSL-G12D/+*^ mice spontaneously transdifferentiate into duct-like precursor cells. As in mice, we found 5.7-fold upregulation of endogenous *Kras* expression in de-differentiated metaplastic cells (Extended Data Fig. 9d–f). Thus, the effect of (low-level) *Kras*^*MUT-iGD*^ is further amplified through non-genetic mechanisms during successive cell state transitions in PACA evolution.

De-differentiation also contributes to lung oncogenesis. It has been associated with progressive amplification of MAPK signalling^30,31^, triggered by cell-extrinsic or cell-intrinsic (genetic) events. Given the low prevalence of *Kras*^*G12D-iGD*^ in early-stage lung adenomas (Fig. 3e), cell-extrinsic triggers might have a more important role for early de-differentiation in the lung.

## *KRAS*–WNT collaboration in the intestine

In intestinal tumour evolution, monoallelic *Kras*^*G12D*^ activation in tissue-resident stem cells induces hyperplasia, while *Kras*^*G12D-iGD*^ emerges at the carcinoma stage (Fig. 3f). This raises the question of what triggers adenoma formation. Given the rapid turnover of intestinal epithelia, which leaves little time to acquire mutations, we reasoned that a block of differentiation might be needed at this evolutionary step.

As this process seems to be independent of *Kras*^*G12D*^ in the intestine (Fig. 4a–c and Extended Data Fig. 8f–h), we screened MCCA organoids from *Kras*^*G12D*^-induced hyperplasias, adenomas and carcinomas for mutations in the WNT pathway—the key regulator of intestinal differentiation. We indeed found that *Apc* and *Ctnnb1* mutations specifically emerged at the adenoma stage, affecting 48% of cases (Fig. 4f and Supplementary Table 12). Moreover, transcriptome analyses of MCCA tissues highlighted WNT pathway upregulation in all adenomas, independent of the *Apc* or *Ctnnb1* mutation status (Fig. 4g).

In the classical model of intestinal tumorigenesis (initiated by *APC* mutation), WNT pathway activation is the gatekeeper event for adenoma formation^32^. Our results suggest that the same applies to *KRAS*-initiated serrated tumorigenesis. As *KRAS*^*MUT*^ cannot block intestinal differentiation, stochastic *KRAS*^*MUT-iGD*^ events are rapidly lost in shedding cells of hyperplastic tissue, explaining why *KRAS*^*MUT-iGD*^ is not observed in early oncogenesis. It is only after WNT-induced block of differentiation at the adenoma stage that the competitive growth advantage exerted by *KRAS*^*MUT-iGD*^ translates into clonal outgrowth.

## *KRAS*–TSG interactions are entity specific

**Fig. 5:**
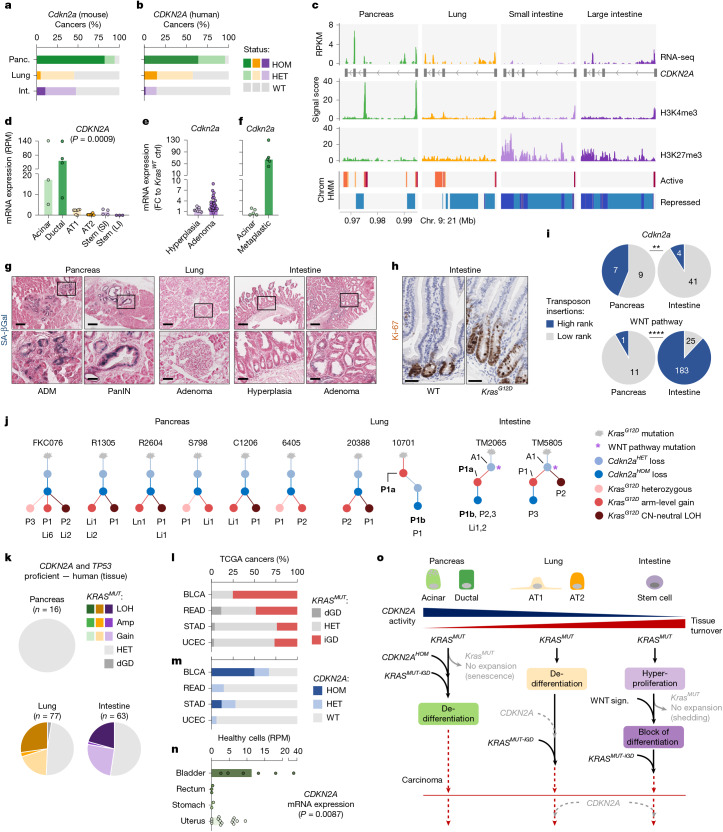
*KRAS*–tumour suppressor interactions are cell-type specific. **a**,**b**, Somatic *CDKN2A* inactivation patterns in the indicated cancer cohorts based on genomics data (Methods). Microdissected MCCA cancers (**a**): from *Ptf1a*^*cre/+*^*;Kras*^*LSL-G12D*/+^ (*n* = 17), *Ela-creER*^*TM*^*;Kras*^*LSL-G12D/+*^ (*n* = 44) and *Vil-cre;Kras*^*LSL-G12D/+*^ (*n* = 19) mice. Human cancer tissues (**b**): ICGC-PanCuRx (*n* = 114), TCGA-LUAD (*n* = 135) and TCGA-COAD (*n* = 136). **c**, *CDKN2A* chromatin status and mRNA expression in healthy human pancreas, lung and intestine inferred from ROADMAP data. Top, transcript coverage plots (reads per kilobase million (RPKM)). Middle, histone modification signal plots. H3K4me3, promoter activation; H3K27me3, Polycomb repression. Signal score, −log_10_[*P*]. Bottom, selected chromatin states of a core 15-state model based on five histone marks (Extended Data Fig. 10c). Coordinates based on the GRCh37 human reference genome. **d**, Cell-type-specific *CDKN2A* expression based on pseudobulk analyses of public scRNA-seq data (Methods). RPM, reads per million. *P* = 0.0009, Kruskal–Wallis test. Data are median. **e**,**f**, The responsiveness of *Cdkn2a* to *Kras*^*G12D*^ during early tumour evolution. **e**, Hyperplasia and adenoma (*Vil-cre;Kras*^*LSL-G12D/+*^) versus healthy (*Kras*^*WT*^) intestinal organoids. **f**, Acinar and metaplastic/PanIN (*Ptf1a*^*cre-ERTM/+*^*;Kras*^*LSL-G12D/+*^*,Rosa26*^*LSL-CAG-tdTomato/+*^) versus healthy (*Kras*^*WT*^) acinar cells (data are from ref. ^29^). FC, fold change. **g**, Representative senescence-associated β-galactosidase (SA-βGal) in precursor lesions of pancreatic (*Ptf1a*^*cre/+*^*;Kras*^*LSL-G12D/+*^,* n* = 4 mice), lung (*Ela-creER*^*TM*^*;Kras*^*LSL-G12D/+*^, *n* = 4 mice) and serrated intestinal (*Vil-cre;Kras*^*LSL-G12D/+*^, *n* = 8 mice) cancer. Scale bars, 200 µm (top) and 50 µm (bottom). **h**, Representative duodenal Ki-67 staining of WT mice (*n* = 3) and hyperplastic crypts of *Vil-cre;Kras*^*LSL-G12D/+*^ mice (*n *= 3). Scale bars, 50 µm. **i**, The selective advantage conferred by *Cdkn2a* and WNT pathway alterations in the indicated tissues, as determined by transposon-based forward genetic screens in *Pdx1-cre;Kras*^*LSL-G12D/+*^*,Rosa26*^*LSL-piggyBac/+*^*;ATP1-S2* (*n* = 49)^43^ and *Vil-cre;Kras*^*LSL-G12D/+*^*,Rosa26*^*LSL-piggyBac/+*^*;ATP1-S2* (*n* = 219) mice (Methods). **P = 0.0018; two-sided *χ*^2^ test. **j**, The chronological order of *Kras*^*G12D*^ and *Cdkn2a* alterations during oncogenesis, as determined by phylogenetic analyses in matched samples from *Ptf1a*^*cre/+*^*;Kras*^*LSL-G12D/+*^, *Ela-creER*^*TM*^*;Kras*^*LSL-G12D/+*^ and *Vil-cre;Kras*^*LSL-G12D/+*^ mice (Extended Data Fig. 12a). A, adenoma; P, primary cancer; Li/LN, liver/lymph-node metastasis. Non-bold and bold labels indicate cell lines and tissues, respectively. **k**, *KRAS*^*MUT*^ allelic status of *CDKN2A*/*TP53*-proficient human pancreatic (ICGC-PanCuRx and TCGA-PAAD, *n*_total_ = 201), lung (TCGA-LUAD, *n*_total_ = 135) and intestinal (TCGA-COAD, *n*_total_ = 136) cancer tissues (Methods). **l**,**m**, *KRAS*^*MUT*^ allelic status (**l**) and *CDKN2A* inactivation patterns (**m**) in human bladder (TCGA-BLCA, *n* = 12), rectal (TCGA-READ, *n* = 35), stomach (TCGA-STAD, *n* = 25) and uterine (TCGA-UCEC, *n* = 67) carcinomas (Methods). **n**, *CDKN2A* mRNA expression for cell-of-origin candidates of the indicated cancer types based on pseudobulk analyses of public scRNA-seq data^47–49^ (Methods). *P* = 0.0087, Kruskal–Wallis test. The bars show the median value. **o**, Oncogene–tumour suppressor interactions and cellular processes in *KRAS*^*MUT*^-initiated pancreatic, lung and serrated intestinal cancer evolution. The simplified model displays interactions between *KRAS*^*MUT*^ and *CDKN2A*, but not other tumour suppressors such as *TP53*. See the Discussion for details.

Given the tissue-specific timing and output of *KRAS*^*MUT*^ dosage variation, we suspected that interactions of the oncogene with tumour suppressor genes (TSGs) might also be context-dependent. RAS signalling is known to engage endogenous tumour suppression, most notably through *CDKN2A* activation^33^. We therefore analysed *Cdkn2a* alteration patterns across pancreatic, lung and intestinal carcinomas. As *Cdkn2a* might be lost during in vitro cell culture^34^, we studied microdissected MCCA tissues. These analyses revealed substantial differences between cancer types, with homozygous *Cdkn2a* loss being frequent in *Kras*^*G12D*^-mutant pancreatic carcinomas (82%), but rare in lung (5%) or intestinal (11%) carcinomas (Fig. 5a and Supplementary Table 13). Likewise, examination of human *KRAS*^*MUT*^ cancers exposed frequent biallelic *CDKN2A* inactivation in human PACA (64%), but not in human LUCA (15%) and human COCA (2%) (Fig. 5b and Supplementary Table 14). Thus, although all cancers are *KRAS* mutant, selective pressure for *CDKN2A* inactivation differs substantially between tissues.

## Cell-type-specific repression of *CDKN2A*

**Extended Data Fig. 10:**
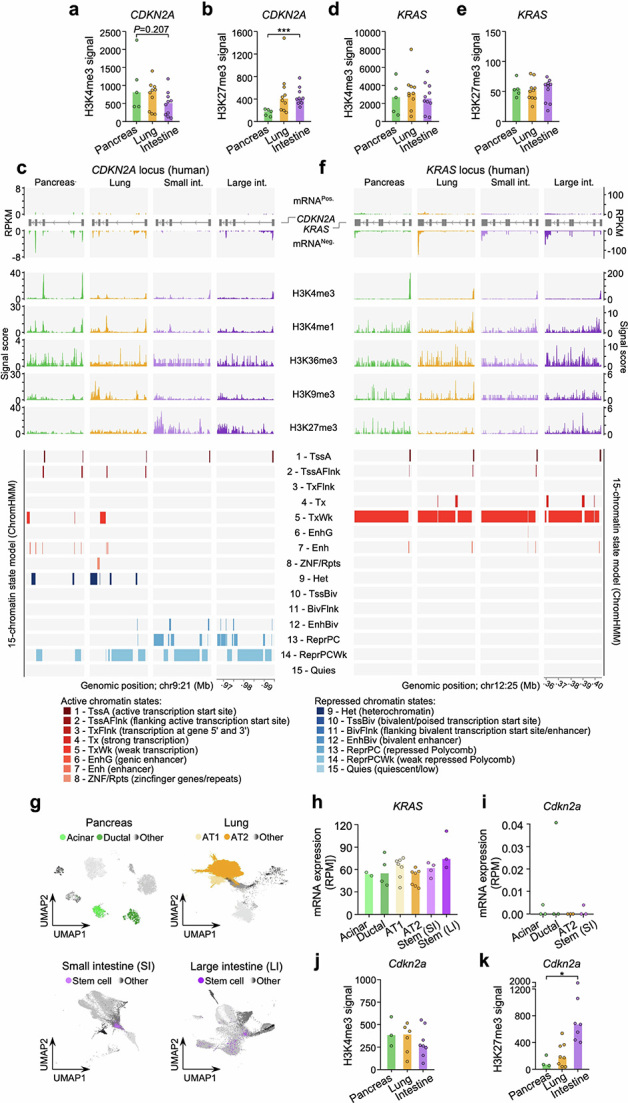
Transcriptional activity and epigenetic status of *CDKN2A* or *KRAS* across tissues and species. **a,b**, Quantitative comparisons of H3K4me3 (**a**) and H3K27me3 (**b**) ChIP-seq signals at the *CDKN2A* locus across human pancreatic, lung, and intestinal tissues using ROADMAP and ENCODE epigenomics datasets (Supplementary Table 15, and as described in Methods). H3K27me3 signal or H3K4me3 signal, normalized reads per kb. ***P = 0.0007, two-sided Mann-Whitney test; bars, median. **c**, mRNA expression, histone modification patterns and chromatin states at the *CDKN2A* locus in healthy human pancreas, lung and small/large intestine as inferred from ROADMAP reference transcriptomes and epigenomes^35^ (related to Fig. 5c). **Top:** mRNA expression of the *CDKN2A* locus (as detected by full-length mRNA sequencing). Coverage plots depicting the mapping of mRNA transcripts either to the forward or the reverse strand of the genome are shown (*CKDN2A* is transcribed from the reverse DNA strand). Cartoons in-between forward and reverse strand coverage plots illustrate *CKDN2A* exon structure. RPKM, reads per kilobase million. **Middle:** Histone modifications at the *CDKN2A* locus. Signal plots for histone marks are shown: H3K4me3 (associated with promoter regions), H3K4me1 (associated with enhancer regions), H3K36me3 (associated with transcribed regions), H3K9me3 (associated with heterochromatin regions) and H3K27me3 (associated with Polycomb repression). Genome-wide signal plots were calculated using the Model-based Analysis of ChIP-seq 2 (MACS2) tool. After normalization, the enrichment/signal scores were provided as negative log_10_ of the Poisson p-values. Signal score, -log_10_(p-value). **Bottom:** Chromatin states found at the *CDKN2A* locus as inferred from the ChromHMM ‘core’ 15-state model. This model was previously trained by integrating 5 chromatin marks (H3K4me3, H3K4me1, H3K36me3, H3K9me3 and H3K27me3) across 127 reference epigenomes covering a broad spectrum of tissues and cell types^35^. Active chromatin states of the model are highlighted in shades of red (states 1–8) and repressed chromatin states in shades of blue (states 9–15). To visualize chromatin states at the *CDKN2A*, the locus was binned, and each genomic bin was then labelled with the chromatin state having the highest posterior probability at the given genomic position. Coordinates at the bottom indicate the genomic position for mRNA expression, histone modifications and chromatin states according to human genome assembly GRCh37. **d,e**, Quantitative comparisons of H3K4me3 (**d**) and H3K27me3 (**e**) ChIP-seq signals at the *KRAS* locus across human pancreatic, lung, and intestinal tissues using ROADMAP and ENCODE epigenomics datasets (Supplementary Table 15, and as described in Methods). Note the different scales of the y axes compared to (**a,b**). No significant differences of histone mark occupancy were detected between tissues (two-sided Mann-Whitney test). H3K27me3 or H3K4me3 signal, normalized reads per kb. Bars, median. **f**, mRNA expression, histone modification patterns and chromatin states at the *KRAS* locus in healthy human pancreas, lung and small/large intestine as inferred from ROADMAP reference transcriptomes and epigenomes^35^. Analyses as in (**c**). **g,h**, Cell type-specific *KRAS* mRNA expression for the cell-of-origin of each cancer type determined by single-cell RNA-seq (human data from^38–40^. Analysis as for *CDKN2A* mRNA expression in Fig. 5d. **g**, UMAPs of pancreatic, lung and intestinal cell types. Only cell types with n ≥ 100 cells are shown for lung and intestine. AT1/AT2, alveolar cells type I/II; SI, small intestine; LI, large intestine. **h**, Bar plot showing cell type-specific *KRAS* mRNA expression based on pseudobulk analyses per donor (see Methods). Expr., expression; RPM, reads per million. Bars, median. **i**, scRNA-seq pseudobulk analysis of *Cdkn2a* mRNA expression across indicated mouse cell types (data from^91^). The number of reads mapping to *Cdkn2a* is extremely low in all cell types – and substantially reduced when compared to their human counterparts (Fig. 5d). This finding is supported by previous observations that expression of *Cdkn2a* is very low or absent in healthy adult tissues of the mouse^41^. AT2, alveolar cells type II; SI, small intestine; RPM, reads per million. **j**, Quantification of H3K4me3 signals at the *Cdkn2a* locus based on public ChIP-seq data from healthy mouse pancreas, lung, and intestinal tissues (Supplementary Table 15, and as described in Methods). Abundance of H3K4me3 at *Cdkn2a* is very low and does not differ between tissues, consistent with the extremely low expression of *Cdkn2a* in the mouse scRNA-seq pseudobulk analyses shown in (**i**). H3K4me3 signal, normalized reads per kb. *P*, two-sided Mann-Whitney test; bars, median. **k**, Quantification of H3K27me3 signals at the *Cdkn2a* locus based on public ChIP-seq data from healthy mouse pancreas, lung, and intestinal tissues (Supplementary Table 15). H3K27me3 signal, normalized reads per kb. Compare to human in (**b**). *P = 0.0167, two-sided Mann-Whitney test; bars, median.

To study the mechanistic basis of this observation, we used ROADMAP and ENCODE epigenomics data^35,36^ to assess the chromatin states at *CDKN2A*, the regulation of which occurs chiefly at the transcriptional level^37^ (Supplementary Table 15). In the pancreas, *CDKN2A* expression was readily detectable, in accordance with trimethylation of histone H3 at lysine 4 (H3K4me3) occupancy at transcription start sites marking the actively transcribed promoter. Conversely, in the intestine, we found very low *CDKN2A* expression and high occupancy of H3K27me3, a repressive mark catalysed by Polycomb repressive complexes (PRCs) (Fig. 5c and Extended Data Fig. 10a,b).

We next integrated H3K4me1, H3K36me3 and H3K9me3 histone marks to investigate 15 distinct chromatin states, as provided through ROADMAP^35^. Indeed, active chromatin states characterized the *CDKN2A* locus in the pancreas, whereas in the intestine Polycomb-repressed states dominated (Fig. 5c and Extended Data Fig. 10c). In the lung, *CDKN2A* showed a bivalent pattern of active and repressive states associated with low expression. Notably, we found no tissue-specific differences for H3K4me3, H3K27me3 or chromatin states at *KRAS* (Extended Data Fig. 10d–f).

To examine *CDKN2A* transcriptional activity specifically in the cell of origin of each cancer type, we analysed human scRNA-seq data^38–40^. Consistent with our epigenetic analyses, *CDKN2A* expression is detectable in pancreatic acinar and ductal cells, but minimal or absent in lung alveolar or intestinal stem cells (Fig. 5d and Extended Data Fig. 10g). By contrast, *KRAS* is expressed across cell types (Extended Data Fig. 10h).

We also examined scRNA-seq and chromatin immunoprecipitation followed by sequencing (ChIP–seq) data from healthy mouse tissues to assess whether *Cdkn2a* regulation is conserved across species (Supplementary Table 15). We found that *Cdkn2a* expression was generally low, consistent with low H3K4me3 promoter occupancy and previous reports of minimal expression in adult mouse tissues^41^. Notably, repressive H3K27me3 occupancy is lower in the pancreas than in the intestine, while the lung exhibited intermediate levels, probably reflecting the bivalent *Cdkn2a* chromatin state (Extended Data Fig. 10i–k). Thus, in humans and mice, Polycomb repression at *CDKN2A* is strong in the intestine, moderate in the lung and weak in the pancreas.

**Extended Data Fig. 11:**
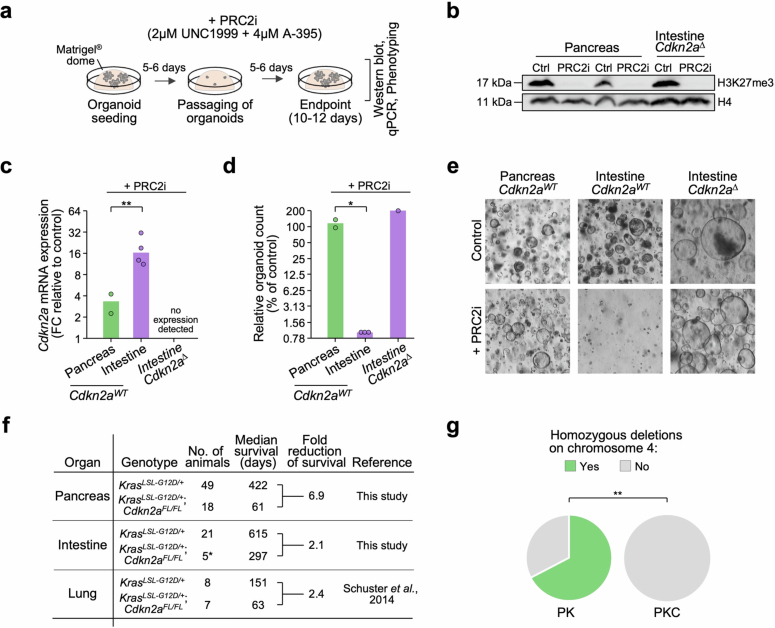
Polycomb-mediated repression of the *Cdkn2a* locus is cell type-specific and shapes tissue-specific *Cdkn2a* tumour suppressor function. **a**, Experimental outline for the inhibition of Polycomb Repressive Complex 2 (PRC2i) in non-transformed pancreatic and intestinal organoids through combinatorial treatment with UNC1999 (2 µM) and A-395 (4 µM). Following standard protocols, cells were treated with PRC2i for 10 to 12 days to not only block de novo H3K27 trimethylation but also to allow dilution of pre-existing H3K27me3 marks through cell division^99^. **b**, Western blot analysis of H3K27me3 in non-transformed pancreatic organoids and *Cdkn2a*-deficient intestinal organoids following Mock-treatment (Ctrl) or PRC2 inhibition (PRC2i). Histone H4 was used as loading control. **c**, Induction of *Cdkn2a* mRNA expression following PRC2 inhibition (PRC2i) in non-transformed pancreatic and intestinal organoids as determined by qPCR. *Cdkn2a* expression levels are normalized to *Gapdh* and presented as relative expression compared to the corresponding Mock-treated control. *Cdkn2a*^∆^, homozygous knockout of *Cdkn2a*. FC, fold change. *P = 0.0124; two-sided *t*-test on log_2_-transformed data; bars, mean. **d**, Organoid counts following PRC2 inhibition (PRC2i) in non-transformed pancreatic and intestinal organoids. Organoid counts of PRC2i-treated cells are shown as percentage relative to organoid counts of the corresponding Mock-treated control. Of note, the marked growth inhibitory effect of PRC2i in intestinal cells is completely rescued by knock-out of *Cdkn2a*, demonstrating that PRC2i-induced block of proliferation is *Cdkn2a*-dependent in this cell type. *Cdkn2a*^*WT*^, wildtype *Cdkn2a*; *Cdkn2a*^∆^, homozygous knockout of *Cdkn2a*. **P = 0.0047; two-sided *t*-test on log_2_-transformed data; bars, mean. **e**, Representative images of pancreatic and intestinal organoids following Mock-treatment (Ctrl) or PRC2 inhibition (PRC2i), corresponding to the quantification of organoid counts shown in (**d**). **f**, Survival analysis of *Kras*^*LSL-G12D/+*^ mice with or without *Cdkn2a* inactivation in the pancreas, intestine and lung, for determining the effect of *Cdkn2a* loss on oncogenesis in different tissues. Homozygous loss of *Cdkn2a* dramatically accelerates *Kras*^*G12D*^-initiated cancer evolution in the pancreas, while having only moderate effects in mouse models of *Kras*^*G12D*^-initiated intestinal or lung cancer. The survival comparison of *Kras*^*LSL-G12D/+*^ versus *Kras*^*LSL-G12D/+*^;*Cdkn2a*^*FL/FL*^ lung cancer mouse models is based on data from^46^. The number of animals included in each group is indicated in the table. *Only two of the five Vil-Cre;*Kras*^*LSL-G12D/+*^;*Cdkn2a*^*FL/FL*^ mice developed intestinal tumours. The remaining three animals did not show signs of intestinal neoplasia even after extended observation periods and died from tumours unrelated to intestinal cancer, most likely due to leakiness of Cre expression. **g**, Frequency of homozygous somatic deletions affecting chromosome 4, which harbours the *Cdkn2a* locus, in pancreatic cancer cell lines derived from *Ptf1a*^*Cre/+*^;*Kras*^*LSL-G12D/+*^ (PK, n = 49) or *Ptf1a*^*Cre/+*^;*Kras*^*LSL-G12D*/+^;*Cdkn2a*^*FL/FL*^ (PKC, n = 18) mice. While such deletions are frequent in PK cancers, their absence in the PKC model indicate that *Cdkn2a*, rather than neighbouring genes, drive this phenotype. However, the co-deletion of genes with *Cdkn2a* can be relevant for other phenotypes, such as for promoting immune evasion and cancer metastasis^45^. **P = 0.0055, two-sided Chi-squared test. Source data

We next tested whether PRC2-catalysed H3K27me3 is mechanistically involved in cell-type-specific repression of *Cdkn2a*. To this end, we pharmacologically inhibited PRC2 (PRC2i) in non-transformed mouse intestinal and pancreatic ductal organoids, followed by quantification of *Cdkn2a* expression and cellular growth (Extended Data Fig. 11a,b). We found that PRC2i induced *Cdkn2a* expression and cellular growth arrest in intestinal organoids, but not in pancreatic cells. Moreover, knocking out *Cdkn2a* rescued the PRC2i-induced inhibition of intestinal cell proliferation (Extended Data Fig. 11c–e). Thus, tissue-specific chromatin states of *Cdkn2a* directly affect its function.

Together, these analyses demonstrate Polycomb-mediated chromatin repression at *CDKN2A* in a tissue-specific manner, directly affecting tumour suppressor expression and function.

## *CDKN2A* chromatin states and response to *KRAS*

Given these tissue-specific differences in PRC2-mediated repression at *Cdkn2a*, we next investigated possible functional consequences for in vivo *Kras*^*MUT*^-driven tumour evolution.

First, we examined whether *Cdkn2a* responsiveness to *Kras*^*G12D*^ differs between intestinal and pancreatic cells during early tumour evolution. Analysing *Vil-cre;Kras*^*LSL-G12D/+*^ MCCA-mouse cohorts, we found that *Cdkn2a* expression only slightly increases as healthy intestinal cells progress to the hyperplasia and adenoma stage (Fig. 5e). By contrast, *Cdkn2a* is strongly induced during *Kras*^*G12D*^-initiated early pancreatic oncogenesis, when healthy acinar cells develop into metaplastic PanINs^29^ (Fig. 5f; data from *Ptf1a*^*cre-ERTM/+*^*;Kras*^*LSL-G12D/+*^*,Rosa*^*26LSL-CAG-tdTomato/+*^ mice).

Second, we investigated *Kras*^*G12D*^-induced cellular senescence across tissues—a key tumour-suppressive mechanism that is mediated by *Cdkn2a*. To this end, we performed senescence-associated β-galactosidase staining of non-transformed MCCA tissues from *Kras*^*G12D*^-mutant pancreatic, intestinal and lung cancer models. We found that senescence was prominent in early pancreatic lesions (ADM, PanIN), but minimal or absent in lung and intestinal precursors (Fig. 5g). Instead, *Kras*^*G12D*^ causes pronounced hyperproliferation in the intestine (Fig. 5h). Thus, tissue-specific *CDKN2A* responsiveness results in distinct functional outputs, with robust tumour-suppressive senescence being mounted only in the pancreas. This suggests that the selective pressure to lose *CDKN2A* is highest in the pancreas, as are its pro-tumorigenic effects.

Third, to quantify selection and clonal outgrowth conferred by *Cdkn2a* inactivation in different organs, we pursued a forward genetic screening approach in mice. We performed insertional mutagenesis using the insect-derived piggyBac transposon system, which we adapted earlier for applications in mice^42,43^. In cancers derived from corresponding mouse colonies (*Pdx1-cre;Kras*^*LSL-G12D/+*^*,Rosa26*^*LSL-piggyBac/+*^*;ATP1-S2* and *Vil-cre;Kras*^*LSL-G12D/+*^*,Rosa26*^*LSL-piggyBac/+*^*;ATP1-S2*), we sequenced and mapped transposon insertions as described previously^44^. To infer the selective advantage conferred by *Cdkn2a* inactivation, we ranked *Cdkn2a* insertions by read coverage relative to all other insertions in each sample (Methods). These analyses revealed that 44% of *Cdkn2a* insertions were top-10 ranked in pancreatic cancers, but only 9% in the intestine (Fig. 5i). Thus, *Cdkn2a* inactivation confers a significantly stronger selective advantage in the pancreas compared with in the intestine. Conversely, transposon insertions in WNT pathway genes were typically top-10 ranked in the intestine, but not in the pancreas (Fig. 5i)—confirming the dependence of *Kras*^*G12D*^-initiated intestinal cancer evolution on WNT pathway activation (Fig. 4f,g).

Fourth, further evidence linking tissue-specific *Cdkn2a* activity and natural selection for its loss comes from our prospective studies in mouse models of *Kras*^*G12D*^-initiated pancreatic, lung and intestinal cancer, where genomic *Cdkn2a* loss is near-ubiquitous in the pancreas but rare in the other organs (Fig. 5a).

Finally, by comparing *Kras*^*G12D*^-mutant mouse cohorts with or without *Cdkn2a* inactivation across tissues, we determined context-dependent effects of *Cdkn2a* loss on oncogenesis. We found that homozygous loss of *Cdkn2a* substantially accelerates *Kras*^*G12D*^-initiated pancreatic cancer evolution (*Ptf1a*^*cre/+*^*;Kras*^*LSL-G12D/+*^*;Cdkn2a*^*fl/fl*^ (PKC) versus *Ptf1a*^*cre/+*^*;Kras*^*LSL-G12D/+*^ (PK) mice; Extended Data Fig. 11f). Notably, deletions affecting chromosome 4 (containing *Cdkn2a*) are frequent in PK mice but absent in PKC mice (Extended Data Fig. 11g), excluding a major contribution of genes nearby *Cdkn2a* to this phenotype, which can be relevant in other contexts^45^. In contrast to the pancreas, *Cdkn2a* loss had a far less pronounced effect on tumour evolution in our intestinal cancer models (*Vil-cre;Kras*^*LSL-G12D/+*^*;Cdkn2a*^*fl/fl*^ versus *Vil-cre;Kras*^*LSL-G12D/+*^ mice; Extended Data Fig. 11f). Likewise, the *Cdkn2a* effects are relatively modest in *Kras*^*G12D*^-driven lung adenocarcinoma models (Extended Data Fig. 11f, data are from ref. ^46^).

Taken together, these functional studies in *KRAS*^*MUT*^-initiated cancers establish a chain of causality linking cell-type-specific *CDKN2A* chromatin states to differential *CDKN2A* expression and tumour suppression, ultimately defining the distinct selective pressures to lose this locus in different tissues.

## Order of gene alteration varies by tissue

**Extended Data Fig. 12:**
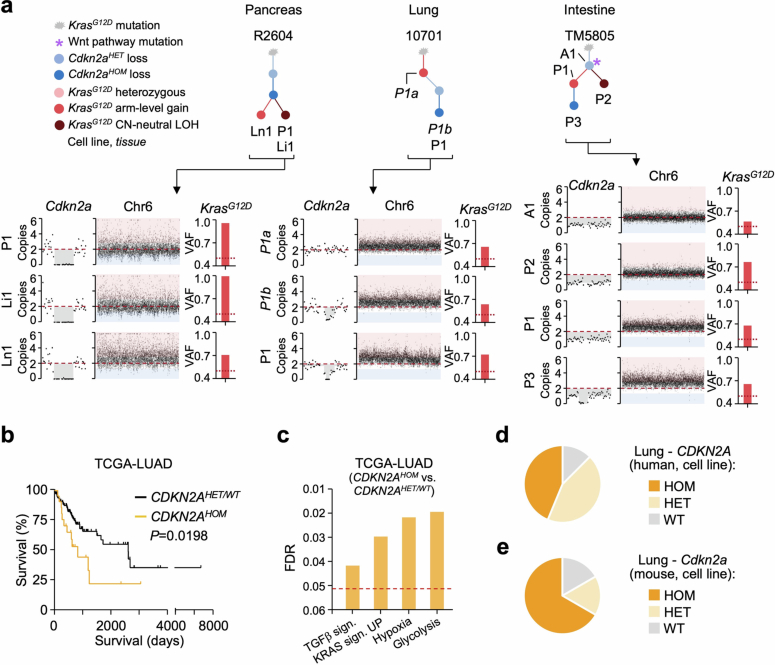
Chronological order of *Kras*^*MUT*^ and *Cdkn2a* alterations in different tissues, and late evolutionary advantage of *CDKN2A*^*HOM*^ in lung cancer. **a**, Chronological order of *Kras*^*G12D*^ gene dosage increase and bi-allelic *Cdkn2a* inactivation during evolution of mouse pancreatic (*Ptf1a*^*Cre/+*^;*Kras*^*LSL-G12D*/+^), lung (Ela-CreER^TM^;*Kras*^*LSL-G12D*/+^) and intestinal cancer (Vil-Cre;*Kras*^*LSL-G12D*/+^). Selected cases from Fig. 5j are shown to illustrate how the sequential order of genetic events was inferred using CNV profiling and *Kras*^*G12D*^ VAF data. **Top:** Phylogenetic trees indicating sequential order of genetic events. Length of lines does not represent evolutionary distances. Stromal contamination of MCCA cancer tissues was accounted for by the data analysis in order to study ‘pure’ cancer genomes (see Methods). **Bottom:** Plots showing *Cdkn2a* or chromosome 6 (home of *Kras*) CNV patterns as determined by WES or lcWGS, and *Kras*^*G12D*^ VAF as detected by amplicon-based deep sequencing. In the pancreas (case R2604), all corresponding cancer lesions share the identical pattern of homozygous *Cdkn2a* deletion, while distinct types of *Kras*^*G12D*^ gene dosage increase are detected in each individual sample. Accordingly, *Cdkn2a*^*HOM*^ preceded the acquisition of *Kras*^*G12D-iGD*^ during the evolution of pancreatic cancer in this mouse. By contrast, in the intestine (case TM5805), the type of *Kras*^*G12D*^ gene dosage increase is shared between corresponding cancer lesions, but patterns of homozygous *Cdkn2a* deletion are heterogenous. Thus, *Cdkn2a*^*HOM*^ occurred after *Kras*^*G12D-iGD*^ was already acquired during serrated intestinal cancer evolution in this mouse. A similar order of genetic events was detected in lung cancer case 10701. A, adenoma; P, primary cancer; Li/LN, liver/lymph-node metastasis. **b**, Overall patient survival related to lung adenocarcinomas (TCGA-LUAD) proficient (*CDKN2A*^*HET/WT*^) or deficient for *CDKN2A* (*CDKN2A*^*HOM*^). Only *KRAS*^*MUT*^ cancers were included in the analysis. *P*, two-sided log-rank test. **c**, Gene set enrichment analysis of genes upregulated in *CDKN2A*^*HOM*^ versus *CDKN2A*^*HET/WT*^ human lung adenocarcinoma tissues (TCGA-LUAD) as detected by RNA-seq. Bar plot shows selected gene sets (a full list of enriched gene sets is provided in Supplementary Table 16). Only *KRAS*^*MUT*^ cancers were included in the analysis. FDR, false discovery rate. **d**, Frequency of distinct somatic *CDKN2A* inactivation states in human, non-small cell lung cancer cell lines (CCLE-NSCLC) (Supplementary Table 17). Re-analysis of data provided by the Cancer Cell Line Encyclopedia^6^. HOM, homozygous; HET, heterozygous; WT, wildtype. **e**, Frequency of defined somatic *Cdkn2a* inactivation states in murine lung carcinoma (mLUCA) cell lines of MCCA (Supplementary Table 17). HOM, homozygous; HET, heterozygous; WT, wildtype.

We previously observed that *Kras*^*MUT*^ allelic imbalance in the pancreas is contingent on homozygous *Cdkn2a* loss^18^. Given the reduced ability of *Kras* to engage *Cdkn2a* in the lungs and intestine, we hypothesized that the sequence of genetic events during cancer evolution might differ between the three tissues. To study the temporal order of gene alterations, we performed phylogenetic studies analysing the allelic imbalance at *Cdkn2a* and *Kras* in matched primary cancers and metastases from our mouse models. Out of 81 lung and intestinal MCCA samples, 5 matched cases were *Cdkn2a*^*HOM*^*Kras*^*G12D-iGD*^ and were therefore amenable to such analyses. One pair displayed identical CNV and loss of heterozygosity (LOH) patterns at *Cdkn2a* and *Kras*, preventing the reconstruction of their sequentiality. In three out of the remaining four cases (one lung, two intestine), we found that *Cdkn2a*^*HOM*^ was acquired after *Kras*^*G12D-iGD*^ (Fig. 5j and Extended Data Fig. 12a), a scenario that we did not observe for the pancreas in a previous study^18^. To further confirm the latter, we investigated 45 novel cell lines from matched primary–metastasis mPACA pairs. In all cases with inferable sequence of genetic events, *Cdkn2a*^*HOM*^ preceded acquisition of *Kras*^*G12D-iGD*^ (Fig. 5j and Extended Data Fig. 12a). These results are consistent with *Cdkn2a* loss licensing oncogenic signalling amplification in the pancreas. Indeed, we observed that PanINs of *Ptf1a*^*cre/+*^*;Kras*^*LSL-G12D/+*^*;Cdkn2a*^*fl/fl*^ mice have strongly increased rates of *Kras*^*G12D-iGD*^ as compared to *Ptf1a*^*cre/+*^*;Kras*^*LSL-G12D/+*^ mice (Extended Data Fig. 7c,d). Overall, these findings highlight that *Kras*^*MUT*^ gene dosage increase is contingent on homozygous *Cdkn2a* inactivation in the pancreas, but not in the lung and intestine.

To investigate whether mutation patterns in human samples can be reconciled with the evolutionary principles identified in mice, we used TCGA and ICGC data for various correlative analyses. The low number of *CDKN2A*^*HOM*^ intestinal cancers (2 out of 136) prevented meaningful analyses in this entity. In the mouse lung, acquisition of *Cdkn2a*^*HOM*^ after *Kras*^*G12D-iGD*^ suggests that *Cdkn2a* inactivation provides a late-stage selective advantage. Indeed, human LUCA with *CDKN2A*^*HOM*^ loss displayed enhanced *KRAS* signalling and reduced survival compared with *CDKN2A*^*HET/WT*^ cancers (Extended Data Fig. 12b,c and Supplementary Table 16). Such late evolutionary advantage could explain why *CDKN2A*^*HOM*^ is more frequent in LUCA cell lines versus tissue collections (Fig. 5a,b, Extended Data Fig. 12d,e and Supplementary Table 17). Thus, while *CDKN2A*^*HOM*^ is not required for *KRAS*^*MUT-iGD*^ acquisition in the lung, it might subsequently provide a selective advantage by allowing unrestrained *KRAS* signalling.

In the mouse pancreas, we showed earlier that *Kras*^*G12D-iGD*^ can be licensed by *Cdkn2a*^*HOM*^ but also by *Trp53* loss^18^. To study these genetic dependencies in humans, we examined the frequency of *KRAS*^*MUT-iGD*^ in PACA proficient for *CDKN2A* and *TP53* (*CDKN2A*^*PROF*^*TP53*^*PROF*^). As *CDKN2A*^*PROF*^*TP53*^*PROF*^ pancreatic cancers are infrequent (<10%), we combined the COMPASS and TCGA-PAAD cohorts. Notably, *KRAS*^*MUT-iGD*^ was frequent in *CDKN2A*^*HOM*^ and/or *TP53*^*HOM*^ cancers (68 out of 185) but did not occur in *CDKN2A*^*PROF*^*TP53*^*PROF*^ pancreatic cancers (0 out of 16) (Fig. 5k and Supplementary Table 18). These data demonstrate that the contingency of *KRAS*^*MUT-iGD*^ on preceding *CDKN2A* loss not only applies to mice but also to human PACA evolution. By contrast, in human LUCA and COCA, the frequency of *KRAS*^*MUT-iGD*^ is independent of TSG status (Fig. 5k and Supplementary Table 18)—consistent with the mouse data.

Thus, oncogene–tumour suppressor interactions display marked tissue specificity. In the pancreas, *CDKN2A*^*HOM*^ precedes and licences *KRAS*^*MUT-iGD*^ during early cancer evolution, whereas *KRAS*^*MUT-iGD*^ is compatible with *CDKN2A* proficiency in the lung and intestine. However, in these organs, late-stage *CDKN2A*^*HOM*^ acquisition can provide a selective advantage through increased oncogenic signalling and tumour aggressiveness.

## *KRAS*–TSG interactions across cancer types

To examine whether these principles of oncogene–tumour suppressor interaction are generalizable beyond pancreatic, lung and intestinal cancer, we extended our analyses to TCGA data encompassing over 10,000 human samples from 33 cancer types. We selected entities comprising large numbers of cases with *KRAS* mutations, including bladder, rectal, stomach and uterine carcinomas. To determine *KRAS*^*MUT*^ and *CDKN2A* gene dosage, we purity-corrected tissue-derived SNV and CNV data (Methods). Moreover, we analysed public human scRNA-seq data to quantify *CDKN2A* expression in healthy epithelial cells, serving as potential cell of origin for the selected cancer types^47–49^. Integrated data analysis revealed that *CDKN2A*^*HOM*^ is most frequent in bladder cancer (50% of cases), consistent with frequent occurrence of *KRAS*^*MUT-iGD*^ in this cancer type and high *CDKN2A* activity in bladder epithelial cells (Fig. 5l–n). By contrast, *CDKN2A* loss is less frequent in tissues with low *CDKN2A* expression and reduced occurrence of *KRAS*^*MUT-iGD*^ (*CDKN2A*^*HOM*^ in 0% of rectal, 12% of stomach and 0% of uterine carcinomas). Overall, findings from this expanded set of tissues support a model in which *KRAS* signalling strength and tissue-specific *CDKN2A* chromatin states jointly shape the *CDKN2A* response and the selective pressure to inactivate this tumour-suppressive barrier.

## Discussion

Our study describes the MCCA, a comprehensive cancer cell resource for mice, encompassing 590 lines from 46 cancer entities and subentities. MCCA comprises large datasets, including molecular profiles and phenotypic annotations of cell lines and mice. The availability of clinical metadata, biobanked tissues and matched normal material further supports broad applicability of the MCCA in biomedical research. All data are accessible through a user-friendly cBioPortal web interface (www.mcca.tum.de) for versatile data mining and integrative analyses, such as the exploration of genotype–phenotype relationships and their context dependencies.

Cellular models constitute a major pillar of functional experimentation, not least given their ease of manipulation and (high throughput) perturbation^6–8,50^. The opportunities for mechanistic research offered by MCCA are potentiated by the immunocompetent transplantability of the resource. We developed computational methods to precisely determine strain composition and MHC haplotypes from genomic data, enabling us to predict immunocompatibility and guide selection of suitable hosts. We found that even extensive SNP mismatch can be compatible with engraftment in MHC-matched transplantations, but the extent of SNP divergence affects the experimental outcomes. This highlights the importance of strain and MHC annotation for MCCA, which will facilitate the in vivo investigation of a broad spectrum of research questions.

MCCA-based analyses of *KRAS*-initiated cancers together with human investigations and functional studies in mice highlighted general principles of tissue-specific cancer evolution, with marked differences between quiescent and proliferative organs (Fig. 5o).

The pancreas is a prototypical tissue in which oncogenesis starts in a terminally differentiated non-proliferative compartment (acinar or ductal cells). Here, the critical first step in oncogenesis is de-differentiation (Fig. 5o). We found that this process is *KRAS*^*MUT*^ dosage sensitive, explaining our previous observation that *KRAS*^*MUT-iGD*^ is acquired early during cancer evolution^18^. The associated engagement of *CDKN2A*, enabled by the active *CDKN2A* chromatin state in differentiated pancreatic cells, constitutes a strong barrier to early tumour progression. Consequently, the selective pressure to inactivate *CDKN2A* is high, and its loss needs to precede *KRAS*^*MUT-iGD*^.

In the lung, we also observed *KRAS*^*MUT*^-dosage-dependent induction of developmental programs. However, in contrast to pancreatic acinar or ductal cells, lung alveolar cells displayed low *CDKN2A* activity. Increased tolerance to *KRAS*^*MUT*^ signalling amplification in these cells explains why homozygous *CDKN2A* loss is less frequent in human LUCA (15%) than in human PACA (64%). Moreover, as *KRAS*^*MUT*^ gene dosage increase is not severely constrained by *CDKN2A* in the lung, the order of *KRAS*^*MUT*^ and *CDKN2A*^*HOM*^ acquisition can be reverse to that observed in the pancreas (Fig. 5o). Finally, cell-type-specific chromatin states (and the resulting gatekeeper activity) of *CDKN2A* explain tissue-specific oncogenicity of *KRAS*^*MUT*^: whereas carcinogenesis is rapid and multifocal in the mouse lung, only one cancer evolves after long time frames in the mouse pancreas.

The intestine is the prototype of a highly proliferative epithelial tissue. Owing to high cellular turnover rates, oncogenesis requires acquisition of *KRAS*^*MUT*^ in long-lived stem or precursor cells. However, clonal expansion of *KRAS*^*MUT*^ beyond the crypt relies on block of differentiation, as does expansion of subsequent *KRAS*^*MUT-iGD*^. In the intestine, this cannot be induced by *KRAS*, but depends on WNT signalling activation, the hallmark event for adenoma formation. Thus, only from this stage onwards can *KRAS*^*MUT-iGD*^ clonally expand and drive aggressive growth and invasion (Fig. 5o). Although *CDKN2A* activity is very low in the intestine (as in lung), *Vil-cre;Kras*^*LSL-G12D/+*^ mice rarely develop carcinomas, even when aged up to 2 years. This apparent paradox can be explained by the contingency of *KRAS*^*MUT*^ on sporadic acquisition of WNT pathway alterations to drive cancer evolution in the intestine.

Our results shed light on central mechanisms underlying tissue-specific oncogene–tumour suppressor collaboration. We highlight genetic interactions, pinpoint the processes that they drive, the stage at which they occur and the dosage at which they interact in different tissues. The varying ability of *CDKN2A* to restrain *KRAS*-induced oncogenesis in different tissues translates into distinct frequencies of complete, partial or lacking *CDKN2A* inactivation (Fig. 5a,b). Moreover, *CDKN2A* haploinsufficiency displays genetic context dependencies, even within the same tissue, as exemplified in the pancreas, in which partial *CDKN2A* inactivation is associated with *KRAS*^*MUT-HET*^ (Fig. 5k). Such findings are consistent with a continuum model of tumour suppressor and oncogene function^51^ and the existence of extensive dosage sensitivities in oncogenesis^52^. We show that gene-dosage sweet spots in genetic interactions are not random but are demarcated by context-specific evolutionary constraints and contingencies. These considerations might also be relevant to cancer prevention and treatment, as both oncogenic dosage increase and co-deletions with *CDKN2A* have been implicated in therapy response and resistance^53,54^.

Overall, our study identifies rules, molecular hallmarks and mechanistic principles defining *KRAS*-initiated cancer evolution in different tissues. The results support a deterministic model of cancer evolution with predictable trajectories that explain the tissue-specific patterns of genomic alterations in human cancers. This work was triggered and developed by key investigations of the MCCA resource, which will be continuously expanded to advance mechanistic and translational cancer research.

## Methods

### Cell line collection, characterization, maintenance and dissemination

MCCA lines were generated either in-house, provided by collaborators or obtained from public repositories. Details of the original source (laboratory or commercial vendor) of each MCCA line are provided in Supplementary Table 1. In case a cell line has been published previously, the original research article for each individual MCCA line is referenced in Supplementary Table 1. MCCA lines were maintained under cell-line-specific conditions. For each MCCA line, the medium composition (basal medium, growth factors and so on) and cell culture requirements (adherent/suspension, extracellular matrix/coating) are provided in Supplementary Table 1.

To ensure the quality, utility and long-term preservation of MCCA cell lines, a strategy for MCCA handling and maintenance was established that covers several aspects. First, rigorous quality controls are performed, ranging from regular mycoplasma testing and human DNA detection PCRs to regenotyping of mouse alleles to prevent contamination or misidentification of cell lines. Moreover, the recombination of genetically engineered alleles was tested in cell lines directly after their isolation from mice to detect potential fibroblast contaminations (which were removed through differential trypsinization). Second, standardized protocols for the culture and maintenance of cell lines were implemented, encompassing (1) the assessment of cell density before molecular characterization; (2) the propagation of cell lines using splitting ratios adapted to proliferation rate; and (3) the amplification of cell lines from the same pool of original cells to minimize variation between batches of cells. Third, comprehensive phenotypic and molecular characterization of cell lines were performed using standardized analytical approaches. To ensure a high quality of analyses, computational approaches optimized for the mouse were applied^13^. Fourth, to ensure secure long-term preservation of the MCCA resource, backup vials for each line have been archived at at least two independent locations. Finally, all information relevant for the request of MCCA lines can be found on the ‘Resource availability’ page at www.mcca.tum.de.

All characterization data generated as part of MCCA are publicly available through a mouse-specific cBioPortal instance (branched from main v.3.7.1) at www.mcca.tum.de.

### Animal cohorts and experiments

Mice were housed under specific-pathogen-free conditions in groups of up to five animals per cage under a 12 h–12 h light–dark cycle at 21–22 °C temperature and 45–65% relative humidity, and supplied ad libitum with standard chow and water. Female and male mice were randomly submitted to respective tumour cohorts. The maximal tumour size/burden permitted by the IACUC and the local authorities (Regierung von Oberbayern) is 1.5 cm in diameter, which was not exceeded in our study. All animal studies were conducted in compliance with European guidelines for the care and use of laboratory animals and were approved by the Institutional Animal Care and Use Committees (IACUC) of the Technische Universität München, Regierung von Oberbayern and the UK Home Office. All genetically engineered mouse alleles included in this study are referenced in Supplementary Table 1.

### Histopathological analyses

For histological characterization, 2-μm-thick specimens from formalin-fixed paraffin-embedded material were routinely stained with haematoxylin and eosin (H&E), scanned (Leica LAS X, v.3.7.5.24914; Leica Aperio ImageScope, v.12.4.3.5008) and submitted to at least two veterinary pathologists experienced in comparative cancer pathology in mouse models. Histomorphological evaluation and tumour grading were performed according to the guidelines of the MMHCC (Mouse Models of Human Cancers Consortium (NIH/National Cancer Institute)) and organ-specific INHAND classifications^57^. If required, immunohistochemistry was performed to validate H&E-based histopathological classification.

### gDNA and RNA isolation

Cells were cultured according to the conditions described in Supplementary Table 1. Isolation of genomic DNA (gDNA) and RNA was conducted according to the manufacturer’s instructions using the DNeasy Blood & Tissue Kit (Qiagen) and the RNeasy Kit (Qiagen), respectively. For gDNA isolation, frozen cell pellets were used. For the collection of RNA, cells were cultured in the corresponding culture medium and immediately lysed with RLT buffer (Qiagen) containing β-mercaptoethanol and homogenized with QIAshredder columns (Qiagen) before proceeding with RNA isolation. gDNA and RNA concentrations were determined using a Qubit fluorometer (Thermo Fisher Scientific).

### Genomic sequencing of MCCA lines

Whole-exome sequencing (WES) was performed using 450 ng of gDNA from mouse cell lines and matched normal samples from mouse tail biopsies. Coding exons were enriched by whole-exome pull-down using the Agilent SureSelect XT Mouse All Exon Kit according to the manufacturer’s instructions and sequenced on the NovaSeq 6000 (Illumina) system.

Low-coverage whole-genome sequencing (lcWGS) was performed using 200 ng of gDNA from mouse cell lines and matched normal samples from mouse tail biopsies when available. Libraries were prepared using the TruSeq DNA Nanokit (Illumina) according to the manufacturer’s instructions. The resulting libraries were analysed on the 2100 Bioanalyzer instrument (Agilent Technologies) and sequenced on the NextSeq 550 (Illumina) or NovaSeq 6000 (Illumina) system.

### Analysis of genomic sequencing data

The analysis of WES data from mouse tumour–normal sample pairs was performed according to the GATK best practice suggestions. The established MoCaSeq analysis pipeline (v.0.4.54)^13^ was used for processing all samples. Raw BCL files were converted into demultiplexed FASTQ files using bcl2fastq (v.2.20.0.422). Raw sequencing reads were trimmed using Trimmomatic (v.0.39)^58^, removing leading and trailing bases with Phred scores below 25 and reads with less than 50 nucleotides. Moreover, an average base quality of 25 was enforced with a sliding window of 10 nucleotides for the reads. Passing reads were then aligned to the GRCm38.p6 reference genome using BWA-MEM (v.0.7.17)^59^ with the default settings. The mapped reads were processed using samblaster (v.0.1.26)^60^, sambamba (v.0.7.0)^61^ and Picard tools (v.2.20.0). Mutect2 from the GATK toolkit (v.4.2.0.0)^62^ was used to call indels and somatic mutations with the default settings. Variants were filtered for read orientation artefacts using GATK. For each tumour sample, the corresponding normal sample was used to filter germline variants. Moreover, candidate somatic mutations were filtered for SNPs by excluding variants listed in the Wellcome Trust Sanger Mouse Genome Project SNP database (v5) (ENA study PRJEB11471). Furthermore, somatic mutations were filtered if (1) the read coverage was below 5 in both the control and tumour; (2) the variant allele frequency (VAF) was below 5%; and (3) the number of reads carrying the variant was below 2 in the tumour sample and equal to 1 or 0 in the normal sample. Annotation of somatic variants was performed using SNPeff (v.4.3)^63^. SNVs with a low predicted impact as well as variants at non-exonic sites were excluded from further analysis. DNA tumour/normal copy ratios were determined using CNVKit (v.0.9.9)^64^. The copy-number calling was performed using the batch command of the CNVKit pipeline for read coverage estimation, normalization and segmentation. The probe regions of the Agilent SureSelect XT Mouse All Exon Kit were used as on-target regions.

The analysis of WES and WGS data from human tumour/normal sample pairs was performed based on a modified version of MoCaSeq adapted to the human genome. Raw sequencing data were obtained from TCGA (dbGAP study phs000178.v11.p8) and the ICGC PanCuRx study (EGA studies EGAD00001003585, EGAD00001004551, EGAD00001006081 and EGAD00001006152). Reads were aligned to the GRCh38.p12 reference genome. Variants were called using Mutect2 from the GATK toolkit (v.4.2.0.0)^62^. Genes were annotated using SNPeff (Ensembl 92) and Gencode (v.31)^65^. Potential somatic variants were filtered for SNPs by excluding SNVs listed in the GnomAD (>1%)^66^ and dbSNP (>5%)^67^ database. The copy ratios were determined by using the batch command of CNVKit (v.0.9.9) in WGS mode and Agilent SureSelect Human All Exon V7 exon probes (S31285117) as on-target regions. For the detection of microsatellite instability (MSI), MSIsensor (v.0.5)^68^ was run with the default parameters and using the GRCm38.p6 microsatellites data provided by the authors (but no evidence for MSI was found in the MCCA; Supplementary Table 6).

The lcWGS data were analysed analogously to the WES data regarding trimming, mapping and postprocessing. Copy-number calling was performed with CNVKit^64^ using the whole-genome sequencing mode combined with the Agilent SureSelect XT Mouse All Exon Kit exon probe regions as on-target regions, according to the CNVKit best practice suggestions. Postprocessing and data visualization were performed in R (v.4.4.1) using data.table (v.1.14.8), ggplot2 (v.3.4.2), pheatmap (v.1.0.12) and ComplexHeatmap (v.2.16.0).

### Purity correction and gene allele state analyses

To determine purity and ploidy values for cancer tissues, we reanalysed TCGA cancer tissue samples using ABSOLUTE^69^ (v.1.0.6) with the default parameters and total copy number as well as mutation data as input. Purity and ploidy estimates from ABSOLUTE were reviewed and curated based on manual inspection of copy-number profiles and SNP/SNV frequencies for each sample and compared to the purity and ploidy values as provided by the PanCanAtlas (https://gdc.cancer.gov/about-data/publications/pancanatlas). The consensus genomic tumour purity value of each sample was then used to bioinformatically adjust the VAF of SNVs as well as the copy ratio of CNV segments as they would be detected in pure cancer cells. The purity and ploidy values for cancer tissues of the ICGC PanCuRx cohort were estimated similarly to as described above (including manual review of purity/ploidy solutions and bioinformatical purity adjustment of SNV VAFs and CNV copy ratios). Only human cancer tissues with *KRAS* exon 2 hotspot mutations (G12* or G13*; referred to as *KRAS*^*MUT*^) were considered for further downstream analyses. Moreover, human cancers with a purity below 20% were excluded from the analyses of *KRAS*, *CDKN2A* and *TP53* allele states to ensure robust detection of allelic imbalances and/or deletions. Estimating tumour purity and gene allele state based on genomics data from bulk tissue samples can be complicated by purity–ploidy ambiguity (multiple possible combinations matching a single genomic profile), intratumour heterogeneity (subclonal copy-number alterations appear with attenuated log_2_ ratios) and low tumour content (increasing stroma and immune cell admixture masking tumour-derived signals). These potential confounders were accounted for in the data analysis by manual review of all candidate purity–ploidy solutions through two independent experts, by evaluation of allelic imbalance in the dominant tumour clone and by exclusion of samples with tumour purity below 20%, respectively. Given the typical low purity of pancreatic cancer tissue samples, analyses of gene allele states in this entity were primarily performed using the ICGC PanCuRx cohort, which encompasses genomics data generated from laser microdissected pancreatic cancer tissues (as opposed to the TCGA-PAAD dataset).

For microdissected MCCA tissues, purity correction of *Kras*^*G12D*^ VAFs was performed based on the quantification of non-recombined *Kras*^*LSL-G12D*^ alleles. Custom-designed TaqMan quantitative PCR (qPCR) assays were used to determine *Kras*^*LSL-G12D*^ allele and total *Kras* locus copy (*Kras*^*Copy*^) quantities (Supplementary Table 19). *Kras*^*LSL-G12D*^ quantities were normalized to *Kras*^*Copy*^ to account for potential copy-number changes at the *Kras* locus in cancer cells. Normalized *Kras*^*LSL-G12D*^ values directly reflect stroma contamination and were used for bioinformatical purity adjustment of tissue-based *Kras*^*G12D*^ VAFs. For this, contaminating stroma reads were subtracted from tissue-based amplicon-based next-generation-sequencing data of the *Kras* locus to finally obtain pure *Kras*^*G12D*^ VAFs.

For the analysis of gene allele states, processed VAFs and copy number ratios were integrated. Purity-adjusted VAF and copy ratio (CR) values were used for cancer tissues, but not cell lines. For analyses of PAAD, NSCLC and COAD cohorts of the CCLE dataset^6^, processed VAF and CR data were downloaded from the cBioPortal study, Cancer Cell Line Encyclopedia^6^ (https://www.cbioportal.org/study/summary?id=cellline_ccle_broad). *Kras*^*G12D*^ (mouse) and *KRAS*^*MUT*^ (human) allelic states were classified using the following thresholds for VAF and CR: dGD (0.05 ≤ VAF < 0.4), HET (0.4 ≤ VAF < 0.61), gain (VAF ≥ 0.61 and 1.3 ≤ CR < 2.8), amp (VAF ≥ 0.61 and CR ≥ 2.8) and LOH (VAF ≥ 0.61 and CR < 1.3). *Cdkn2a* (mouse) and *CDKN2A* (human) allelic states were classified as follows: WT (VAF = 0 and CR ≥ 0.87), HET (0 < VAF < 0.85 or 0.19 < CR < 0.87) and HOM (VAF ≥ 0.85 or CR ≤ 0.19).

### Analysis of TMB and CNV load

For the analysis of TMB in MCCA, cell lines with available WES data of the cancer and matched normal control were used, resulting in a final set of 190 samples from the intestine, liver, lung, pancreas and stomach. Somatic mutations were retained if they met the following criteria: (1) read coverage ≥10 at the variant site in both tumour and matched normal sample; (2) VAF ≥ 10%; (3) ≥3 variant-supporting reads in the tumour; and (4) no variant-supporting reads in the matched normal. Protein-coding exon coordinates were obtained from the GENCODE M25 annotation (*Mus musculus*), including all exons from protein-coding transcripts. Overlapping regions were collapsed to generate a non-redundant, non-overlapping set of exonic intervals representing the protein-coding exome. Mutations outside these regions were excluded. TMB was calculated as the number of all protein-coding exonic mutations divided by the total exonic length (in megabases). A detailed description for the analysis of the ‘effective’ pTMB in different transplantation scenarios is provided in the ‘Immunophenotyping’ section. For the TMB analyses in human cancer cell lines, pre-processed somatic mutation calls were retrieved from DepMap 24Q4 (10.25452/figshare.plus.27993248.v1; file, OmicsSomaticMutations.csv)^50^ for the same set of CCLE samples of ref. ^7^ that was also used for the cross-species comparison of MCCA and CCLE transcriptomes. Samples corresponding to the same tissue types as those selected in MCCA were retained, resulting in 336 CCLE samples. The exome was defined using GENCODE v38 (*Homo sapiens*) according to the same collapsing strategy as for MCCA. As matched normal samples are not available for CCLE, mutations were retained if they met the following criteria: (1) read coverage ≥ 10; (2) VAF ≥ 10%; and (3) ≥3 variant-supporting reads in the tumour. TMB was computed as above (and pTMB as described in the ‘Immunophenotyping’ section). For TCGA samples, WES data were obtained for tumours and matched normal controls as described above. Samples with annotated low quality or low tumour purity estimates (<40%) were excluded. To avoid patient-level redundancy, when multiple samples were available from the same individual, one sample was randomly selected. Samples matching the selected MCCA tissue types were retained, yielding 1,551 samples. For the selected samples, somatic mutation calls were obtained from the GDC data portal and VAFs were purity-corrected as described below. Mutations were filtered using the same criteria as for MCCA, and the exome was defined as for CCLE. TMB was calculated using the same approach.

For the analysis of CNV load in MCCA, copy-number profiles generated by CNVkit (see the ‘Analysis of genomic sequencing data’ section) were obtained for 590 MCCA cell lines as determined by WES (*n* = 200) or lcWGS (*n* = 390). Tissues represented by at least five MCCA lines were selected for downstream analyses, including soft tissue, bile duct, intestine, liver, lung, pancreas, stomach, lymphoid T, lymphoid B, myeloid, nervous system and oesophagus, resulting in a final set of 562 samples. CNV load was calculated as the percentage of the autosomal genome affected by copy-number alterations. In detail, genomic segments with an absolute log_2_-transformed copy ratio of >0.2 were defined as altered, and their total length was divided by the total autosomal genome length and multiplied by 100. For the CCLE cell lines reported in ref. ^7^, preprocessed copy-number segment profiles were obtained from cBioPortal^15^. Samples corresponding to the same tissue types as those selected in MCCA were retained, resulting in 606 samples. CNV load was computed using the same approach as for MCCA using R (v.4.4.1) and with data.table (v.1.14.8). For TCGA, pre-processed copy-number segment data were obtained as described above and samples with annotated low quality or low tumour purity estimates (<40%) were excluded. When multiple samples were available from the same individual, one was randomly selected. Samples matching the selected MCCA tissue types were retained, yielding 1,962 samples. Copy-number ratios for the selected samples were retrieved from the GDC portal and purity-corrected as described below. CNV load was calculated as described for MCCA and CCLE.

The weighted genome instability index (wGII) was determined as described previously^70^. wGII estimates copy-number instability based on the proportion of the genome with aberrant copy number compared with the median ploidy, weighted on a per chromosome basis; and correlates with copy-number instability in cancer cell lines.

### Immunophenotyping

For the analysis of mouse genetic background (strain) from genomic sequencing data, a library of strain-specific signature SNPs was first established. For this, a total of 21,923,209 SNPs was extracted from the whole-genome sequencing catalogue of the Mouse Genomes Project, which comprises 29 widely used inbred mouse strains^20^. Pearson correlation of SNP patterns was calculated for each pairwise inbred strain combination. Correlation values were used for hierarchical clustering to assign the 29 inbred mouse strains into 15 genealogically related strain groups. Strain-specific marker SNPs were required to be unique to a particular strain group but were allowed to be shared within the same group. In total, 1,097,314 genome-wide signature SNPs were determined. This library of signature SNPs was then used to infer the percentage strain composition of MCCA lines from genomic sequencing data. For this purpose, the genome was binned into consecutive 10 Mb bins. Strain-specific signature SNPs were assigned to each bin according to their genomic position. Genomic sequencing data were used for performing variant calling and SNP detection in MCCA lines. The list of identified SNP variants was matched against the library of strain-specific signature SNPs. An enrichment score was calculated for each bin per strain, based on the number of matching SNPs and normalized to the number of expected SNPs. The length of all bins with a sufficient enrichment score was summed to derive a genome-wide enrichment score for each of the 29 inbred strains. Genome-wide enrichment scores for the top three highest scoring strains were reported for each MCCA line (Supplementary Table 5).

The backcross status of MCCA lines was estimated from sequencing data (genomic strain composition; Supplementary Table 5) based on the following equation:

$${\mathrm{cBS}}_{{N}}={\log }_{2}\,\left(\frac{2\times 100 \% }{\sum {\mathrm{FS}}_{ \% }-\sum {\mathrm{AS}}_{ \% }}\right)$$

where cBS_*N*_ is the computational backcross status, $\sum {{\rm{FS}}}_{ \% }$ is the sum of foreign strain contribution and $\sum {{\rm{AS}}}_{ \% }$ is the sum of allele-clustered SNP contribution). Three specific considerations are accounted for in the equation. Related to ${\log }_{2}()$: each successive backcross generation reduces the genetic contribution of the original strain background by 50% in the genome of the backcrossed mouse. Backcross generation can therefore be inferred by calculating the log_2_ from the fraction of the original strain background remaining in the backcrossed mouse genome. Related to $(2\times 100 \% )$: Both copies of a diploid genome (the maternal and paternal) need to be backcrossed and considered in the analyses. Related to $(\sum {{\rm{FS}}}_{ \% }-\sum {{\rm{AS}}}_{ \% })$: SNPs clustered around non-congenic alleles withstand backcrossing and thereby confound the calculation of backcross status. We therefore filter SNPs found in close genomic proximity to non-congenic mouse alleles. To this end, the genetic background (strain) of the ES cell, in which a mouse allele was originally engineered, was annotated for the mouse alleles present in each individual MCCA line (Supplementary Table 1). This information was then used to remove SNPs corresponding to the strain of a given allele within a 25 Mb window of its genomic integration site (for mouse alleles with unknown genomic integration sites, a value of 1% per allele was subtracted from the total enrichment score of the corresponding genetic background). This procedure was performed for all genetically engineered alleles of a given MCCA line to finally compute allele-adjusted enrichment scores for each detected strain contribution (Supplementary Table 5).

For MHC haplotype analysis from genomic sequencing data, a library of MHC-specific signature SNPs was generated first. For this, the MHC locus was divided into 6 gene clusters (*H2-K*, *-A*, *-E*, *-D*, *-Q* and *-T*) based on their MHC subclass assignment (class I or II, classical or non-classical). The identification of MHC-specific signature SNPs was performed in a way that is comparable to the identification of strain signature SNPs but for each *H2* gene cluster individually. First, a total of 375,097 MHC SNPs was derived from the whole-genome sequencing catalogue of the Mouse Genomes Project, comprising 29 inbred mouse strains^20^. In the second step, Pearson correlation of SNP patterns with hierarchical clustering was used to define MHC haplotypes for each MHC gene cluster individually. Identified *H2* gene cluster haplotype groups were verified using existing MHC haplotype information, if available (https://www.imgt.org/IMGTrepertoireMH/Polymorphism/haplotypes/mouse/MHC/Mu_haplotypes.html). For each individual *H2* gene cluster, signature SNPs were required to be exclusive to a particular haplotype group but were allowed to be shared within the same group. In total, 44,219 MHC gene cluster signature SNPs were identified. This panel of signature SNPs was finally used to determine *H2* gene cluster-specific MHC haplotypes for MCCA lines based on genomic sequencing data. Such as for the genome-wide strain analysis, MHC gene clusters were binned (here into segments of 1 Mb size) and the enrichment score for each bin was calculated. On the basis of the enrichment scores, the MHC haplotype was assigned to each of the 6 *H2* gene clusters, which, in combination, defines the full MHC haplotype of an individual MCCA line (Supplementary Table 5).

For sex analysis, the number of uniquely mapped reads was calculated for each chromosome using samtools coverage (v.1.17). Next, the ratio of Y-chromosome-specific read counts over the sum of all autosome-specific read counts was calculated. On the basis of samples with available sex annotation, a cut-off of 0.05 was selected for the Y chromosome mapping ratio to assign male (≥0.05) or female (<0.05) sex (Supplementary Table 5).

For the quantification of the ‘effective’ pTMB of MCCA lines in defined immunocompetent transplantation scenarios, information on genetic background (strain), MHC haplotyping and TMB were integrated. The TMB was analysed as described in the ‘Analysis of TMB and CNV load’ section, but with two modifications. First, variants detected in the tumour were not filtered for their presence or absence in the matched normal sample, as somatic and germline variants need to be considered for this type of analysis. Second, as only non-synonymous (protein-altering) variants can be potentially immunogenic in immunocompetent settings, the TMB output was filtered for variants with a predicted impact classified as MODERATE or HIGH based on the Ensembl Variant Effect Predictor (referred to as the TMB of protein-coding alterations, pTMB). These variants were then annotated as either somatic (present in the tumour, absent in the normal) or germline (present in both tumour and normal). Germline variants were further required (1) to be reported as a strain-specific germline variant in the SNP catalogue of the Mouse Genomes Project^20^; (2) to match the genetic background annotation of the corresponding MCCA line as provided in Supplementary Table 5; and (3) to localize outside of SNP-dense genomic regions (which are indicative of errors in the genome assembly, as reported previously^71^). The ‘effective’ pTMB of a given MCCA line was then calculated from its protein-altering germline variants that do not match the genetic background of the corresponding recipient mouse (plus the protein-altering somatic mutations found in the same MCCA line). As the ‘effective’ pTMB of an MCCA line is recipient dependent, two distinct scenarios of immunocompetent transplantation were analysed. Scenario 1: MHC and the first (if possible, also the second) most dominant genetic background detected in an MCCA line match the recipient (reduced contribution of germline variants to the ‘effective’ pTMB). Scenario 2: MHC but only the most dominant genetic background detected in an MCCA line do match the recipient (elevated contribution of germline variants to the ‘effective’ pTMB).

### 3′ RNA-seq

Library preparation for bulk-sequencing of poly(A)-RNA was done as described previously^72^. In brief, barcoded cDNA of each sample was generated with Maxima RT polymerase (Thermo Fisher Scientific) using oligo-dT primer containing barcodes, unique molecular identifiers (UMIs) and an adaptor. The ends of the cDNAs were extended by a template switch oligo (TSO) and full-length cDNA was amplified with primers binding to the TSO site and the adaptor. The NEBNext Ultra II FS kit was used to fragment cDNA. After end-repair and A-tailing, a TruSeq adapter was ligated, and 3′-end fragments were finally amplified using primers with Illumina P5 and P7 overhangs. In comparison to a previous study^72^, the P5 and P7 sites were exchanged to allow sequencing of the cDNA in read1 and barcodes and UMIs in read2 to achieve a better cluster recognition. The library was sequenced on the NextSeq 550 (Illumina) system with 63 cycles for the cDNA in read1 and 16 cycles for the barcodes and UMIs in read2.

The 3′ RNA-seq data were processed using the published Drop-seq pipeline (v1.0) to generate sample- and gene-wise UMI tables^73^. The reference genomes GRCm38 and GRCh38 were used for alignment of mouse and human samples, respectively. Transcript and gene definitions were used according to the Gencode (v.38)^65^. The data were processed in R using the DESeq2 package (v.1.46.0) for read normalization and variance stabilizing transformation^74^.

### Analysis of transcriptome data

Batch correction was performed to obtain a single, full MCCA transcriptome dataset. Transcriptomes of MCCA lines were generated by 3′ RNA-seq in three independent batches (batch B1–B3), and each individual cell line was sequenced in technical replicates. To facilitate batch correction, the largest batch (B1) included 53 reference samples that were matched to B2 (27 reference samples) and B3 (26 reference samples). B1, B2 and B3 count matrices were transformed using the variance stabilizing transformation (vst()) function of the DESeq2 package (v.1.46.0)^74^. Next, the batch effect was examined based on the clustering of MCCA batches and reference samples in dimensionality-reduction plots (PCA and UMAP). One technical replicate from B1 was removed owing to a clear separation from the other technical replicates of the same sample. Next, the duplicate correlation (dupcor()) function of limma (v.3.5.4)^75^ was used to compute the correlation of technical replicates for matching reference samples across batches. The dupcor() function estimates the correlation between duplicates (here matching reference samples) by fitting a mixed linear model individually for each gene. Finally, to remove the batch effect, the removeBatchEffect function of limma was applied by using lineage information (as covariate), the batch information and the consensus duplicate correlation (returned by dupcor). The resulting batch corrected expression matrix was evaluated for the absence of batch effects using dimensionality-reduction plots (PCA and UMAP). Last, the gene coefficients obtained from removeBatchEffect were retained and the duplicated reference samples (from B2 and B3) were removed from the dataset.

GSVA was performed on rlog-normalized gene expression data using the GSVA R package (v.1.52.3)^76^. Gene set libraries MSigDb-Hallmark-2020, KEGG-2021 and WikiPathway-2021 of enrichR (v.1.62.0)^77^ as well as gene sets described previously^26,55,56^ were used for analyses. Gaussian kernel was used for nonparametric estimation of the cumulative distribution function of (sorted) expression levels, and normalized GSVA scores were extracted.

To assess pathways enriched in hepatic cell lines of MCCA cultured in 2D versus 3D Matrigel conditions, RNA-seq raw counts were normalized using the median-of-ratios method and variance-stabilized with the rlog transformation in DESeq2 (v.1.46.0) under R (v.4.4.1). Gene set enrichment analysis was conducted in enrichR (v.1.62.0), using the MSigDb-Hallmark-2020 gene set library.

For *KRAS*^*G12D*^ overexpression experiments, count matrices were transformed using variance-stabilizing transformation as described in the ‘3′ RNA-seq’ section. Genes with a low sum of counts across all samples were removed (≥30 for CACO2, HBEC3KT and HPDE; ≥60 for HCEC1CT, MODEK and 266-6). The PCA was conducted on rlog-normalized data by selecting the top 10% of the most variable protein-coding genes based on their s.d. across samples. For a given principal component, the 250 genes with the highest positive loadings and the 250 genes with the most negative loadings were extracted. Loadings represent the coefficients that quantify how strongly each gene contributes to the variance captured by a principal component. Positive and negative loadings correspond to genes driving the separation of samples toward opposite directions along the principal component axis, reflecting, for example, the *KRAS*^*G12D*^ dosage-dependent induction of transcriptional changes on PC1. Gene set enrichment analysis of these gene sets was performed using enrichR (v.1.62.0)^77^. The gene set libraries MSigDb-Hallmark-2020, KEGG-2021 and GO-Biological-Process2023 of enrichR (v.1.62.0)^77^ were analysed.

For the comparison of transcriptomes of acinar cells 0 and 24 h after explantation from *Ptf1a*^*cre/+*^*;Kras*^*LSL-G12D/+*^ mice, RNA-seq raw counts were normalized using the median-of-ratios method and variance-stabilized with rlog transformation in DESeq2 (v.1.46.0) under R (v.4.4.1). Differential expression analysis was performed with DESeq2, and genes were considered to be differentially expressed at a FDR < 0.05. Significance values for *Kras* were extracted accordingly.

Raw count RNA-seq data from the TCGA-LUAD were downloaded through the GDC data portal (https://portal.gdc.cancer.gov/). Only cancer tissues with *KRAS* exon2 hotspot mutations (G12* or G13*) were considered for analysis. Differential expression analysis was performed in R (v.4.4.1) with Limma (v.3.5.4)^75^. A gene was considered to be differentially expressed if its FDR-adjusted *P* value was below 0.05. Gene set enrichment analysis was performed using enrichR (v.1.62.0)^77^ with the gene set libraries MSigDb-Hallmark-2020 and KEGG-2021. Postprocessing and data visualization were performed in R (v4.4.1) using data.table (v.1.14.8), ggplot2 (v.3.4.2), pheatmap (v.1.0.12) and ComplexHeatmap (v.2.16.0).

### Genome and transcriptome stability of cell lines

To determine the stability of genomes and transcriptomes of mouse cancer cell lines, pancreatic cancer cell lines that have been cultured and characterized multiple times over a period of 10 years (up to 13 passages difference) was used (Supplementary Table 2). Cell lines that contained mixed populations of epithelial and mesenchymal cells were not included (to avoid confounding effects on the analysis of transcriptome stability), resulting in a set of 30 cell lines shared between both datasets.

The stability of mouse pancreatic cancer cell line transcriptomes was assessed by comparing cell lines cultured and profiled by RNA-seq in 2022 (data from MCCA) and 2016 (data from ref. ^18^). RNA-seq raw counts from both cohorts were normalized using the median-of-ratios method and variance-stabilized with rlog transformation using DESeq2 (v.1.46.0) in R (v.4.4.1). The top 10% most variable genes, ranked by s.d., were selected and used to cluster the samples by Euclidean distance and complete linkage. Cell lines were assigned into transcriptomic clusters in each dataset to assess the similarity of transcriptome clustering for each individual sample over time and passages.

Genome stability was investigated by comparing the log_2_-transformed copy ratio values of copy-number profiles for the same cell line as detected by WES in 2012 (data from ref. ^18^) or lcWGS in 2022 (data from MCCA). Both datasets were analysed using MoCaSeq (described in the ‘Analysis of genomic sequencing data’ section). Copy-number segments generated by HMMCopy were binned to 1 Mb intervals for each chromosome. If a bin contained two different log_2_-transformed values, a weighted mean (based on the size of each overlap) was calculated to assign a single log_2_-transformed value to the bin. The bins were then smoothed using the median and a window size of 5 bins, excluding chromosomes associated with chromothripsis and other complex rearrangements. For a cell line with sufficient copy-number changes (median log_2_ > 0), the median log_2_-transformed value was subtracted from every bin to recentre the data. To quantify the stability of a cell line, the Euclidean distance between the log_2_-transformed copy ratio value of WES and lcWGS data was calculated per bin and averaged across each chromosome.

To compare the genome stability of MCCA lines to human models, two separately generated copy-number datasets of large-scale human cancer cell line projects were used: CCLE (Broad Institute) and Genomics of Drug Sensitivity in Cancer (GDSC, Sanger Institute). Genome stability was investigated by comparing the log_2_-transformed copy ratio values of copy-number profiles for the same cell line as detected by WES (CCLE, data from ref. ^7^) or SNP array (GDSC, data obtained from https://cellmodelpassports.sanger.ac.uk/downloads). The copy-number profiles of 625 cell lines matched between CCLE and GDSC were analysed using the same analytical workflow as described above for the comparison of mouse cancer cell line genomes. Notably, a direct comparison of genome stability in mouse and human cancer cell lines has certain limitations. For example, while the majority of cell lines in CCLE and GDSC (473 out of 625) were obtained from the same supplier (Supplementary Table 2), the exact number of passages separating individual cell line pairs in both projects is unclear. Furthermore, the increased genome instability in human cell line pairs might be linked to the increased copy-number load observed in human cancers.

### Mouse–human cross-species comparison of cancer genomes and transcriptomes

For the mouse–human cross-species genome analyses, CNV profiles of MCCA lines were generated using CNVkit (v.0.9.9) with a bin size of 20 kb. Germline CNVs were manually filtered out before the analysis. For each cancer entity, consensus CNV profiles were obtained by binning the genome and calculating the average of normalized copy-number values at each genomic bin across all cancers of a given disease type. The resulting consensus plots provide entity-specific CNV landscapes and were used for the annotation of orthologues of recurrently amplified or deleted human cancer genes. These genes were identified for each disease type individually, by first assembling all genes affected by recurrent copy-number alterations in the corresponding human cancer type (CCLE and TCGA cohorts). In a second step, this list of genes was filtered for those genes reported in the Cancer Gene Census. Finally, a representative set of cancer genes was selected for each disease type, based on gene amplification/deletion frequency and literature search. These overlays enable direct comparison of mouse and human CNV patterns, highlighting conserved lineage- and entity-specific alterations.

For the mouse–human cross-species transcriptome analyses, gene-level RNA-seq count data for human cancer cell lines were obtained from the CCLE dataset provided by ref. ^7^ (file, CCLE_RNAseq_genes_counts_20180929.gct.gz). Counts were normalized using the median-of-ratios method and variance-stabilized by vst transformation using DESeq2 (v.1.46.0) in R (v.4.4.1). For mouse cancer cell lines from the MCCA dataset, previously generated batch-corrected variance-stabilized counts were used. Only tissues and tumour types represented in both MCCA and CCLE datasets were retained. Tissue nomenclature was manually curated to harmonize labels between datasets. Human–mouse orthologous gene names were obtained from Ensembl (v.103, 10.1093/nar/gkae1071) using biomaRt (v.2.60.1), retaining only 1:1 orthologues (*n* = 13,134). Genes with one-to-many or many-to-one relationships were excluded. For each tissue, the top 10% most variable orthologous genes were selected based on standard deviation, separately for MCCA and CCLE. The intersection of these sets was defined as the set of common top variable genes. Pearson correlation coefficients were computed between MCCA and CCLE samples using these genes. The resulting correlation matrices were clustered using the Ward.D2 method. The morphology of human pancreatic cancer cell lines was classified based on the expression of epithelial (*CDH1* associated) and mesenchymal (*VIM* associated) gene signatures described previously^78^. The upregulation/downregulation of each signature was determined by GSVA, performed on rlog-normalized gene expression data using the GSVA R package (v.1.52.3)^76^.

### Epigenetic analyses

ROADMAP RNA-seq and ChIP–seq data shown in Fig. 5c were obtained from the NIH ROADMAP epigenomics web portal (https://egg2.wustl.edu/roadmap/web_portal/chr_state_learning.html)^35^. For the pancreas (E098), lung (E096), small intestine (E109) and large intestine (E106) tissues, the consolidated and normalized bigWig files (GRCh37, hg19) were converted to the bedGraph format using the UCSC utility tool bigWigToBedGraph (v.1.04.00)^79^. For quantification of *CDKN2A* and *KRAS* mRNA expression levels, the normalized reads per kilobase million counts were used. For analysing histone modifications at the *CDKN2A* or *KRAS* locus, the signal scores were calculated as negative log_10_ of the Poisson *P* values.

For the chromHMM analysis provided by ROADMAP, the core 15-state model was selected, and the joint mnemonics bed files were obtained from the NIH ROADMAP epigenomics web portal^35^ (https://egg2.wustl.edu/roadmap/web_portal/chr_state_learning.html). This model was previously trained by integrating 5 chromatin marks (H3K4me3, H3K4me1, H3K36me3, H3K9me3, H3K27me3) for 127 reference epigenomes, whereby the chromatin state with the highest posterior probability given by the model was assigned to each genomic bin. The states were additionally stratified into eight active (TssA, TssAFlnk, TxFlnk, Tx, TxWk, EnhG, Enh, ZNF/Rpts) and seven inactive or repressed (Het, TssBiv, BivFlnk, EnhBiv, ReprPC, ReprPCWk, Quies) states. The chromHMM data were filtered to the pancreas (E098), lung (E096), small intestine (E109) and large intestine (E106) tissues and processed/harmonized using RNA-seq and ChIP–seq analyses.

For the quantitative comparison of ChIP–seq signals at *CDKN2A* and *KRAS* across healthy human tissues (Extended Data Fig. 10a,b,d,e), H3K4me3 and H3K27me3 ChIP–seq raw data of ENCODE and ROADMAP reference epigenomes were downloaded as FASTQ format from the ENCODE web portal^80,81^ (Supplementary Table 15). All datasets were processed with nf-core’s ChIP–seq pipeline (v.2.0.0)^82^, run separately for each histone mark, with narrow settings for H3K4me3 and broad settings for H3K27me3. Normalized coverage tracks (BigWig files) generated by the pipeline were then used to compute the H3K4me3 or H3K27me3 signals, defined as the sum of continuous signal values across the genomic region of interest normalized to its kb length. For H3K27me3, the ChIP–seq signal was quantified by determining histone occupancy across all exonic regions of *CDKN2A* (ENST00000304494.10 and ENST00000579755.2) or *KRAS* (ENST00000256078.10 and ENST00000311936.8). For H3K4me3, the ChIP–seq signal only at the promoter containing exons of *CDKN2A* or *KRAS* was analysed. The *CDKN2A* Ex1β promoter region was excluded from the analyses, as the H3K4me3 peak at this promoter could not be discriminated from the promoter signal of *CDKN2B-AS1* (300 bp upstream of *CDKN2A* Ex1β).

H3K4me3 and H3K27me3 ChIP–seq data of healthy mouse tissues were obtained from refs. ^83–90^ (Supplementary Table 15). The analytical workflow described for human tissues above was applied with identical parameter settings to ChIP–seq data from healthy mouse tissues. ENSMUST00000060501.4 and ENSMUST00000107131.1 transcript annotations were used for *Cdkn2a*. This approach provided a quantitative measure of H3K4me3 and H3K27me3 signals that could be compared across tissues and between species.

### Single-cell sequencing and pseudobulk analyses

Single-cell RNA-seq datasets from multiple human tissues were processed for tissue-specific pseudobulk analysis using Python (v.3.9.12) with Scanpy (v.1.9.3), Pandas (v.1.5.3) and Numpy (v.1.24.4). Datasets included epithelial cells from the pancreas, lung, rectum, stomach, bladder and uterus as well as small and large intestine. For the pancreas, lung and intestinal datasets, raw count matrices were downloaded^38–40^ and processed by filtering cells based on gene counts (min., 300; max., 2,500) and mitochondrial content (<50%), removing genes expressed in fewer than three cells, normalizing and log-transforming. Next the PCA, neighbourhood graphs (n_neighbours = 50), Leiden clustering and UMAP embeddings were computed. Only epithelial cells were retained, and clusters with fewer than 100 cells were excluded. For the bladder^47^, uterus^49^, stomach and rectum^48^, epithelial cells were selected from publicly available datasets, filtering for healthy samples and relevant tissue annotations. For cell-of-origin profiling, specific epithelial subtypes were extracted from each tissue: acinar and ductal (pancreas), type I and type II pneumocytes (lung), stem cells (small intestine, large intestine, rectum), surface foveolar and cycling cells (stomach), bladder urothelial cells (bladder) and non-ciliated epithelial cells (uterus). *CDKN2A* and *KRAS* mRNA expression levels were determined through pseudobulk analysis. For each group of cells in a specific tissue, the counts of gene-specific reads detected per cell were summed and divided by the sum of total reads detected in all cells of an individual donor. These normalized gene mRNA expression levels were independent of cluster size and sequencing depth, and therefore comparable across cell types and datasets. Donor samples contributing <50 cells of a particular group of cells were aggregated into a single sample. Donor-specific pseudobulk *CDKN2A* and *KRAS* mRNA expression values were used for statistical comparison of gene expression between cell types.

A comparable analysis was performed for mouse single-cell RNA-seq data^91^, focusing on the lung, pancreas and intestine datasets. Similarly, samples were subset based on the cell-of-origin classifications defined for the human data, and pseudobulk aggregation was applied to these samples.

For the analysis of scRNA-seq data from ref. ^29^, the dataset was subset to defined cell states of *Kras*^*G12D*^-driven pancreatic cancer evolution: *Kras*^*G12D*^-mutant acinar cells (*Cpa1*^+^tdTomato^+^*Krt19*^−^) and *Kras*^*G12D*^-mutant metaplastic ADM/PanIN cells (*Cpa1*^−^tdTomato^+^*Krt19*^+^), as well as *Kras*^*G12D*^-mutant cancer cells of an additional 15-month-old mouse. *Kras*^*WT*^ acinar cells (*Cpa1*^+^tdTomato^−^*Krt19*^−^) of the dataset were used as control. These four groups of cells were assigned based on the localization of marker gene expression in UMAPs, which were computed from a PCA (n_combs = 30), neighbourhood graphs (n_pcs = 30) and Leiden clustering. The normalized mRNA expression of *Kras* or *Cdkn2a* was determined by calculating the mean of normalized read counts in each cell state and were statistically compared across independent samples.

### In vivo transposon mutagenesis screens, QiSeq and CIS analysis

In vivo transposon mutagenesis screens in the pancreas and intestine have been performed as described previously^42,43,52^. Quantitative transposon insertion site sequencing (QiSeq) was performed as described previously^43,44^. In brief, genomic DNA was sheared to around 250 bp fragments using a Covaris M220 ultrasonicator. Fragmented DNA was end-repaired, A-tailed and ligated to a Y-shaped splinkerette adapter consisting of a double-stranded region with a hairpin loop and a single-stranded overhang. Library preparation was carried out separately for the 5′ and 3′ transposon ends. In a first PCR, junction-containing fragments were selectively amplified using transposon-specific and splinkerette-specific primers, as the adapter design prevents priming from the splinkerette during the initial cycle. A nested PCR introduced Illumina P5 and P7 adapters together with sample-specific barcodes. Libraries were quantified by qPCR with P5/P7-specific primers, pooled in equimolar amounts and sequenced on the Illumina MiSeq platform (paired-end, 65 bp). For the analysis of sequencing raw data, reads containing transposon-genome junctions were extracted, aligned to the mm10 reference genome using SSAHA2 and collapsed to unique insertion sites per tumour, with read counts quantified per site. For the pancreas samples, sequencing data were obtained from a previous study^43^ and analysed using the same computational workflow.

The subsequent common insertion site (CIS) analysis was conducted as follows. For each sample, insertion counts were normalized to library size and expressed as counts per hundred reads by dividing the counts per insertion by the total number of reads and multiplying by 100. Insertions with normalized counts of <0.02 were excluded. Insertion coordinates from all samples of the same cohort were merged into a single BED file. CISs were identified with MACS2 (v.2.2.7.1) (https://github.com/macs3-project/MACS) using four window sizes (5, 30, 60 and 100 kb) with genome size, shift and extension parameters adjusted accordingly, and with the nomodel and nolambda options enabled. Peaks were filtered at *q* < 0.05, and neighbouring peaks within 10 kb were collapsed. CISs were annotated with overlapping and flanking genomic features. Insertion sites with fewer than ten supporting reads and/or normalized counts of <0.02 were excluded from further analysis. Insertion sites within the *Cdkn2a* locus (including *Cdkn2a* and *Ncruc*/*Gm12610*) or in WNT pathway-related genes (including *Apc*, *Ctnnd1*, *Rnf4* and *Rspo2*) were then ranked according to sequencing read coverage and normalized insertion counts relative to all other insertions in the respective cancer. Finally, *Cdkn2a* locus or WNT pathway gene transposon insertions were classified as either high-ranked (top 10) or low-ranked based on their rank in each cancer sample. Assuming that the stronger the selective advantage conferred by a gene perturbation, the more pronounced the expansion of the affected cell, such ranking can serve as a direct measure for the selective advantage conferred by a transposon insertion during cancer evolution.

### Orthotopic transplantation

For mPACA transplantation experiments, 2,500–10,000 cancer cells were orthotopically grafted into the pancreas of syngeneic immunocompetent C57BL/6J or 129 WT mice. For orthotopic transplantation, mice were anaesthetized with a combination of medetomidine, midazolam and fentanyl. The left flank was carefully shaved, the eyes were protected with ointment and the abdomen was disinfected. When anaesthesia was complete, a 1.5 cm left-lateral incision caudal to the spleen was made, and the pancreas was located, and was then carefully pulled out of the abdomen to make it accessible for intraparenchymal injection. Cell suspension was administered slowly using a 27 G needle at a depth of 3–4 mm. The needle was left in this position for at least 30 s to avoid leakage of the bubble. The pancreas and spleen were carefully placed back in their anatomical position and covered with PBS to avoid organ adhesion. The peritoneum was closed with interrupted sutures (5-0 Ethilon) and the skin with wound clips. The mice were kept in a 37 °C heating chamber until they woke up.

For mCACO, transplantation experiments were performed as previously described^92^. In brief, organoids were dissociated into clusters of 5–10 cells and resuspended in a transplantation medium consisting of PBS, B27, N2, l-Glutamine (Thermo Fisher Scientific), 10% Matrigel (Corning) and 10 µM Y-27632 (Stem Cell Technologies). For each injection (2–3 per mouse), 50 dissociated organoids were prepared in a volume of 100 µl. The procedure involved anaesthetizing the mice, followed by gently rinsing the colon with PBS using a syringe and a straight oral gavage needle. Colonoscopy was performed using a rigid endoscope from Karl STORZ (1.9 mm in diameter) with linear Hopkins lens optics (ColoView System). Organoids were injected into the submucosa of the colon using a flexible fine needle (Hamilton; 33 gauge, custom length of 16 inches, custom point style of 4 at 45°). Correct submucosal injections were identified by the formation of a bubble that occluded the intestinal lumen. A scoring system was used to correlate the quality of injections with the experimental outcomes.

Note that MCCA lines might occasionally not engraft in fully immunophenotype-matched hosts due to non-immunological reasons, such as a lack of specific niche factors. These effects are especially relevant when transplanting low cell numbers (<10,000), which can, however, be rescued by increasing the number of injected cells.

### scAAV8-based somatic mutagenesis in mice

scAAV8-based somatic mutagenesis in mice was performed as described in detail previously^28^. In brief, scAAV8 particles were produced by transfecting HEK293T cells with scAAV and helper plasmid pDP8.ape. scAAV8-producing HEK293T cells were collected, resuspended and lysed through repeated freeze–thaw cycles. Free nucleic acids were digested with Benzonase nuclease (Sigma-Aldrich) and scAAV8 particle purified from the supernatant using iodixanol-based gradient ultracentrifugation (Backman Coulter). Vivaspin 20 centrifugal concentrator columns (Sigma-Aldrich) and Ringer lactate solution were used for buffer exchange of the extracted scAAV8-containing iodixanol solution. scAAV8 titres were subsequently determined by qPCR. For this, scAAV8 viral capsids were first disrupted using alkaline lysis. The sample was neutralized before qPCR-based quantification of scAAV8 viral genomes. Next, 1 × 10^12^ scAAV8 viral genomes were diluted in PBS and intraperitoneally injected into 8-week-old *Ptf1a*^*cre/+*^*;Kras*^*LSL-G12D/+*^*,Rosa26*^*CAG-LSL-Cas9/CAG-LSL-Cas9*^ mice. Pancreata were dissected from mice 8 weeks after injection of scAAV8-Tgfbr2-sgRNA or scAAV8-Rosa26-sgRNA, or from age-matched non-injected mice. Pancreas tissue was formalin-fixed and paraffin-embedded to prepare H&E stains for the quantification of acini. The acini number was determined in H&E sections by counting acini per field of view in at least five images with ×40 magnification per animal. The averaged acini count per animal was finally used to compare pancreatic remodelling across conditions.

### Amplicon-based deep sequencing

Amplicon-based deep sequencing of the mouse *Kras*^*G12D*^ mutation, human *KRAS*^*G12D*^ mutation or the mouse *Ctnnb1* exon3 hotspot mutations was performed using either 50 ng of genomic DNA (gDNA) or 1.5 µl of reverse-transcribed mRNA (cDNA). In brief, the exon2 of *Kras* and *KRAS* or exon3 of *Ctnnb1* was amplified using Kapa HiFi HotStart ReadyMix (Roche, 30 cycles) and primers with TruSeq adaptor overhangs (Supplementary Table 19). In a second PCR step (ten cycles), TruSeq index primer sequences (Illumina) were added. After each PCR step, solid-phase reversible immobilization clean-up (0.8×) was performed using an Agencourt AMPure XP kit (Beckman Coulter). The pooled library was quantified by SYBR Green qPCR (Sigma-Aldrich) and a Kapa Biosystems library quantification kit (Roche). The resulting library was sequenced on a NextSeq 550 (Illumina) system. Raw reads were mapped to the matching mouse (GRCm38) or human (GRCh38) reference genome assembly. G12D mutation-specific VAFs were calculated at the corresponding genomic position. *Ctnnb1* exon3 hotspot mutations were determined using Mutect2 from the GATK toolkit (v.4.2.0.0)^62^.

For amplicon-based deep sequencing of all mouse *Apc*-coding exons, 50 ng of gDNA was used as input for PCR-based amplification with pools of primers listed in Supplementary Table 19. PCR products were enzymatically fragmented, and libraries were prepared using the TruSeq DNA Nanokit (Illumina) according to the manufacturer’s instructions. After read mapping to GRCm38, mutation calling was conducted with Mutect2 from the GATK toolkit (v.4.2.0.0)^62^.

To determine *Kras*^*G12D*^ VAFs from laser microdissected tissue, lysates were directly prepared in the sample collection tube by adding proteinase K to a final concentration of 0.4 mg ml^−1^ and incubating overnight at 56 °C, followed by heat inactivation at 95 °C for 15 min. *Kras* exon 2 was amplified using a nested PCR strategy: an initial PCR with outer primers (KAPA HiFi HotStart, 25 cycles, annealing 59 °C) was performed, followed by purification with 0.8× AMPure XP beads (Beckman Coulter). A second PCR with inner primers and Illumina TruSeq overhangs (10 cycles, annealing 55 °C) was conducted. Finally, a third PCR was used to add sample-specific barcodes and P5/P7 adapters (10 cycles, annealing 65 °C). Cycling conditions for all PCRs were as follows: 98 °C for 20 s, annealing at the indicated temperature for 20 s, and extension at 72 °C for 45 s, with a final extension at 72 °C for 2 min. A list of all of the primers used for the three PCR steps is provided in Supplementary Table 19. Final libraries were purified (0.8× AMPure XP) and sequenced on the Illumina NextSeq 1000 system. Sequencing reads were aligned to the *Kras* reference (GRCm38), and G12D mutation-specific VAFs were calculated at the corresponding genomic position.

### cDNA synthesis and TaqMan qPCR

cDNA synthesis was synthesized from 1 mg of RNA by using SuperScript II Reverse Transcriptase (Thermo Fisher Scientific) according to standard protocols. Notably, reverse transcription was performed using random hexamers to avoid biased reverse transcription of endogenous versus lentiviral transcripts (in case oligo(dT) primers are used). TaqMan qPCR was performed using TaqMan chemistry (Thermo Fisher Scientific) and a list of the primers and probes is provided in Supplementary Table 19. Quantification of *Kras*^*LSL-G12D*^ and *KRAS*^*MUT*^ mRNA was normalized to *Kras* or *GAPDH*, respectively. TaqMan qPCR was conducted on the StepOnePlus system (Applied Biosystems).

### Flow cytometry

Mouse pancreatic ductal adenocarcinoma (PDAC) cell lines were cultured under standard conditions and treated with recombinant mouse IFNγ (BioLegend) at a final concentration of 100 ng ml^−1^ for 3 days. Untreated cells served as controls. After treatment, surface expression of MHC class I was assessed by flow cytometry. Cells were collected and transferred into 96-well V-bottom plates for staining. After centrifugation (5 min at 1,500 rpm, 4 °C), cells were washed with FACS buffer (PBS supplemented with 1% BSA and 5 mM EDTA) and incubated with an extracellular staining mix containing Fc block and either anti-MHC class I antibody (H-2Kb, AF6-88.5.5.3, eFluor 450, eBioscience, 48-5958-82) or the corresponding isotype control (mouse IgG2a κ, eFluor 450, eBioscience, 48-4724-82). Staining was carried out for 30 min at 4 °C, protected from light. After surface staining, cells were washed and incubated for 15 min at 4 °C with a viability dye (iFluor 840 maleimide, AAT Bioquest). After a final wash, cells were resuspended in FACS buffer and acquired on the CytoFlex Flow Cytometer (Beckman Coulter). Flow cytometry data were analysed using FlowJo software (v.10.10.0, FlowJo, BD). Appropriate gating strategies were applied to exclude debris, dead cells and doublets, and to quantify MHC-I surface expression.

### Doxycycline-titratable gene overexpression

The pINDUCER20 (ref. ^93^) vector system was used for doxycycline-inducible *KRAS*^*G12D*^ and GFP overexpression. HEK293FT cells were used for lentivirus production and maintained in DMEM supplemented with 10% FCS and 1% penicillin–streptomycin. In brief, the puromycin resistance was first exchanged with a hygromycin cassette and the cDNAs of oncogenic *KRAS*^*G12D*^ (CCDS 8702.1, 35G>A) or GFP were cloned in a second step into the pINDUCER20 lentiviral vector. Stbl3 bacteria (Thermo Fisher Scientific) were chemically transformed, and the plasmid DNA sequence was verified. For lentivirus production, HEK293FT cells were transfected using TransIT-LT1 (Mirus Bioscience) with standard virus packaging plasmids and the respective pINDUCER20 vectors according to the manufacturer’s recommendations. Virus-containing supernatant was pooled 48 h and 72 h after transfection, briefly centrifuged to pellet detached HEK293FT cells and filtered through 0.45-mm filters (Filtropur, Sarstedt). Lentiviral particles were stored at −80 °C until use.

For lentiviral transduction, 100,000–200,000 HPDE^94^, HBEC3KT^95^, HCEC1CT^96^, MODEK^97^ or 266-6 (ref. ^98^) cells were seeded per well of a six-well plate. Acinar WT cells are not viable in vitro; thus, the acinar carcinoma cell line 266-6 was selected as model system. The cells were transduced in the presence of 1 μg μl^−1^ polybrene (Sigma-Aldrich). Then, 2 days after transduction, cells were selected with hygromycin (Sigma-Aldrich) for at least 7 days. HPDE and HBEC3KT cells were cultured in keratinocyte-SFM medium (Thermo Fisher Scientific), supplemented with bovine pituitary extract, EGF (Thermo Fisher Scientific) and 1% penicillin–streptomycin; HCEC1CT cells in a mixture of DMEM (80%) and MEM199 (20%) supplemented with 2% FCS, EGF, insulin-transferrin-selenium, hydrocortisone (Thermo Fisher Scientific) and 1% penicillin–streptomycin; and MODEK and 266-6 cells in DMEM supplemented with 10% FCS and 1% penicillin–streptomycin. After successful transduction, the inducibility of *KRAS*^*G12D*^ expression was tested using 1:10 doxycycline dilution series ranging from 0.1 to 1,000 ng ml^−1^. HCEC1CT and MODEK showed a reduced sensitivity/response of *KRAS*^*G12D*^ induction to doxycycline treatment. To cover the dynamic induction of *KRAS*^*G12D*^ expression levels across cell lines, the doxycycline concentration range was therefore adapted for HCEC1CT and MODEK. For doxycycline-titratable induction of *KRAS*^*G12D*^ or GFP expression, cells were either seeded in 3D (HPDE, HBEC3KT, HCEC1CT, MODEK) or 2D conditions (266-6; gelatin type A coating). For 3D conditions, 150 cells were seeded per dome consisting of 50% Matrigel (Corning). After 7 days of initial growth, target gene expression was induced for 3 days by adding the indicated amounts of doxycycline (Sigma-Aldrich) to the corresponding penicillin–streptomycin-free culturing medium. At the end point, for each cell line and doxycycline concentration, at least 20 individual spheroids were imaged to assess phenotypes and RNA was isolated by pooling four domes from the identical condition to analyse transcriptomic changes. Bright-field images were used to classify spheroids into cohesive and discohesive phenotypes. Criteria included the extent of cell-to-cell adhesion, epithelial architecture of clusters, occurrence of detached single cells, and the emergence of cell membrane protrusions as compared to the growth pattern observed for spheroids of the respective untreated model. The expert biologist was blinded for phenotype grading of bright-field spheroid images. For 2D conditions, 250,000 cells were seeded per well of a six-well plate. Target gene expression was induced the next day for 3 days using the indicated doxycycline concentrations. At the end point, RNA was isolated from one well of a six-well plate per condition. TaqMan qPCR and 3′ RNA-seq library preparation were performed as described above. The 3′ RNA-seq data analysis was conducted as described below.

### Microdissection

From formalin-fixed paraffin-embedded material, one 2-µm-thick and five 10-μm-thick consecutive tissue sections were prepared and air-dried overnight. The 2 µm section was stained with H&E according to standard procedures and submitted for histopathological grading and annotation of tumour areas for microdissection. The five consecutive 10 µm sections were used for tumour microdissection. Paraffin was removed through short incubation with xylene. The specimens were briefly stained with haematoxylin and kept wet for the microdissection procedure. Individually assessed and scored samples were then microdissected under a Primovert microscope (Zeiss). gDNA was extracted using the QIAamp DNA Mini Kit (Qiagen) according to the manufacturer’s instructions, which included the use of carrier RNA to increase DNA binding during purification and a 90 °C ATL buffer incubation step to reverse formaldehyde modifications. gDNA concentrations were measured using the Qubit fluorometer (Thermo Fisher Scientific). Depending on the total available gDNA, 20–80 ng of gDNA was used as input for lcWGS, 5–20 ng for each TaqMan qPCR reaction (*Kras*^*LSL-G12D*^, *Kras*^*Copy*^) and 4–20 ng for amplicon-based deep sequencing of the *Kras*^*G12D*^ mutation. TaqMan qPCR was performed in technical quadruplicates for each target. Ratios of *Kras*^*LSL-G12D*^ to *Kras*^*Copy*^ quantifications were calculated for purity estimation of microdissected tumour tissue samples. Finally, these purity values facilitated the computational subtraction of stroma contamination from lcWGS and *Kras*^*G12D*^ amplicon sequencing data.

### Laser microdissection

Laser microdissection (LMD) was performed using the Leica LMD6 system (Leica Microsystems) to isolate defined lesions from either cryosections or paraffin-embedded sections. For cryosections, tissue was sectioned at a thickness of 7 µm and mounted onto FrameSlides with a 4.0 µm PEN membrane (Leica Microsystems). The slides were thawed at room temperature for 1 h, briefly fixed in freshly prepared 80% ethanol for 20 s and then air-dried for at least 20 min. The sections were stained with methylene blue (1:10 dilution in distilled H_2_O) for 20 s and rinsed twice in 80% ethanol, followed by an additional drying step (≥20 min) to optimize the laser-cutting efficiency. Paraffin sections (7 µm thick) were mounted onto glass slides with a 2.0 µm PEN membrane (Leica Microsystems), deparaffinized externally (Institute of Pathology, CEP) and transported in distilled H_2_O. Methylene blue staining and drying steps were performed as described for cryosections. LMD was carried out using Leica software (v.8.4). Annotated regions of interest were identified under ×10 magnification using the Leica LMD software and excised. Dissected tissue was collected directly into the lids of eight-well PCR strip tubes containing 10 µl of lysis buffer (1:1 dilution in distilled H_2_O; DirectPCR, Viagen Biotech), and the samples were immediately sealed and stored at −80 °C until further downstream analysis (see the ‘Amplicon-based deep sequencing’ section (the last paragraph is related to laser microdissected tissues)).

### ADM ex vivo assay

Pancreata of 8-week-old *Ptf1a*^*cre/+*^*;Kras*^*LSL-G12D/+*^ mice were collected, cut into pieces and digested twice in McCoy’s 5A Medium (Sigma-Aldrich), containing 0.5 mg ml^−1^ collagenase P (Sigma-Aldrich), 0.002% trypsin inhibitor from soybean (Sigma-Aldrich) and 0.1% BSA, for 10 min at 37 °C. Cells were passed through a 100 µm mesh, washed with McCoy’s 5A Medium (Sigma-Aldrich) containing 0.02% trypsin inhibitor from soybean (Sigma-Aldrich) and 0.1% BSA, and spun down for 5 min at 100*g*. The cells were then recovered in culture medium (Waymouth’s MB752/1 medium (Gibco), supplemented with 0.1% FCS (Merck), 1× insulin–transferrin–selenium (Gibco), 50 µg ml^−1^ bovine pituitary extract (Gibco), 10 mM HEPES (Gibco), 0.1% BSA, 0.01% trypsin inhibitor from soybean (Sigma-Aldrich), 2.6 mg ml^−1^ NaHCO_3_ and 30% FCS) and were incubated for 30 to 60 min at 37 °C. After recovery (which defines the 0 h timepoint), acinar cells were cultured under suspension conditions in ultra-low-attachment plates using culture medium for 24 h at 37 °C (which defines the 24 h timepoint). For the isolation of RNA, acinar cells were pelleted at the defined timepoints. The 3′ RNA-seq and analysis of transcriptome data were conducted as described in the corresponding methods sections.

### SA-βGal and Ki-67 staining

For SA-βGal staining, mouse tissues of mice were fixed in 4% paraformaldehyde (methanol free, for 1 h at 4 °C) and subsequently cryoprotected in 15% and 30% sucrose (each at least for 2 h at 4 °C) before being embedded in Tissue-Tek O.C.T. compound. The tissue blocks were frozen in a dry-ice ethanol bath and stored at −80 °C. Cryosections at a thickness of 5 μm were performed using the Leica Biosystems Cryostat (CM3050, Leica). β-Galactosidase staining on cryosections was performed using the Senescence β-Galactosidase Staining Kit (Cell Signaling) according to the manufacturer’s protocol. Nuclear counterstaining was performed using Nuclear Fast Red (Certistain, Merck).

For Ki-67 staining, formalin-fixed paraffin-embedded duodenal sections from WT and *Vil-cre;Kras*^*LSL-G12D/+*^ mice were deparaffinized and rehydrated through graded ethanol series. Antigen retrieval was performed according to the manufacturer’s protocol. The sections were incubated with anti-Ki-67 antibody (Abcam, ab16667) at the recommended dilution, followed by detection using an appropriate HRP-conjugated secondary antibody and chromogenic substrate (according to the manufacturer’s instructions). Nuclei were counterstained with haematoxylin.

### PRC2 inhibition in organoids

Intestinal and pancreatic ductal organoids were treated with a combination of A-395 hydrochloride (Sigma-Aldrich) and UNC1999 (Sigma-Aldrich), as described previously^99^. Treatment was initiated immediately after seeding into Matrigel and continued over the course of two passages (pancreas, 12 days; intestine, 10 days) to not only facilitate the block of de novo H3K27me3, but also to allow for the dilution of existing H3K27me3 through cell division. To ensure sufficient cell proliferation during the treatment period, organoids were split once between passages. The final concentrations were 4 µM A-395 and 2 µM UNC1999, added directly to the organoid culture medium. Control organoids were treated with the corresponding vehicle controls (distilled H_2_O for A-395, DMSO for UNC1999). Organoids were cultured under standard conditions and monitored throughout the treatment period. At the end of treatment, organoids were counted, and cell pellets were collected for RNA isolation and western blot analysis.

### Western blotting of histone marks

Cell pellets were lysed in RIPA buffer (Thermo Fisher Scientific) containing protease (Pierce Mini Tablets) and phosphatase inhibitor mixes I and II (SERVA), and sheared using a Covaris M220 (20 °C, 2 min, peak power 50, duty factor 20, 200 cycles per burst). The protein concentration was measured using Pierce BCA Protein Assay Kit (Thermo Fisher Scientific), and 40 µg per sample was denatured in 5× Lämmli buffer at 95 °C for 5 min. Proteins were separated on Mini-PROTEAN TGX gels (BioRad) at 65 V (stacking) and 90 V (resolving) and transferred to 0.45 µm nitrocellulose membranes (Thermo Fisher Scientific), soaked in Power Blotter 1-Step transfer buffer (Thermo Fisher Scientific) using the Power Blotter Station PB0010 (Thermo Fisher Scientific). Membranes were blocked in 5% BSA/TBS for 1 h, incubated overnight at 4 °C with anti-H3K27me3 (tri-methyl-histone H3 (Lys27) rabbit monoclonal antibody, Cell Signaling, 9733, 1:1,000) and anti-H4 (histone H4 (L64C1) mouse monoclonal antibody, Cell Signaling, 2935, 1:1,000) in 2.5% BSA/TBST, washed and incubated with anti-mouse Dylight 680 (Cell Signaling, 5470, 1:8,000) or anti-rabbit Dylight 800 (Cell Signaling, 5151P, 1:8,000) for 1 h. After the final washes, the blots were imaged on the LI-COR Odyssey Fc system and analysed using Image Studio.

### Statistics and reproducibility

For each experiment, all statistics were performed as indicated in the respective Figure and Extended Data Figure legends. Statistical testing across all classes was performed to account for multiple testing. Continuous variables were tested for normal distribution. Non-parametric tests were used for non-normally distributed data. GraphPad Prism (v.8.0.1) was used for significance calculations. Complex statistical techniques are explained in detail in the relevant Methods section.

### Materials availability

MCCA lines are available from the lead contact or the original contributor on request. All detailed information can be found at the ‘Resource availability’ and ‘Contacts’ pages on www.mcca.tum.de, or in Supplementary Table 1.

### Reporting summary

Further information on research design is available in the Nature Portfolio Reporting Summary linked to this article.

## Online content

Any methods, additional references, Nature Portfolio reporting summaries, source data, extended data, supplementary information, acknowledgements, peer review information; details of author contributions and competing interests; and statements of data and code availability are available at 10.1038/s41586-026-10187-2.

## Supplementary information

Supplementary Tables Supplementary Tables 1–19.

Reporting Summary

Supplementary Video 1 Showcase for integrative analyses of MCCA data layers through the interactive public web portal.

## Source data

Source Data Fig. 4

Source Data Extended Data Fig. 11

## Data Availability

The following reference genomes were used: GRCm38.p6 (https://www.ncbi.nlm.nih.gov/datasets/genome/GCF_000001635.26/) and GRCh38.p12 (https://www.ncbi.nlm.nih.gov/datasets/genome/GCF_000001405.38/). The following gene annotations were used: mouse gene annotations (GENCODE mouse M25; https://www.gencodegenes.org/mouse/release_M25.html), human gene annotations (GENCODE human v38; https://www.gencodegenes.org/human/release_38.html), Agilent WES mouse target regions (Agilent SureSelect XT Mouse All Exon, V1; https://earray.chem.agilent.com/suredesign/), Agilent WES human target regions (Agilent SureSelect Human All Exon V7 exon, S31285117; https://earray.chem.agilent.com/suredesign/) and Ensembl human-mouse orthologous gene names (v103; 10.1093/nar/gkae1071). The following SNP annotations were used: MGP SNP database (v5 from https://www.sanger.ac.uk/data/mouse-genomes-project/), GnomAD database (v.2.0.1 from https://gnomad.broadinstitute.org/) and dbSNP database (9606-b150 from https://www.ncbi.nlm.nih.gov/snp/). TCGA data were downloaded from dbGAP (https://www.ncbi.nlm.nih.gov/projects/gap/cgi-bin/study.cgi?study_id=phs000178.v11.p8) through GDC (https://portal.gdc.cancer.gov/). TCGA purity/ploidy reference values were obtained from the PanCanAtlas (https://gdc.cancer.gov/about-data/publications/pancanatlas). PanCuRx data were downloaded from EGA (EGAD00001003585, EGAD00001004551, EGAD00001006081 and EGAD00001006152 as part of https://ega-archive.org/studies/EGAS00001002543). CCLE data were downloaded from cBioPortal (https://www.cbioportal.org/; studies: Cancer Cell Line Encyclopedia^6,7^ and DepMap 24Q4 (10.25452/figshare.plus.27993248.v1))^50^. GDSC data were downloaded from the CellModelPassports database (https://cellmodelpassports.sanger.ac.uk/downloads). ROADMAP data were downloaded from the ROADMAP database (https://egg2.wustl.edu/roadmap/web_portal/chr_state_learning.html). ROADMAP and ENCODE ChIP–seq data were downloaded from the ENCODE database (https://www.encodeproject.org/). Mouse scRNA-seq data were downloaded from the GEO (GSE141017) and the Tabula Muris Senis consortium (10.6084/m9.figshare.8273102.v2)^100^. Mouse pancreatic cancer WES data were downloaded from the ENA (PRJEB23116). Human scRNA-seq data were downloaded from GEO (GSE84133 and GSE185224), from refs. ^39,48,49^ and the Tabula Sapiens consortium (https://figshare.com/articles/dataset/Tabula_Sapiens_v2/27921984)^101^. Mouse ChIP–seq data were downloaded from ENA (PRJNA63471, PRJNA737464, PRJNA529029, PRJNA246383, PRJNA291874, PRJNA1094907, PRJNA664361 and PRJNA892467). lcWGS, WES and 3′ RNA-seq data generated in this study are deposited under ENA accession number PRJEB78428. Processed genomic and transcriptomic data of MCCA lines are publicly available through www.mcca.tum.de. Source data are provided with this paper.

## Code availability

Bioinformatics analysis was performed using publicly available programs and parameters described in the Methods. MoCaSeq (v.0.4.54) source code is available at GitHub (https://github.com/roland-rad-lab/MoCaSeq). StrainMapper (v.1.0.0) source code generated in this study is available at GitHub (https://github.com/roland-rad-lab/StrainMapper).
