# Supplementary material for: A disease model resource reveals core principles of tissue-specific cancer evolution
Source: Nature. 2026 Feb 25;653(8113):57. doi: 10.1038/s41586-026-10187-2 (PMC13149333; doi:10.1038/s41586-026-10187-2)
Supplement: Supplementary file 1 — Supplementary Tables 1–19. [file 41586_2026_10187_MOESM1_ESM.zip › 2024-10-21642B-s1/Supplementary-Table-Legends.docx]

**Supplementary Table 1.** Annotation of MCCA lines

Worksheet 1: Annotation of MCCA lines – Overview

Worksheet 2: Annotation of MCCA lines – Triggers

Worksheet 3: Annotation of MCCA lines – Genetic alleles

Worksheet 4: Annotation of MCCA lines – Diseases

**Supplementary Table 2.** Stability of cancer cell line transcriptomes and genomes

Worksheet 1: MCCA-Panc – Transcriptome-RNA

Worksheet 2: MCCA-Panc – Genome-CNV

Worksheet 3: CCLE-vs-GDSC – Genome-CNV

**Supplementary Table 3.** Gene set enrichment analyses of pairwise transcriptome comparison for mouse hepatic cancer cell lines grown in 2D versus 3D conditions

Worksheet 1: Upregulated in 2D

Worksheet 2: Upregulated in 3D

**Supplementary Table 4.** Cross-species comparison of MCCA and CCLE transcriptomes

Worksheet 1: Lymphoid neoplasms

Worksheet 2: Myeloid neoplasms

Worksheet 3: Nervous system neoplasms

Worksheet 4: Intestinal cancers (2D)

Worksheet 5: Lung cancers (SCLC)

Worksheet 6: Lung cancers (NSCLC)

Worksheet 7: Liver cancers (HCC)

Worksheet 8: Stomach cancers

Worksheet 9: Pancreatic cancers (PDAC)

Worksheet 10: MCCA-to-CCLE – BestMatches

Worksheet 11: CCLE-to-MCCA – BestMatches

**Supplementary Table 5.** Immunophenotyping of MCCA lines

Worksheet 1: Annotation of MCCA lines - Immunophenotypes

Worksheet 2: Annotation of MCCA lines - MHC haplotypes

**Supplementary Table 6.** Effective pTMB in distinct transplantation scenarios

**Supplementary Table 7.** *Kras^G12D^* allelic status in MCCA samples of pancreas, lung and intestine

**Supplementary Table 8.** *KRAS^MUT^* allelic status in hCCLE samples of pancreas, lung and intestine (Barretina et al., 2012)

**Supplementary Table 9.** *KRAS^MUT^* allelic status in TCGA and ICGC samples of pancreas, lung and intestine

**Supplementary Table 10.** Gene set enrichment analyses of the top250 genes driving transcriptomic separation along the indicated principal components upon doxycycline-inducible overexpression of *KRAS^G12D^* or GFP in human non-transformed pancreatic, lung, and intestinal cells

Worksheet 1: HPDE-PC1pos

Worksheet 2: HPDE-PC1neg

Worksheet 3: HPDE-PC2pos

Worksheet 4: HPDE-narrow-PC1pos

Worksheet 5: HPDE-narrow-PC1neg

Worksheet 6: HBEC3KT-PC1pos

Worksheet 7: HBEC3KT-PC1neg

Worksheet 8: HBEC3KT-PC2neg

Worksheet 9: HCEC1CT-PC1pos

Worksheet 10: HCEC1CT-PC1neg

**Supplementary Table 11.** Gene set enrichment analyses of top250 PC1 genes induced by doxycycline-titratable overexpression of *KRAS^G12D^* in murine, non-transformed cells of the intestine

Worksheet 1: MODEK-PC1pos

Worksheet 2: MODEK-PC1neg

**Supplementary Table 12.** Stage-specific acquisition of *Apc* and *Ctnnb1* mutations during *Kras^G12D^*-driven serrated intestinal cancer evolution

**Supplementary Table 13.** *Cdkn2a* status in MCCA samples of pancreas, lung and intestine

**Supplementary Table 14.** *CDKN2A* status in TCGA and ICGC samples of pancreas, lung and intestine

**Supplementary Table 15.** H3K4me3 and H3K27me3 ChIP-seq signals in healthy tissues

Worksheet 1: Human

Worksheet 2: Mouse

**Supplementary Table 16.** Gene set enrichment analyses of genes upregulated in TCGA-LUAD with *CDKN2A^HOM^* versus *CDKN2A^HET/WT^*

**Supplementary Table 17.** *CDKN2A* status in CCLE and MCCA samples of the lung

Worksheet 1: *CDKN2A* status in hCCLE samples of the lung (Barretina et al., 2012)

Worksheet 2: *Cdkn2a* status in MCCA lines of the lung

**Supplementary Table 18.** *KRAS^MUT^* allelic status in TCGA and ICGC pancreatic, lung and intestinal cancers depending on *CDKN2A* and *TP53* status

**Supplementary Table 19.** Oligonucleotides used in this study
