# Supplementary material for: A disease model resource reveals core principles of tissue-specific cancer evolution
Source: Nature. 2026 Feb 25;653(8113):57. doi: 10.1038/s41586-026-10187-2 (PMC13149333; doi:10.1038/s41586-026-10187-2)
Supplement: Supplementary file 2 — Reporting Summary [file 41586_2026_10187_MOESM2_ESM.pdf]

nature portfolio

Corresponding author(s): Roland Rad

Last updated by author(s): Dec 23, 2025

Reporting Summary

Nature Portfolio wishes to improve the reproducibility of the work that we publish. This form provides structure for consistency and transparency in reporting. For further information on Nature Portfolio policies, see our Editorial Policies and the Editorial Policy Checklist.

Statistics

For all statistical analyses, confirm that the following items are present in the figure legend, table legend, main text, or Methods section.

n/a	Confirmed	
☐	☒	The exact sample size (*n*) for each experimental group/condition, given as a discrete number and unit of measurement
☐	☒	A statement on whether measurements were taken from distinct samples or whether the same sample was measured repeatedly
☐	☒	The statistical test(s) used AND whether they are one- or two-sided<br>*Only common tests should be described solely by name; describe more complex techniques in the Methods section.*
☐	☒	A description of all covariates tested
☐	☒	A description of any assumptions or corrections, such as tests of normality and adjustment for multiple comparisons
☐	☒	A full description of the statistical parameters including central tendency (e.g. means) or other basic estimates (e.g. regression coefficient) AND variation (e.g. standard deviation) or associated estimates of uncertainty (e.g. confidence intervals)
☐	☒	For null hypothesis testing, the test statistic (e.g. *F*, *t*, *r*) with confidence intervals, effect sizes, degrees of freedom and *P* value noted<br>*Give P values as exact values whenever suitable.*
☒	☐	For Bayesian analysis, information on the choice of priors and Markov chain Monte Carlo settings
☒	☐	For hierarchical and complex designs, identification of the appropriate level for tests and full reporting of outcomes
☐	☒	Estimates of effect sizes (e.g. Cohen's *d*, Pearson's *r*), indicating how they were calculated

Our web collection on statistics for biologists contains articles on many of the points above.

Software and code

Policy information about availability of computer code

Data collection	MiSeq, NextSeq 550, NextSeq100 or NovaSeq 6000 (Illumina), StepOnePlus™ Real-Time PCR System (Applied Biosystems), CytoFlex Flow Cytometer (Beckman Coulter), LI-COR Odyssey® Fc, GDC Data Transfer Tool (v2.3.0_Ubuntu_x64-py3.8-ubuntu-20.04)
Data analysis	Statistical analysis were conducted with R v4.4.1 and Graphpad Prism v8.01<br>Heatmap visualization: pheatmap (v1.0.12) and ComplexHeatmap (v2.16.0)<br>RNAseq mapping: Drop-seq pipeline (v1.0)<br>WES/WGS/lcWGS analysis: bcl2fastq (v2.20.0.422), MoCaSeq (v0.4.54), Trimmomatic (v0.39), BWA-MEM (v0.7.17), Picard tools (v2.20.0), GATK (v4.2.0.0), SNPeff (v4.3), CNVKit (v0.9.9), sambamba (v0.7.0), samblaster (v0.1.26), ABSOLUTE (v1.0.6), MSIsensor (v0.5)<br>Single-cell RNA processing and analysis: Python (3.9.12), Scanpy (v1.9.3), Pandas (1.5.3), Numpy (1.24.4)<br>RNAseq Analysis: R (v4.2.1), DESeq2 (v1.46.0), limma (v3.5.4), GSVA (v1.52.3), enrichR (v1.62.0), ggplot2 (v3.4.2), data.table (v1.14.8)<br>Additional analysis: bigWigToBedGraph (v1.04.00), samtools (v1.17), cBioPortal (v3.7.1), biomaRt (v2.60.1), nf-core's ChIP-seq pipeline (v2.0.0), MACS2 (v2.2.7.1)<br>Histology: Leica LAS X (v3.7.5.24914), Leica Aperio ImageScope (v12.4.3.5008)<br>MoCaSeq (v0.4.54) source code is available on https://github.com/roland-rad-lab/MoCaSeq. StrainMapper (v1.0.0) source code generated in this study is available on https://github.com/roland-rad-lab/StrainMapper.

For manuscripts utilizing custom algorithms or software that are central to the research but not yet described in published literature, software must be made available to editors and reviewers. We strongly encourage code deposition in a community repository (e.g. GitHub). See the Nature Portfolio guidelines for submitting code & software for further information.

Data

Policy information about availability of data

All manuscripts must include a data availability statement. This statement should provide the following information, where applicable:

- Accession codes, unique identifiers, or web links for publicly available datasets
- A description of any restrictions on data availability
- For clinical datasets or third party data, please ensure that the statement adheres to our policy

The following reference genomes were used: GRCm38.p6 (https://www.ncbi.nlm.nih.gov/datasets/genome/GCF_000001635.26/) and GRCh38.p12 (https://www.ncbi.nlm.nih.gov/datasets/genome/GCF_000001405.38/). The following gene annotations were used: mouse gene annotations (GENCODE mouse M25, https://www.gencodegenes.org/mouse/release_M25.html), human gene annotations (GENCODE human v38, https://www.gencodegenes.org/human/release_38.html), Agilent WES mouse target regions (Agilent SureSelect XT Mouse All Exon, V1, https://earray.chem.agilent.com/suredesign/), Agilent WES human target regions (Agilent SureSelect Human All Exon V7 exon, S31285117, https://earray.chem.agilent.com/suredesign/) and Ensembl human-mouse orthologous gene names (v103, https://doi.org/10.1093/nar/gkae1071). The following SNP annotations were used: MGP SNP database (v5 from https://www.sanger.ac.uk/data/mouse-genomes-project/), GnomAD database (v2.0.1 from https://gnomad.broadinstitute.org/) and dbSNP database (9606-b150 from https://www.ncbi.nlm.nih.gov/snp/). TCGA data were downloaded from dbGAP (https://www.ncbi.nlm.nih.gov/projects/gap/cgi-bin/study.cgi?study_id=phs000178.v11.p8) through GDC (https://portal.gdc.cancer.gov/). TCGA purity/ploidy reference values were obtained from the PanCanAtlas (https://gdc.cancer.gov/about-data/publications/pancanatlas). PanCuRx data were downloaded from EGA (EGAD00001003585, EGAD00001004551, EGAD00001006081 and EGAD00001006152 as part of https://ega-archive.org/studies/EGAS00001002543). CCLE data were downloaded from cBioPortal (https://www.cbioportal.org/, studies: Cancer Cell Line Encyclopedia (Novartis/Broad, Nature 2012) and Cancer Cell Line Encyclopedia (Broad, 2019)) and DepMap 24Q4 (https://doi.org/10.25452/figshare.plus.27993248.v1). GDSC data were downloaded from CellModelPassports database (https://cellmodelpassports.sanger.ac.uk/downloads). ROADMAP data were downloaded from ROADMAP database (https://egg2.wustl.edu/roadmap/web_portal/chr_state_learning.html). ROADMAP and ENCODE ChIP-seq data were downloaded from ENCODE database (https://www.encodeproject.org/). Mouse scRNA-seq data were downloaded from GEO (GSE141017) and the Tabula Muris Senis consortium (https://doi.org/10.6084/m9.figshare.8273102.v2). Mouse pancreatic cancer WES data was downloaded from ENA (PRJEB23116). Human scRNA-seq data were downloaded from GEO (GSE84133 and GSE185224), from Oliver et al. (https://www.nature.com/articles/s41586-024-07571-1), Garcia-Alonso et al. (https://www.nature.com/articles/s41588-021-00972-2), Sikkema et al. (https://www.nature.com/articles/s41591-023-02327-2) and the Tabula Sapiens consortium (https://figshare.com/articles/dataset/Tabula_Sapiens_v2/27921984). Mouse ChIP-seq data were downloaded from ENA (PRJNA63471, PRJNA737464, PRJNA529029, PRJNA246383, PRJNA291874, PRJNA1094907, PRJNA664361 and PRJNA892467). Low-coverage whole genome sequencing, whole exome sequencing and 3-prime RNA sequencing data generated in this study are deposited under ENA accession number PRJEB78428. Processed genomic and transcriptomic data of MCCA lines is publicly available through www.mcca.tum.de. Source data are provided with this paper.

Research involving human participants, their data, or biological material

Policy information about studies with human participants or human data. See also policy information about sex, gender (identity/presentation), and sexual orientation and race, ethnicity and racism.

Reporting on sex and gender	n/a
Reporting on race, ethnicity, or other socially relevant groupings	n/a
Population characteristics	n/a
Recruitment	n/a
Ethics oversight	n/a

Note that full information on the approval of the study protocol must also be provided in the manuscript.

Field-specific reporting

Please select the one below that is the best fit for your research. If you are not sure, read the appropriate sections before making your selection.

☒ Life sciences ☐ Behavioural & social sciences ☐ Ecological, evolutionary & environmental sciences

For a reference copy of the document with all sections, see nature.com/documents/nr-reporting-summary-flat.pdf

Life sciences study design

All studies must disclose on these points even when the disclosure is negative.

Sample size	No statistical methods were used to predetermine sample size. Sample size for in vitro experiments or animal studies was selected based on our previous experience and publications. The sample size of external datasets was determined based on the availability of openly accessible samples within each study.
Data exclusions	No data exclusion was performed.

Replication	All experimental findings were reliably reproduced as indicated in the figure legends.
Randomization	For in vitro experiments, potential sources of bias were controlled through standardized processing, replication and randomized sample allocation. Animal studies were conducted in age- and sex-matched mice that were randomly assigned to experimental groups. For studies involving autochthonous mouse models, mice were not randomized and selected according to genotype.
Blinding	Blinding was not implemented for in vitro experiments and animal studies, as data generation and and analyses were based on standardized protocols, instrumentation, and automated pipelines that are not subjective. For manual scoring of spheroid phenotypes in GFP/KRAS-overexpression models, blinding was performed to exclude potential observer-related bias.

Reporting for specific materials, systems and methods

We require information from authors about some types of materials, experimental systems and methods used in many studies. Here, indicate whether each material, system or method listed is relevant to your study. If you are not sure if a list item applies to your research, read the appropriate section before selecting a response.

Materials & experimental systems

n/a	Involved in the study	
☐	☒	Antibodies
☐	☒	Eukaryotic cell lines
☒	☐	Palaeontology and archaeology
☐	☒	Animals and other organisms
☒	☐	Clinical data
☒	☐	Dual use research of concern
☒	☐	Plants

Methods

n/a	Involved in the study	
☒	☐	ChIP-seq
☐	☒	Flow cytometry
☒	☐	MRI-based neuroimaging

Antibodies

Antibodies used	H-2Kb, clone AF6-88.5.5.3, eFluor™ 450, eBioscience™, #48-5958-82<br>Mouse IgG2a κ, eFluor™ 450, eBioscience™, #48-4724-8<br>Tri-Methyl-Histone H3 [Lys27] Rabbit mAb, Cell Signaling, #9733<br>Histone H4 [L64C1] Mouse mAb, Cell Signaling, #2935<br>anti-rabbit Dylight 800 (Cell Signaling, #5151P)<br>anti-mouse Dylight 680 (Cell Signaling, #5470)
Validation	n/a

Eukaryotic cell lines

Policy information about cell lines and Sex and Gender in Research

Cell line source(s)	The source and sex for each model of the Mouse Cancer Cell line Atlas is provided in Supplementary Table 1. Additional cell lines as follows: 266-6 (ATCC; CRL-2151), MODEK (non-commercial, provided by D. Kaiserlian, Institute Pasteur, Paris, France), HEK293T (ATCC; CRL-11268), HEK293FT (ThermoFisher Scientific; R70007), HPDE (Merck; SCC442), HBEC3KT (Evercyte; CkHT-004-0230) and HCEC1CT (Evercyte; CkHT-039-0229).
Authentication	Cell line authentication of mouse cancer cell lines was performed by re-genotyping of engineered alleles from genomic DNA.
Mycoplasma contamination	All cell lines were routinely tested for mycoplasma infection.
Commonly misidentified lines<br>(See ICLAC register)	No commonly misidentified cell line was used.

Animals and other research organisms

Policy information about studies involving animals; ARRIVE guidelines recommended for reporting animal research, and Sex and Gender in Research

Laboratory animals	For transplantation studies, 8-12 weeks old C57BL/6J or C57BL6/6J;129-F1 hybrid mice were used. For scAAV-sgRNA experiments, 8-12 weeks old mice on a C57BL6/6J;129-mixed genetic background were used. Autochthonous mouse models were aged and monitored for up two years for tumor development (specified in Supplementary Table 1).
Wild animals	No wild animals were used in this study.
Reporting on sex	Female and male mice were randomly submitted to respective tumor cohorts and all results thus apply to both sexes.

Field-collected samples | Mice were housed under specific-pathogen-free conditions.

Ethics oversight | All animal studies were conducted in compliance with European guidelines for the care and use of laboratory animals and were approved by the Institutional Animal Care and Use Committees (IACUC) of Technische Universität München, Regierung von Oberbayern and the UK Home Office.

Note that full information on the approval of the study protocol must also be provided in the manuscript.

Plants

Seed stocks | *Report on the source of all seed stocks or other plant material used. If applicable, state the seed stock centre and catalogue number. If plant specimens were collected from the field, describe the collection location, date and sampling procedures.*

Novel plant genotypes | *Describe the methods by which all novel plant genotypes were produced. This includes those generated by transgenic approaches, gene editing, chemical/radiation-based mutagenesis and hybridization. For transgenic lines, describe the transformation method, the number of independent lines analyzed and the generation upon which experiments were performed. For gene-edited lines, describe the editor used, the endogenous sequence targeted for editing, the targeting guide RNA sequence (if applicable) and how the editor was applied.*

Authentication | *Describe any authentication procedures for each seed stock used or novel genotype generated. Describe any experiments used to assess the effect of a mutation and, where applicable, how potential secondary effects (e.g. second site T-DNA insertions, mosiacism, off-target gene editing) were examined.*

Flow Cytometry

Plots

Confirm that:

☒ The axis labels state the marker and fluorochrome used (e.g. CD4-FITC).

☒ The axis scales are clearly visible. Include numbers along axes only for bottom left plot of group (a 'group' is an analysis of identical markers).

☒ All plots are contour plots with outliers or pseudocolor plots.

☒ A numerical value for number of cells or percentage (with statistics) is provided.

Methodology

Sample preparation | Murine pancreatic ductal adenocarcinoma (PDAC) cell lines were washed, blocked with Fc receptor blocking reagent, stained with anti-MHC class I antibody (H-2Kb, clone AF6-88.5.5.3, eFluor™ 450) or isotype control (Mouse IgG2a κ, eFluor™ 450) for 30 min at 4 °C, washed, and incubated with viability dye (iFluor® 840 maleimide, 15 min, 4 °C). After a final wash, cells were resuspended in FACS buffer (PBS + 1% BSA + 5 mM EDTA) and acquired.

Instrument | CytoFlex Flow Cytometer (Beckman Coulter).

Software | FlowJo version 10.10.0 (FlowJo LLC, BD).

Cell population abundance | Viable single cells were gated and analyzed. The frequency of MHC class I–positive cells and the relative surface expression (geometric mean fluorescence intensity) were quantified. Across all samples, between 45-85% of recorded events were identified as single cells, of which ~35-90% represented the defined cell population of interest. Within these, the majority were viable, with live-cell frequencies typically exceeding 85-95% across experimental conditions.

Gating strategy | Sequential gating was applied to exclude debris, doublets, and dead cells. From singlets, viable cells were selected, and MHC class I expression was quantified. Representative gating is shown in Extended Data Fig. 5c (anti-MHC class I antibody staining vs. isotype control).

☒ Tick this box to confirm that a figure exemplifying the gating strategy is provided in the Supplementary Information.
