# Supplementary figures and images for: A disease model resource reveals core principles of tissue-specific cancer evolution (part 2 of 3)
Source: Nature. 2026 Feb 25;653(8113):57. doi: 10.1038/s41586-026-10187-2 (PMC13149333; doi:10.1038/s41586-026-10187-2)

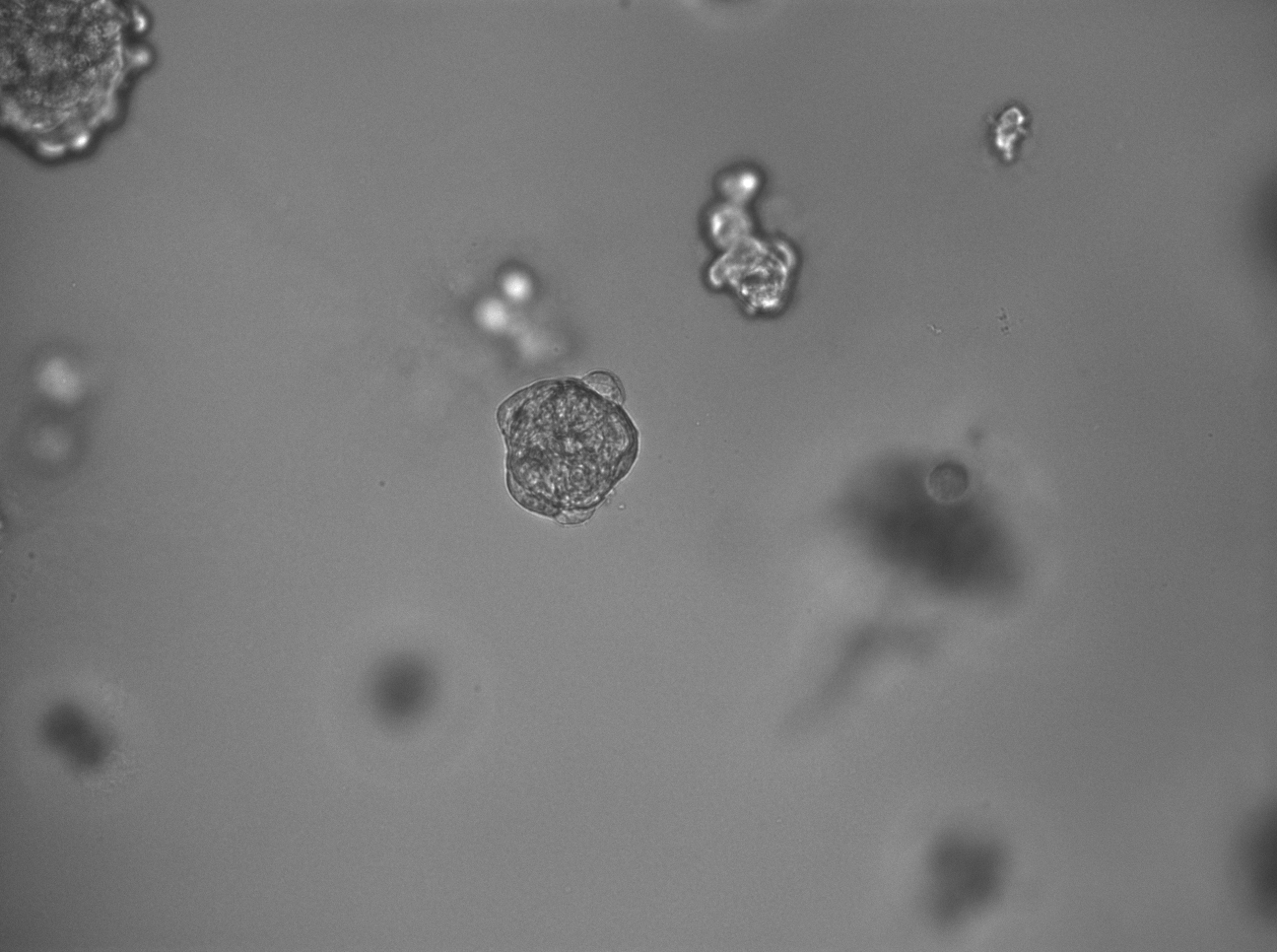

Supplement: Supplementary file 4 — Source Data Fig. 4 [file 41586_2026_10187_MOESM4_ESM.zip › HCEC1CT/HCEC1CT-KRAS_D10_Dox-00125_C03e_20x_ch00.jpg]

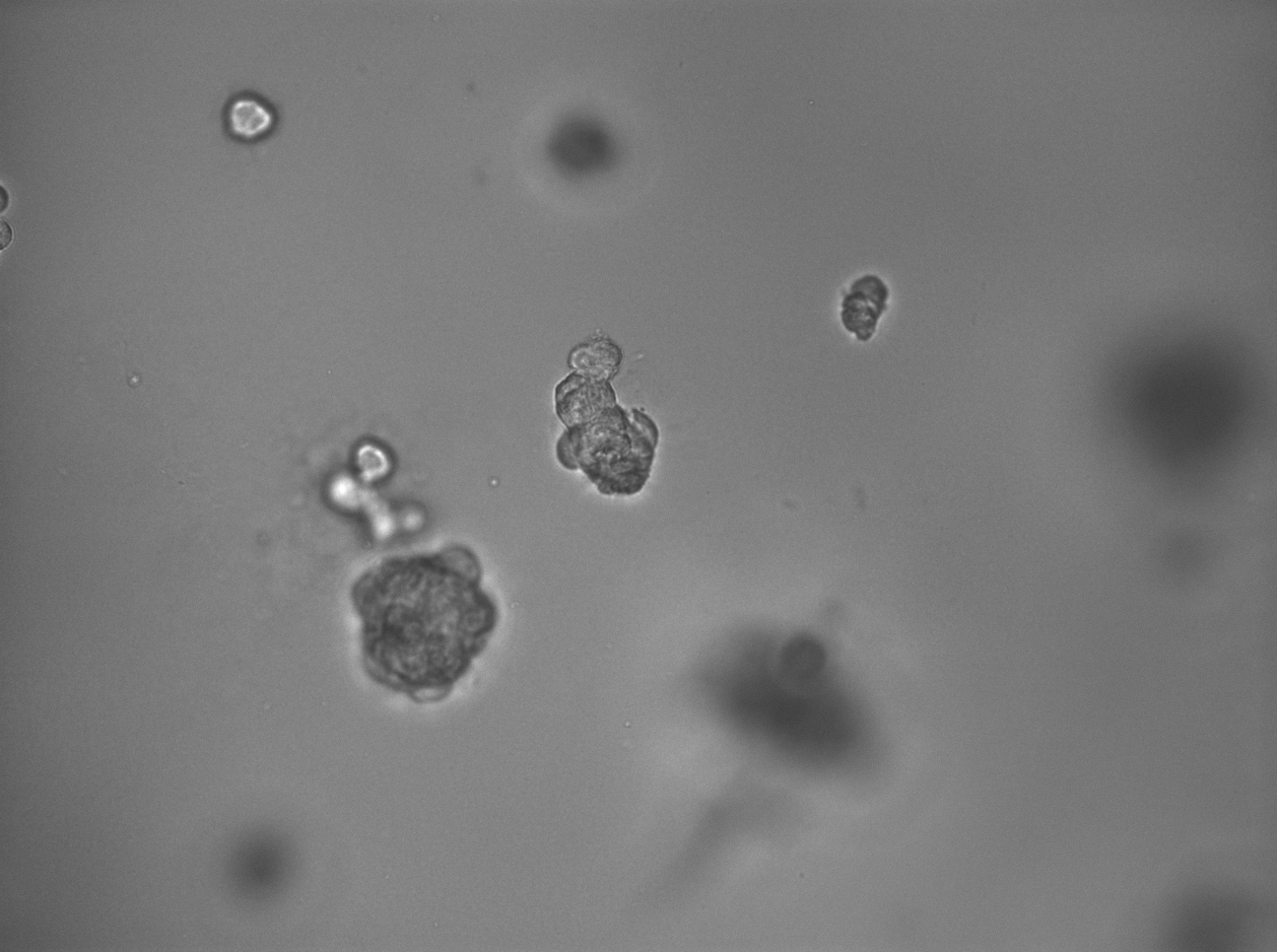

Supplement: Supplementary file 4 — Source Data Fig. 4 [file 41586_2026_10187_MOESM4_ESM.zip › HCEC1CT/HCEC1CT-KRAS_D10_Dox-00125_C03f_20x_ch00.jpg]

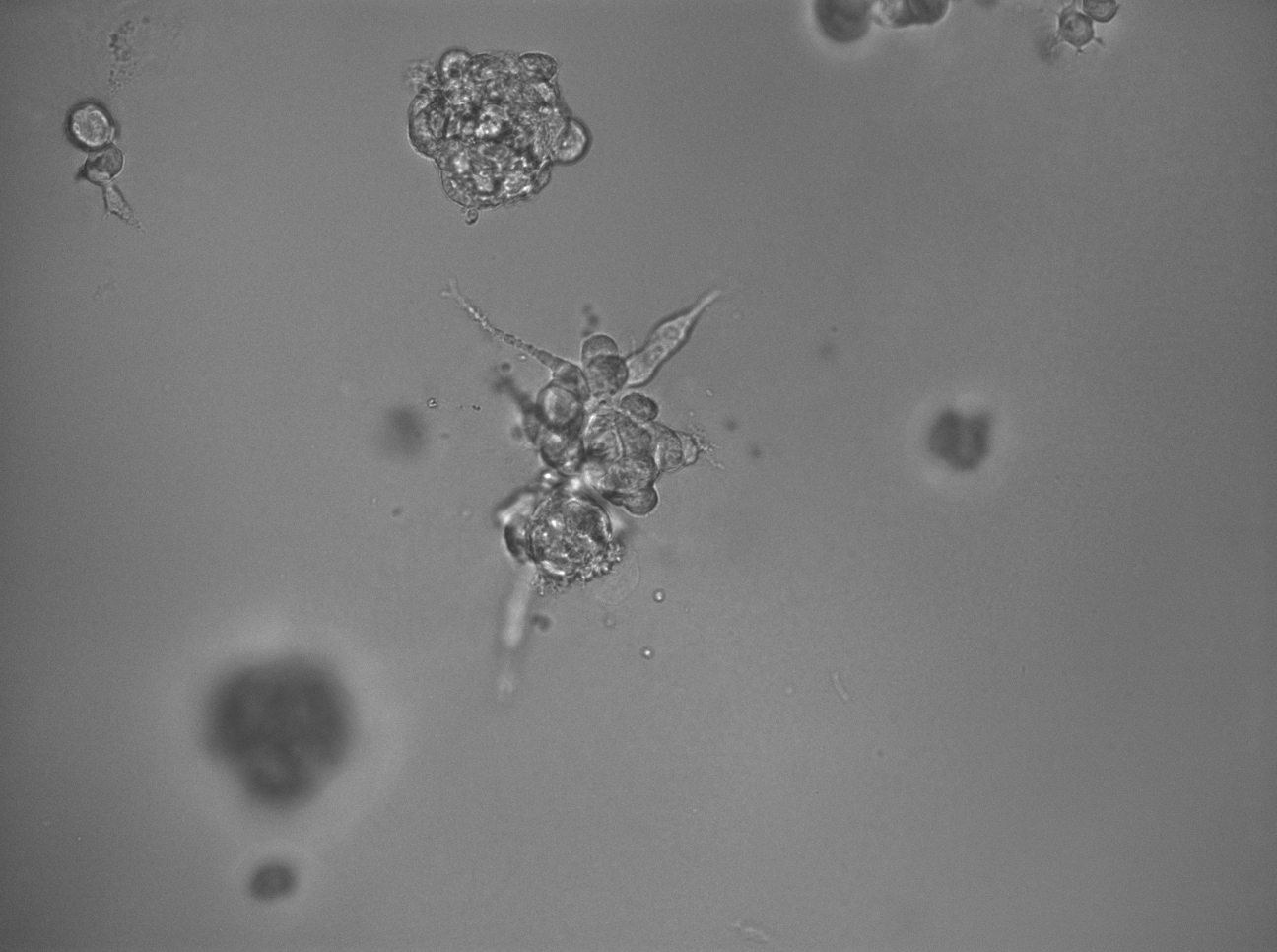

Supplement: Supplementary file 4 — Source Data Fig. 4 [file 41586_2026_10187_MOESM4_ESM.zip › HCEC1CT/HCEC1CT-KRAS_D10_Dox-00125_C03g_20x_ch00.jpg]

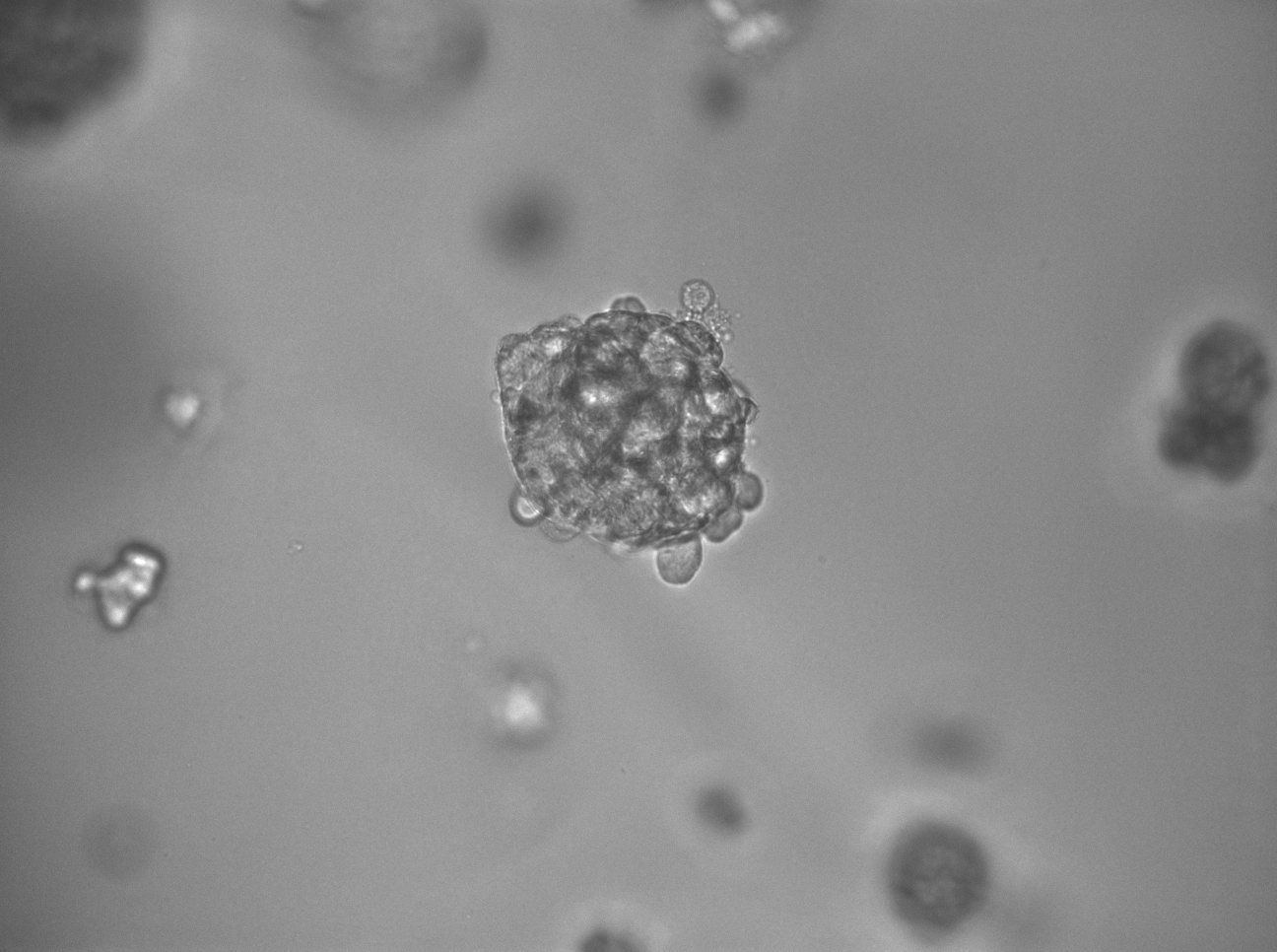

Supplement: Supplementary file 4 — Source Data Fig. 4 [file 41586_2026_10187_MOESM4_ESM.zip › HCEC1CT/HCEC1CT-KRAS_D10_Dox-00250_D01a_20x_ch00.jpg]

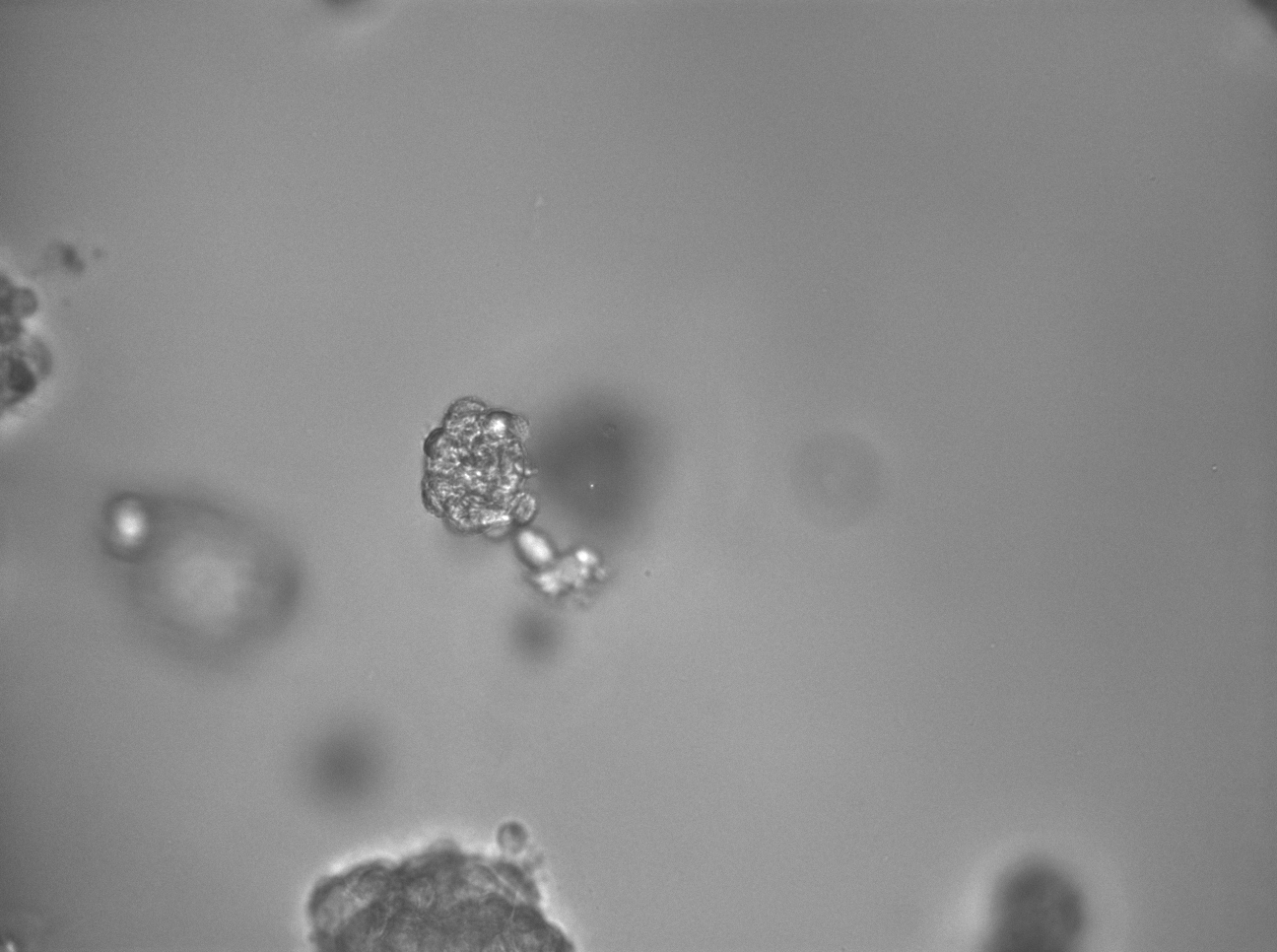

Supplement: Supplementary file 4 — Source Data Fig. 4 [file 41586_2026_10187_MOESM4_ESM.zip › HCEC1CT/HCEC1CT-KRAS_D10_Dox-00250_D01b_20x_ch00.jpg]

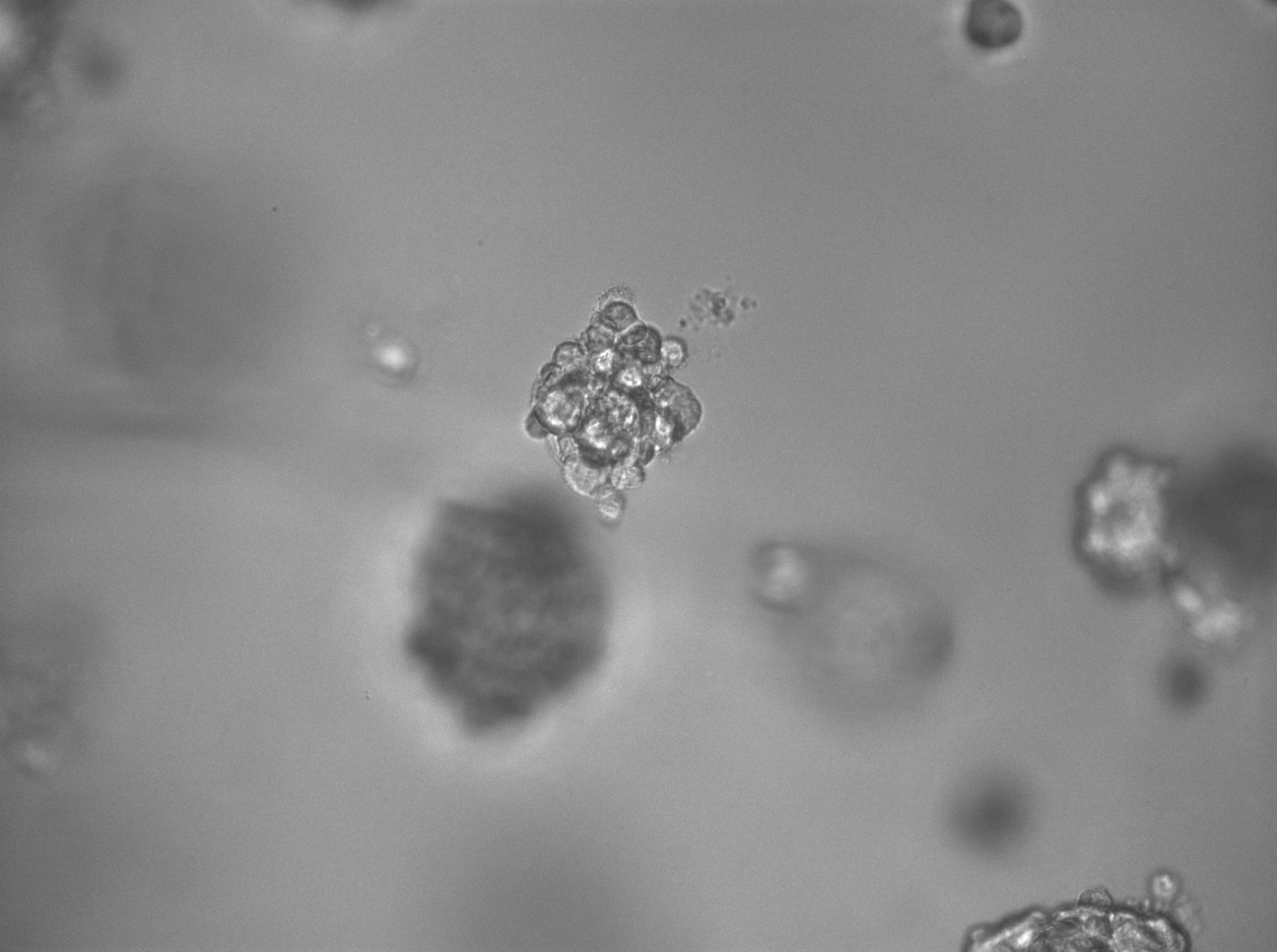

Supplement: Supplementary file 4 — Source Data Fig. 4 [file 41586_2026_10187_MOESM4_ESM.zip › HCEC1CT/HCEC1CT-KRAS_D10_Dox-00250_D01c_20x_ch00.jpg]

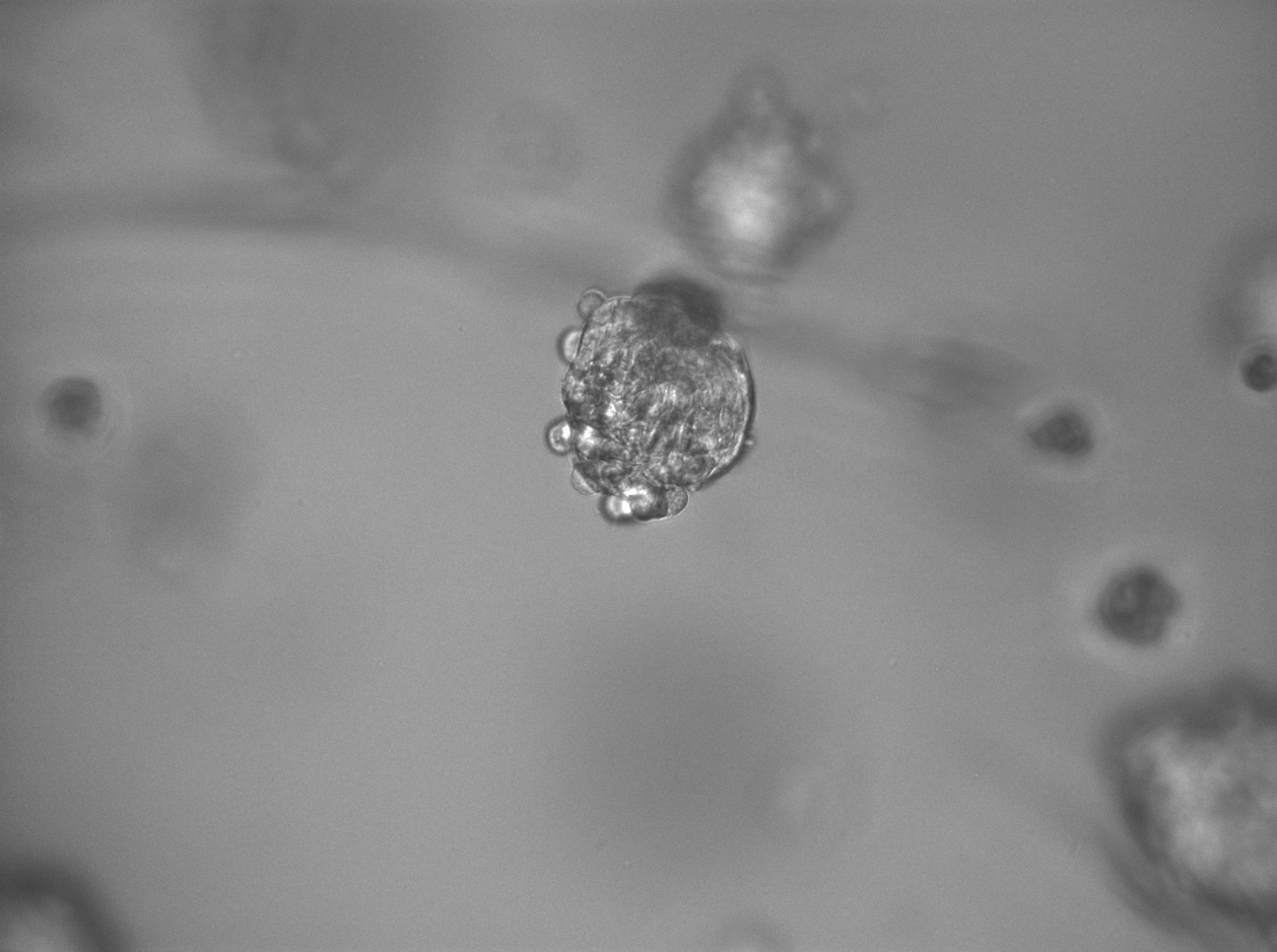

Supplement: Supplementary file 4 — Source Data Fig. 4 [file 41586_2026_10187_MOESM4_ESM.zip › HCEC1CT/HCEC1CT-KRAS_D10_Dox-00250_D01d_20x_ch00.jpg]

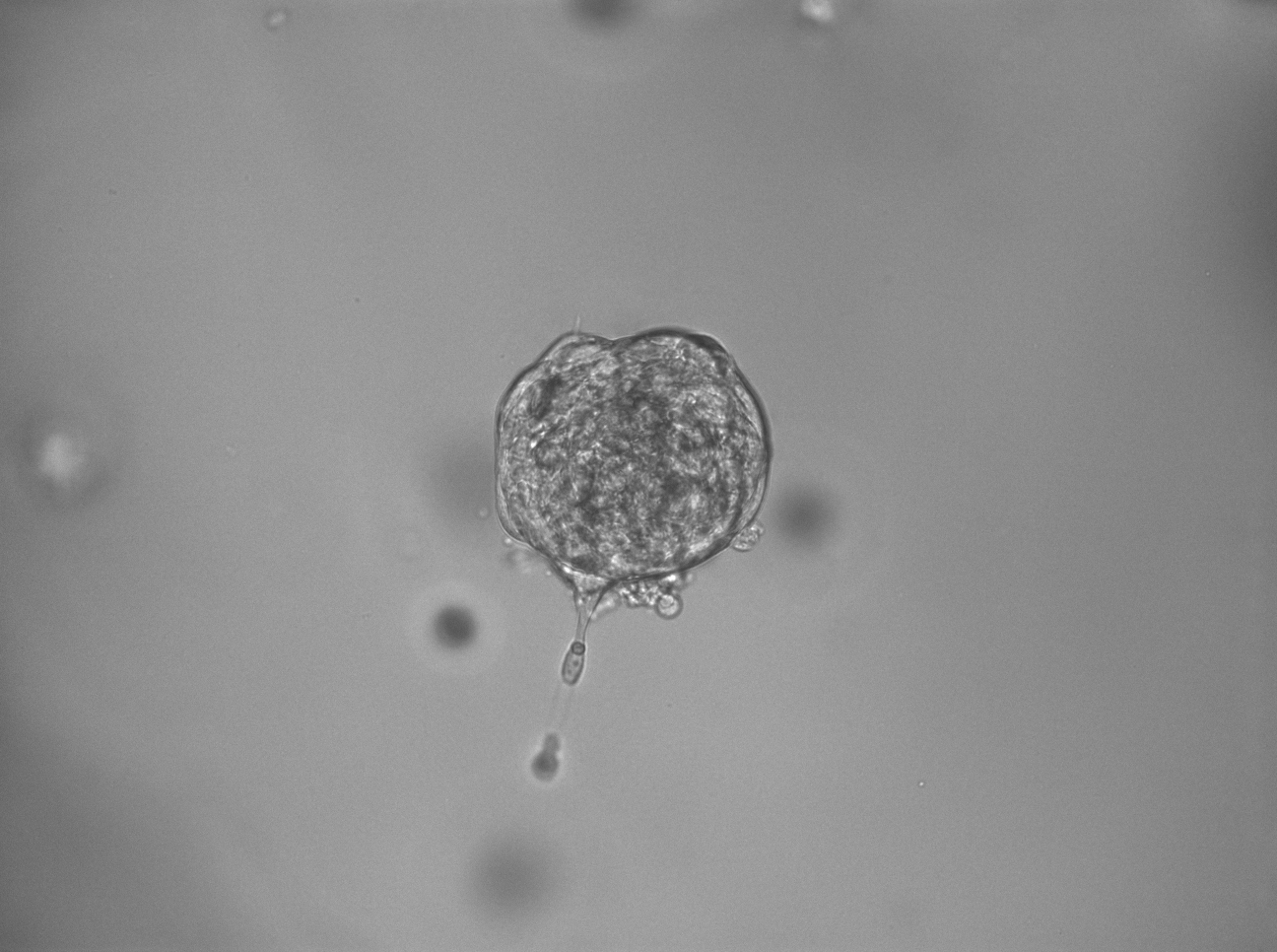

Supplement: Supplementary file 4 — Source Data Fig. 4 [file 41586_2026_10187_MOESM4_ESM.zip › HCEC1CT/HCEC1CT-KRAS_D10_Dox-00250_D01e_20x_ch00.jpg]

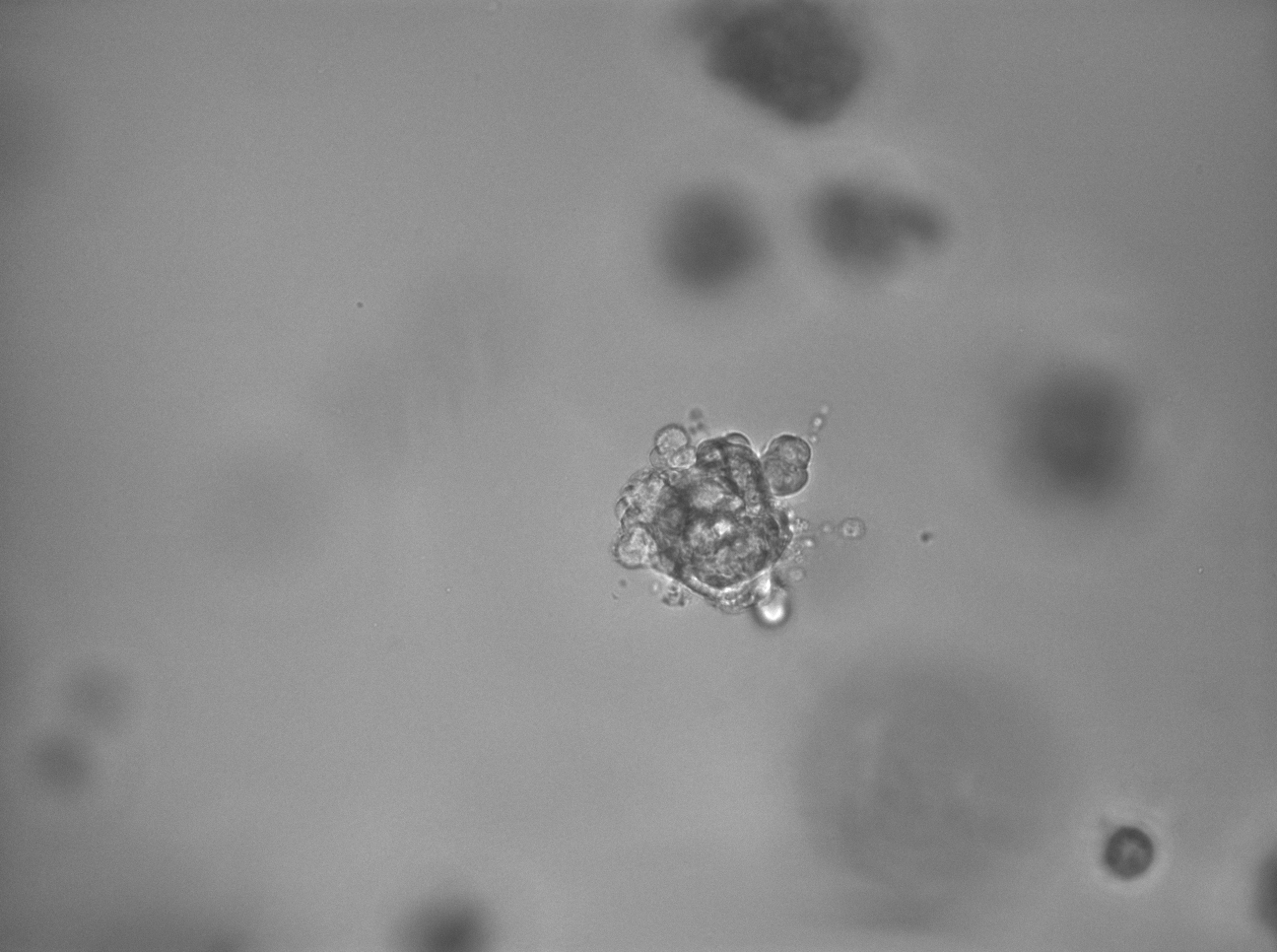

Supplement: Supplementary file 4 — Source Data Fig. 4 [file 41586_2026_10187_MOESM4_ESM.zip › HCEC1CT/HCEC1CT-KRAS_D10_Dox-00250_D01f_20x_ch00.jpg]

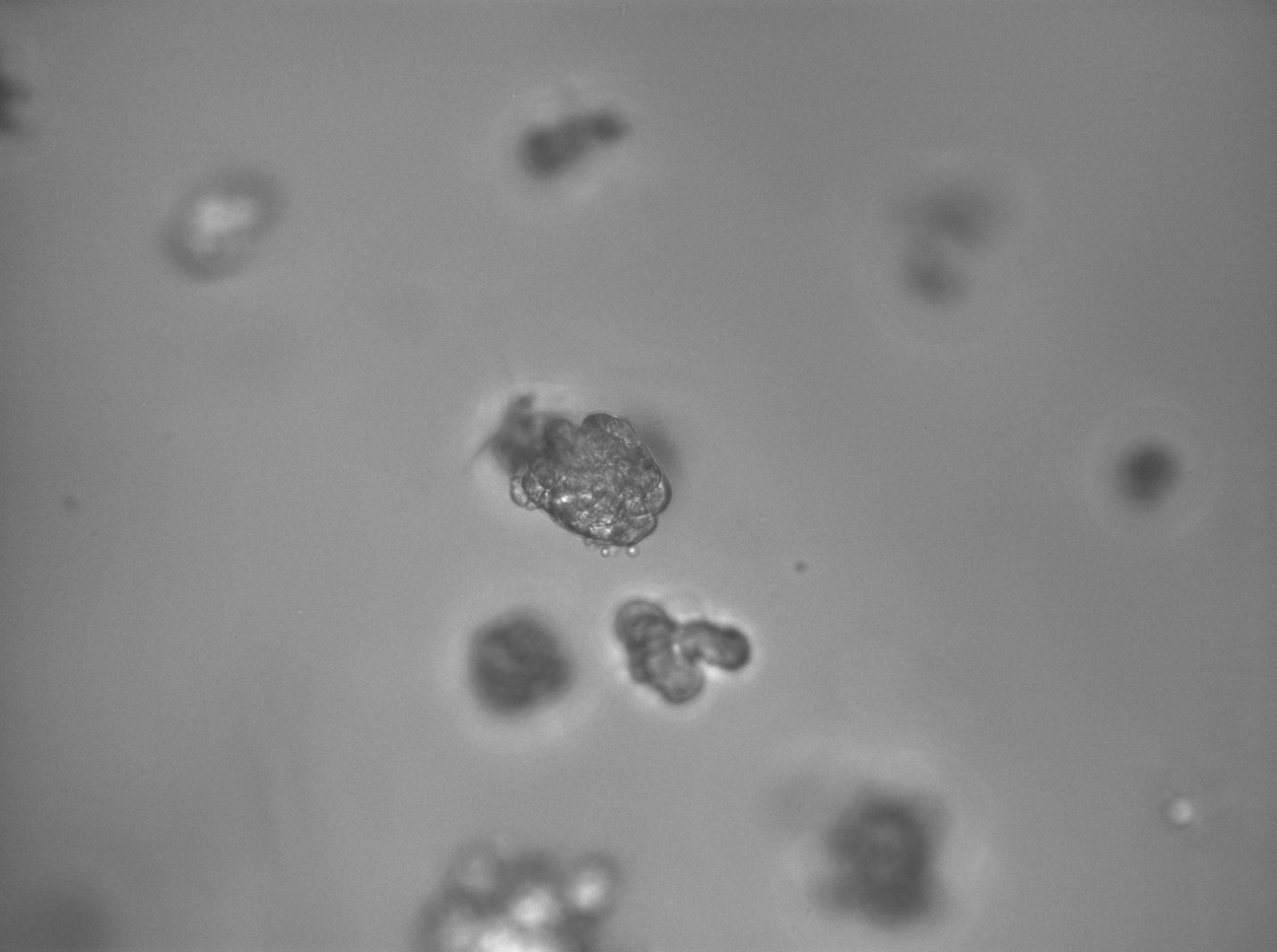

Supplement: Supplementary file 4 — Source Data Fig. 4 [file 41586_2026_10187_MOESM4_ESM.zip › HCEC1CT/HCEC1CT-KRAS_D10_Dox-00250_D01g_20x_ch00.jpg]

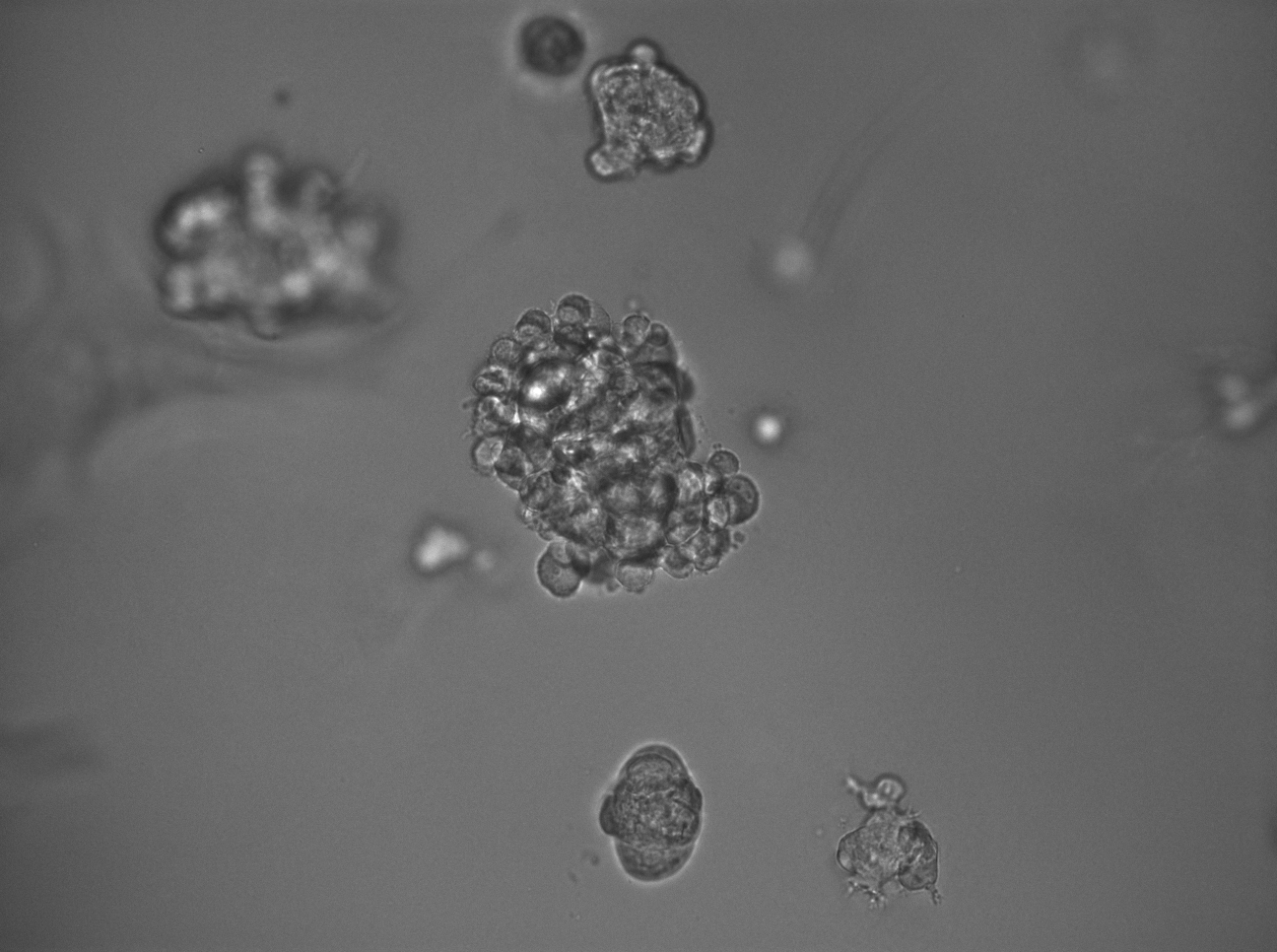

Supplement: Supplementary file 4 — Source Data Fig. 4 [file 41586_2026_10187_MOESM4_ESM.zip › HCEC1CT/HCEC1CT-KRAS_D10_Dox-00250_D02a_20x_ch00.jpg]

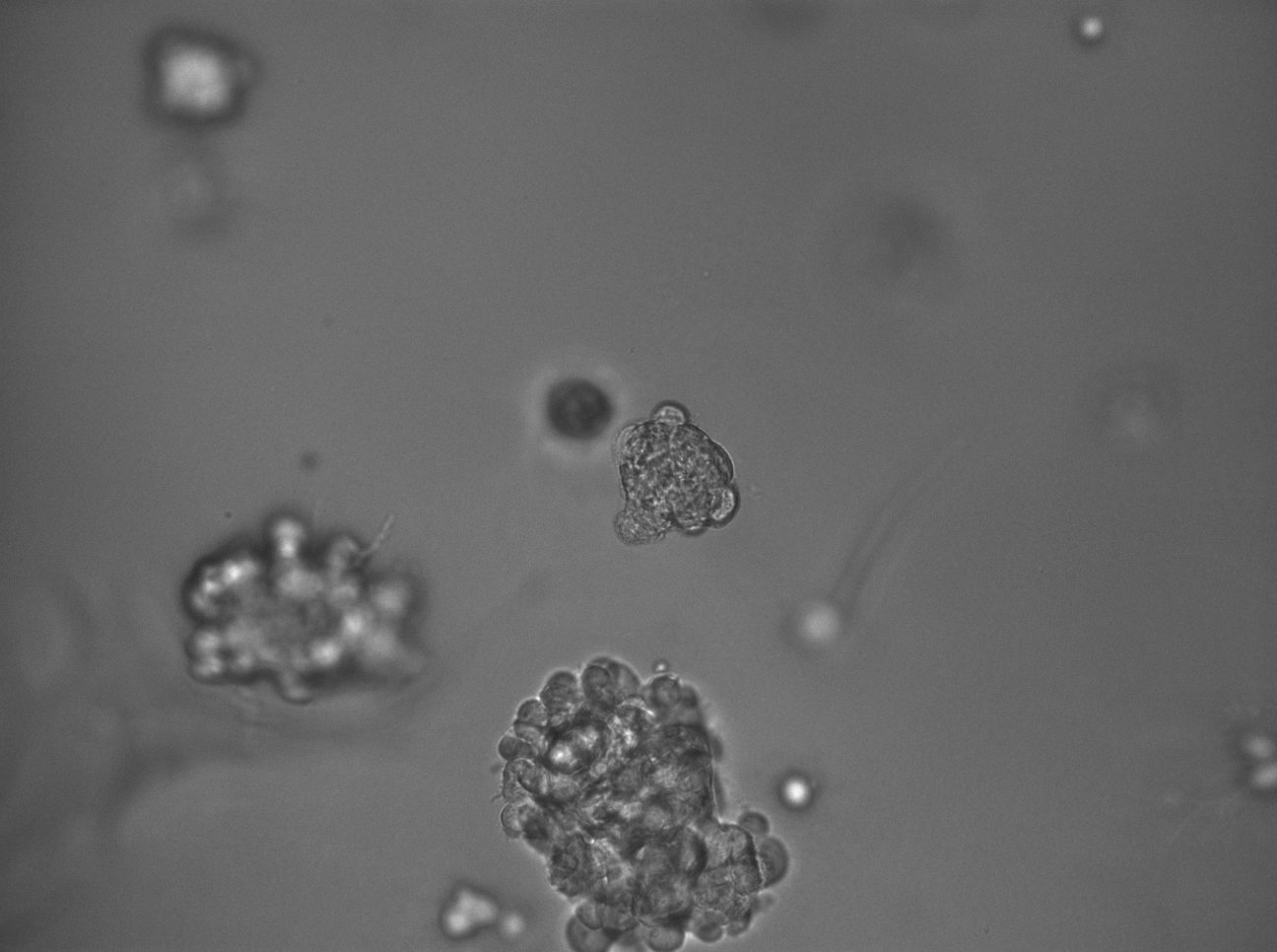

Supplement: Supplementary file 4 — Source Data Fig. 4 [file 41586_2026_10187_MOESM4_ESM.zip › HCEC1CT/HCEC1CT-KRAS_D10_Dox-00250_D02b_20x_ch00.jpg]

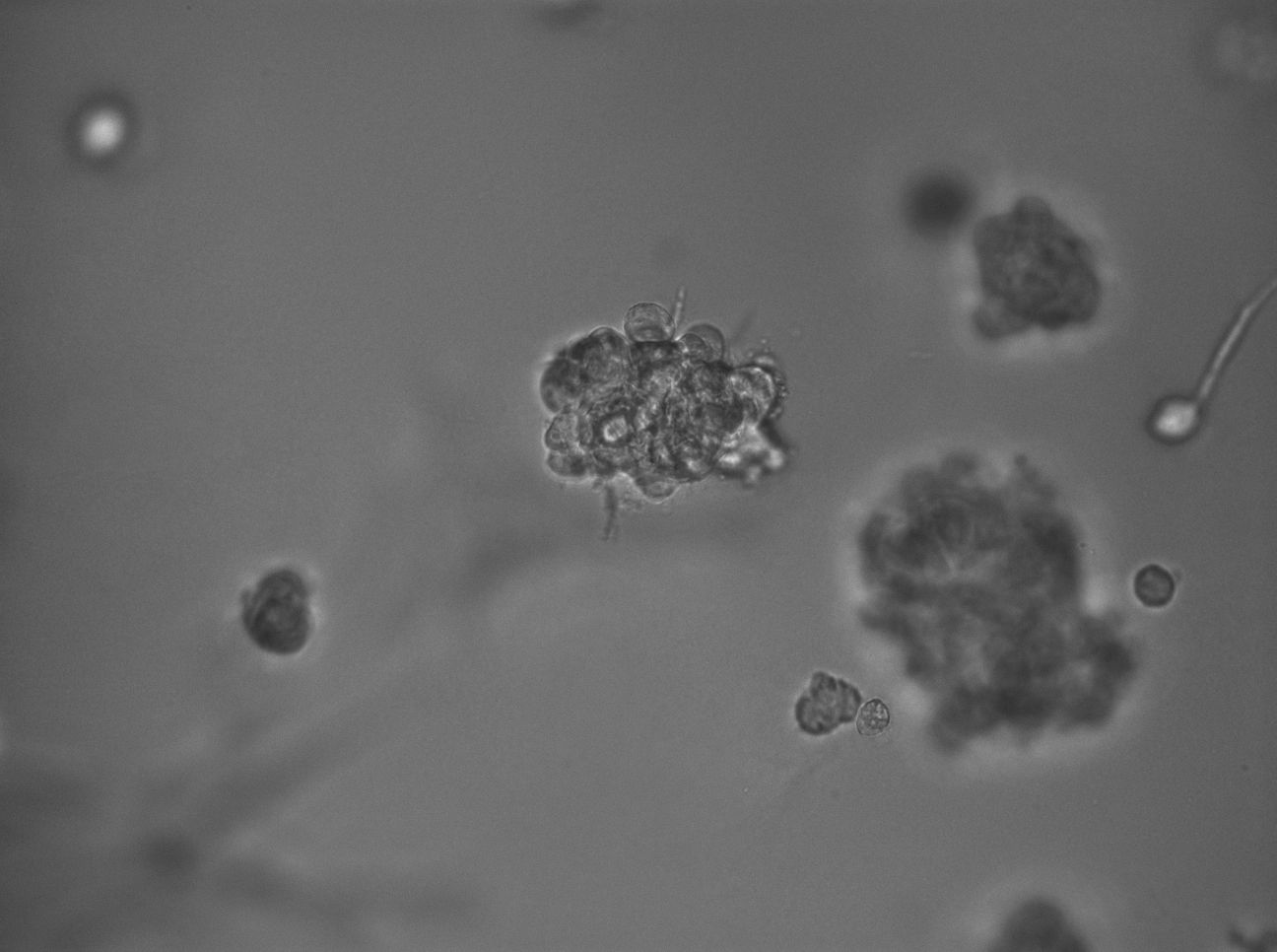

Supplement: Supplementary file 4 — Source Data Fig. 4 [file 41586_2026_10187_MOESM4_ESM.zip › HCEC1CT/HCEC1CT-KRAS_D10_Dox-00250_D02c_20x_ch00.jpg]

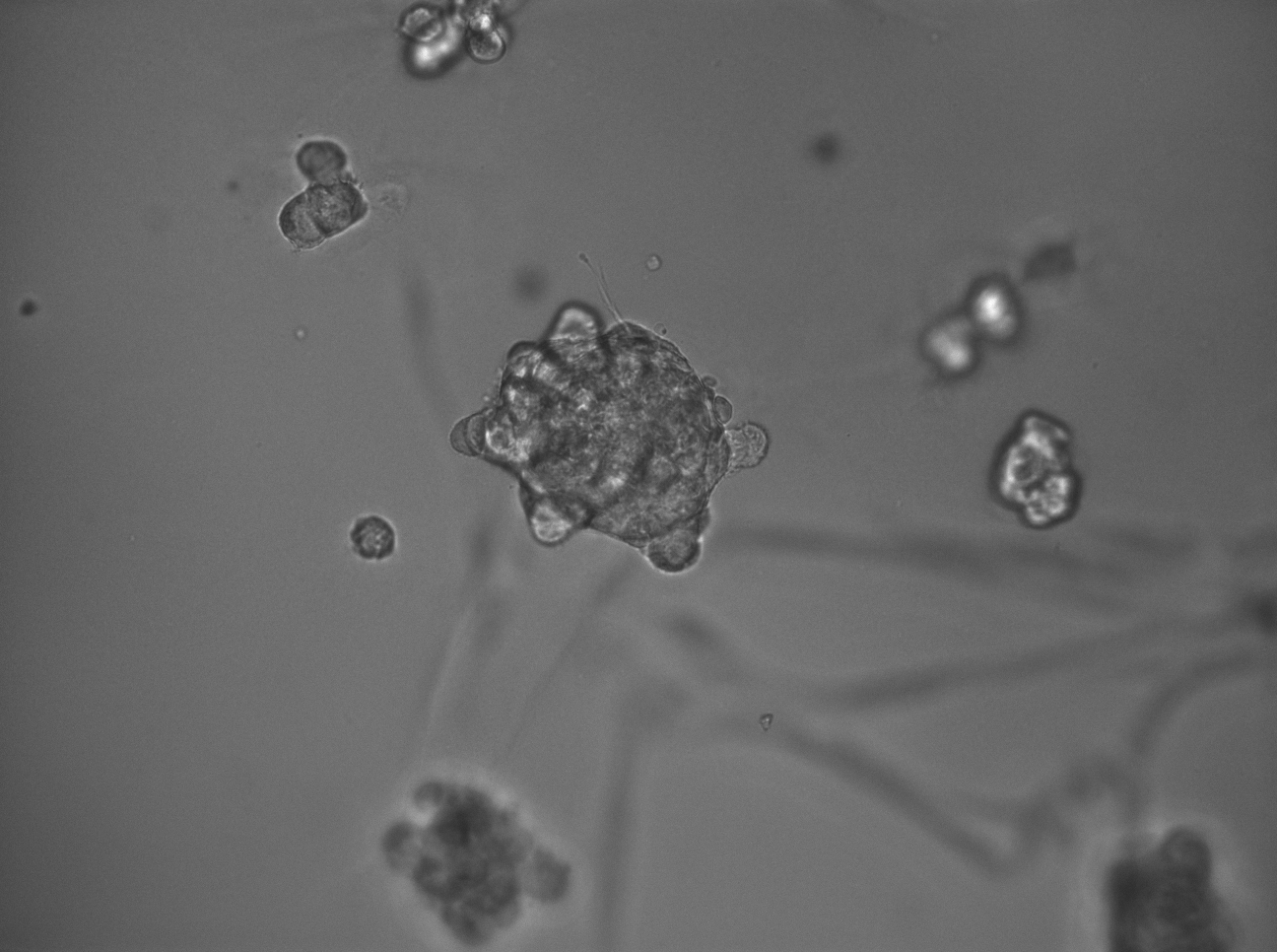

Supplement: Supplementary file 4 — Source Data Fig. 4 [file 41586_2026_10187_MOESM4_ESM.zip › HCEC1CT/HCEC1CT-KRAS_D10_Dox-00250_D02d_20x_ch00.jpg]

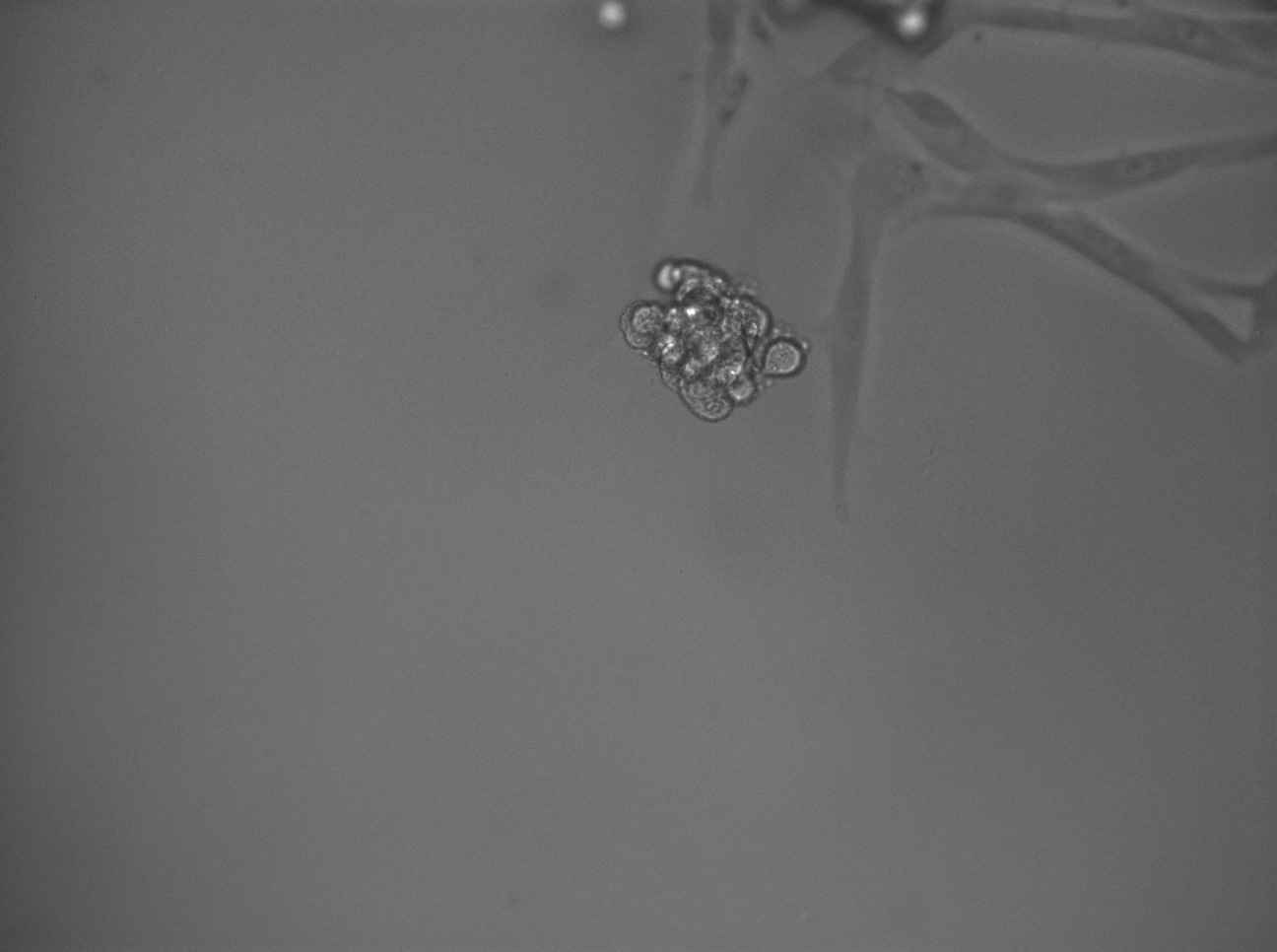

Supplement: Supplementary file 4 — Source Data Fig. 4 [file 41586_2026_10187_MOESM4_ESM.zip › HCEC1CT/HCEC1CT-KRAS_D10_Dox-00250_D02e_20x_ch00.jpg]

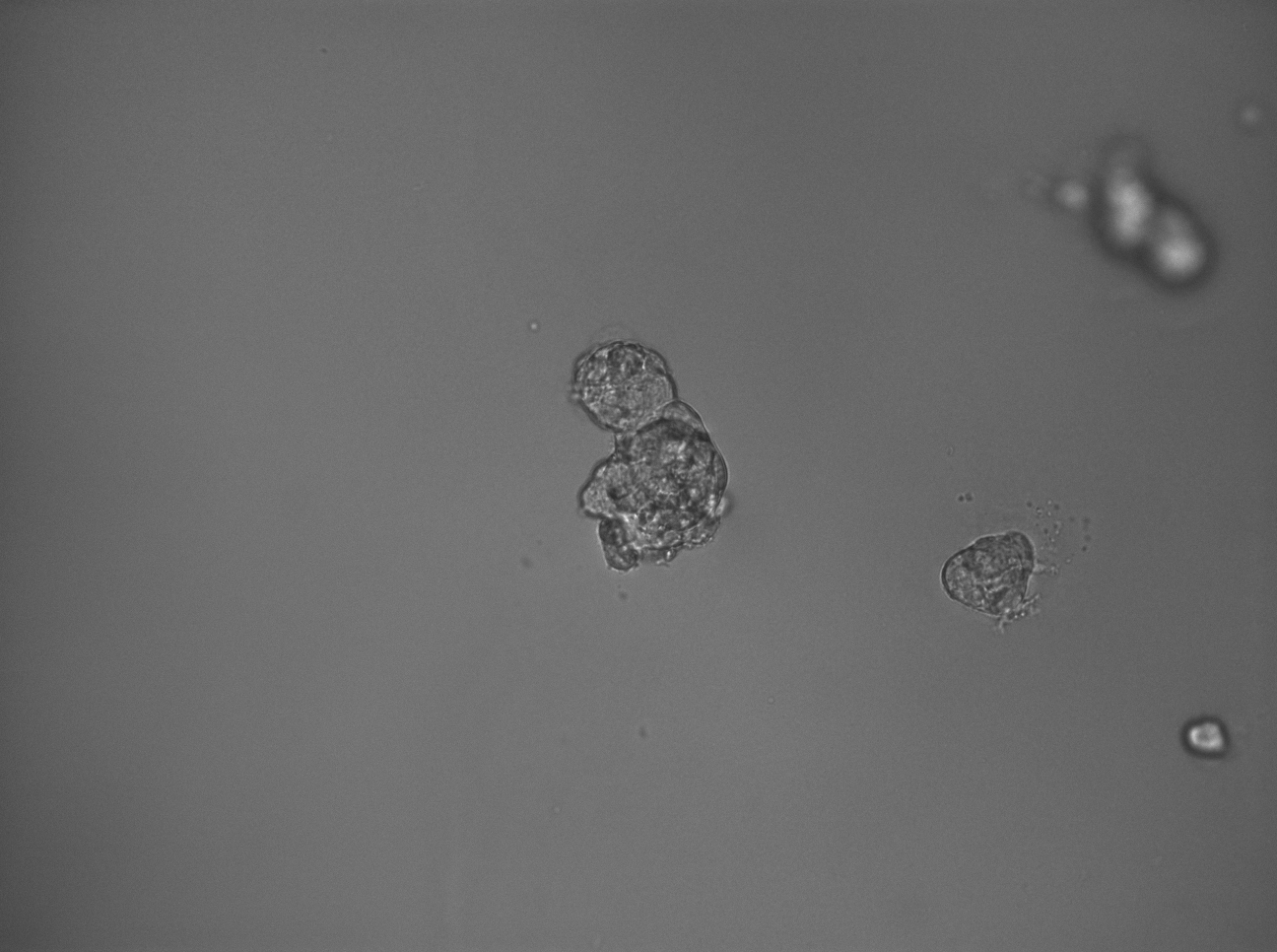

Supplement: Supplementary file 4 — Source Data Fig. 4 [file 41586_2026_10187_MOESM4_ESM.zip › HCEC1CT/HCEC1CT-KRAS_D10_Dox-00250_D02f_20x_ch00.jpg]

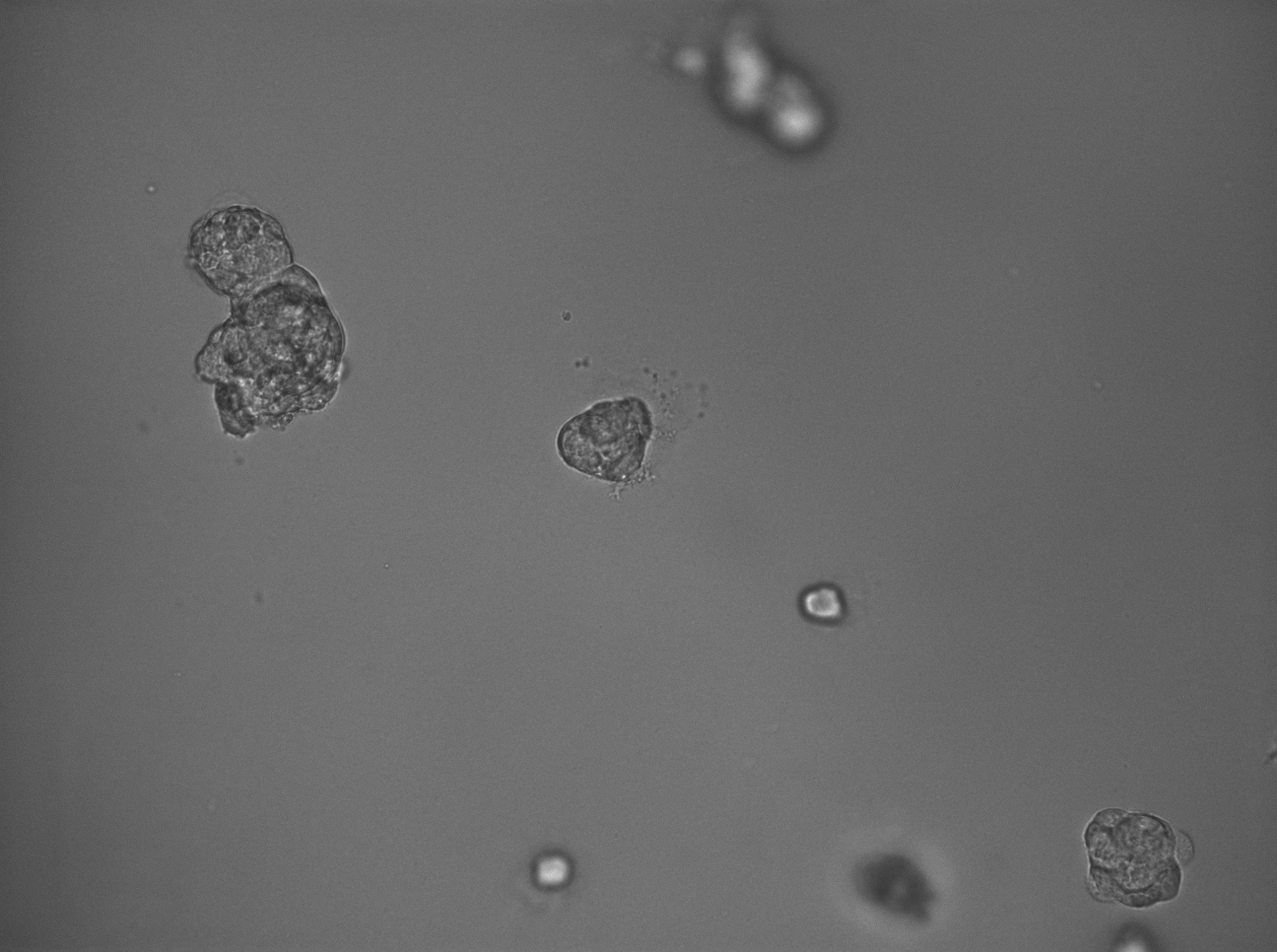

Supplement: Supplementary file 4 — Source Data Fig. 4 [file 41586_2026_10187_MOESM4_ESM.zip › HCEC1CT/HCEC1CT-KRAS_D10_Dox-00250_D02g_20x_ch00.jpg]

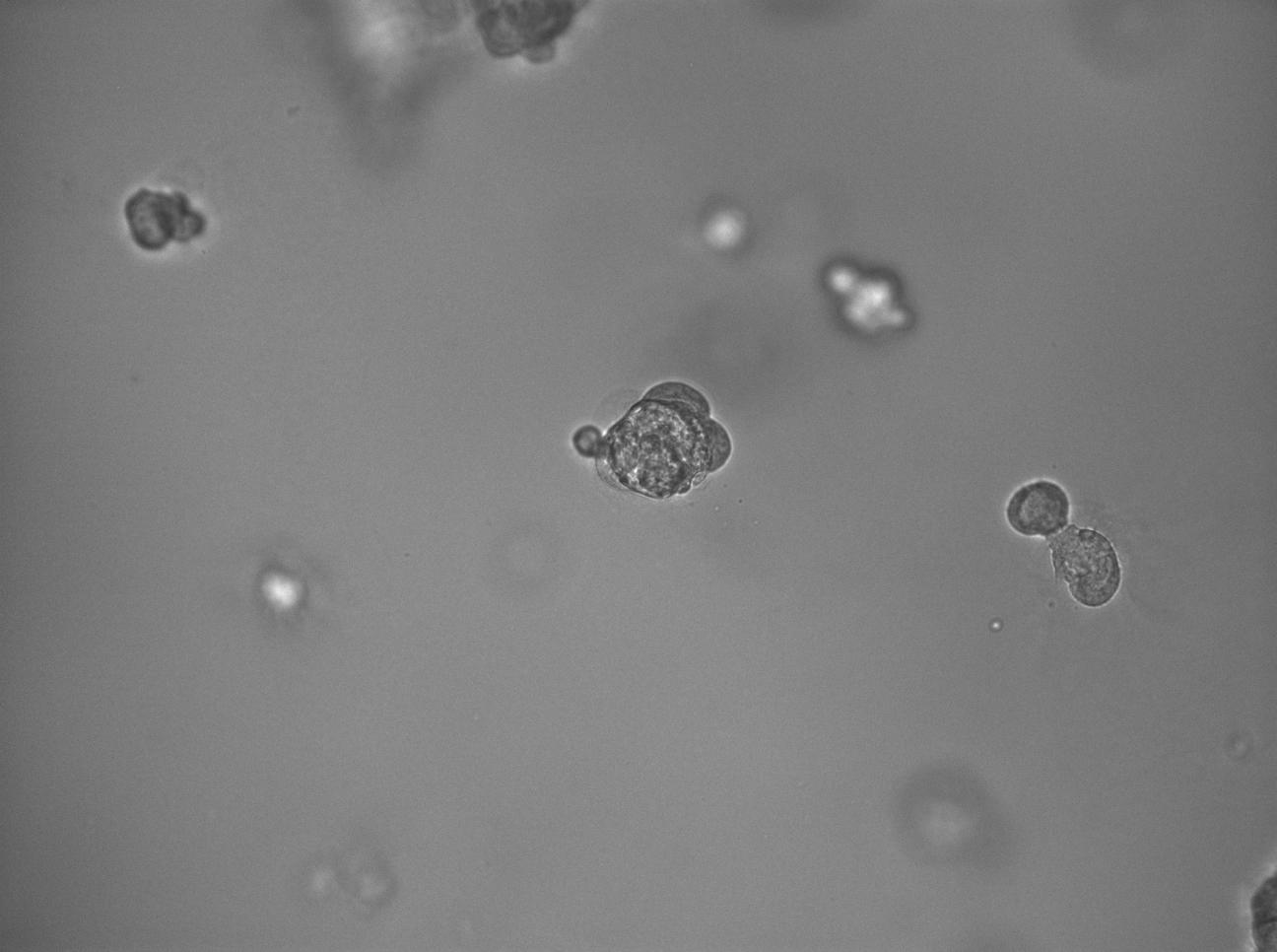

Supplement: Supplementary file 4 — Source Data Fig. 4 [file 41586_2026_10187_MOESM4_ESM.zip › HCEC1CT/HCEC1CT-KRAS_D10_Dox-00250_D04a_20x_ch00.jpg]

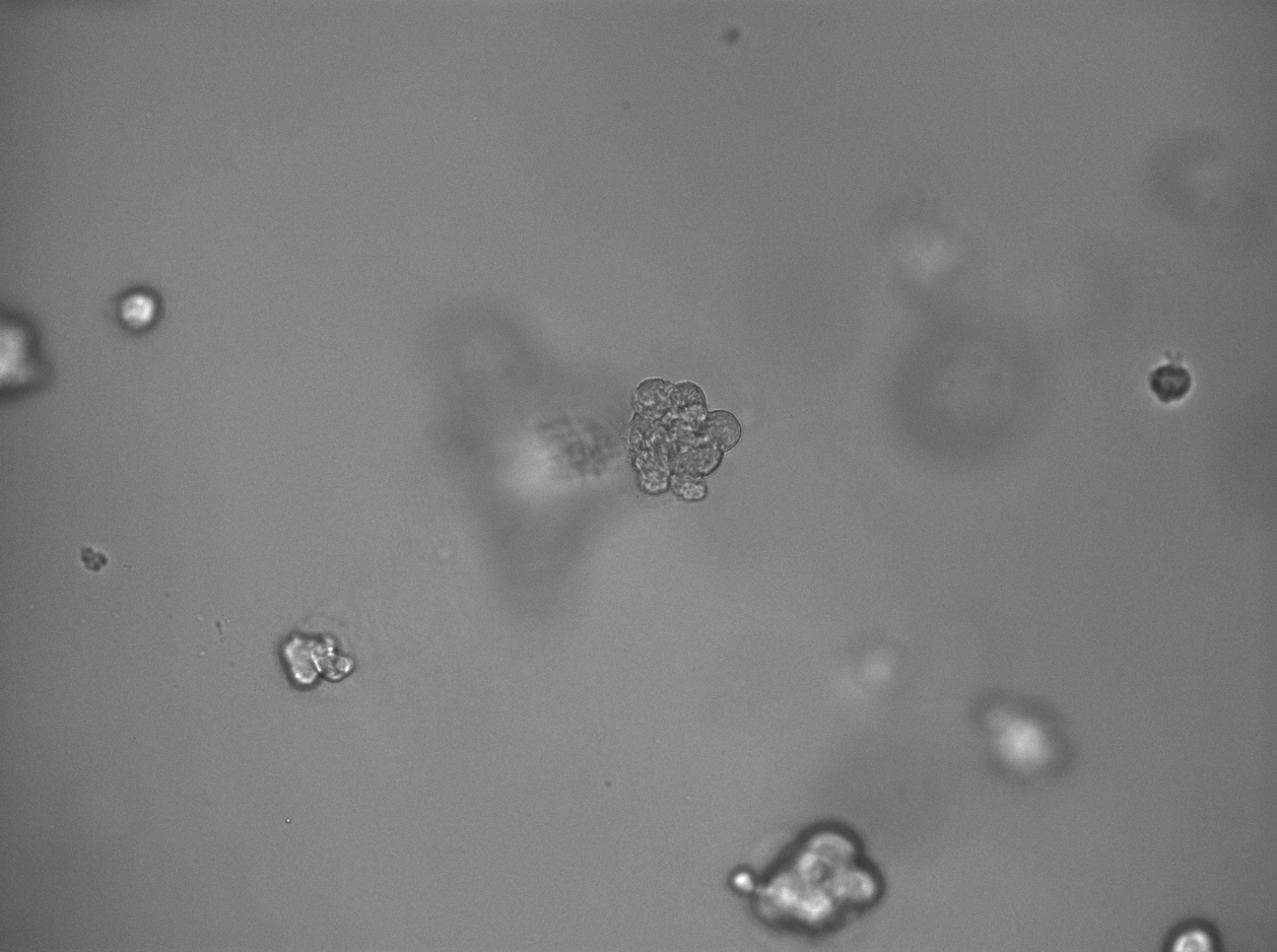

Supplement: Supplementary file 4 — Source Data Fig. 4 [file 41586_2026_10187_MOESM4_ESM.zip › HCEC1CT/HCEC1CT-KRAS_D10_Dox-00250_D04b_20x_ch00.jpg]

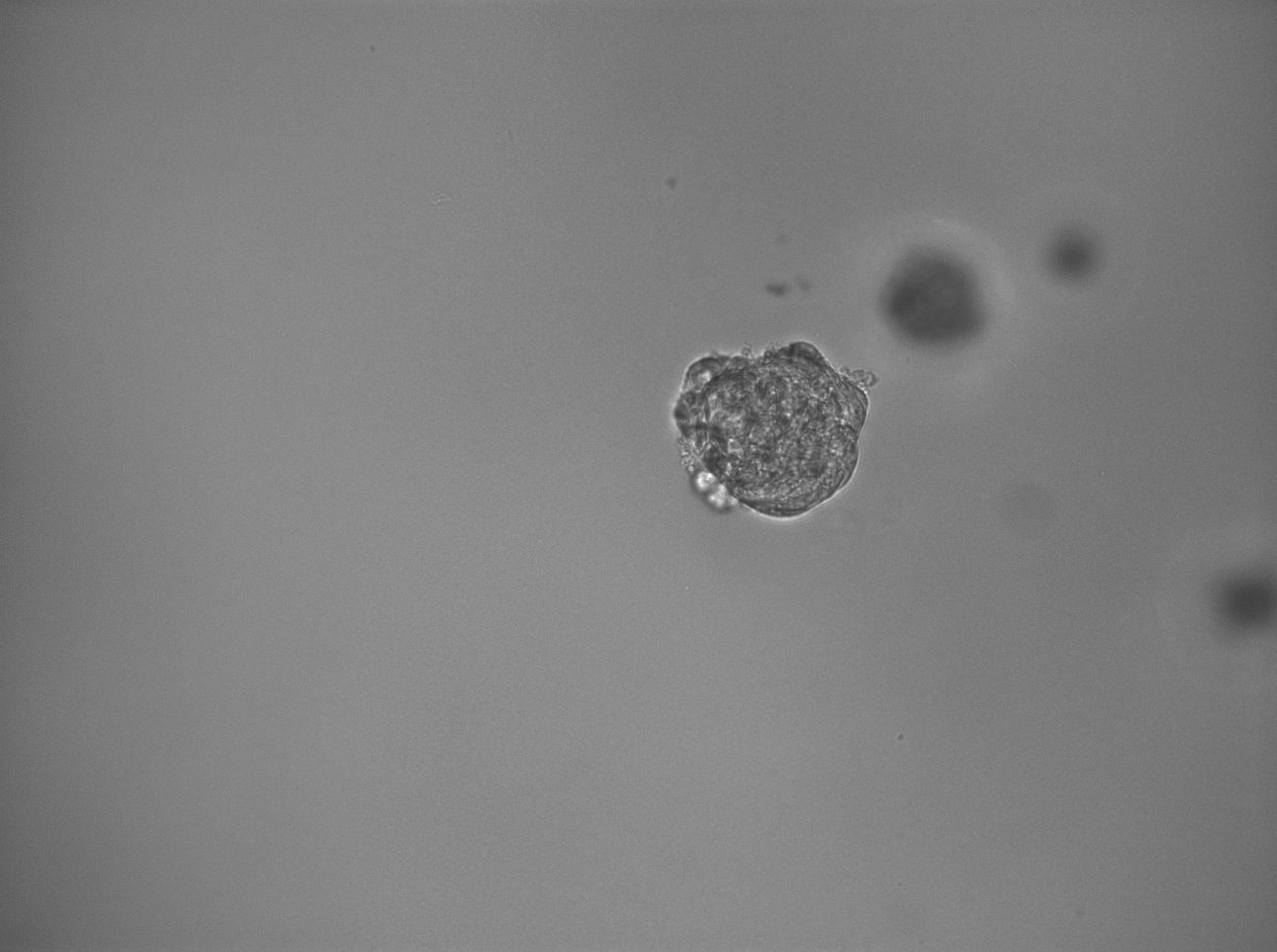

Supplement: Supplementary file 4 — Source Data Fig. 4 [file 41586_2026_10187_MOESM4_ESM.zip › HCEC1CT/HCEC1CT-KRAS_D10_Dox-00250_D04c_20x_ch00.jpg]

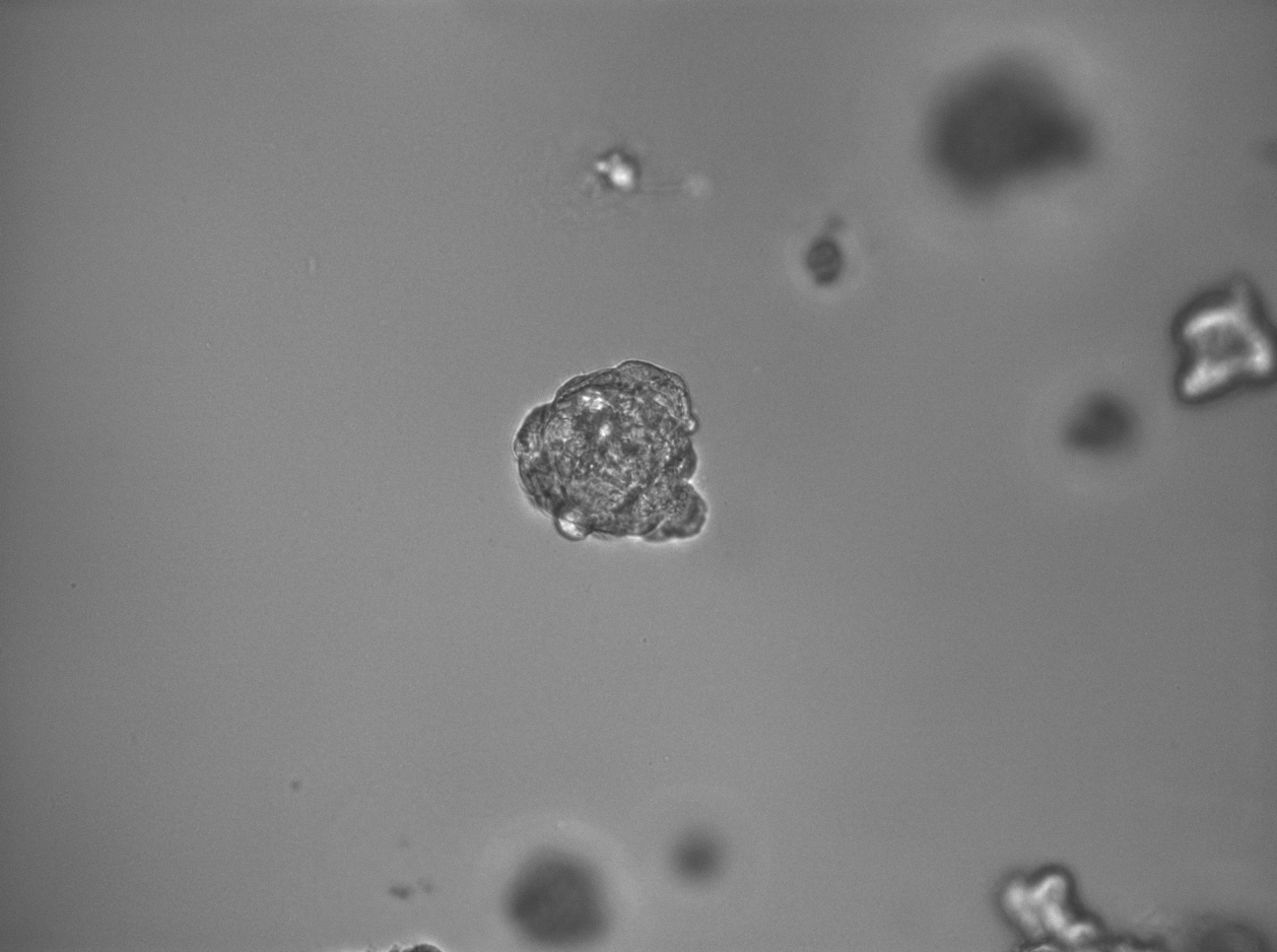

Supplement: Supplementary file 4 — Source Data Fig. 4 [file 41586_2026_10187_MOESM4_ESM.zip › HCEC1CT/HCEC1CT-KRAS_D10_Dox-00250_D04d_20x_ch00.jpg]

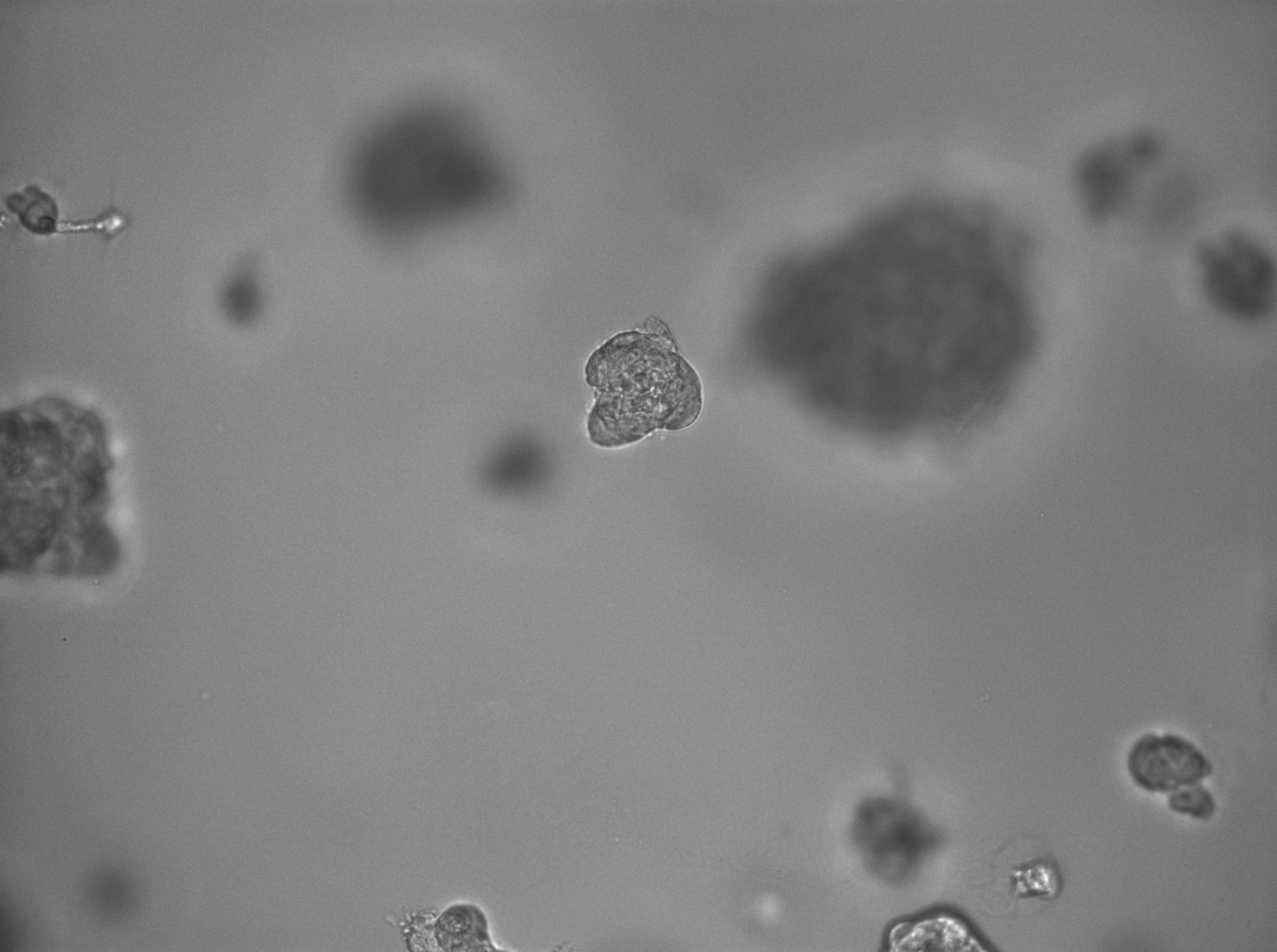

Supplement: Supplementary file 4 — Source Data Fig. 4 [file 41586_2026_10187_MOESM4_ESM.zip › HCEC1CT/HCEC1CT-KRAS_D10_Dox-00250_D04e_20x_ch00.jpg]

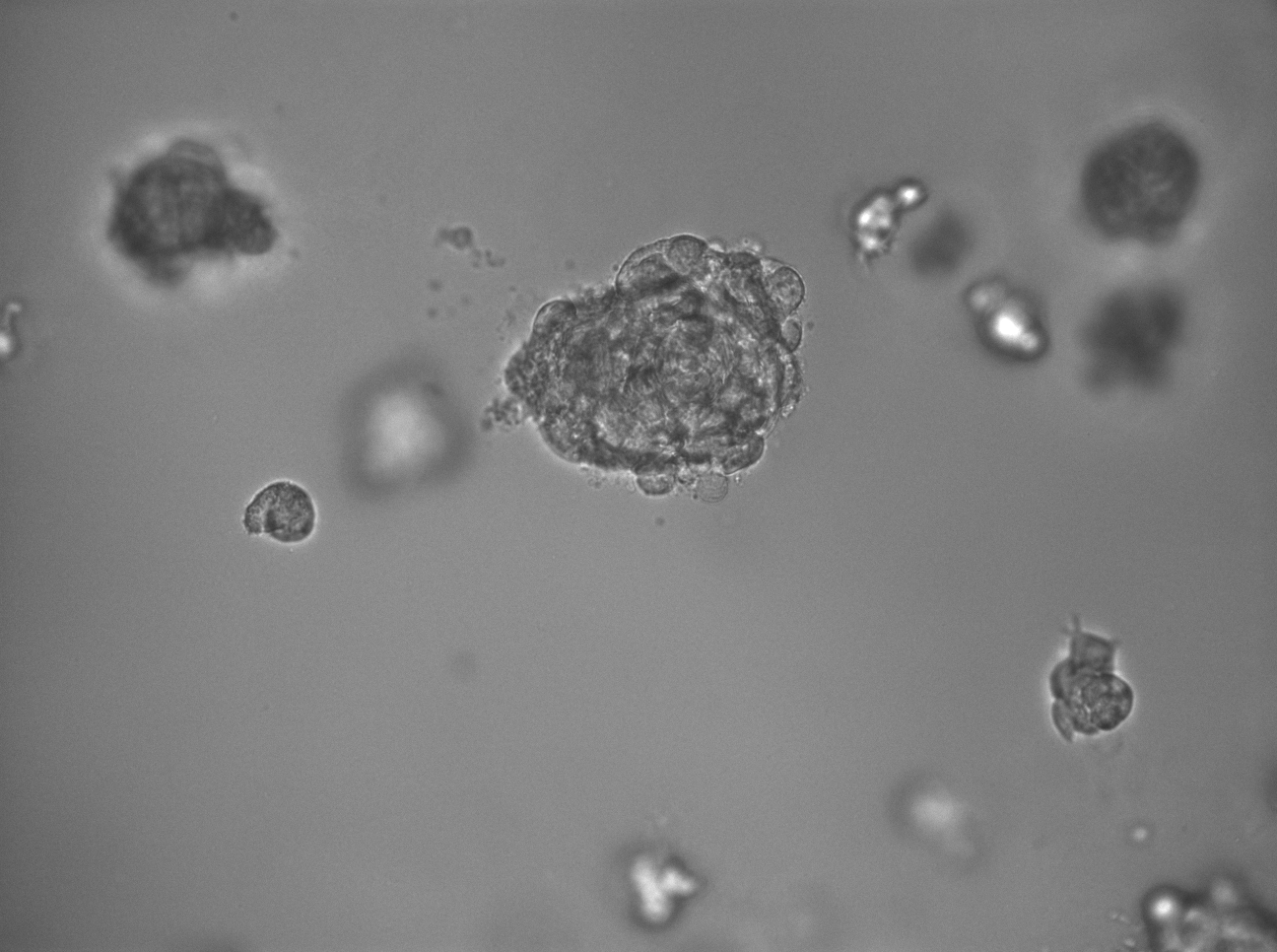

Supplement: Supplementary file 4 — Source Data Fig. 4 [file 41586_2026_10187_MOESM4_ESM.zip › HCEC1CT/HCEC1CT-KRAS_D10_Dox-00250_D04f_20x_ch00.jpg]

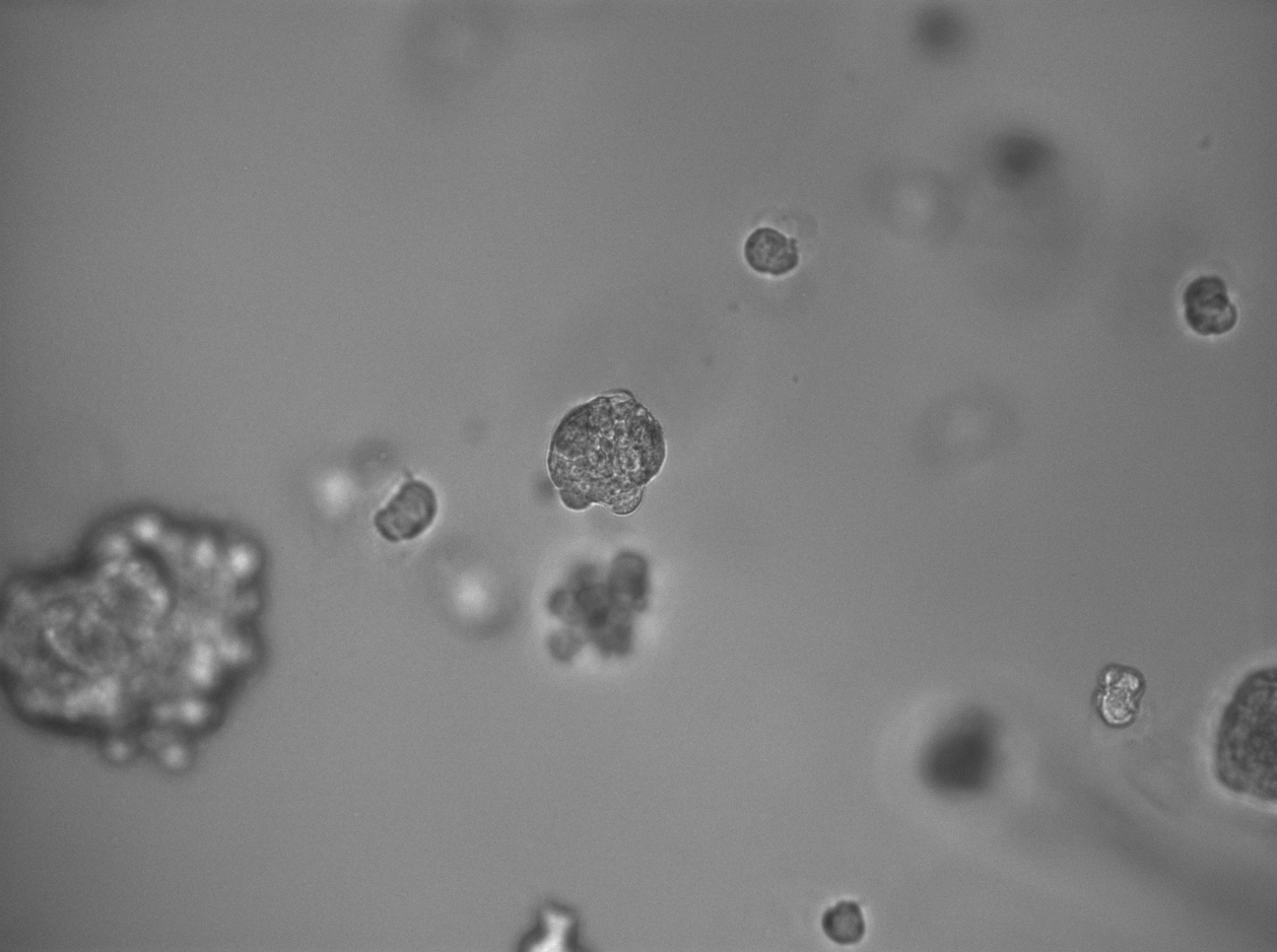

Supplement: Supplementary file 4 — Source Data Fig. 4 [file 41586_2026_10187_MOESM4_ESM.zip › HCEC1CT/HCEC1CT-KRAS_D10_Dox-00250_D04g_20x_ch00.jpg]

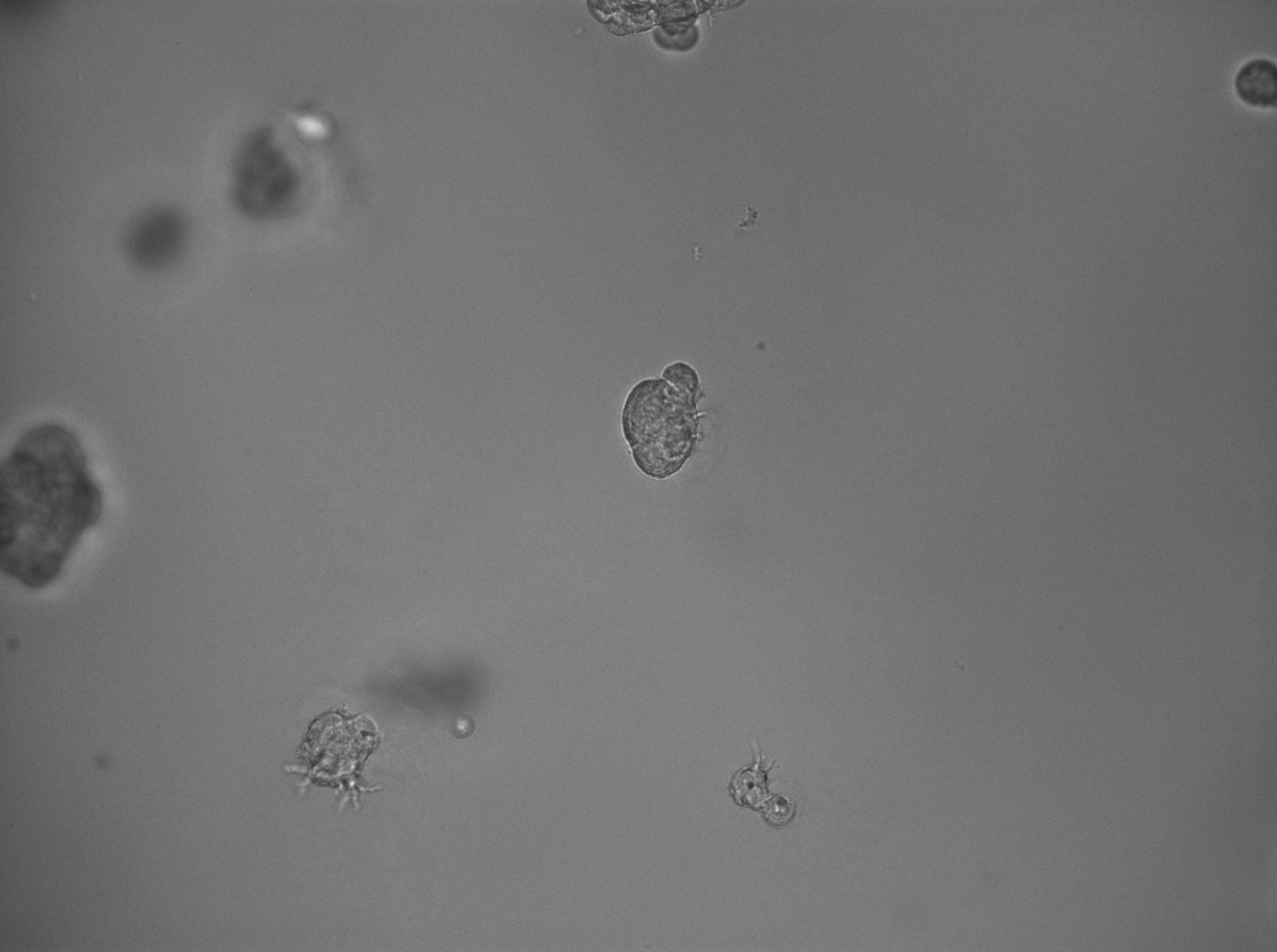

Supplement: Supplementary file 4 — Source Data Fig. 4 [file 41586_2026_10187_MOESM4_ESM.zip › HCEC1CT/HCEC1CT-KRAS_D10_Dox-00500_E01a_20x_ch00.jpg]

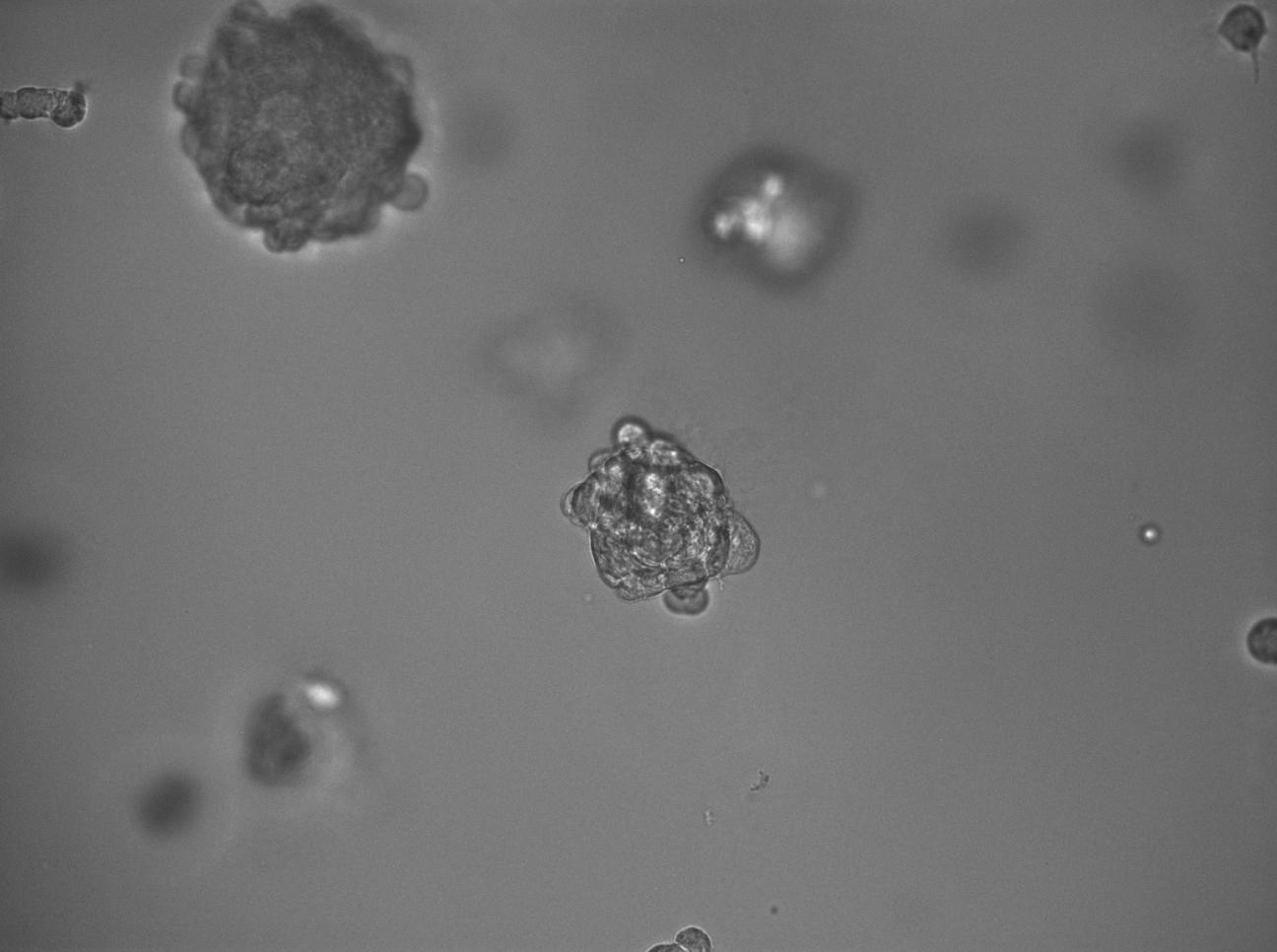

Supplement: Supplementary file 4 — Source Data Fig. 4 [file 41586_2026_10187_MOESM4_ESM.zip › HCEC1CT/HCEC1CT-KRAS_D10_Dox-00500_E01b_20x_ch00.jpg]

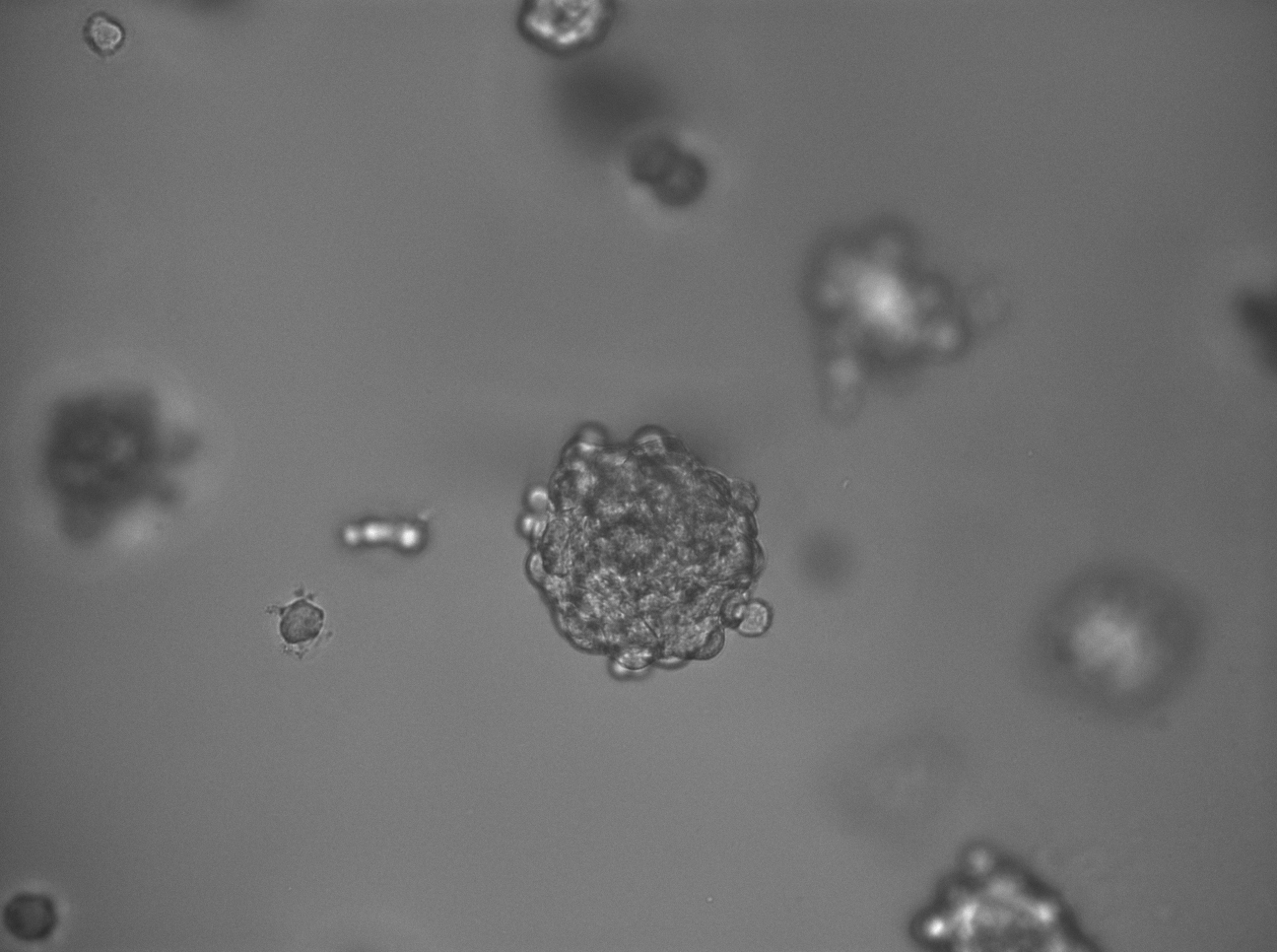

Supplement: Supplementary file 4 — Source Data Fig. 4 [file 41586_2026_10187_MOESM4_ESM.zip › HCEC1CT/HCEC1CT-KRAS_D10_Dox-00500_E01c_20x_ch00.jpg]

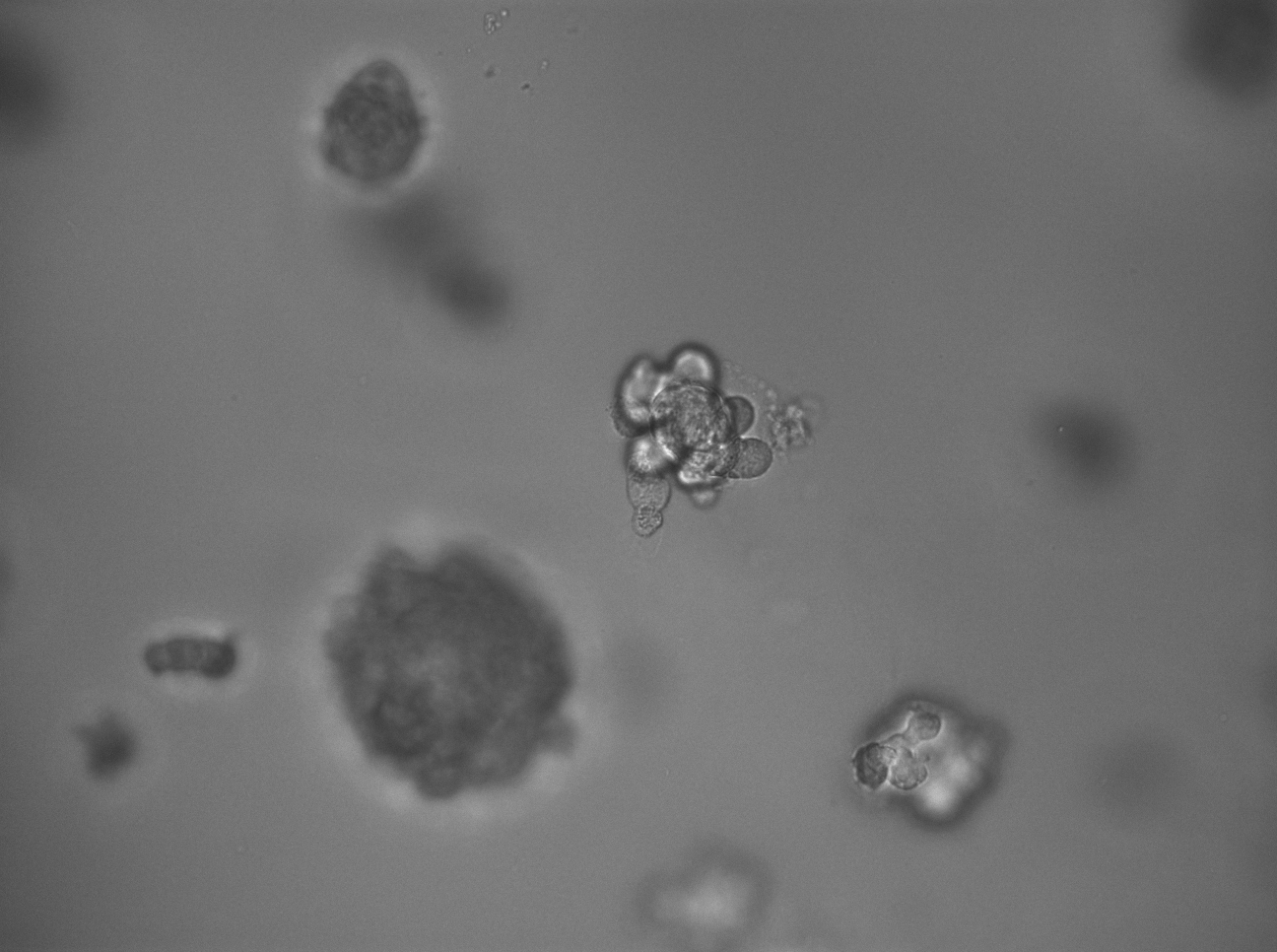

Supplement: Supplementary file 4 — Source Data Fig. 4 [file 41586_2026_10187_MOESM4_ESM.zip › HCEC1CT/HCEC1CT-KRAS_D10_Dox-00500_E01d_20x_ch00.jpg]

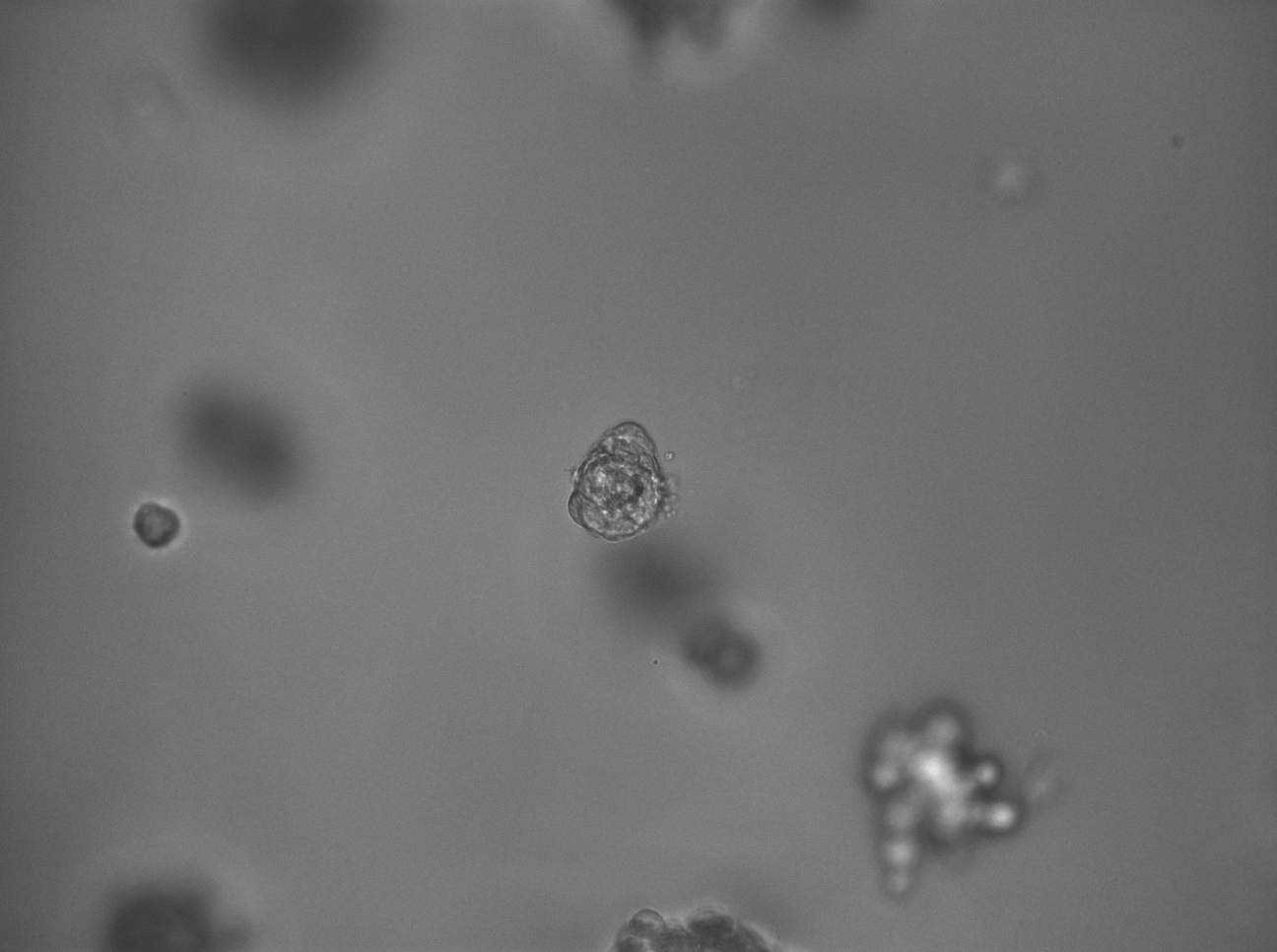

Supplement: Supplementary file 4 — Source Data Fig. 4 [file 41586_2026_10187_MOESM4_ESM.zip › HCEC1CT/HCEC1CT-KRAS_D10_Dox-00500_E01e_20x_ch00.jpg]

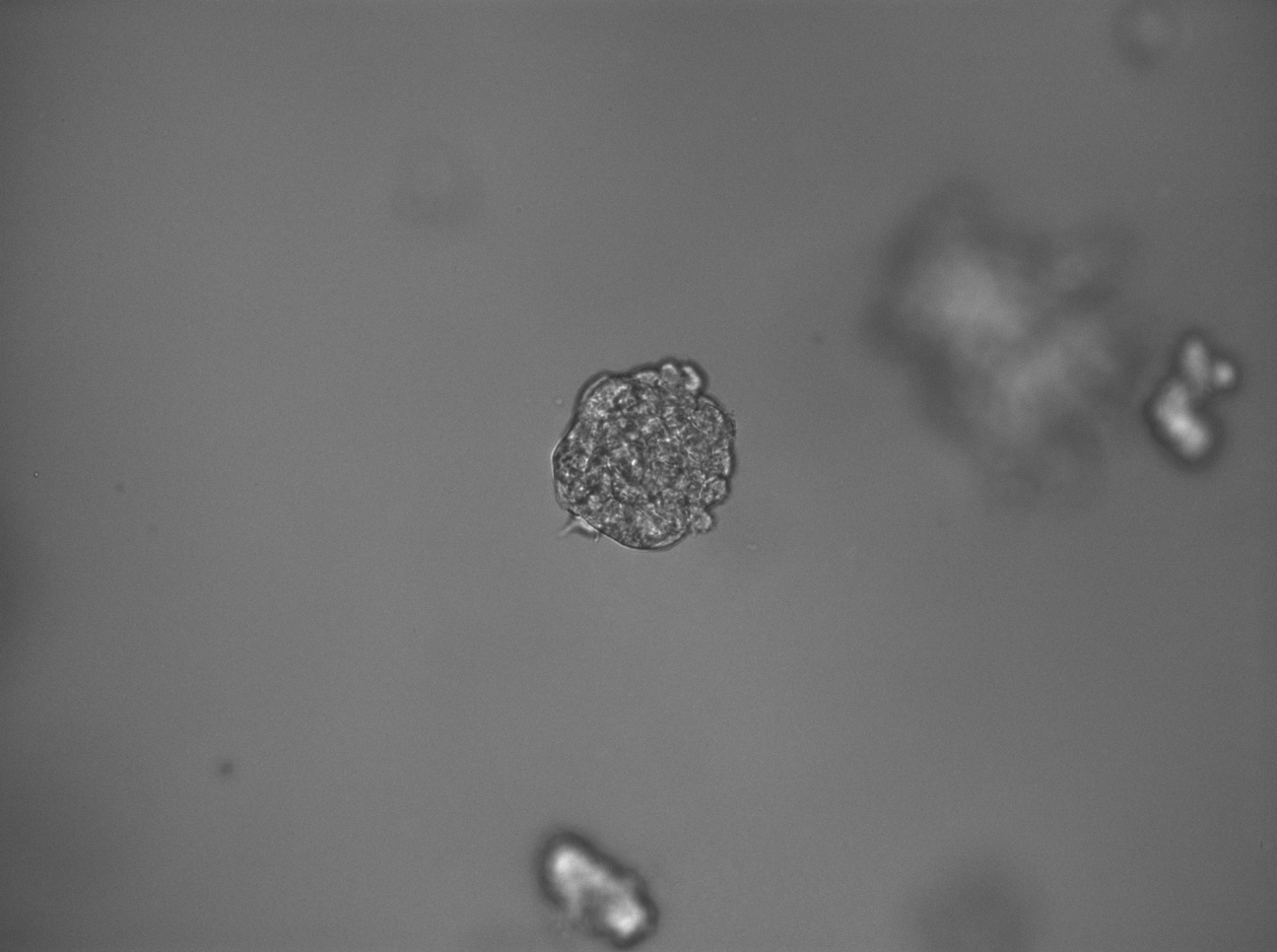

Supplement: Supplementary file 4 — Source Data Fig. 4 [file 41586_2026_10187_MOESM4_ESM.zip › HCEC1CT/HCEC1CT-KRAS_D10_Dox-00500_E01f_20x_ch00.jpg]

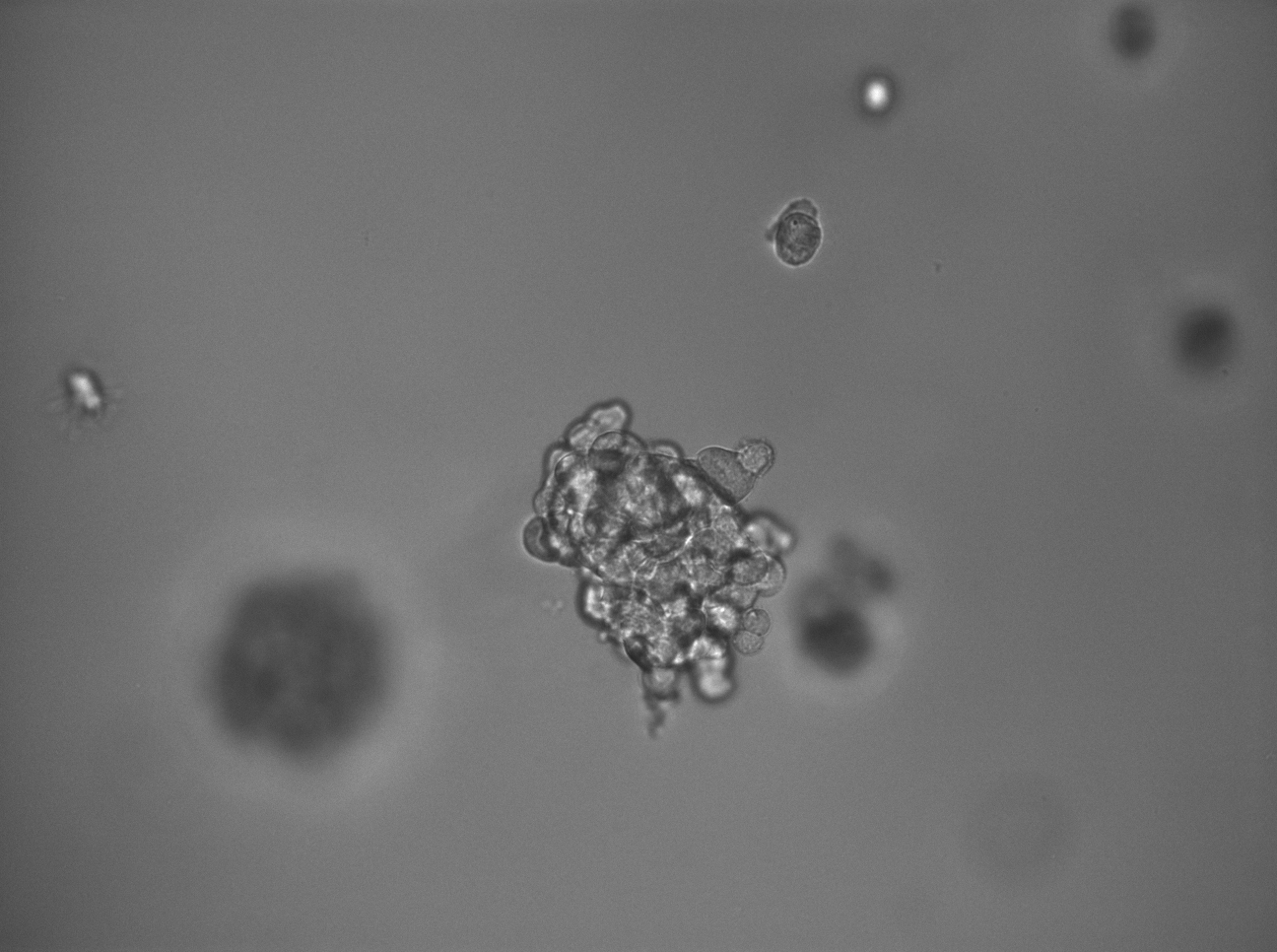

Supplement: Supplementary file 4 — Source Data Fig. 4 [file 41586_2026_10187_MOESM4_ESM.zip › HCEC1CT/HCEC1CT-KRAS_D10_Dox-00500_E01g_20x_ch00.jpg]

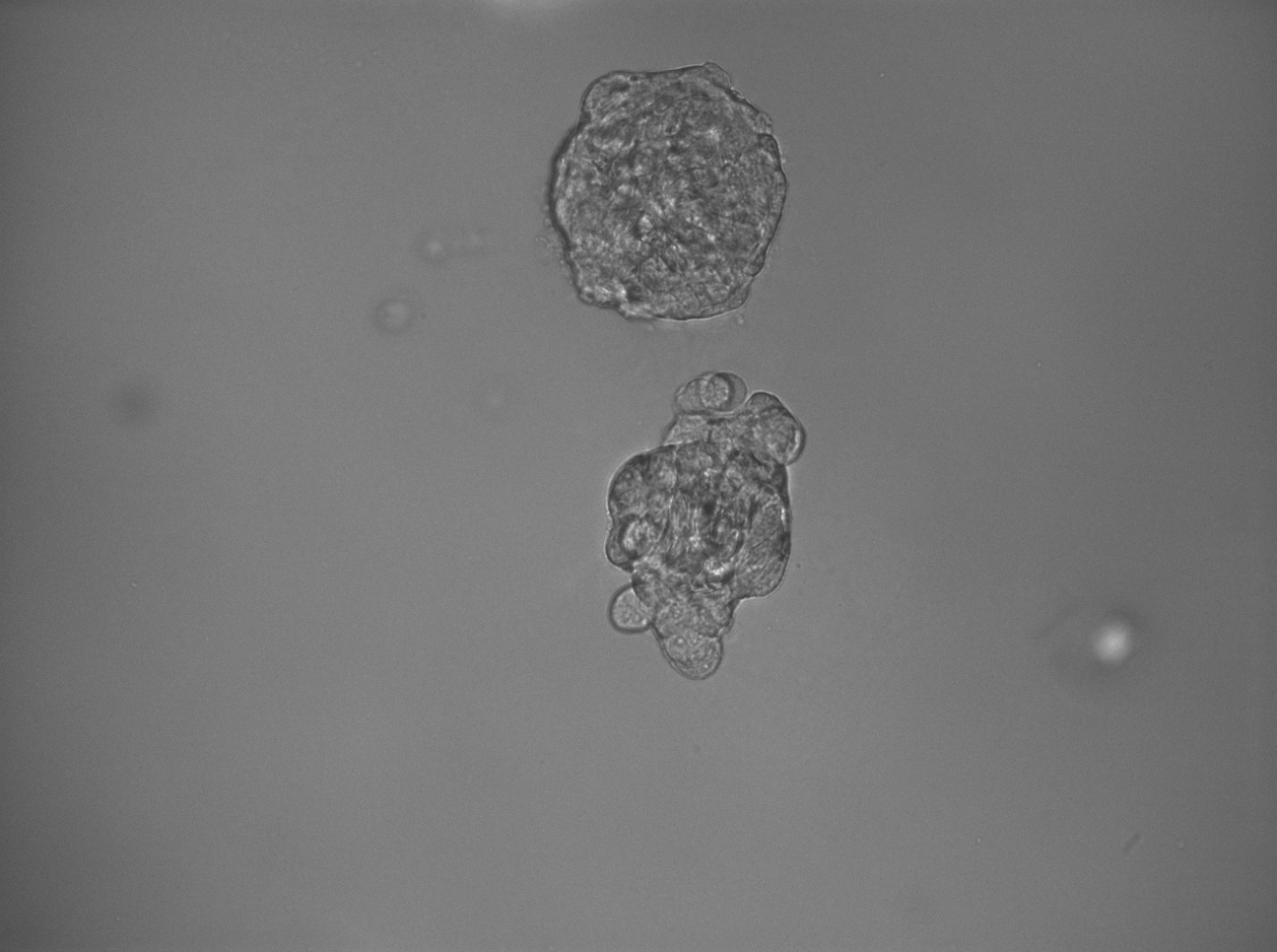

Supplement: Supplementary file 4 — Source Data Fig. 4 [file 41586_2026_10187_MOESM4_ESM.zip › HCEC1CT/HCEC1CT-KRAS_D10_Dox-00500_E02a_20x_ch00.jpg]

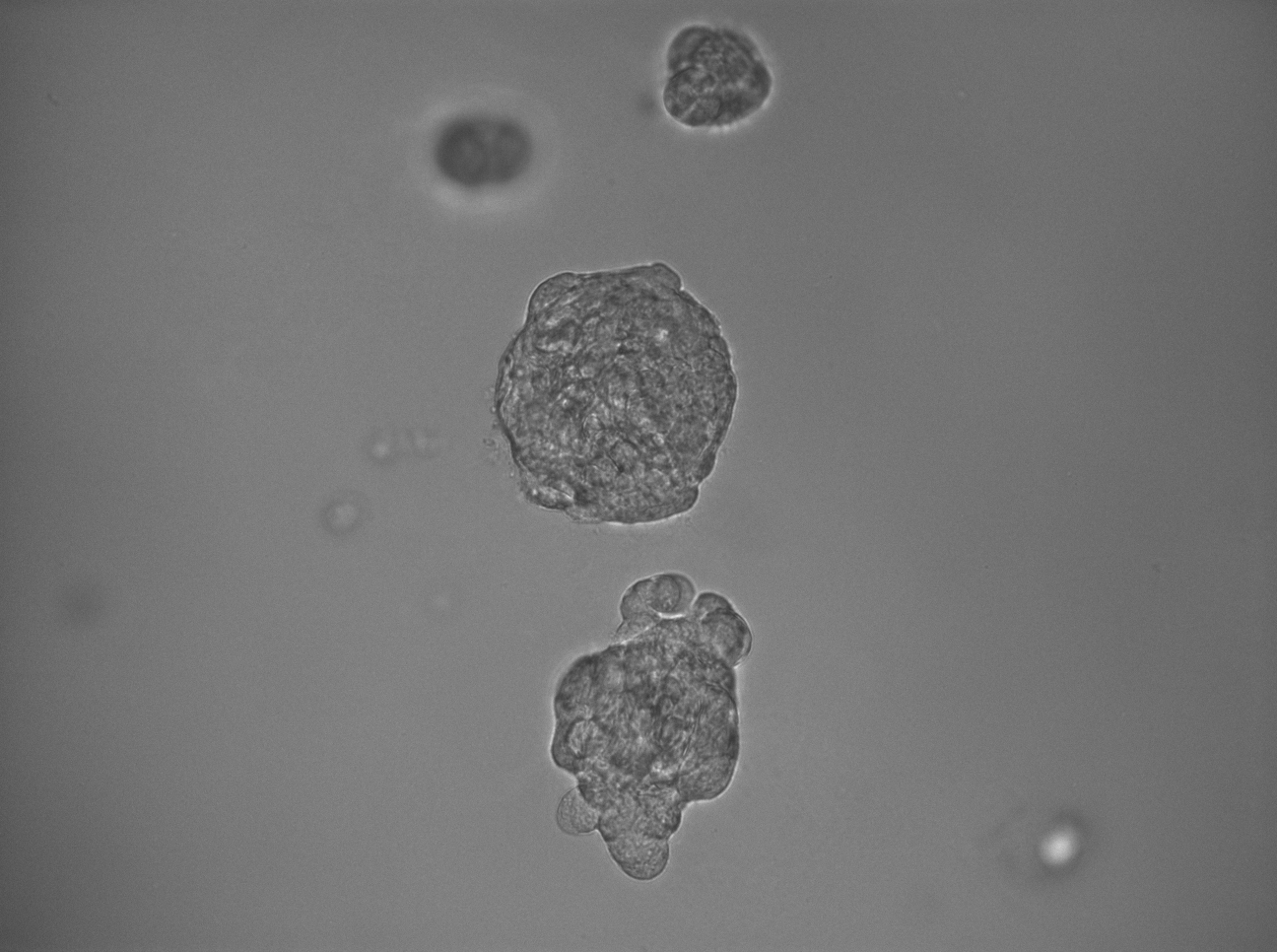

Supplement: Supplementary file 4 — Source Data Fig. 4 [file 41586_2026_10187_MOESM4_ESM.zip › HCEC1CT/HCEC1CT-KRAS_D10_Dox-00500_E02b_20x_ch00.jpg]

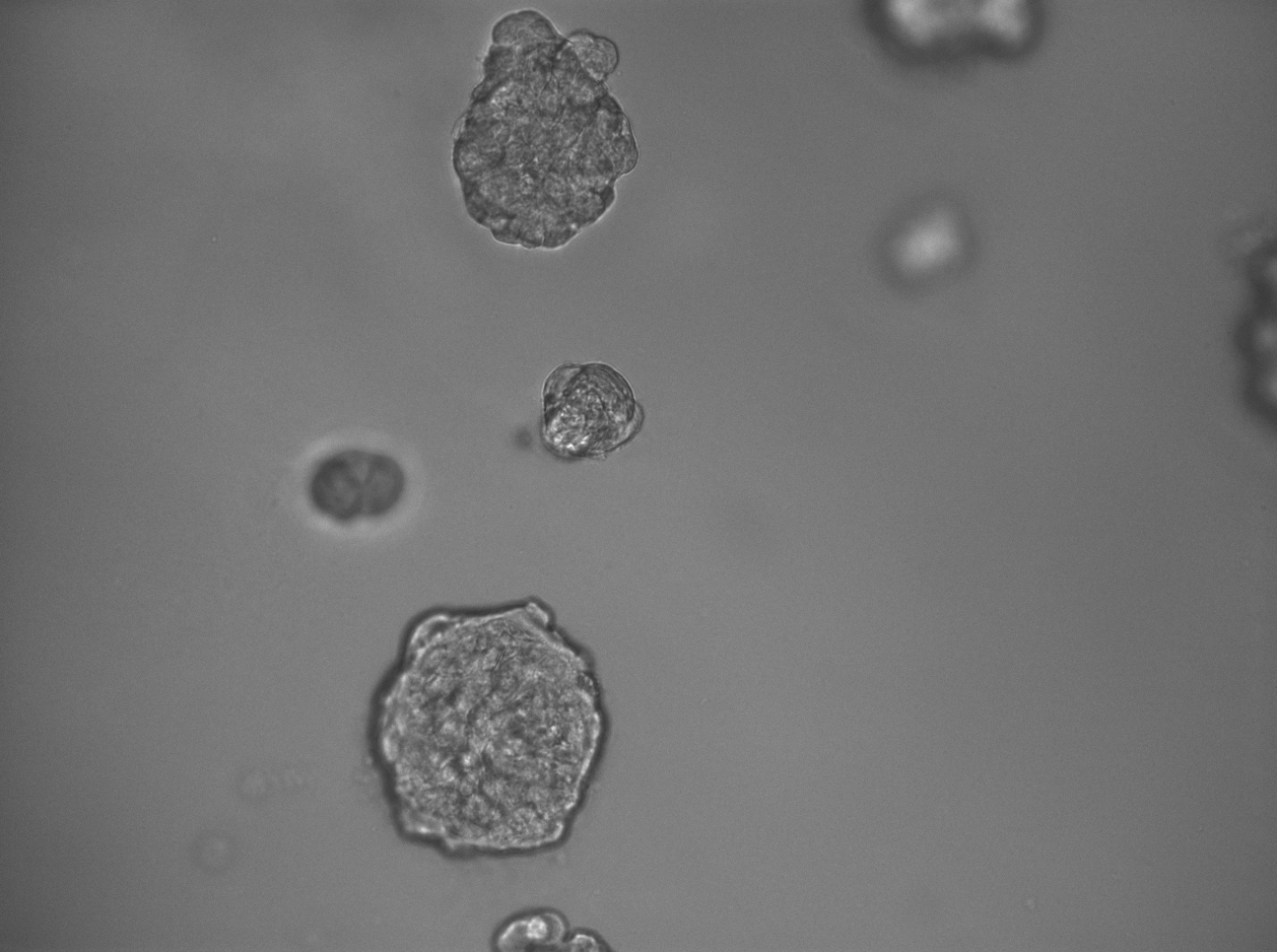

Supplement: Supplementary file 4 — Source Data Fig. 4 [file 41586_2026_10187_MOESM4_ESM.zip › HCEC1CT/HCEC1CT-KRAS_D10_Dox-00500_E02c_20x_ch00.jpg]

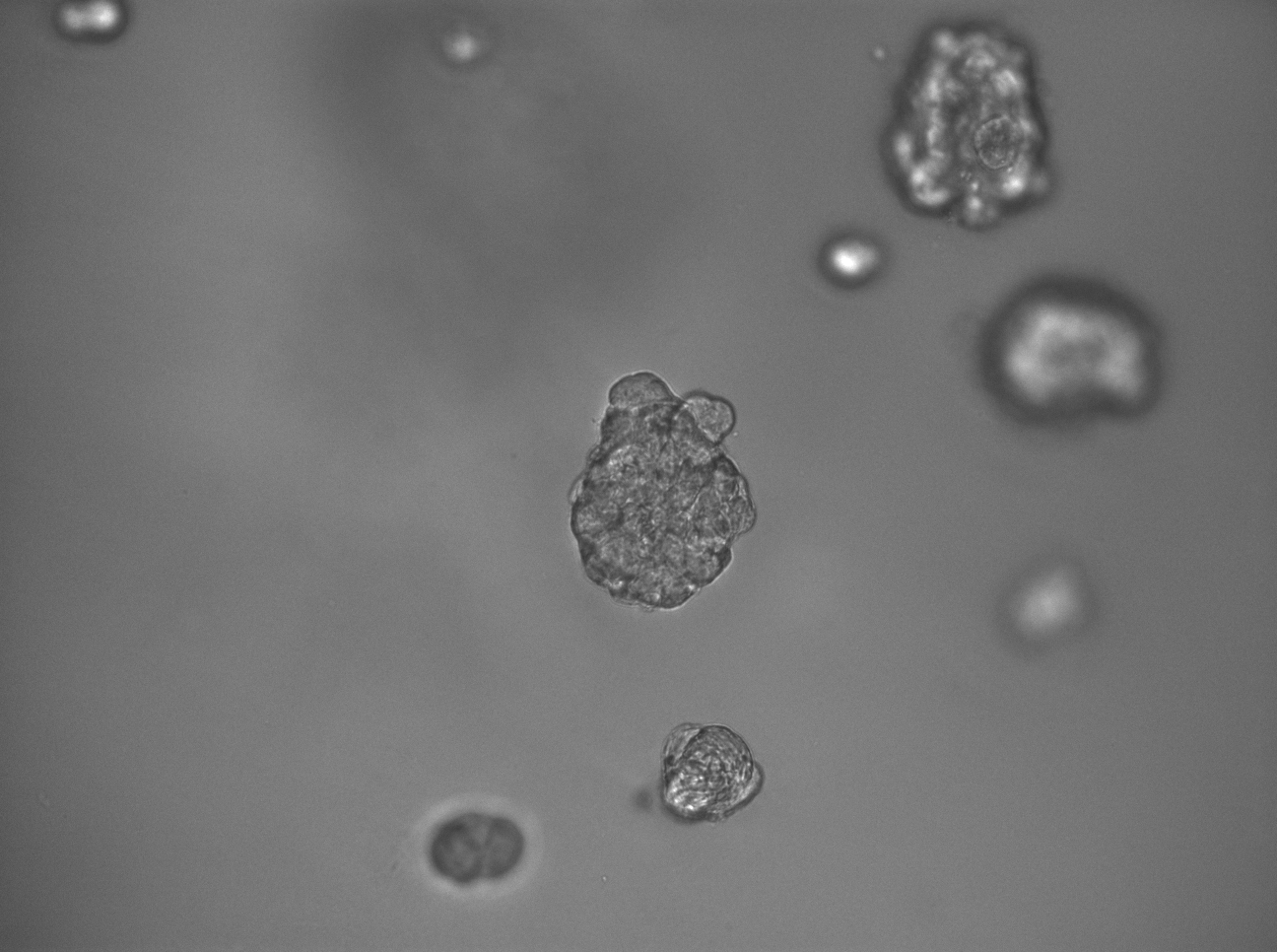

Supplement: Supplementary file 4 — Source Data Fig. 4 [file 41586_2026_10187_MOESM4_ESM.zip › HCEC1CT/HCEC1CT-KRAS_D10_Dox-00500_E02d_20x_ch00.jpg]

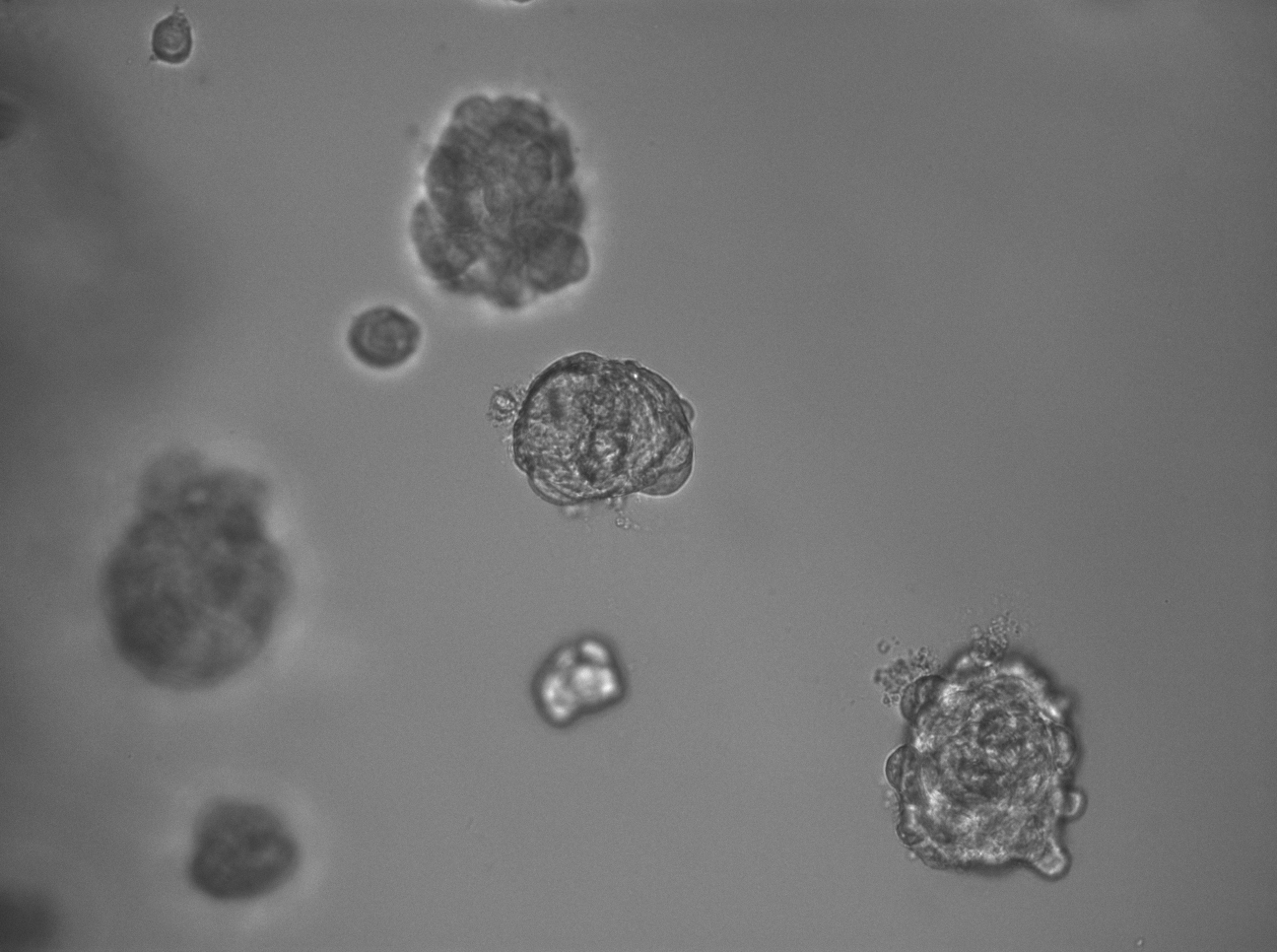

Supplement: Supplementary file 4 — Source Data Fig. 4 [file 41586_2026_10187_MOESM4_ESM.zip › HCEC1CT/HCEC1CT-KRAS_D10_Dox-00500_E02e_20x_ch00.jpg]

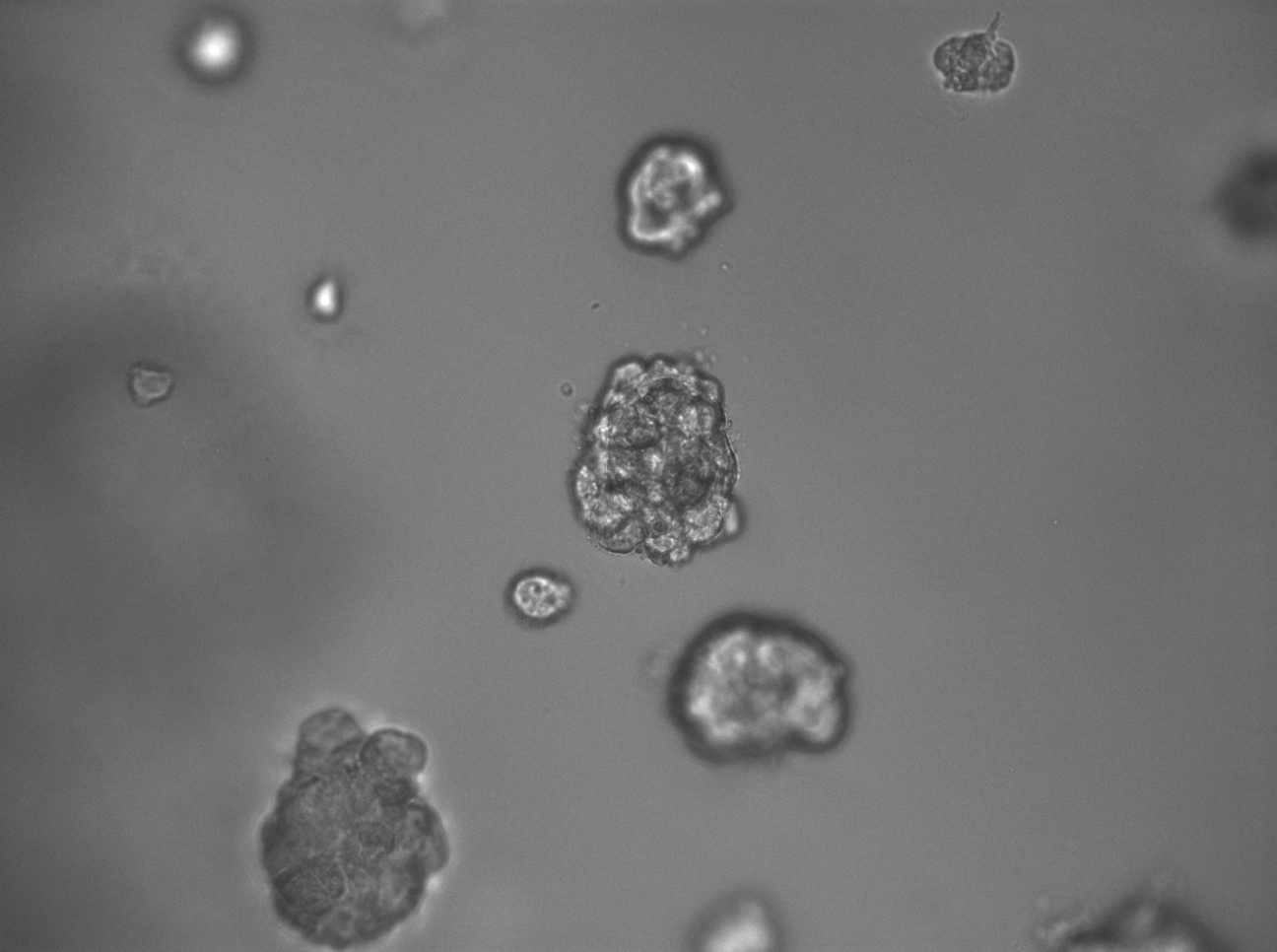

Supplement: Supplementary file 4 — Source Data Fig. 4 [file 41586_2026_10187_MOESM4_ESM.zip › HCEC1CT/HCEC1CT-KRAS_D10_Dox-00500_E02f_20x_ch00.jpg]

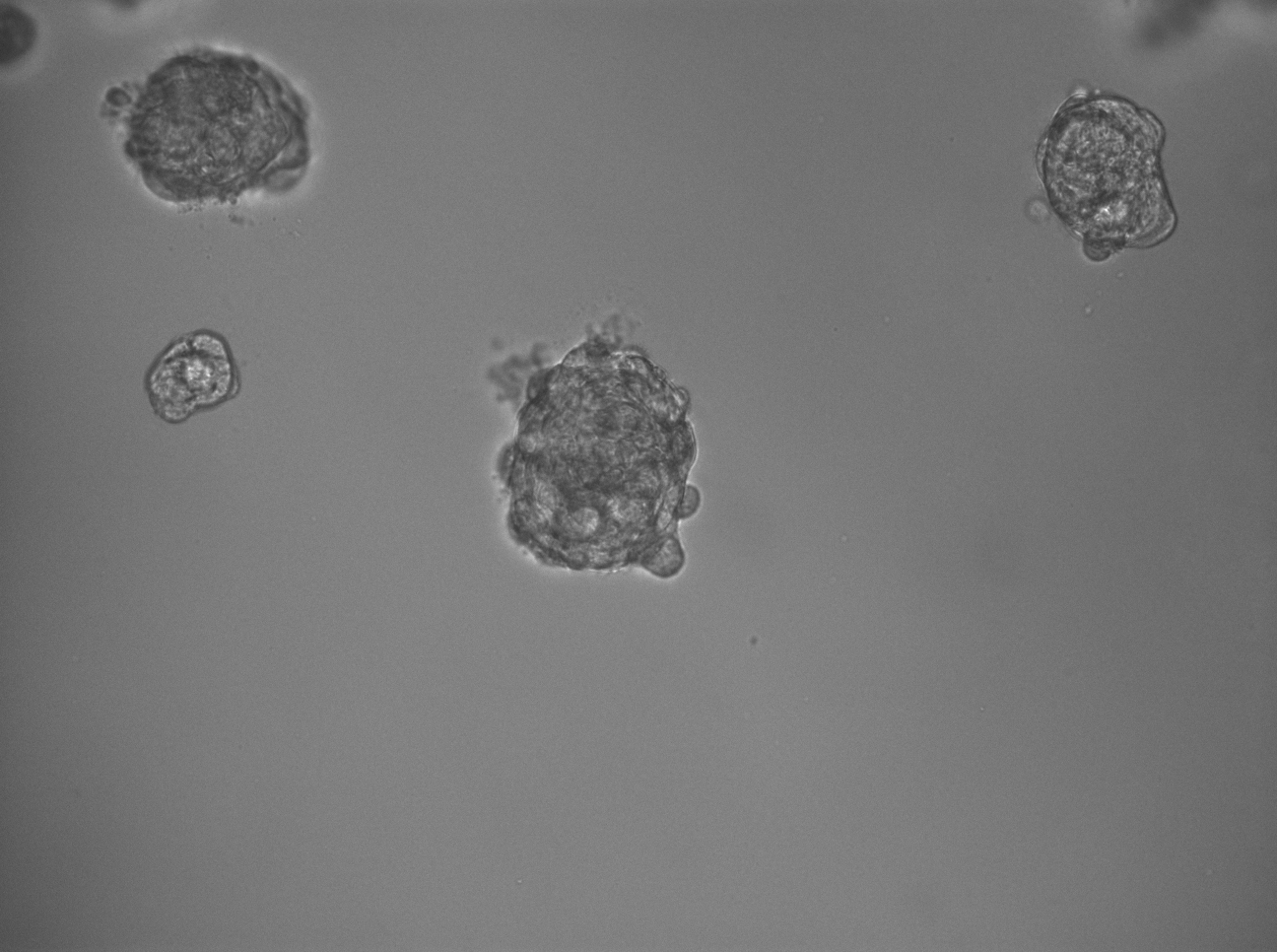

Supplement: Supplementary file 4 — Source Data Fig. 4 [file 41586_2026_10187_MOESM4_ESM.zip › HCEC1CT/HCEC1CT-KRAS_D10_Dox-00500_E02g_20x_ch00.jpg]

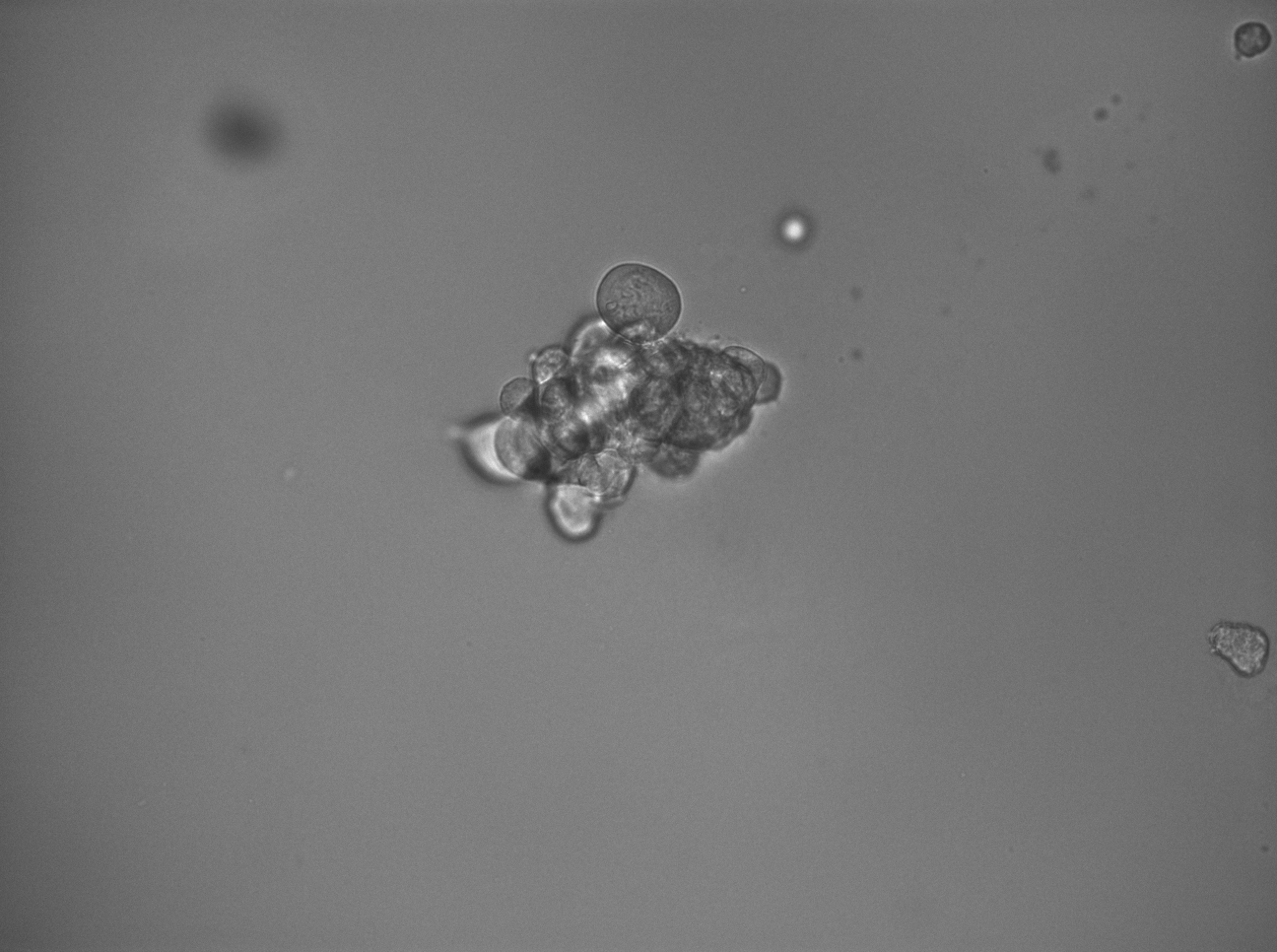

Supplement: Supplementary file 4 — Source Data Fig. 4 [file 41586_2026_10187_MOESM4_ESM.zip › HCEC1CT/HCEC1CT-KRAS_D10_Dox-00500_E03a_20x_ch00.jpg]

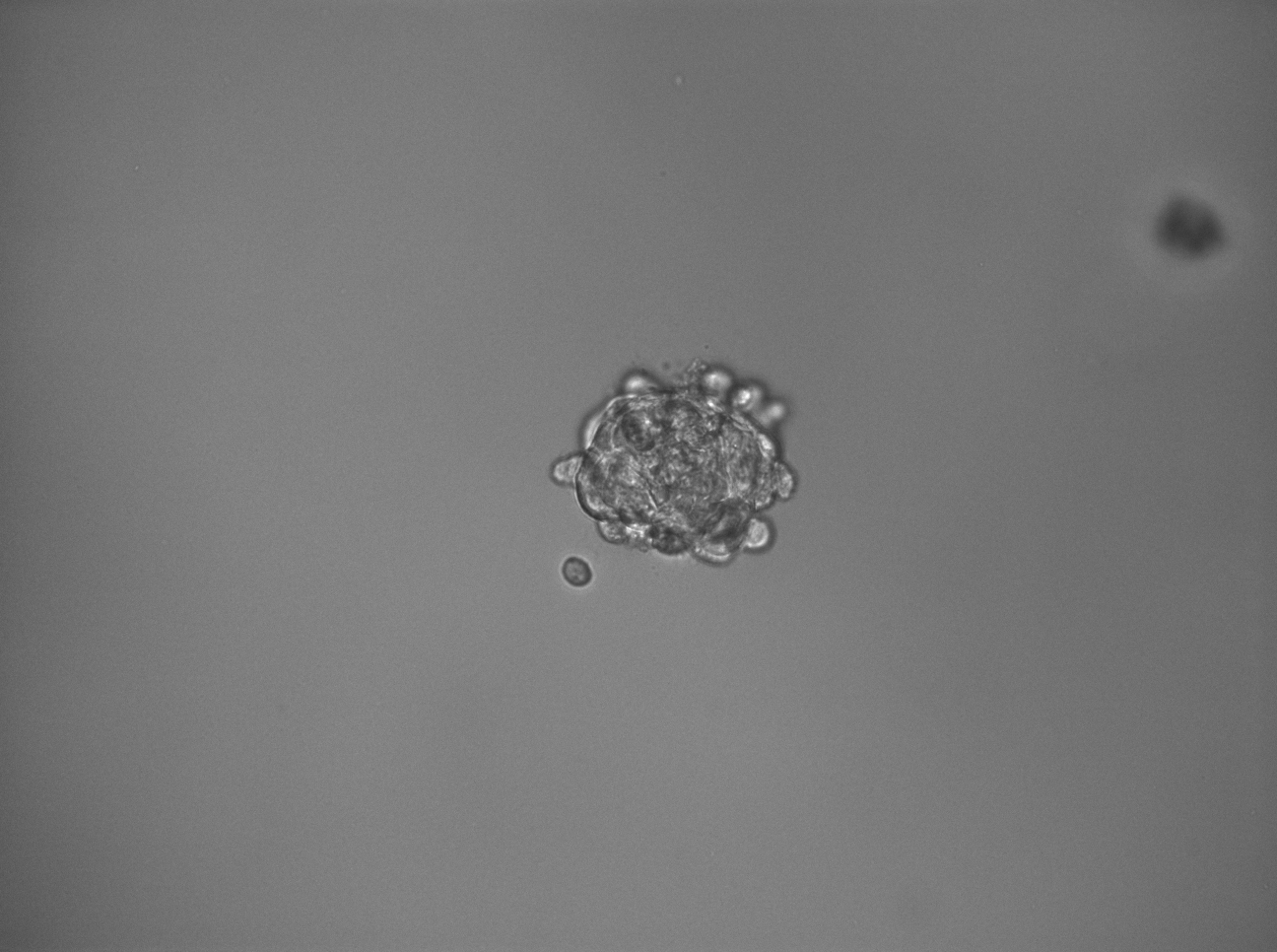

Supplement: Supplementary file 4 — Source Data Fig. 4 [file 41586_2026_10187_MOESM4_ESM.zip › HCEC1CT/HCEC1CT-KRAS_D10_Dox-00500_E03b_20x_ch00.jpg]

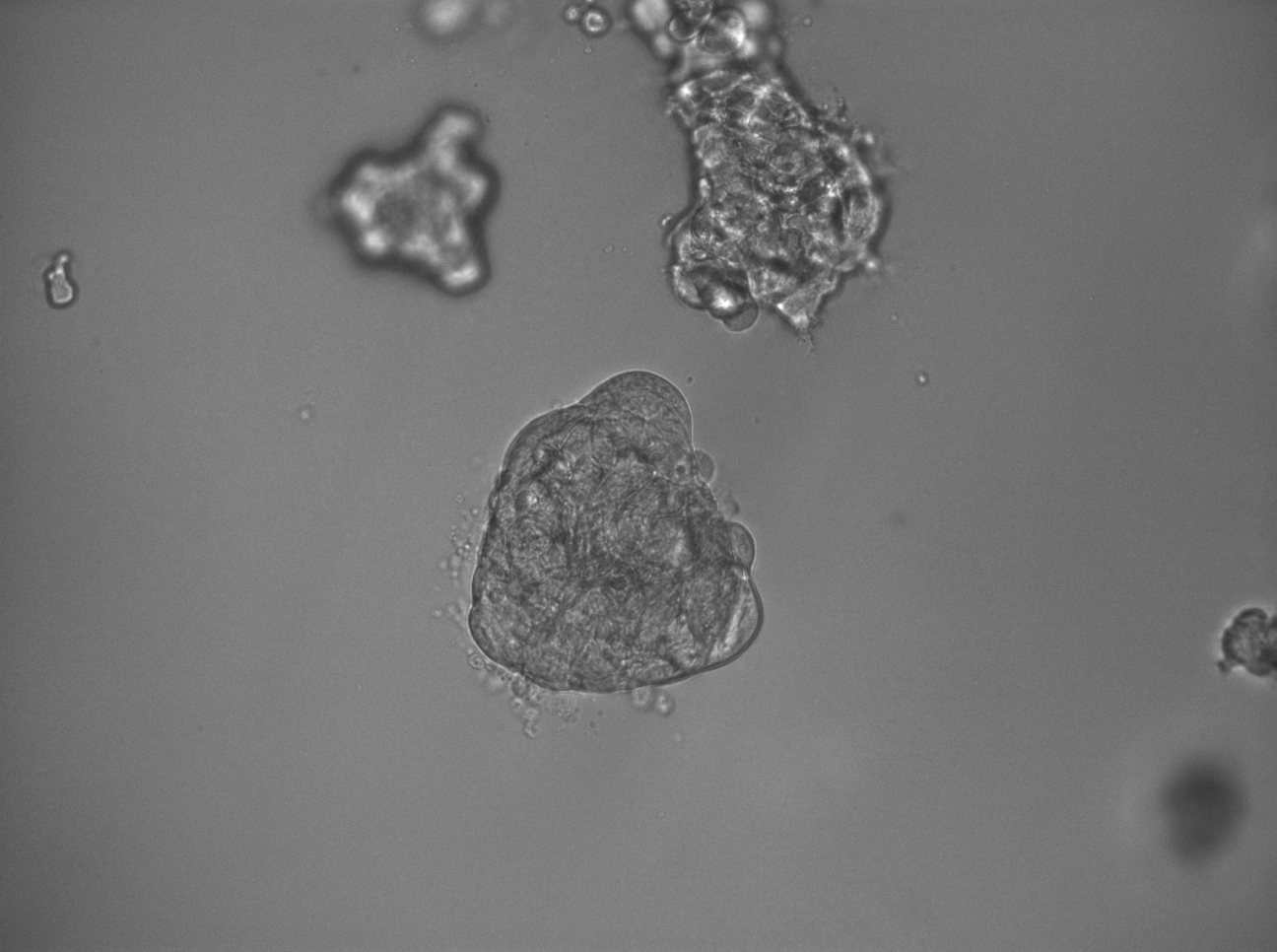

Supplement: Supplementary file 4 — Source Data Fig. 4 [file 41586_2026_10187_MOESM4_ESM.zip › HCEC1CT/HCEC1CT-KRAS_D10_Dox-00500_E03c_20x_ch00.jpg]

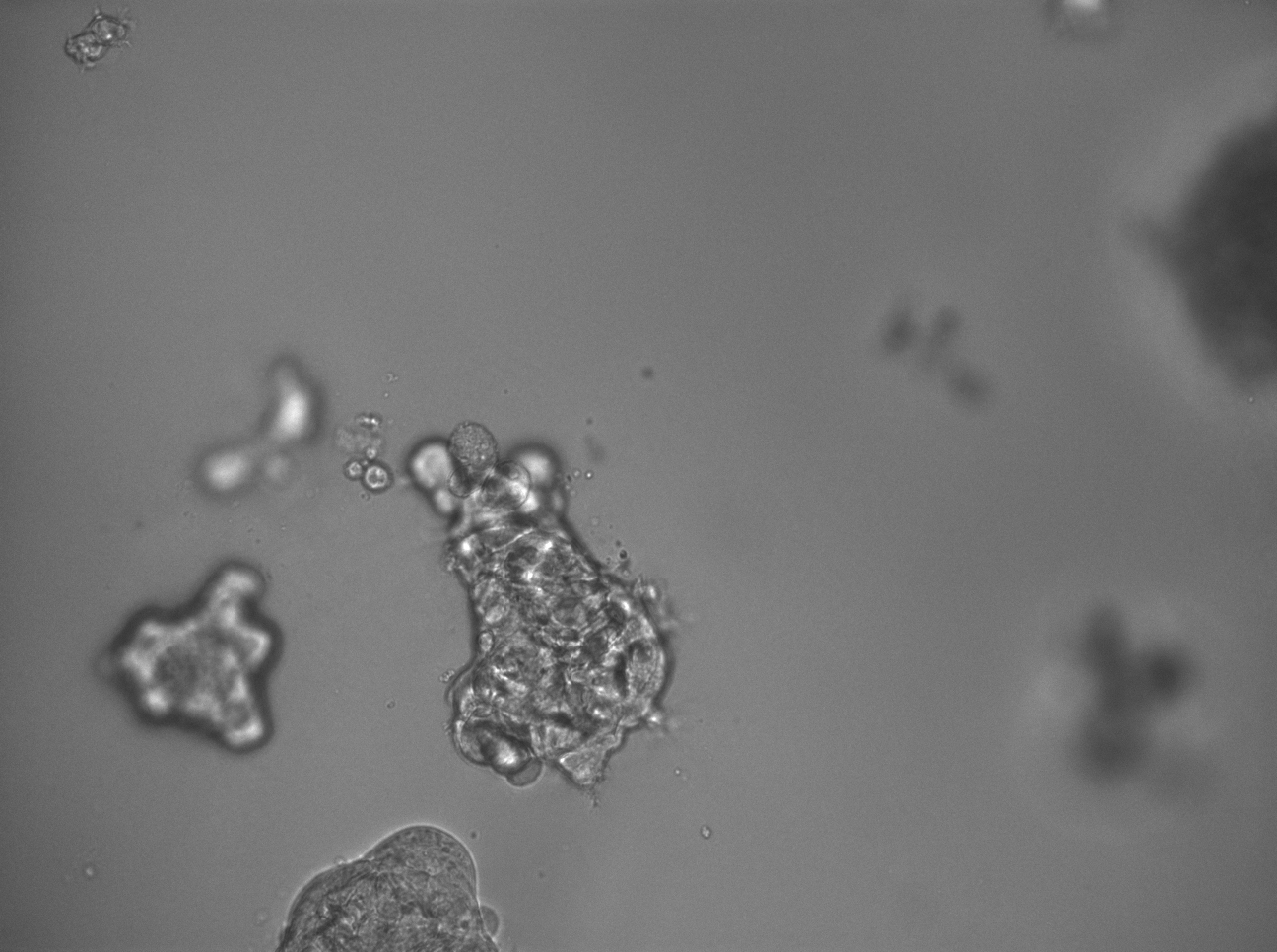

Supplement: Supplementary file 4 — Source Data Fig. 4 [file 41586_2026_10187_MOESM4_ESM.zip › HCEC1CT/HCEC1CT-KRAS_D10_Dox-00500_E03d_20x_ch00.jpg]

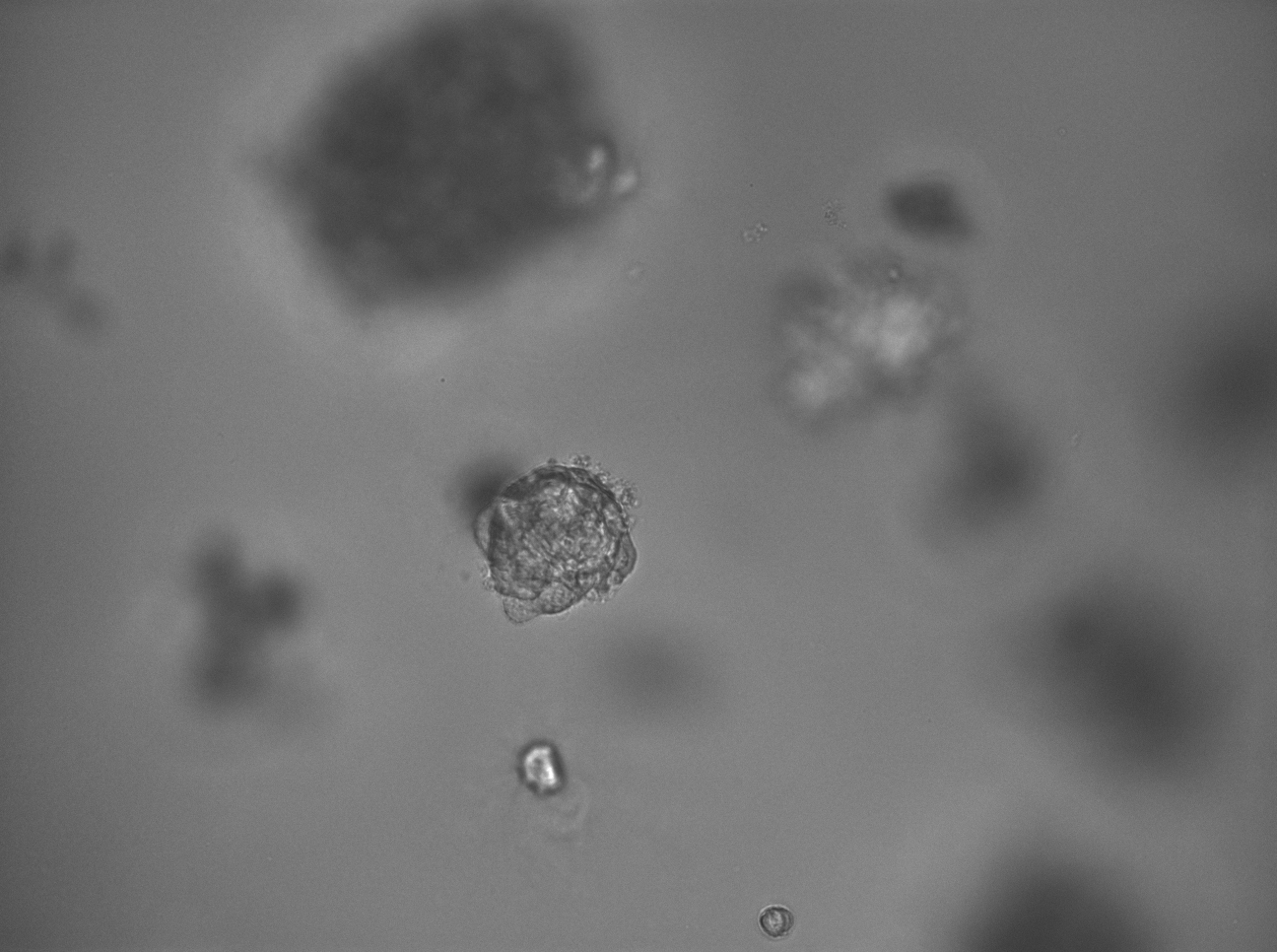

Supplement: Supplementary file 4 — Source Data Fig. 4 [file 41586_2026_10187_MOESM4_ESM.zip › HCEC1CT/HCEC1CT-KRAS_D10_Dox-00500_E03e_20x_ch00.jpg]

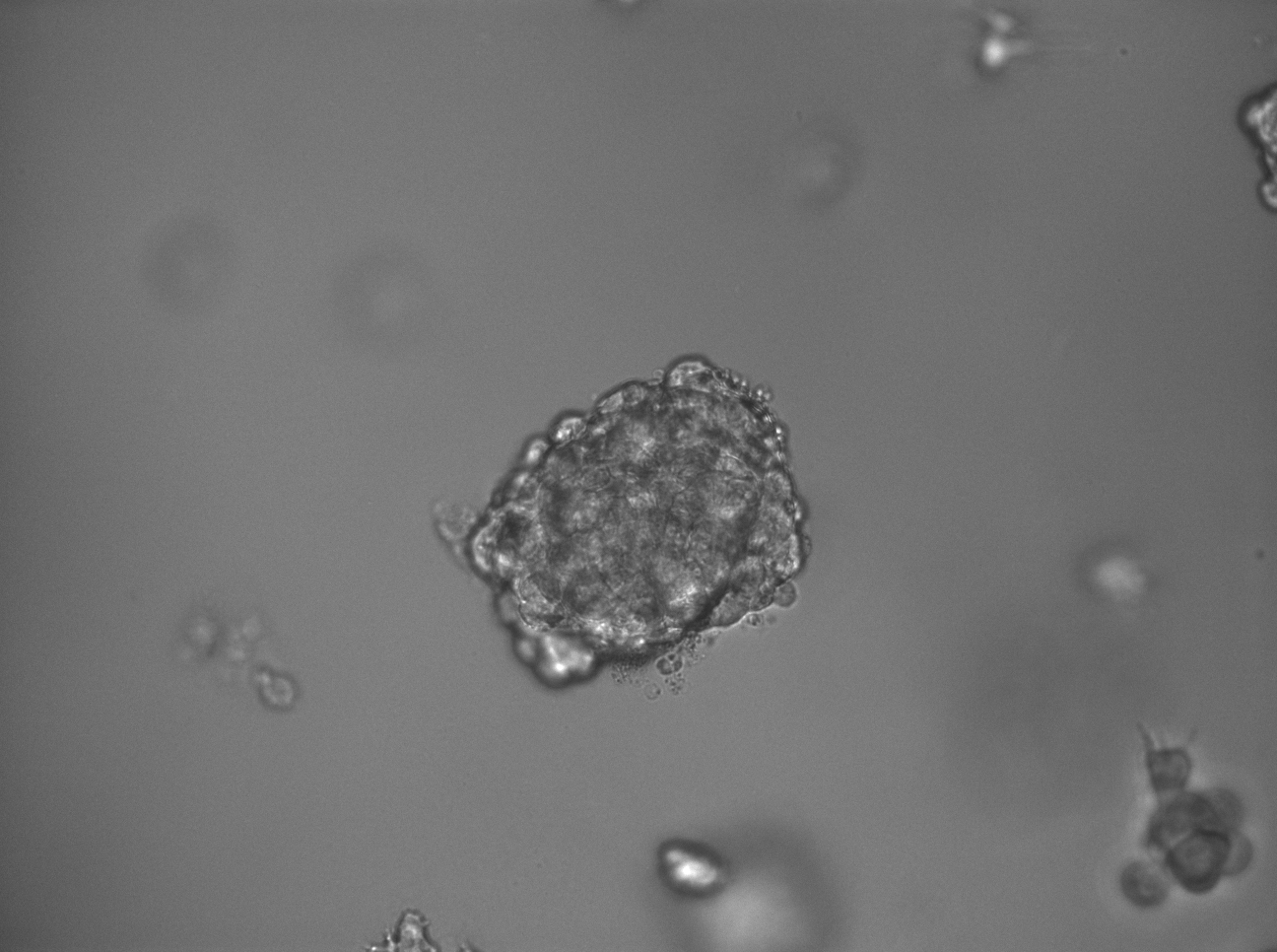

Supplement: Supplementary file 4 — Source Data Fig. 4 [file 41586_2026_10187_MOESM4_ESM.zip › HCEC1CT/HCEC1CT-KRAS_D10_Dox-00500_E03f_20x_ch00.jpg]

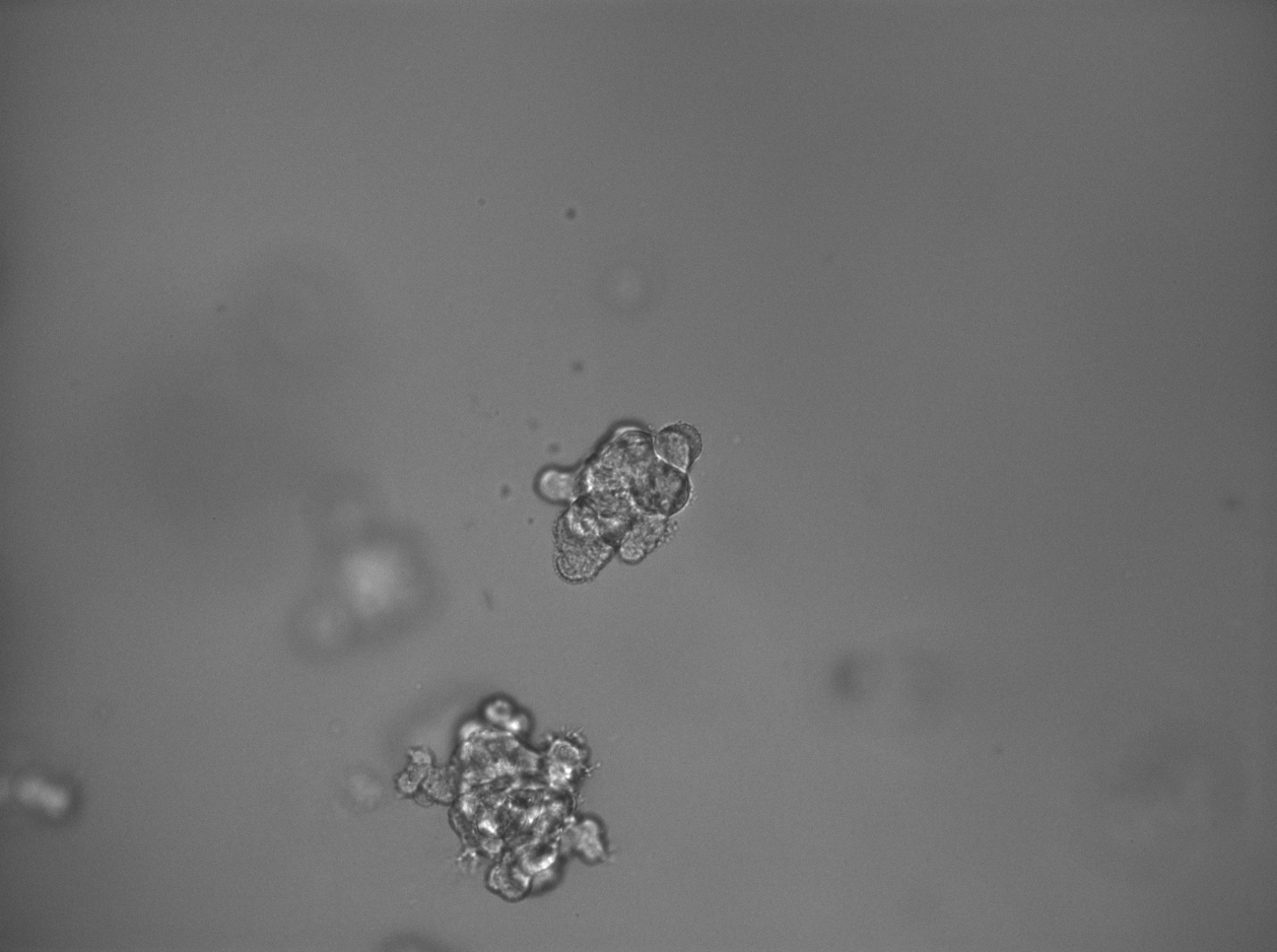

Supplement: Supplementary file 4 — Source Data Fig. 4 [file 41586_2026_10187_MOESM4_ESM.zip › HCEC1CT/HCEC1CT-KRAS_D10_Dox-00500_E03g_20x_ch00.jpg]

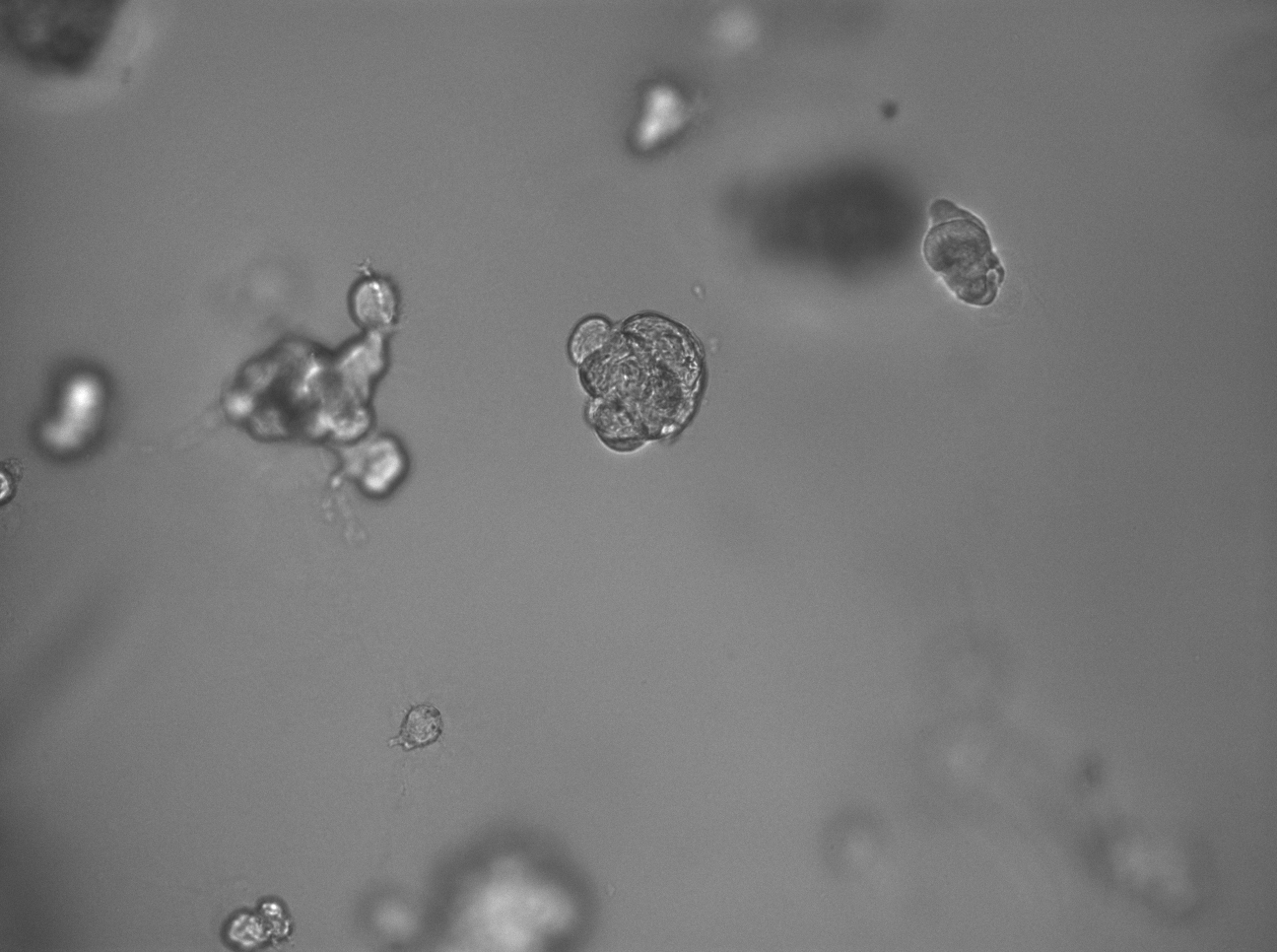

Supplement: Supplementary file 4 — Source Data Fig. 4 [file 41586_2026_10187_MOESM4_ESM.zip › HCEC1CT/HCEC1CT-KRAS_D10_Dox-01000_F01a_20x_ch00.jpg]

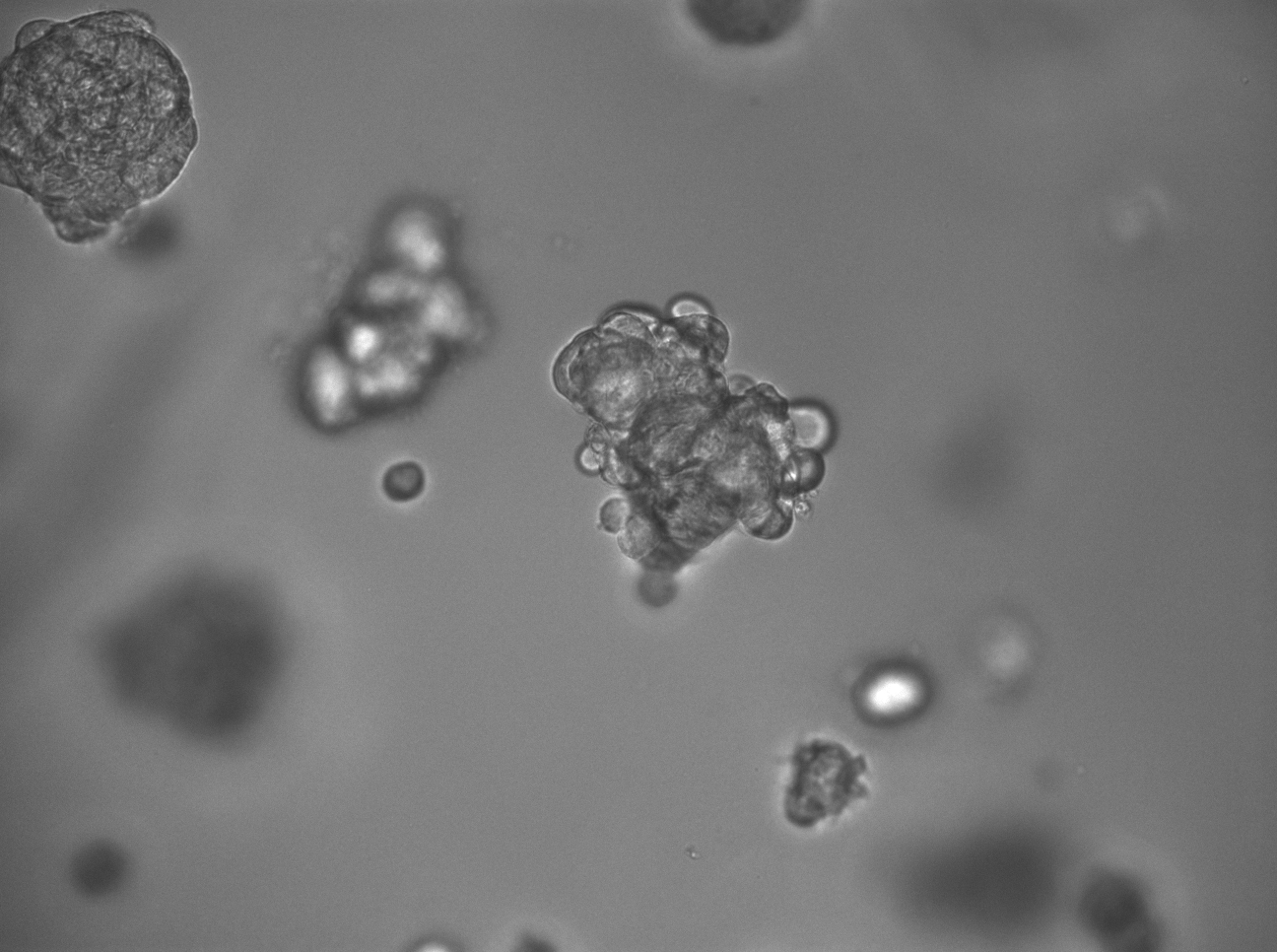

Supplement: Supplementary file 4 — Source Data Fig. 4 [file 41586_2026_10187_MOESM4_ESM.zip › HCEC1CT/HCEC1CT-KRAS_D10_Dox-01000_F01b_20x_ch00.jpg]

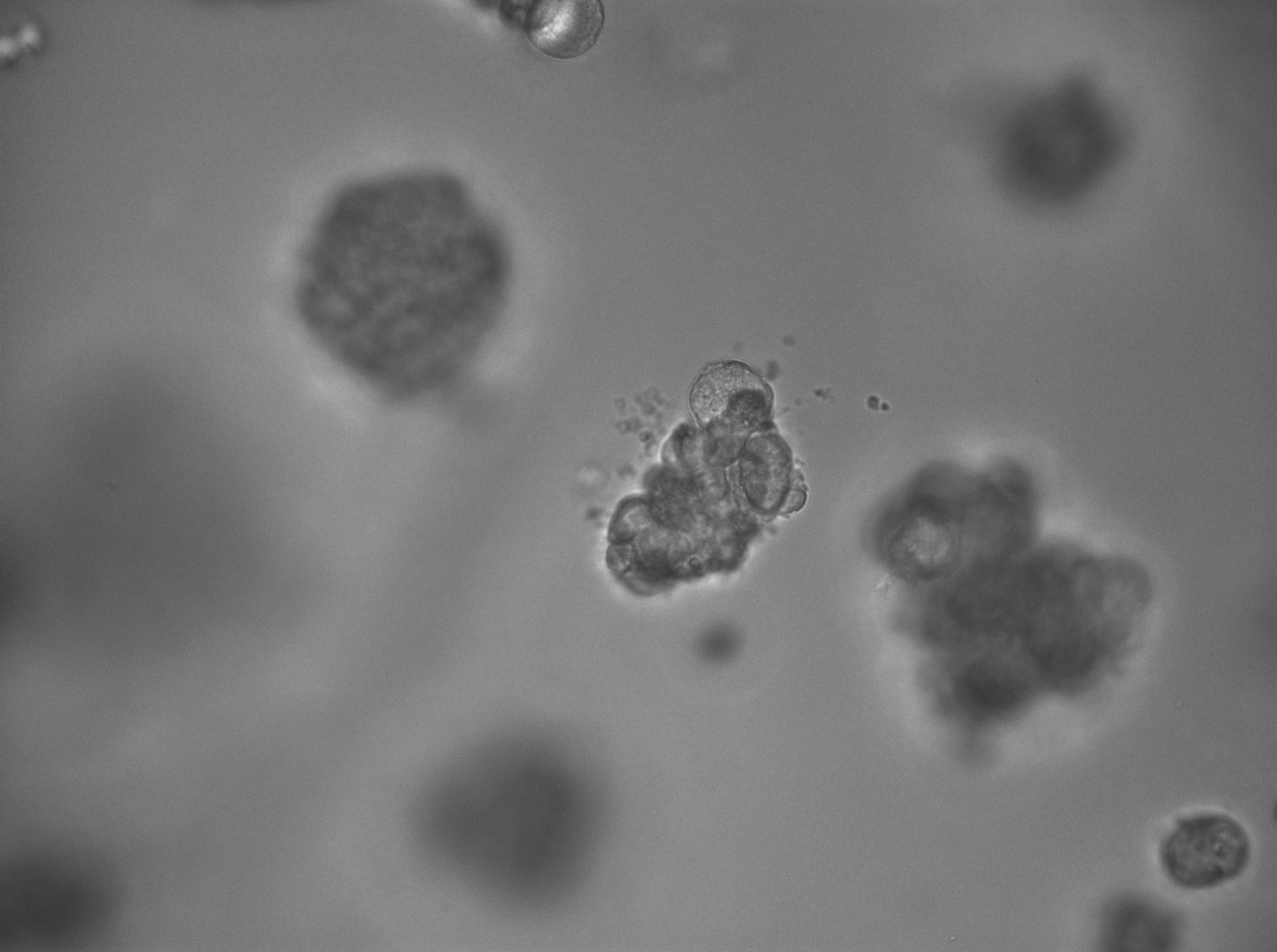

Supplement: Supplementary file 4 — Source Data Fig. 4 [file 41586_2026_10187_MOESM4_ESM.zip › HCEC1CT/HCEC1CT-KRAS_D10_Dox-01000_F01c_20x_ch00.jpg]

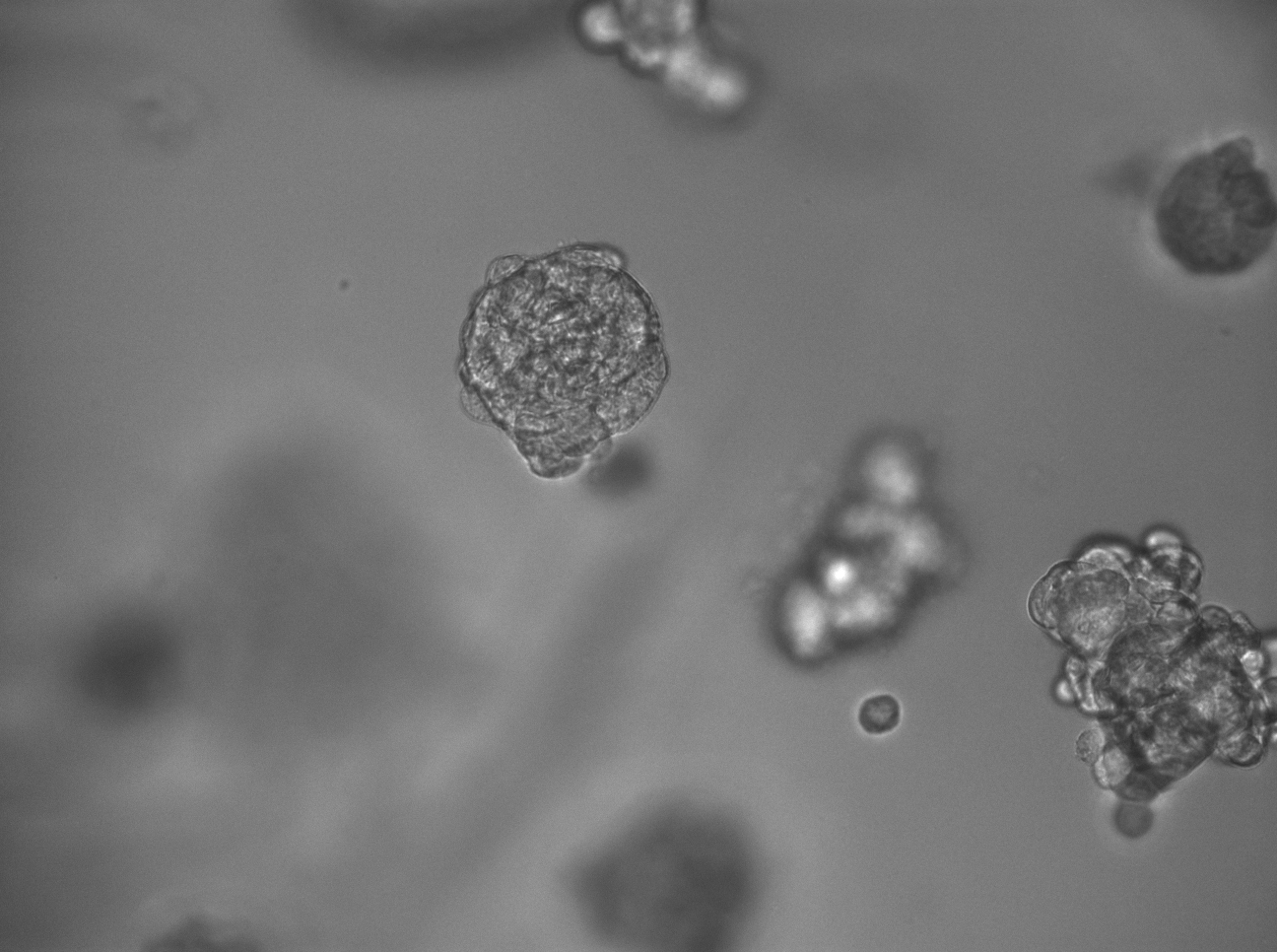

Supplement: Supplementary file 4 — Source Data Fig. 4 [file 41586_2026_10187_MOESM4_ESM.zip › HCEC1CT/HCEC1CT-KRAS_D10_Dox-01000_F01d_20x_ch00.jpg]

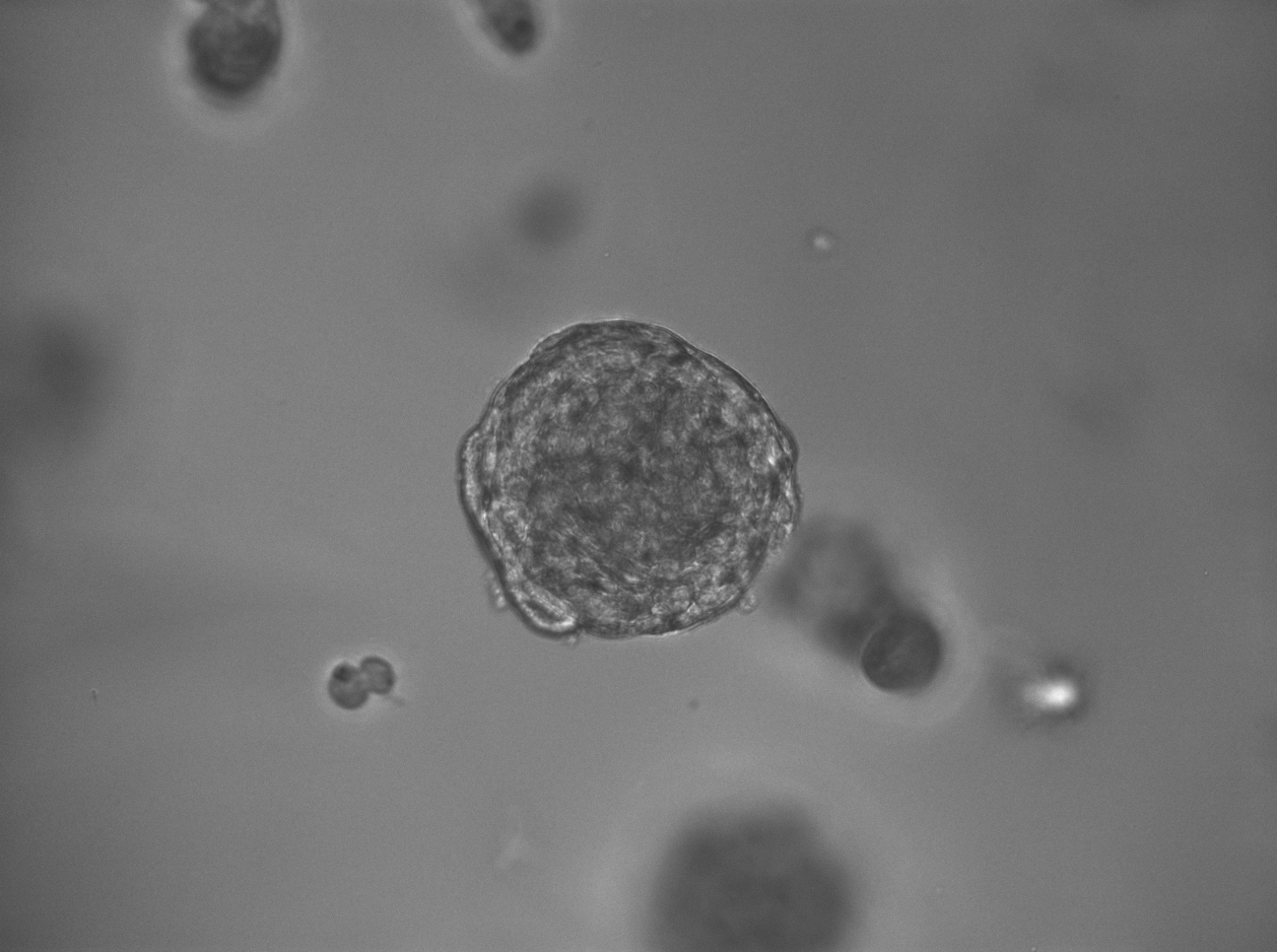

Supplement: Supplementary file 4 — Source Data Fig. 4 [file 41586_2026_10187_MOESM4_ESM.zip › HCEC1CT/HCEC1CT-KRAS_D10_Dox-01000_F01e_20x_ch00.jpg]

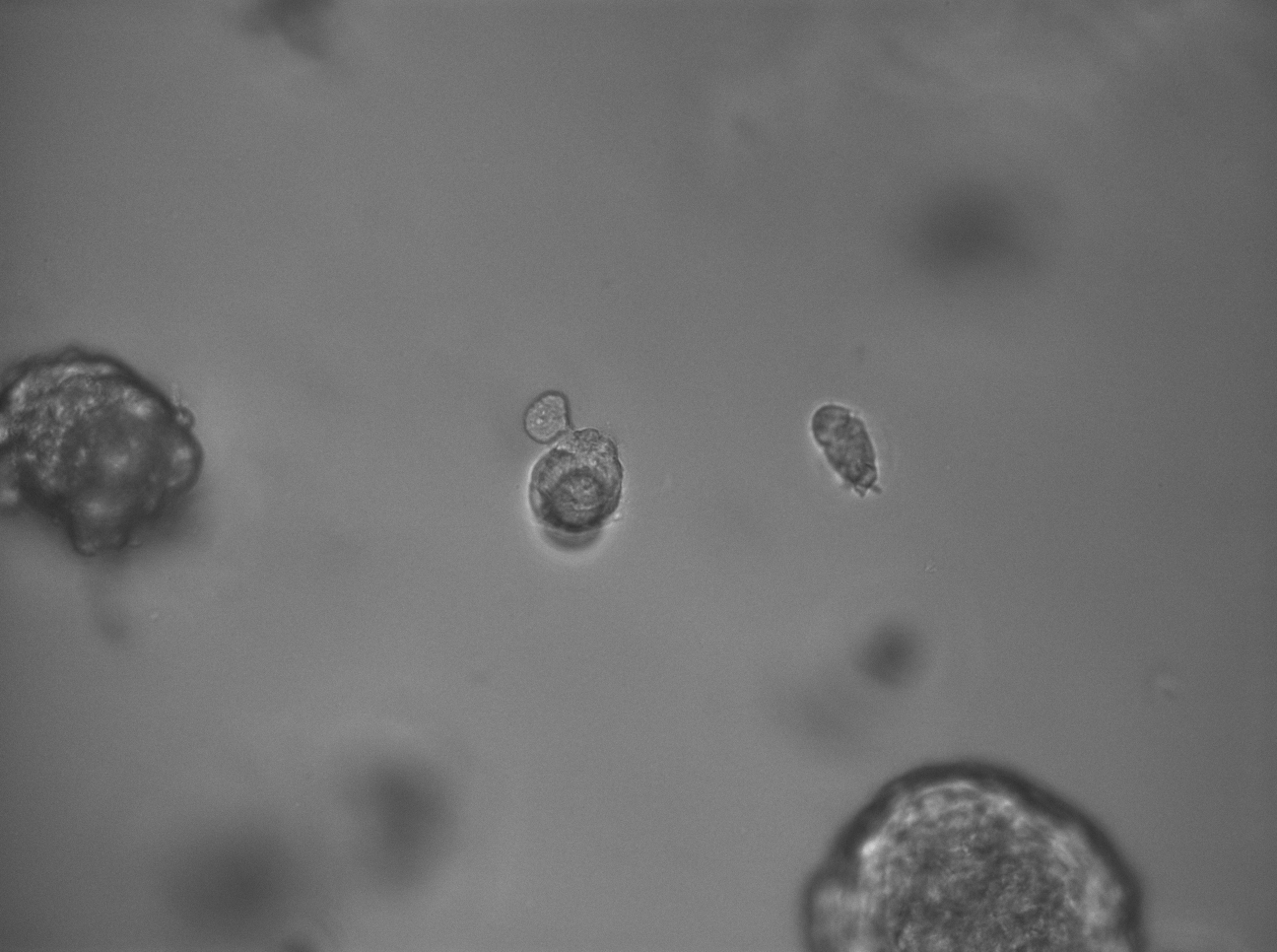

Supplement: Supplementary file 4 — Source Data Fig. 4 [file 41586_2026_10187_MOESM4_ESM.zip › HCEC1CT/HCEC1CT-KRAS_D10_Dox-01000_F01f_20x_ch00.jpg]

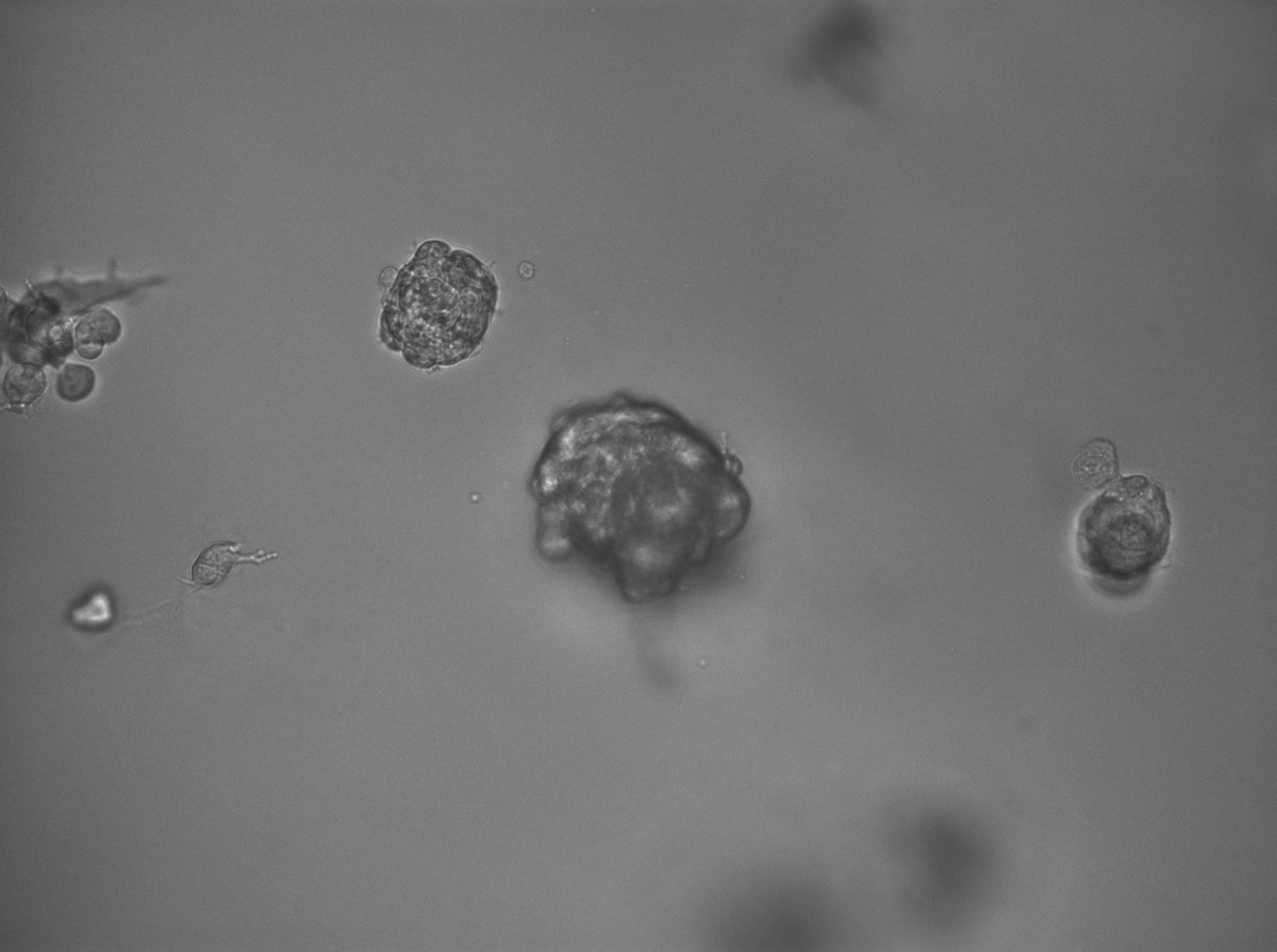

Supplement: Supplementary file 4 — Source Data Fig. 4 [file 41586_2026_10187_MOESM4_ESM.zip › HCEC1CT/HCEC1CT-KRAS_D10_Dox-01000_F01g_20x_ch00.jpg]

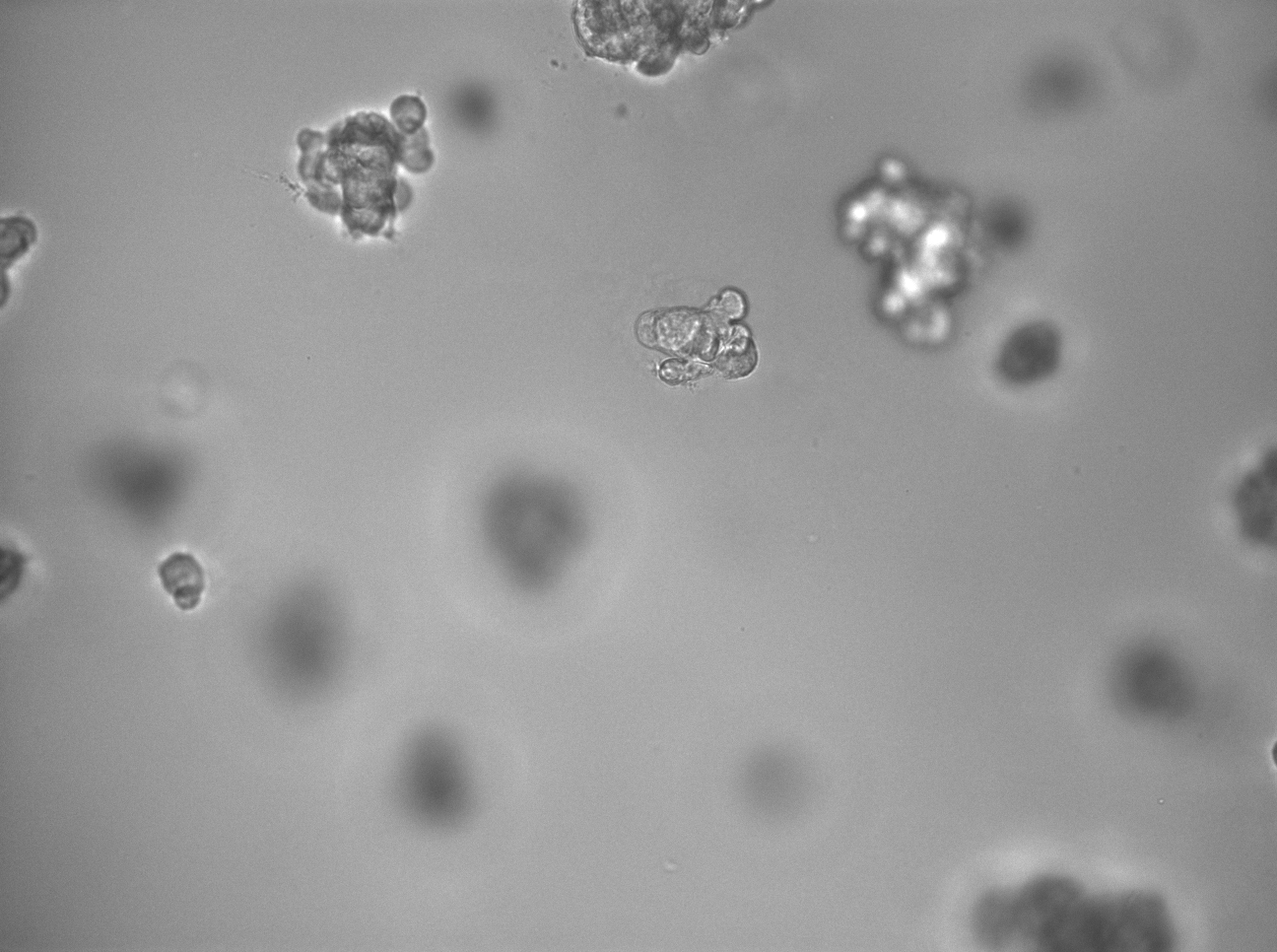

Supplement: Supplementary file 4 — Source Data Fig. 4 [file 41586_2026_10187_MOESM4_ESM.zip › HCEC1CT/HCEC1CT-KRAS_D10_Dox-01000_F02a_20x_ch00.jpg]

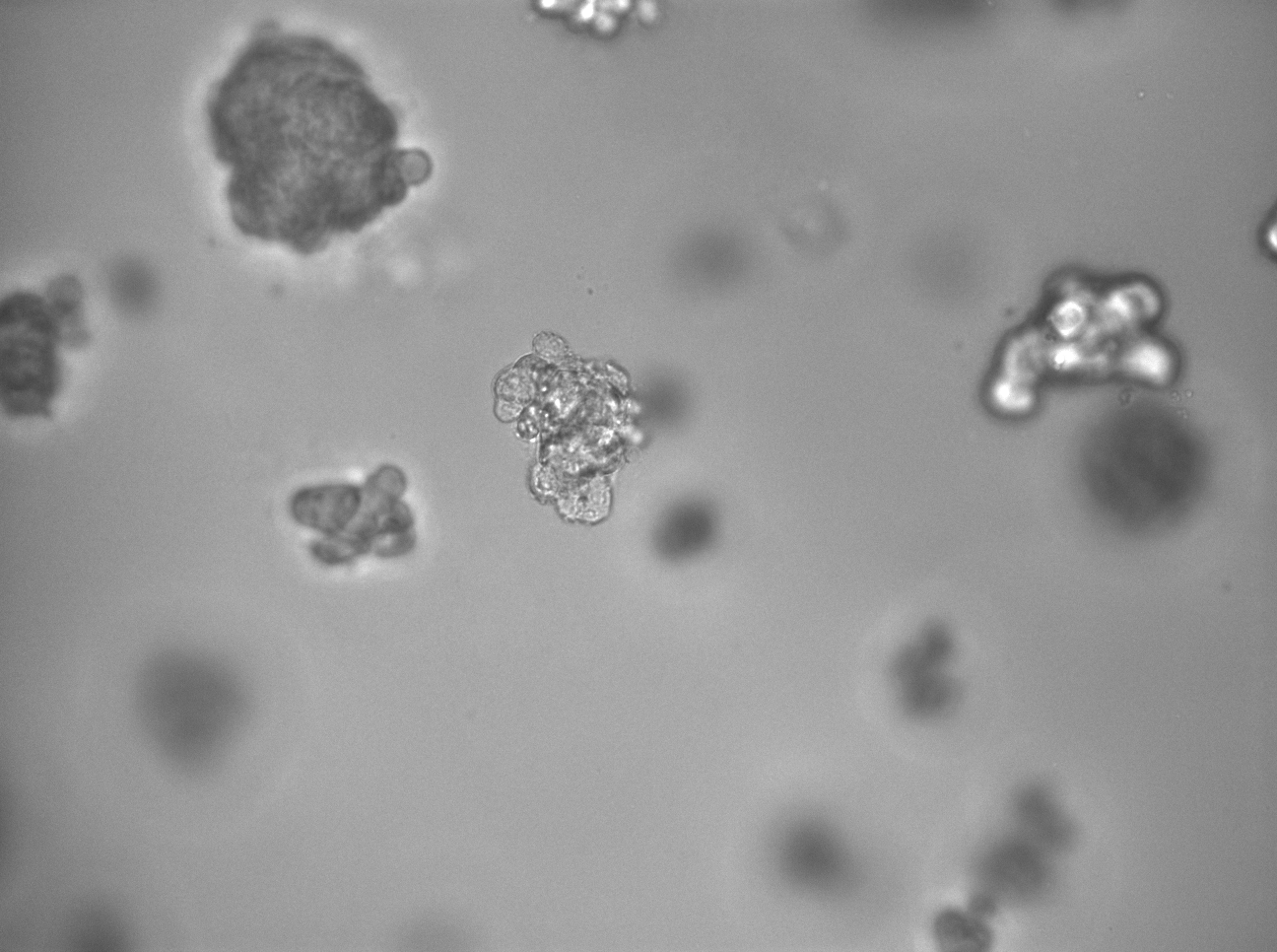

Supplement: Supplementary file 4 — Source Data Fig. 4 [file 41586_2026_10187_MOESM4_ESM.zip › HCEC1CT/HCEC1CT-KRAS_D10_Dox-01000_F02b_20x_ch00.jpg]

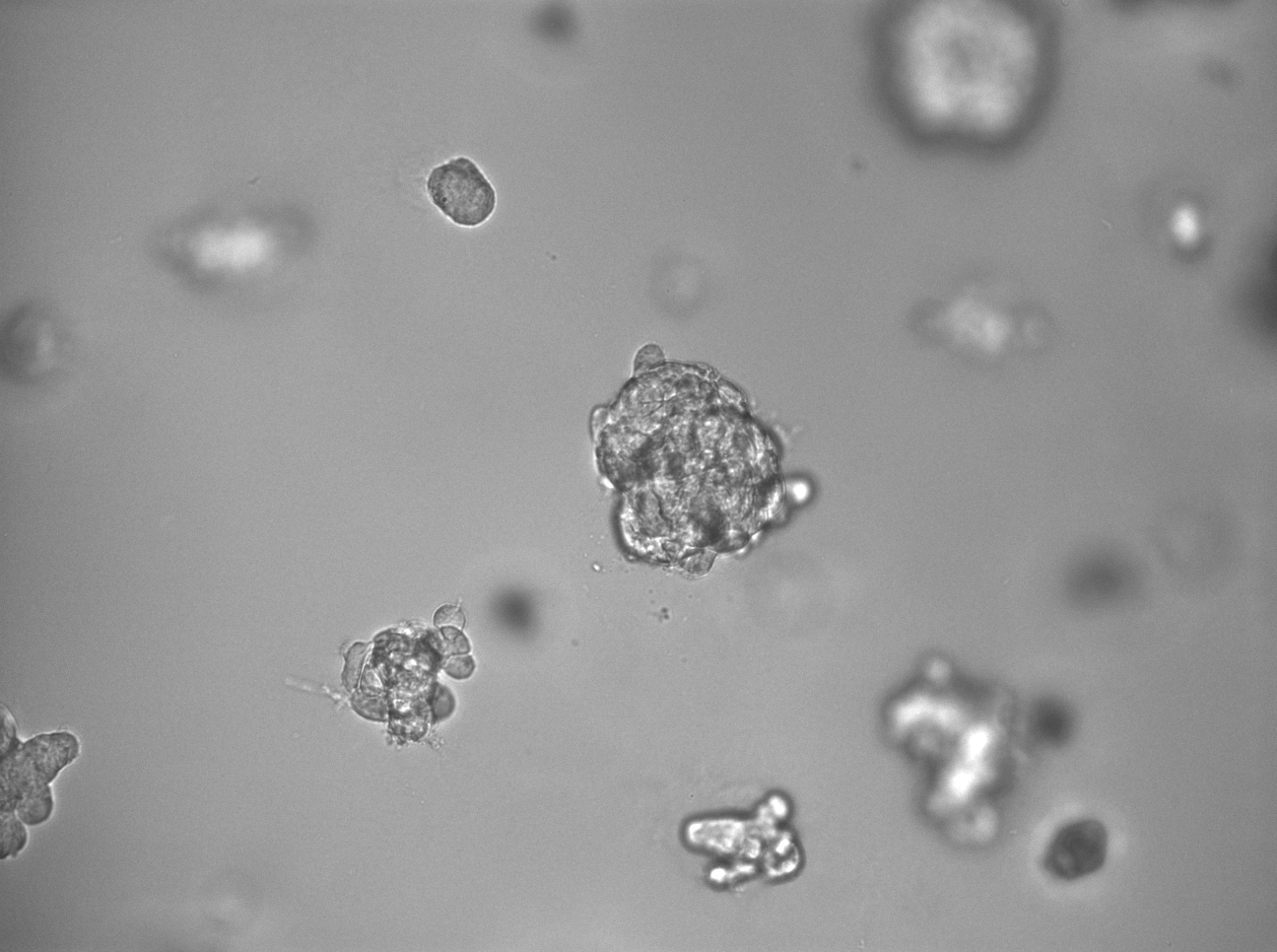

Supplement: Supplementary file 4 — Source Data Fig. 4 [file 41586_2026_10187_MOESM4_ESM.zip › HCEC1CT/HCEC1CT-KRAS_D10_Dox-01000_F02c_20x_ch00.jpg]

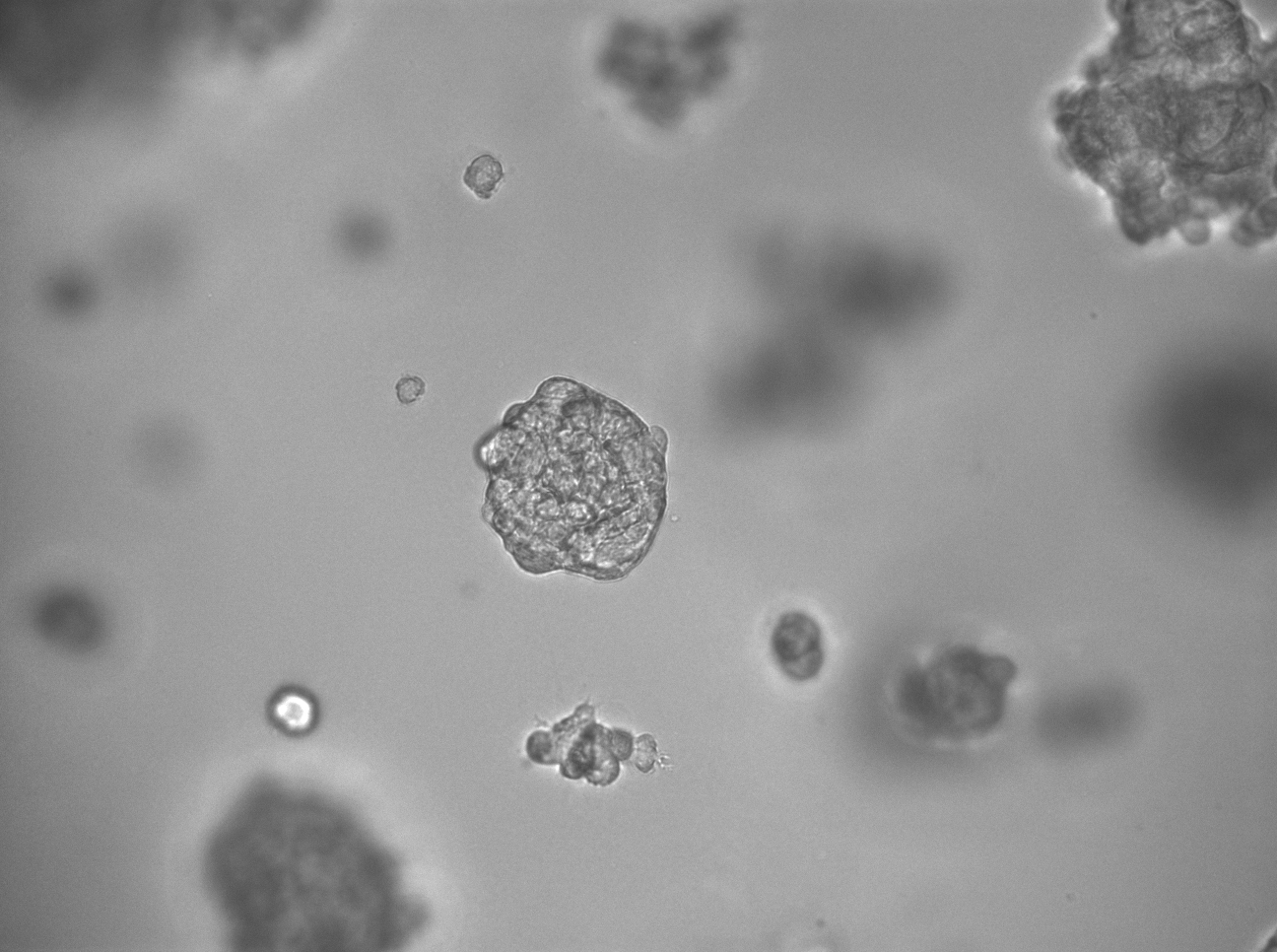

Supplement: Supplementary file 4 — Source Data Fig. 4 [file 41586_2026_10187_MOESM4_ESM.zip › HCEC1CT/HCEC1CT-KRAS_D10_Dox-01000_F02d_20x_ch00.jpg]

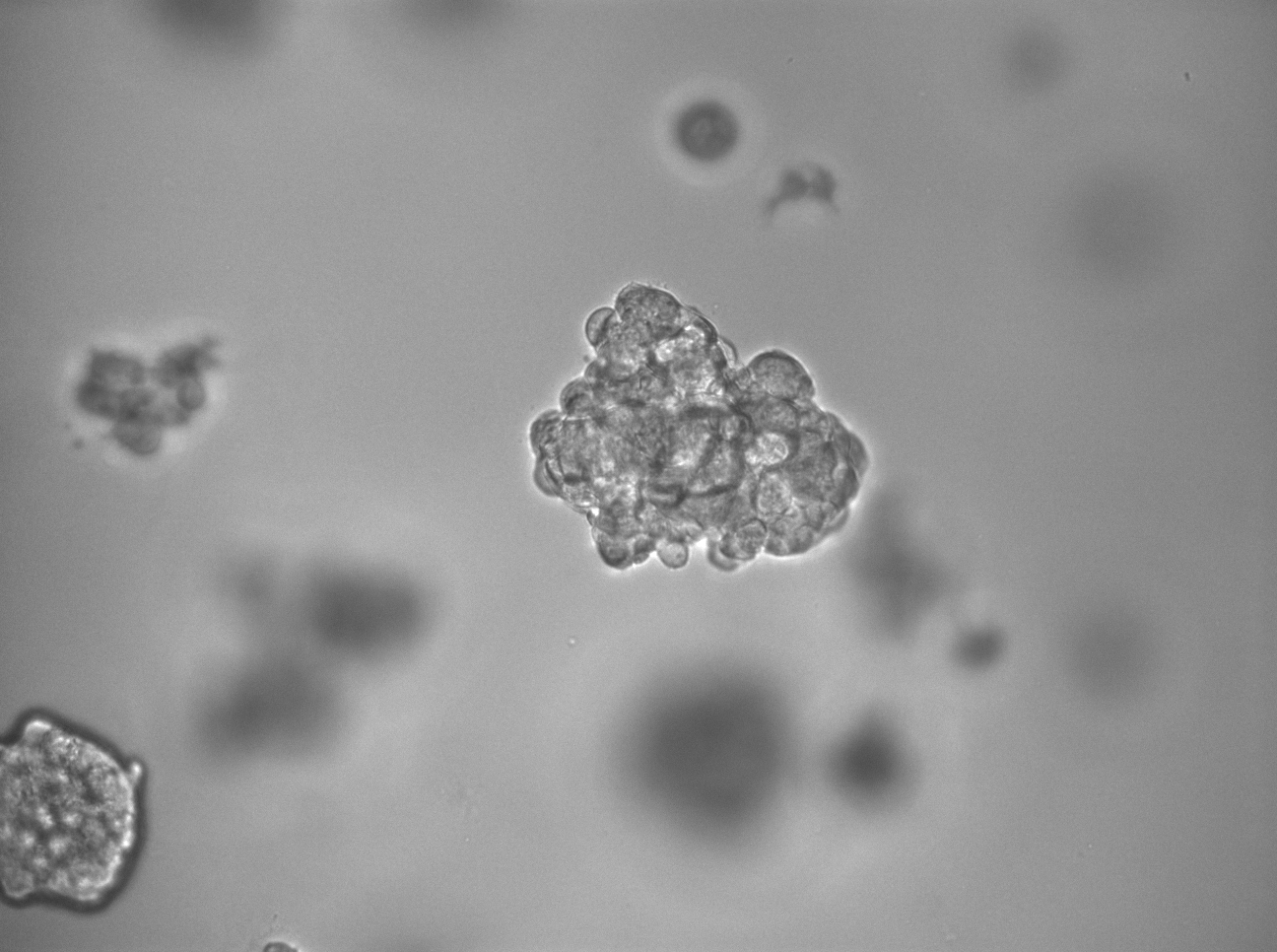

Supplement: Supplementary file 4 — Source Data Fig. 4 [file 41586_2026_10187_MOESM4_ESM.zip › HCEC1CT/HCEC1CT-KRAS_D10_Dox-01000_F02e_20x_ch00.jpg]

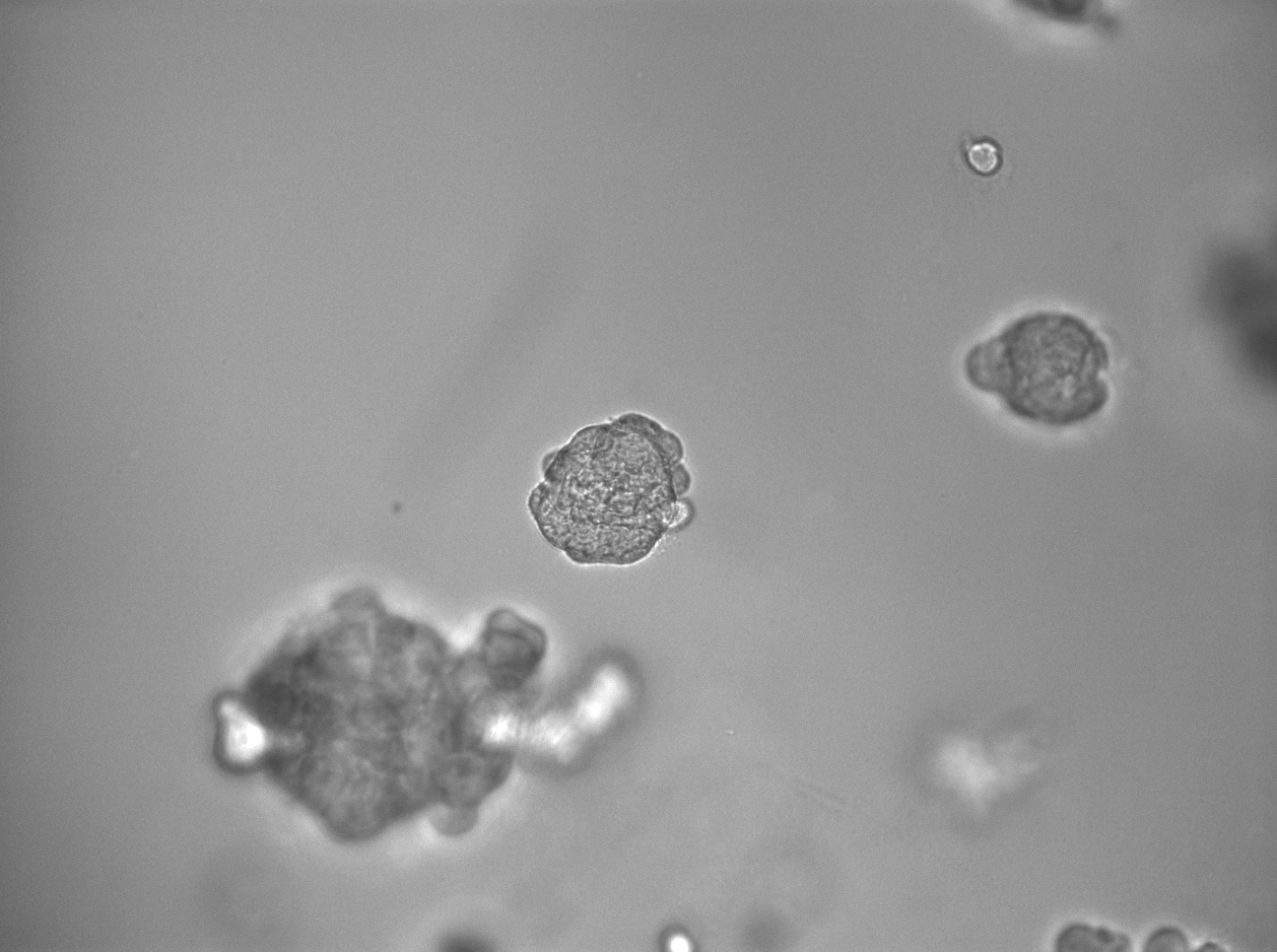

Supplement: Supplementary file 4 — Source Data Fig. 4 [file 41586_2026_10187_MOESM4_ESM.zip › HCEC1CT/HCEC1CT-KRAS_D10_Dox-01000_F02f_20x_ch00.jpg]

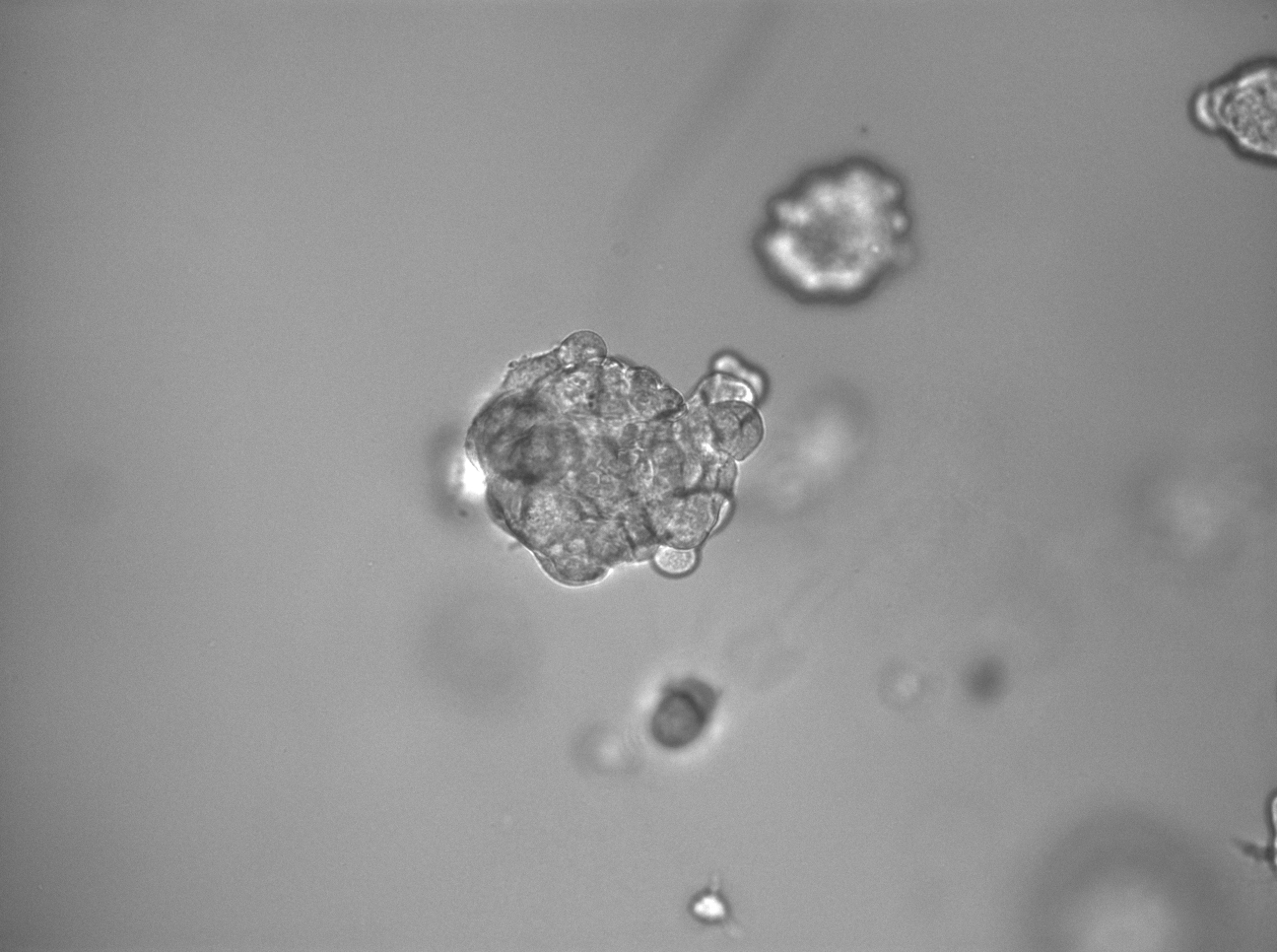

Supplement: Supplementary file 4 — Source Data Fig. 4 [file 41586_2026_10187_MOESM4_ESM.zip › HCEC1CT/HCEC1CT-KRAS_D10_Dox-01000_F02g_20x_ch00.jpg]

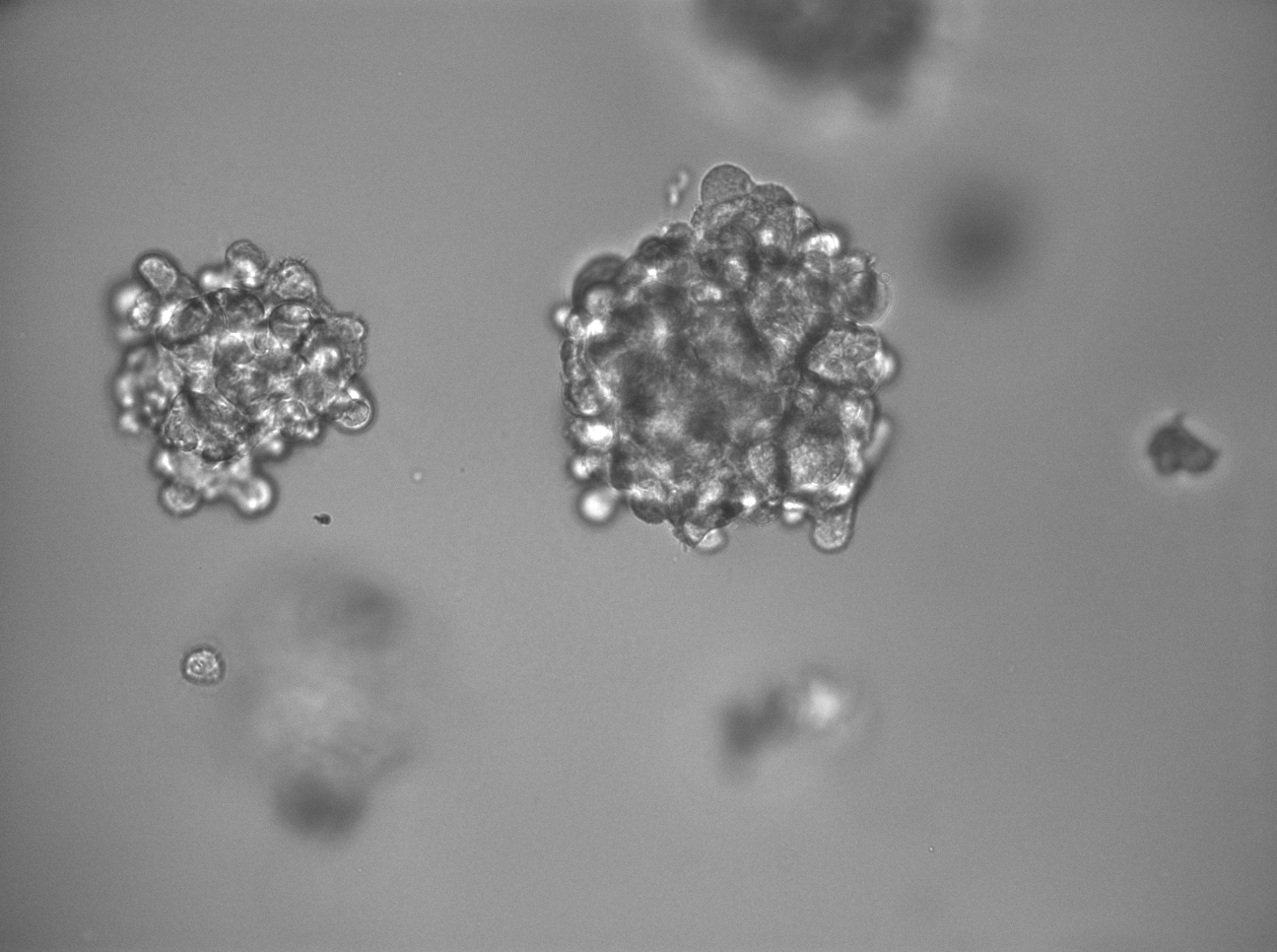

Supplement: Supplementary file 4 — Source Data Fig. 4 [file 41586_2026_10187_MOESM4_ESM.zip › HCEC1CT/HCEC1CT-KRAS_D10_Dox-01000_F03a_20x_ch00.jpg]

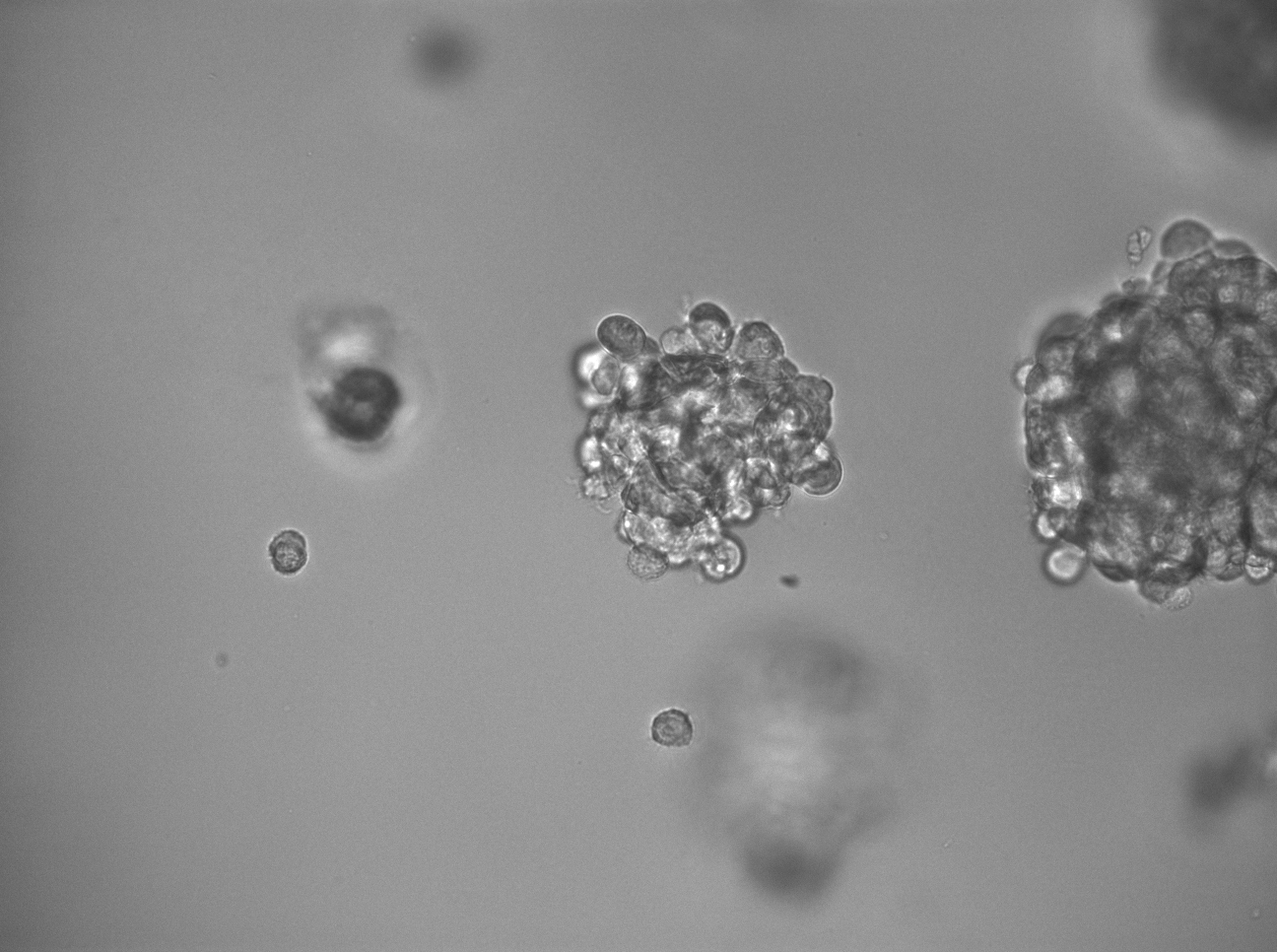

Supplement: Supplementary file 4 — Source Data Fig. 4 [file 41586_2026_10187_MOESM4_ESM.zip › HCEC1CT/HCEC1CT-KRAS_D10_Dox-01000_F03b_20x_ch00.jpg]

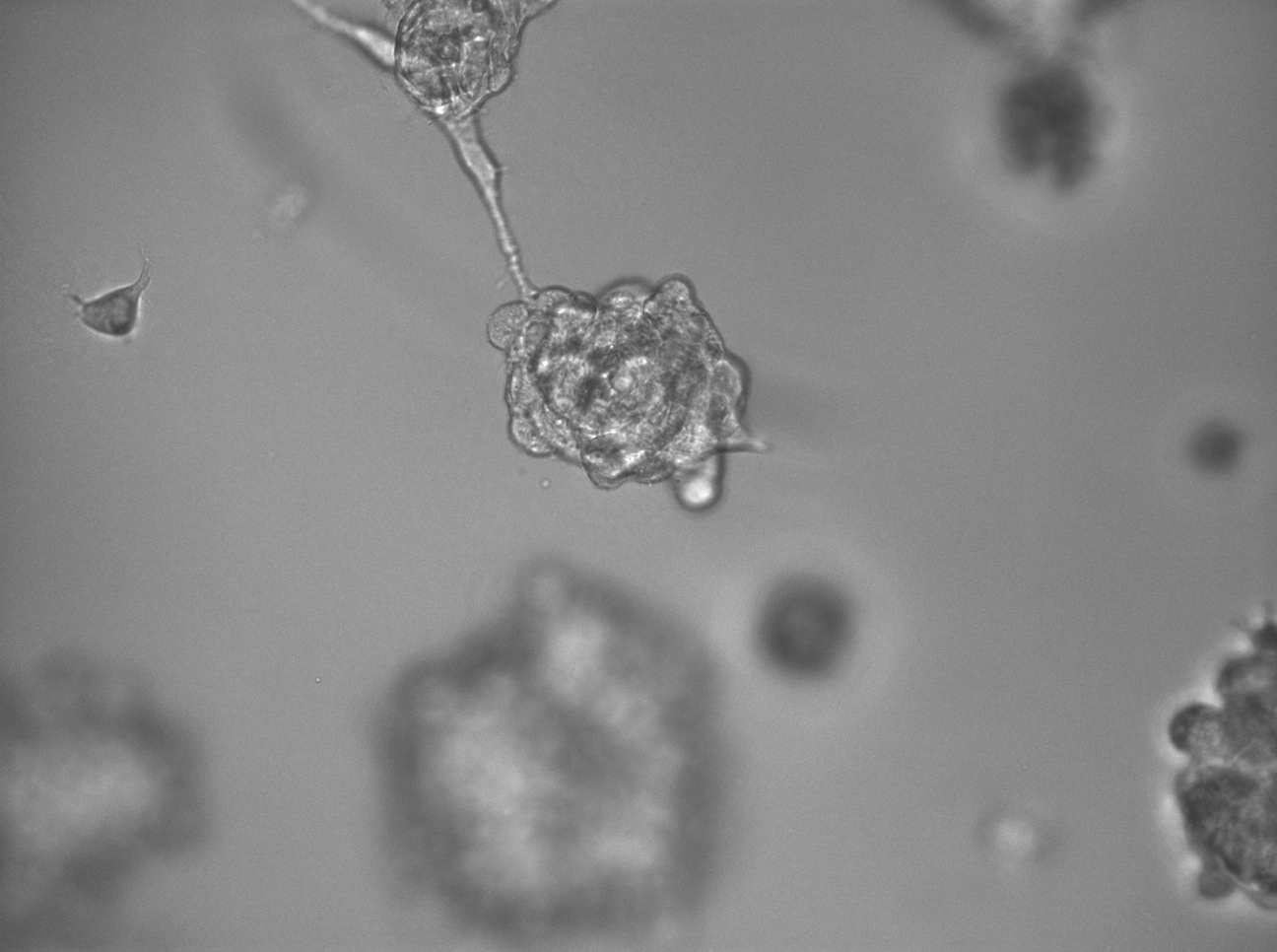

Supplement: Supplementary file 4 — Source Data Fig. 4 [file 41586_2026_10187_MOESM4_ESM.zip › HCEC1CT/HCEC1CT-KRAS_D10_Dox-01000_F03c_20x_ch00.jpg]

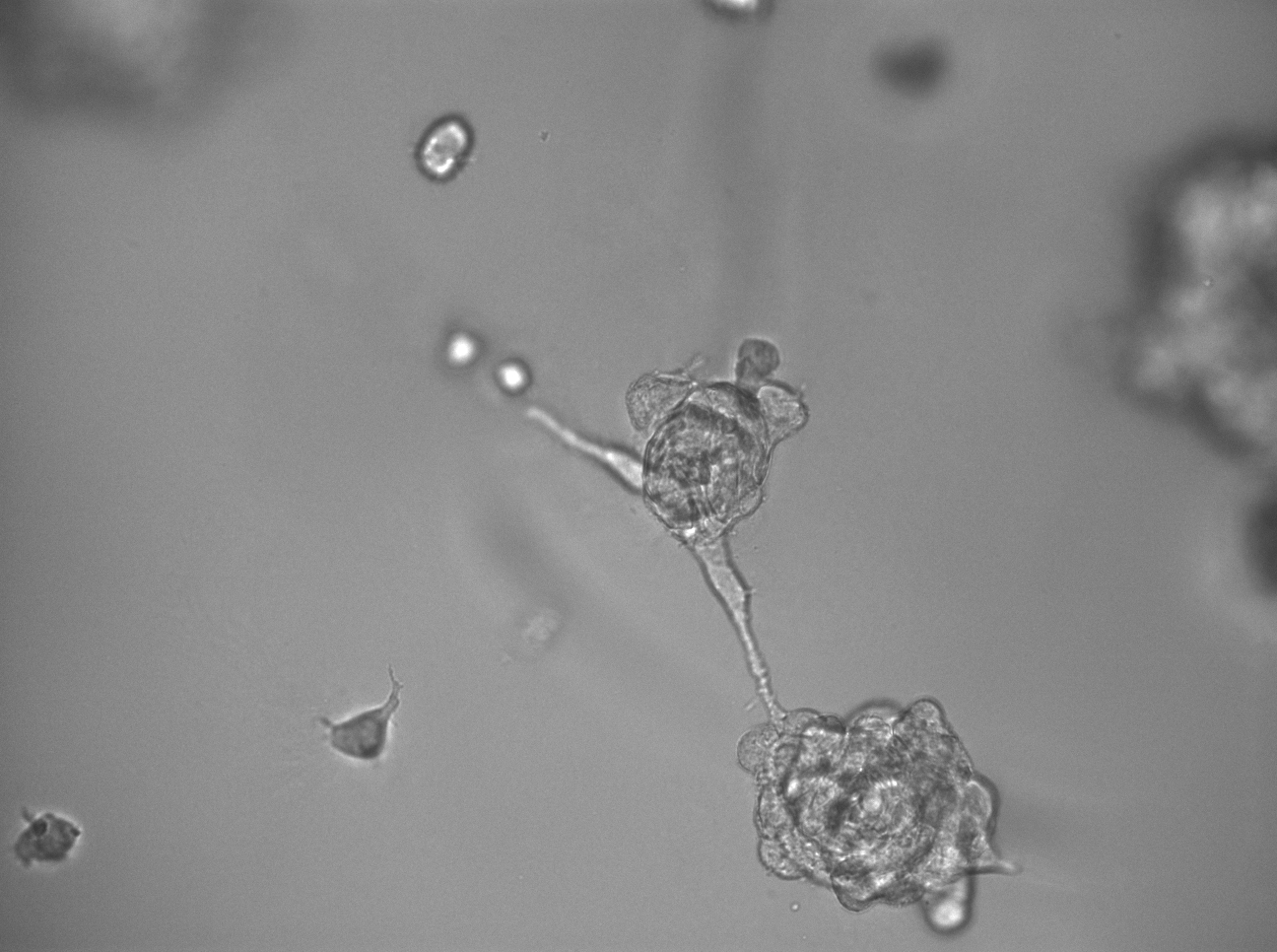

Supplement: Supplementary file 4 — Source Data Fig. 4 [file 41586_2026_10187_MOESM4_ESM.zip › HCEC1CT/HCEC1CT-KRAS_D10_Dox-01000_F03d_20x_ch00.jpg]

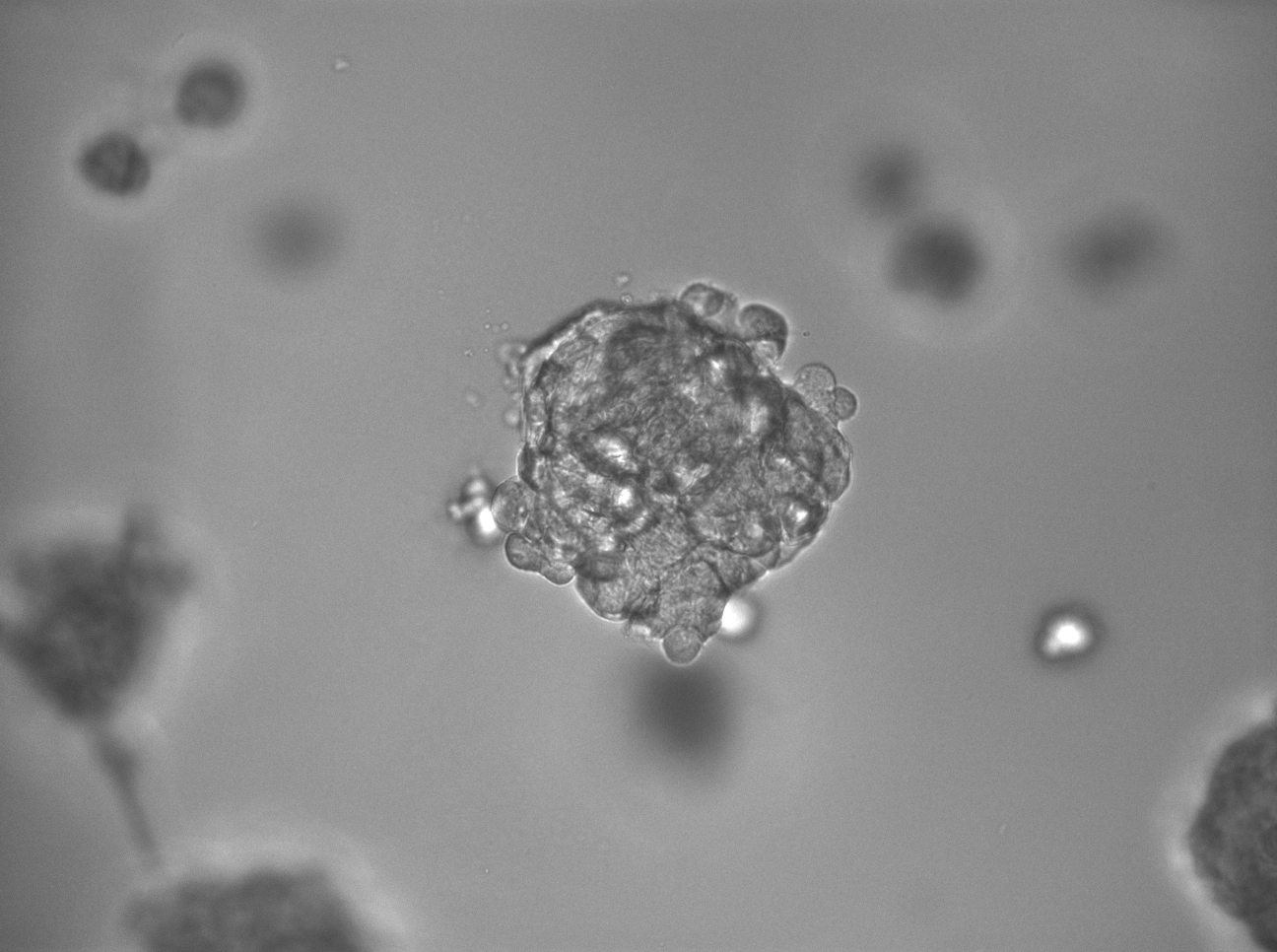

Supplement: Supplementary file 4 — Source Data Fig. 4 [file 41586_2026_10187_MOESM4_ESM.zip › HCEC1CT/HCEC1CT-KRAS_D10_Dox-01000_F03e20x_ch00.jpg]

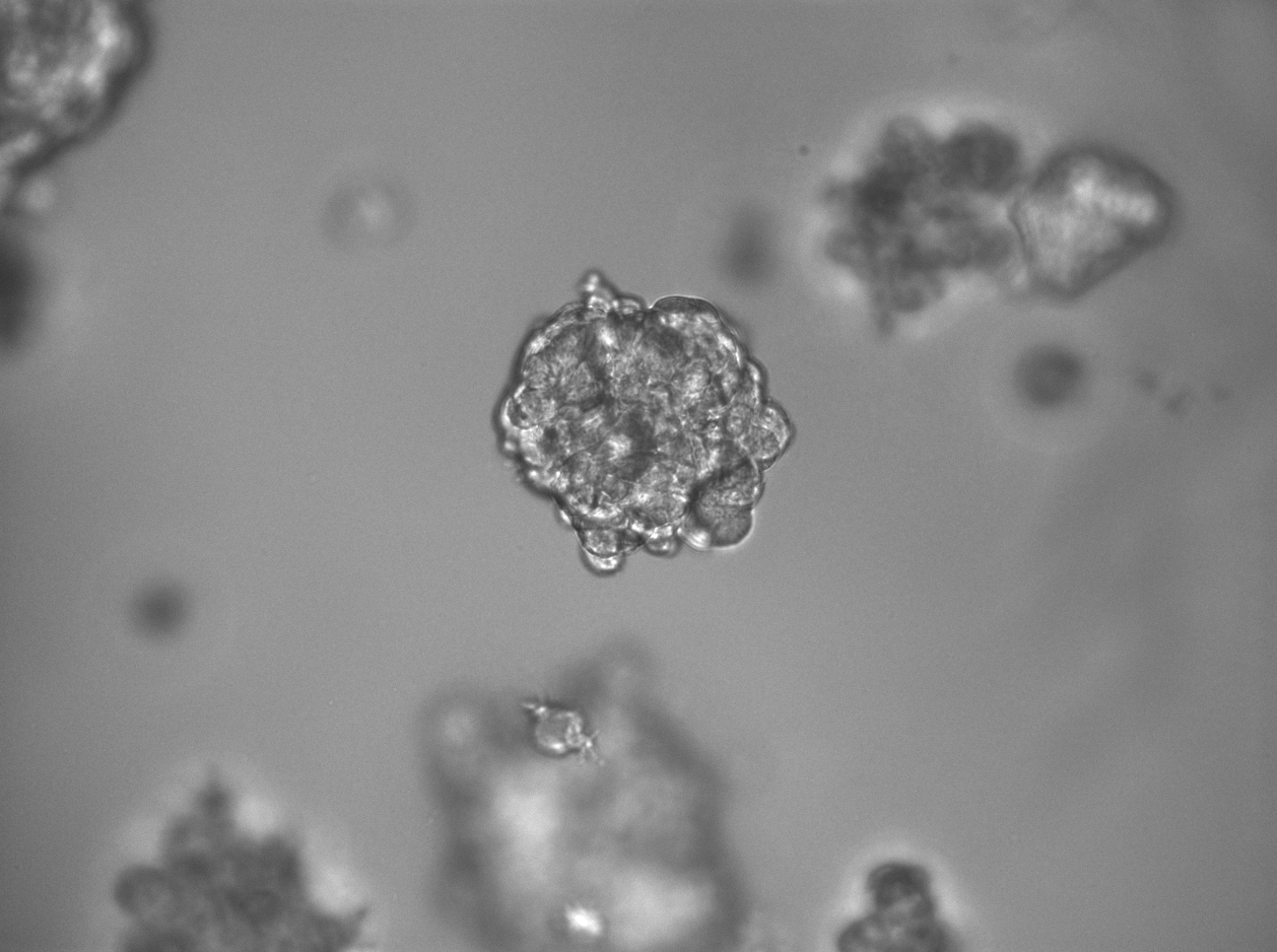

Supplement: Supplementary file 4 — Source Data Fig. 4 [file 41586_2026_10187_MOESM4_ESM.zip › HCEC1CT/HCEC1CT-KRAS_D10_Dox-01000_F03f_20x_ch00.jpg]

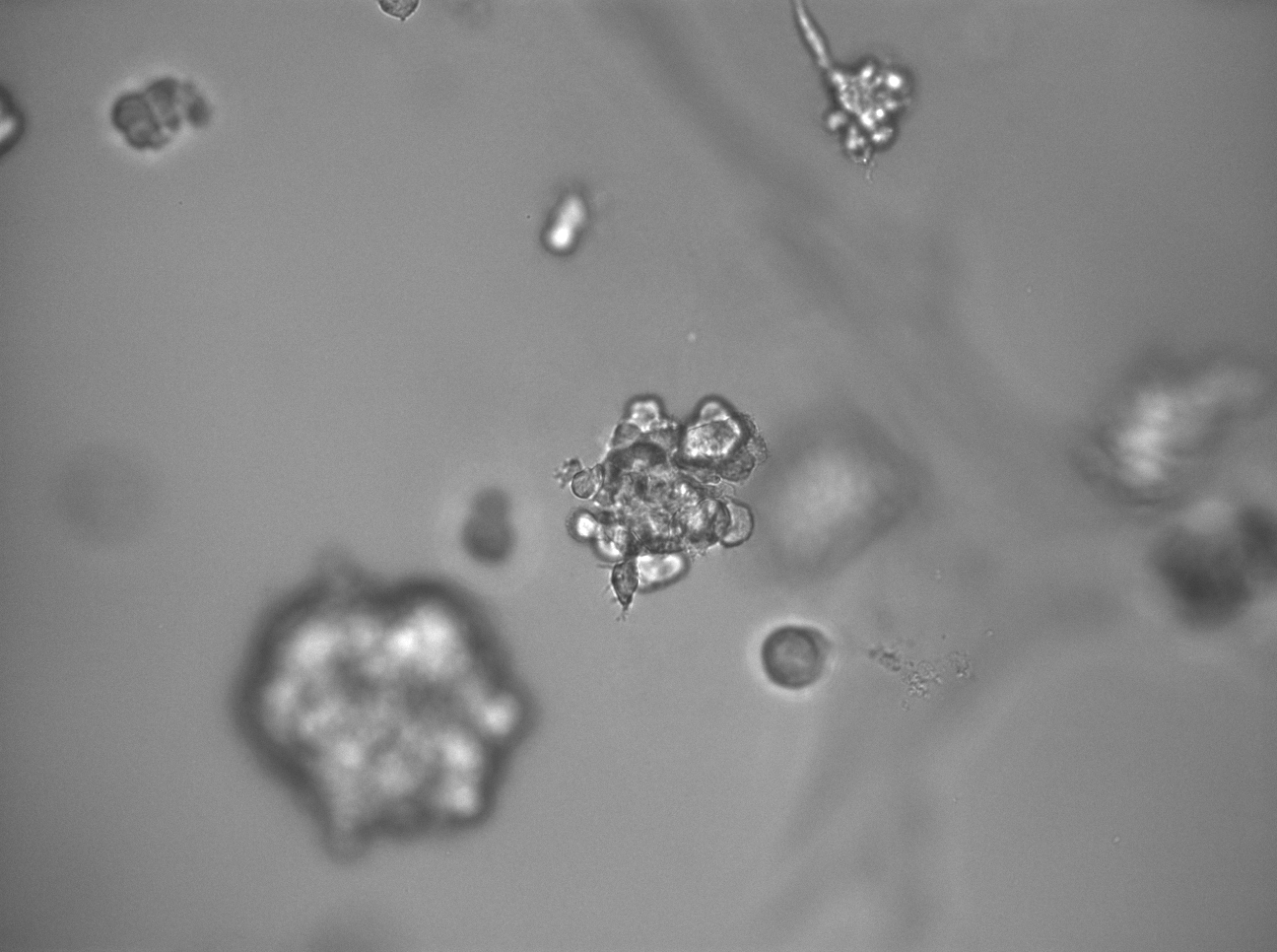

Supplement: Supplementary file 4 — Source Data Fig. 4 [file 41586_2026_10187_MOESM4_ESM.zip › HCEC1CT/HCEC1CT-KRAS_D10_Dox-01000_F03g_20x_ch00.jpg]

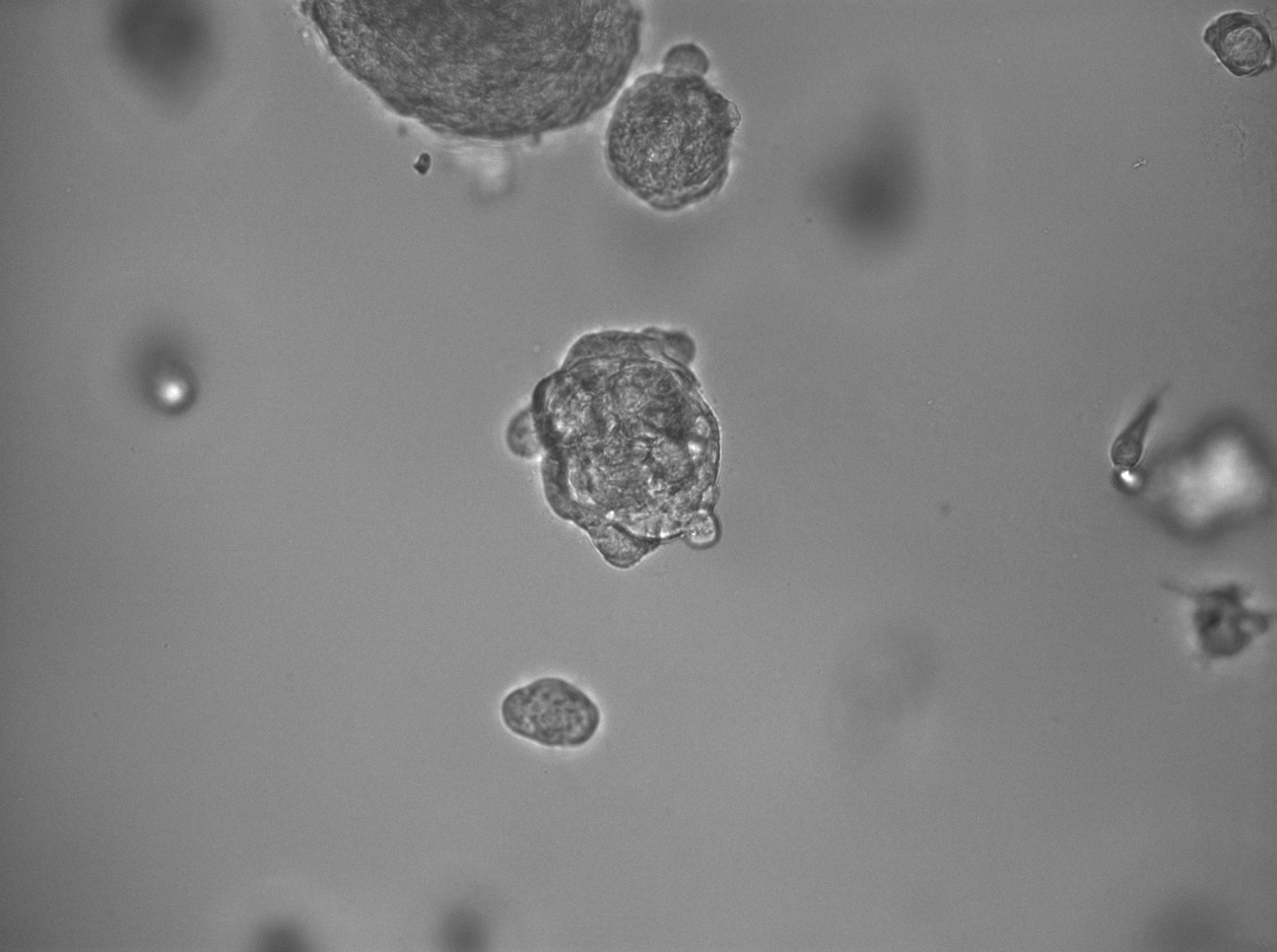

Supplement: Supplementary file 4 — Source Data Fig. 4 [file 41586_2026_10187_MOESM4_ESM.zip › HCEC1CT/HCEC1CT-KRAS_D10_Dox-02000_A01a_20x_ch00.jpg]

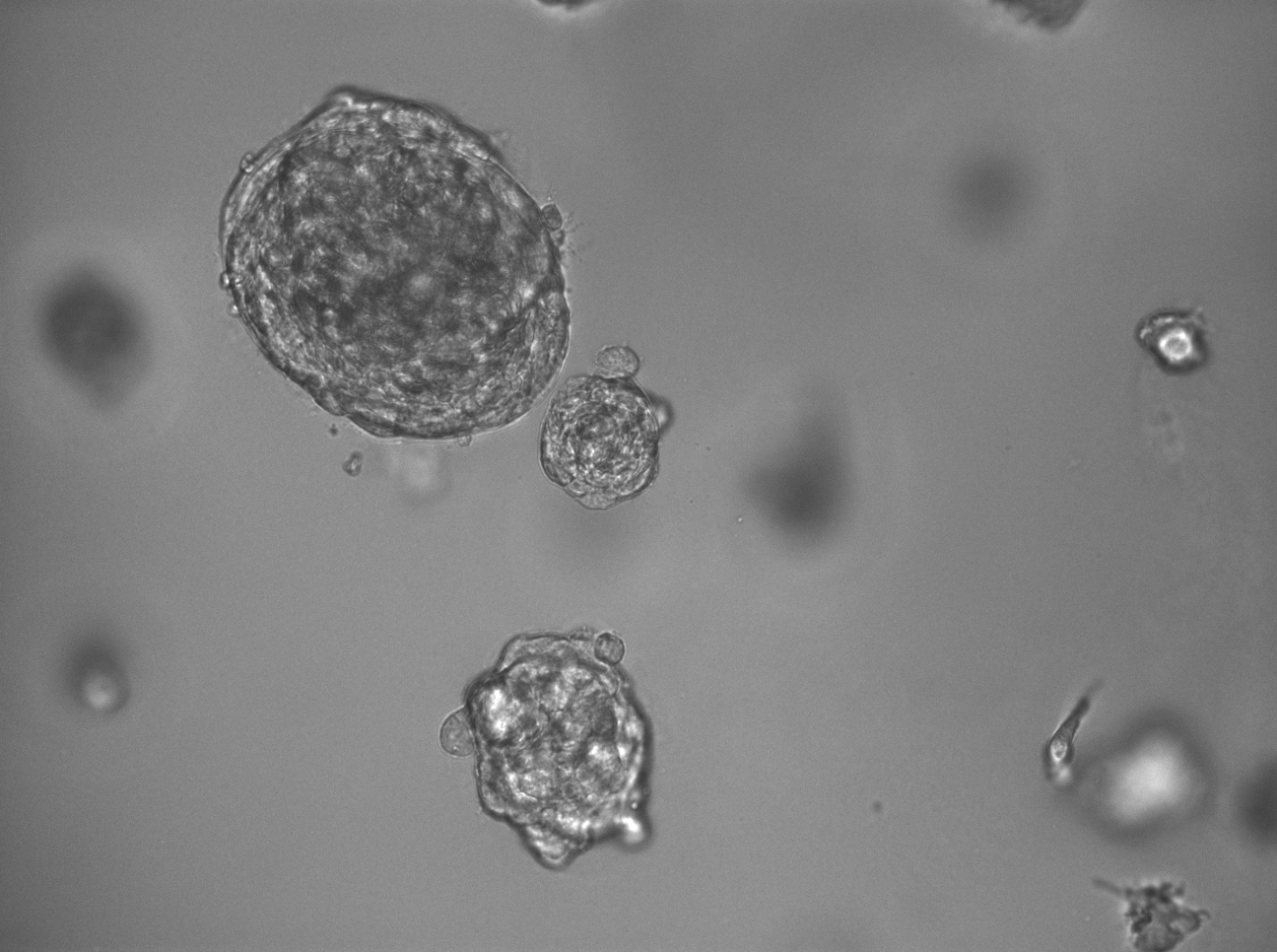

Supplement: Supplementary file 4 — Source Data Fig. 4 [file 41586_2026_10187_MOESM4_ESM.zip › HCEC1CT/HCEC1CT-KRAS_D10_Dox-02000_A01b_20x_ch00.jpg]

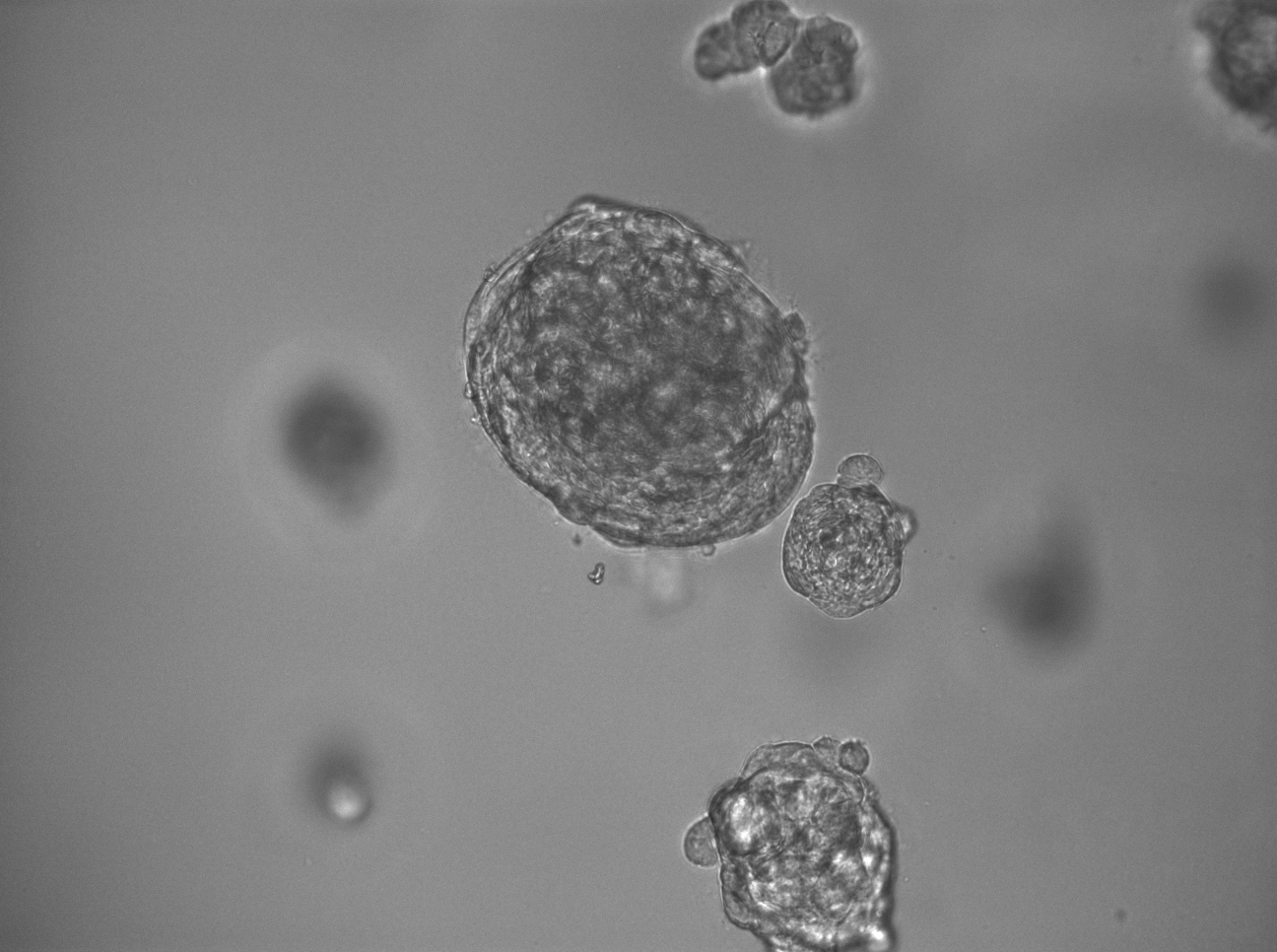

Supplement: Supplementary file 4 — Source Data Fig. 4 [file 41586_2026_10187_MOESM4_ESM.zip › HCEC1CT/HCEC1CT-KRAS_D10_Dox-02000_A01c_20x_ch00.jpg]

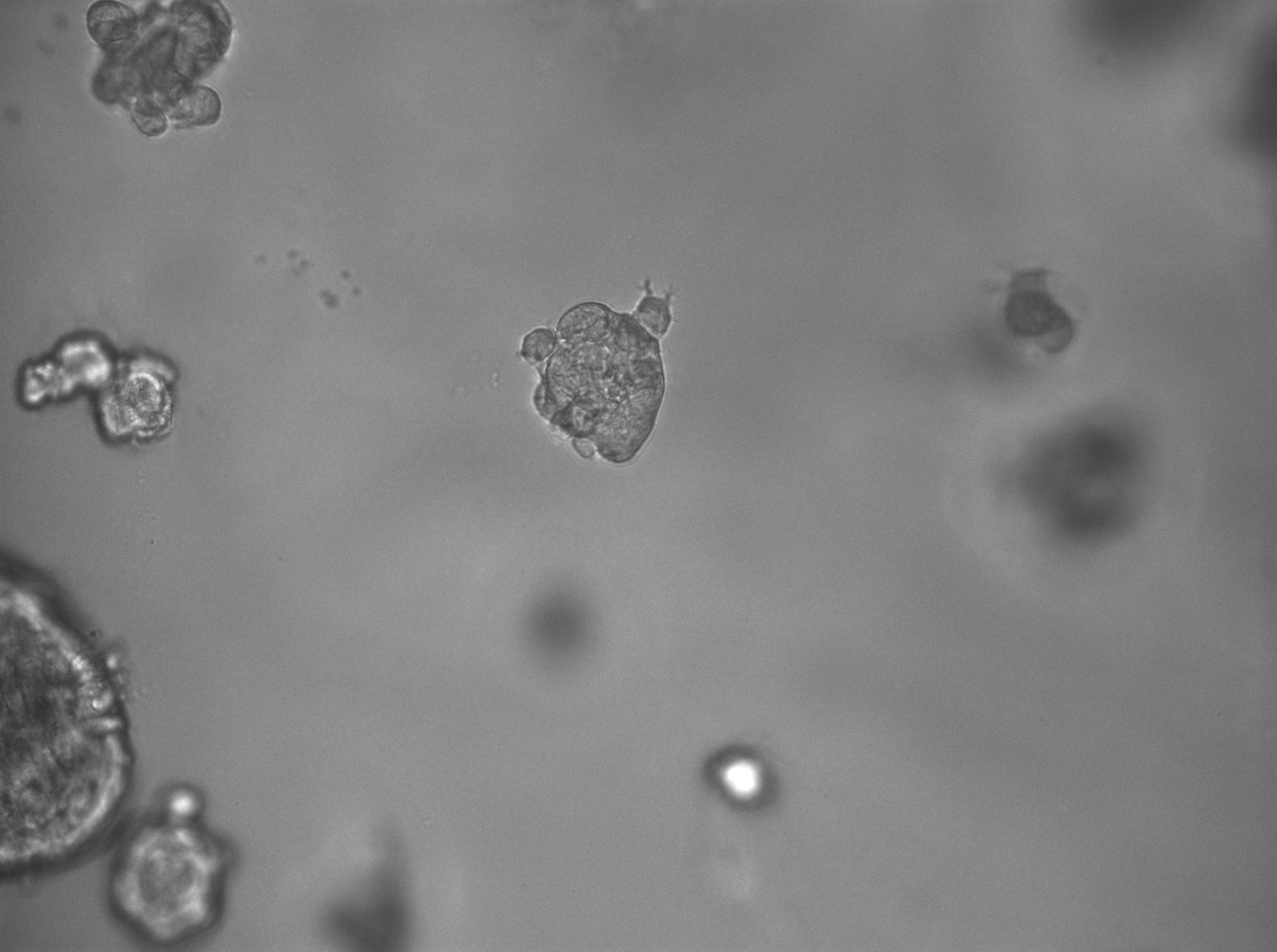

Supplement: Supplementary file 4 — Source Data Fig. 4 [file 41586_2026_10187_MOESM4_ESM.zip › HCEC1CT/HCEC1CT-KRAS_D10_Dox-02000_A01d_20x_ch00.jpg]

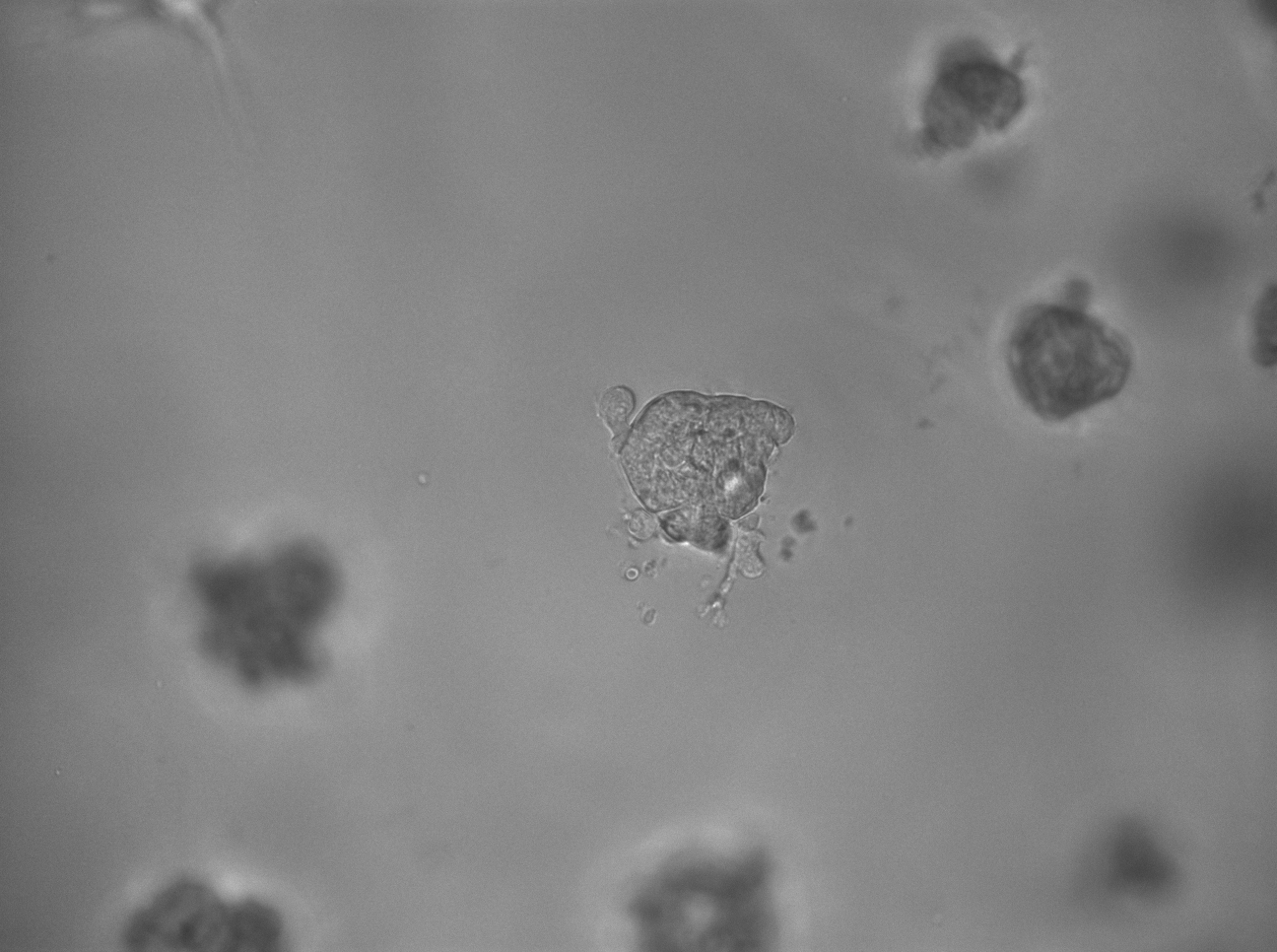

Supplement: Supplementary file 4 — Source Data Fig. 4 [file 41586_2026_10187_MOESM4_ESM.zip › HCEC1CT/HCEC1CT-KRAS_D10_Dox-02000_A01e_20x_ch00.jpg]

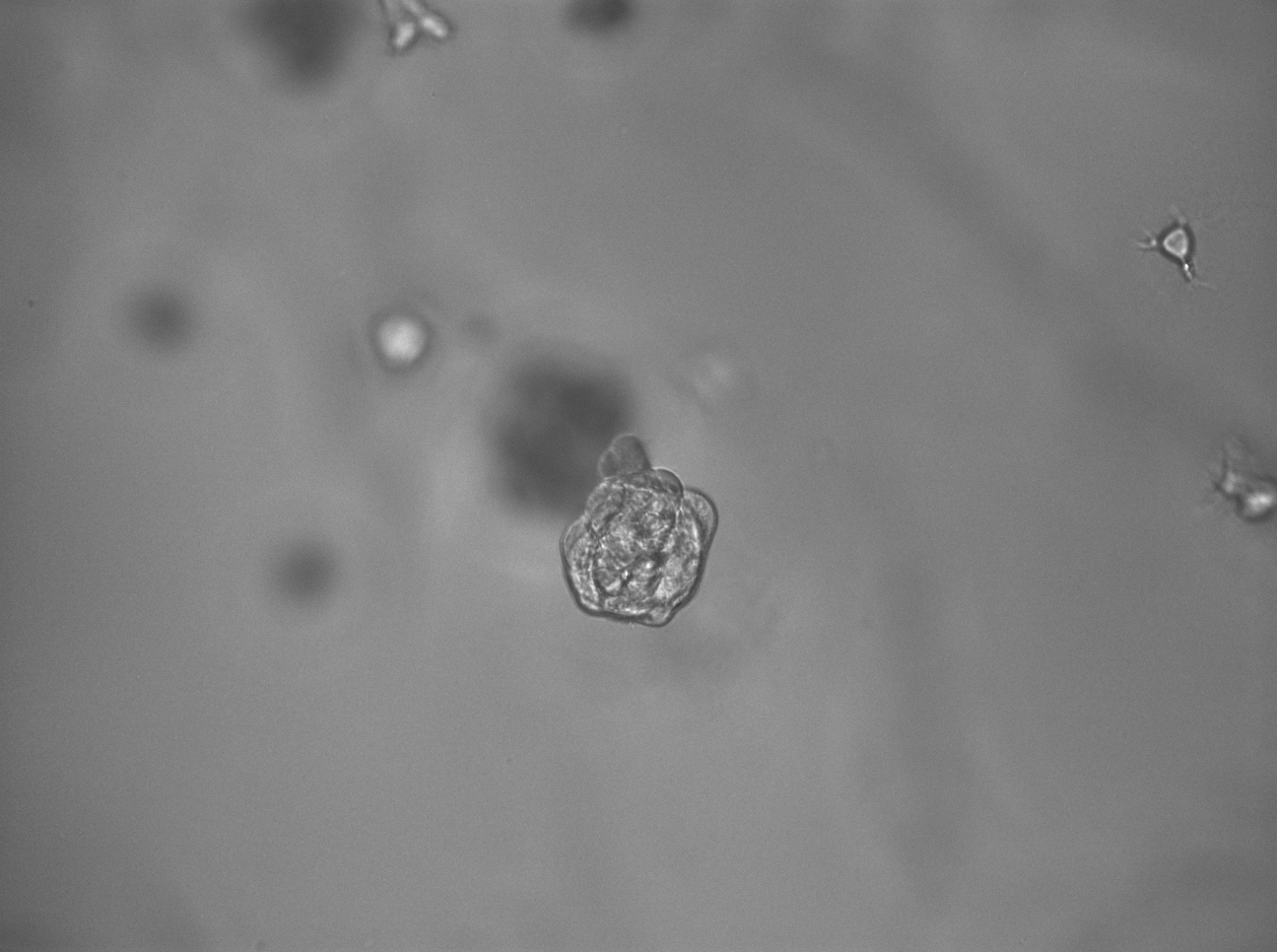

Supplement: Supplementary file 4 — Source Data Fig. 4 [file 41586_2026_10187_MOESM4_ESM.zip › HCEC1CT/HCEC1CT-KRAS_D10_Dox-02000_A01f_20x_ch00.jpg]

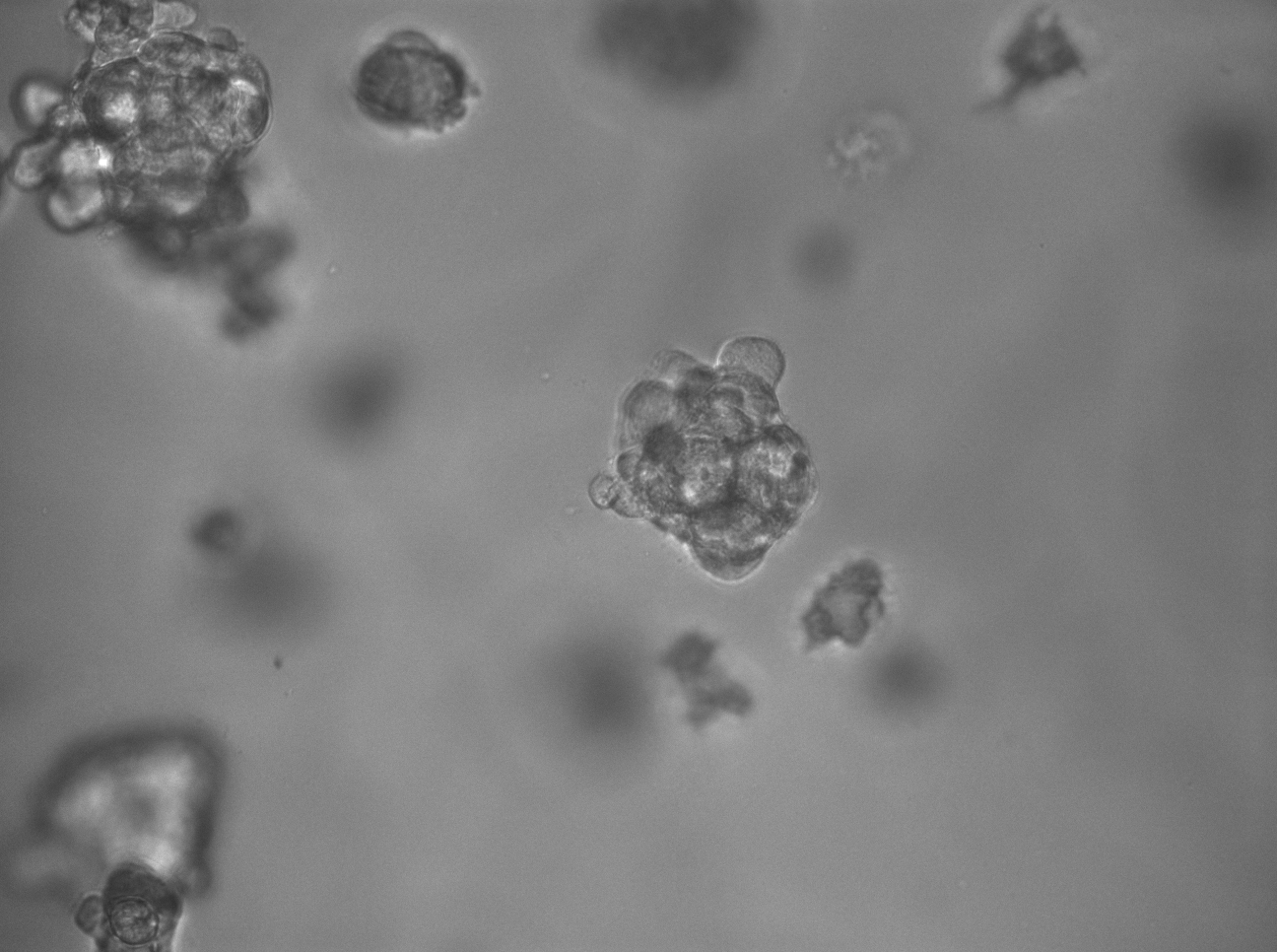

Supplement: Supplementary file 4 — Source Data Fig. 4 [file 41586_2026_10187_MOESM4_ESM.zip › HCEC1CT/HCEC1CT-KRAS_D10_Dox-02000_A01g_20x_ch00.jpg]

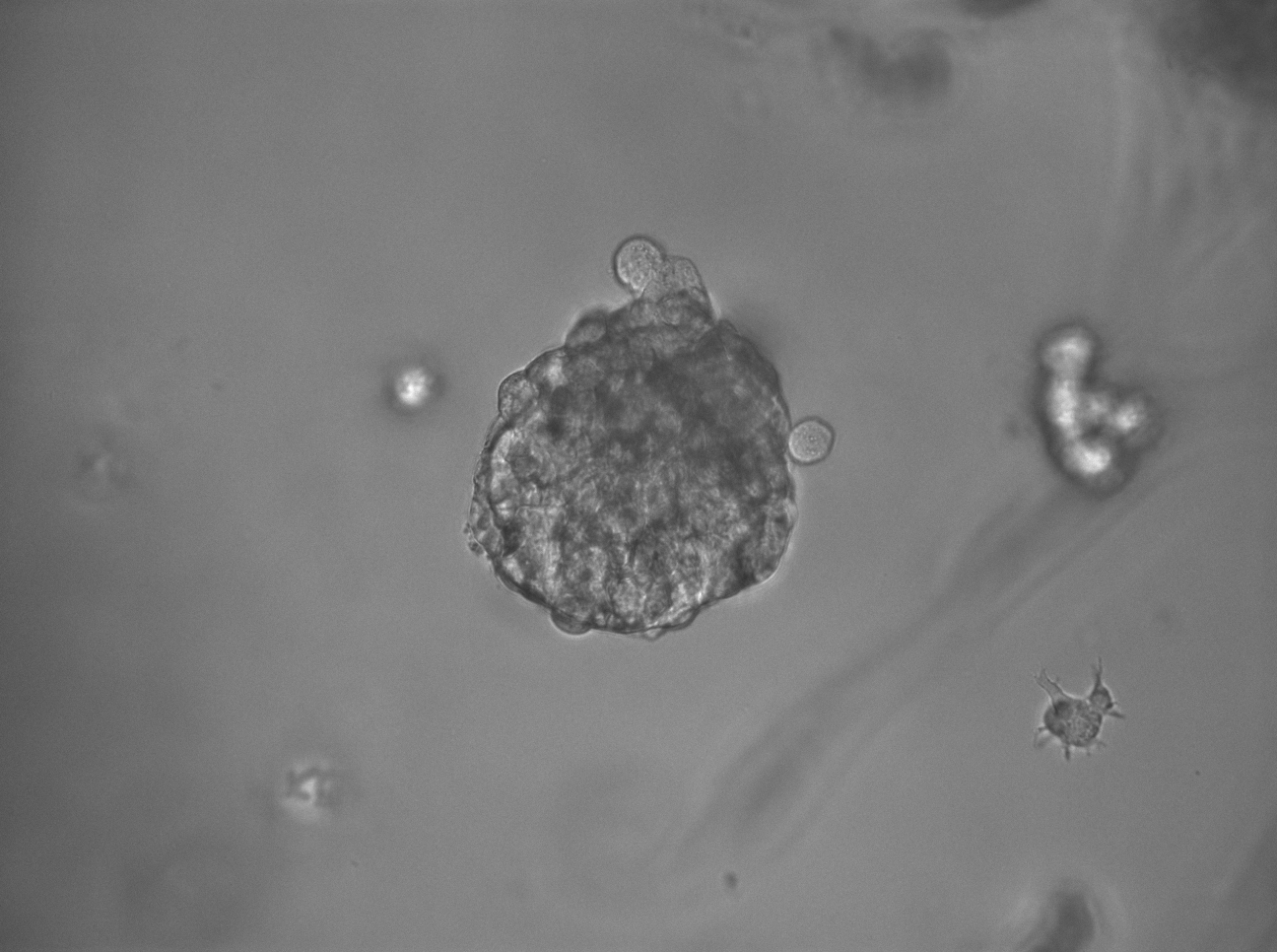

Supplement: Supplementary file 4 — Source Data Fig. 4 [file 41586_2026_10187_MOESM4_ESM.zip › HCEC1CT/HCEC1CT-KRAS_D10_Dox-02000_A02a_20x_ch00.jpg]

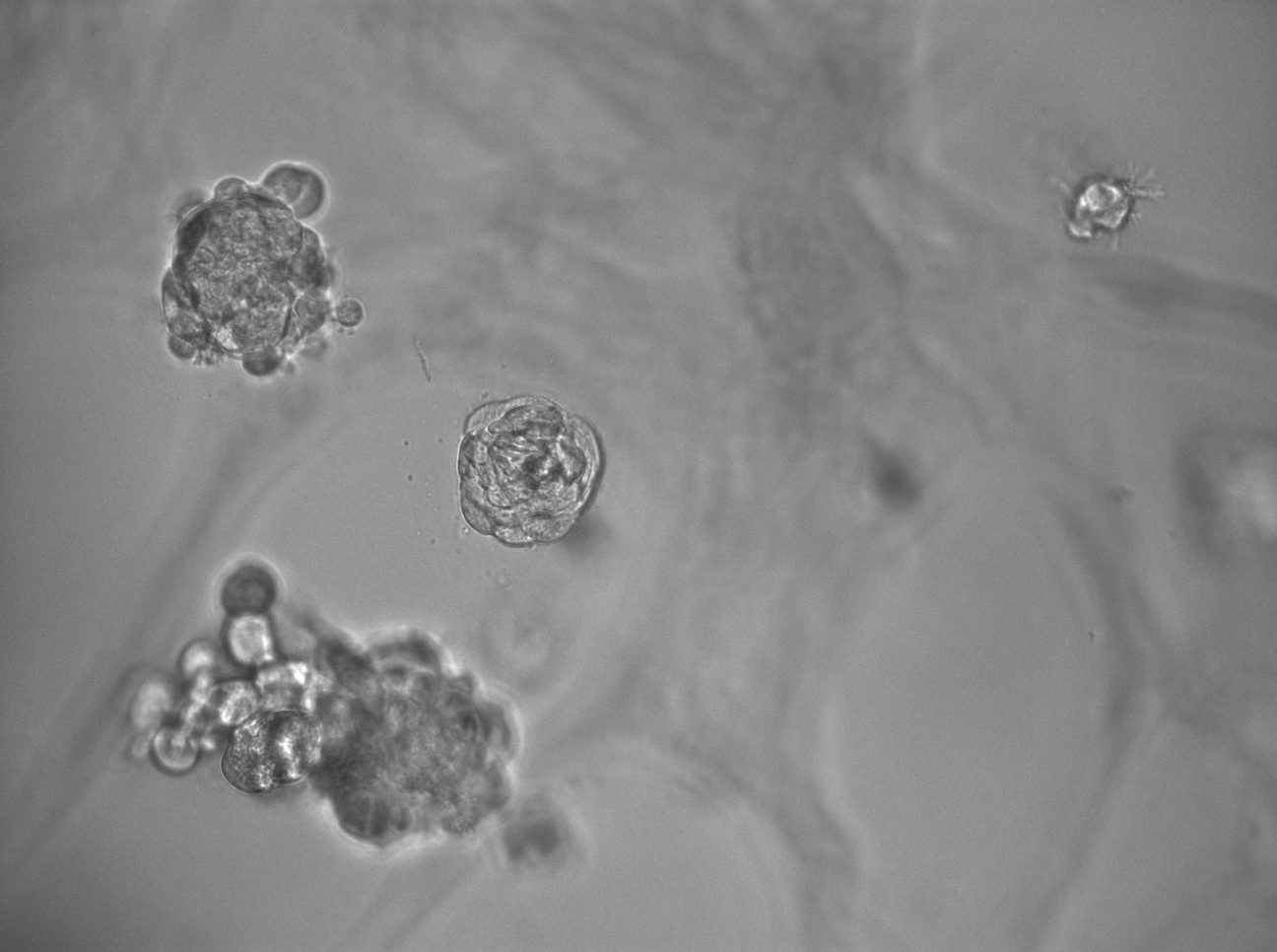

Supplement: Supplementary file 4 — Source Data Fig. 4 [file 41586_2026_10187_MOESM4_ESM.zip › HCEC1CT/HCEC1CT-KRAS_D10_Dox-02000_A02b_20x_ch00.jpg]

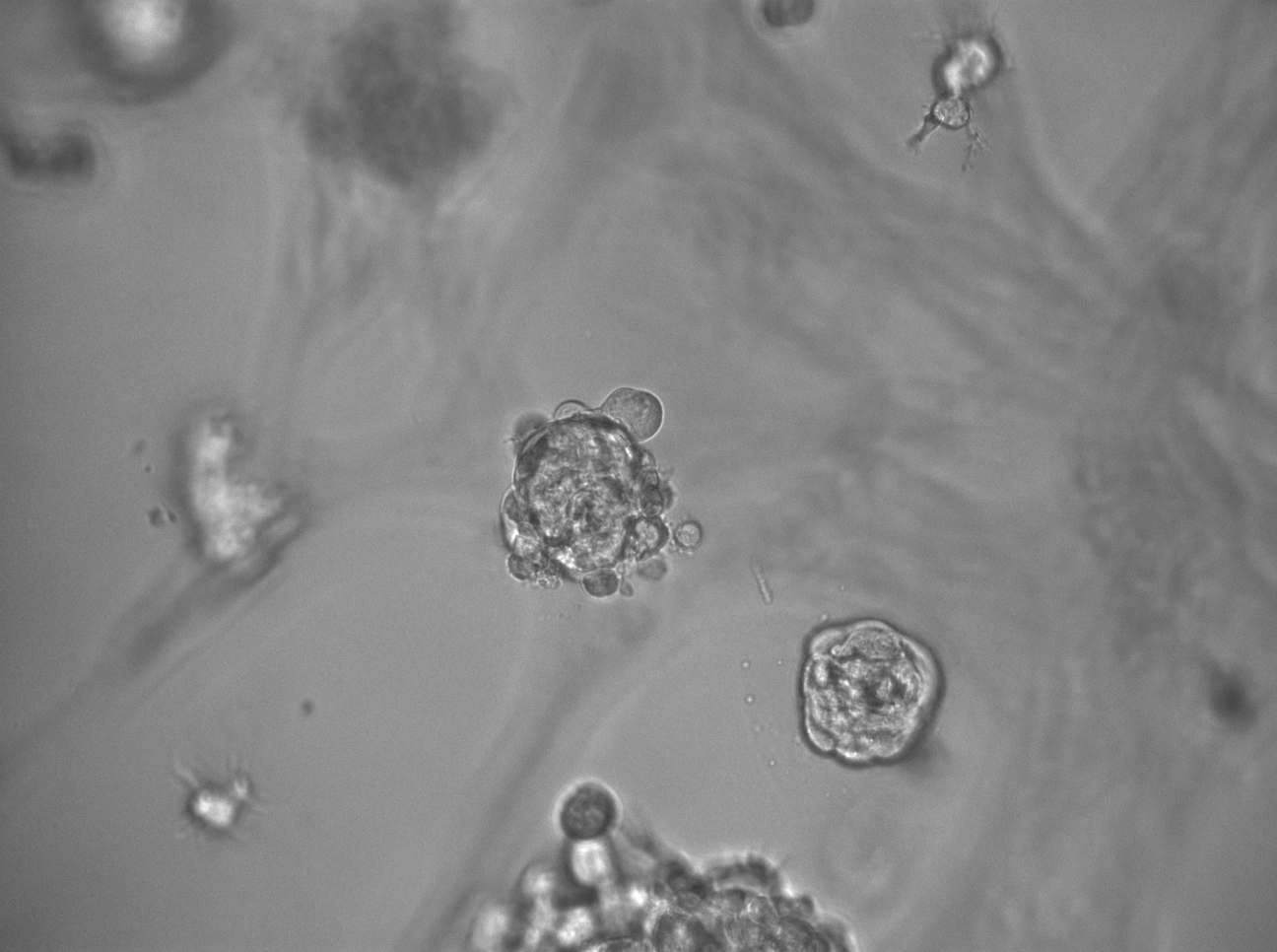

Supplement: Supplementary file 4 — Source Data Fig. 4 [file 41586_2026_10187_MOESM4_ESM.zip › HCEC1CT/HCEC1CT-KRAS_D10_Dox-02000_A02c_20x_ch00.jpg]

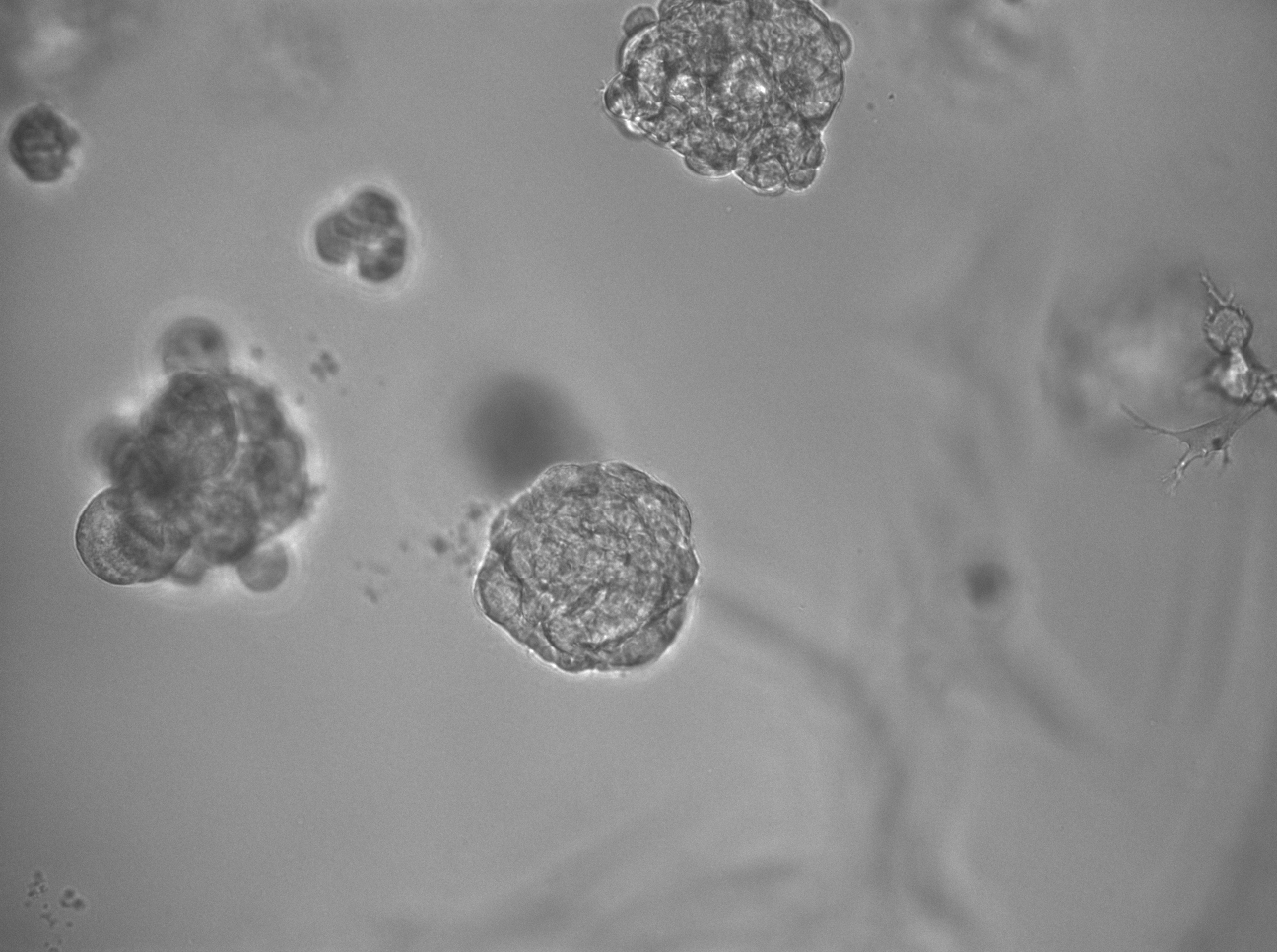

Supplement: Supplementary file 4 — Source Data Fig. 4 [file 41586_2026_10187_MOESM4_ESM.zip › HCEC1CT/HCEC1CT-KRAS_D10_Dox-02000_A02d_20x_ch00.jpg]

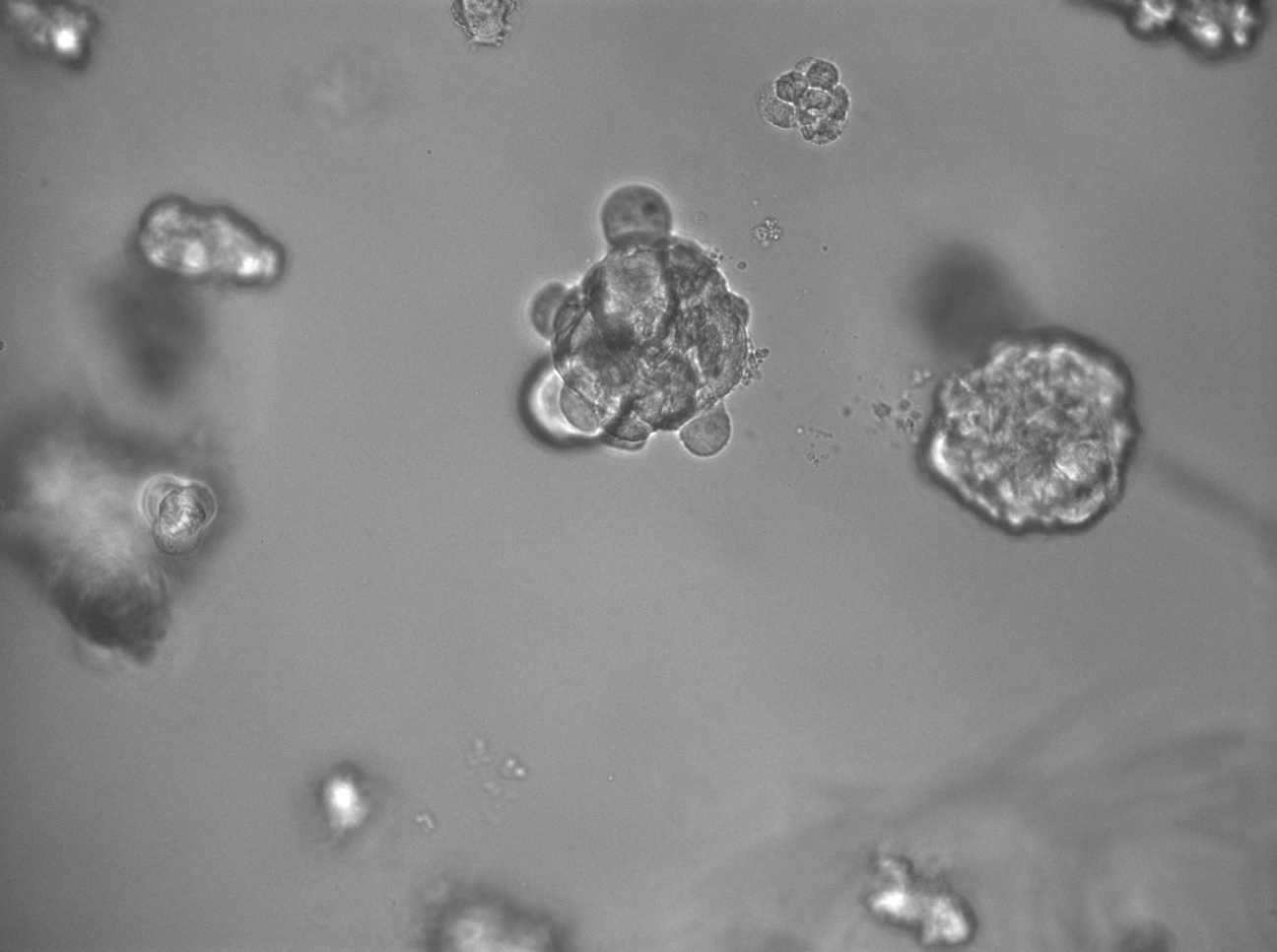

Supplement: Supplementary file 4 — Source Data Fig. 4 [file 41586_2026_10187_MOESM4_ESM.zip › HCEC1CT/HCEC1CT-KRAS_D10_Dox-02000_A02e_20x_ch00.jpg]

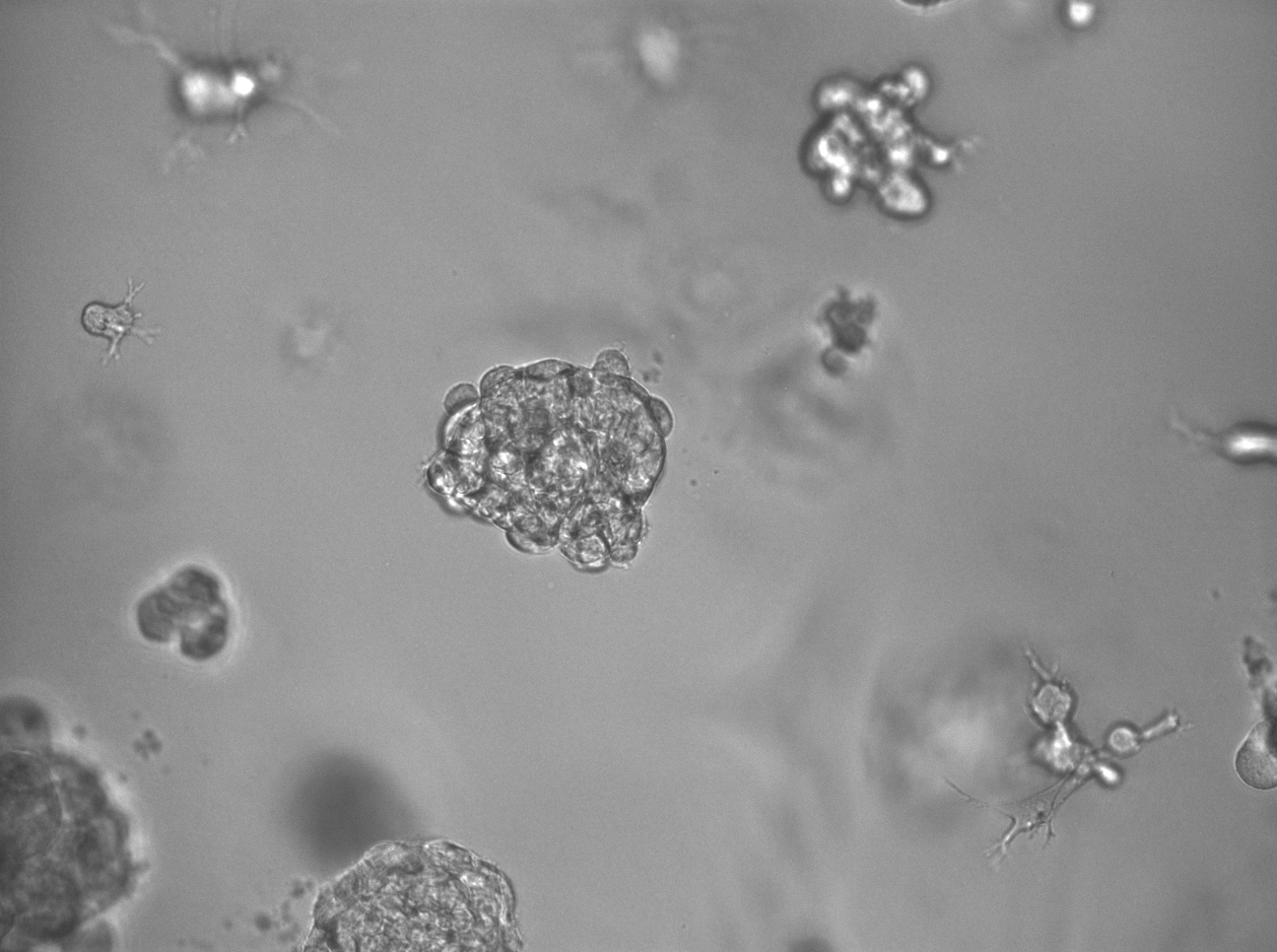

Supplement: Supplementary file 4 — Source Data Fig. 4 [file 41586_2026_10187_MOESM4_ESM.zip › HCEC1CT/HCEC1CT-KRAS_D10_Dox-02000_A02f_20x_ch00.jpg]

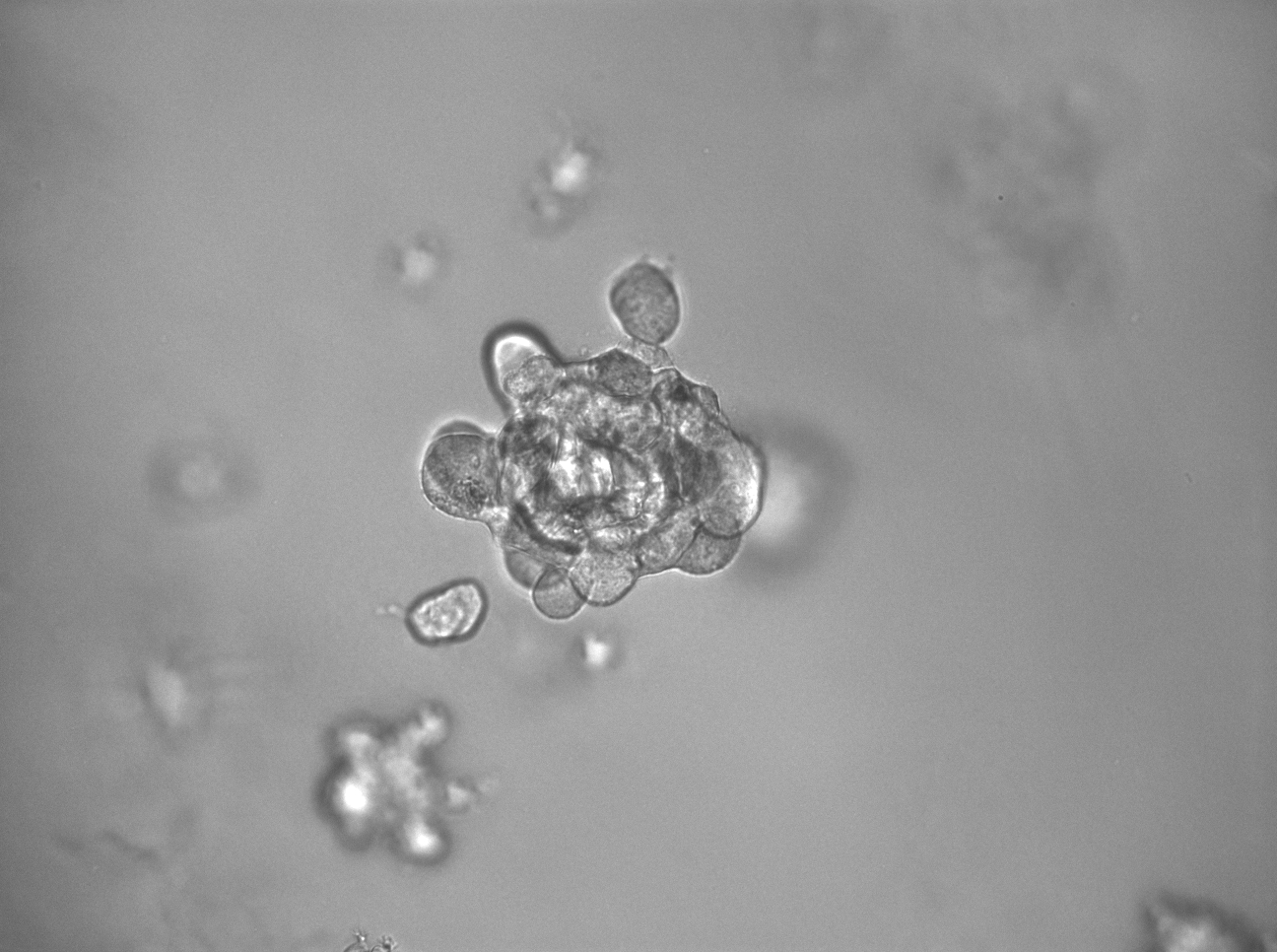

Supplement: Supplementary file 4 — Source Data Fig. 4 [file 41586_2026_10187_MOESM4_ESM.zip › HCEC1CT/HCEC1CT-KRAS_D10_Dox-02000_A02g_20x_ch00.jpg]

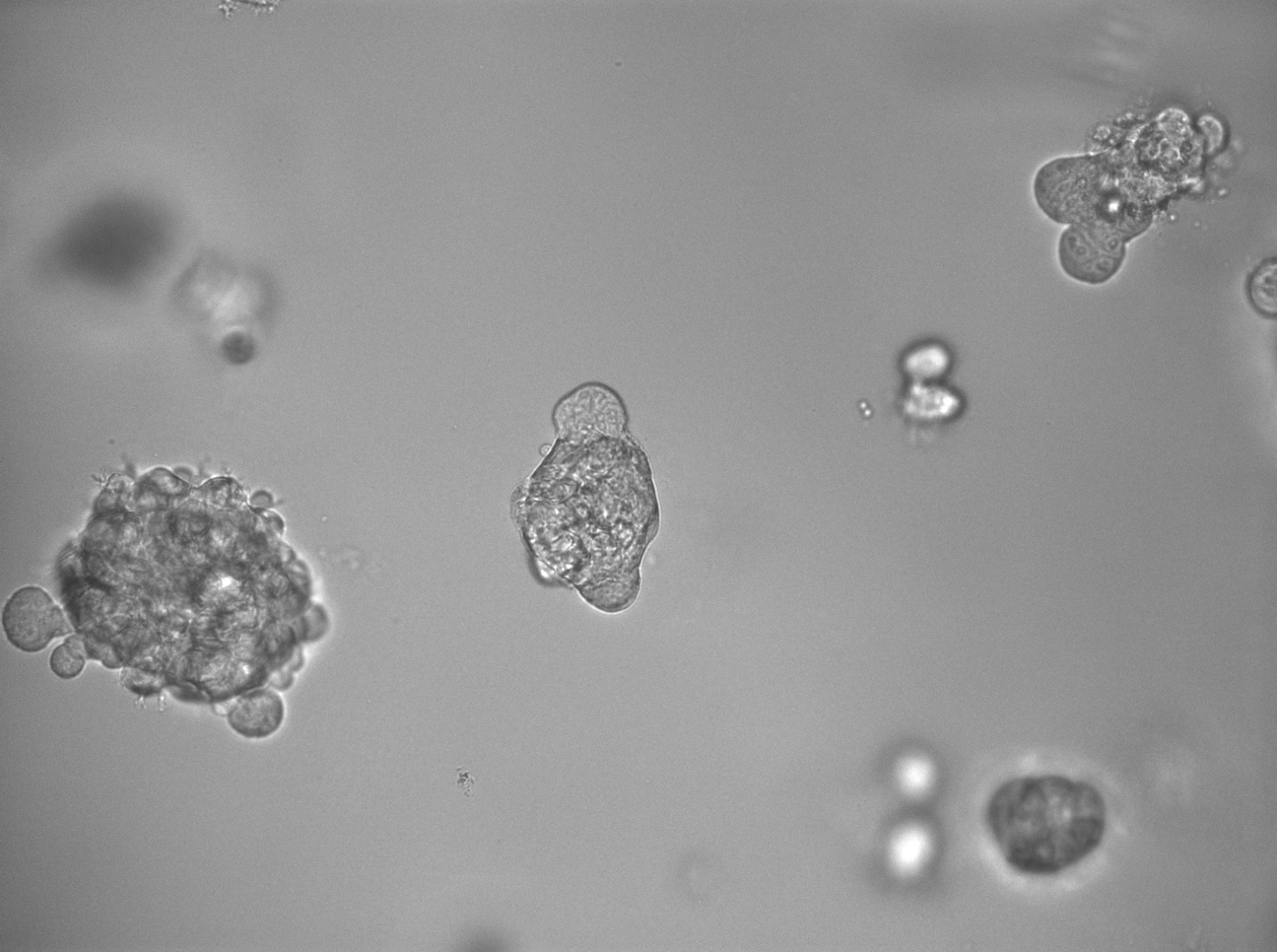

Supplement: Supplementary file 4 — Source Data Fig. 4 [file 41586_2026_10187_MOESM4_ESM.zip › HCEC1CT/HCEC1CT-KRAS_D10_Dox-02000_A03a_20x_ch00.jpg]

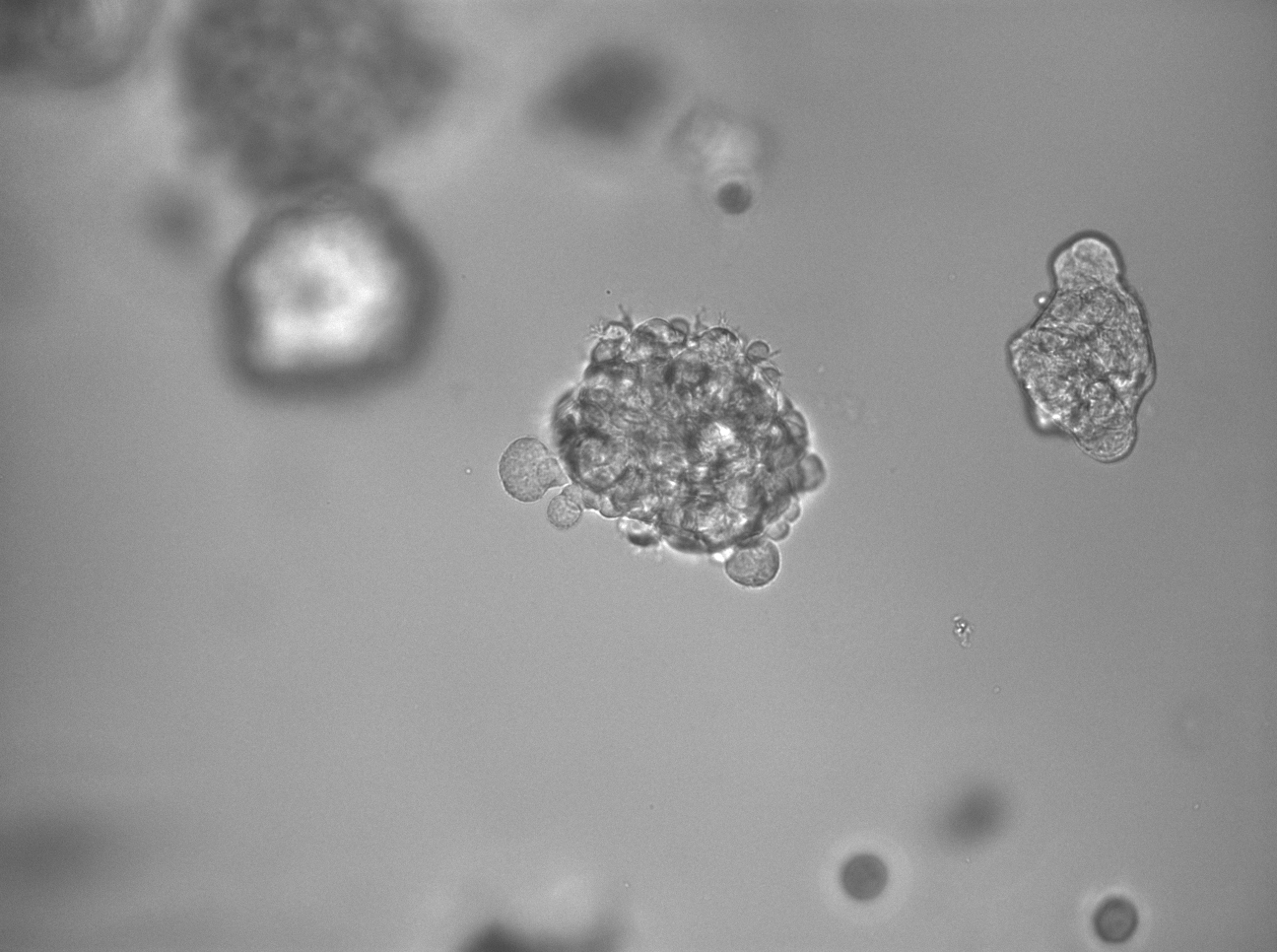

Supplement: Supplementary file 4 — Source Data Fig. 4 [file 41586_2026_10187_MOESM4_ESM.zip › HCEC1CT/HCEC1CT-KRAS_D10_Dox-02000_A03b_20x_ch00.jpg]

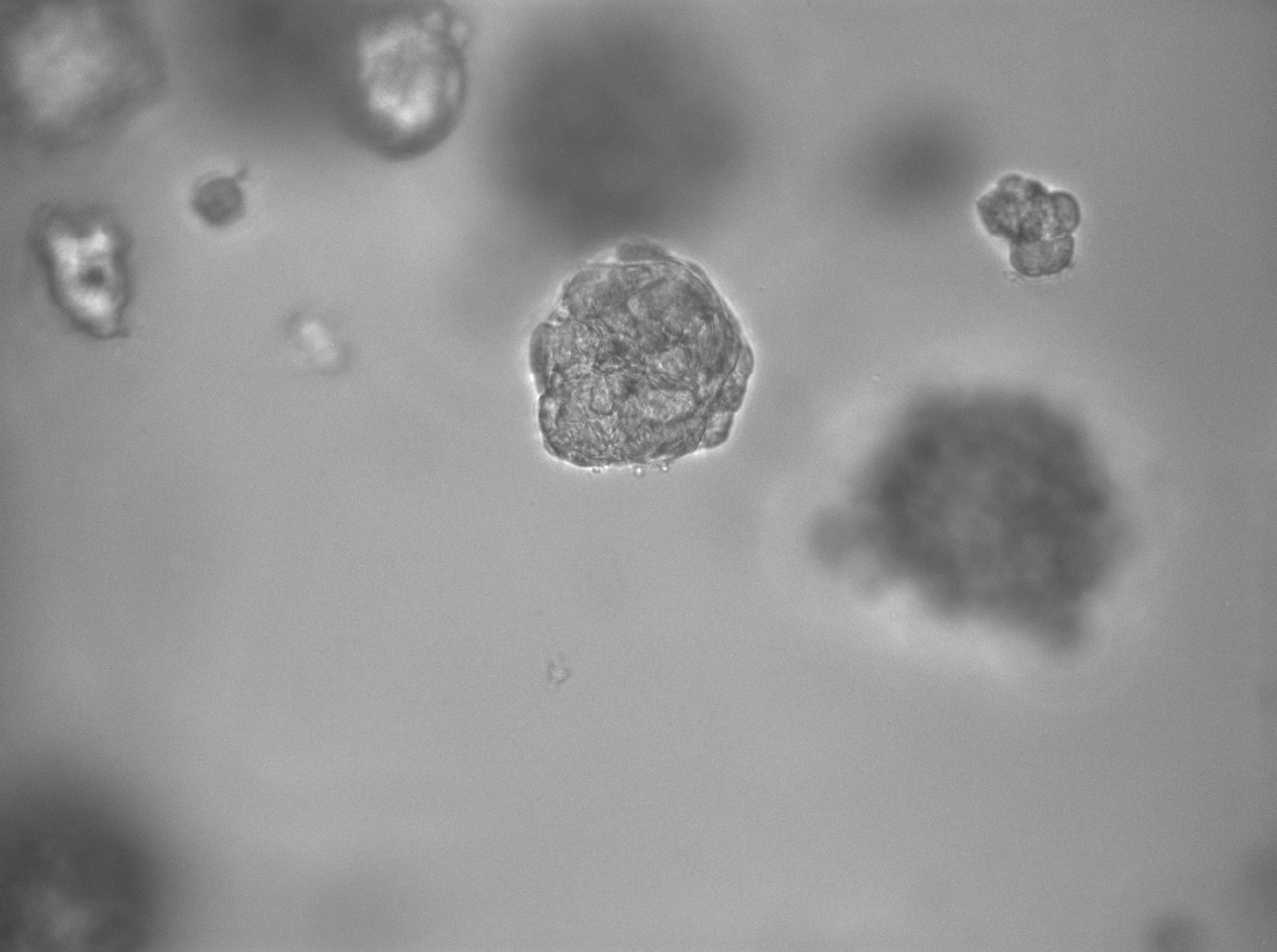

Supplement: Supplementary file 4 — Source Data Fig. 4 [file 41586_2026_10187_MOESM4_ESM.zip › HCEC1CT/HCEC1CT-KRAS_D10_Dox-02000_A03c_20x_ch00.jpg]

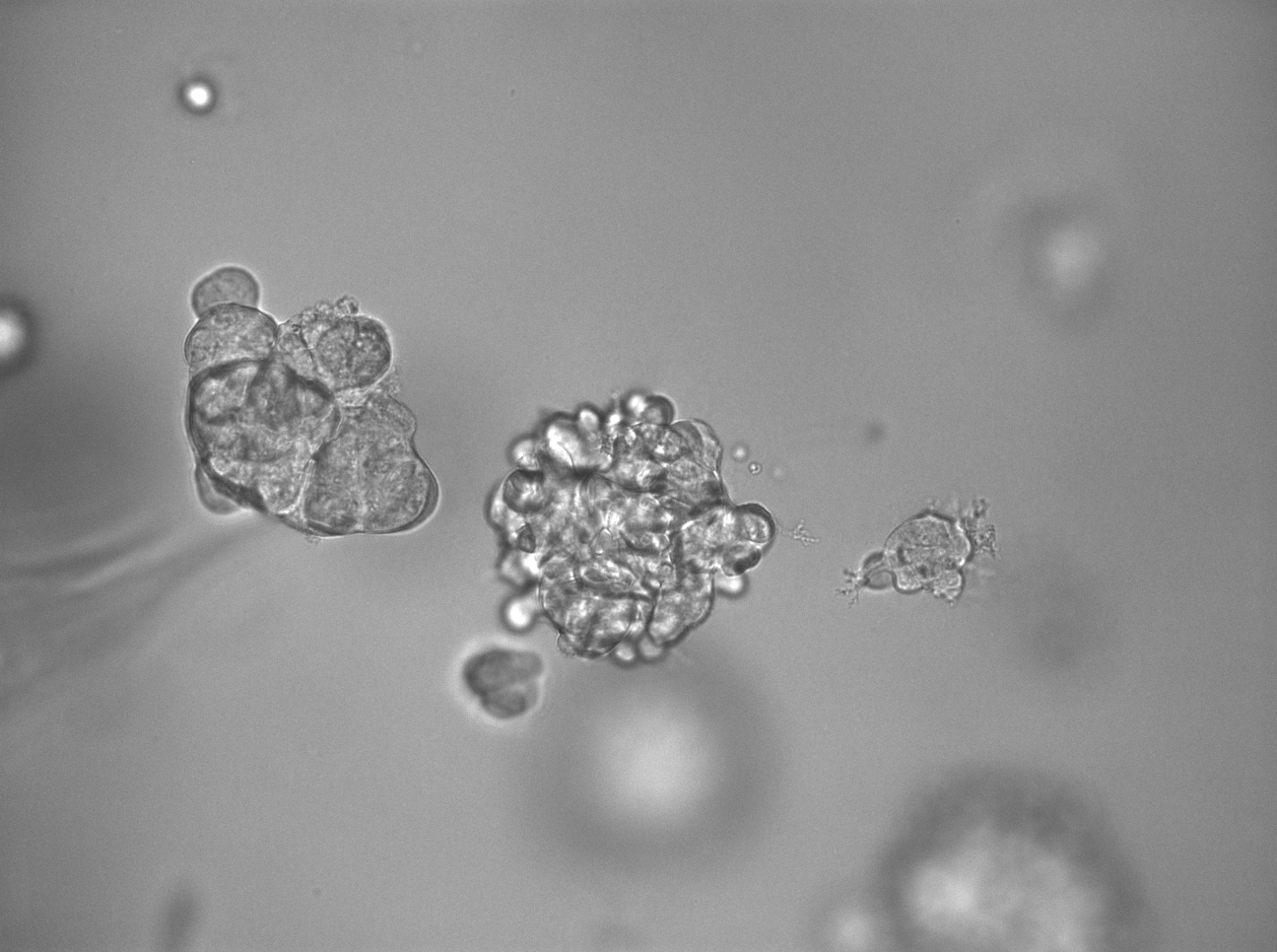

Supplement: Supplementary file 4 — Source Data Fig. 4 [file 41586_2026_10187_MOESM4_ESM.zip › HCEC1CT/HCEC1CT-KRAS_D10_Dox-02000_A03d_20x_ch00.jpg]

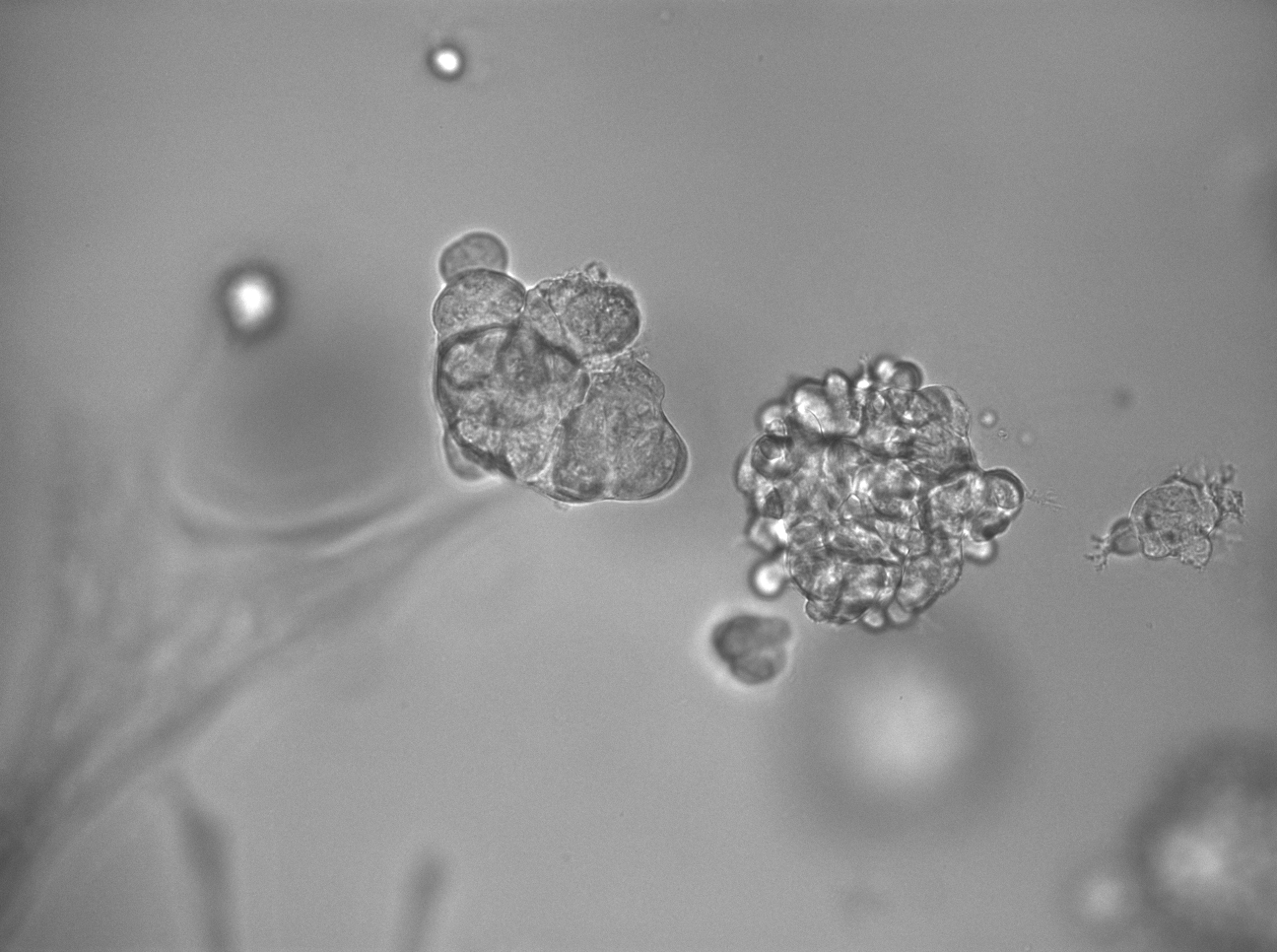

Supplement: Supplementary file 4 — Source Data Fig. 4 [file 41586_2026_10187_MOESM4_ESM.zip › HCEC1CT/HCEC1CT-KRAS_D10_Dox-02000_A03e_20x_ch00.jpg]

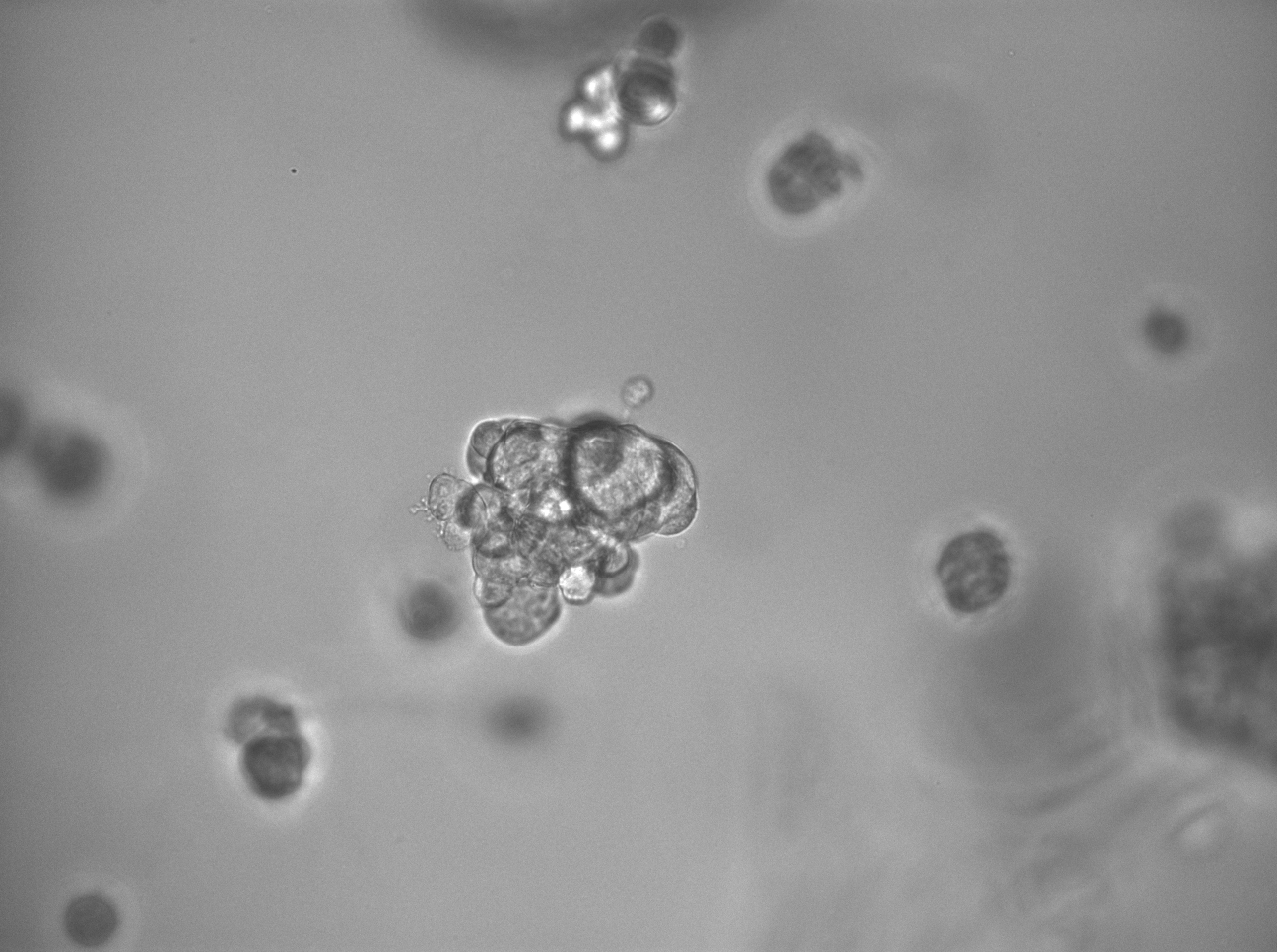

Supplement: Supplementary file 4 — Source Data Fig. 4 [file 41586_2026_10187_MOESM4_ESM.zip › HCEC1CT/HCEC1CT-KRAS_D10_Dox-02000_A03f_20x_ch00.jpg]

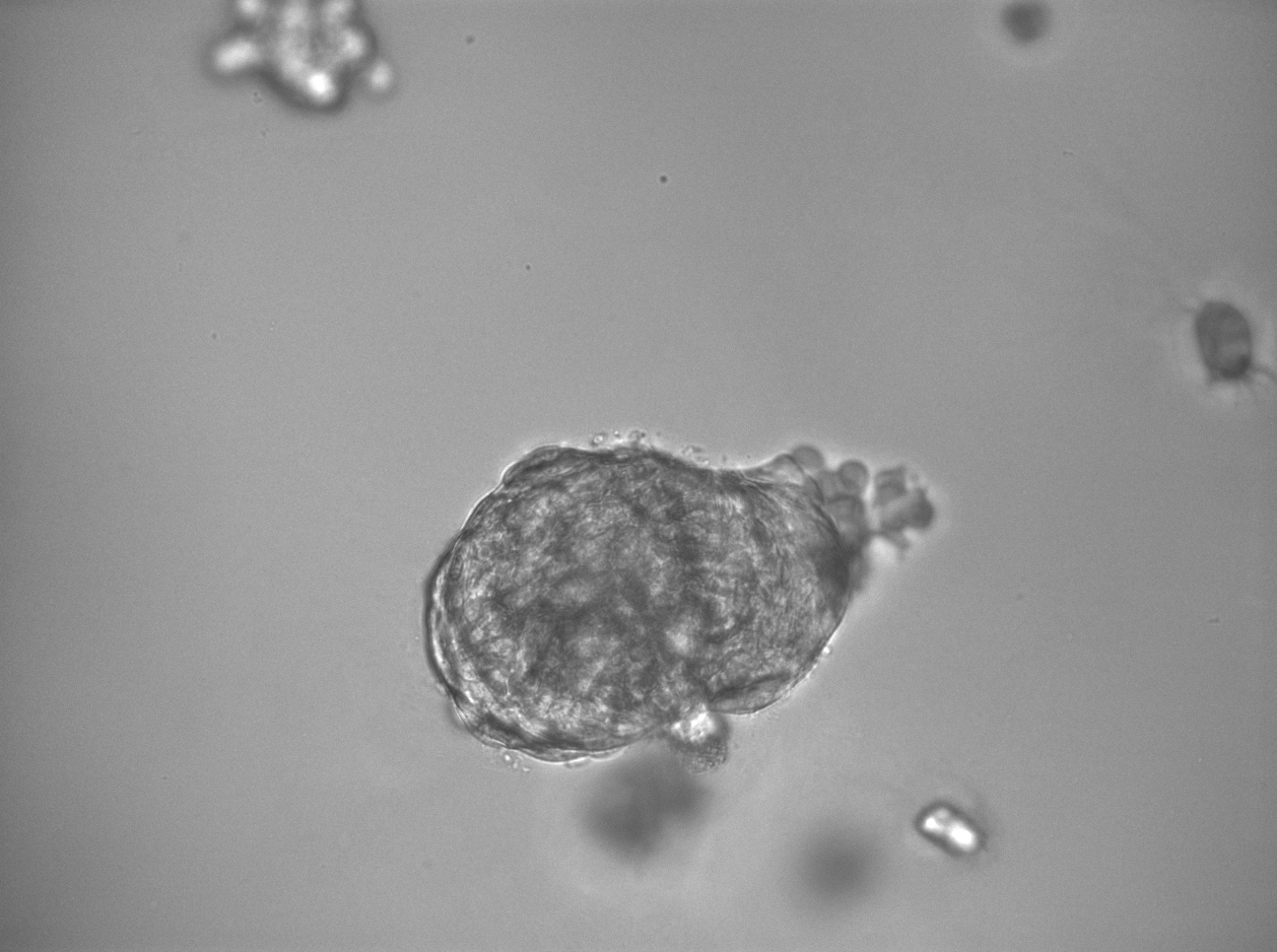

Supplement: Supplementary file 4 — Source Data Fig. 4 [file 41586_2026_10187_MOESM4_ESM.zip › HCEC1CT/HCEC1CT-KRAS_D10_Dox-02000_A03g_20x_ch00.jpg]

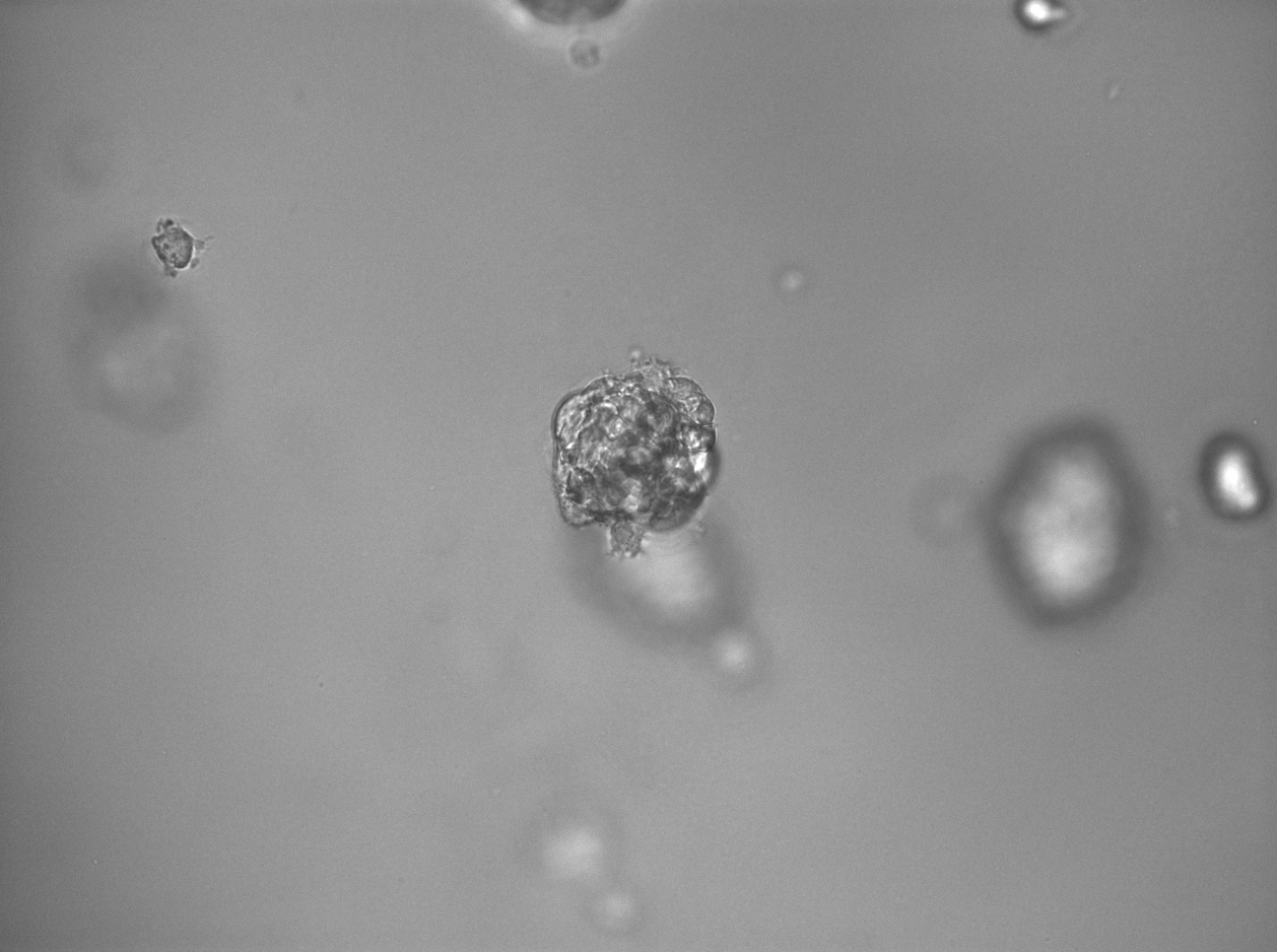

Supplement: Supplementary file 4 — Source Data Fig. 4 [file 41586_2026_10187_MOESM4_ESM.zip › HCEC1CT/HCEC1CT-KRAS_D10_Dox-00000_A01a_20x_ch00.jpg]

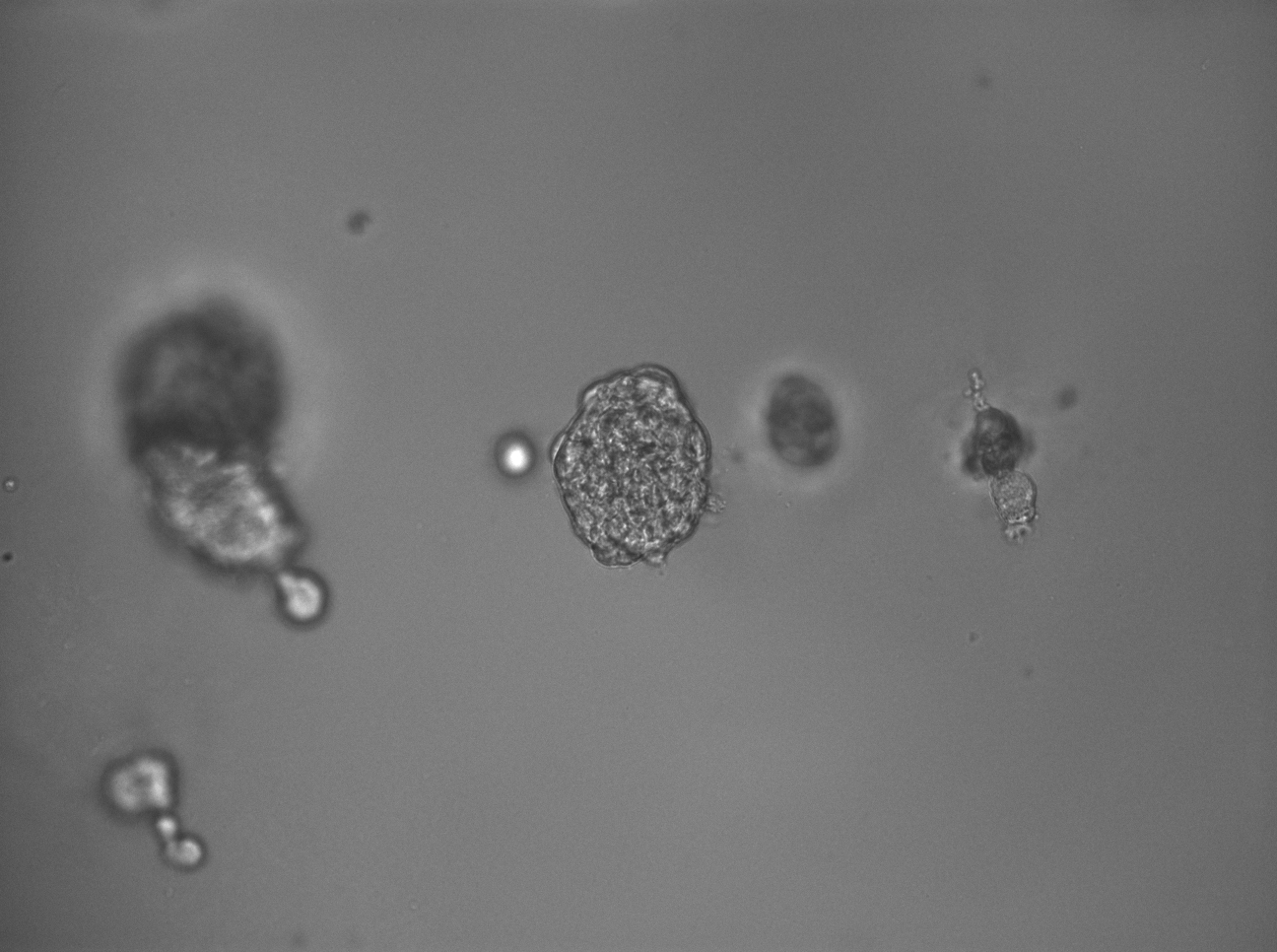

Supplement: Supplementary file 4 — Source Data Fig. 4 [file 41586_2026_10187_MOESM4_ESM.zip › HCEC1CT/HCEC1CT-KRAS_D10_Dox-00000_A01b_20x_ch00.jpg]

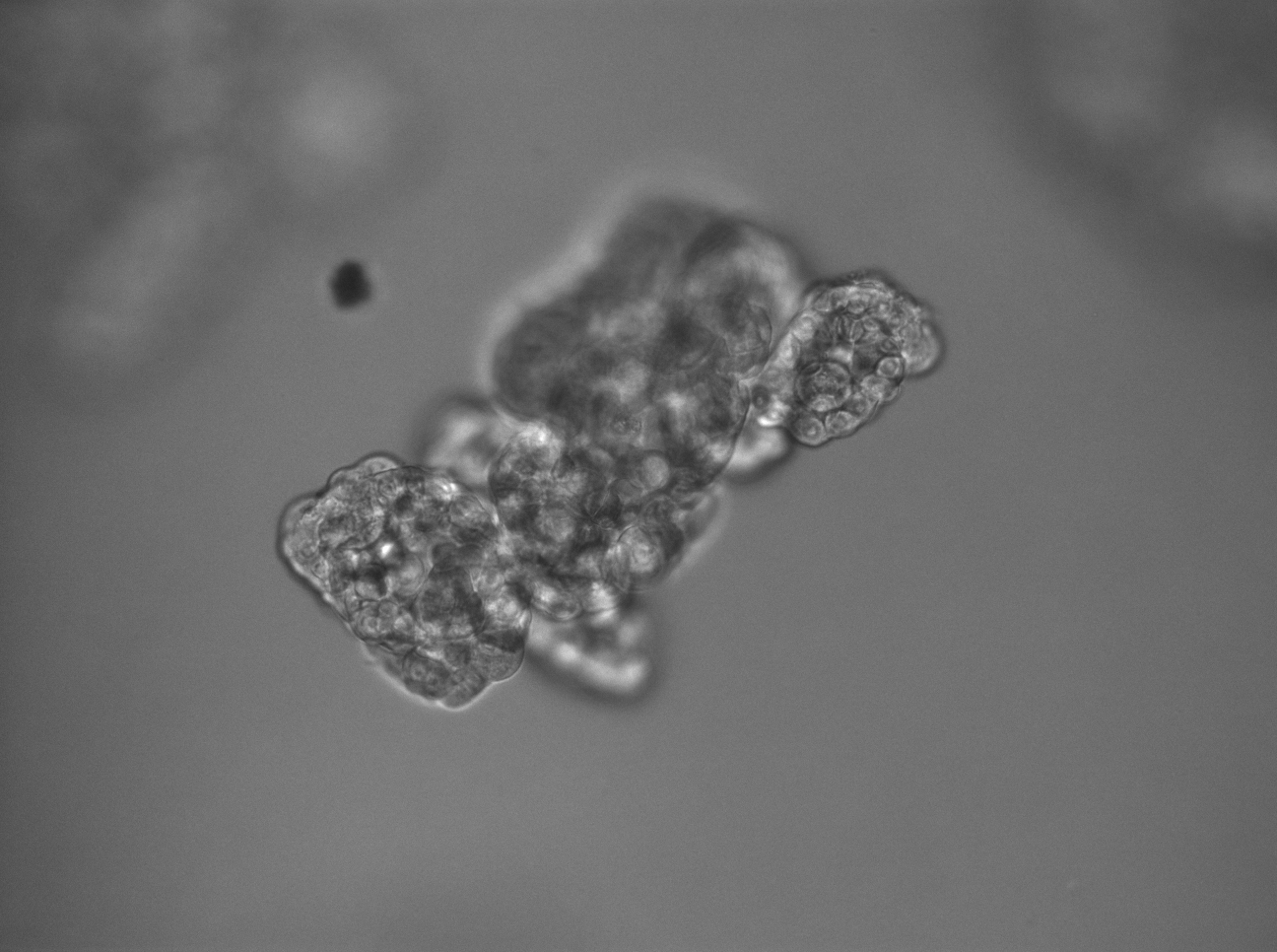

Supplement: Supplementary file 4 — Source Data Fig. 4 [file 41586_2026_10187_MOESM4_ESM.zip › HPDE/HPDE-GFP_D10_Dox-00000_C01_20xa_ch00.jpg]

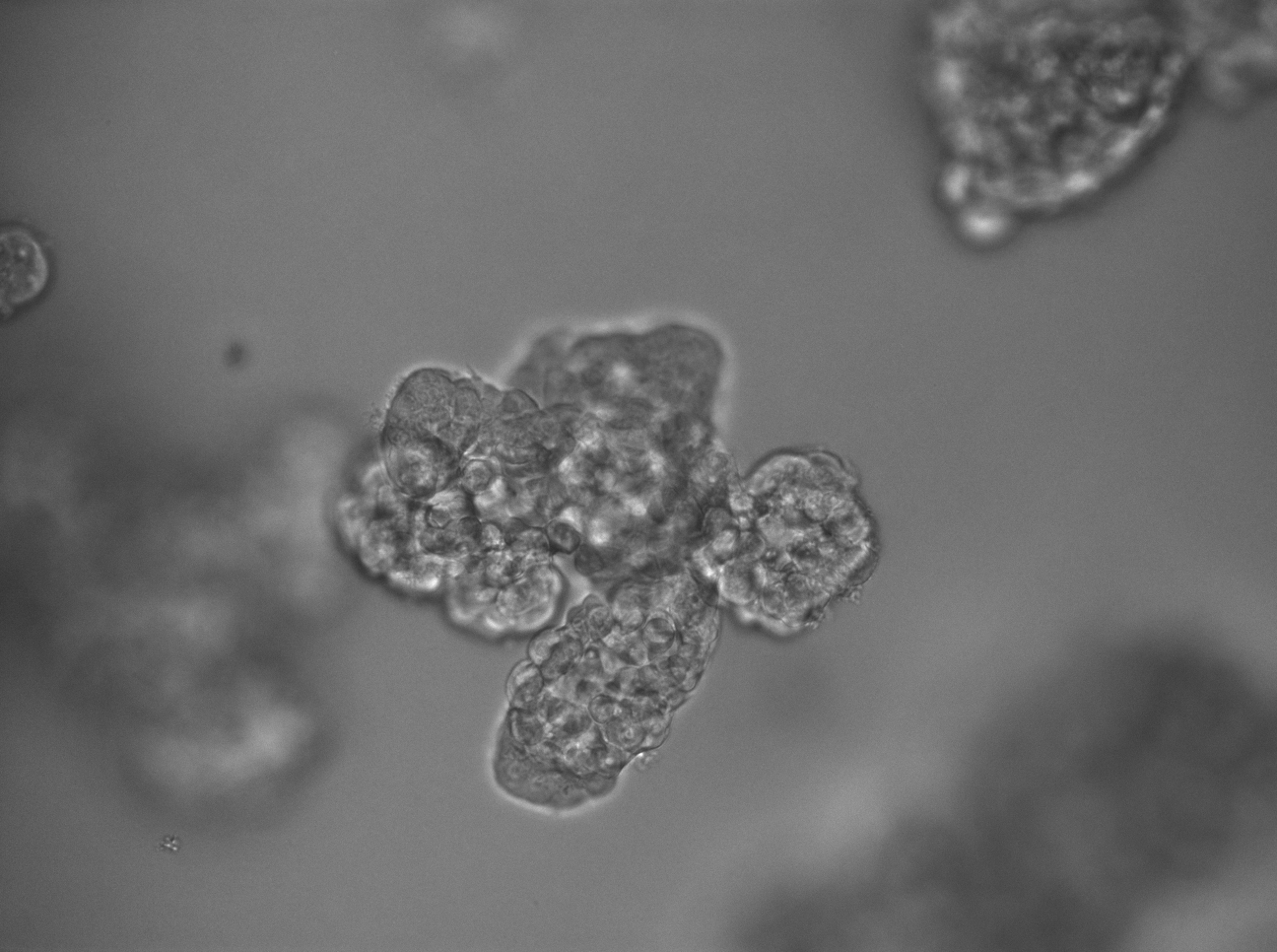

Supplement: Supplementary file 4 — Source Data Fig. 4 [file 41586_2026_10187_MOESM4_ESM.zip › HPDE/HPDE-GFP_D10_Dox-00000_C01_20xb_ch00.jpg]

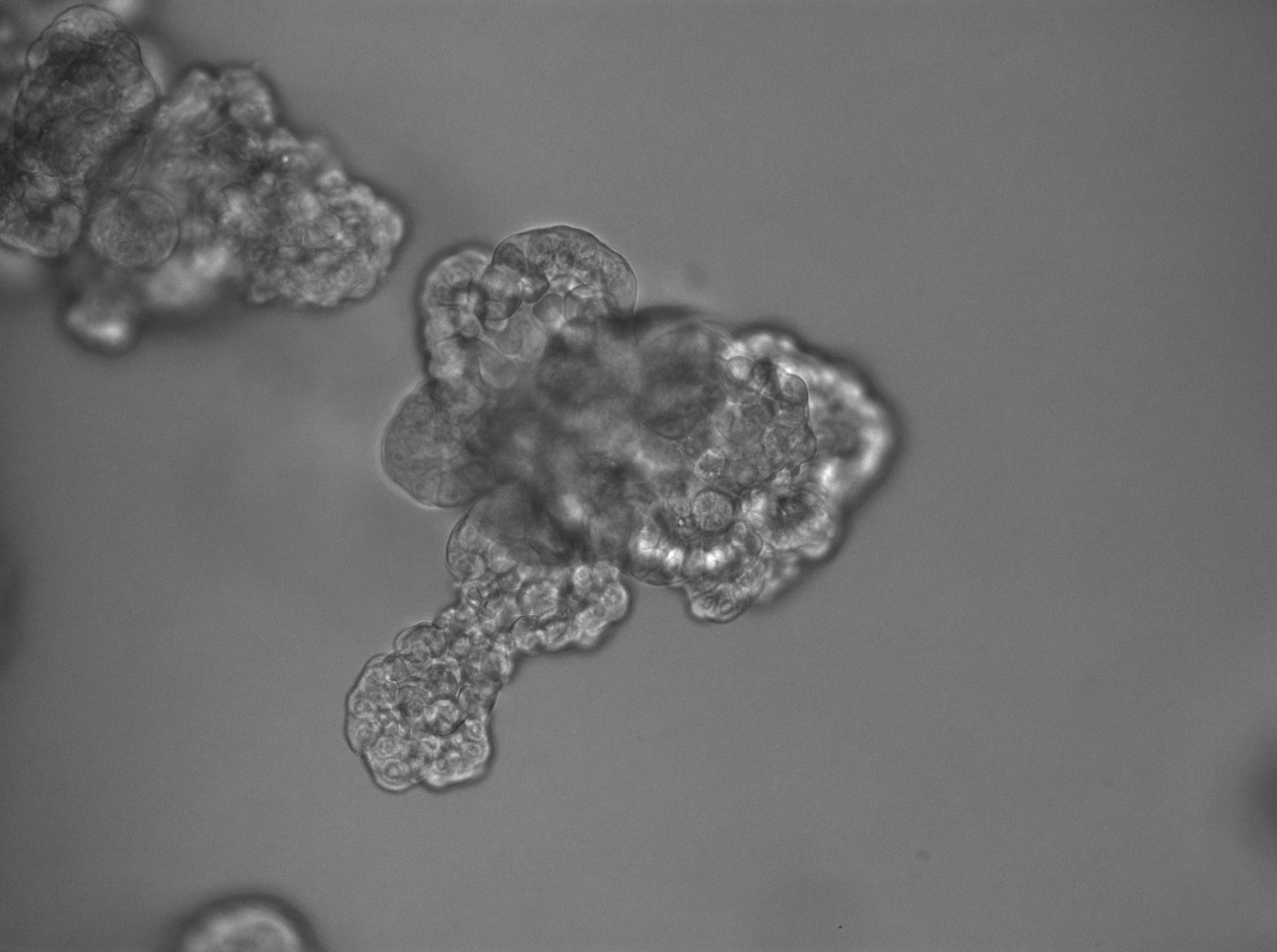

Supplement: Supplementary file 4 — Source Data Fig. 4 [file 41586_2026_10187_MOESM4_ESM.zip › HPDE/HPDE-GFP_D10_Dox-00000_C01_20xc_ch00.jpg]

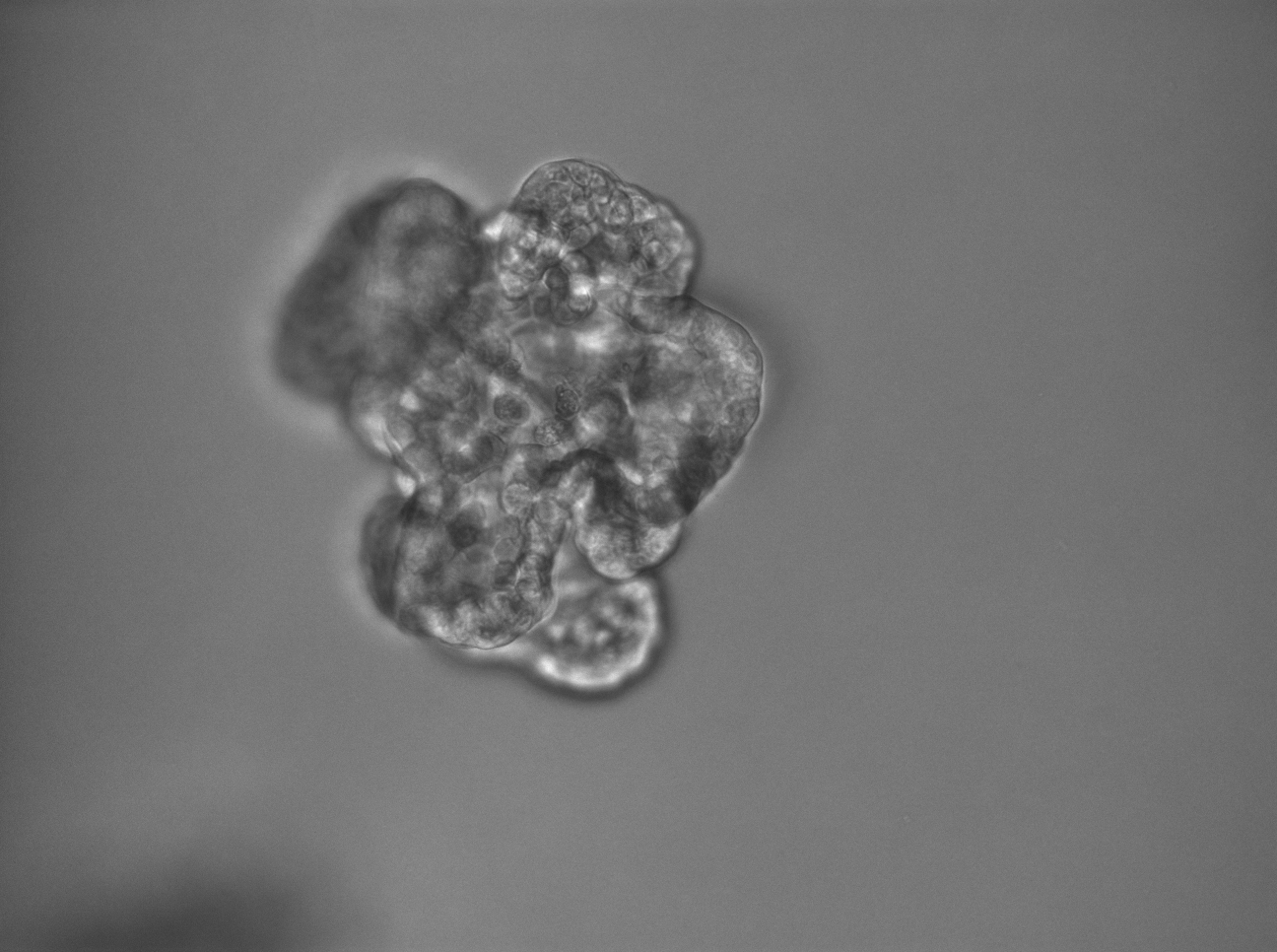

Supplement: Supplementary file 4 — Source Data Fig. 4 [file 41586_2026_10187_MOESM4_ESM.zip › HPDE/HPDE-GFP_D10_Dox-00000_C01_20xd_ch00.jpg]

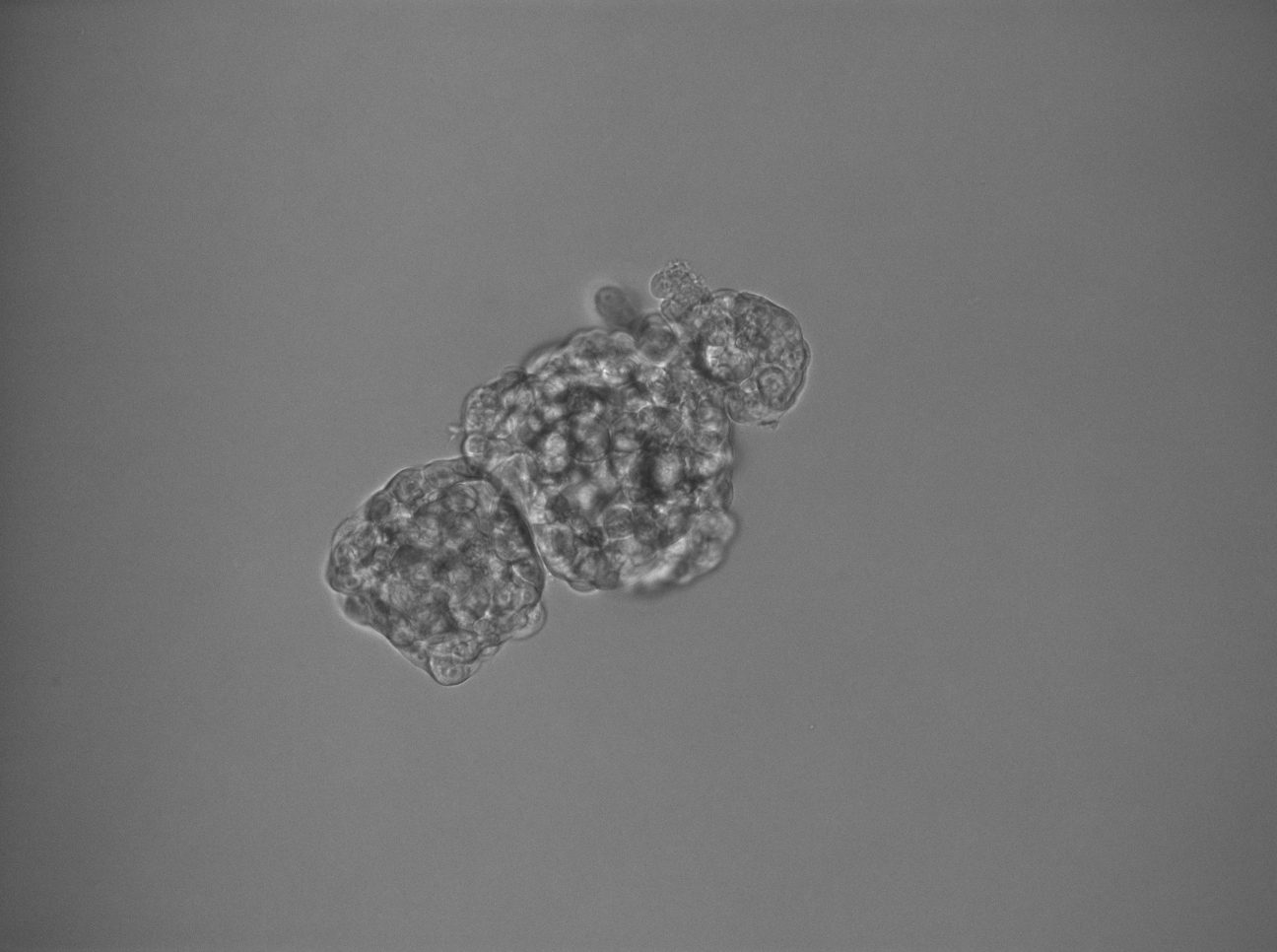

Supplement: Supplementary file 4 — Source Data Fig. 4 [file 41586_2026_10187_MOESM4_ESM.zip › HPDE/HPDE-GFP_D10_Dox-00000_C01_20xe_ch00.jpg]

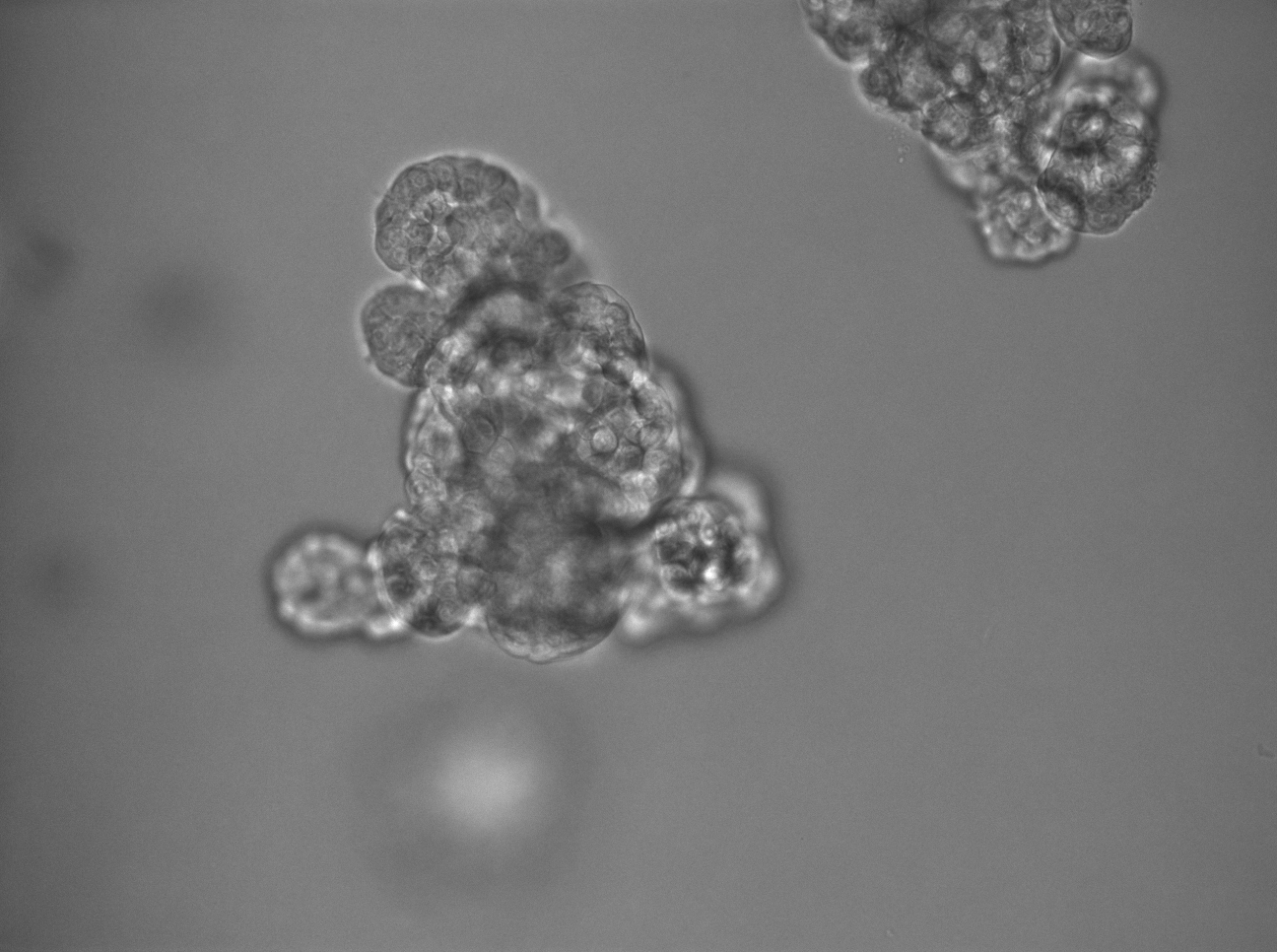

Supplement: Supplementary file 4 — Source Data Fig. 4 [file 41586_2026_10187_MOESM4_ESM.zip › HPDE/HPDE-GFP_D10_Dox-00000_C02_20xa_ch00.jpg]

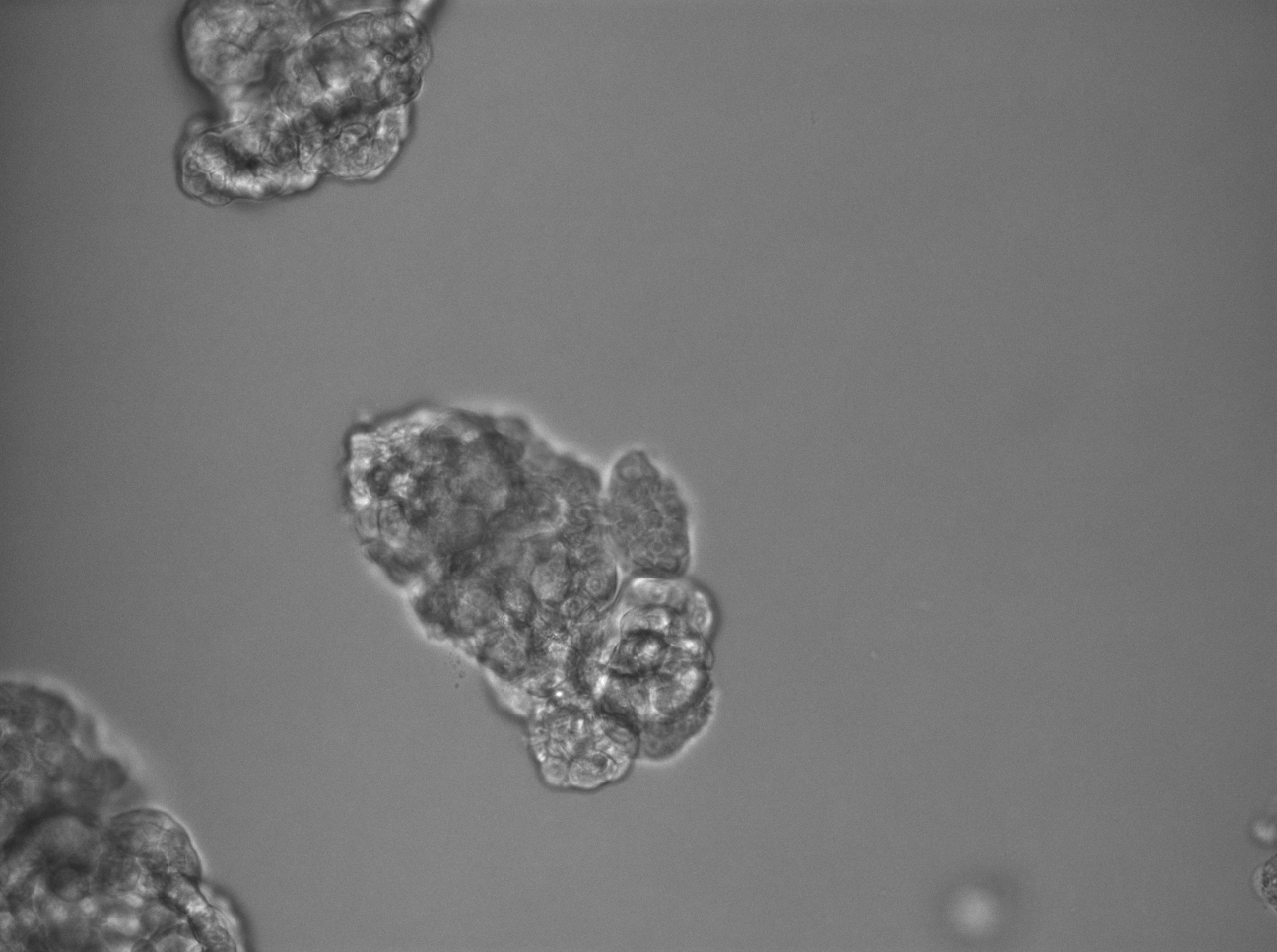

Supplement: Supplementary file 4 — Source Data Fig. 4 [file 41586_2026_10187_MOESM4_ESM.zip › HPDE/HPDE-GFP_D10_Dox-00000_C02_20xb_ch00.jpg]

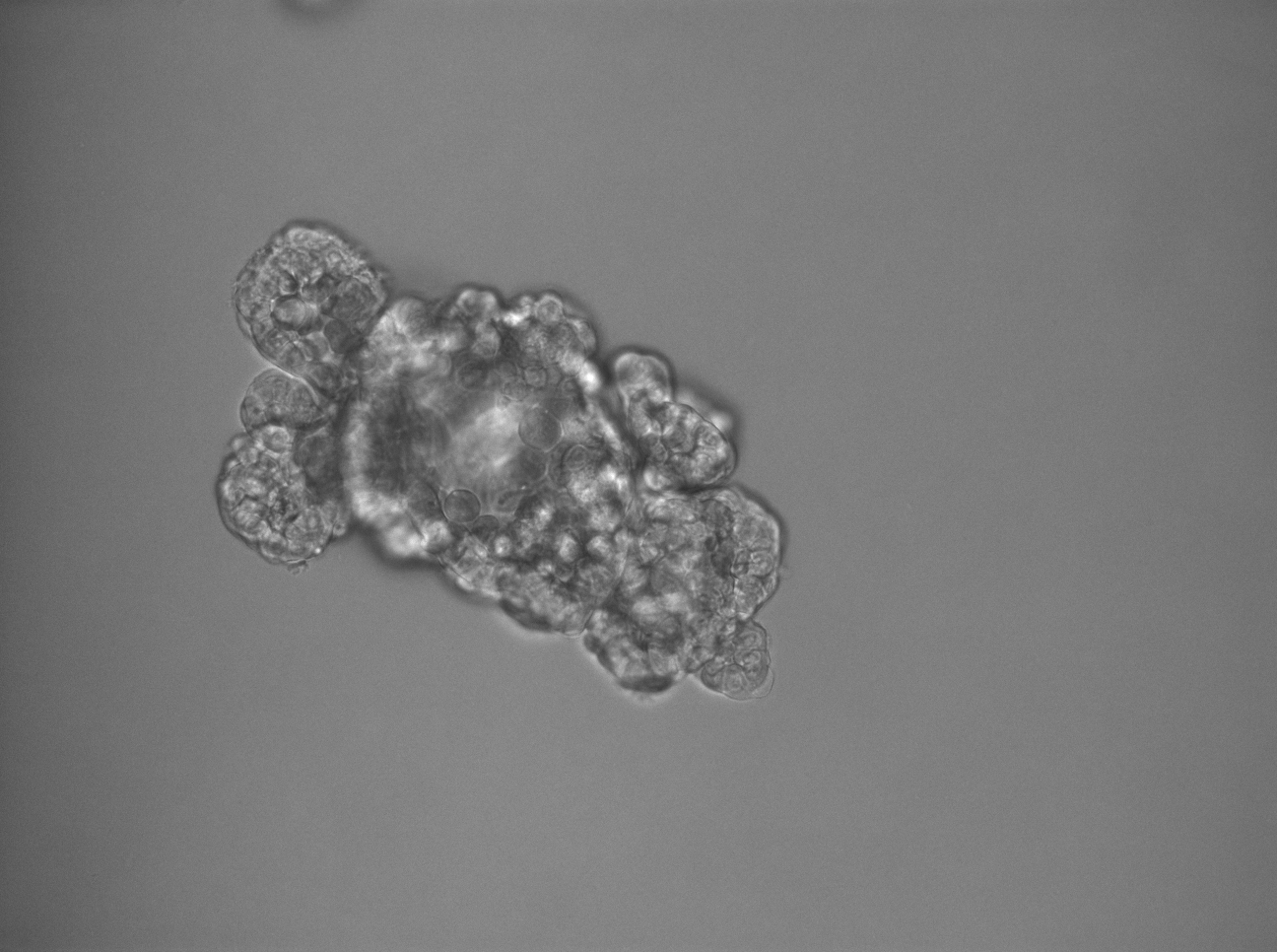

Supplement: Supplementary file 4 — Source Data Fig. 4 [file 41586_2026_10187_MOESM4_ESM.zip › HPDE/HPDE-GFP_D10_Dox-00000_C02_20xc_ch00.jpg]

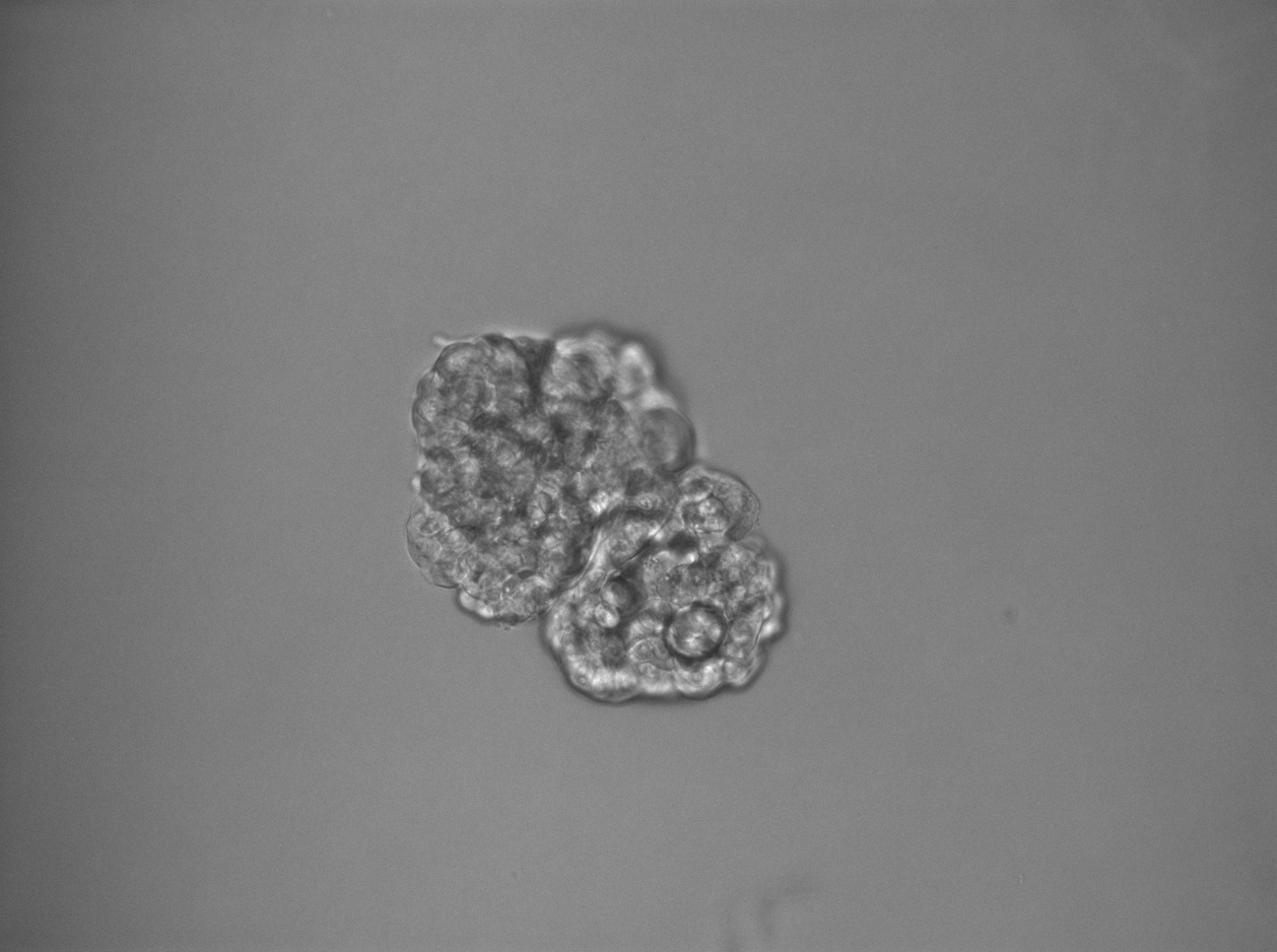

Supplement: Supplementary file 4 — Source Data Fig. 4 [file 41586_2026_10187_MOESM4_ESM.zip › HPDE/HPDE-GFP_D10_Dox-00000_C02_20xd_ch00.jpg]

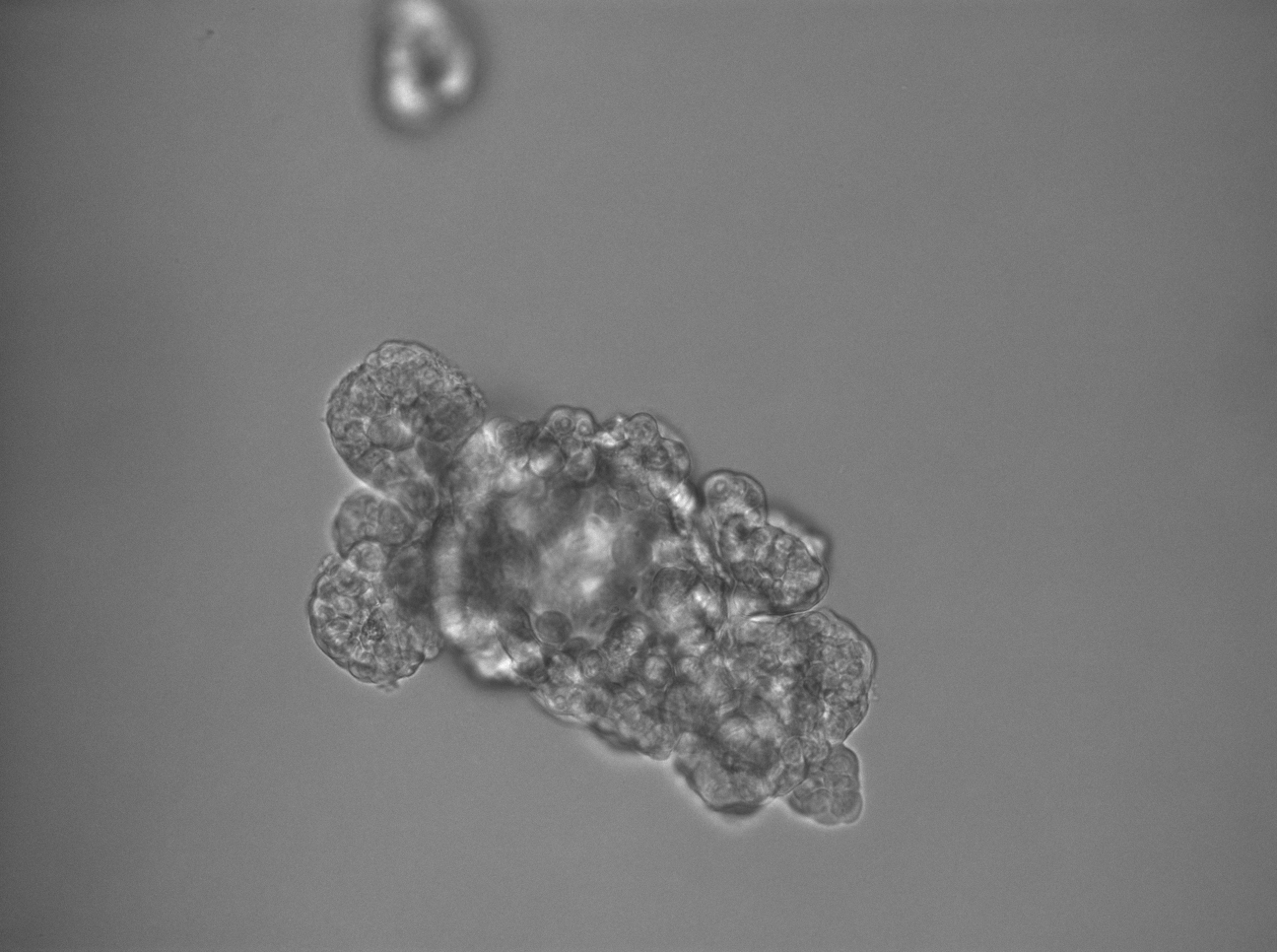

Supplement: Supplementary file 4 — Source Data Fig. 4 [file 41586_2026_10187_MOESM4_ESM.zip › HPDE/HPDE-GFP_D10_Dox-00000_C02_20xe_ch00.jpg]

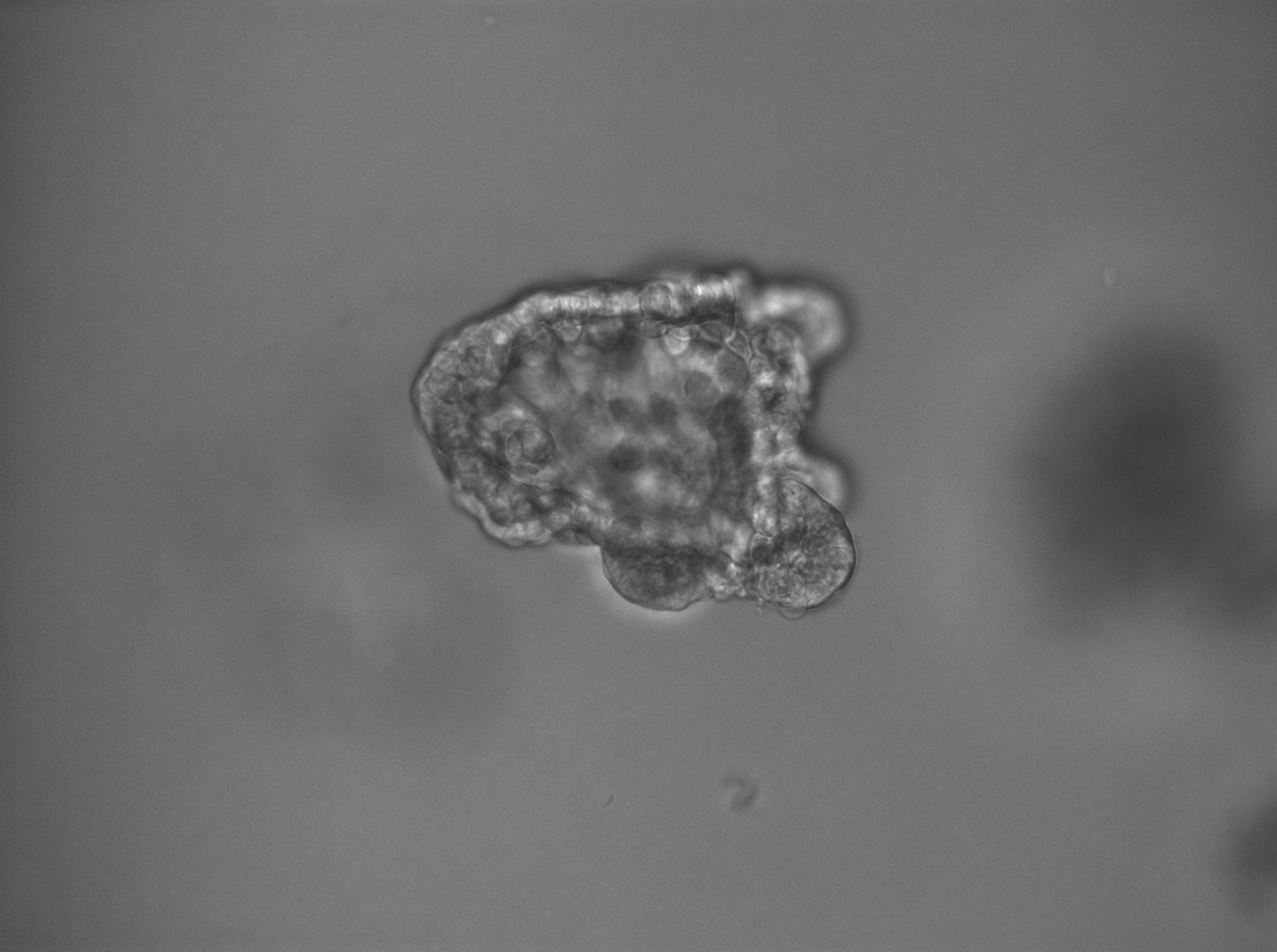

Supplement: Supplementary file 4 — Source Data Fig. 4 [file 41586_2026_10187_MOESM4_ESM.zip › HPDE/HPDE-GFP_D10_Dox-00000_C03_20xa_ch00.jpg]
