# Supplementary figures and images for: A disease model resource reveals core principles of tissue-specific cancer evolution (part 1 of 3)
Source: Nature. 2026 Feb 25;653(8113):57. doi: 10.1038/s41586-026-10187-2 (PMC13149333; doi:10.1038/s41586-026-10187-2)

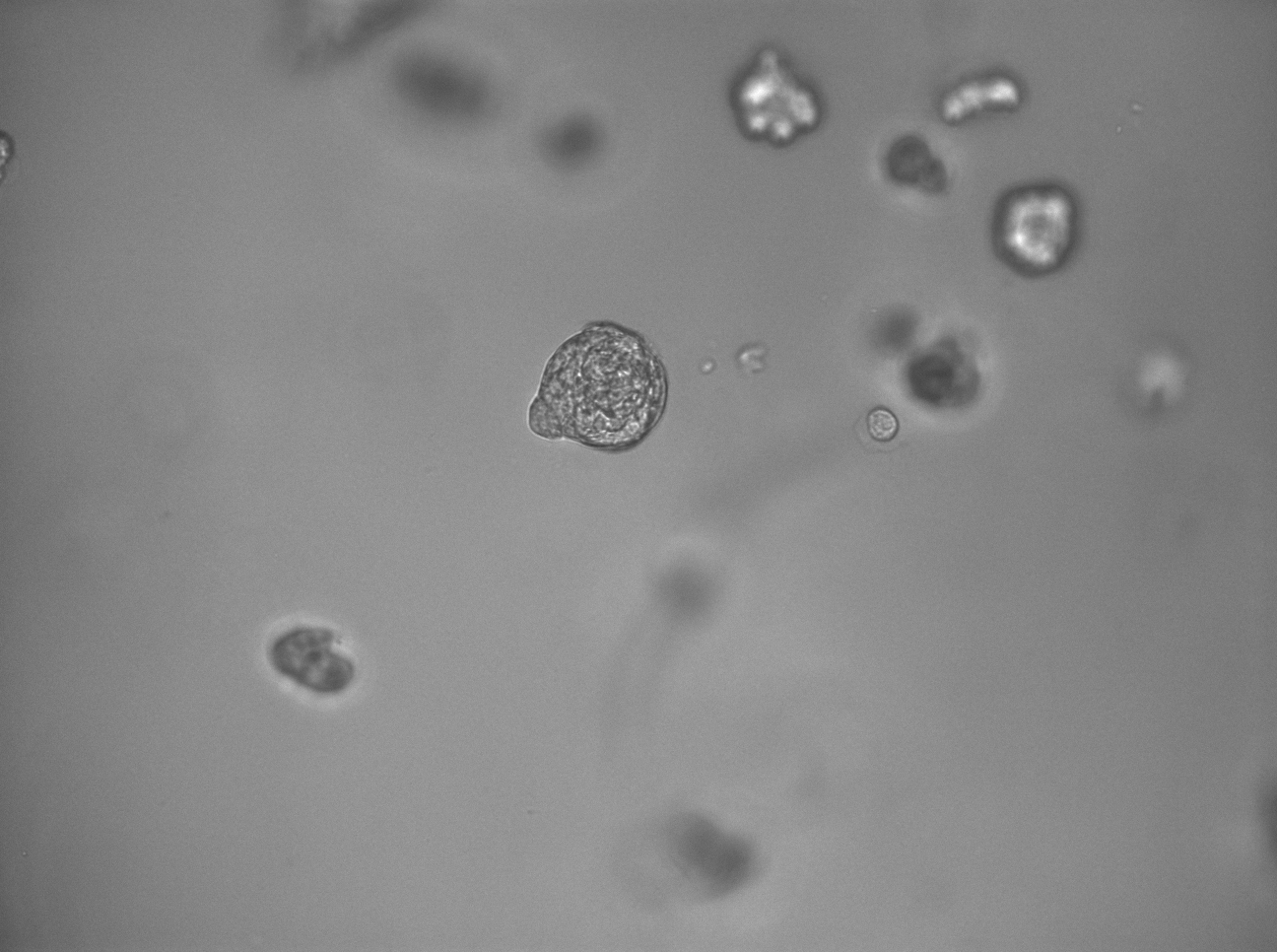

Supplement: Supplementary file 4 — Source Data Fig. 4 [file 41586_2026_10187_MOESM4_ESM.zip › HCEC1CT/HCEC1CT-GFP_D10_Dox-00000_B01a_20x_ch00.jpg]

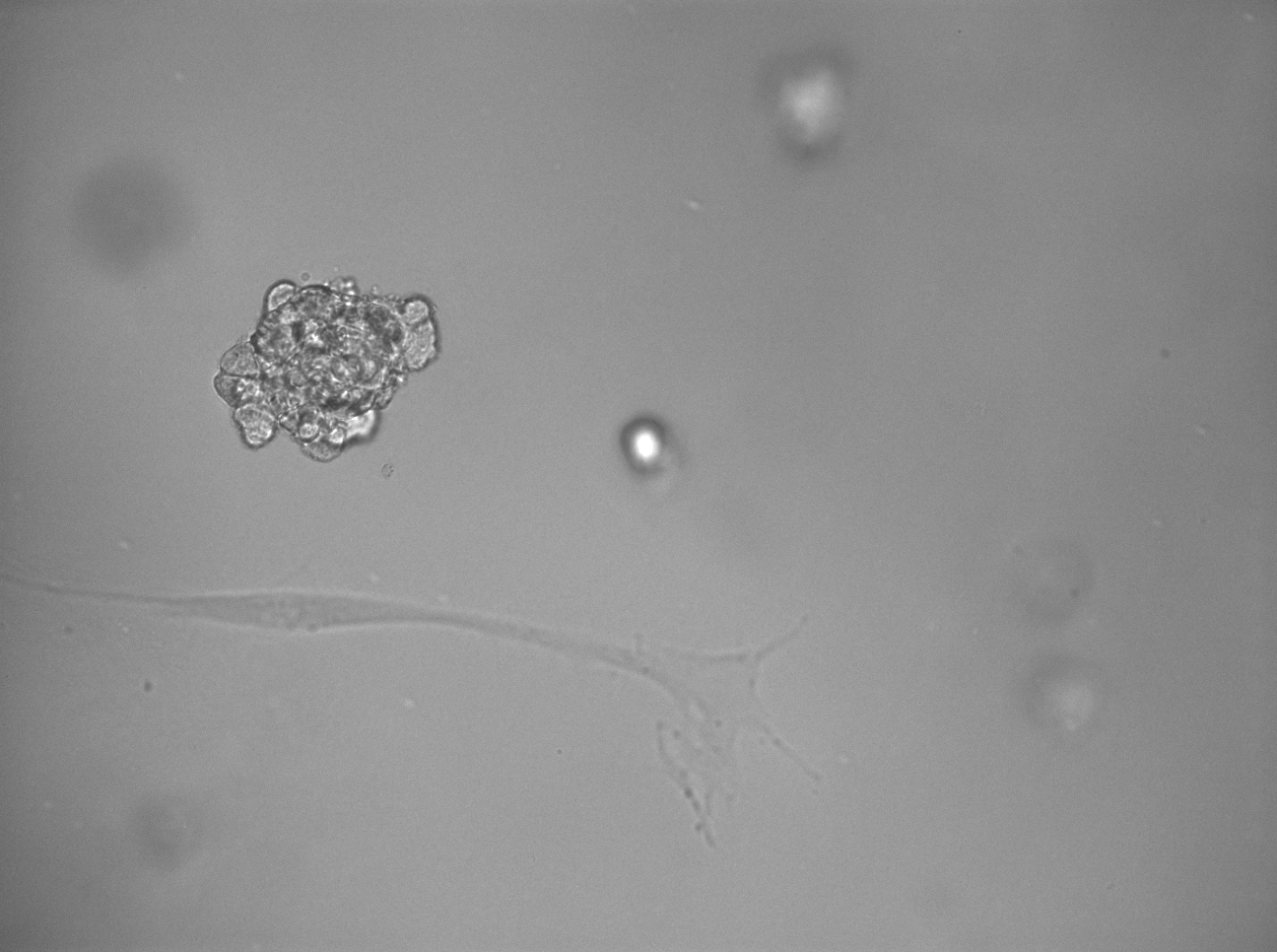

Supplement: Supplementary file 4 — Source Data Fig. 4 [file 41586_2026_10187_MOESM4_ESM.zip › HCEC1CT/HCEC1CT-GFP_D10_Dox-00000_B01b_20x_ch00.jpg]

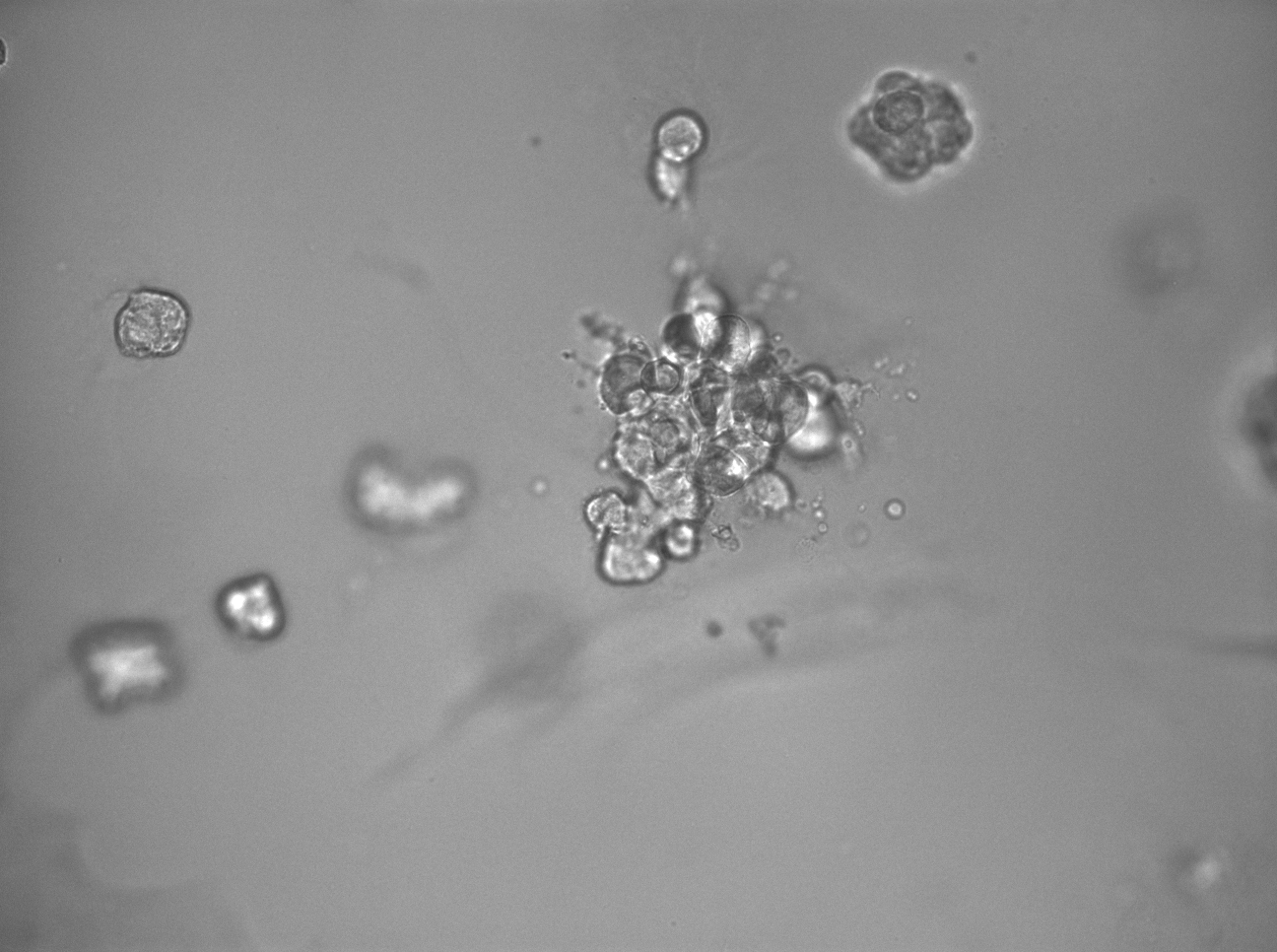

Supplement: Supplementary file 4 — Source Data Fig. 4 [file 41586_2026_10187_MOESM4_ESM.zip › HCEC1CT/HCEC1CT-GFP_D10_Dox-00000_B01c_20x_ch00.jpg]

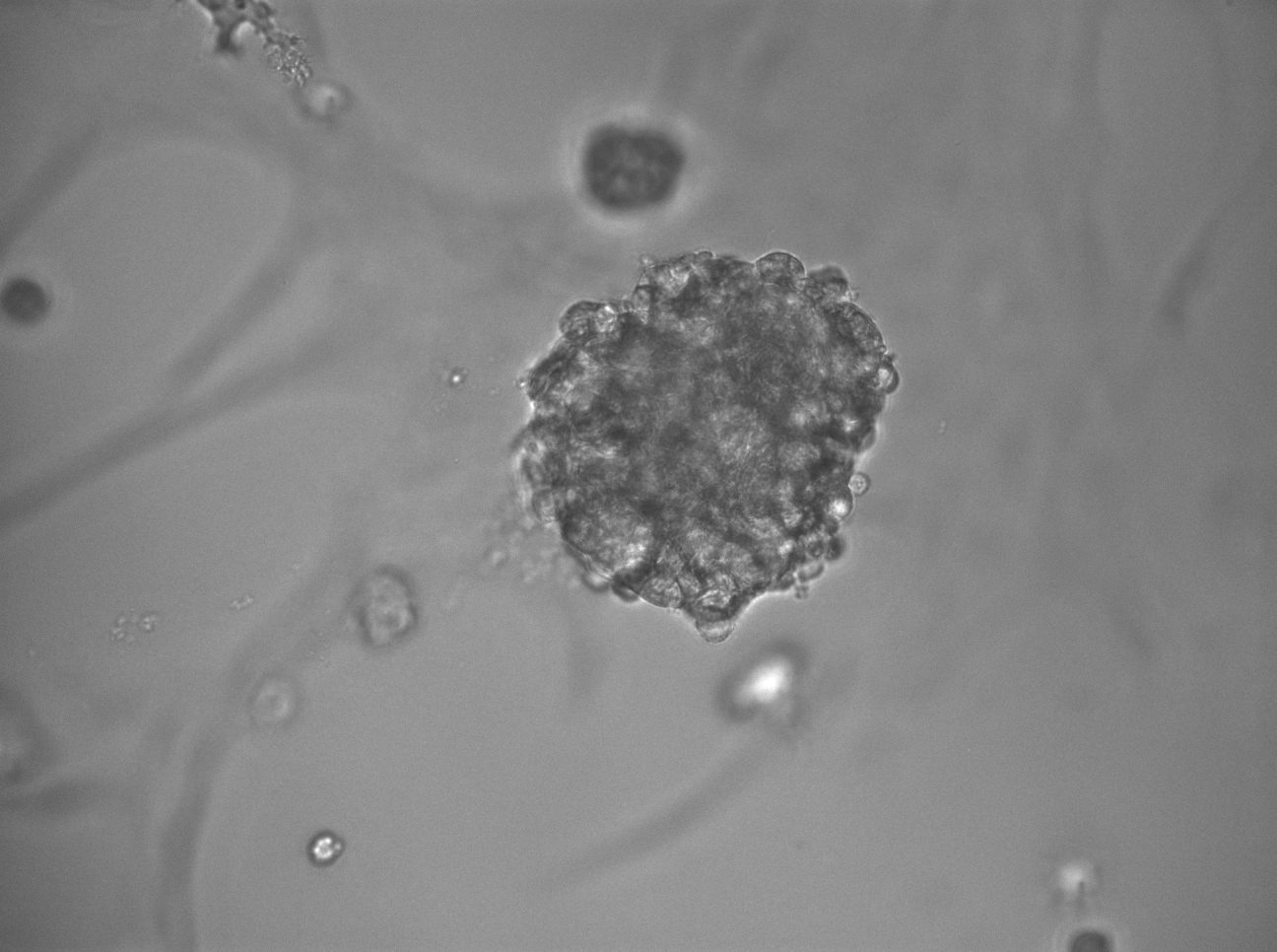

Supplement: Supplementary file 4 — Source Data Fig. 4 [file 41586_2026_10187_MOESM4_ESM.zip › HCEC1CT/HCEC1CT-GFP_D10_Dox-00000_B01d_20x_ch00.jpg]

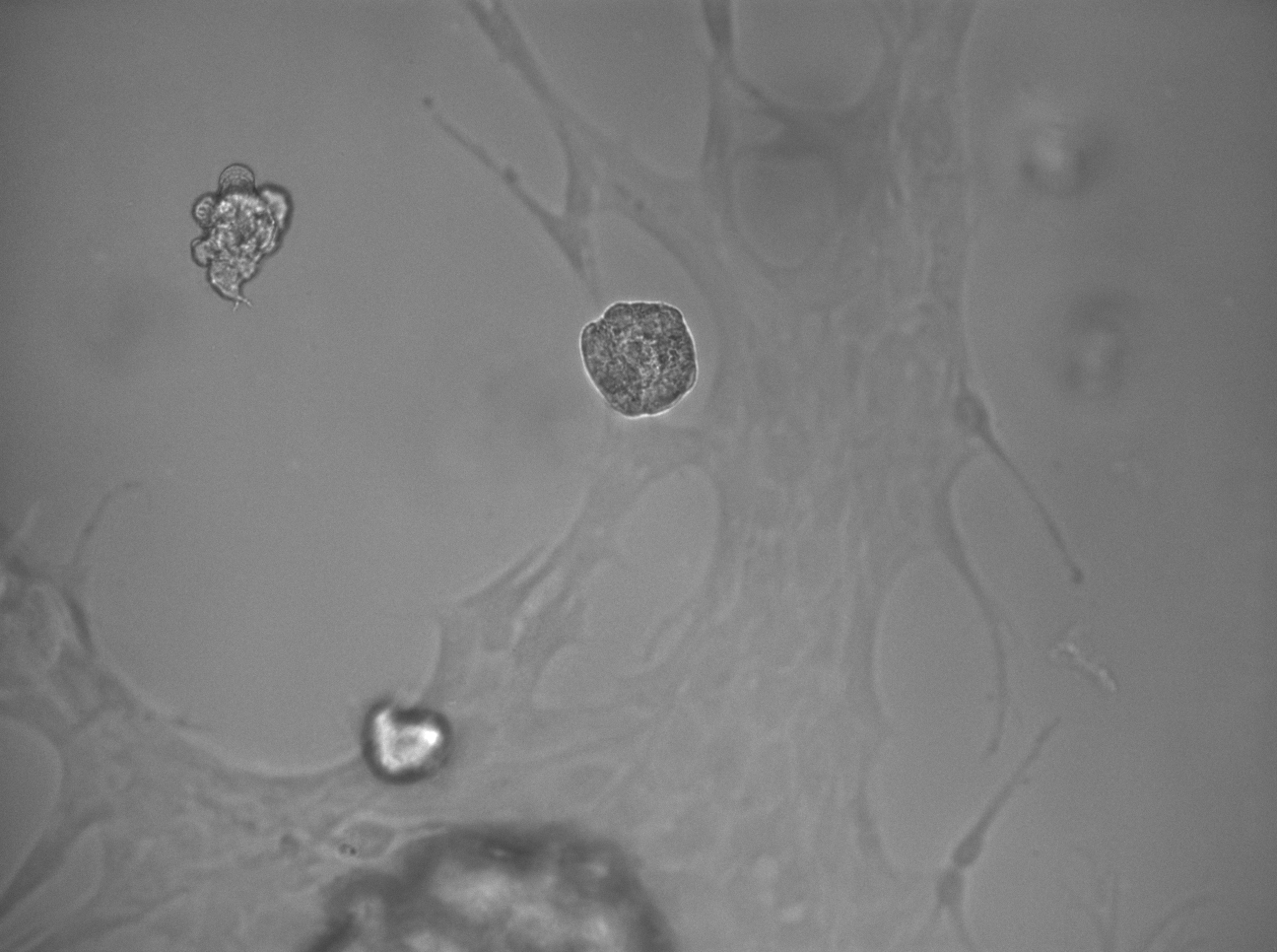

Supplement: Supplementary file 4 — Source Data Fig. 4 [file 41586_2026_10187_MOESM4_ESM.zip › HCEC1CT/HCEC1CT-GFP_D10_Dox-00000_B01e_20x_ch00.jpg]

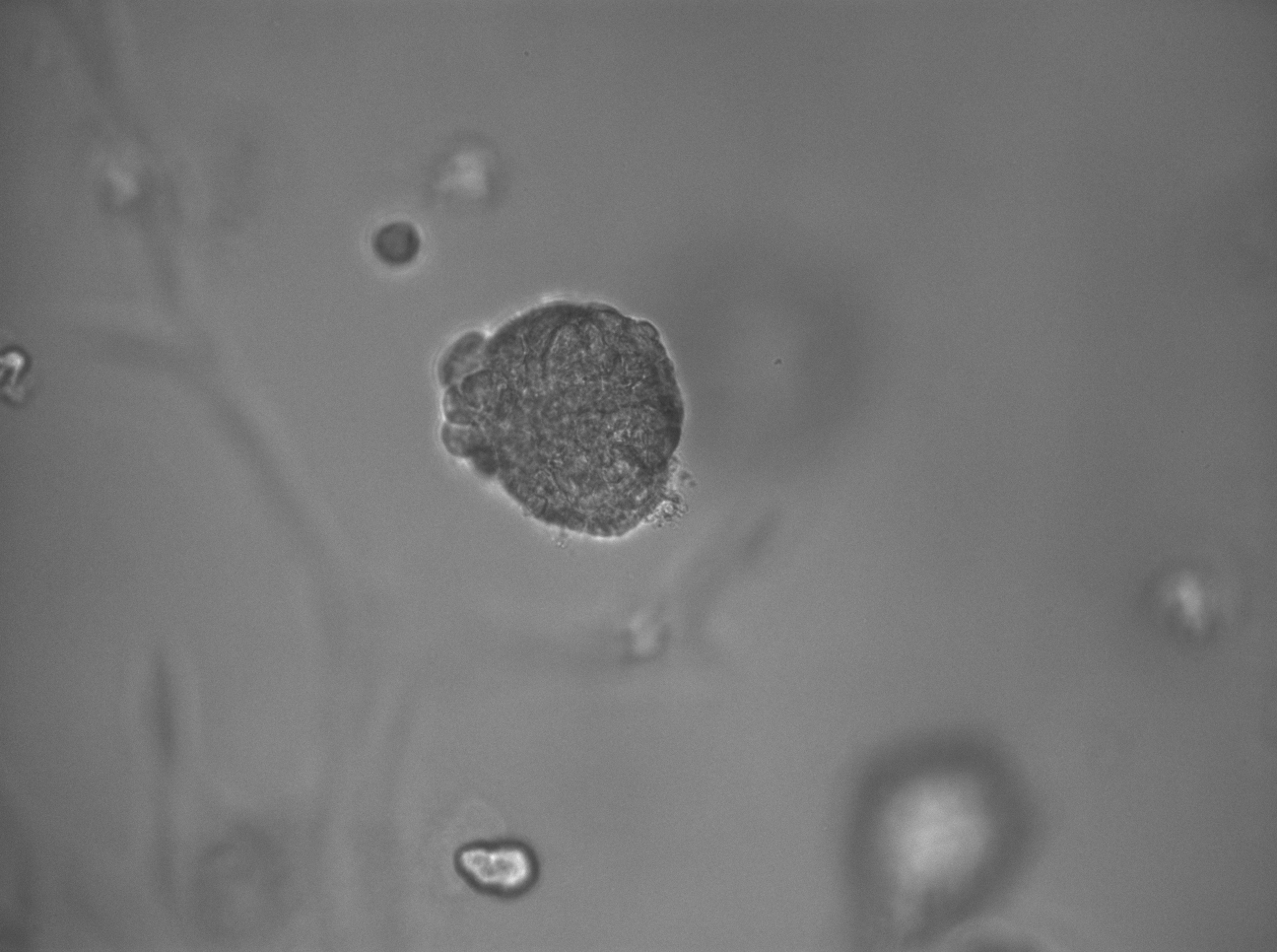

Supplement: Supplementary file 4 — Source Data Fig. 4 [file 41586_2026_10187_MOESM4_ESM.zip › HCEC1CT/HCEC1CT-GFP_D10_Dox-00000_B01f_20x_ch00.jpg]

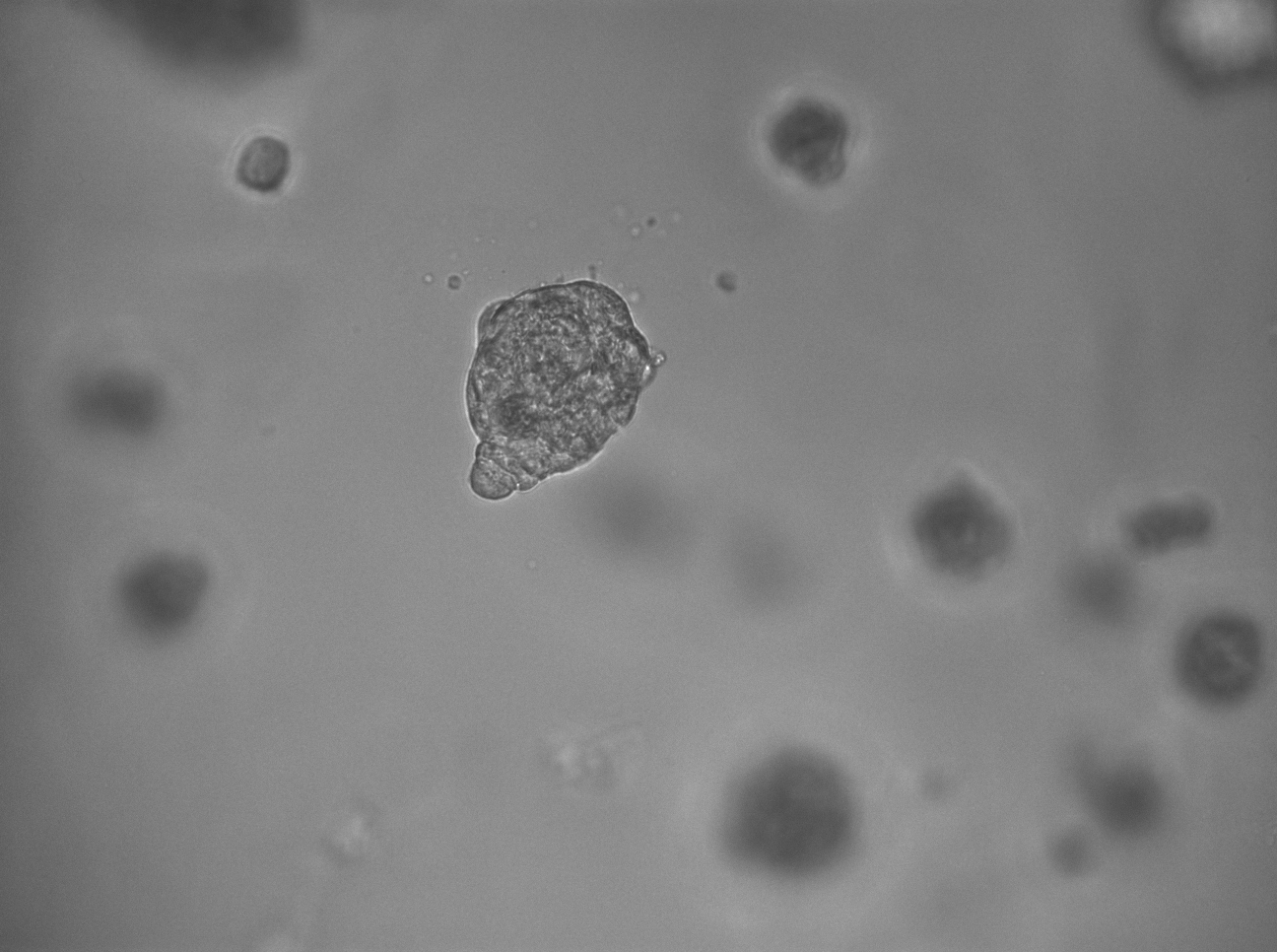

Supplement: Supplementary file 4 — Source Data Fig. 4 [file 41586_2026_10187_MOESM4_ESM.zip › HCEC1CT/HCEC1CT-GFP_D10_Dox-00000_B01g_20x_ch00.jpg]

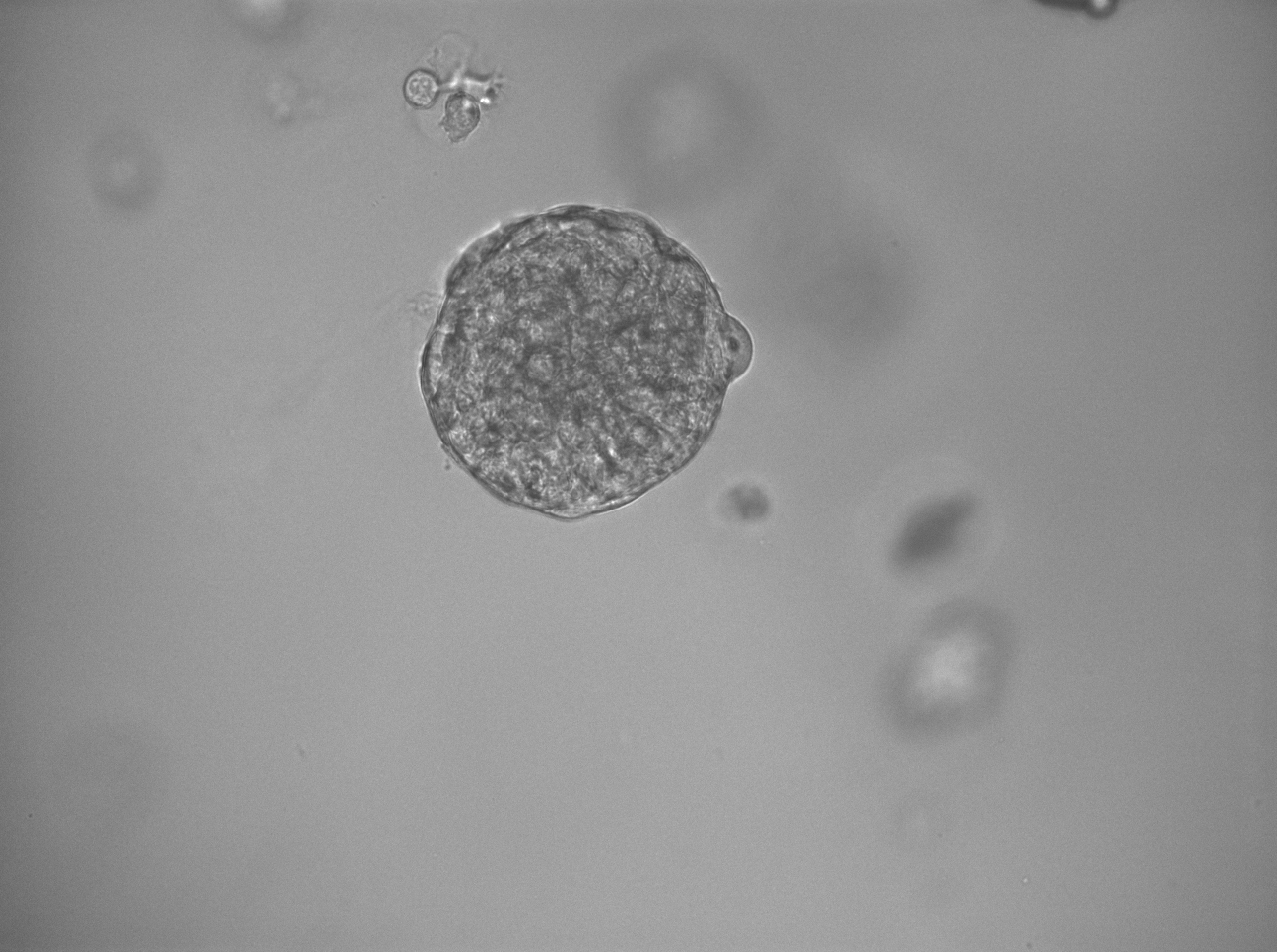

Supplement: Supplementary file 4 — Source Data Fig. 4 [file 41586_2026_10187_MOESM4_ESM.zip › HCEC1CT/HCEC1CT-GFP_D10_Dox-00000_B02a_20x_ch00.jpg]

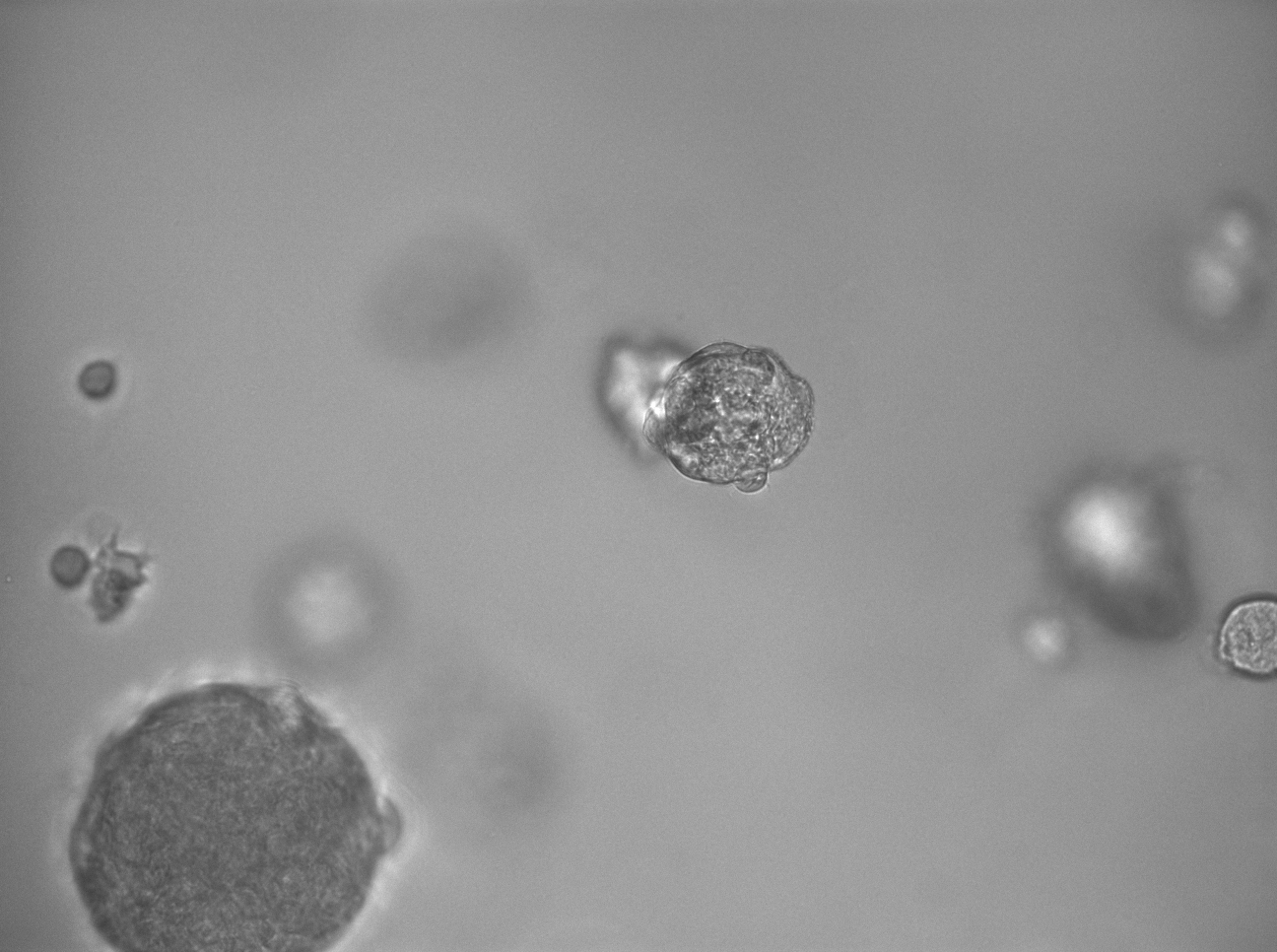

Supplement: Supplementary file 4 — Source Data Fig. 4 [file 41586_2026_10187_MOESM4_ESM.zip › HCEC1CT/HCEC1CT-GFP_D10_Dox-00000_B02b_20x_ch00.jpg]

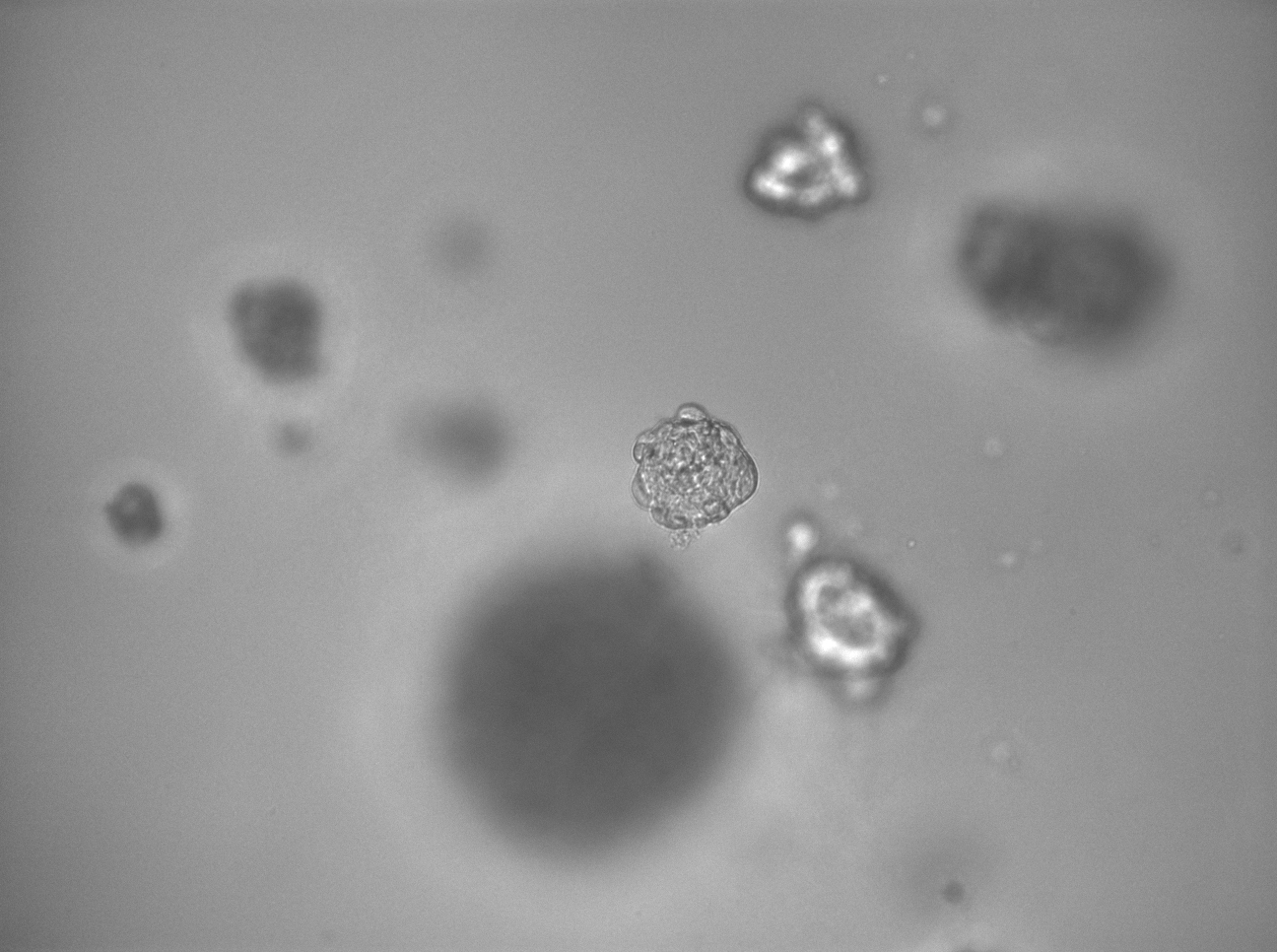

Supplement: Supplementary file 4 — Source Data Fig. 4 [file 41586_2026_10187_MOESM4_ESM.zip › HCEC1CT/HCEC1CT-GFP_D10_Dox-00000_B02c_20x_ch00.jpg]

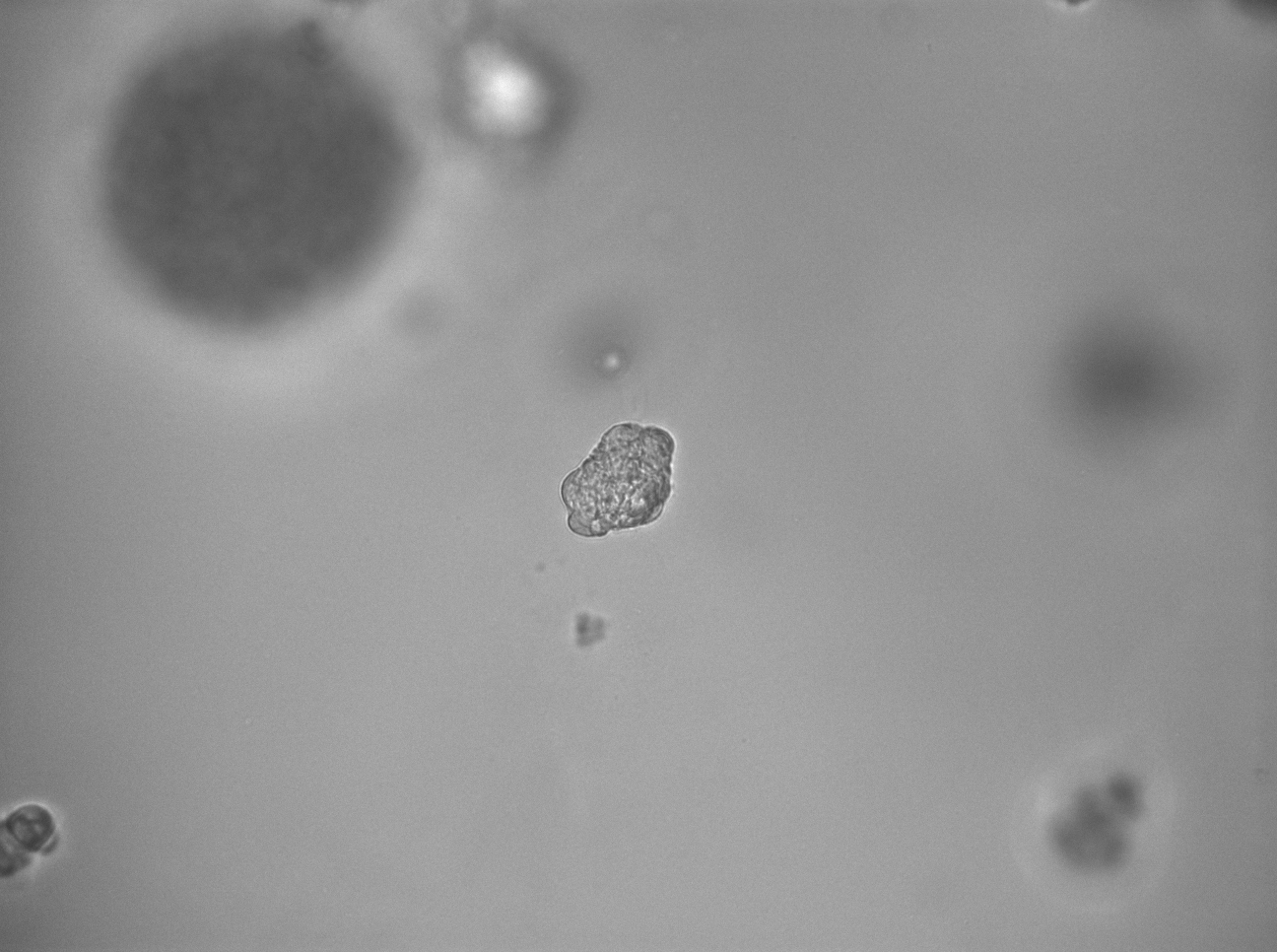

Supplement: Supplementary file 4 — Source Data Fig. 4 [file 41586_2026_10187_MOESM4_ESM.zip › HCEC1CT/HCEC1CT-GFP_D10_Dox-00000_B02d_20x_ch00.jpg]

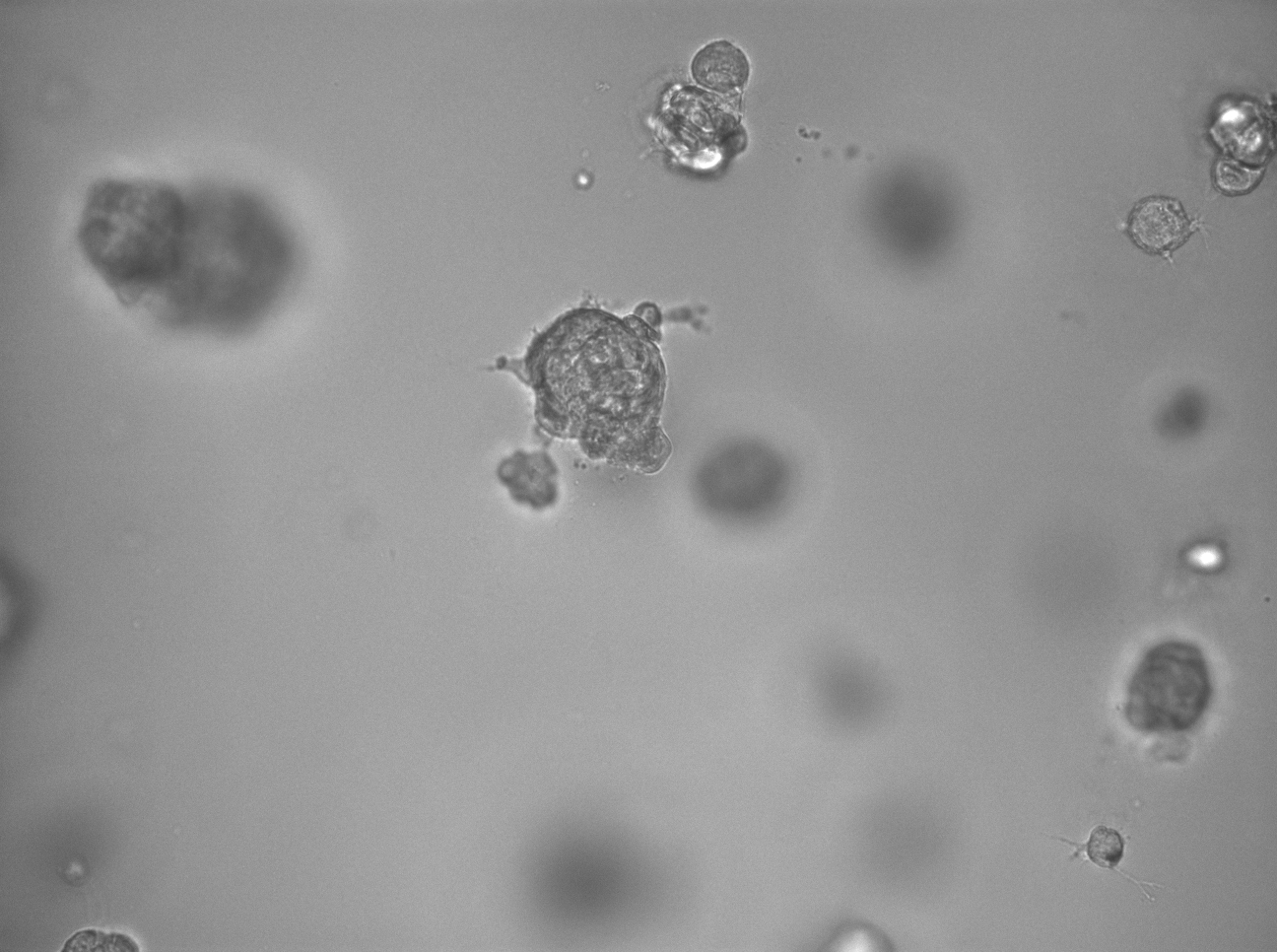

Supplement: Supplementary file 4 — Source Data Fig. 4 [file 41586_2026_10187_MOESM4_ESM.zip › HCEC1CT/HCEC1CT-GFP_D10_Dox-00000_B02e_20x_ch00.jpg]

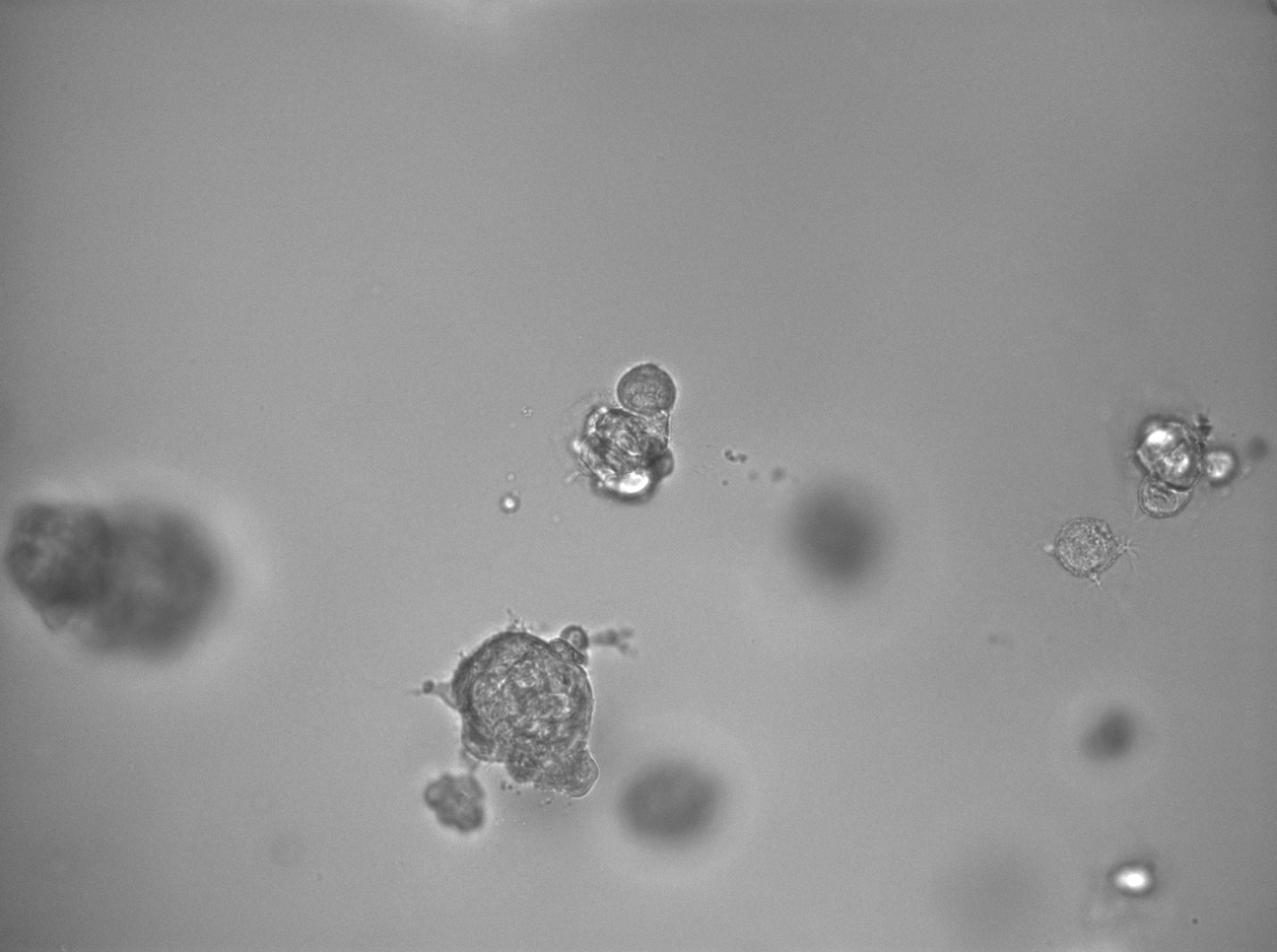

Supplement: Supplementary file 4 — Source Data Fig. 4 [file 41586_2026_10187_MOESM4_ESM.zip › HCEC1CT/HCEC1CT-GFP_D10_Dox-00000_B02f_20x_ch00.jpg]

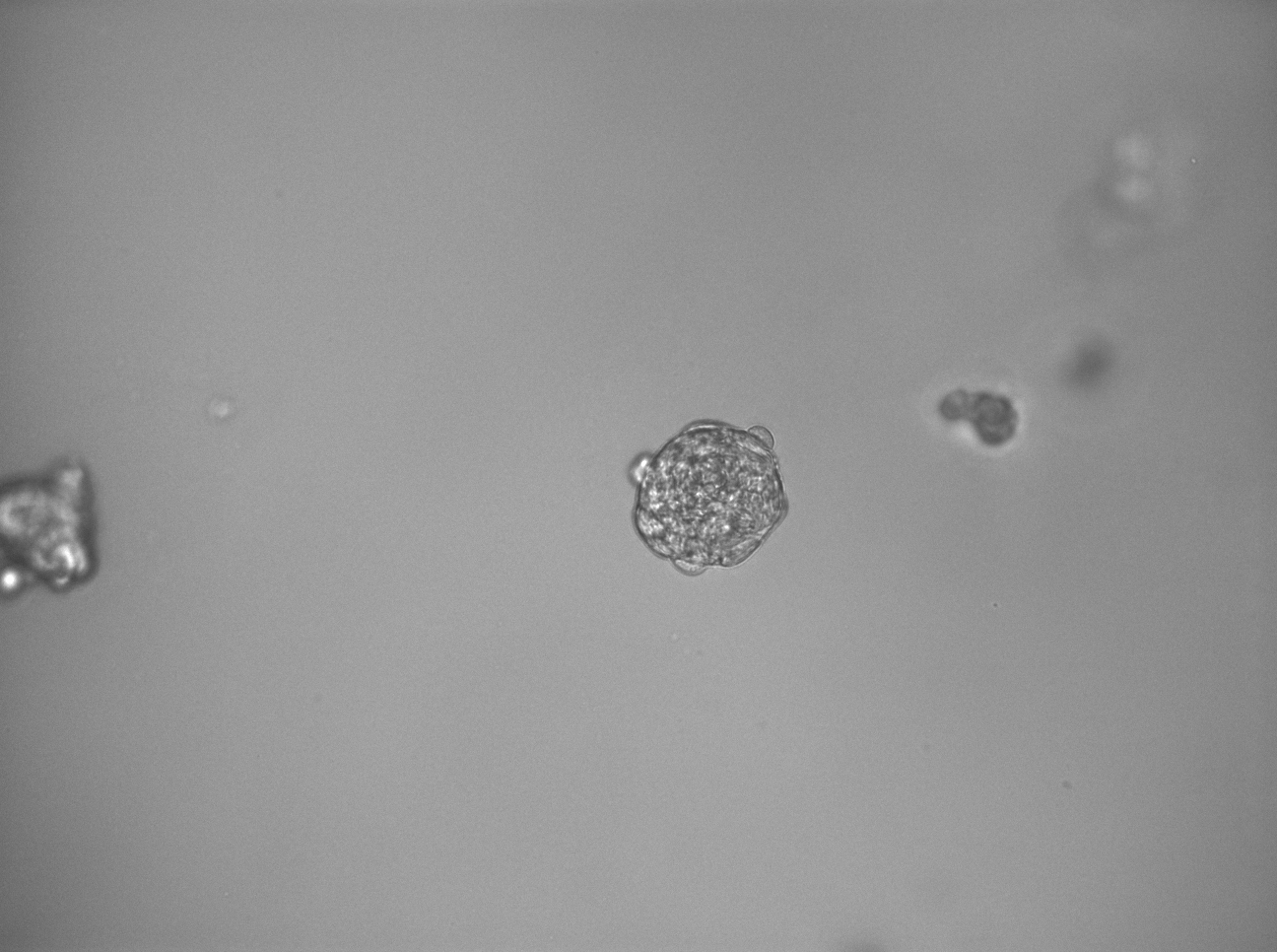

Supplement: Supplementary file 4 — Source Data Fig. 4 [file 41586_2026_10187_MOESM4_ESM.zip › HCEC1CT/HCEC1CT-GFP_D10_Dox-00000_B02g_20x_ch00.jpg]

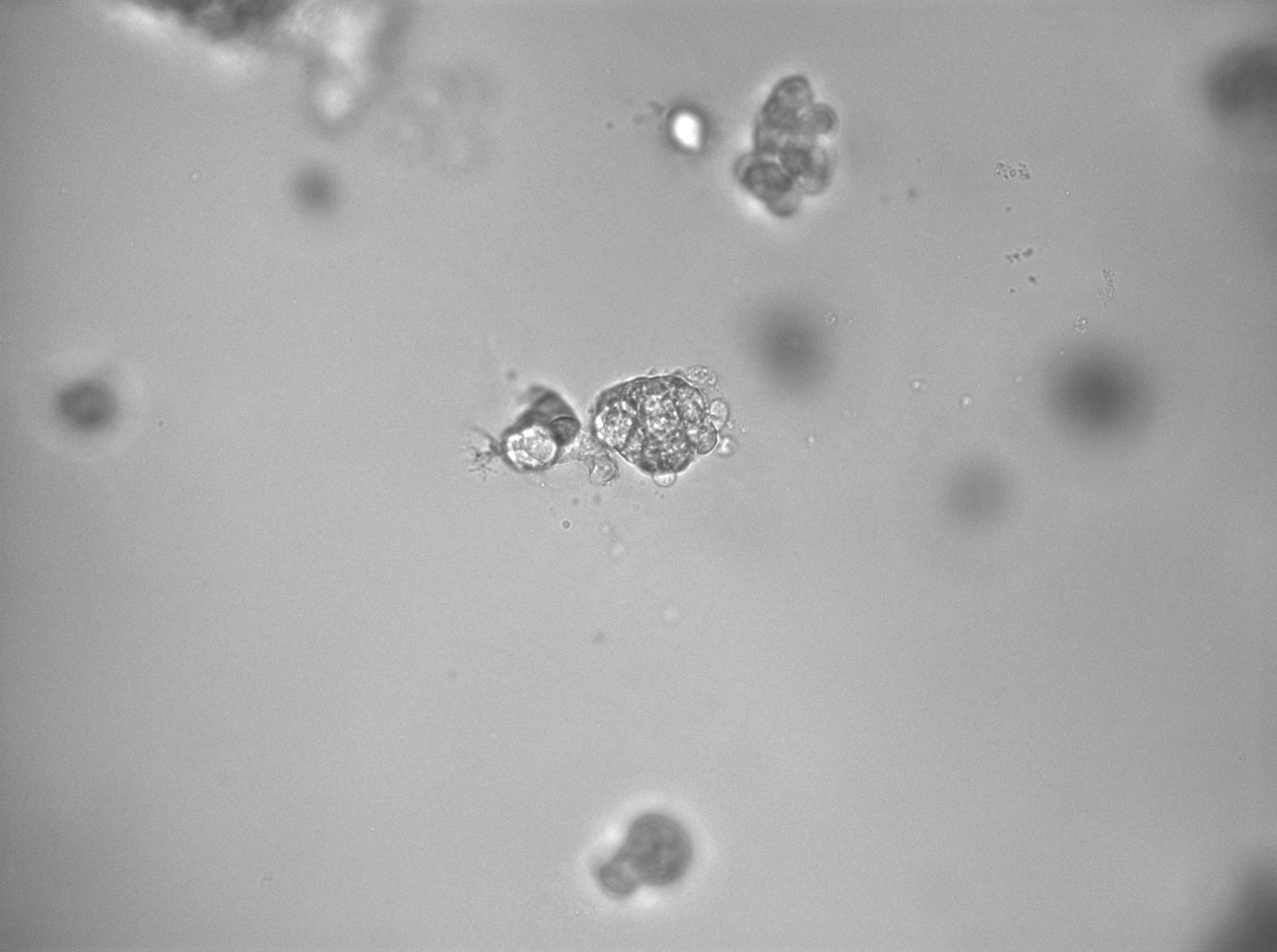

Supplement: Supplementary file 4 — Source Data Fig. 4 [file 41586_2026_10187_MOESM4_ESM.zip › HCEC1CT/HCEC1CT-GFP_D10_Dox-00000_B03a_20x_ch00.jpg]

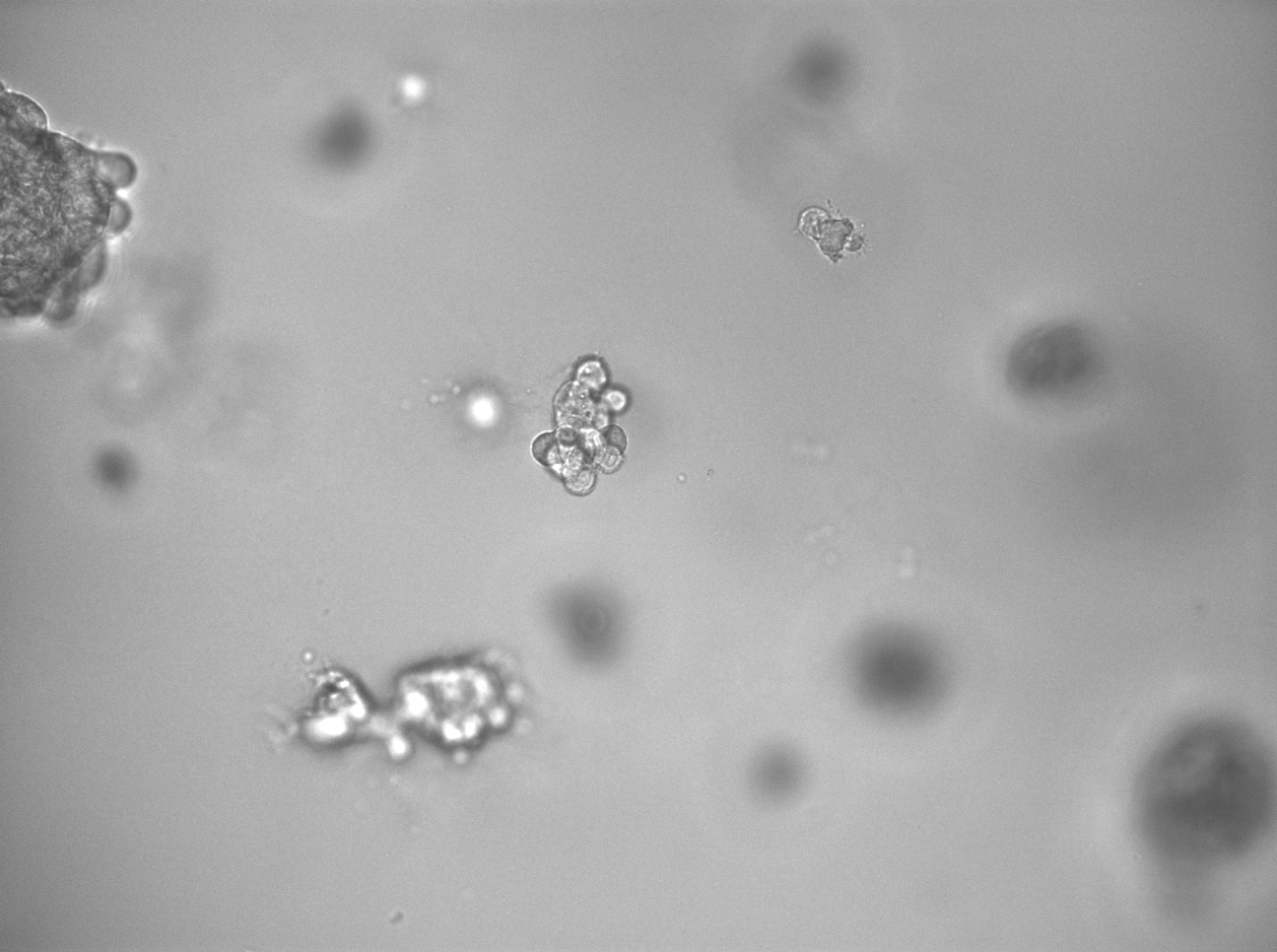

Supplement: Supplementary file 4 — Source Data Fig. 4 [file 41586_2026_10187_MOESM4_ESM.zip › HCEC1CT/HCEC1CT-GFP_D10_Dox-00000_B03b_20x_ch00.jpg]

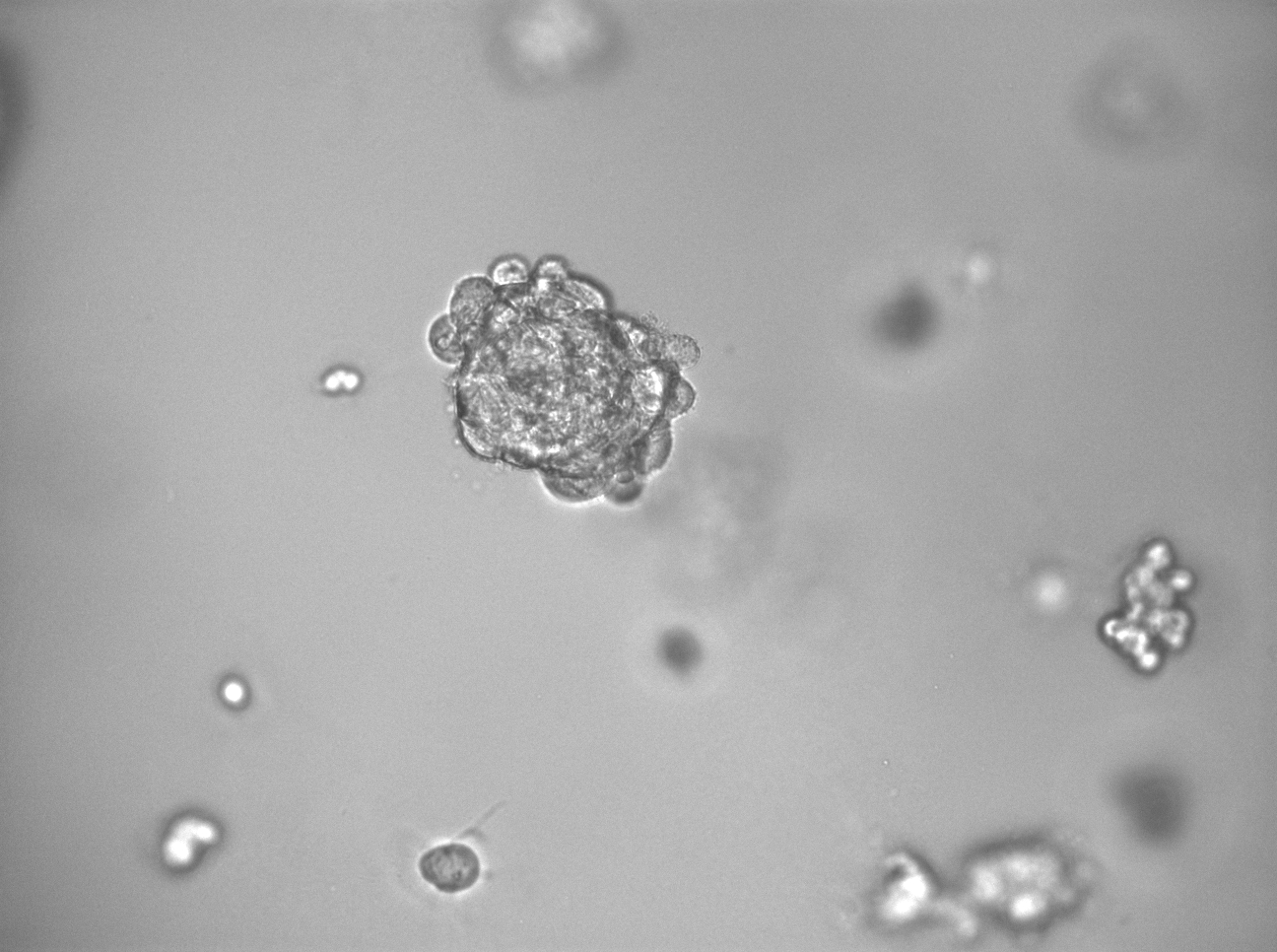

Supplement: Supplementary file 4 — Source Data Fig. 4 [file 41586_2026_10187_MOESM4_ESM.zip › HCEC1CT/HCEC1CT-GFP_D10_Dox-00000_B03c_20x_ch00.jpg]

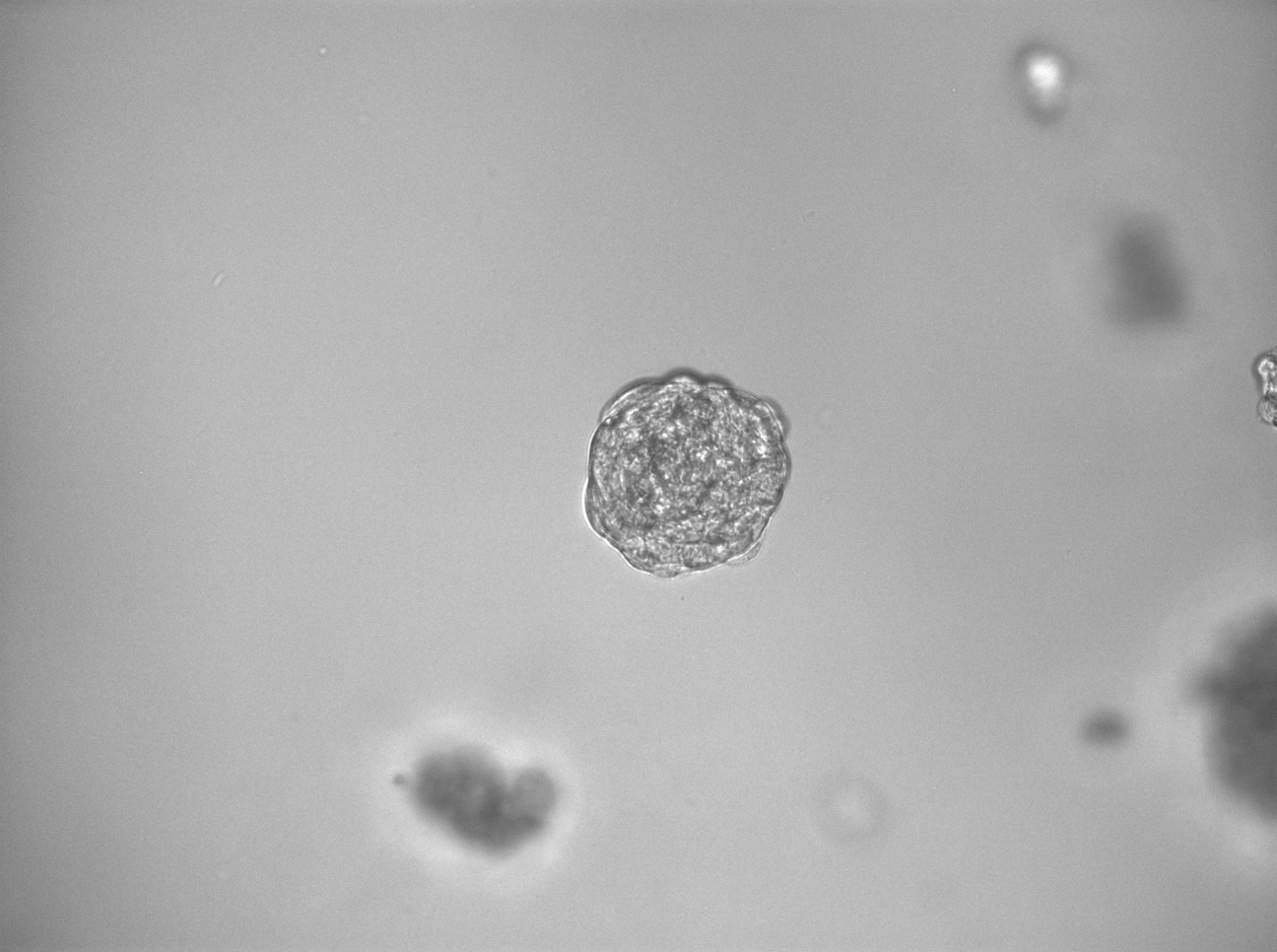

Supplement: Supplementary file 4 — Source Data Fig. 4 [file 41586_2026_10187_MOESM4_ESM.zip › HCEC1CT/HCEC1CT-GFP_D10_Dox-00000_B03d_20x_ch00.jpg]

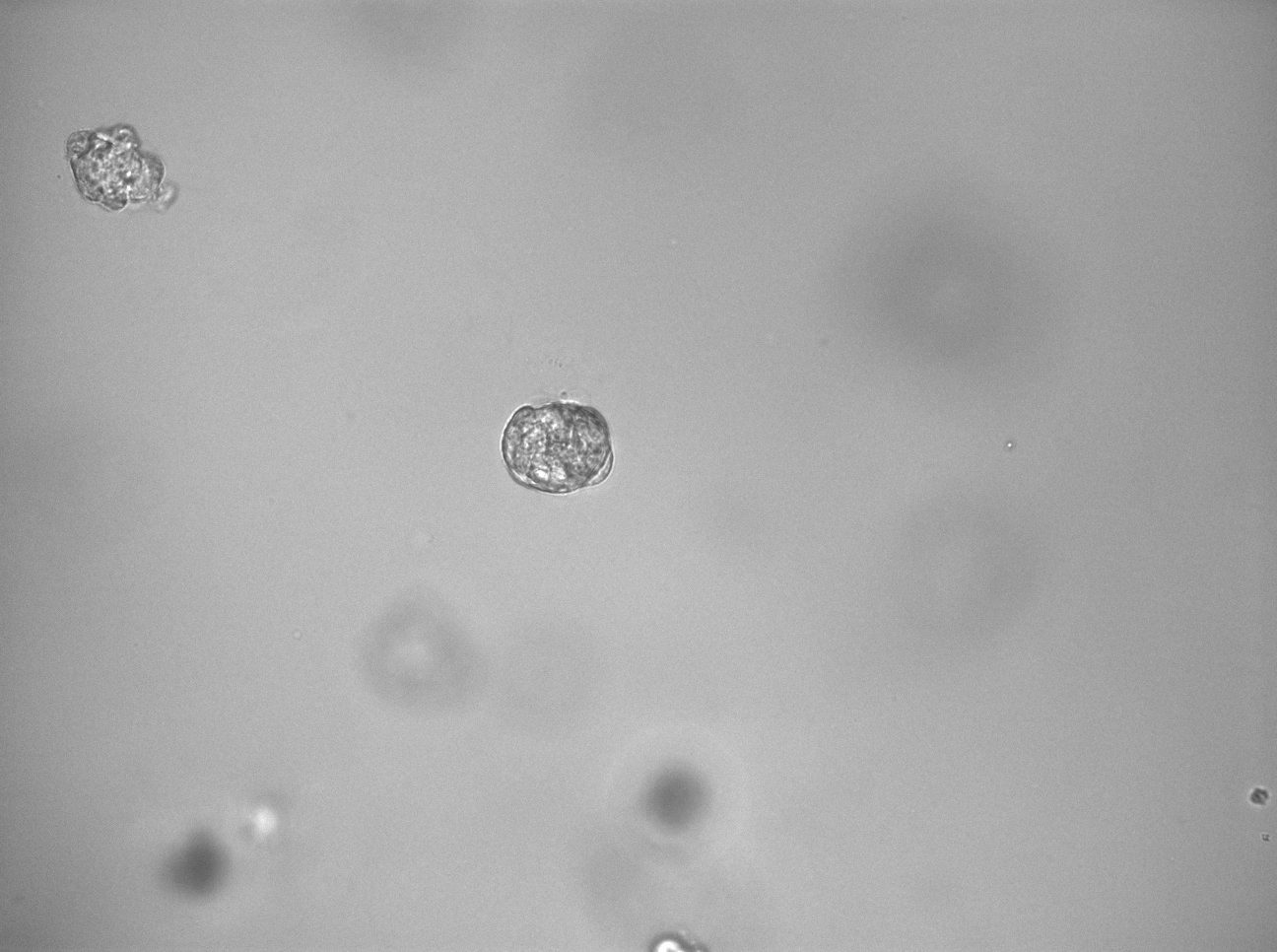

Supplement: Supplementary file 4 — Source Data Fig. 4 [file 41586_2026_10187_MOESM4_ESM.zip › HCEC1CT/HCEC1CT-GFP_D10_Dox-00000_B03e_20x_ch00.jpg]

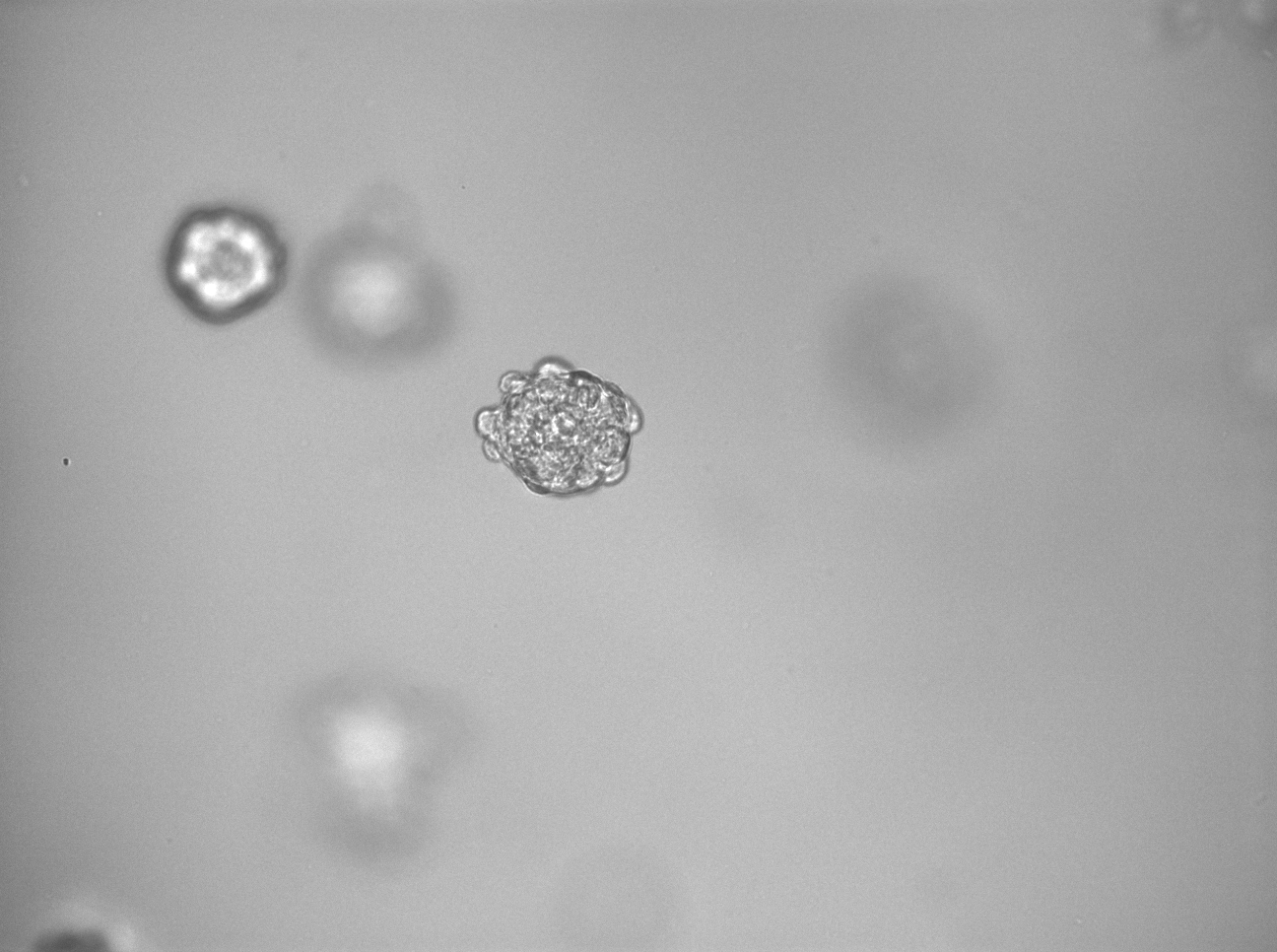

Supplement: Supplementary file 4 — Source Data Fig. 4 [file 41586_2026_10187_MOESM4_ESM.zip › HCEC1CT/HCEC1CT-GFP_D10_Dox-00000_B03f_20x_ch00.jpg]

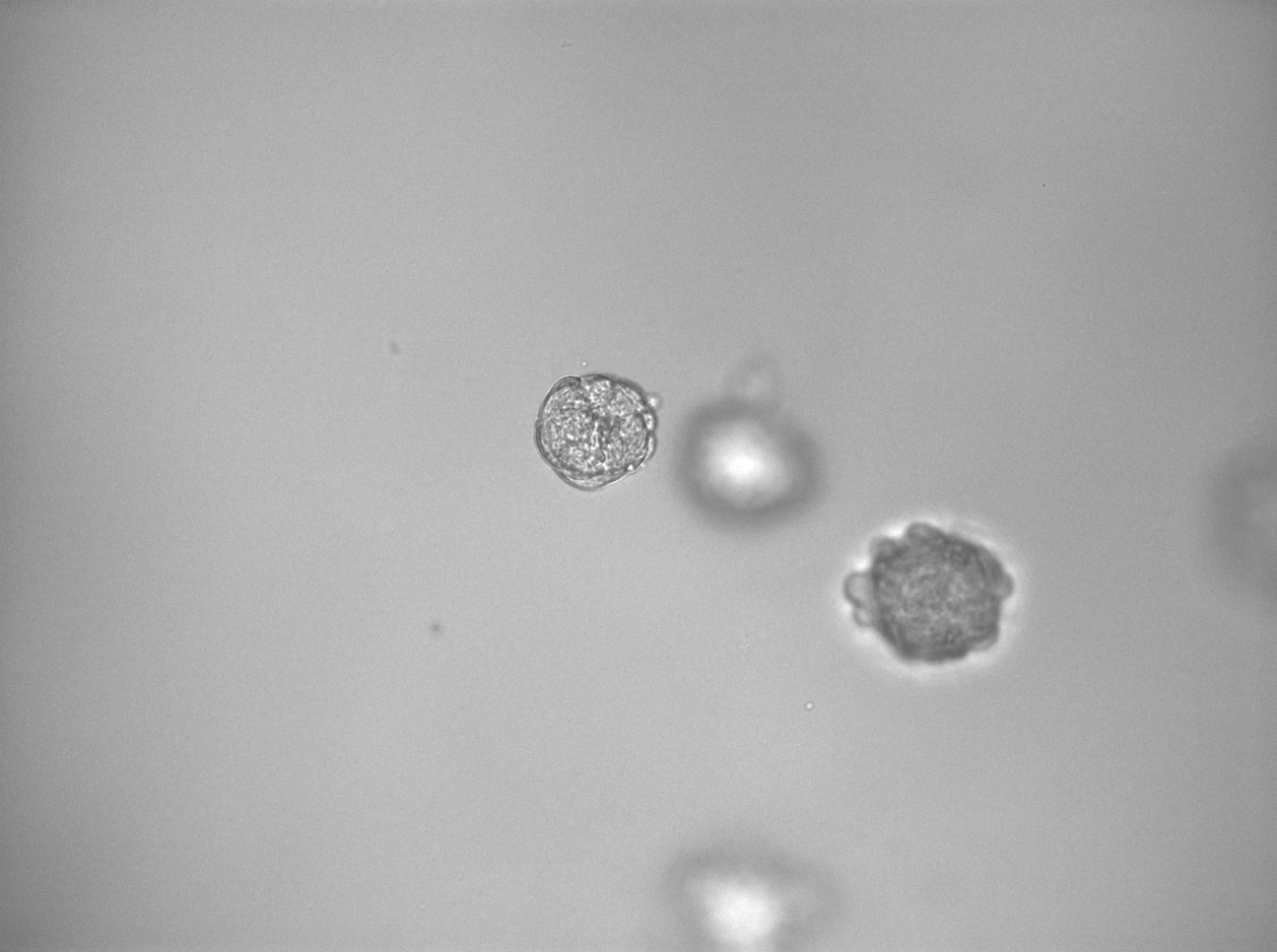

Supplement: Supplementary file 4 — Source Data Fig. 4 [file 41586_2026_10187_MOESM4_ESM.zip › HCEC1CT/HCEC1CT-GFP_D10_Dox-00000_B03g_20x_ch00.jpg]

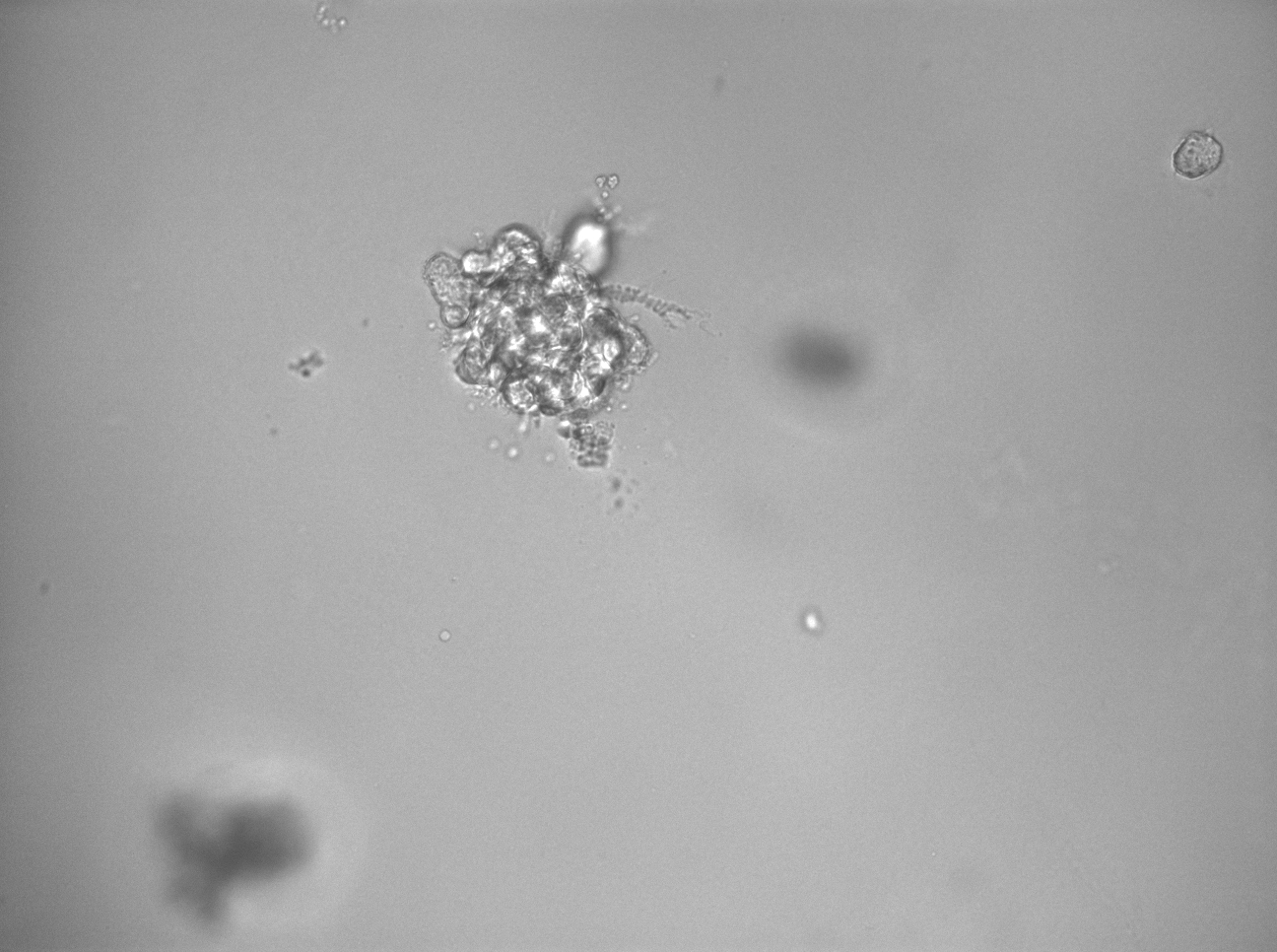

Supplement: Supplementary file 4 — Source Data Fig. 4 [file 41586_2026_10187_MOESM4_ESM.zip › HCEC1CT/HCEC1CT-GFP_D10_Dox-02000_F01a_20x_ch00.jpg]

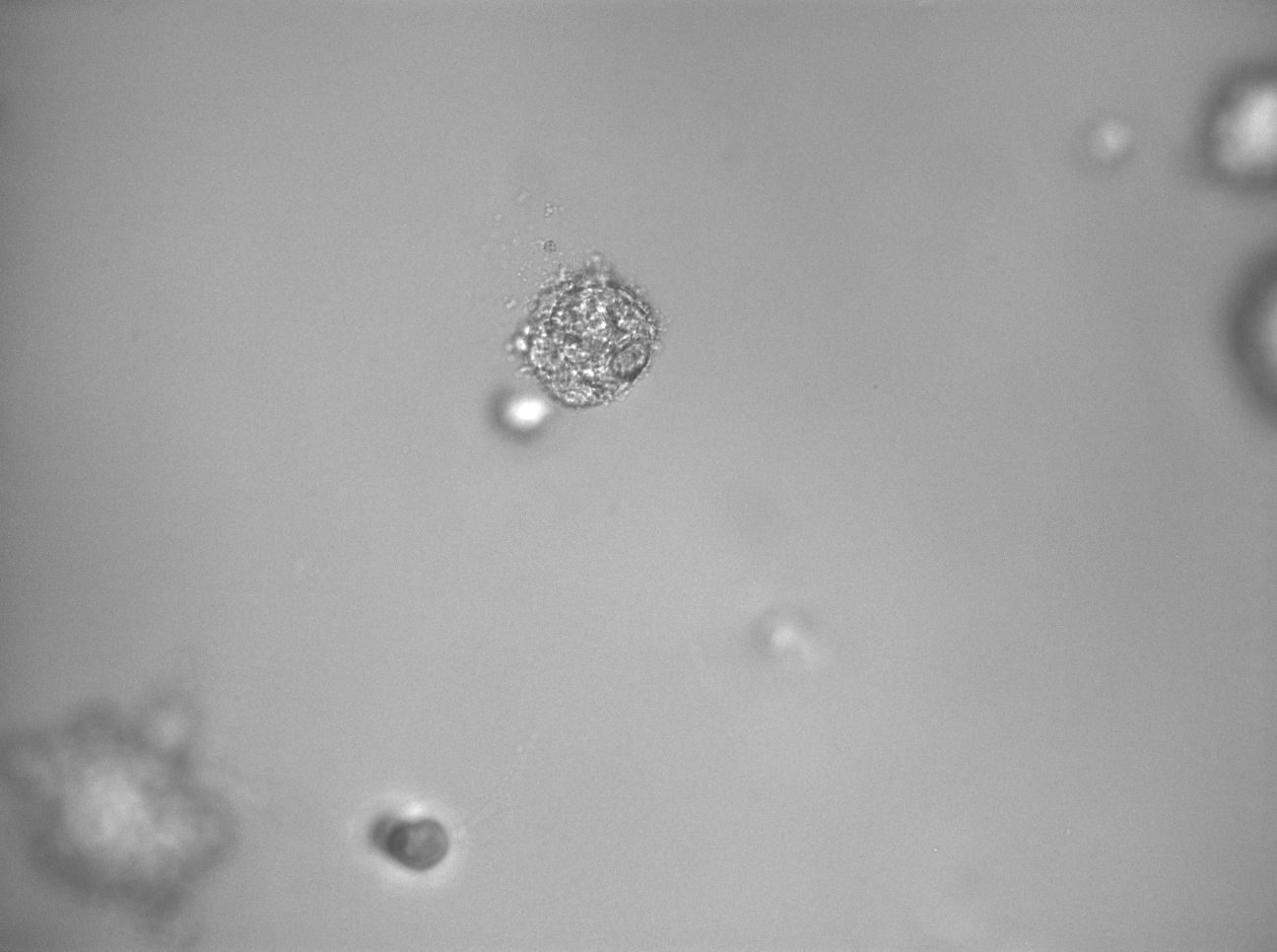

Supplement: Supplementary file 4 — Source Data Fig. 4 [file 41586_2026_10187_MOESM4_ESM.zip › HCEC1CT/HCEC1CT-GFP_D10_Dox-02000_F01b_20x_ch00.jpg]

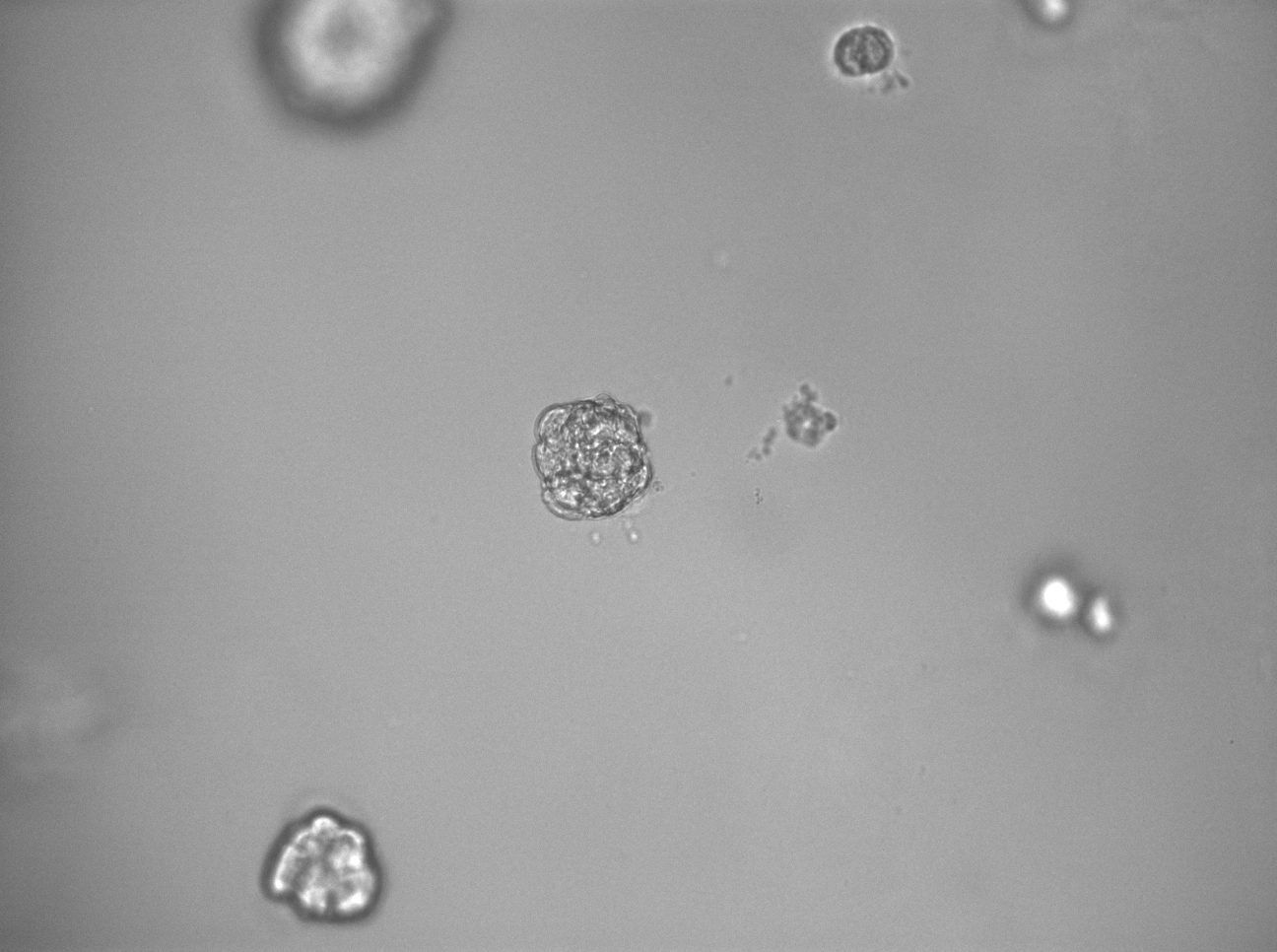

Supplement: Supplementary file 4 — Source Data Fig. 4 [file 41586_2026_10187_MOESM4_ESM.zip › HCEC1CT/HCEC1CT-GFP_D10_Dox-02000_F01c_20x_ch00.jpg]

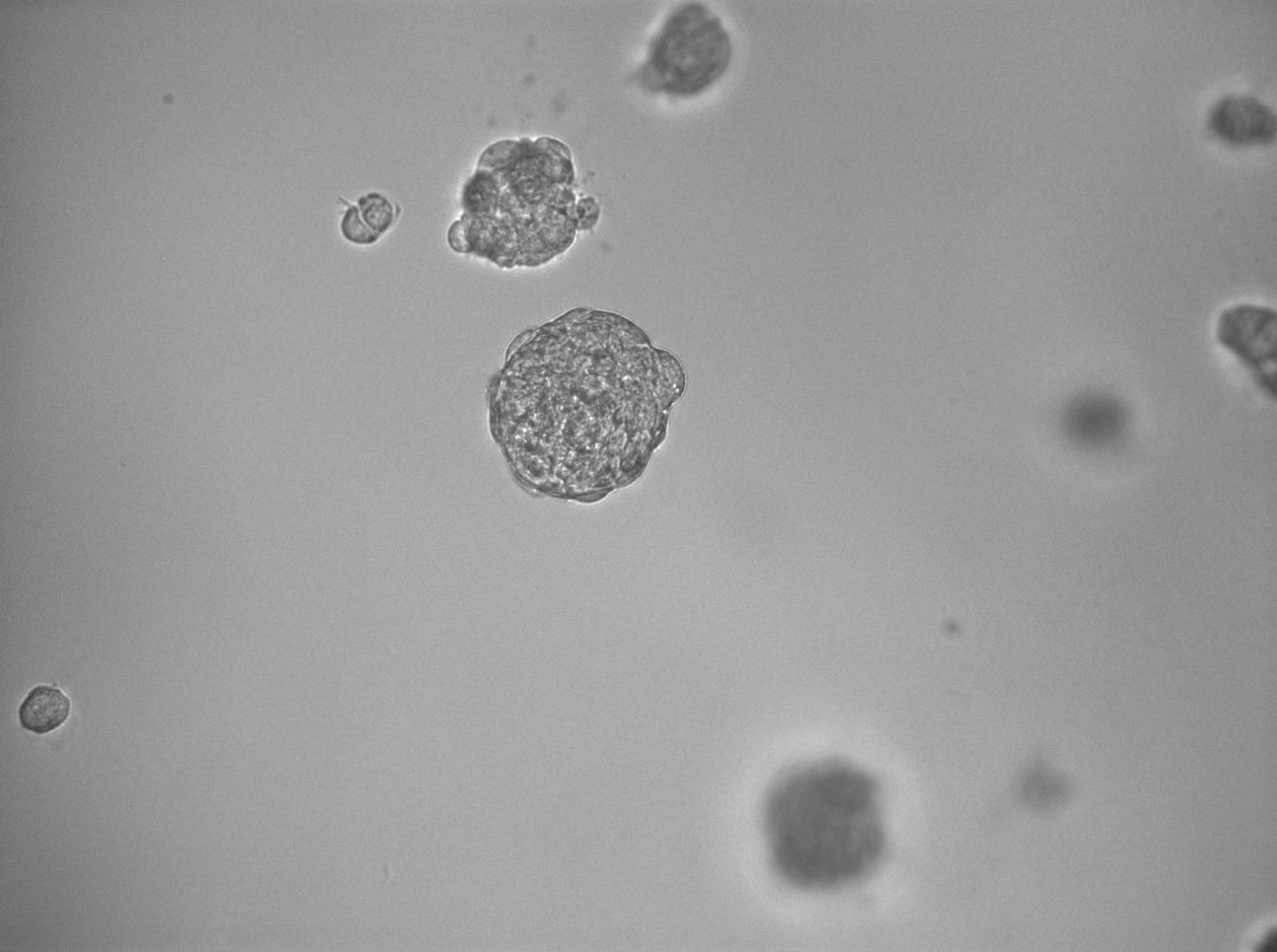

Supplement: Supplementary file 4 — Source Data Fig. 4 [file 41586_2026_10187_MOESM4_ESM.zip › HCEC1CT/HCEC1CT-GFP_D10_Dox-02000_F01d_20x_ch00.jpg]

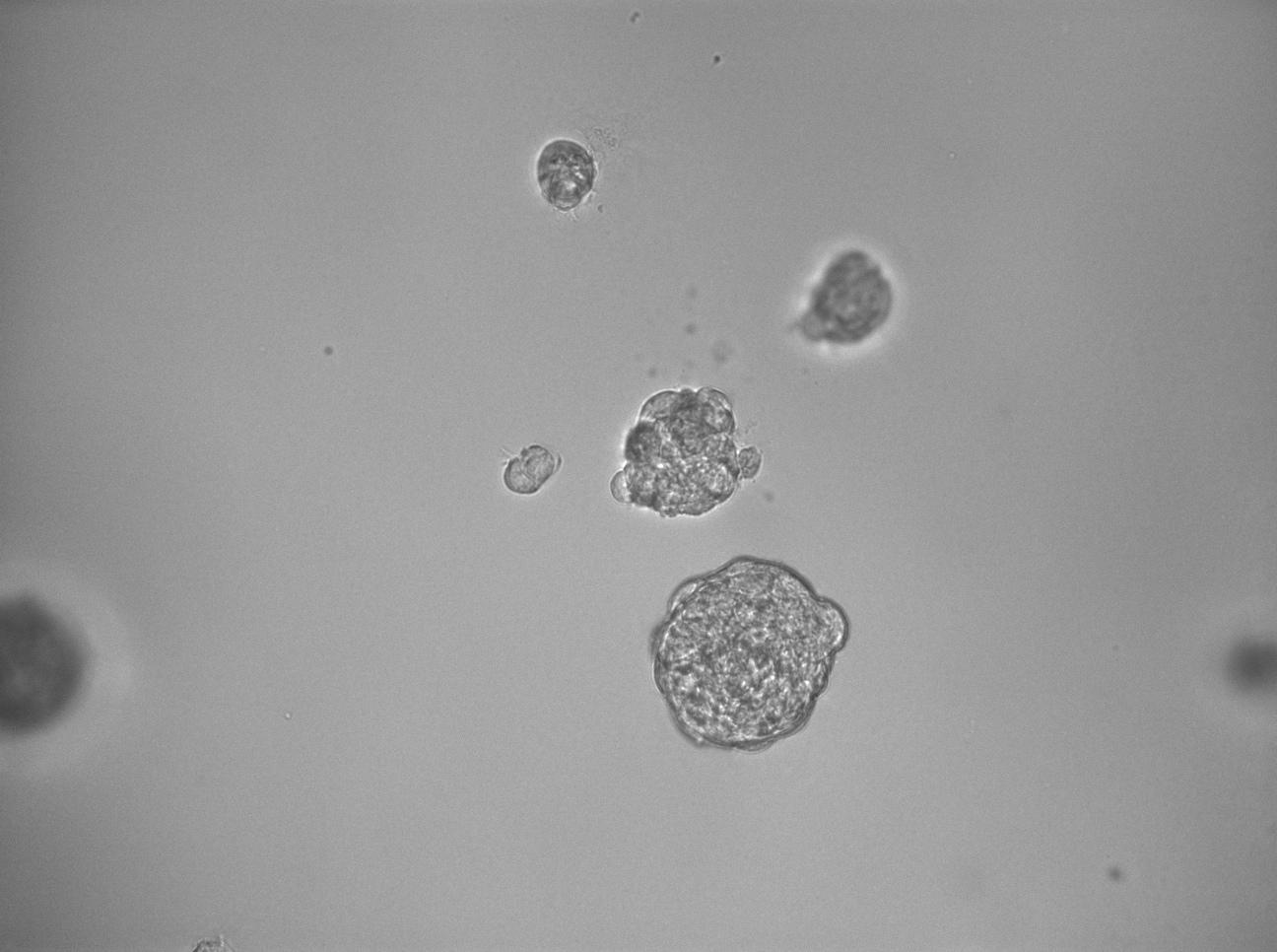

Supplement: Supplementary file 4 — Source Data Fig. 4 [file 41586_2026_10187_MOESM4_ESM.zip › HCEC1CT/HCEC1CT-GFP_D10_Dox-02000_F01e_20x_ch00.jpg]

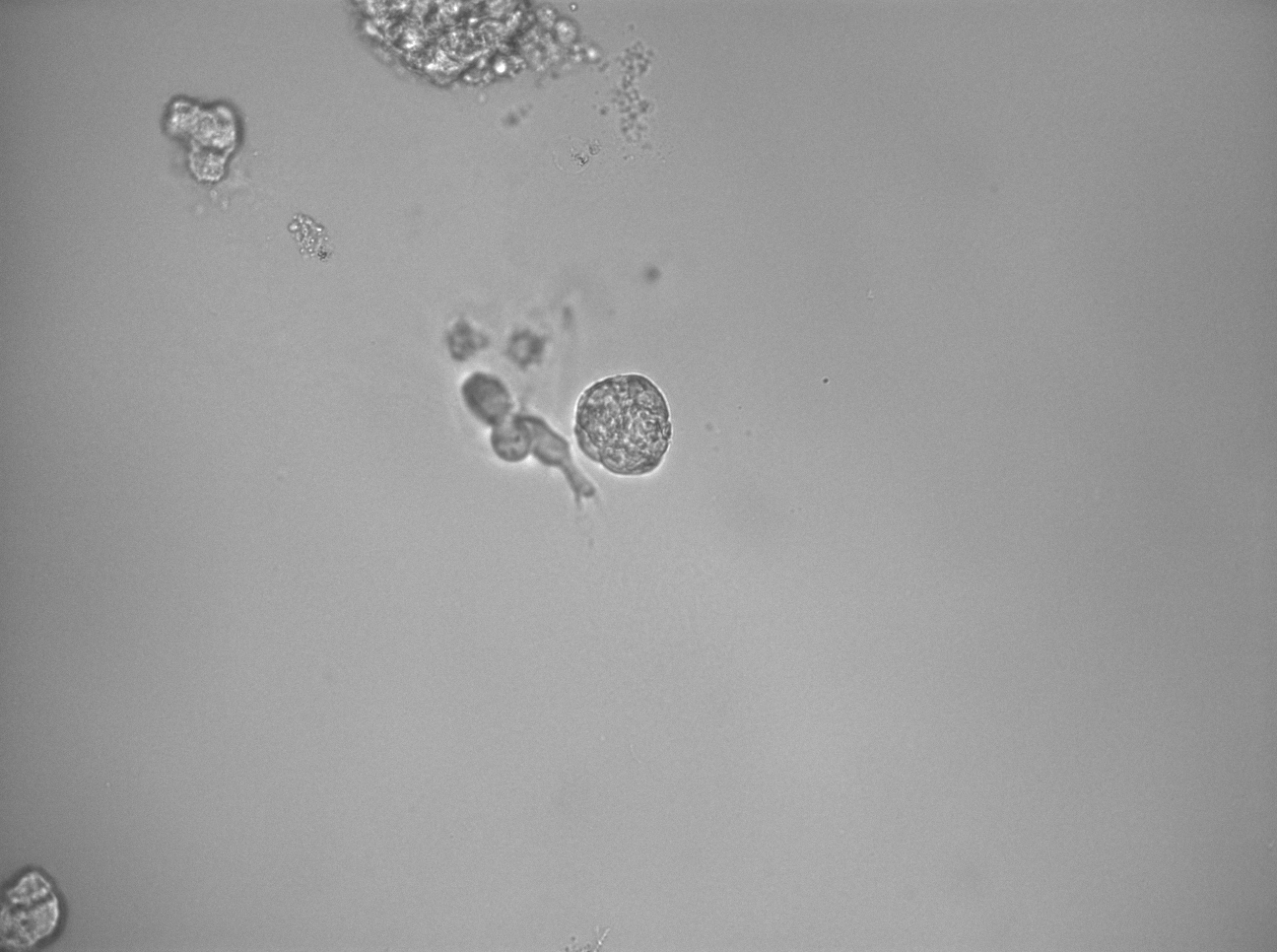

Supplement: Supplementary file 4 — Source Data Fig. 4 [file 41586_2026_10187_MOESM4_ESM.zip › HCEC1CT/HCEC1CT-GFP_D10_Dox-02000_F01f_20x_ch00.jpg]

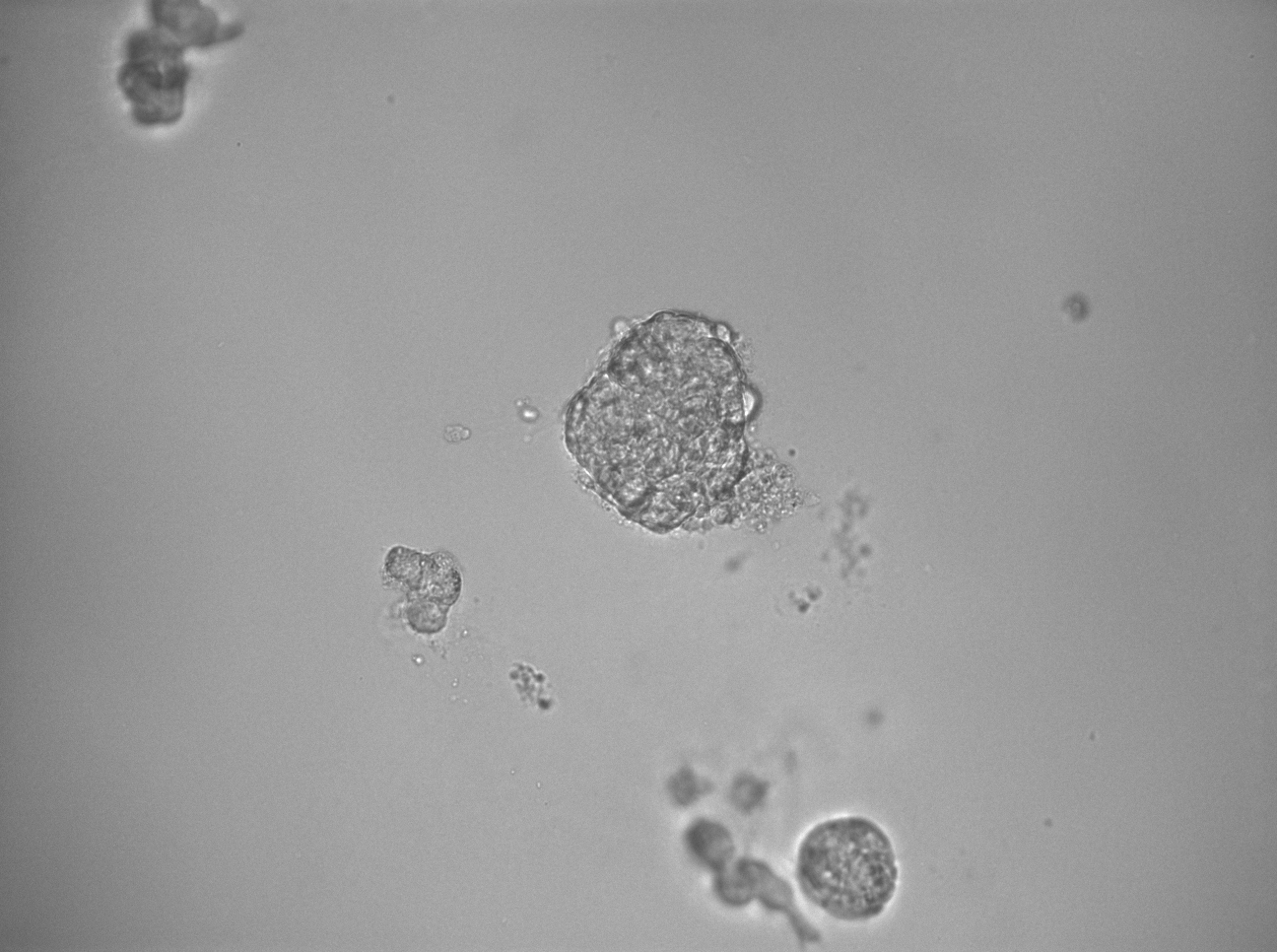

Supplement: Supplementary file 4 — Source Data Fig. 4 [file 41586_2026_10187_MOESM4_ESM.zip › HCEC1CT/HCEC1CT-GFP_D10_Dox-02000_F01g_20x_ch00.jpg]

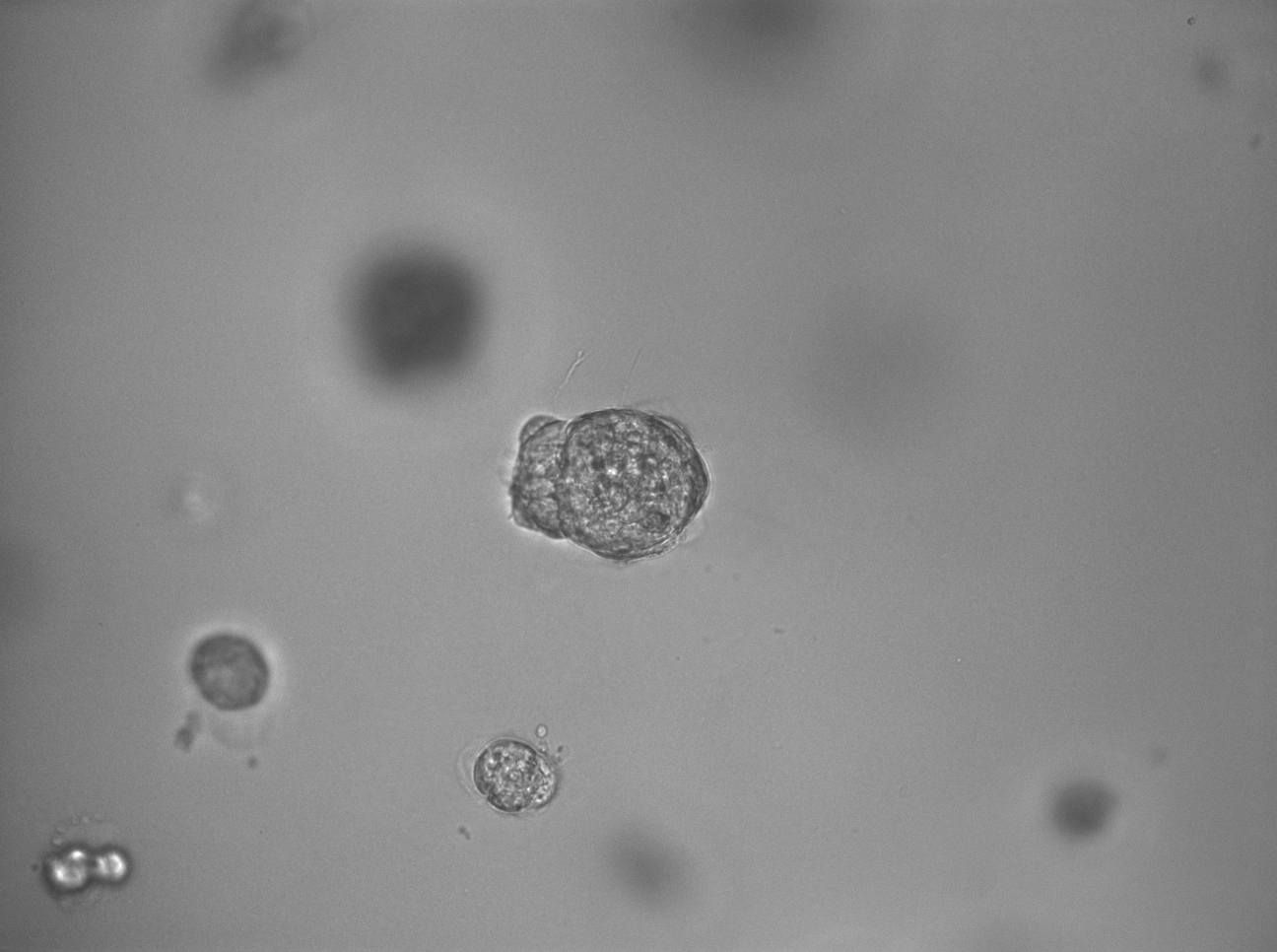

Supplement: Supplementary file 4 — Source Data Fig. 4 [file 41586_2026_10187_MOESM4_ESM.zip › HCEC1CT/HCEC1CT-GFP_D10_Dox-02000_F02a_20x_ch00.jpg]

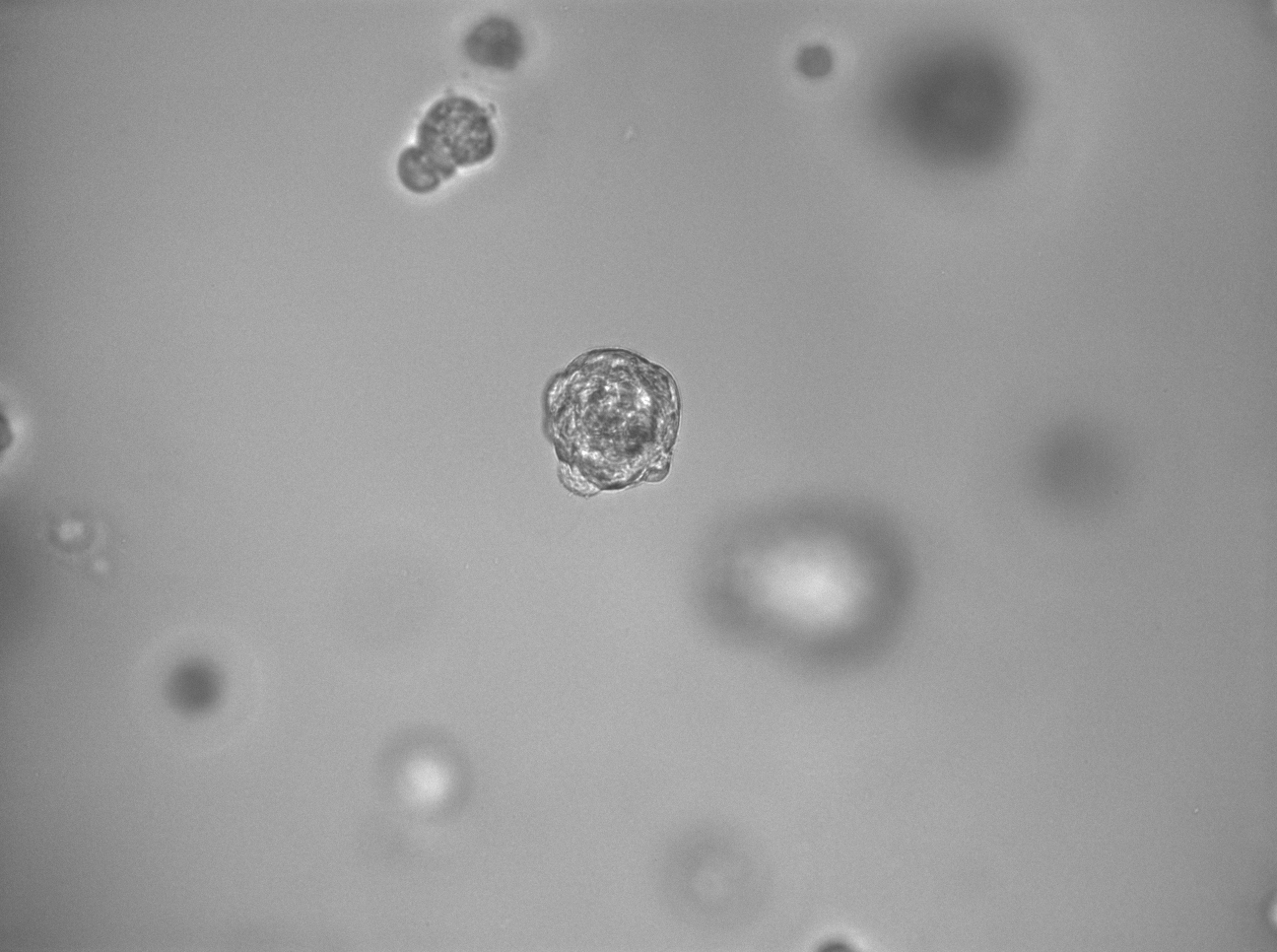

Supplement: Supplementary file 4 — Source Data Fig. 4 [file 41586_2026_10187_MOESM4_ESM.zip › HCEC1CT/HCEC1CT-GFP_D10_Dox-02000_F02b_20x_ch00.jpg]

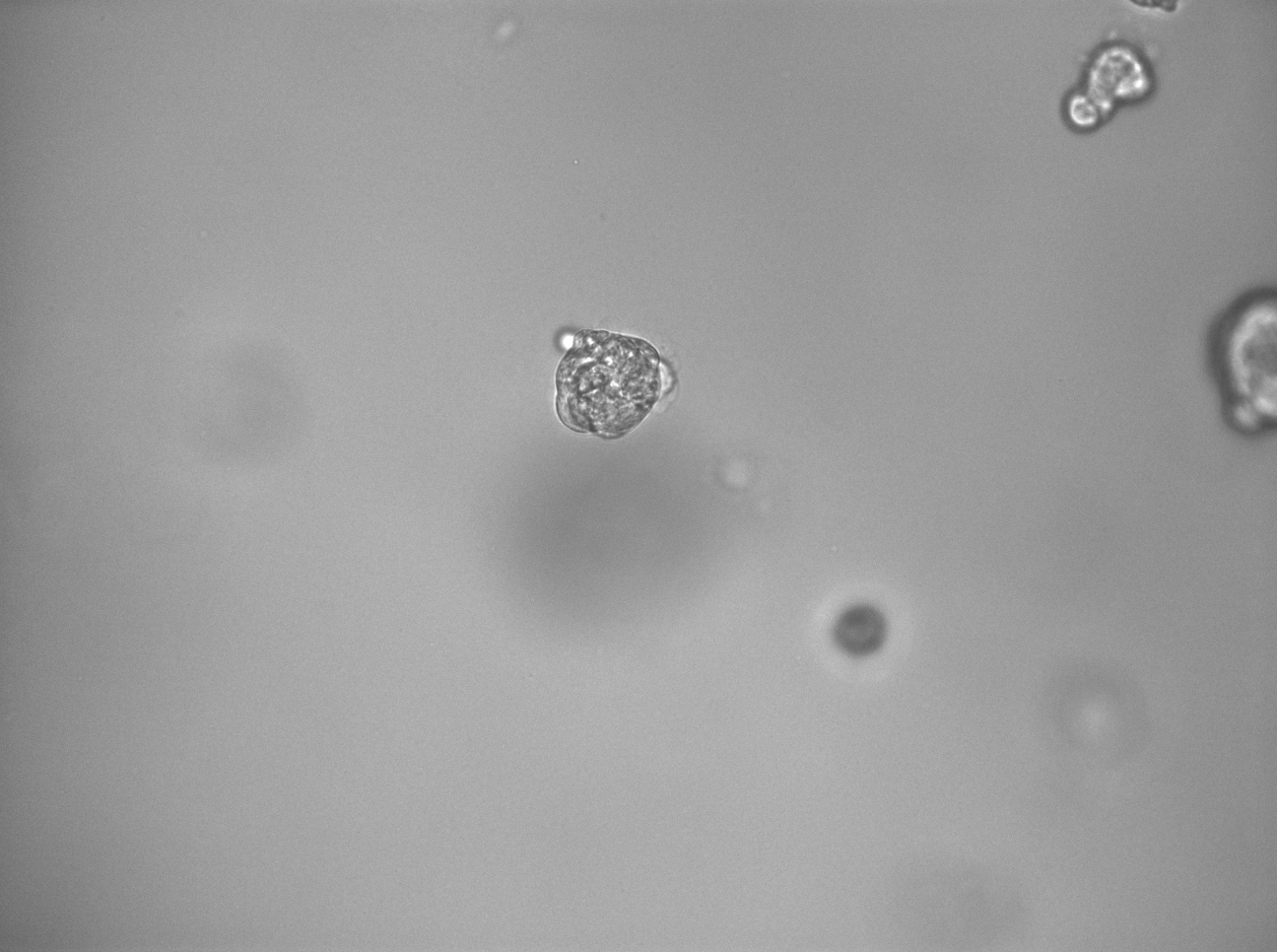

Supplement: Supplementary file 4 — Source Data Fig. 4 [file 41586_2026_10187_MOESM4_ESM.zip › HCEC1CT/HCEC1CT-GFP_D10_Dox-02000_F02c_20x_ch00.jpg]

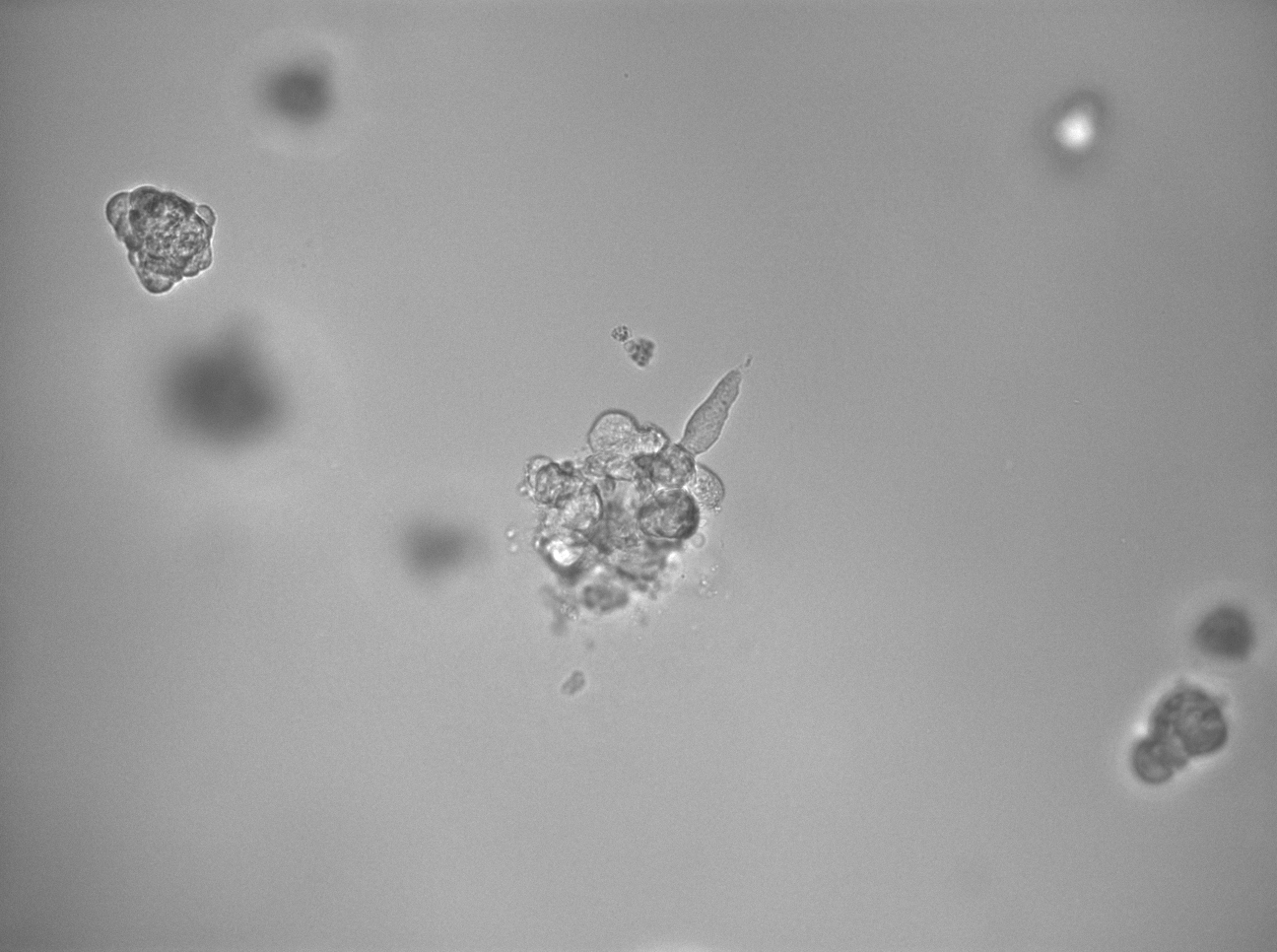

Supplement: Supplementary file 4 — Source Data Fig. 4 [file 41586_2026_10187_MOESM4_ESM.zip › HCEC1CT/HCEC1CT-GFP_D10_Dox-02000_F02d_20x_ch00.jpg]

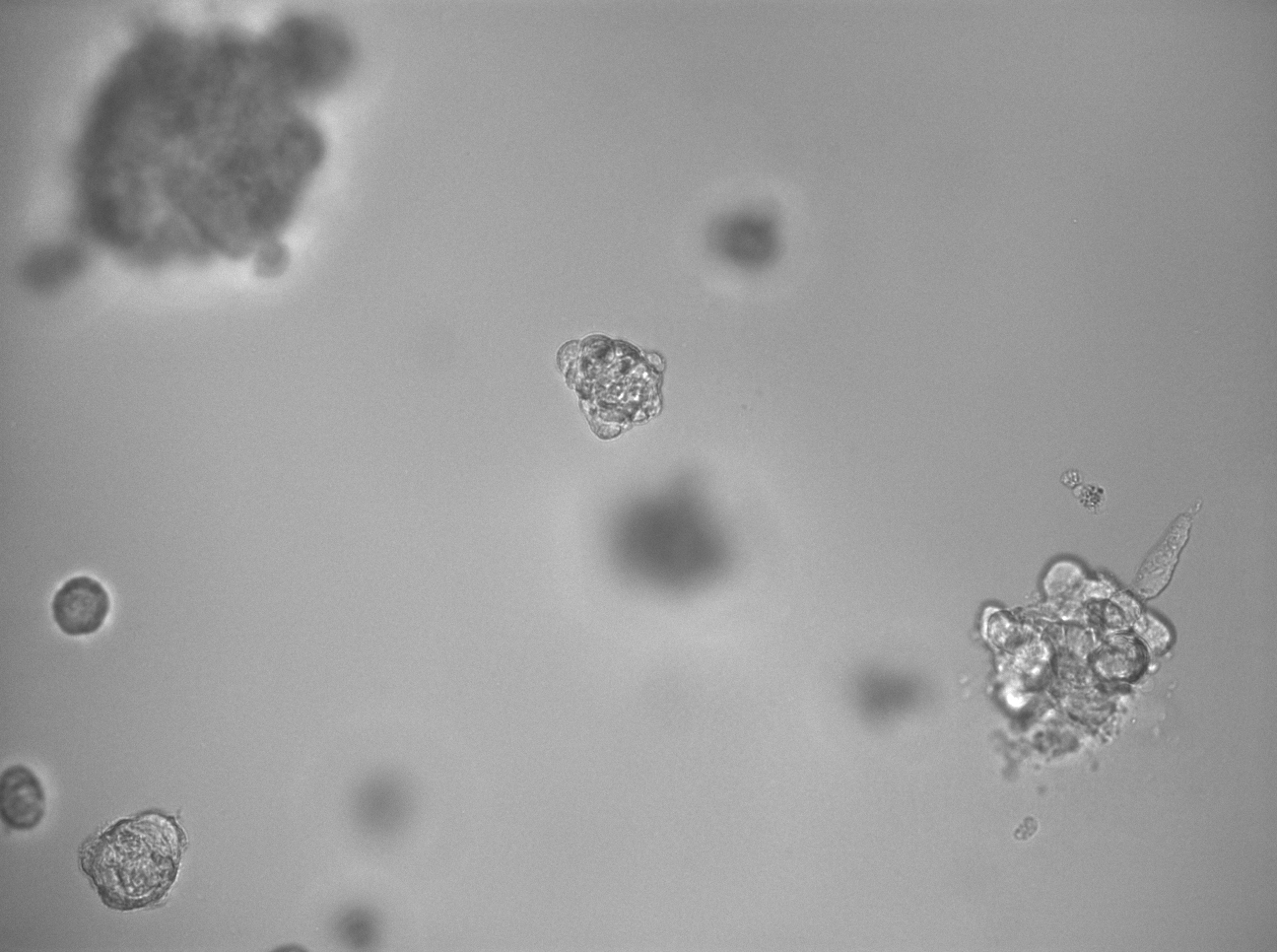

Supplement: Supplementary file 4 — Source Data Fig. 4 [file 41586_2026_10187_MOESM4_ESM.zip › HCEC1CT/HCEC1CT-GFP_D10_Dox-02000_F02e_20x_ch00.jpg]

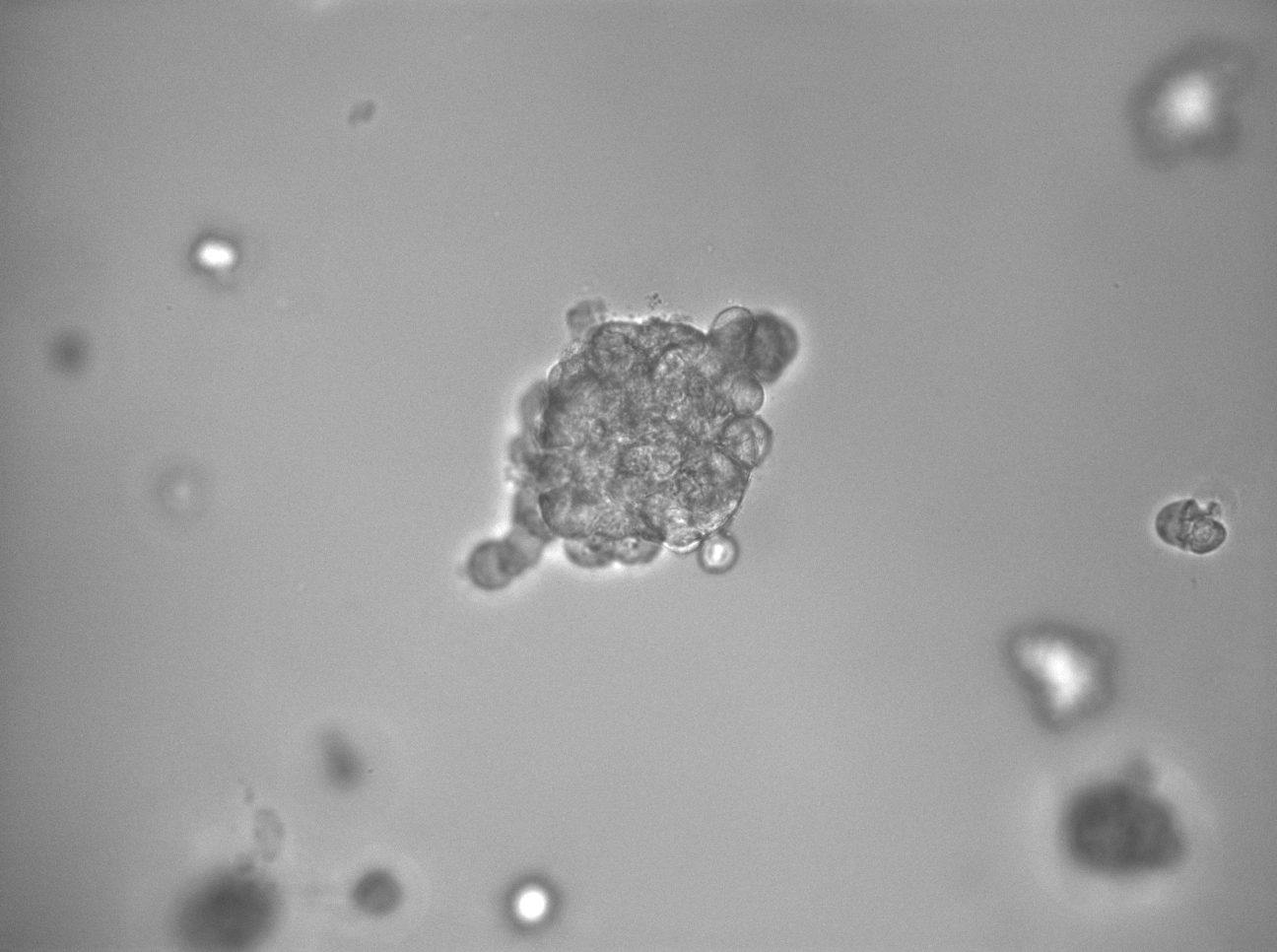

Supplement: Supplementary file 4 — Source Data Fig. 4 [file 41586_2026_10187_MOESM4_ESM.zip › HCEC1CT/HCEC1CT-GFP_D10_Dox-02000_F02f_20x_ch00.jpg]

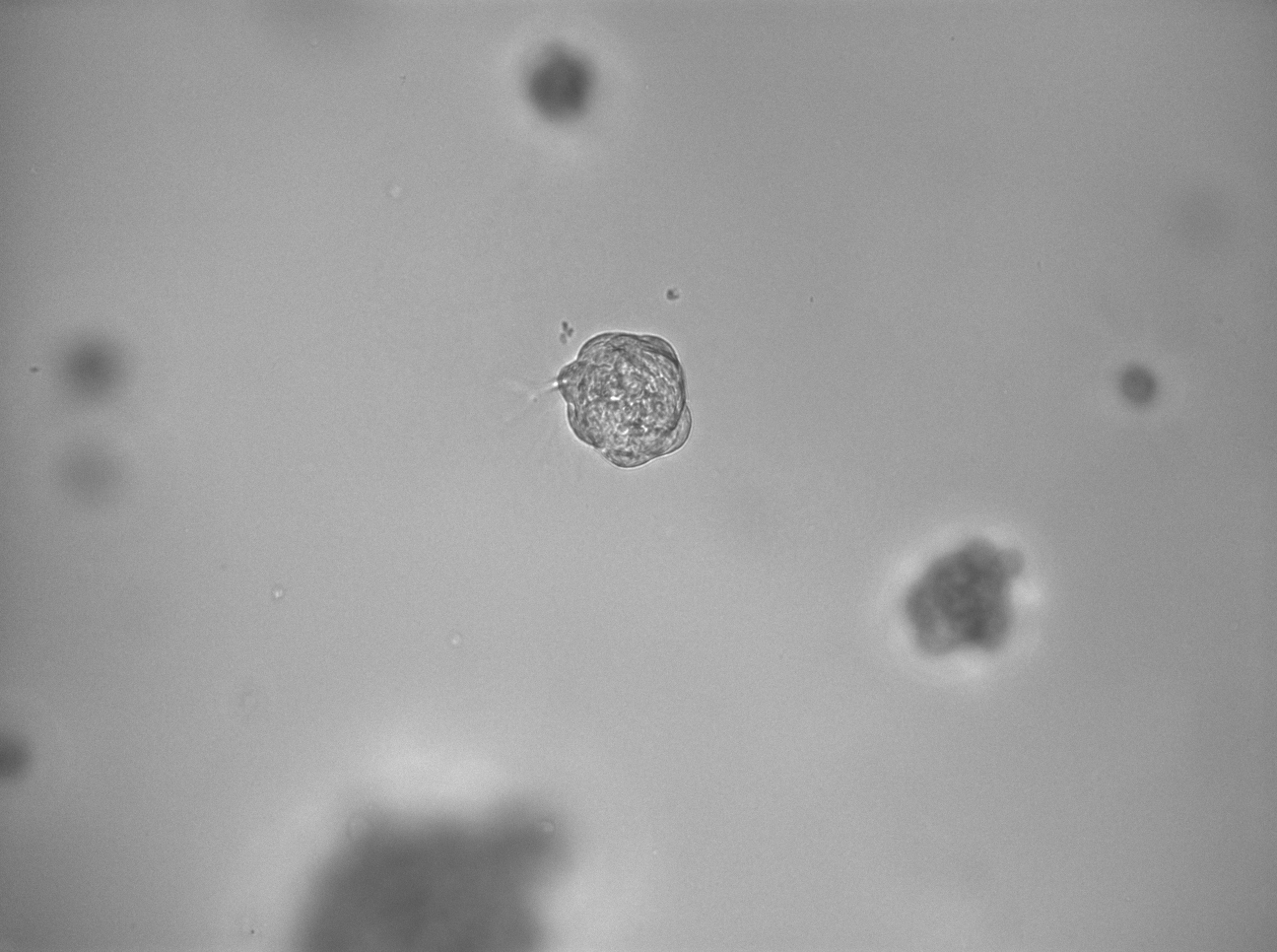

Supplement: Supplementary file 4 — Source Data Fig. 4 [file 41586_2026_10187_MOESM4_ESM.zip › HCEC1CT/HCEC1CT-GFP_D10_Dox-02000_F02g_20x_ch00.jpg]

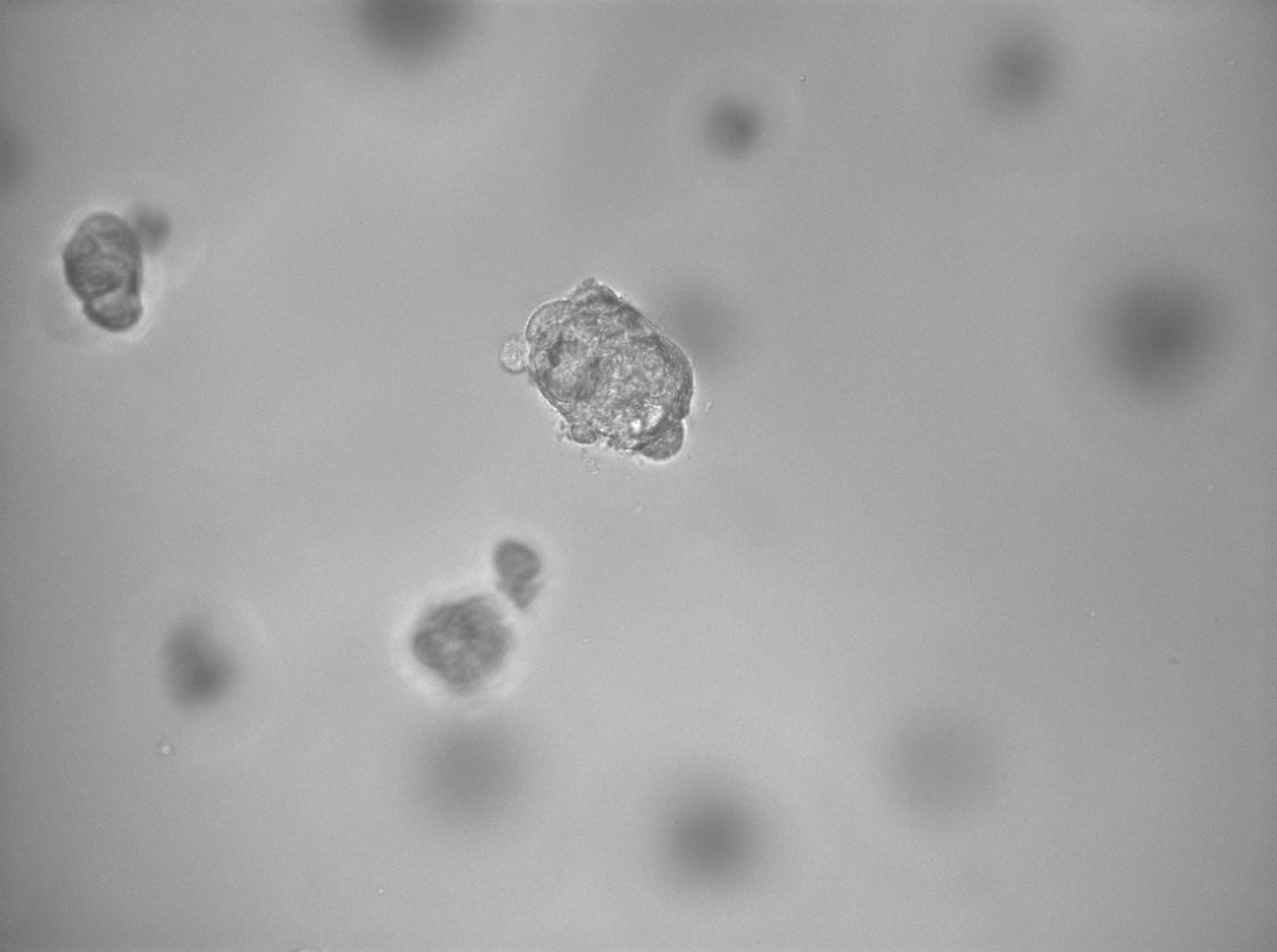

Supplement: Supplementary file 4 — Source Data Fig. 4 [file 41586_2026_10187_MOESM4_ESM.zip › HCEC1CT/HCEC1CT-GFP_D10_Dox-02000_F03a_20x_ch00.jpg]

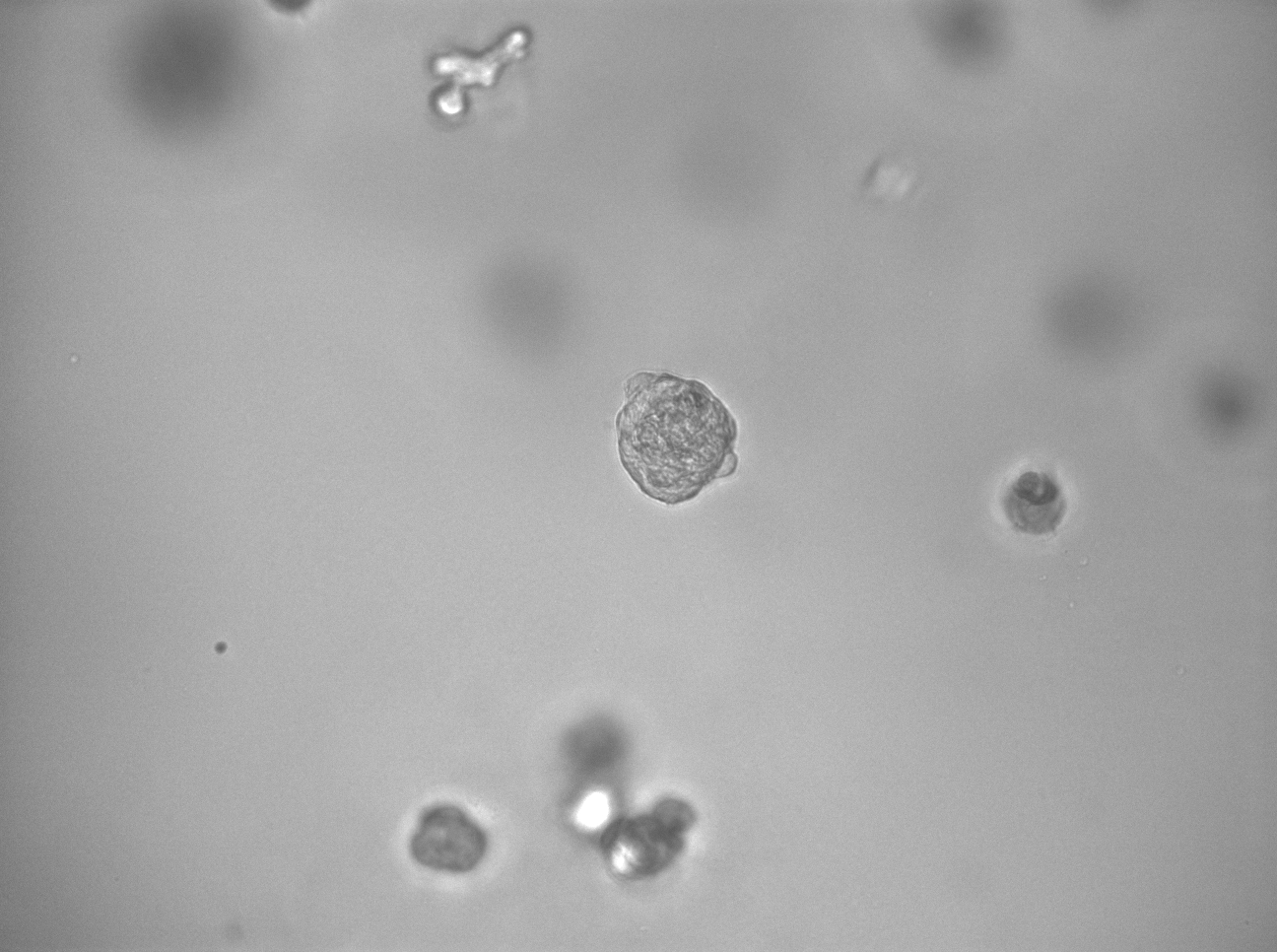

Supplement: Supplementary file 4 — Source Data Fig. 4 [file 41586_2026_10187_MOESM4_ESM.zip › HCEC1CT/HCEC1CT-GFP_D10_Dox-02000_F03b_20x_ch00.jpg]

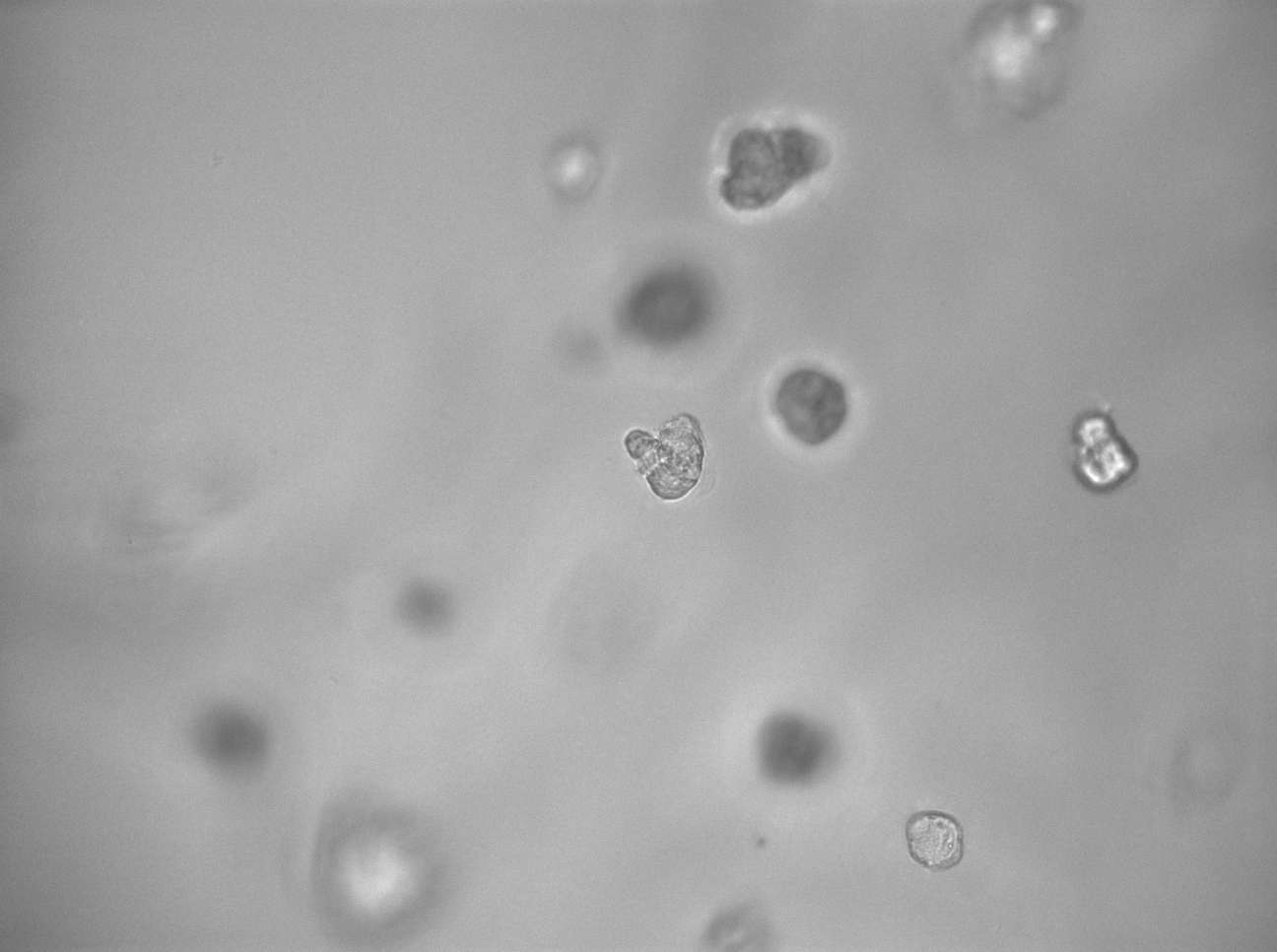

Supplement: Supplementary file 4 — Source Data Fig. 4 [file 41586_2026_10187_MOESM4_ESM.zip › HCEC1CT/HCEC1CT-GFP_D10_Dox-02000_F03c_20x_ch00.jpg]

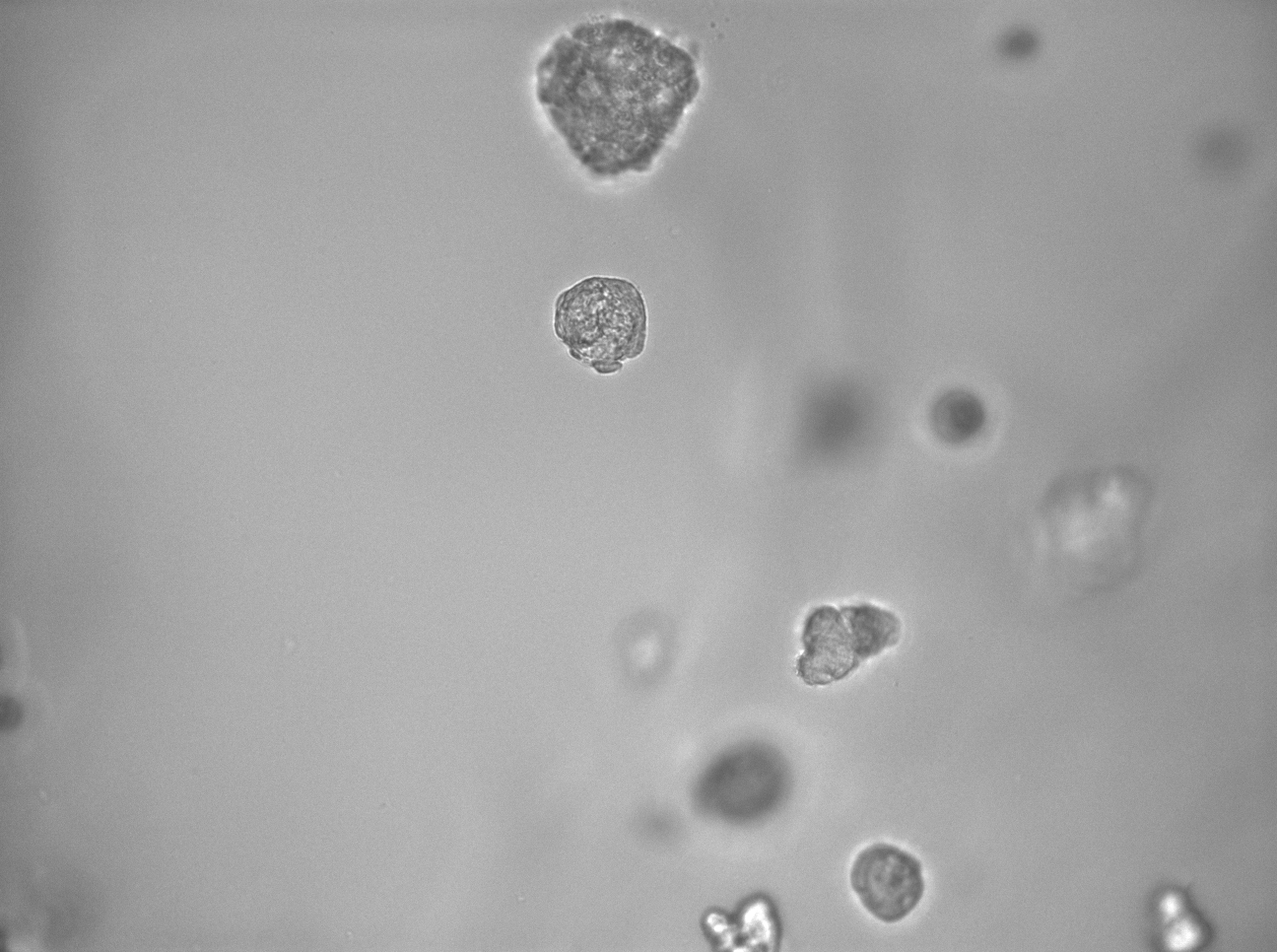

Supplement: Supplementary file 4 — Source Data Fig. 4 [file 41586_2026_10187_MOESM4_ESM.zip › HCEC1CT/HCEC1CT-GFP_D10_Dox-02000_F03d_20x_ch00.jpg]

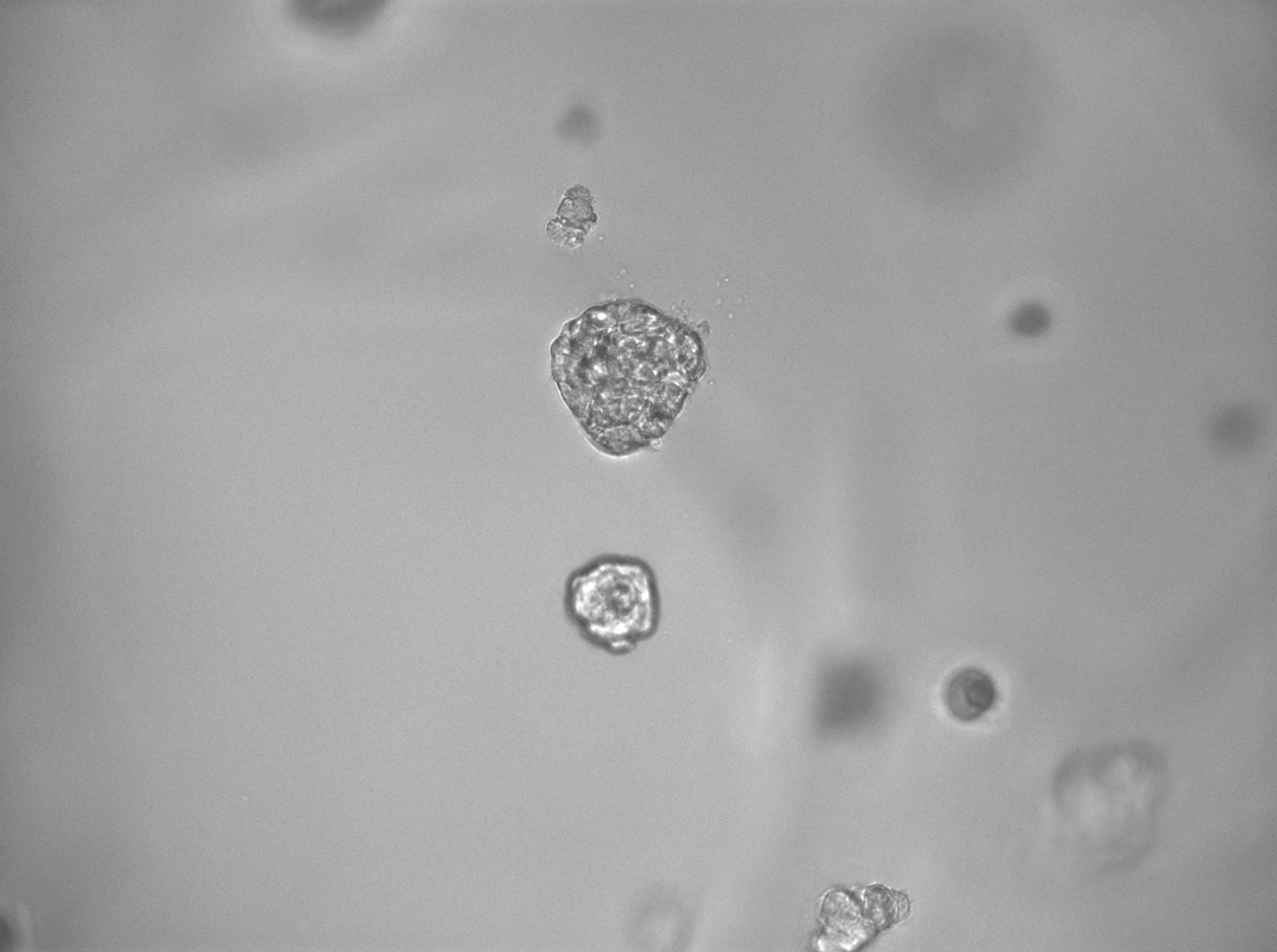

Supplement: Supplementary file 4 — Source Data Fig. 4 [file 41586_2026_10187_MOESM4_ESM.zip › HCEC1CT/HCEC1CT-GFP_D10_Dox-02000_F03e_20x_ch00.jpg]

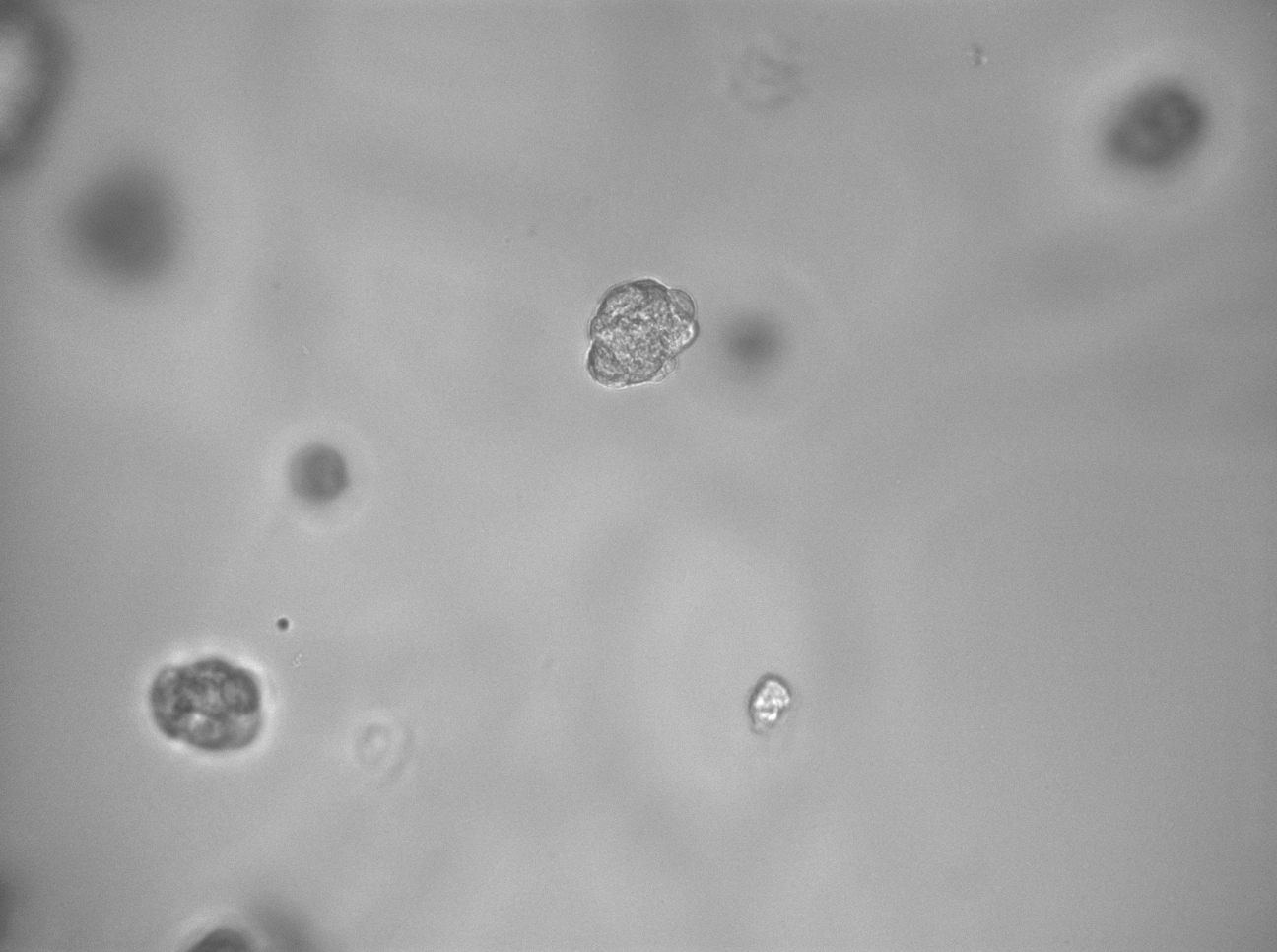

Supplement: Supplementary file 4 — Source Data Fig. 4 [file 41586_2026_10187_MOESM4_ESM.zip › HCEC1CT/HCEC1CT-GFP_D10_Dox-02000_F03f_20x_ch00.jpg]

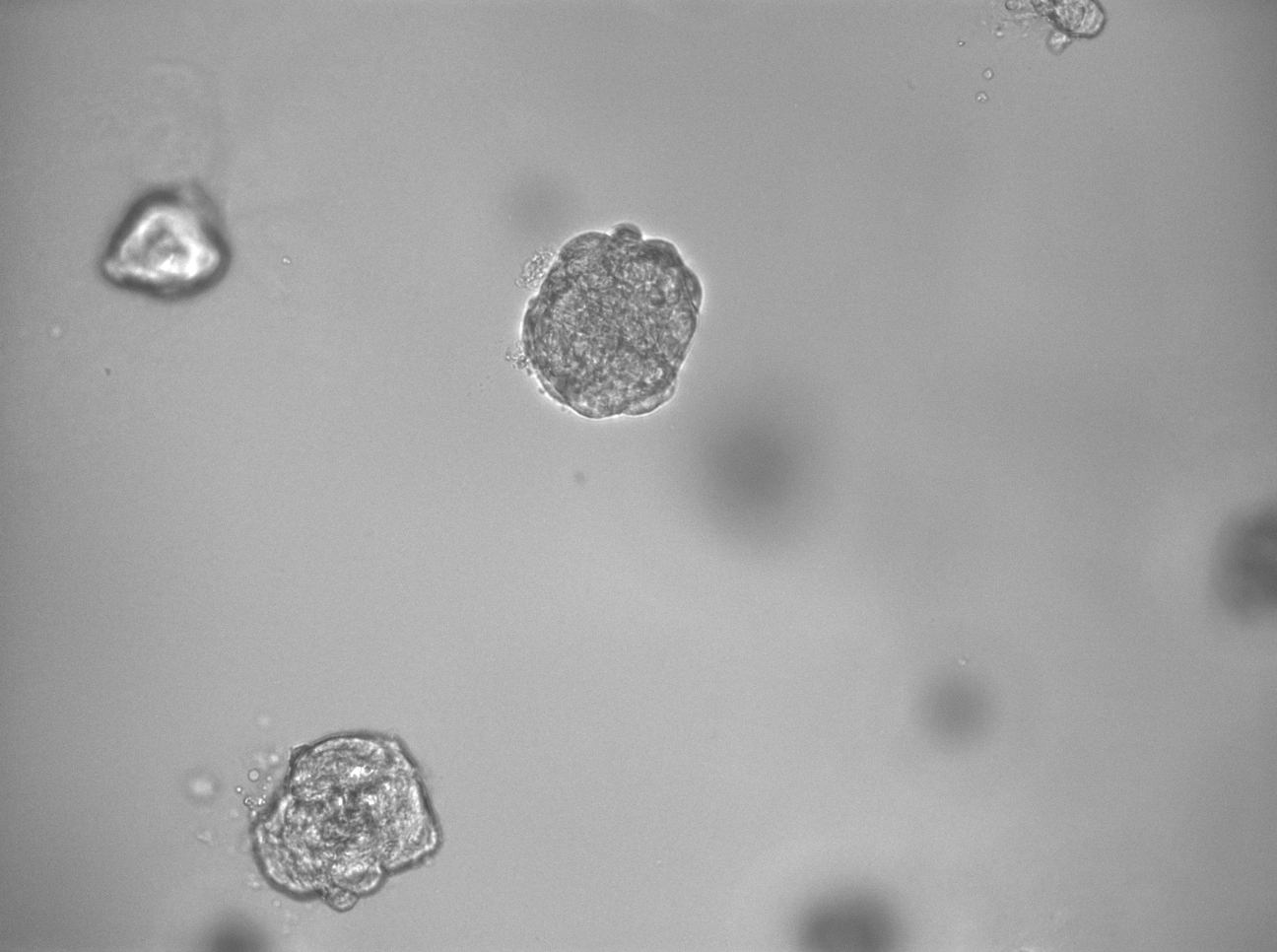

Supplement: Supplementary file 4 — Source Data Fig. 4 [file 41586_2026_10187_MOESM4_ESM.zip › HCEC1CT/HCEC1CT-GFP_D10_Dox-02000_F03g_20x_ch00.jpg]

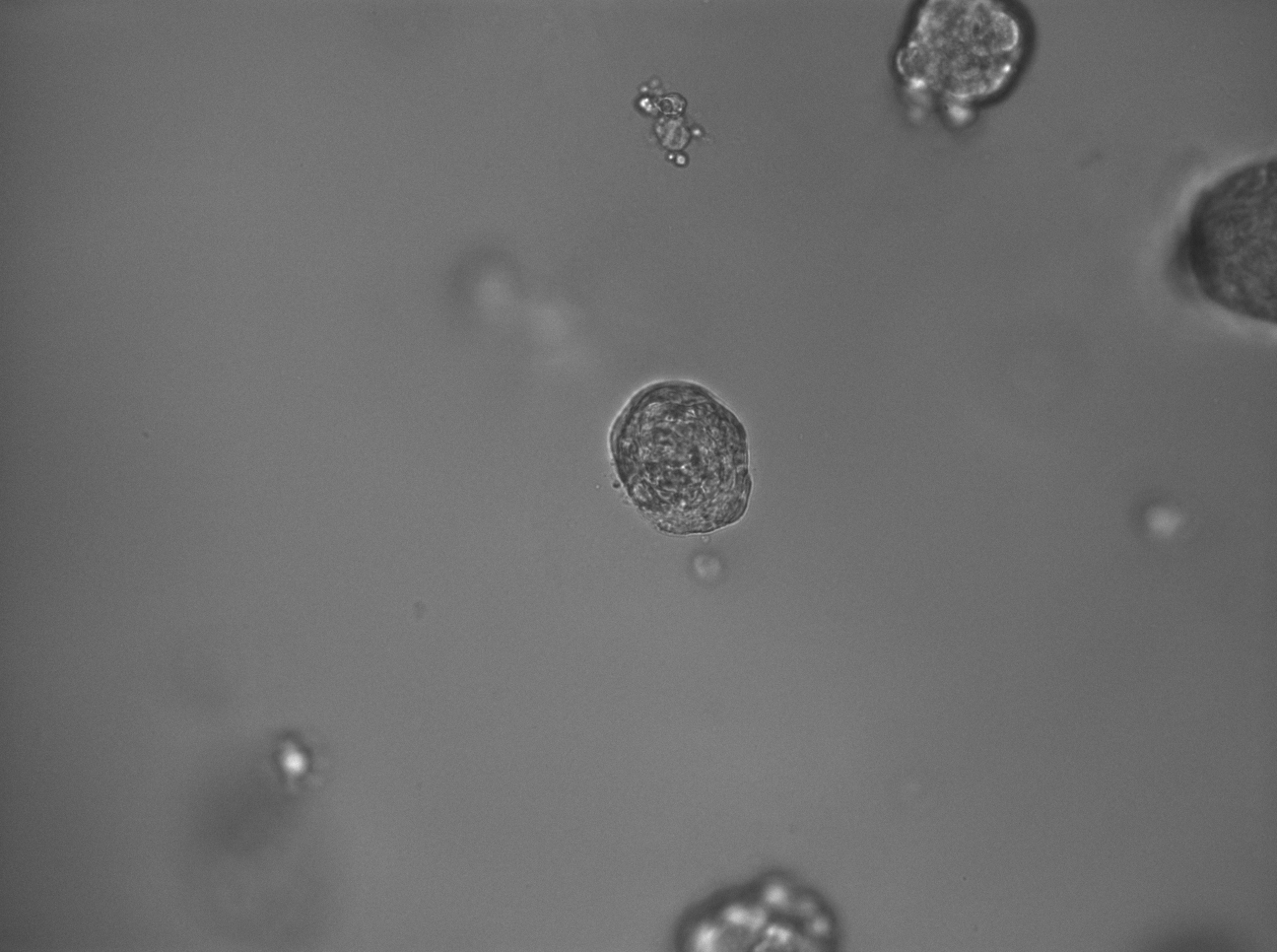

Supplement: Supplementary file 4 — Source Data Fig. 4 [file 41586_2026_10187_MOESM4_ESM.zip › HCEC1CT/HCEC1CT-KRAS_D10_Dox-00000_A01c_20x_ch00.jpg]

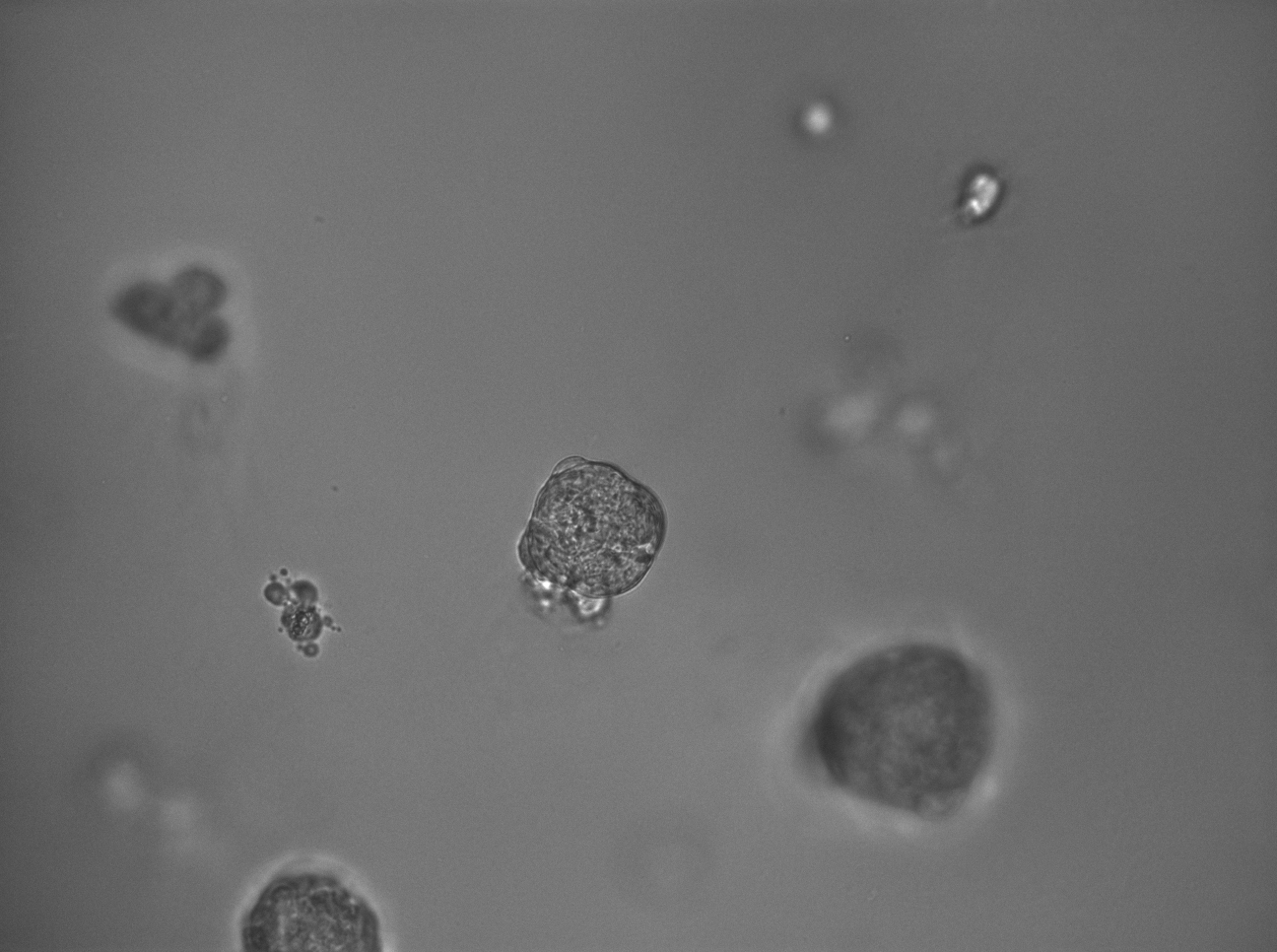

Supplement: Supplementary file 4 — Source Data Fig. 4 [file 41586_2026_10187_MOESM4_ESM.zip › HCEC1CT/HCEC1CT-KRAS_D10_Dox-00000_A01d_20x_ch00.jpg]

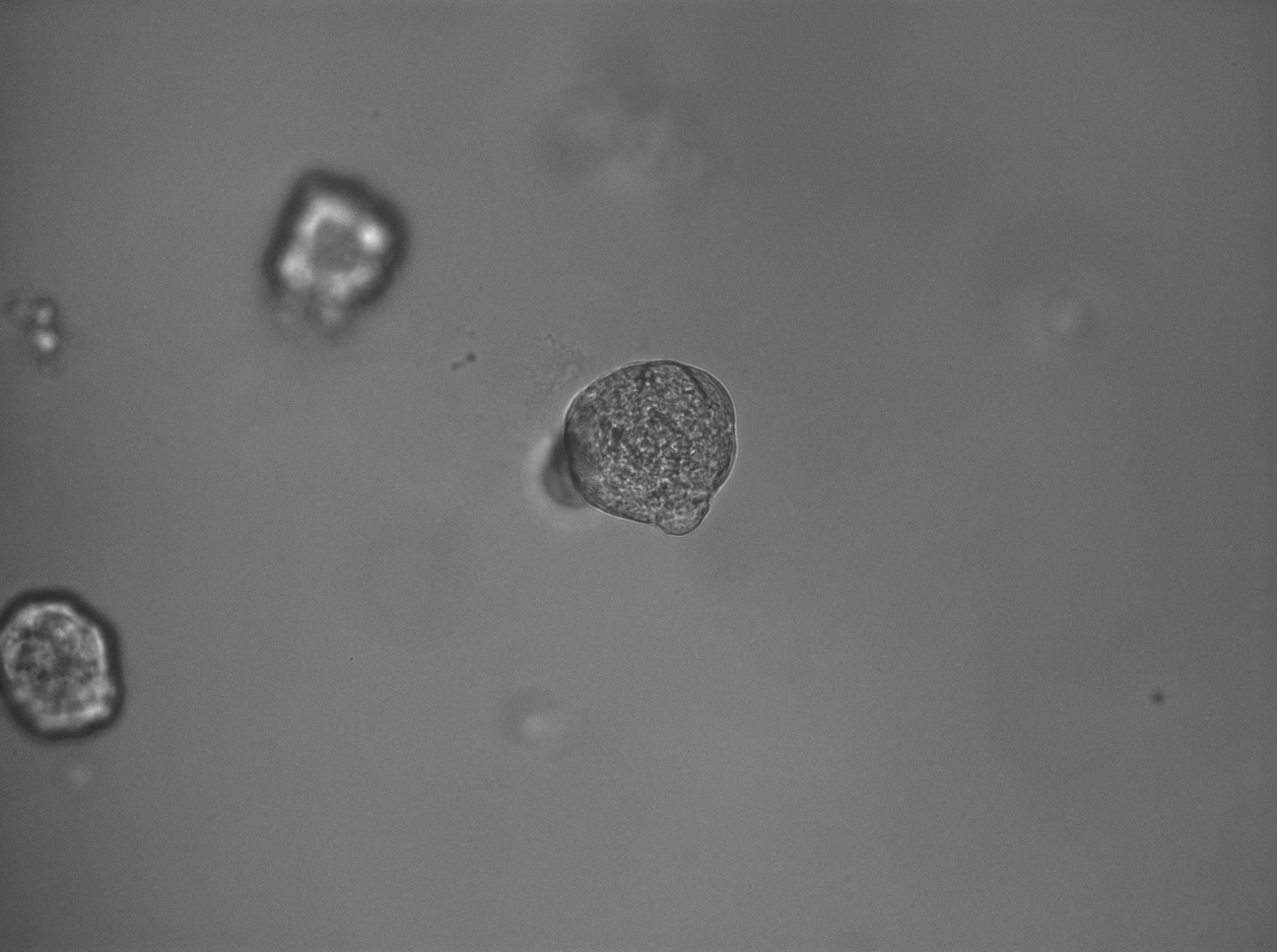

Supplement: Supplementary file 4 — Source Data Fig. 4 [file 41586_2026_10187_MOESM4_ESM.zip › HCEC1CT/HCEC1CT-KRAS_D10_Dox-00000_A01e_20x_ch00.jpg]

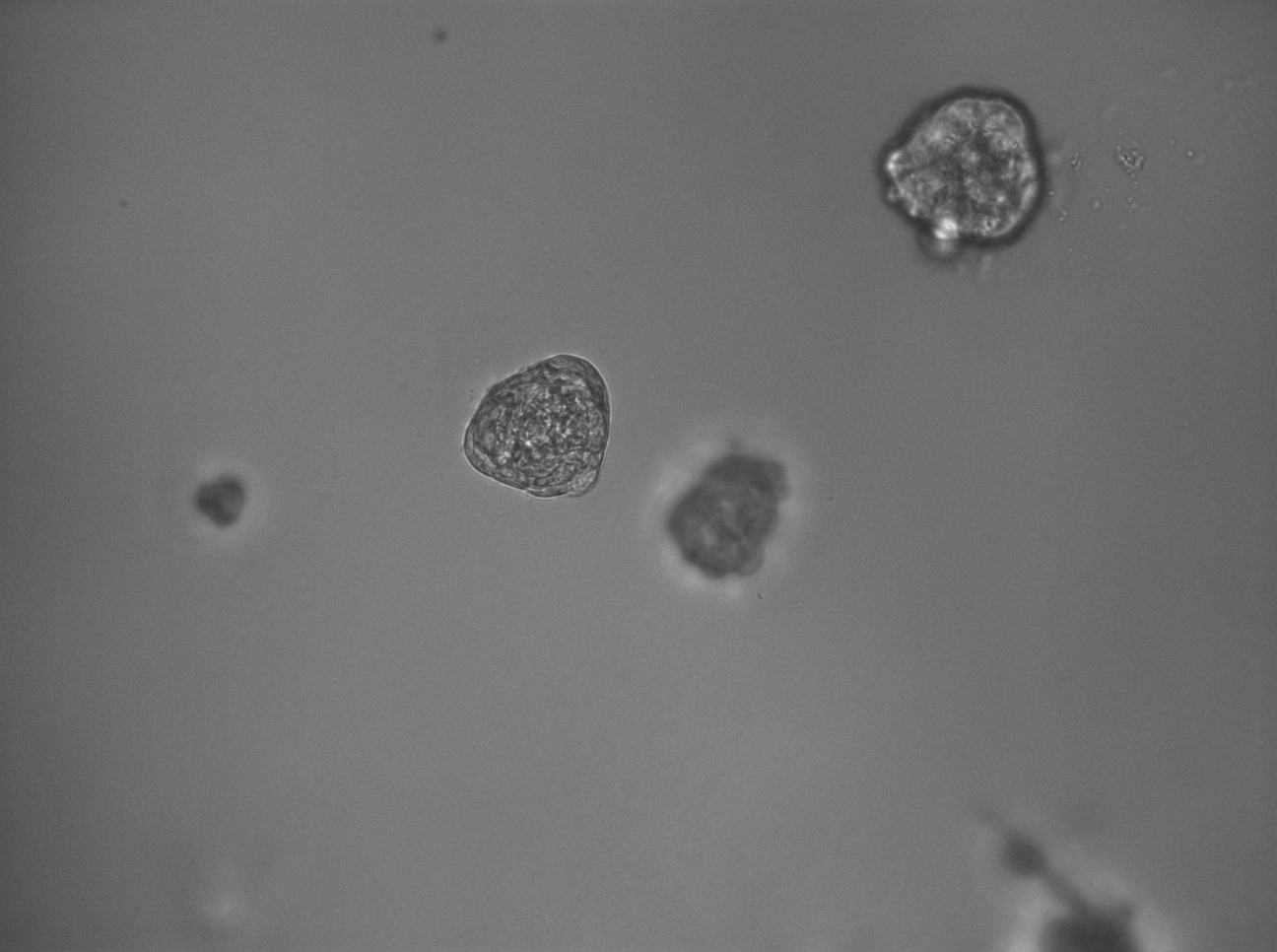

Supplement: Supplementary file 4 — Source Data Fig. 4 [file 41586_2026_10187_MOESM4_ESM.zip › HCEC1CT/HCEC1CT-KRAS_D10_Dox-00000_A01f_20x_ch00.jpg]

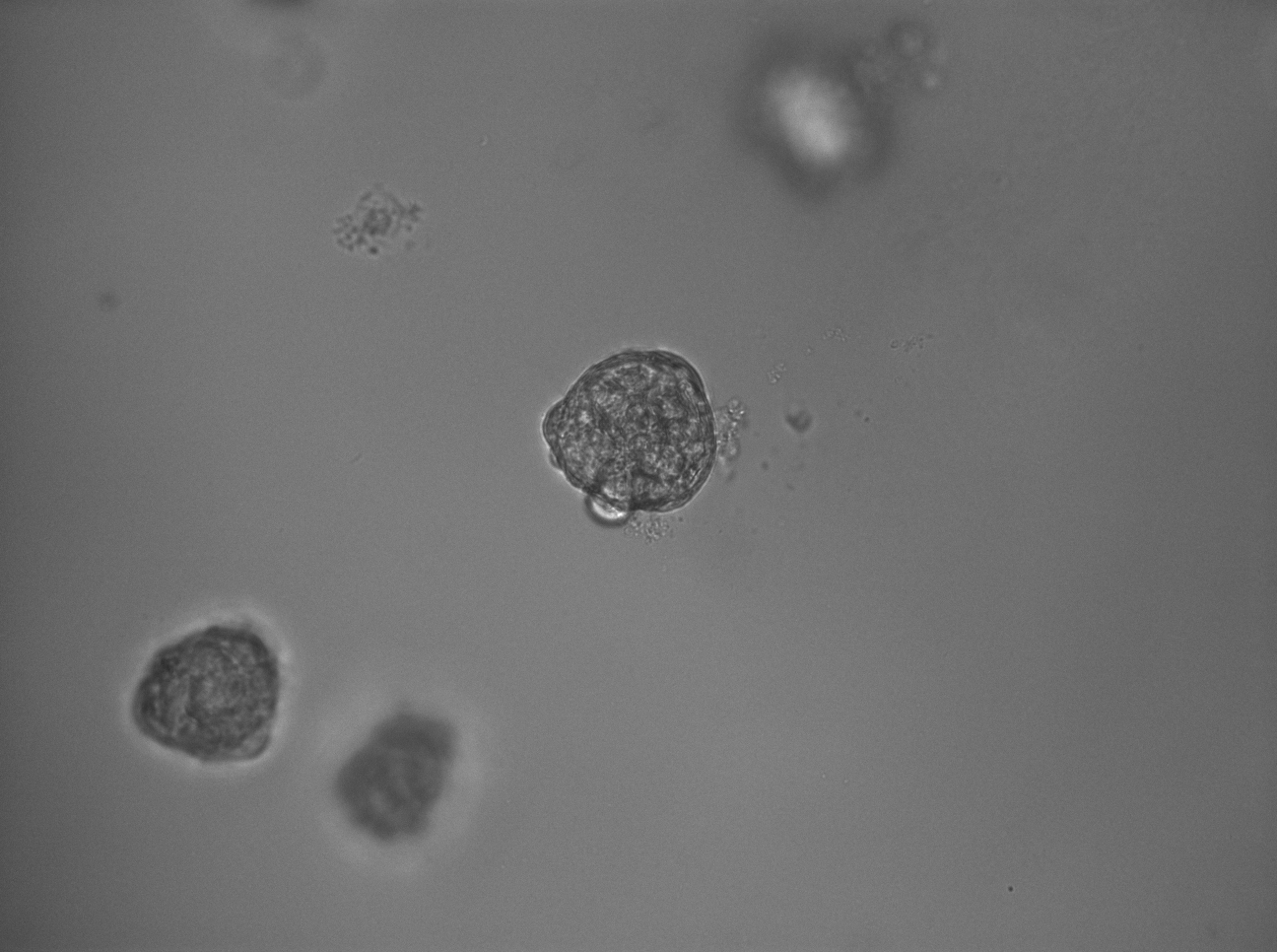

Supplement: Supplementary file 4 — Source Data Fig. 4 [file 41586_2026_10187_MOESM4_ESM.zip › HCEC1CT/HCEC1CT-KRAS_D10_Dox-00000_A01g_20x_ch00.jpg]

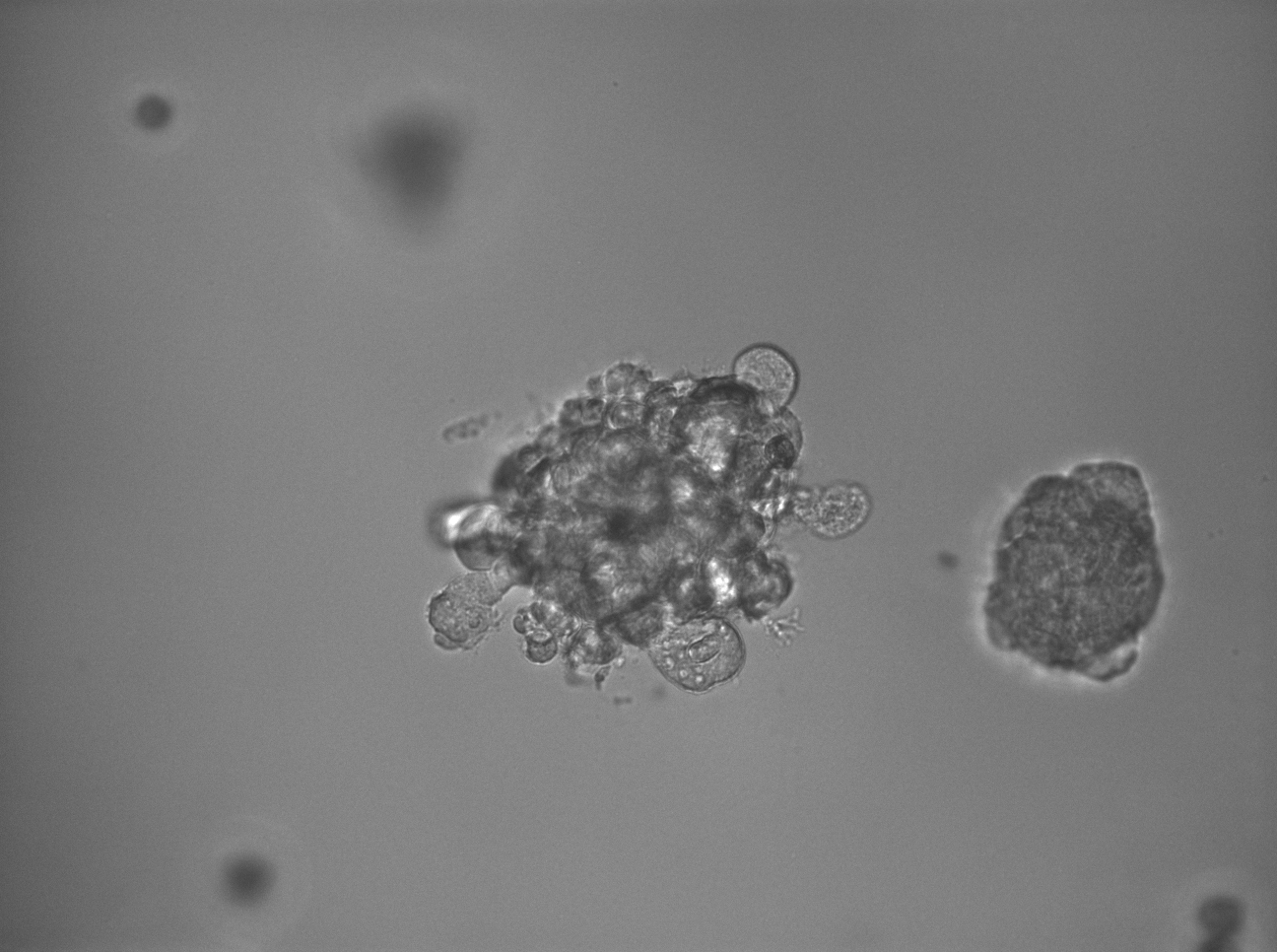

Supplement: Supplementary file 4 — Source Data Fig. 4 [file 41586_2026_10187_MOESM4_ESM.zip › HCEC1CT/HCEC1CT-KRAS_D10_Dox-00000_A02a_20x_ch00.jpg]

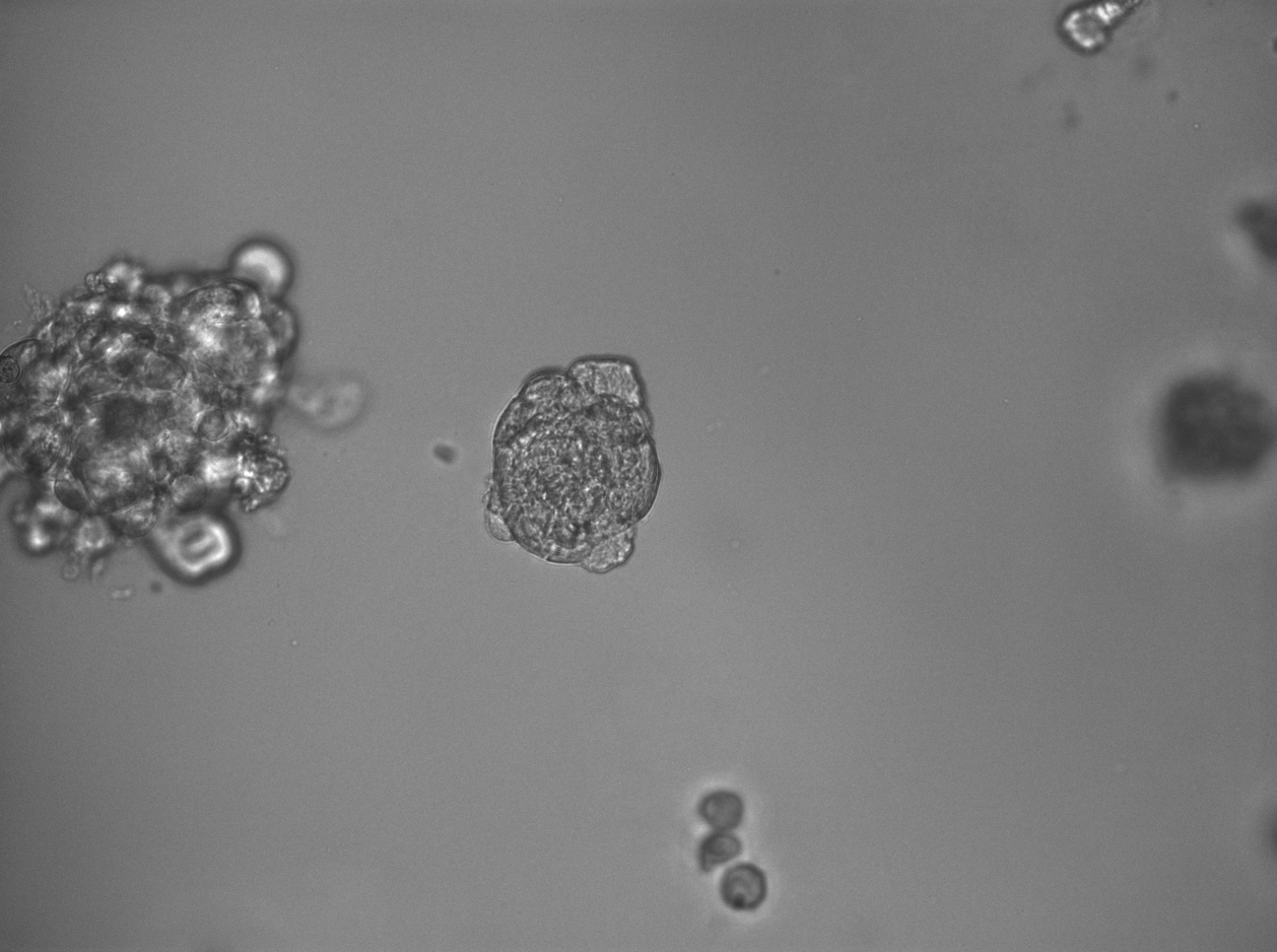

Supplement: Supplementary file 4 — Source Data Fig. 4 [file 41586_2026_10187_MOESM4_ESM.zip › HCEC1CT/HCEC1CT-KRAS_D10_Dox-00000_A02b_20x_ch00.jpg]

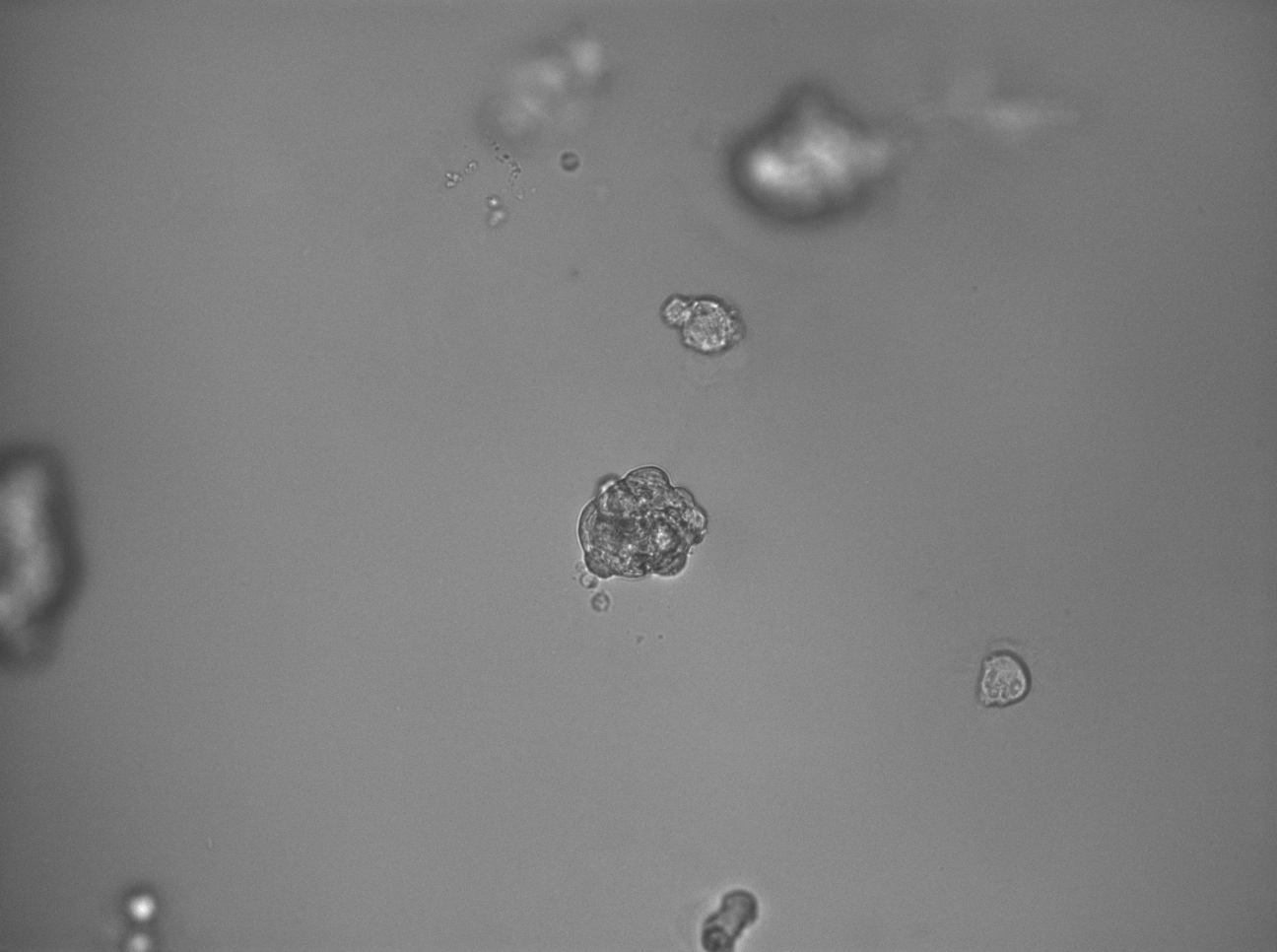

Supplement: Supplementary file 4 — Source Data Fig. 4 [file 41586_2026_10187_MOESM4_ESM.zip › HCEC1CT/HCEC1CT-KRAS_D10_Dox-00000_A02c_20x_ch00.jpg]

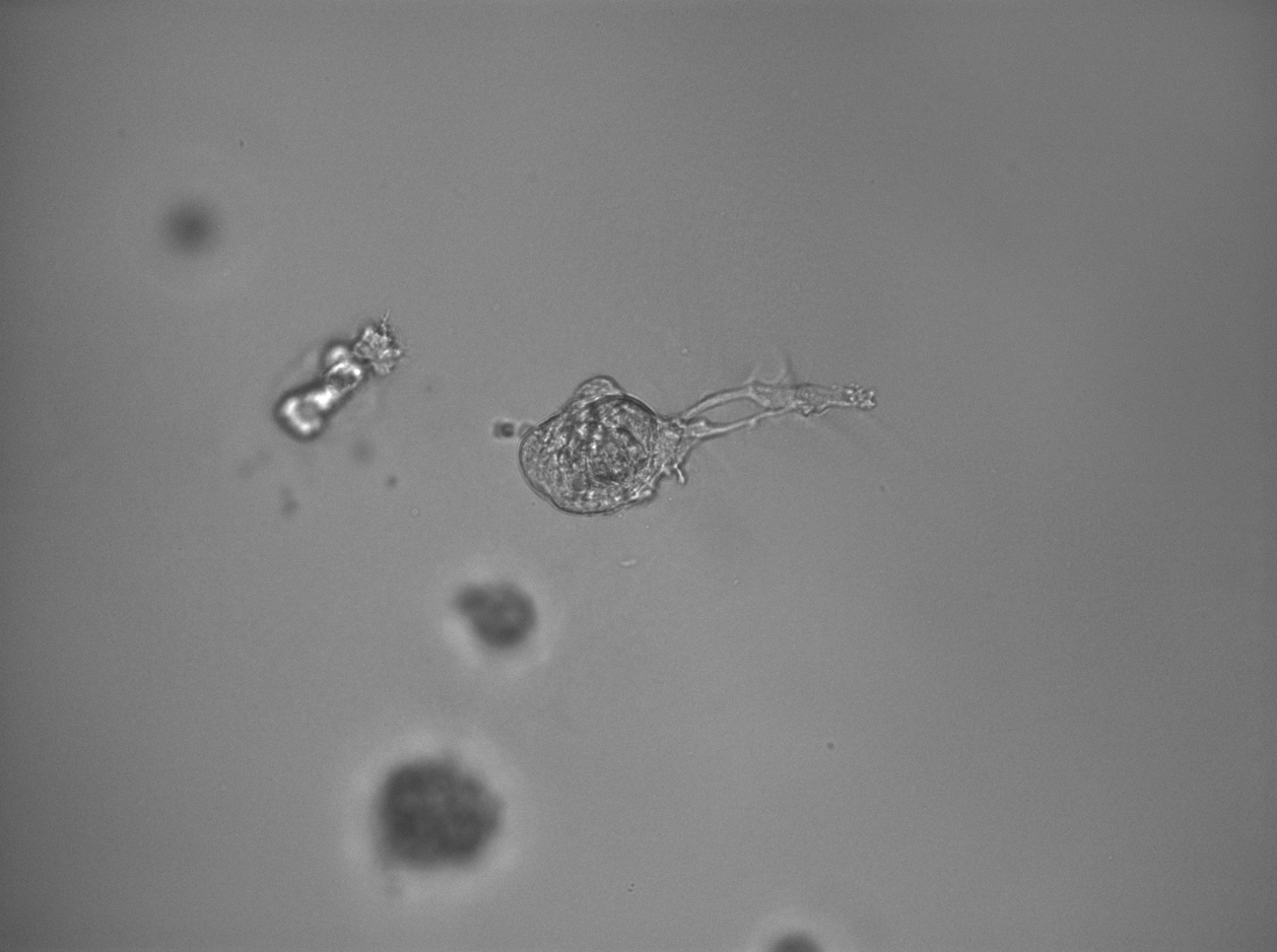

Supplement: Supplementary file 4 — Source Data Fig. 4 [file 41586_2026_10187_MOESM4_ESM.zip › HCEC1CT/HCEC1CT-KRAS_D10_Dox-00000_A02d_20x_ch00.jpg]

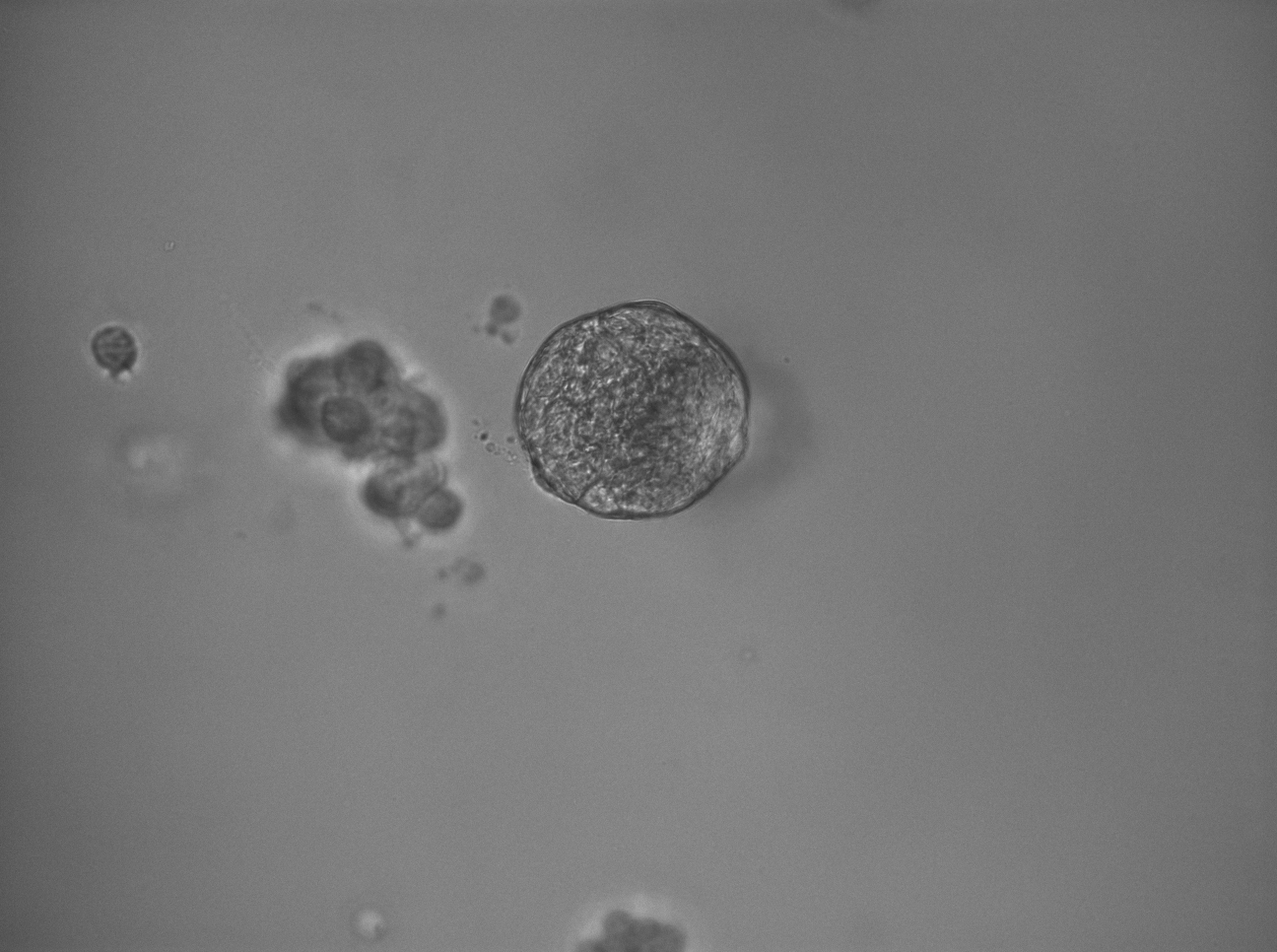

Supplement: Supplementary file 4 — Source Data Fig. 4 [file 41586_2026_10187_MOESM4_ESM.zip › HCEC1CT/HCEC1CT-KRAS_D10_Dox-00000_A02e_20x_ch00.jpg]

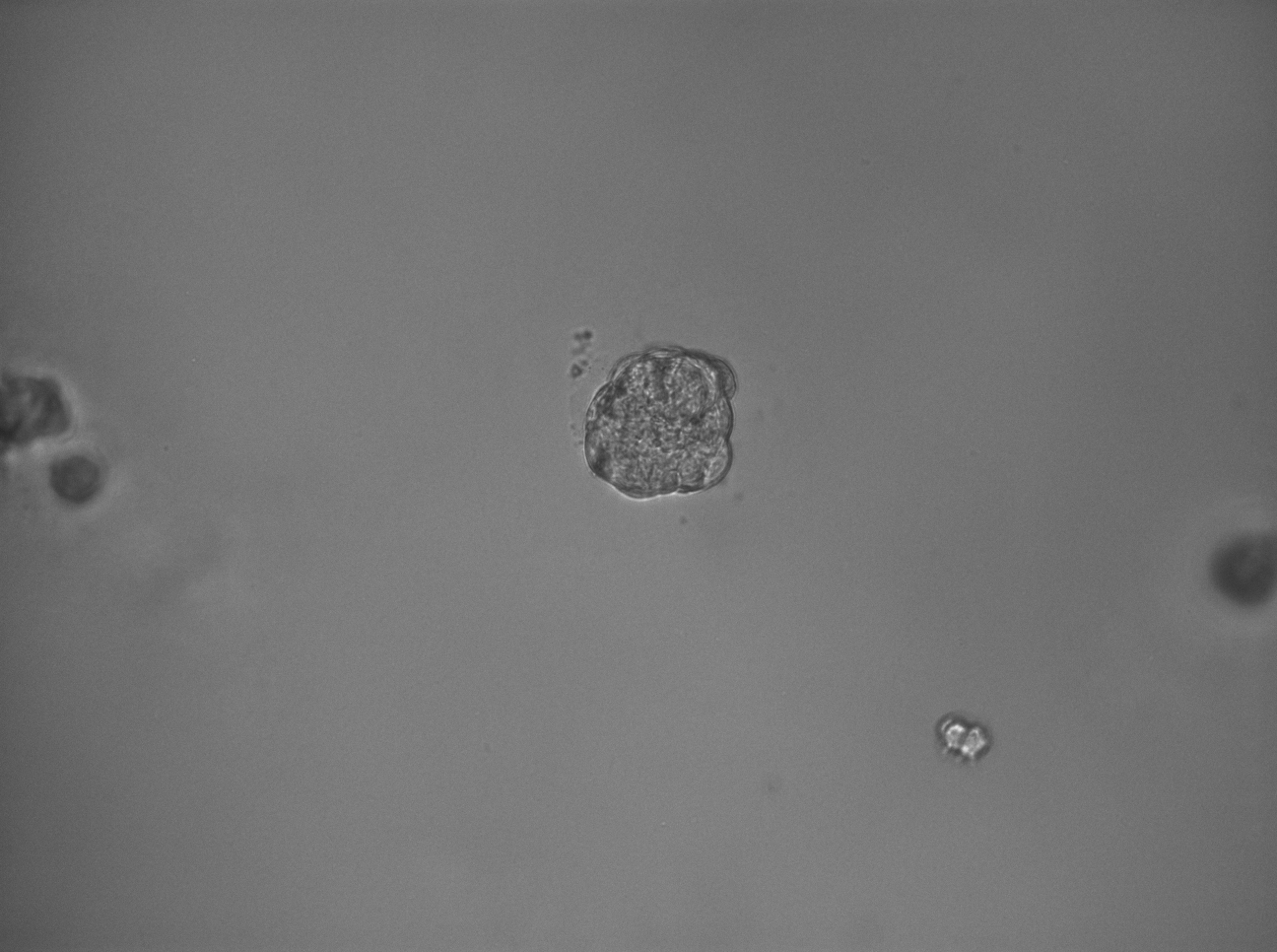

Supplement: Supplementary file 4 — Source Data Fig. 4 [file 41586_2026_10187_MOESM4_ESM.zip › HCEC1CT/HCEC1CT-KRAS_D10_Dox-00000_A02f_20x_ch00.jpg]

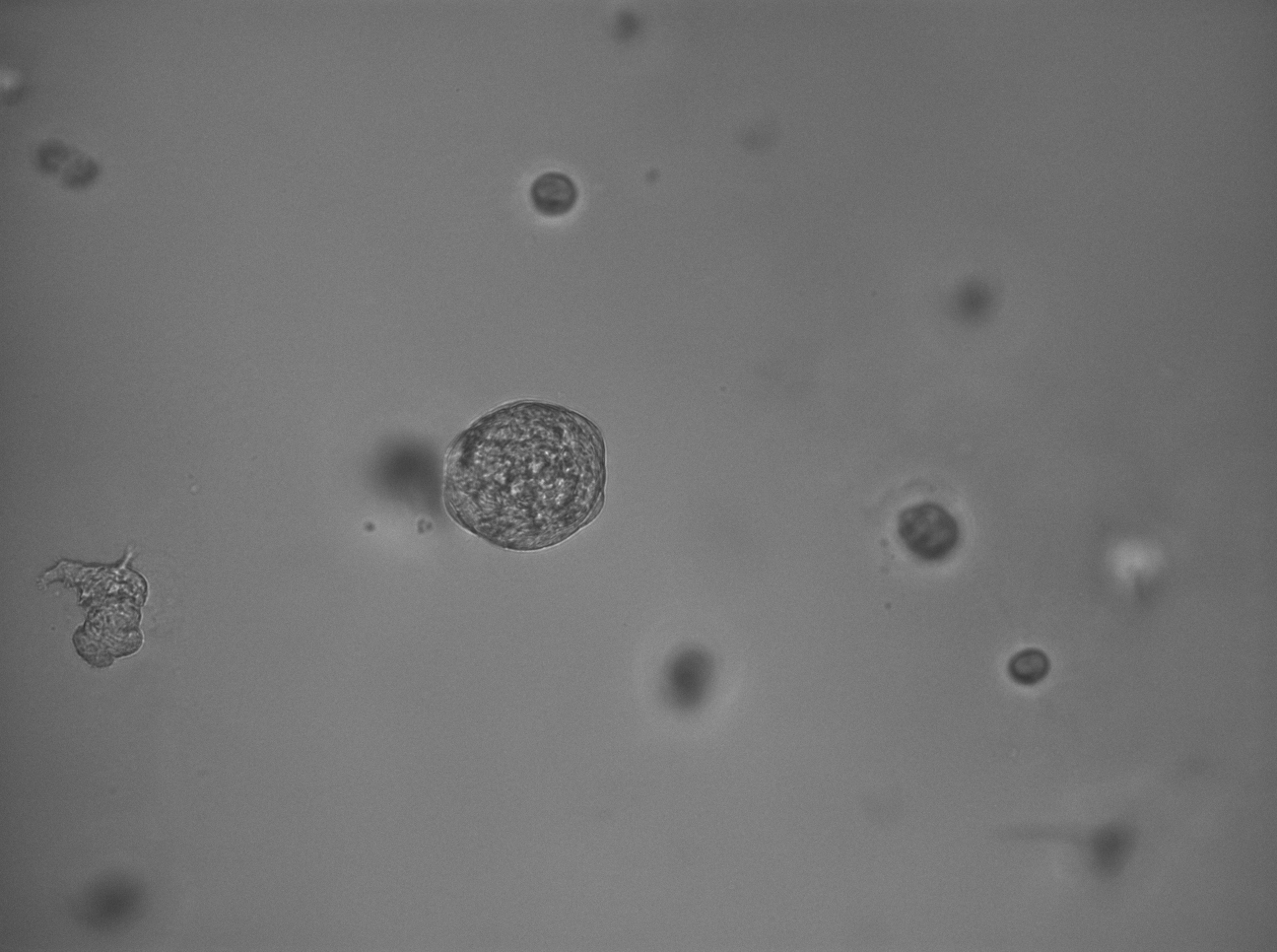

Supplement: Supplementary file 4 — Source Data Fig. 4 [file 41586_2026_10187_MOESM4_ESM.zip › HCEC1CT/HCEC1CT-KRAS_D10_Dox-00000_A02g_20x_ch00.jpg]

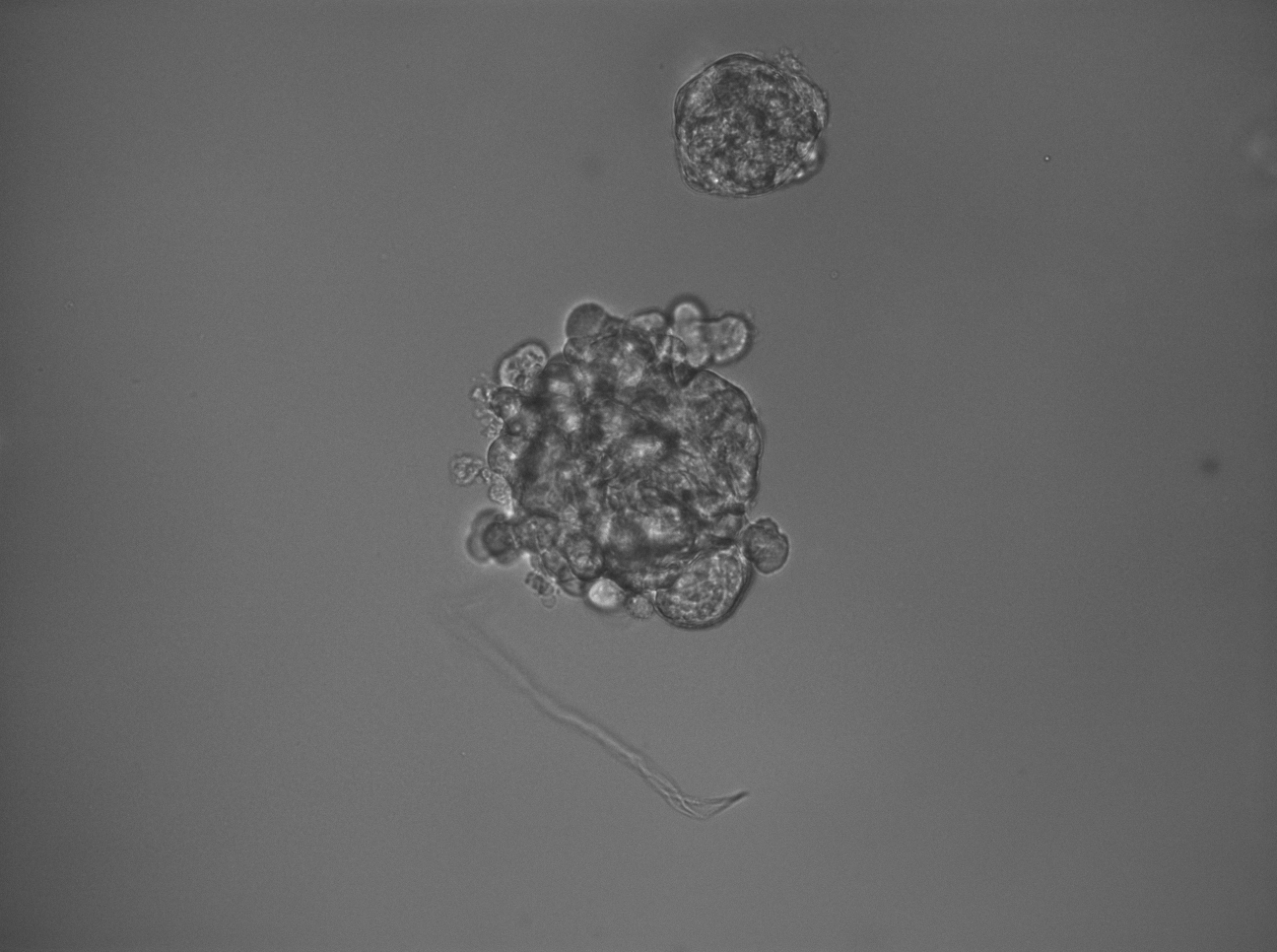

Supplement: Supplementary file 4 — Source Data Fig. 4 [file 41586_2026_10187_MOESM4_ESM.zip › HCEC1CT/HCEC1CT-KRAS_D10_Dox-00000_A03a_20x_ch00.jpg]

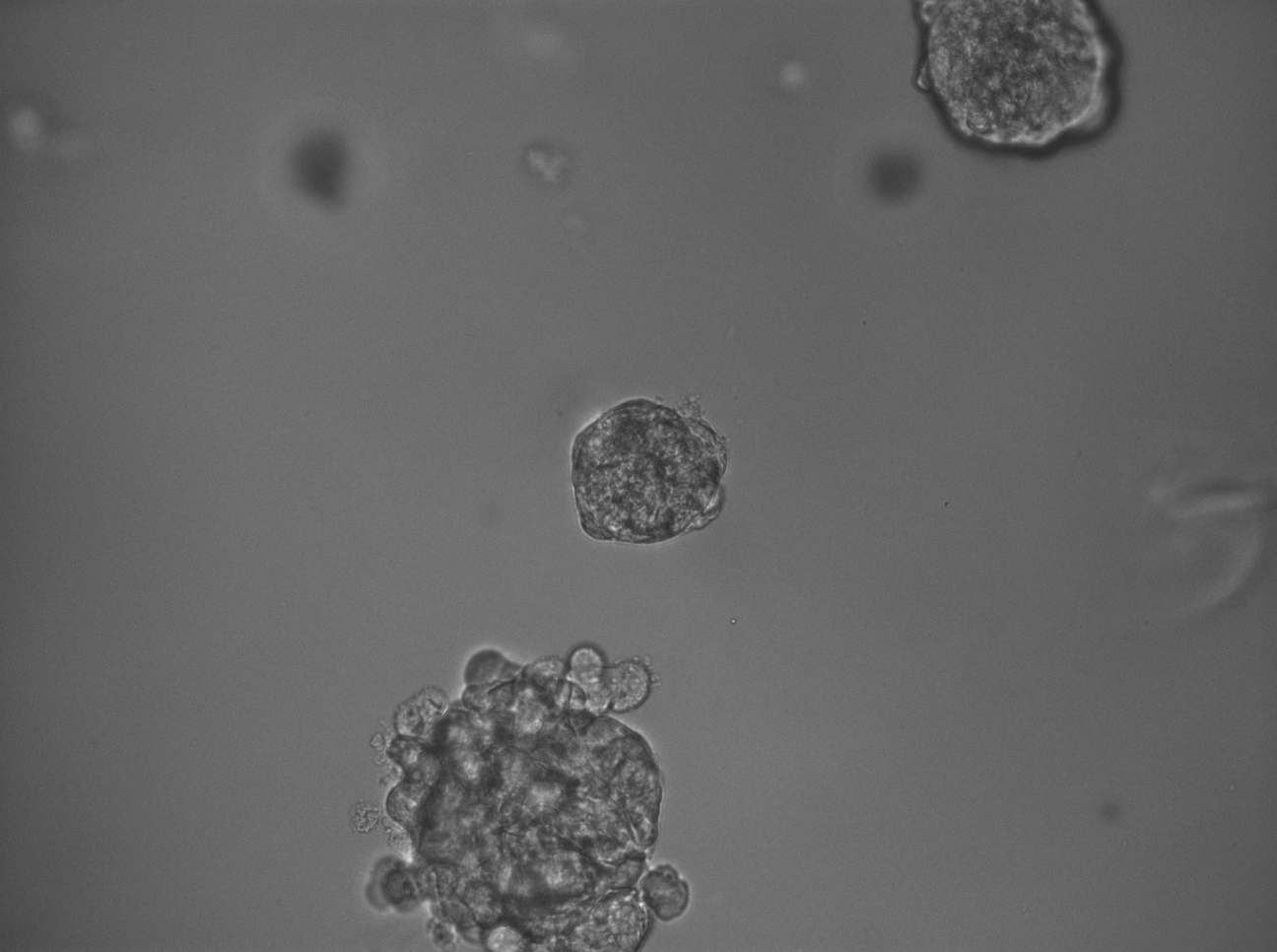

Supplement: Supplementary file 4 — Source Data Fig. 4 [file 41586_2026_10187_MOESM4_ESM.zip › HCEC1CT/HCEC1CT-KRAS_D10_Dox-00000_A03b_20x_ch00.jpg]

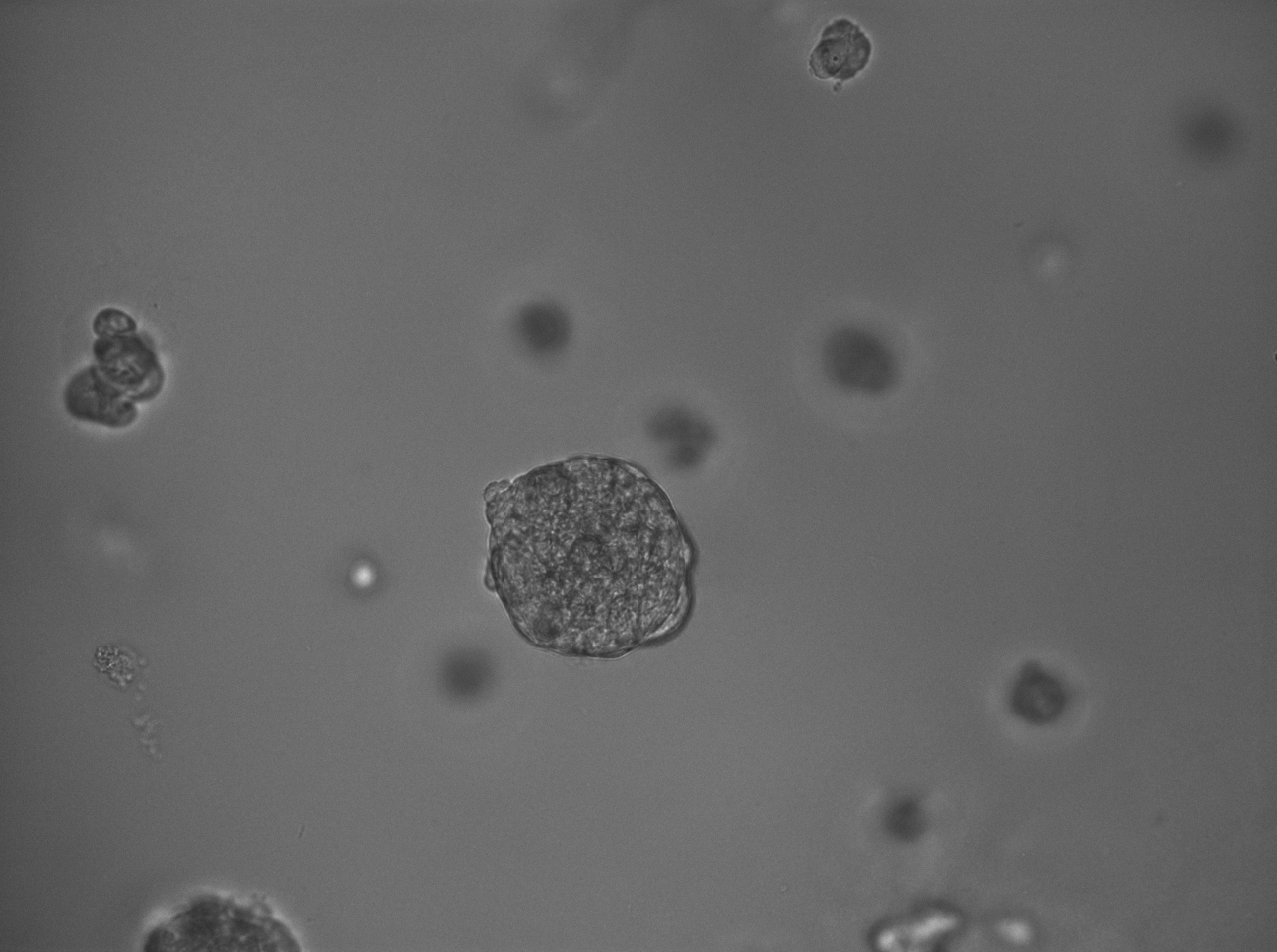

Supplement: Supplementary file 4 — Source Data Fig. 4 [file 41586_2026_10187_MOESM4_ESM.zip › HCEC1CT/HCEC1CT-KRAS_D10_Dox-00000_A03c_20x_ch00.jpg]

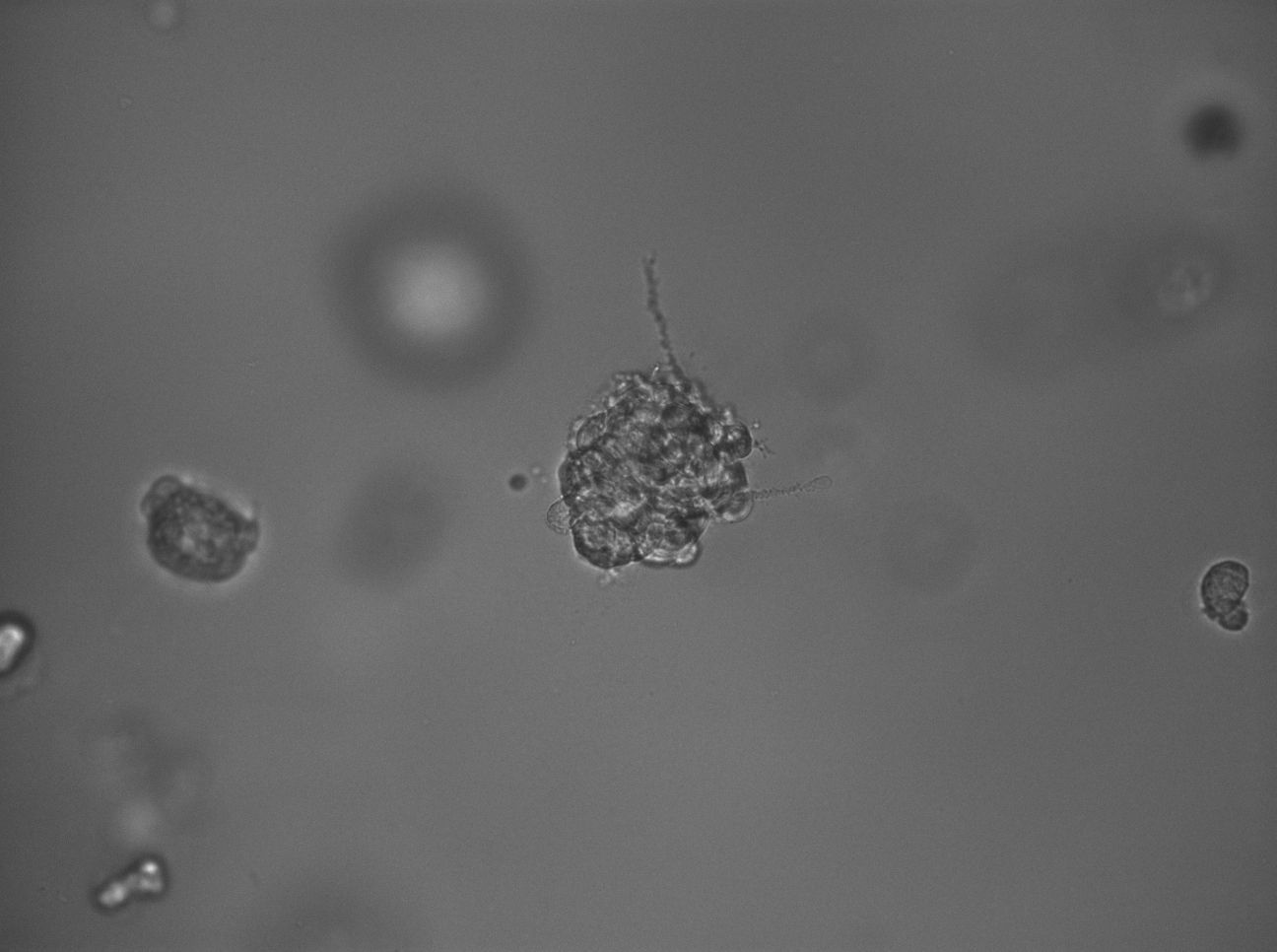

Supplement: Supplementary file 4 — Source Data Fig. 4 [file 41586_2026_10187_MOESM4_ESM.zip › HCEC1CT/HCEC1CT-KRAS_D10_Dox-00000_A03d_20x_ch00.jpg]

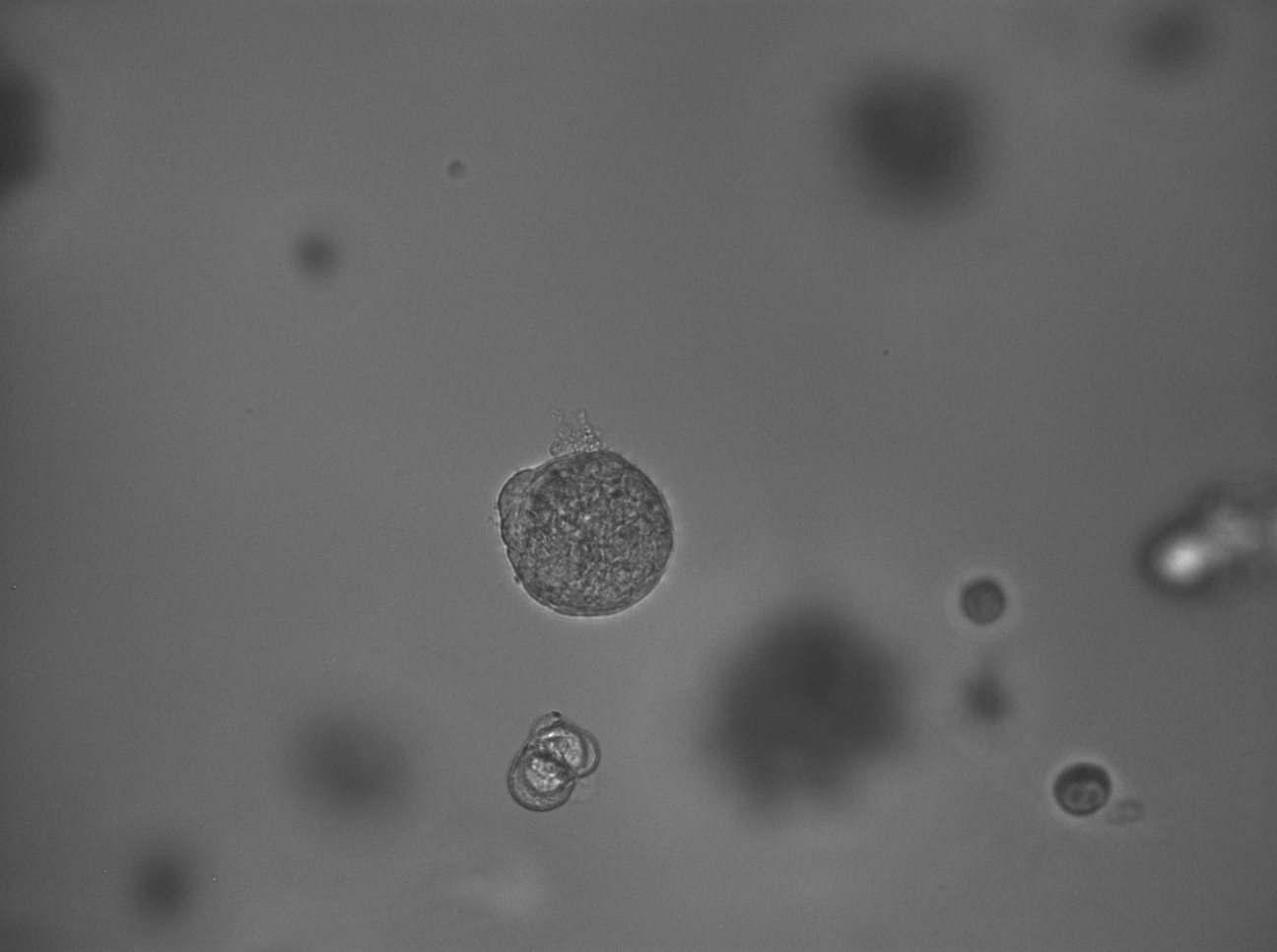

Supplement: Supplementary file 4 — Source Data Fig. 4 [file 41586_2026_10187_MOESM4_ESM.zip › HCEC1CT/HCEC1CT-KRAS_D10_Dox-00000_A03e_20x_ch00.jpg]

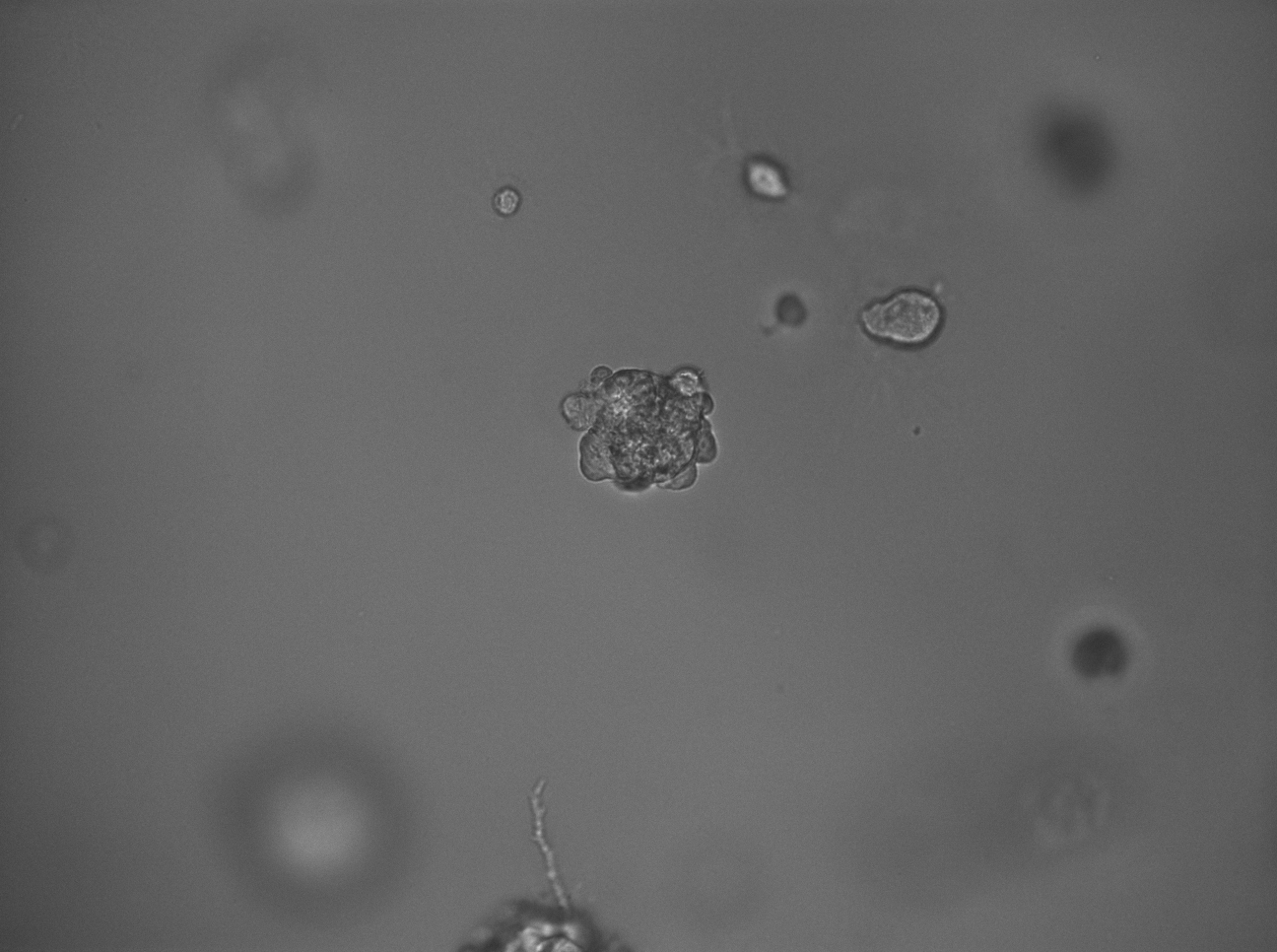

Supplement: Supplementary file 4 — Source Data Fig. 4 [file 41586_2026_10187_MOESM4_ESM.zip › HCEC1CT/HCEC1CT-KRAS_D10_Dox-00000_A03f_20x_ch00.jpg]

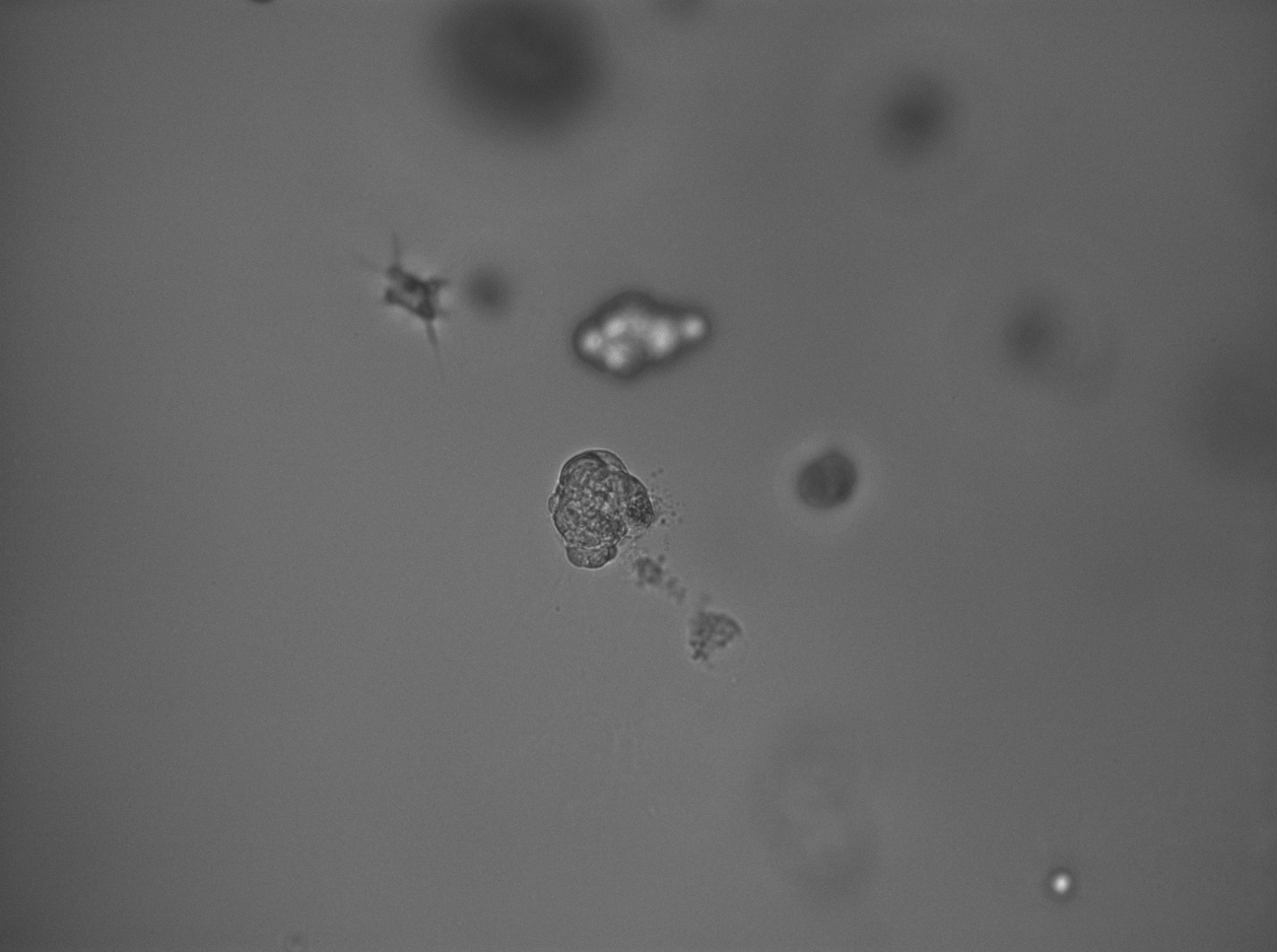

Supplement: Supplementary file 4 — Source Data Fig. 4 [file 41586_2026_10187_MOESM4_ESM.zip › HCEC1CT/HCEC1CT-KRAS_D10_Dox-00000_A03g_20x_ch00.jpg]

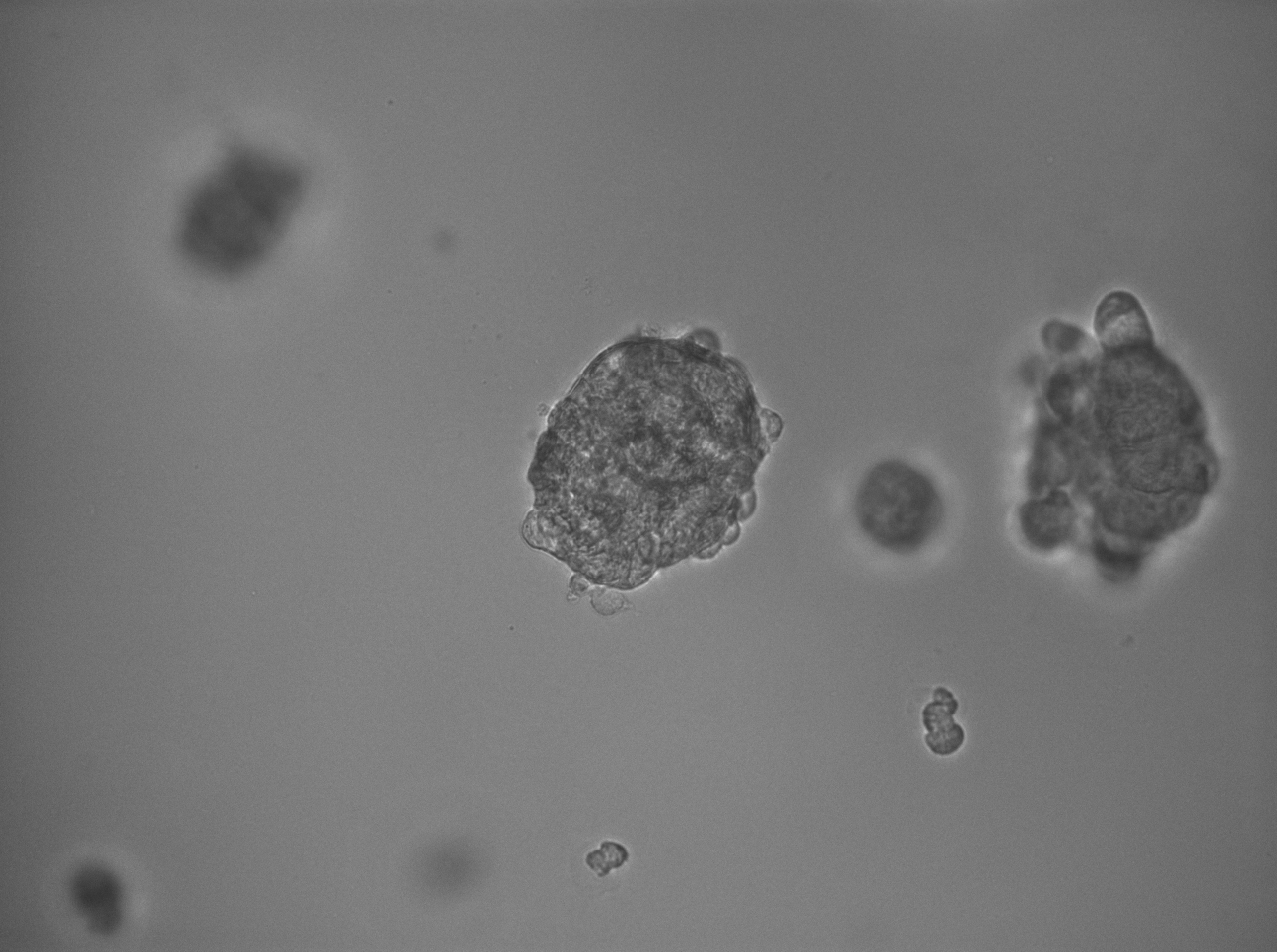

Supplement: Supplementary file 4 — Source Data Fig. 4 [file 41586_2026_10187_MOESM4_ESM.zip › HCEC1CT/HCEC1CT-KRAS_D10_Dox-00063_B01a_20x_ch00.jpg]

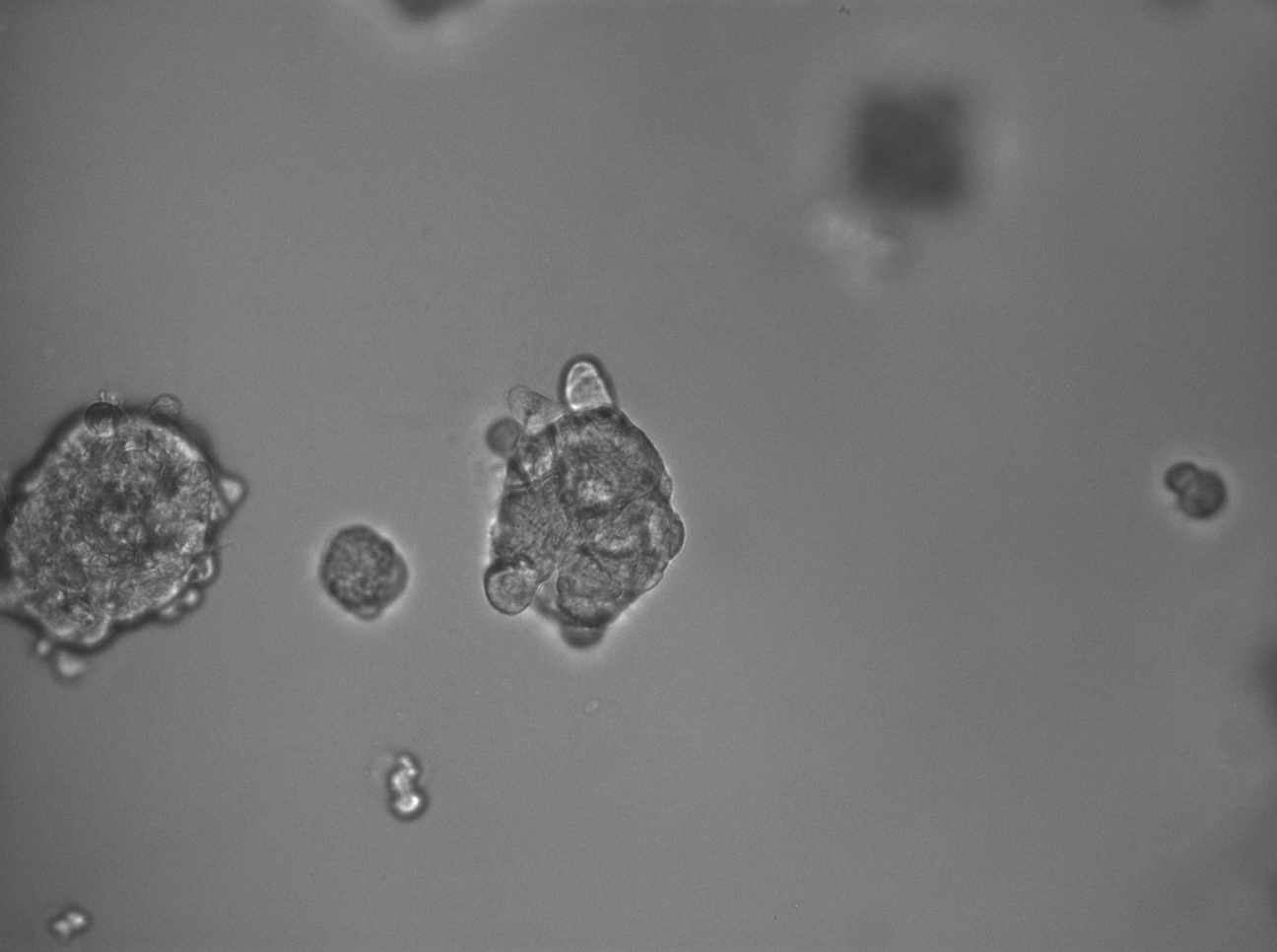

Supplement: Supplementary file 4 — Source Data Fig. 4 [file 41586_2026_10187_MOESM4_ESM.zip › HCEC1CT/HCEC1CT-KRAS_D10_Dox-00063_B01b_20x_ch00.jpg]

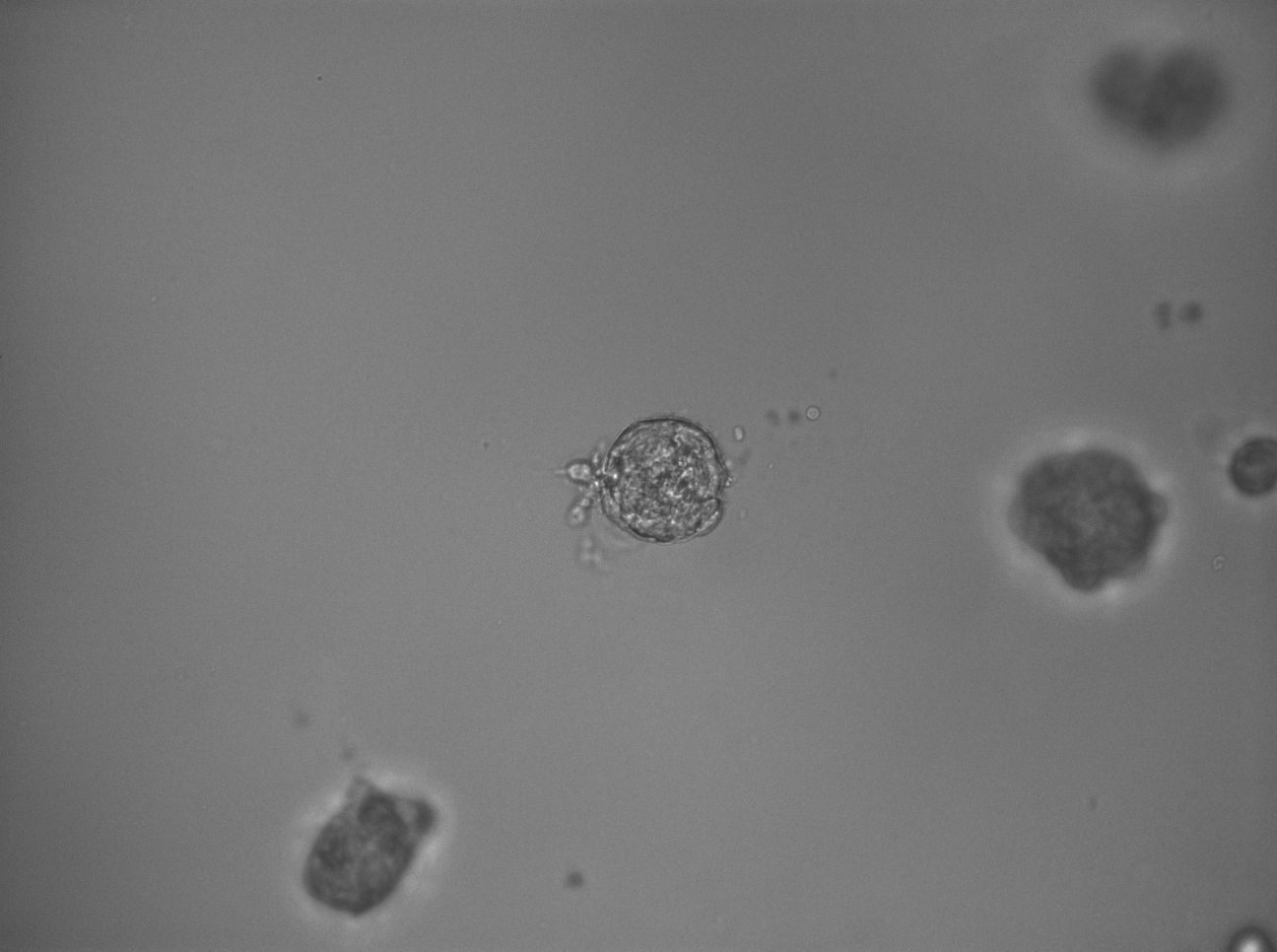

Supplement: Supplementary file 4 — Source Data Fig. 4 [file 41586_2026_10187_MOESM4_ESM.zip › HCEC1CT/HCEC1CT-KRAS_D10_Dox-00063_B01c_20x_ch00.jpg]

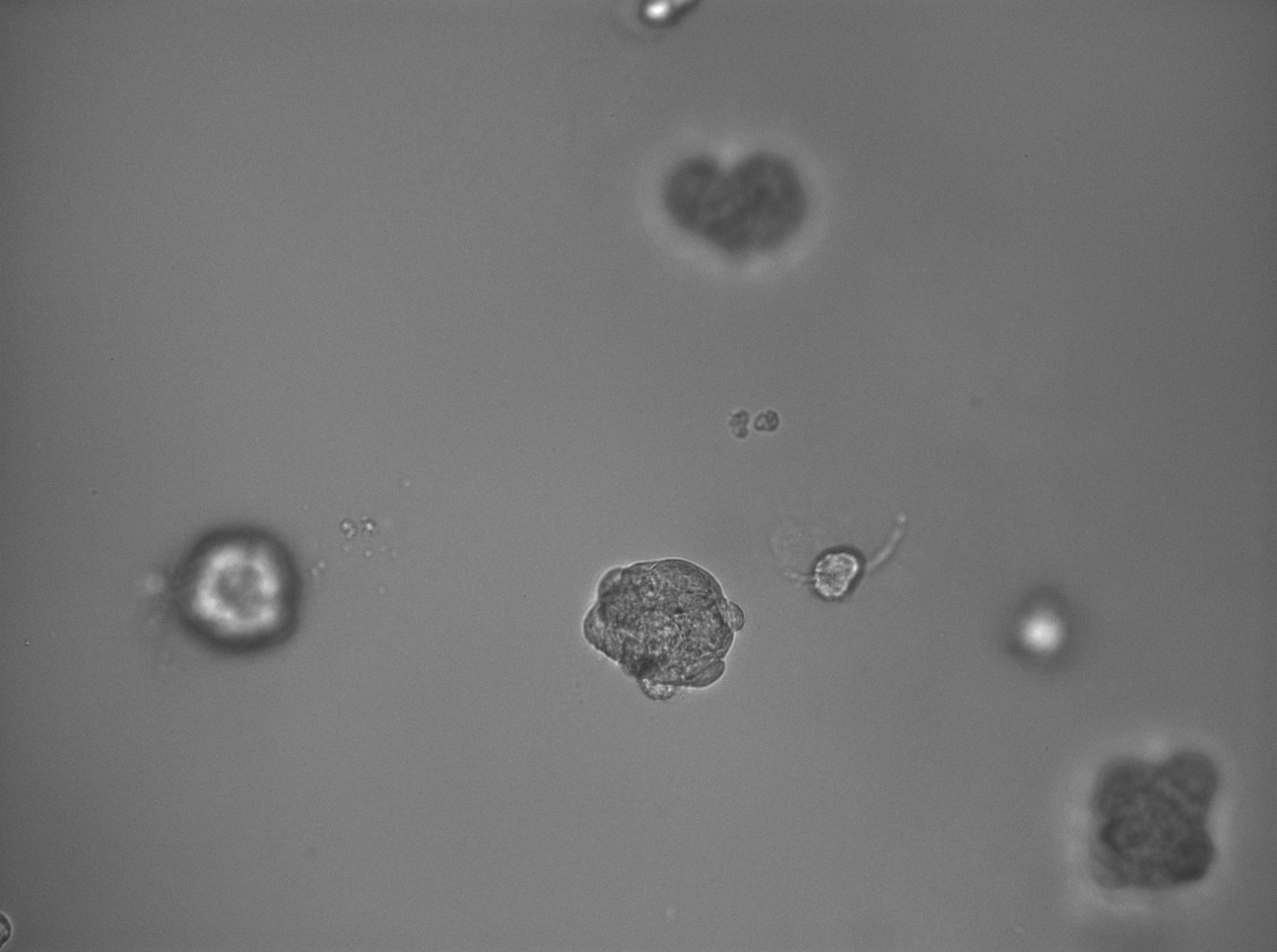

Supplement: Supplementary file 4 — Source Data Fig. 4 [file 41586_2026_10187_MOESM4_ESM.zip › HCEC1CT/HCEC1CT-KRAS_D10_Dox-00063_B01d_20x_ch00.jpg]

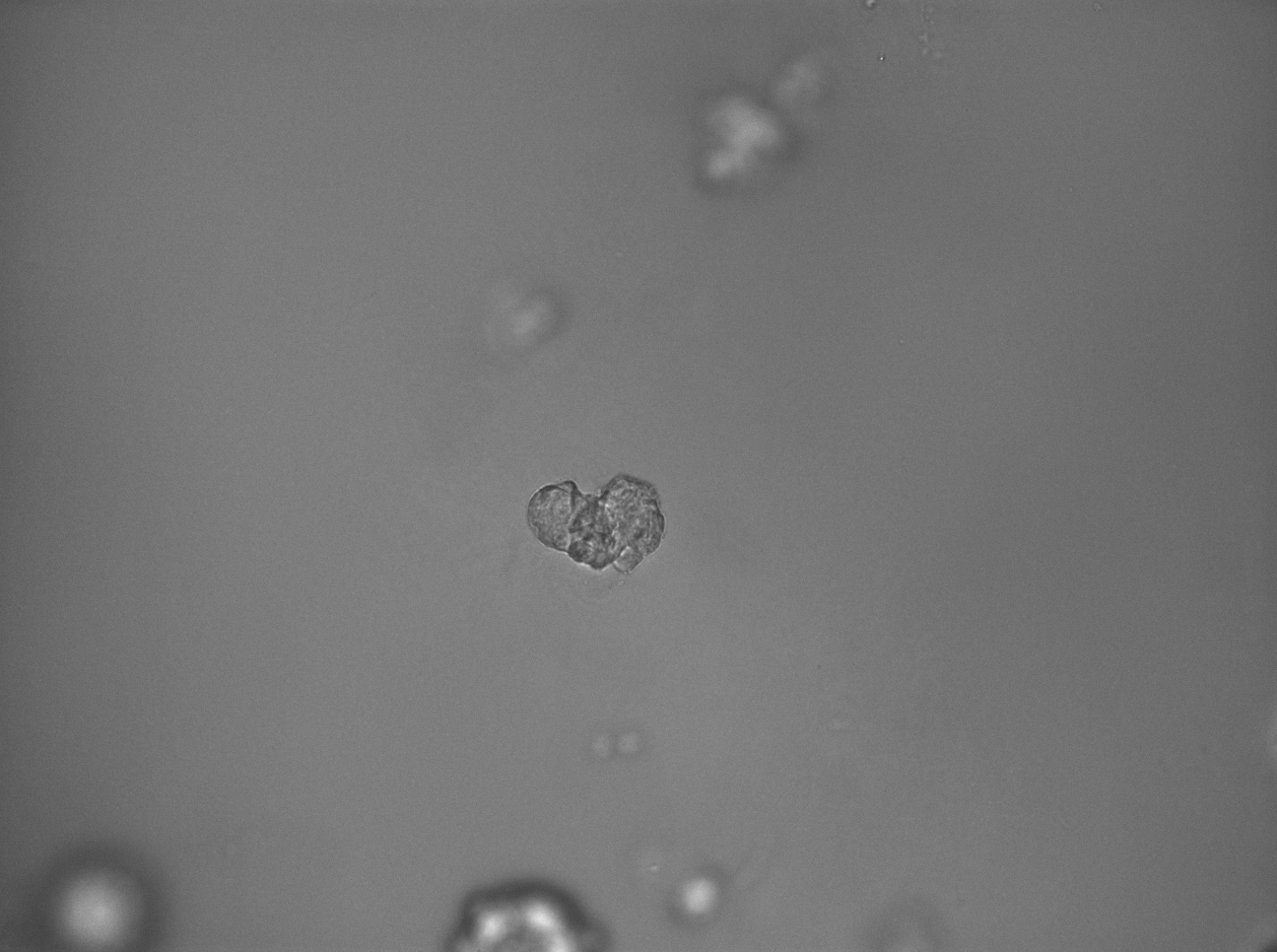

Supplement: Supplementary file 4 — Source Data Fig. 4 [file 41586_2026_10187_MOESM4_ESM.zip › HCEC1CT/HCEC1CT-KRAS_D10_Dox-00063_B01e_20x_ch00.jpg]

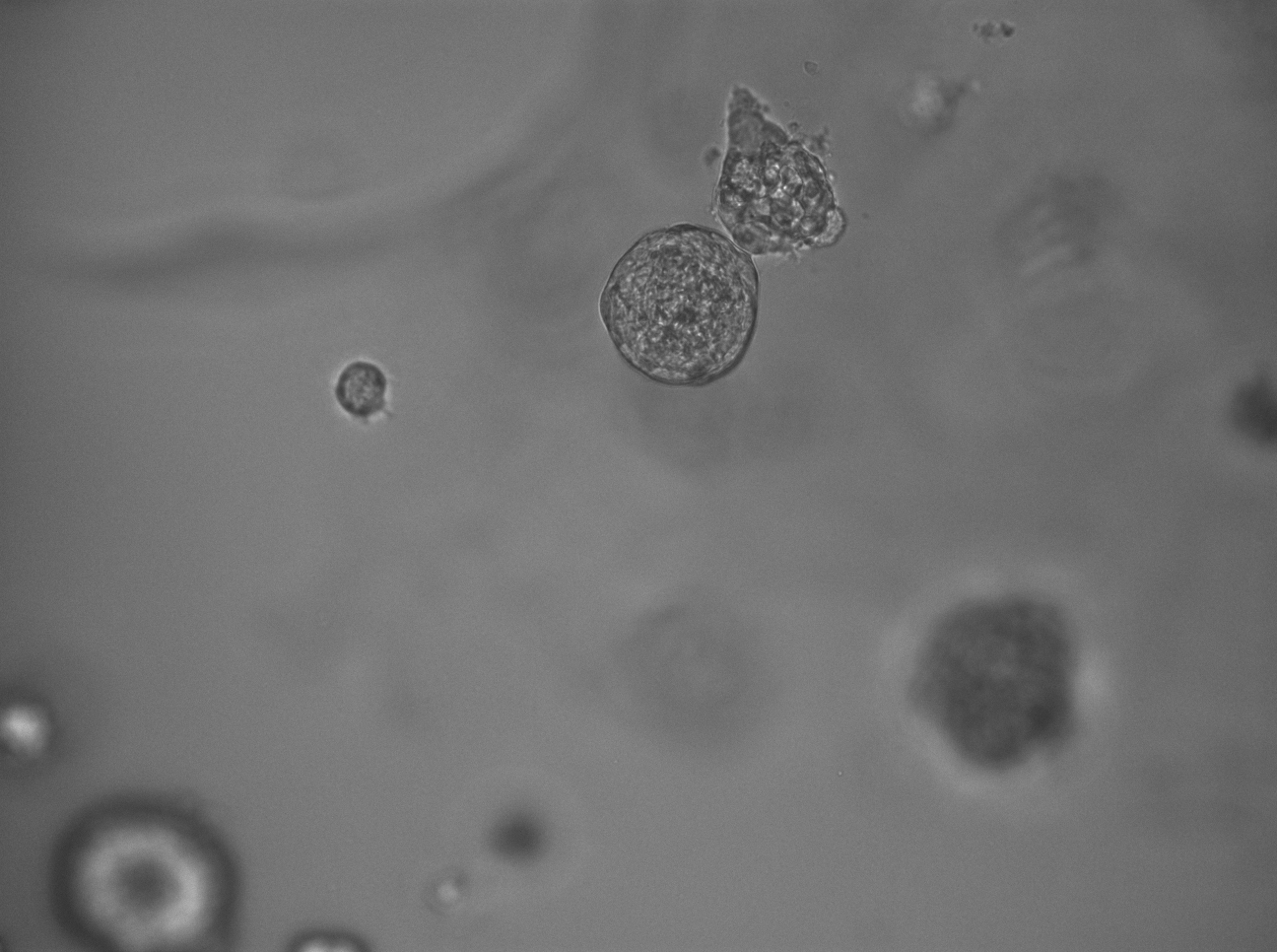

Supplement: Supplementary file 4 — Source Data Fig. 4 [file 41586_2026_10187_MOESM4_ESM.zip › HCEC1CT/HCEC1CT-KRAS_D10_Dox-00063_B01f_20x_ch00.jpg]

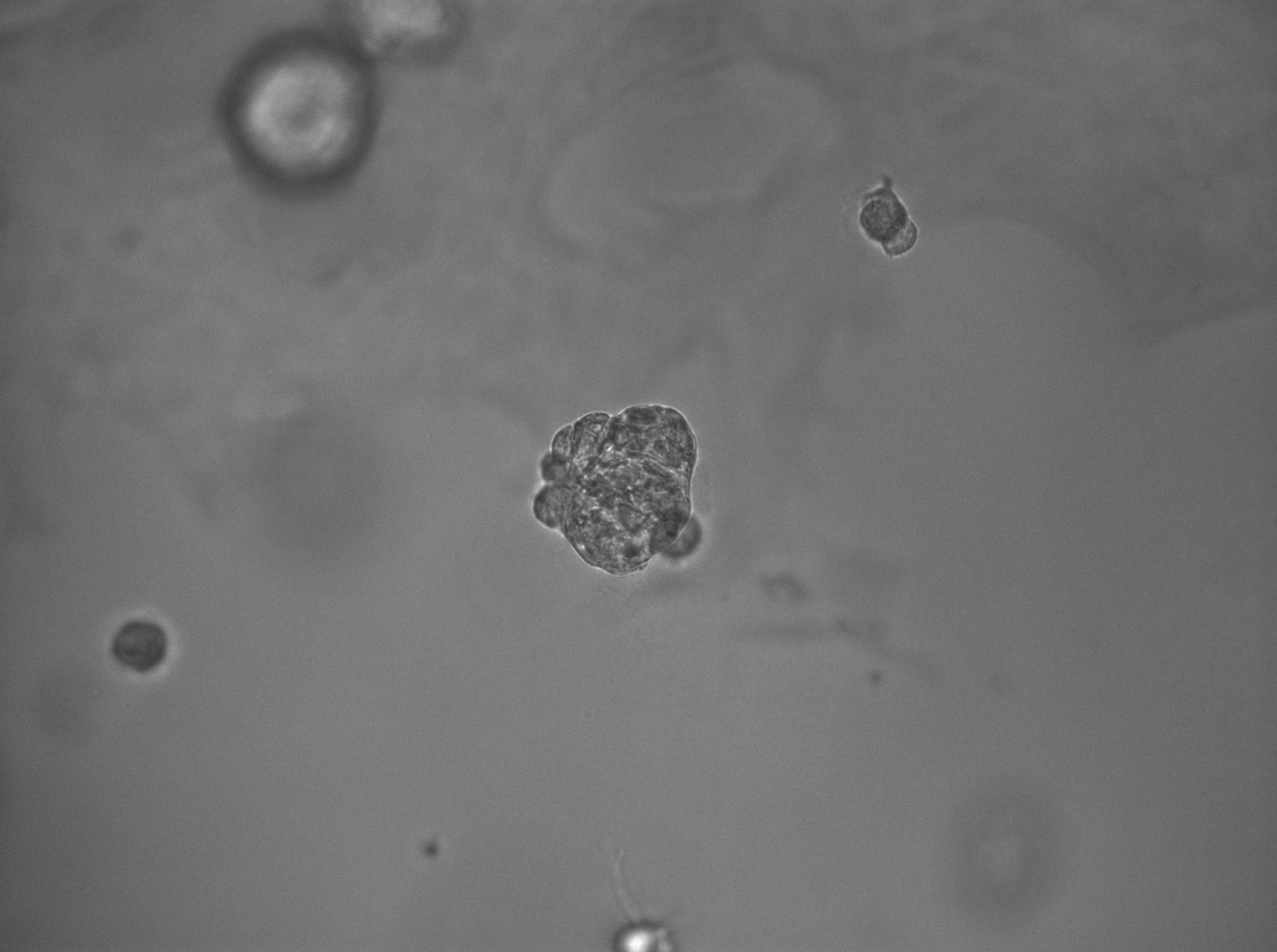

Supplement: Supplementary file 4 — Source Data Fig. 4 [file 41586_2026_10187_MOESM4_ESM.zip › HCEC1CT/HCEC1CT-KRAS_D10_Dox-00063_B01g_20x_ch00.jpg]

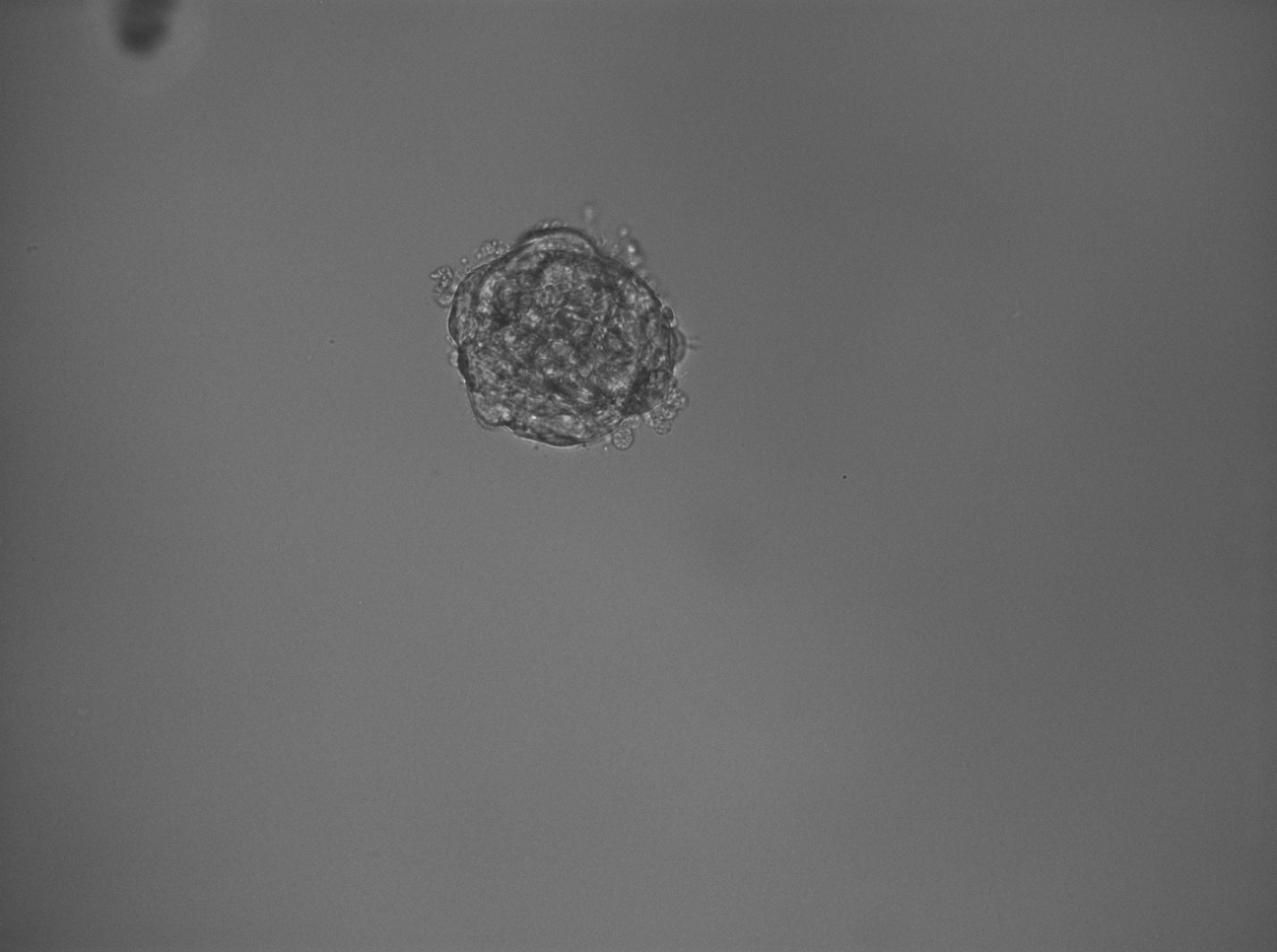

Supplement: Supplementary file 4 — Source Data Fig. 4 [file 41586_2026_10187_MOESM4_ESM.zip › HCEC1CT/HCEC1CT-KRAS_D10_Dox-00063_B02a_20x_ch00.jpg]

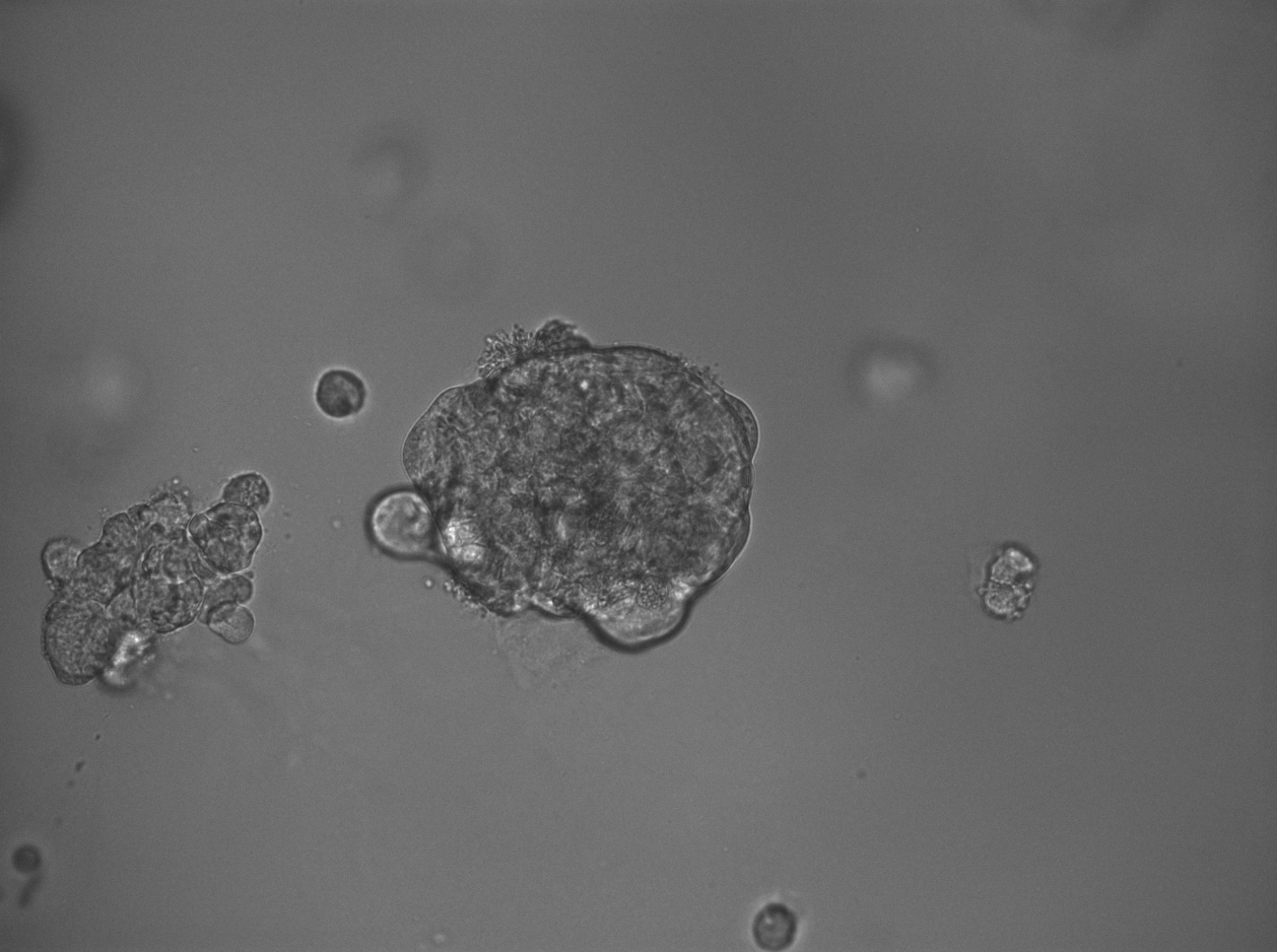

Supplement: Supplementary file 4 — Source Data Fig. 4 [file 41586_2026_10187_MOESM4_ESM.zip › HCEC1CT/HCEC1CT-KRAS_D10_Dox-00063_B02b_20x_ch00.jpg]

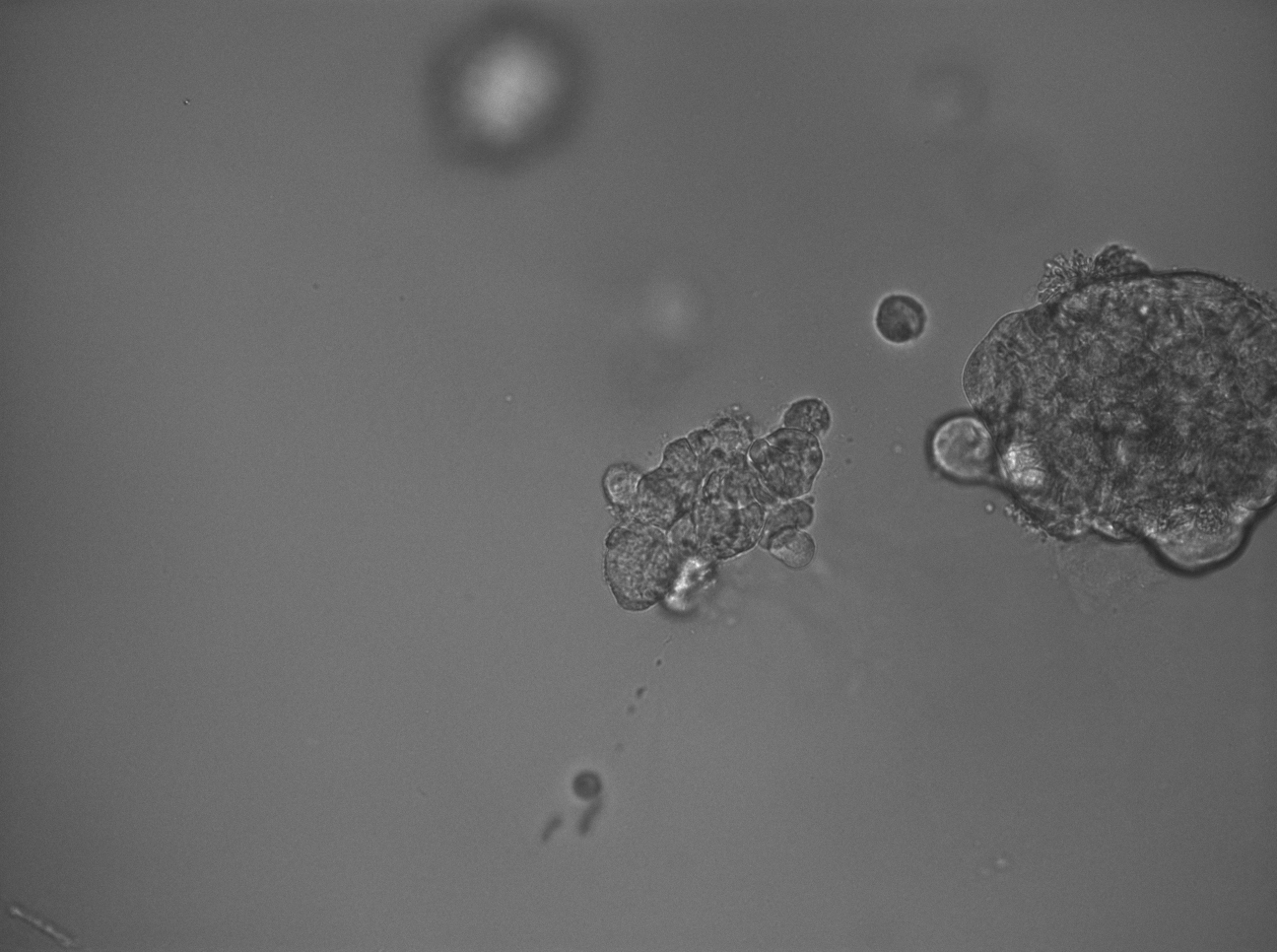

Supplement: Supplementary file 4 — Source Data Fig. 4 [file 41586_2026_10187_MOESM4_ESM.zip › HCEC1CT/HCEC1CT-KRAS_D10_Dox-00063_B02c_20x_ch00.jpg]

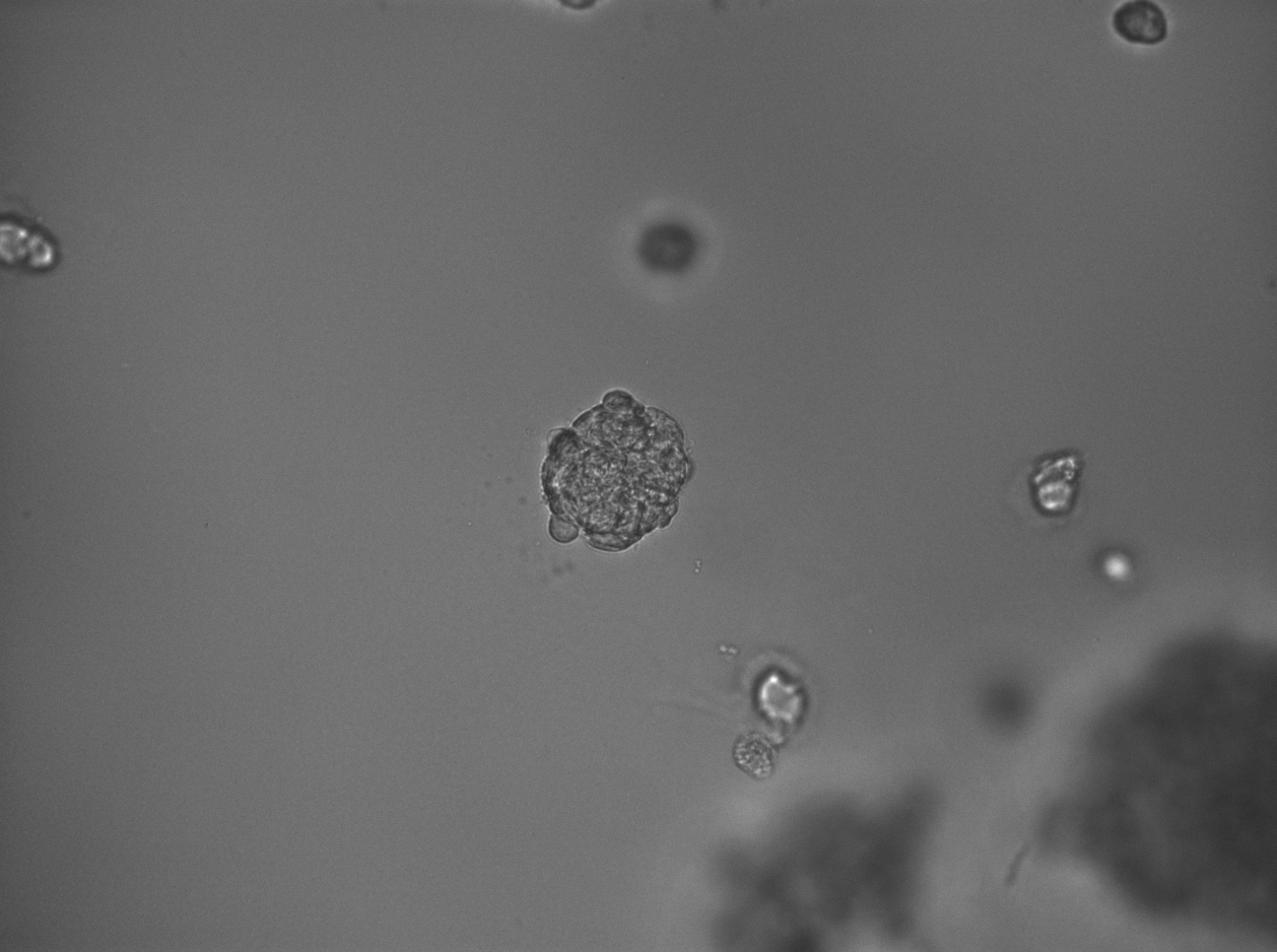

Supplement: Supplementary file 4 — Source Data Fig. 4 [file 41586_2026_10187_MOESM4_ESM.zip › HCEC1CT/HCEC1CT-KRAS_D10_Dox-00063_B02d_20x_ch00.jpg]

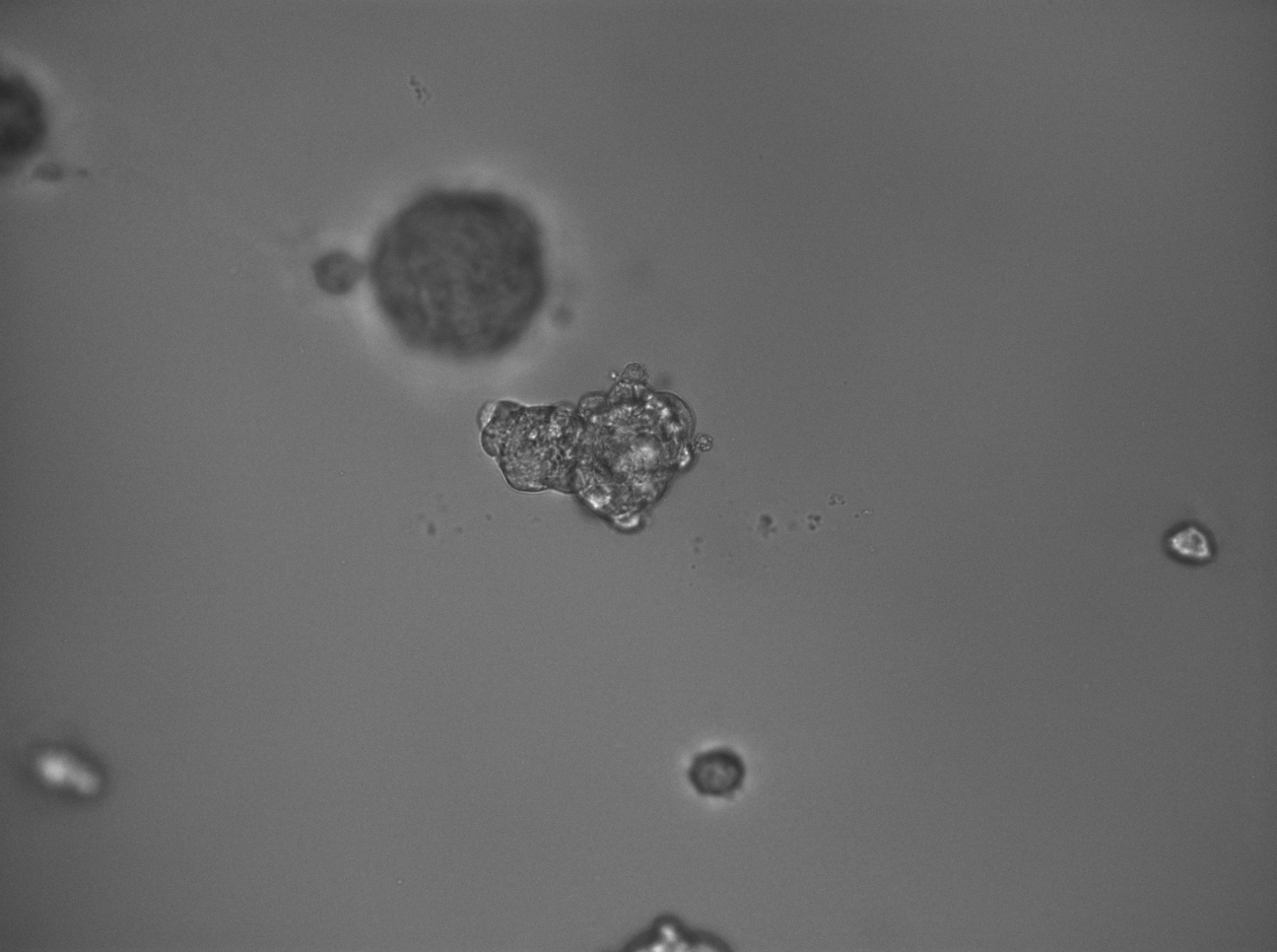

Supplement: Supplementary file 4 — Source Data Fig. 4 [file 41586_2026_10187_MOESM4_ESM.zip › HCEC1CT/HCEC1CT-KRAS_D10_Dox-00063_B02e_20x_ch00.jpg]

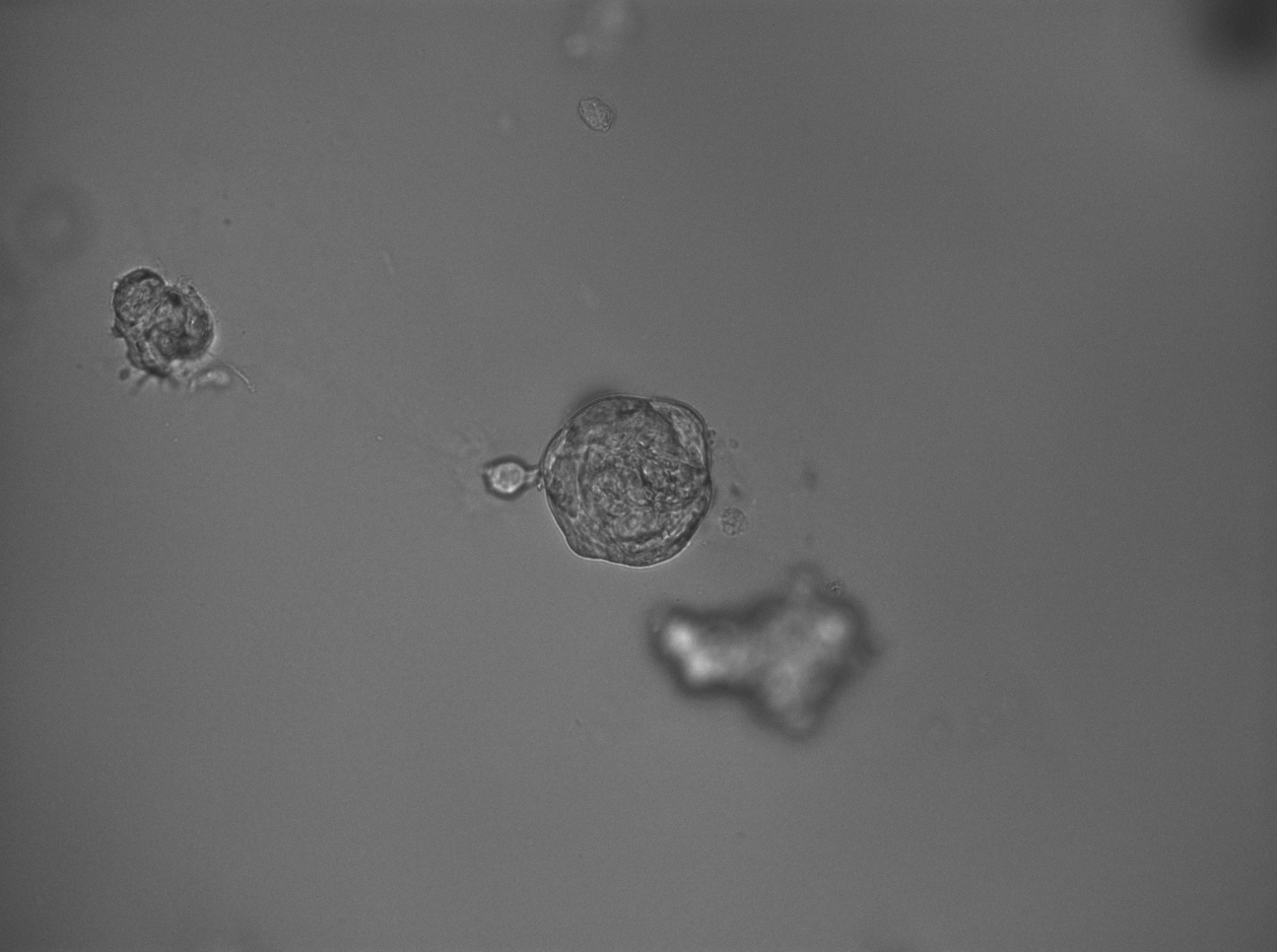

Supplement: Supplementary file 4 — Source Data Fig. 4 [file 41586_2026_10187_MOESM4_ESM.zip › HCEC1CT/HCEC1CT-KRAS_D10_Dox-00063_B02f_20x_ch00.jpg]

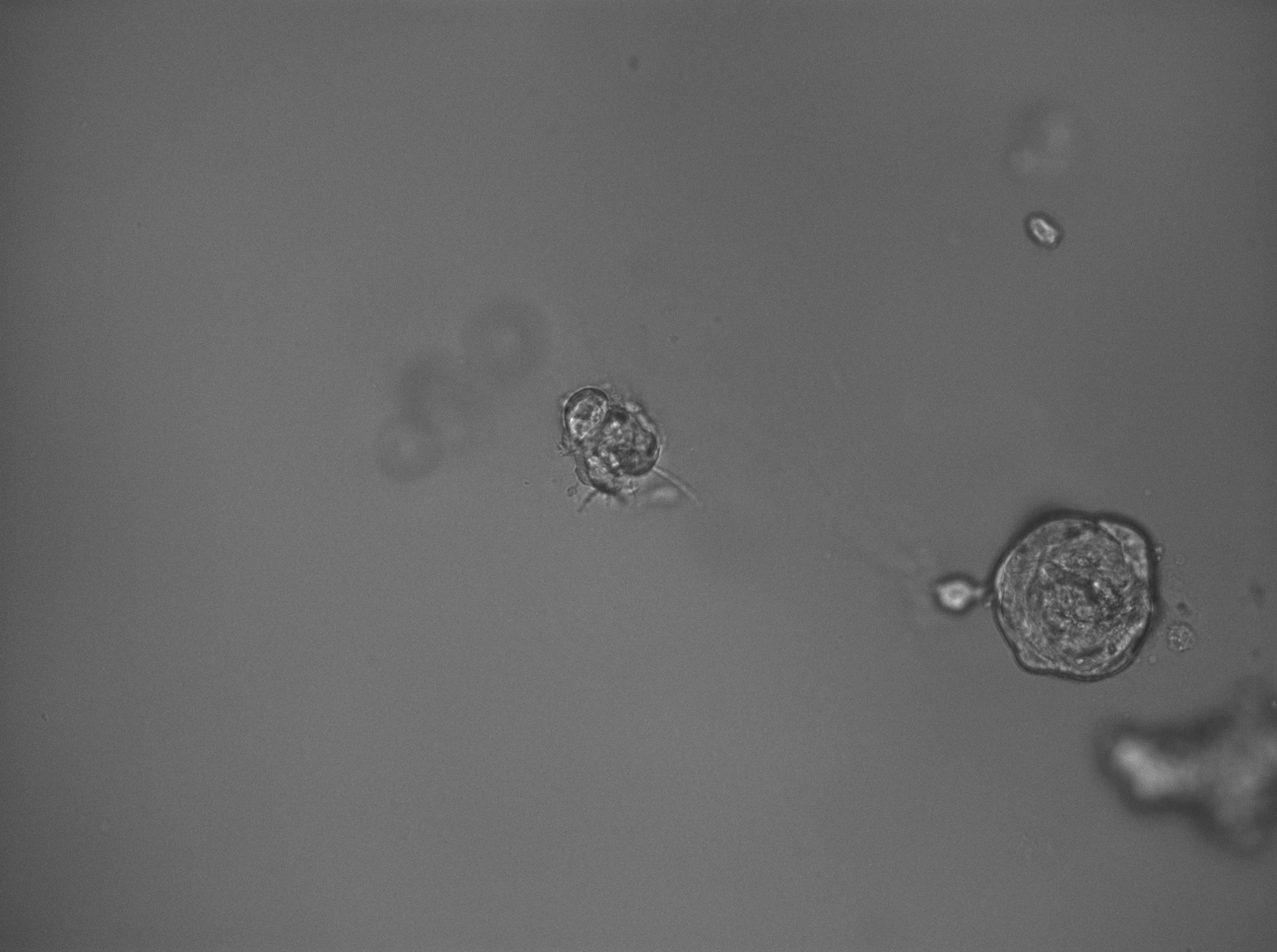

Supplement: Supplementary file 4 — Source Data Fig. 4 [file 41586_2026_10187_MOESM4_ESM.zip › HCEC1CT/HCEC1CT-KRAS_D10_Dox-00063_B02g_20x_ch00.jpg]

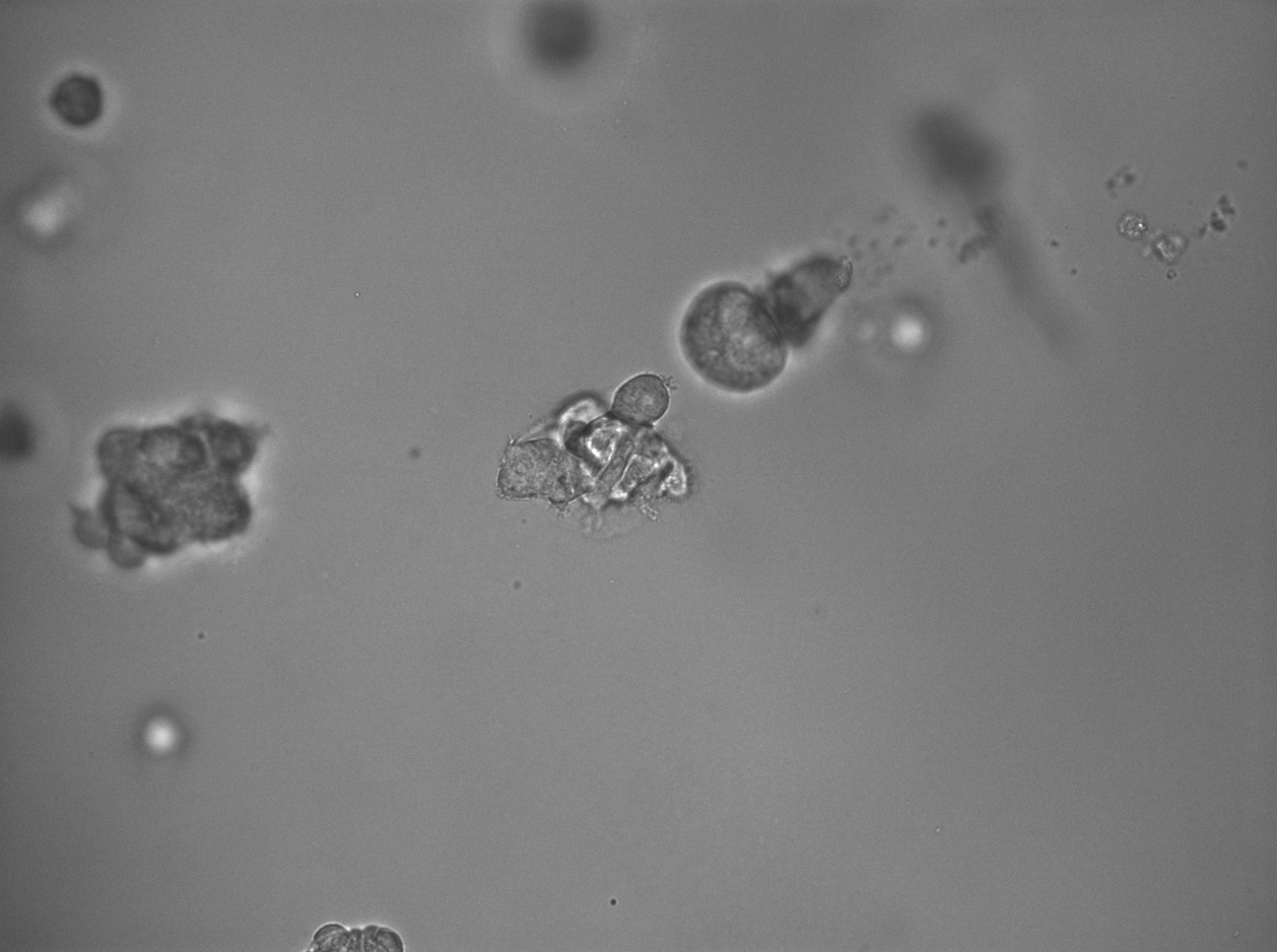

Supplement: Supplementary file 4 — Source Data Fig. 4 [file 41586_2026_10187_MOESM4_ESM.zip › HCEC1CT/HCEC1CT-KRAS_D10_Dox-00063_B03a_20x_ch00.jpg]

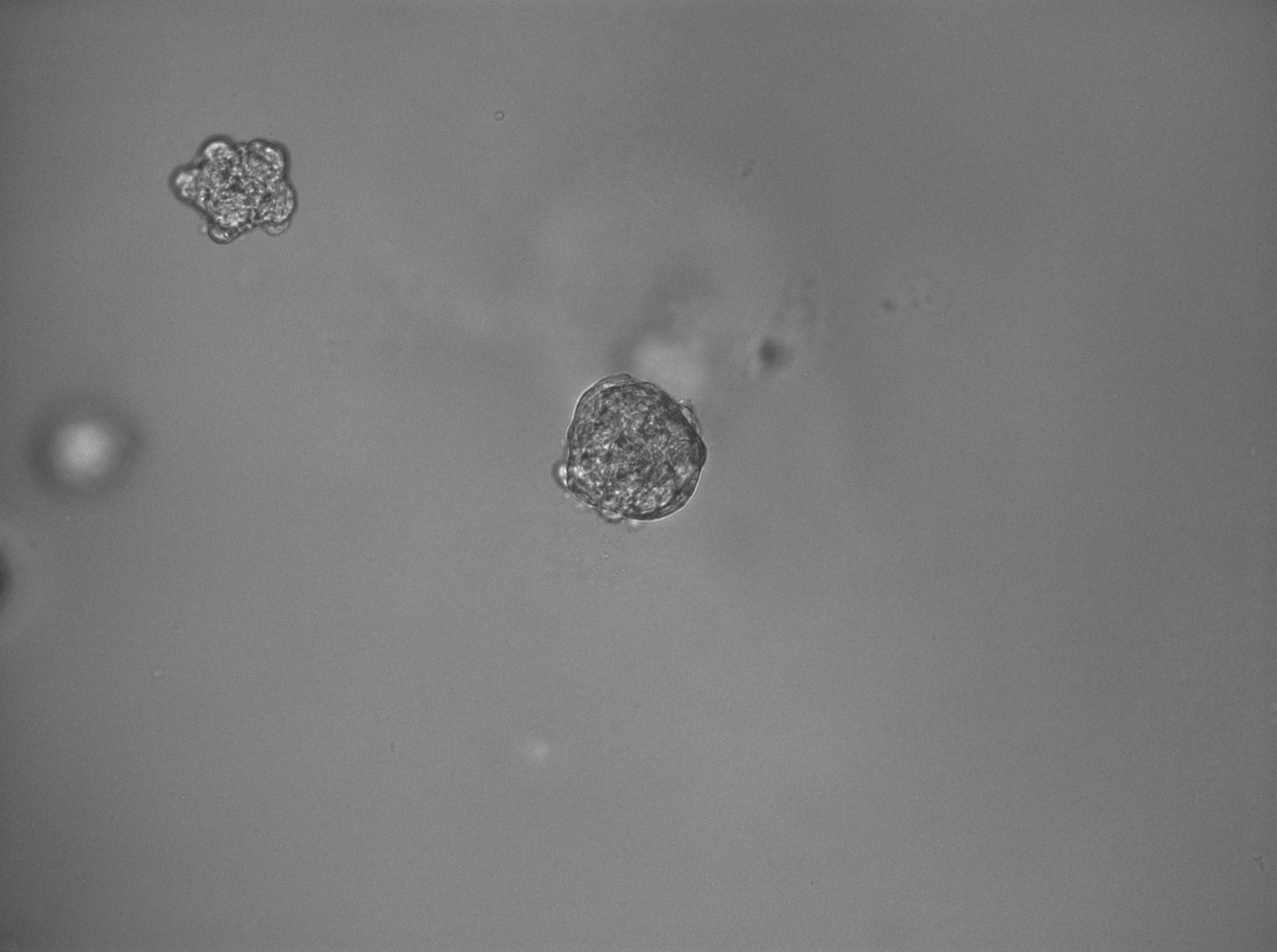

Supplement: Supplementary file 4 — Source Data Fig. 4 [file 41586_2026_10187_MOESM4_ESM.zip › HCEC1CT/HCEC1CT-KRAS_D10_Dox-00063_B03b_20x_ch00.jpg]

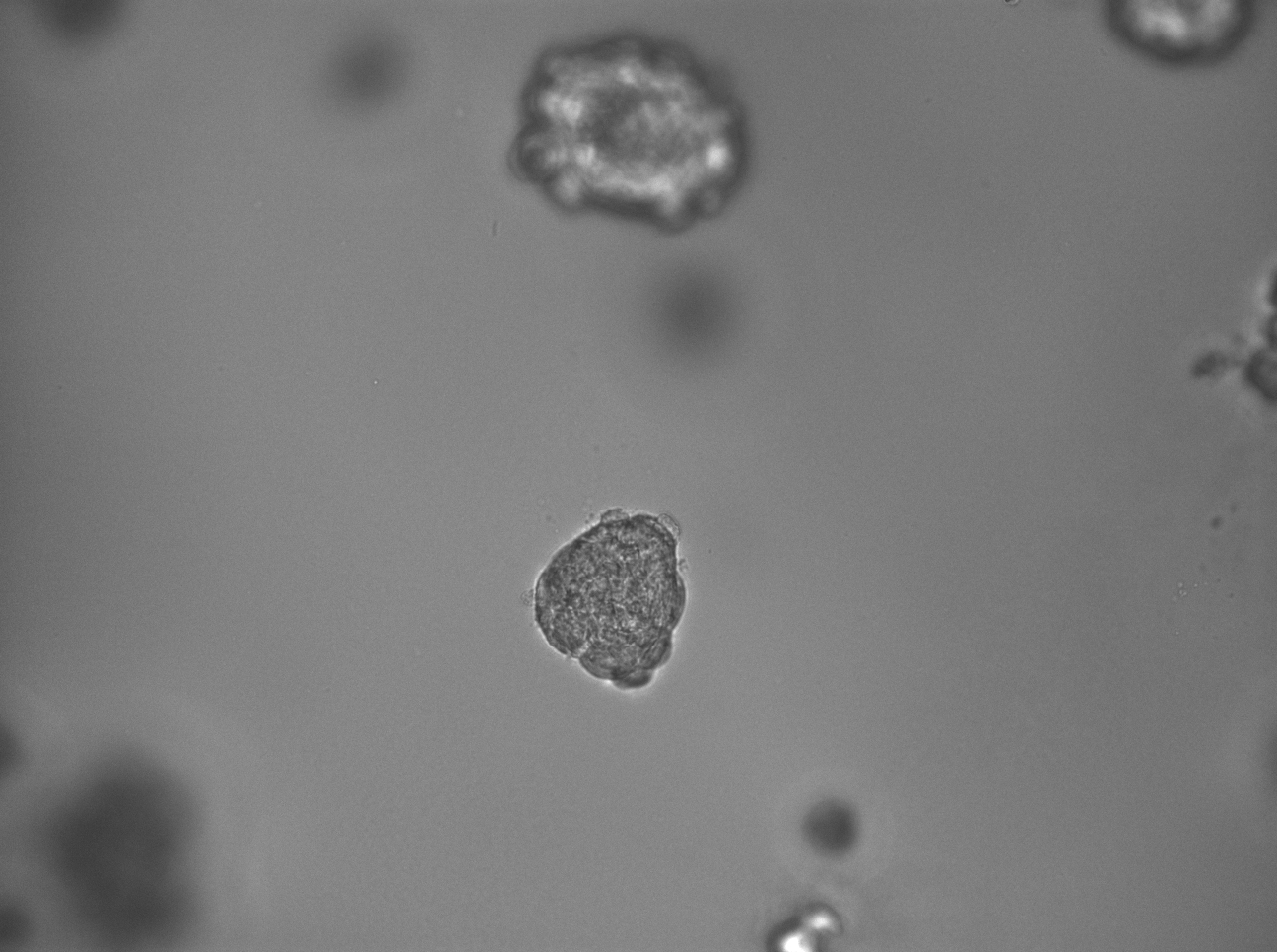

Supplement: Supplementary file 4 — Source Data Fig. 4 [file 41586_2026_10187_MOESM4_ESM.zip › HCEC1CT/HCEC1CT-KRAS_D10_Dox-00063_B03c_20x_ch00.jpg]

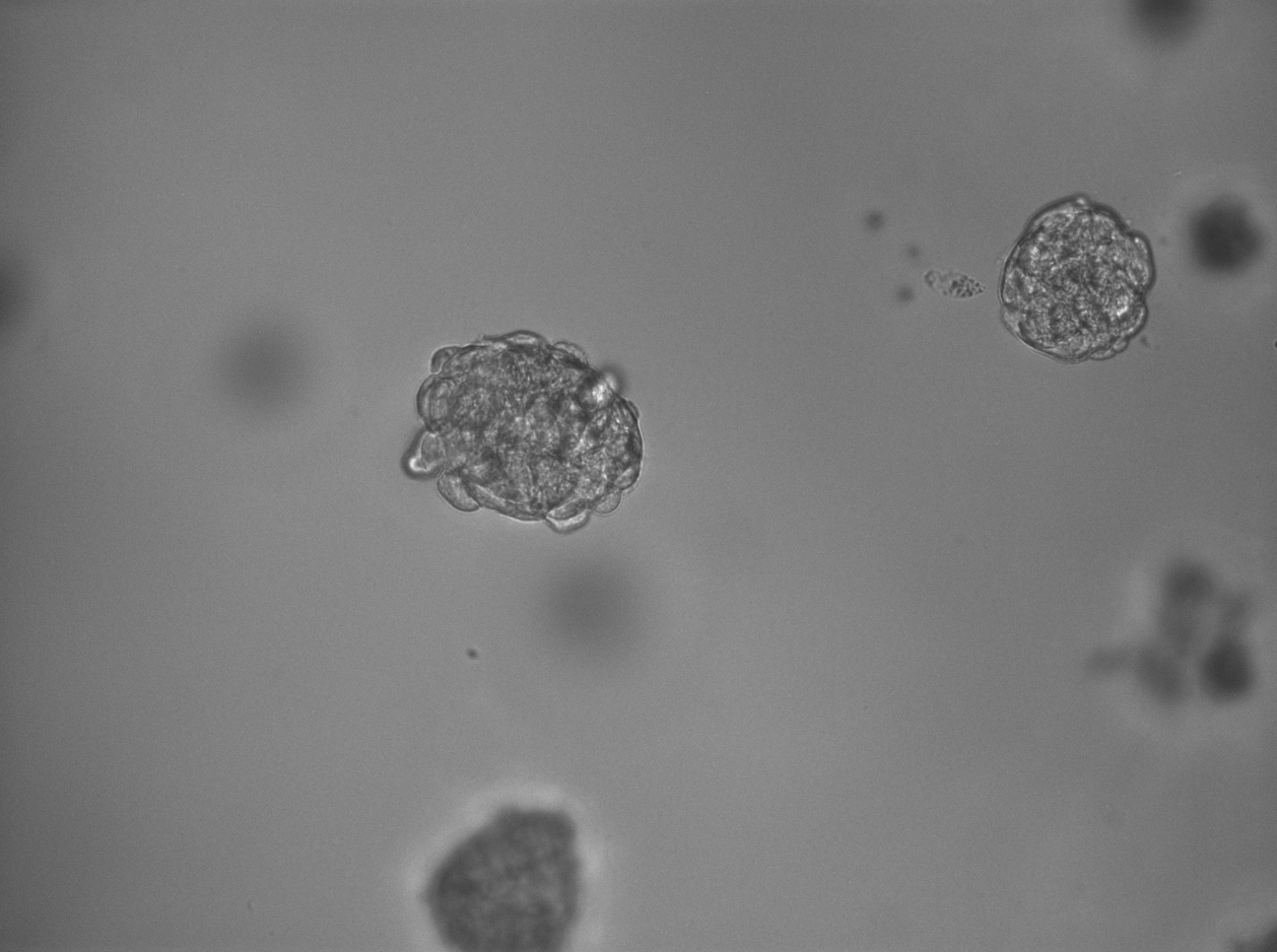

Supplement: Supplementary file 4 — Source Data Fig. 4 [file 41586_2026_10187_MOESM4_ESM.zip › HCEC1CT/HCEC1CT-KRAS_D10_Dox-00063_B03d_20x_ch00.jpg]

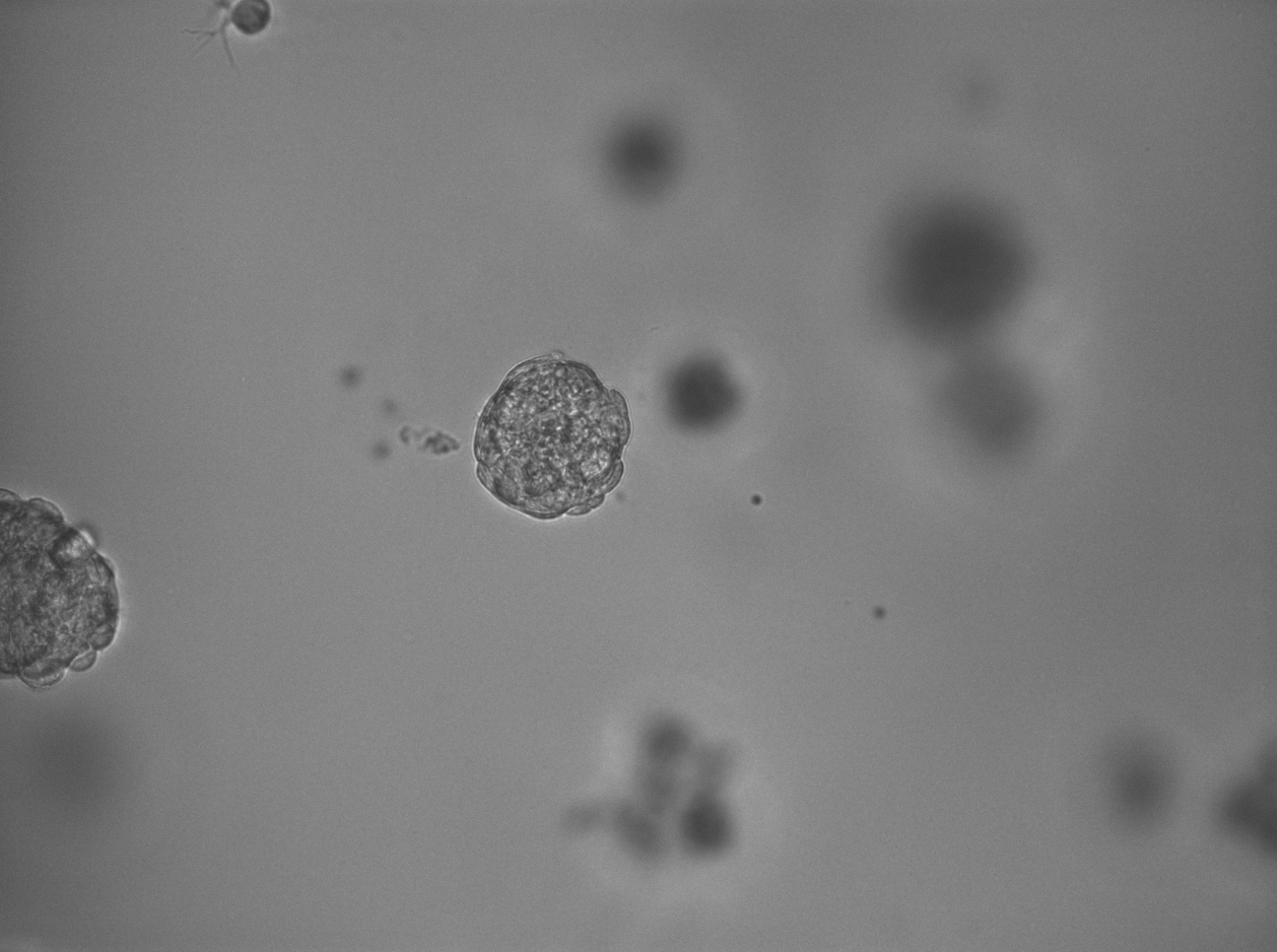

Supplement: Supplementary file 4 — Source Data Fig. 4 [file 41586_2026_10187_MOESM4_ESM.zip › HCEC1CT/HCEC1CT-KRAS_D10_Dox-00063_B03e_20x_ch00.jpg]

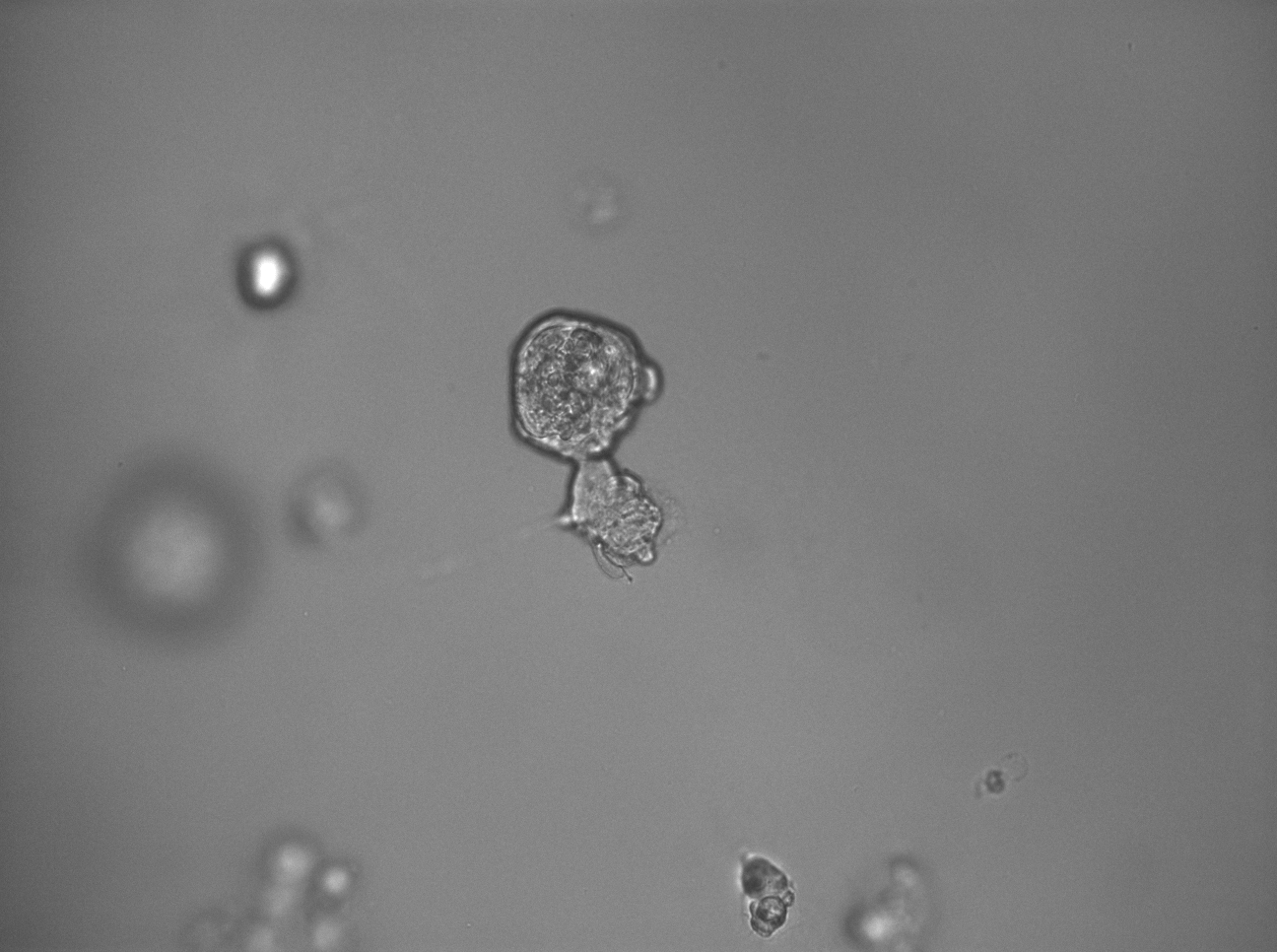

Supplement: Supplementary file 4 — Source Data Fig. 4 [file 41586_2026_10187_MOESM4_ESM.zip › HCEC1CT/HCEC1CT-KRAS_D10_Dox-00063_B03f_20x_ch00.jpg]

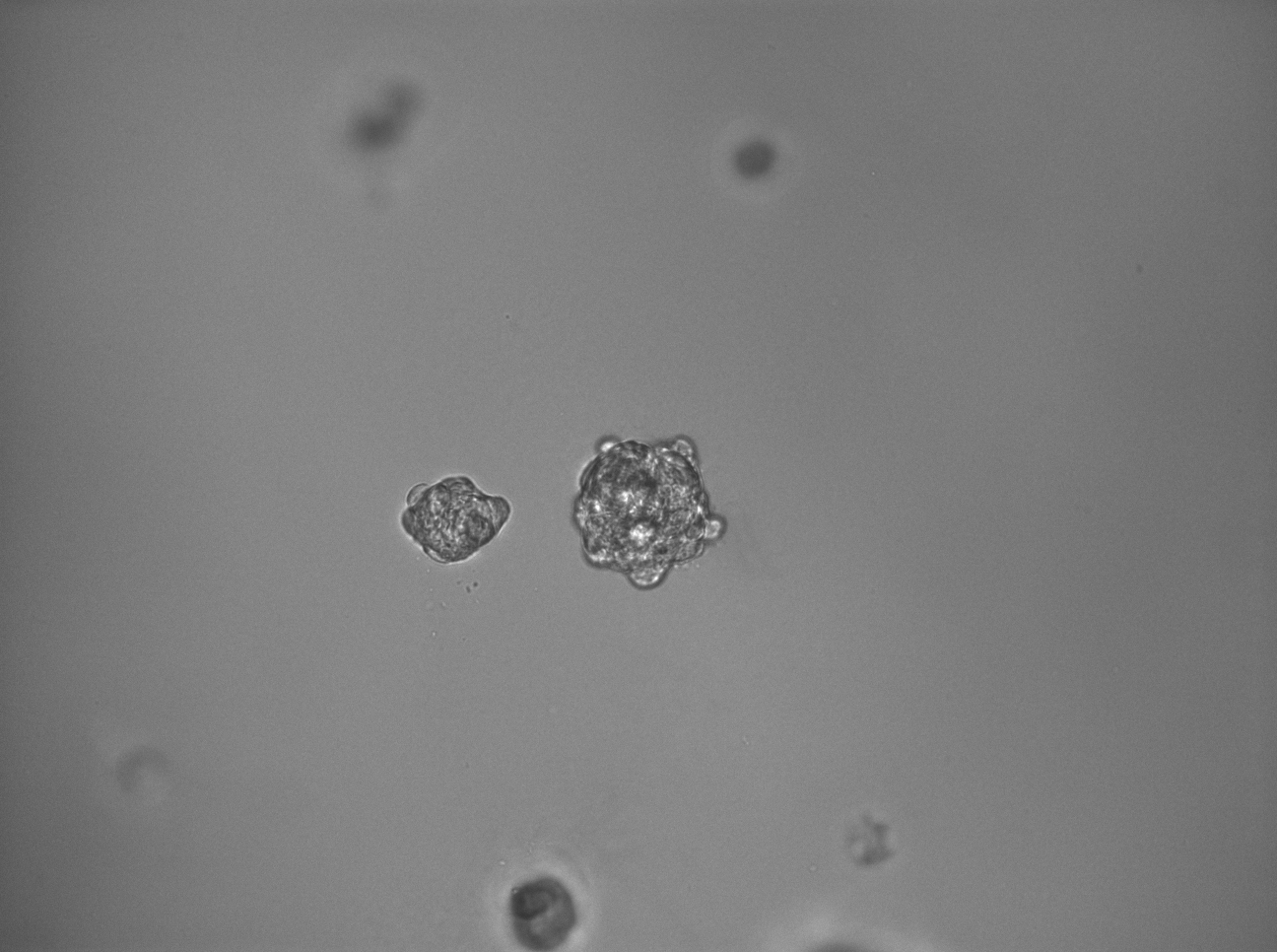

Supplement: Supplementary file 4 — Source Data Fig. 4 [file 41586_2026_10187_MOESM4_ESM.zip › HCEC1CT/HCEC1CT-KRAS_D10_Dox-00063_B03g_20x_ch00.jpg]

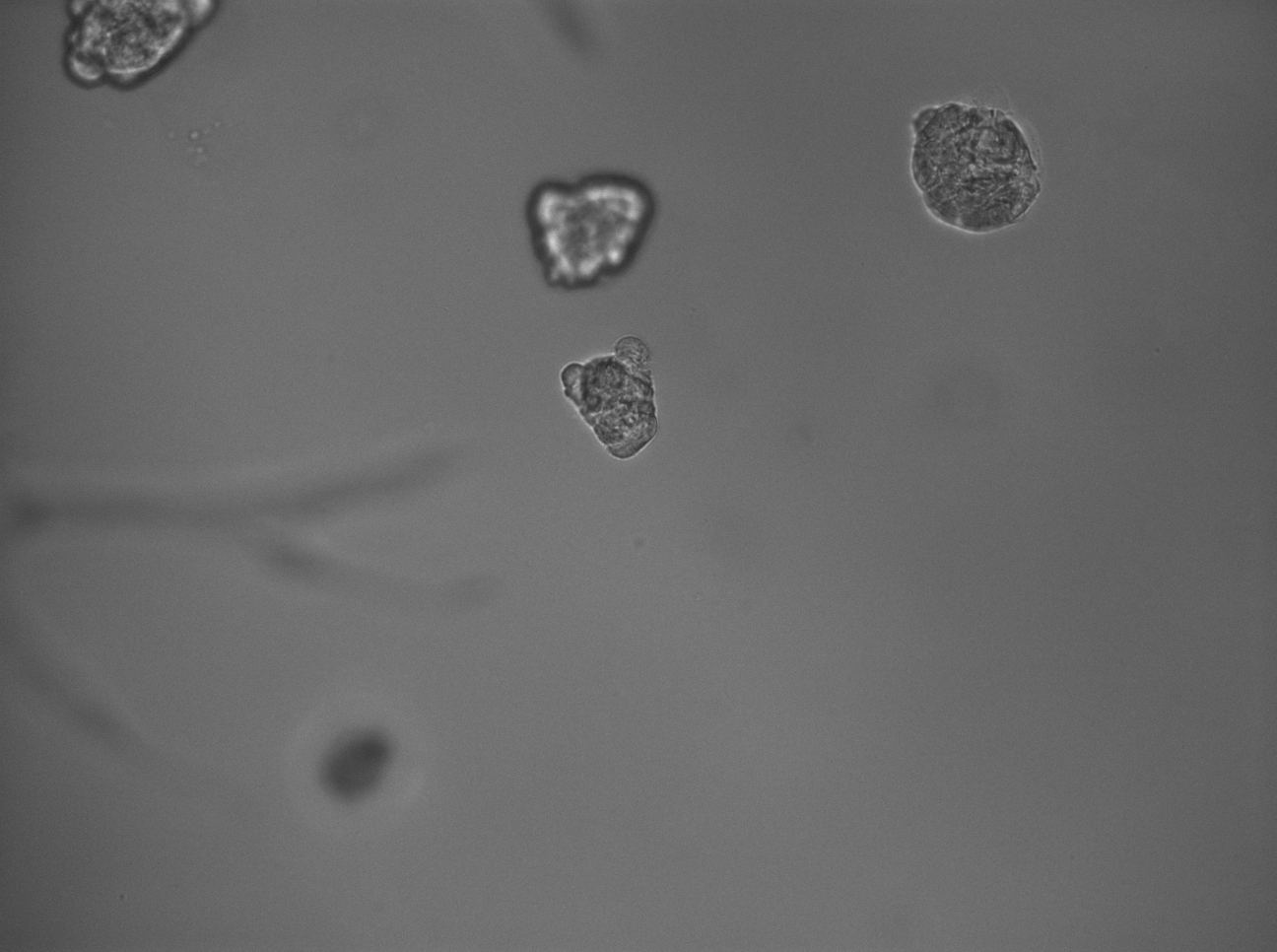

Supplement: Supplementary file 4 — Source Data Fig. 4 [file 41586_2026_10187_MOESM4_ESM.zip › HCEC1CT/HCEC1CT-KRAS_D10_Dox-00125_C01a_20x_ch00.jpg]

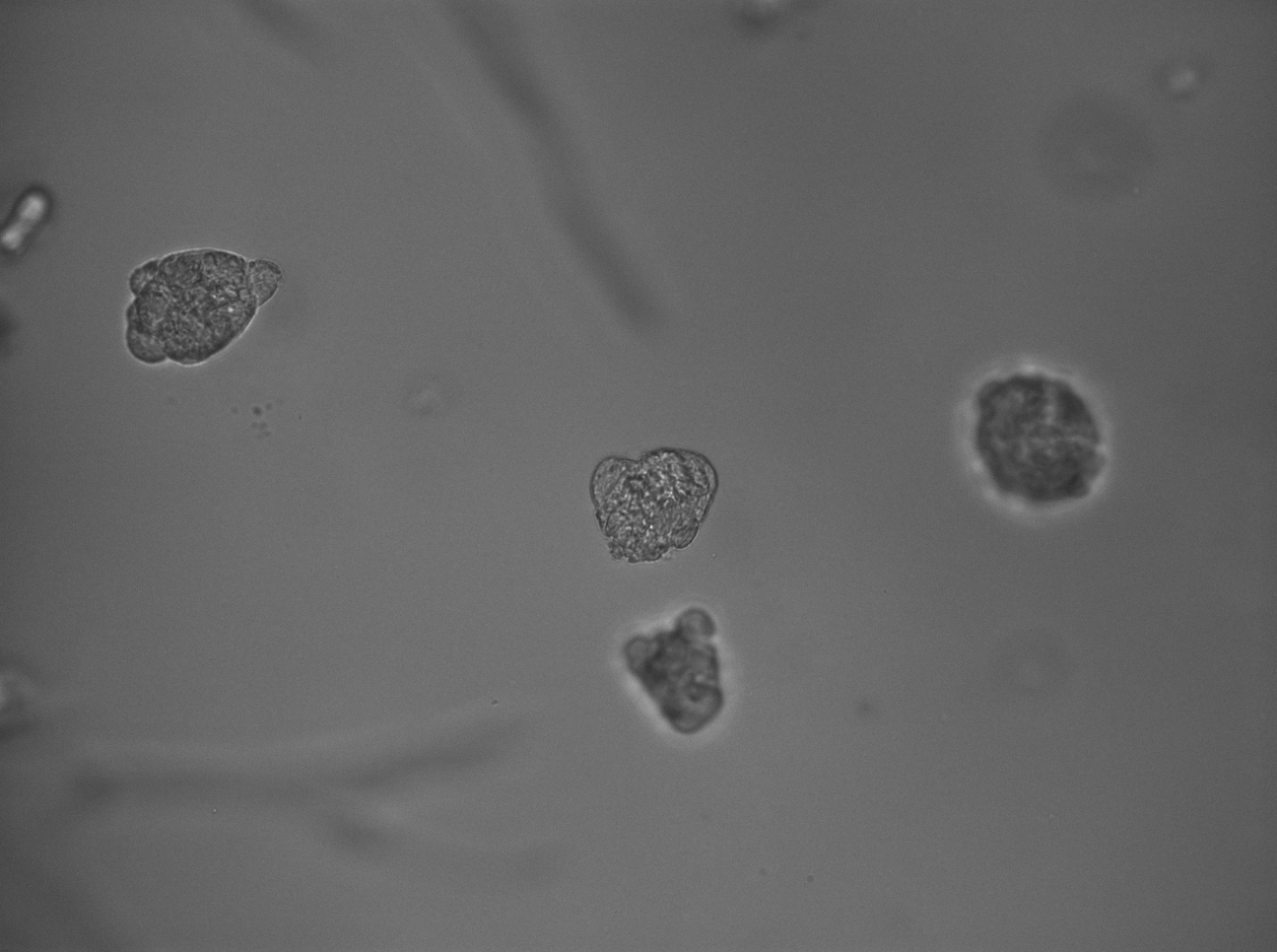

Supplement: Supplementary file 4 — Source Data Fig. 4 [file 41586_2026_10187_MOESM4_ESM.zip › HCEC1CT/HCEC1CT-KRAS_D10_Dox-00125_C01b_20x_ch00.jpg]

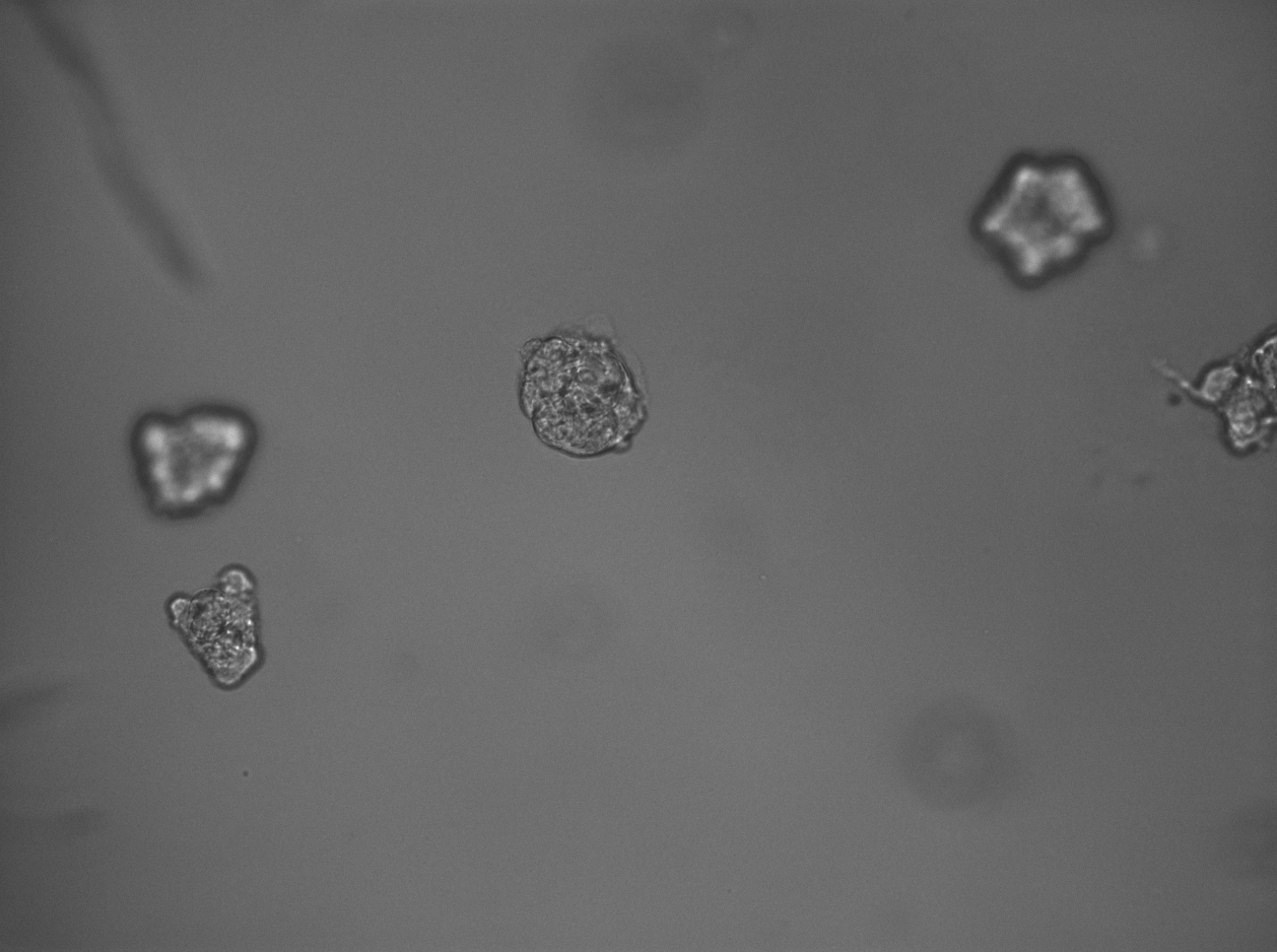

Supplement: Supplementary file 4 — Source Data Fig. 4 [file 41586_2026_10187_MOESM4_ESM.zip › HCEC1CT/HCEC1CT-KRAS_D10_Dox-00125_C01c_20x_ch00.jpg]

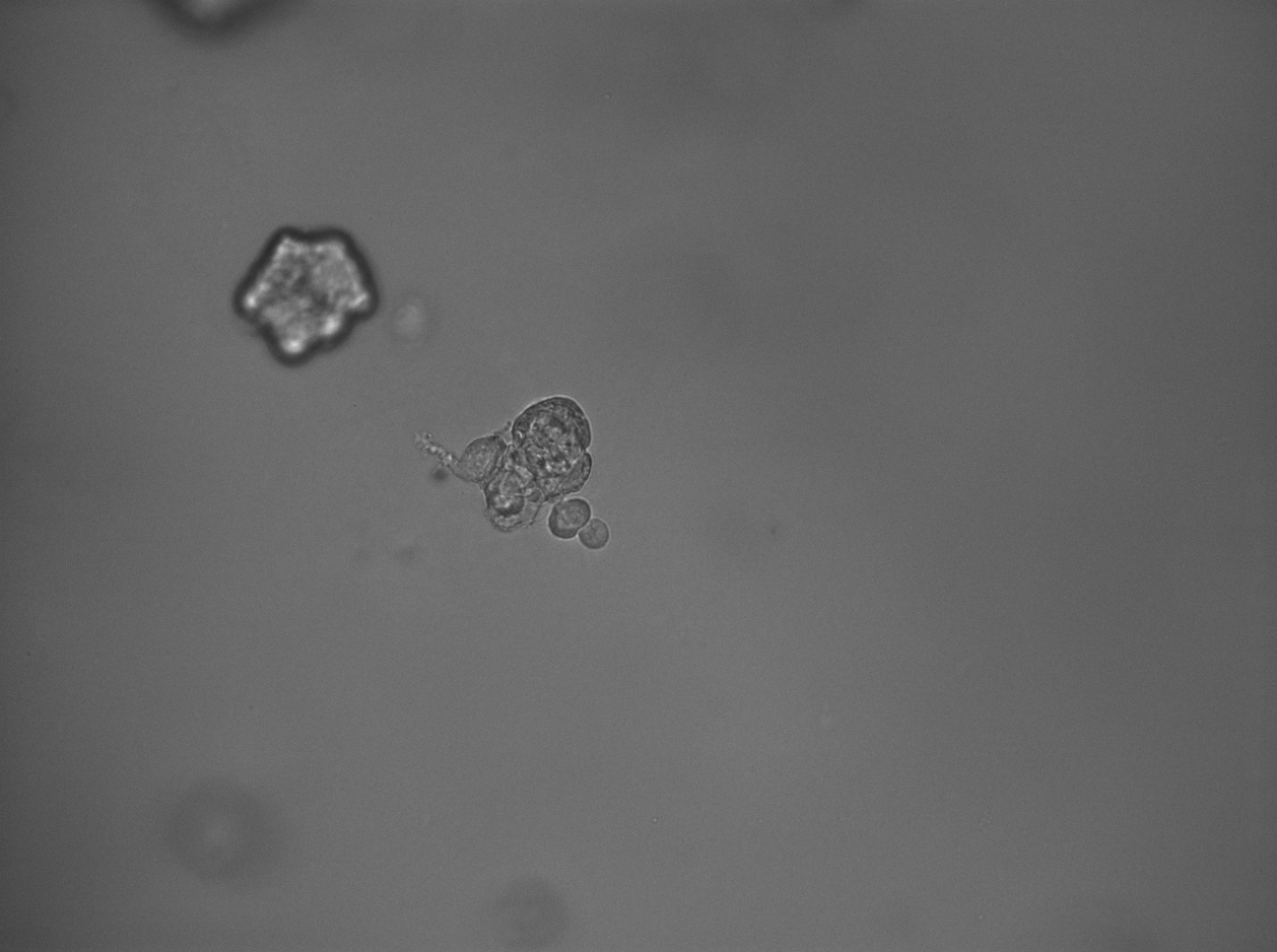

Supplement: Supplementary file 4 — Source Data Fig. 4 [file 41586_2026_10187_MOESM4_ESM.zip › HCEC1CT/HCEC1CT-KRAS_D10_Dox-00125_C01d_20x_ch00.jpg]

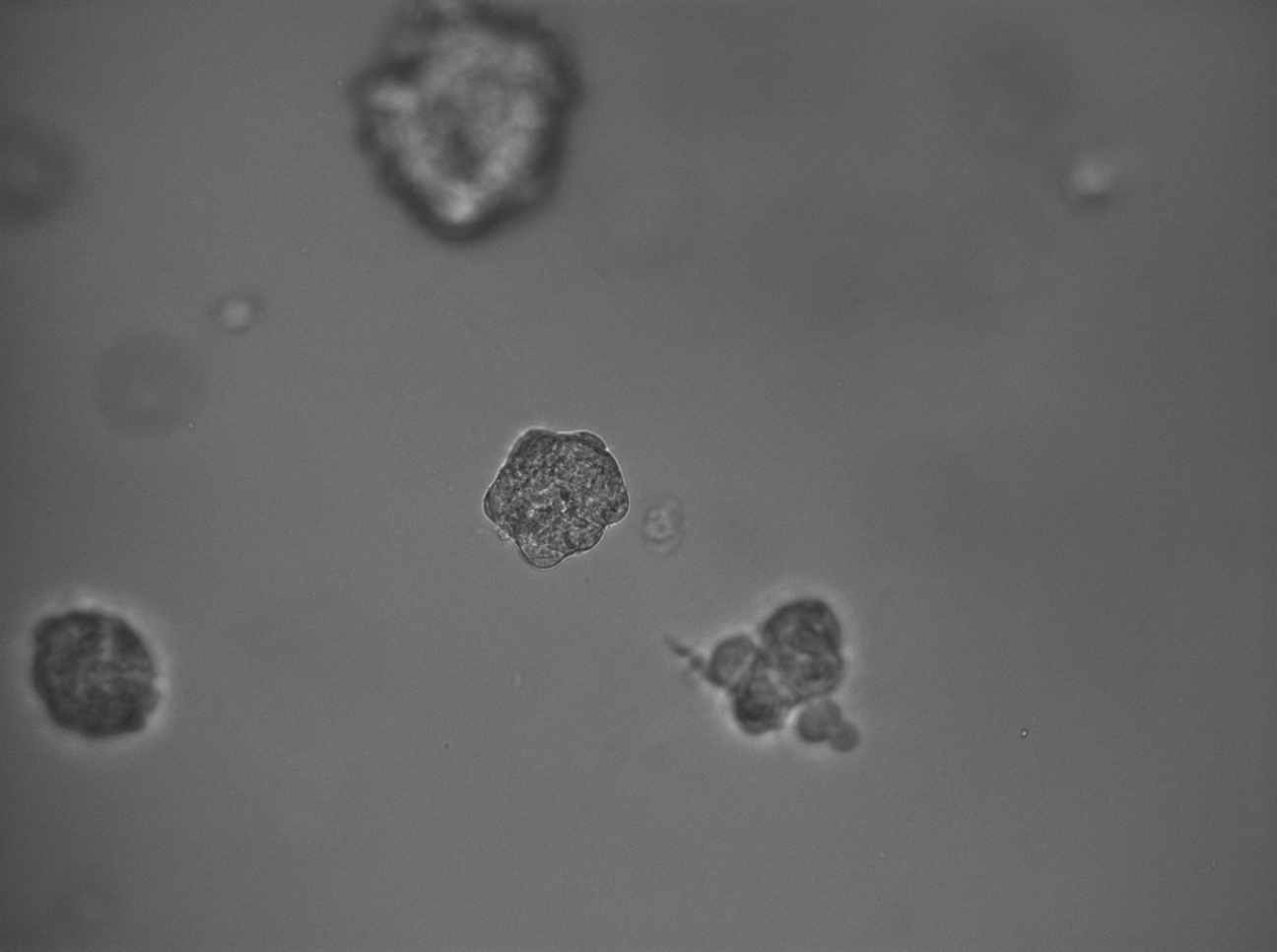

Supplement: Supplementary file 4 — Source Data Fig. 4 [file 41586_2026_10187_MOESM4_ESM.zip › HCEC1CT/HCEC1CT-KRAS_D10_Dox-00125_C01e_20x_ch00.jpg]

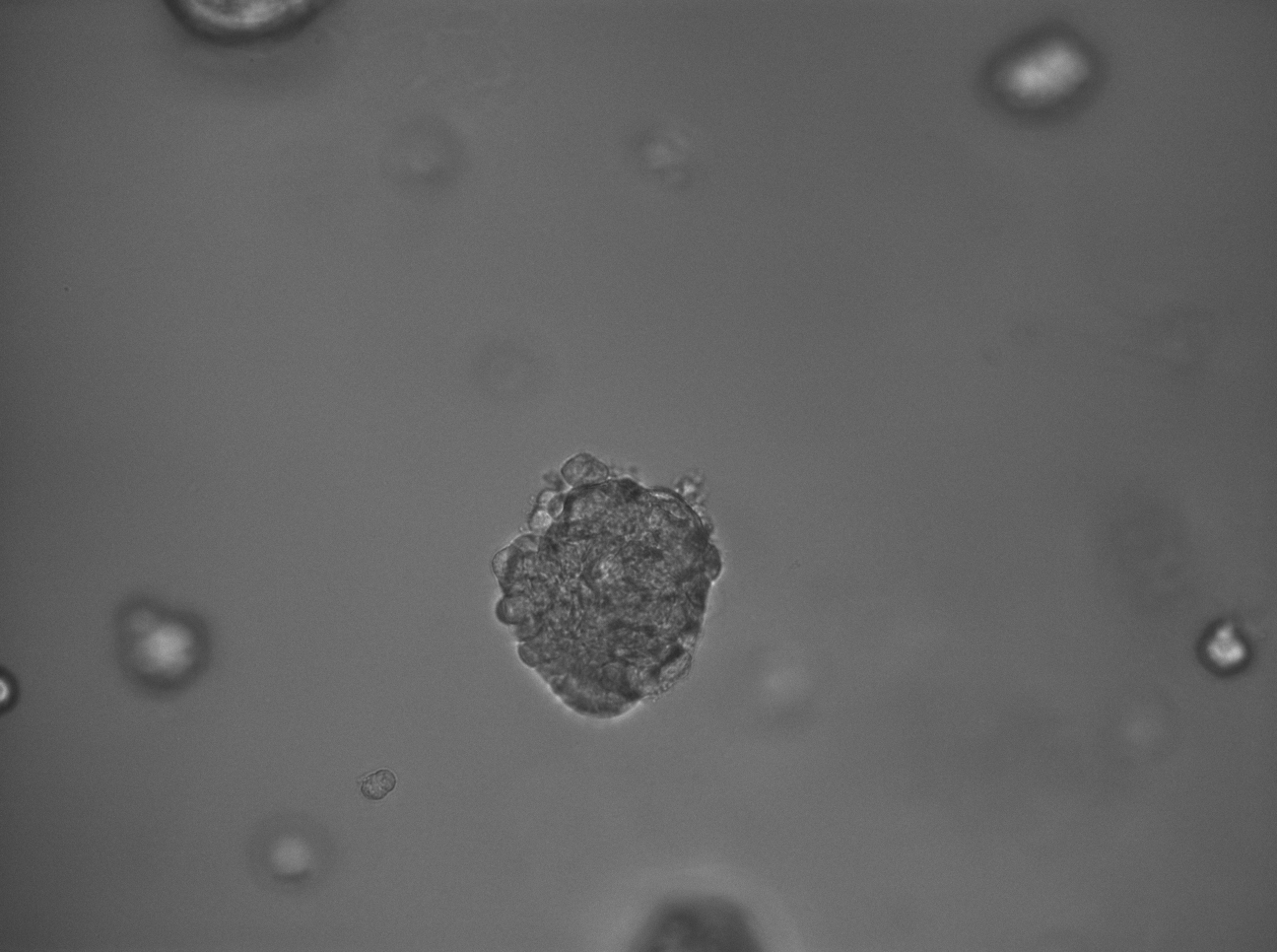

Supplement: Supplementary file 4 — Source Data Fig. 4 [file 41586_2026_10187_MOESM4_ESM.zip › HCEC1CT/HCEC1CT-KRAS_D10_Dox-00125_C01f_20x_ch00.jpg]

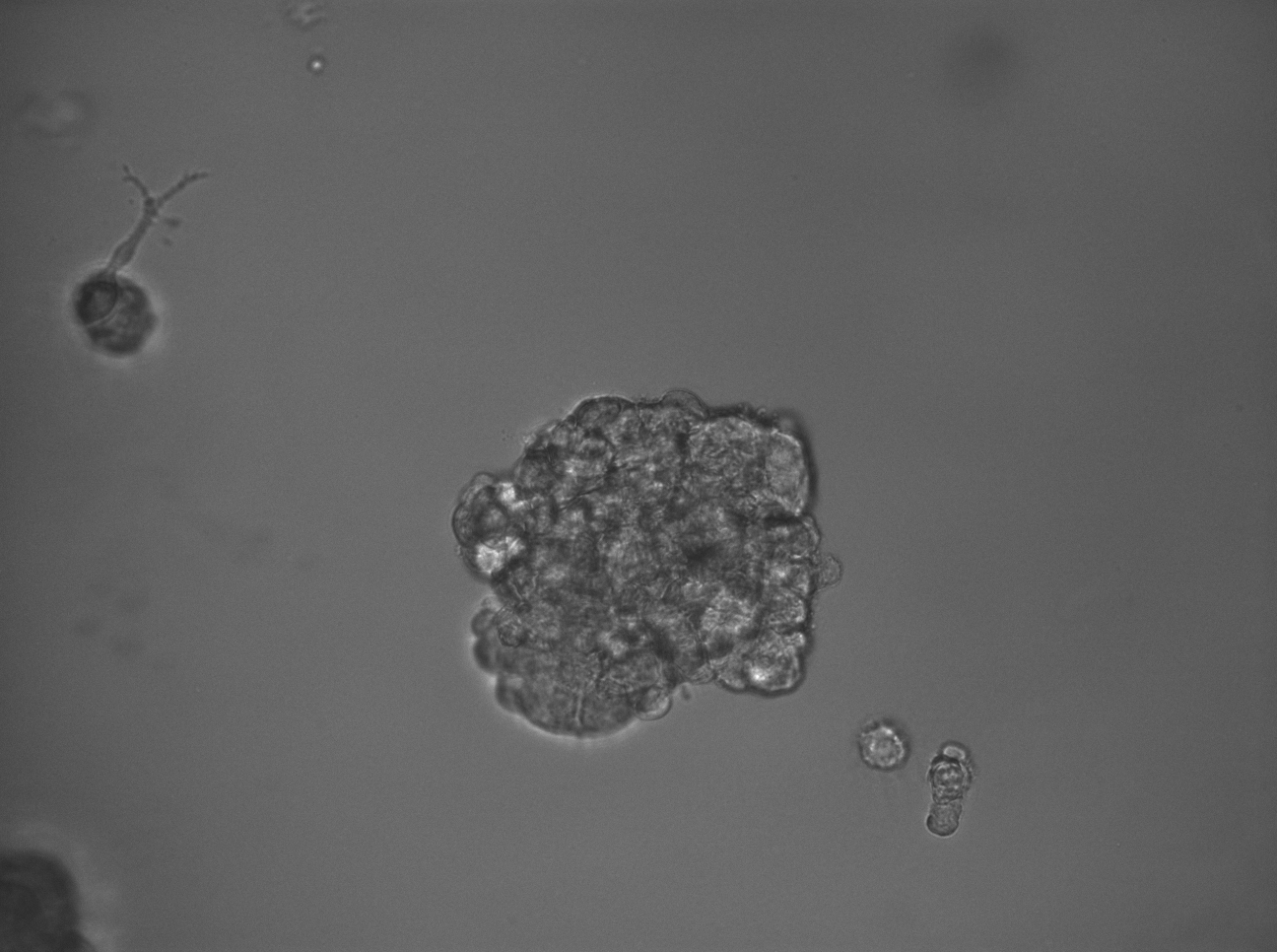

Supplement: Supplementary file 4 — Source Data Fig. 4 [file 41586_2026_10187_MOESM4_ESM.zip › HCEC1CT/HCEC1CT-KRAS_D10_Dox-00125_C01g_20x_ch00.jpg]

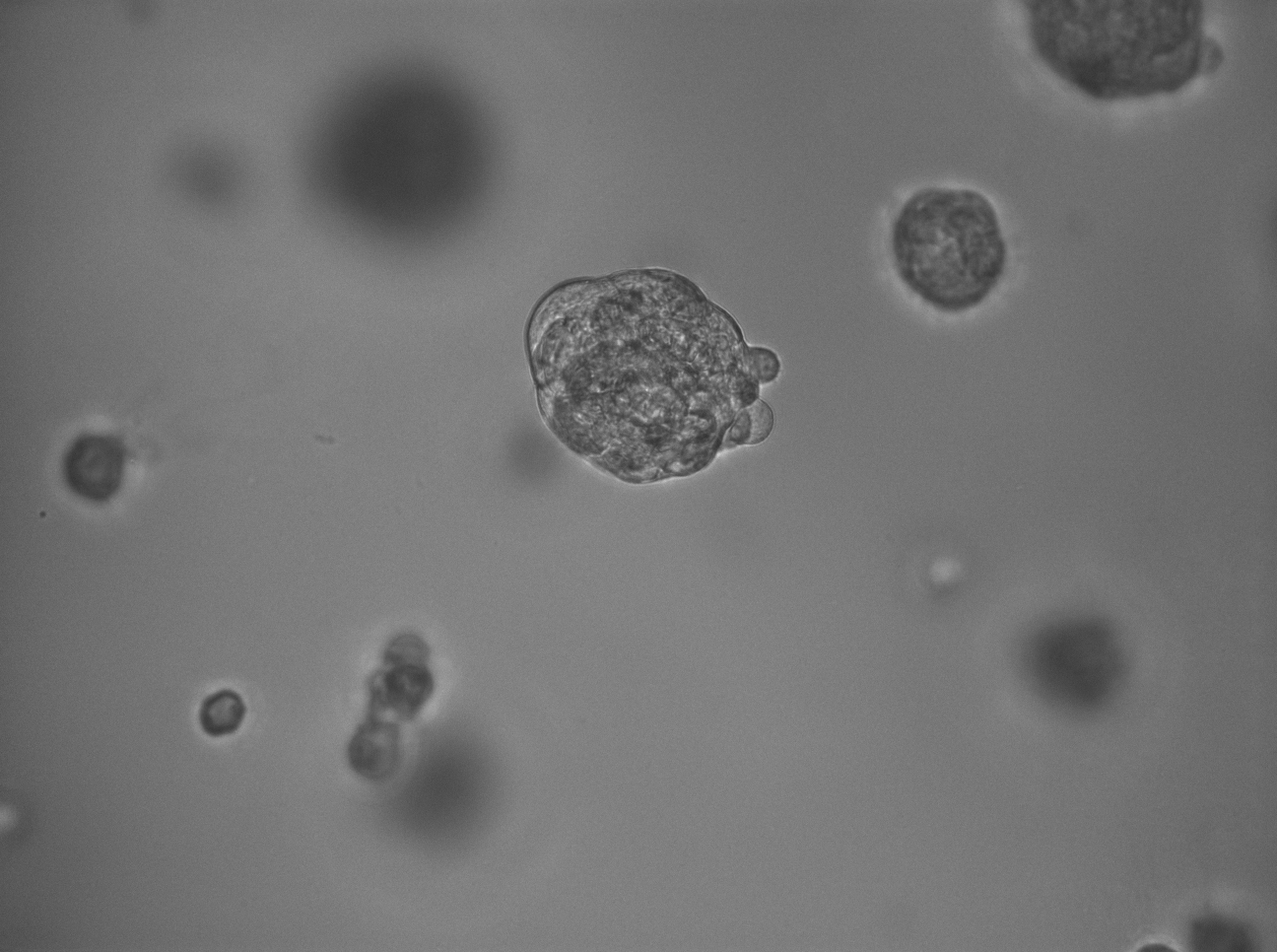

Supplement: Supplementary file 4 — Source Data Fig. 4 [file 41586_2026_10187_MOESM4_ESM.zip › HCEC1CT/HCEC1CT-KRAS_D10_Dox-00125_C02a_20x_ch00.jpg]

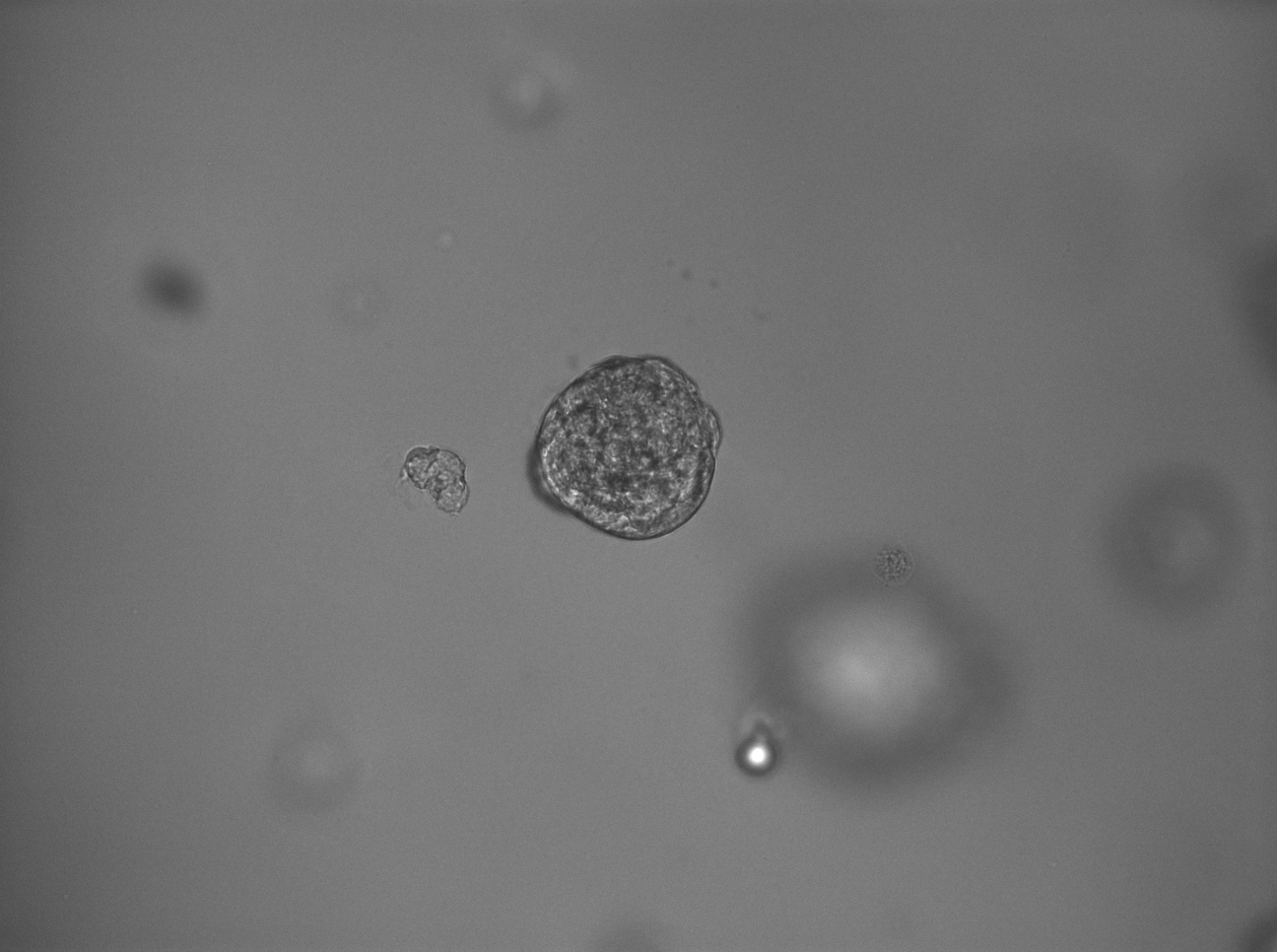

Supplement: Supplementary file 4 — Source Data Fig. 4 [file 41586_2026_10187_MOESM4_ESM.zip › HCEC1CT/HCEC1CT-KRAS_D10_Dox-00125_C02b_20x_ch00.jpg]

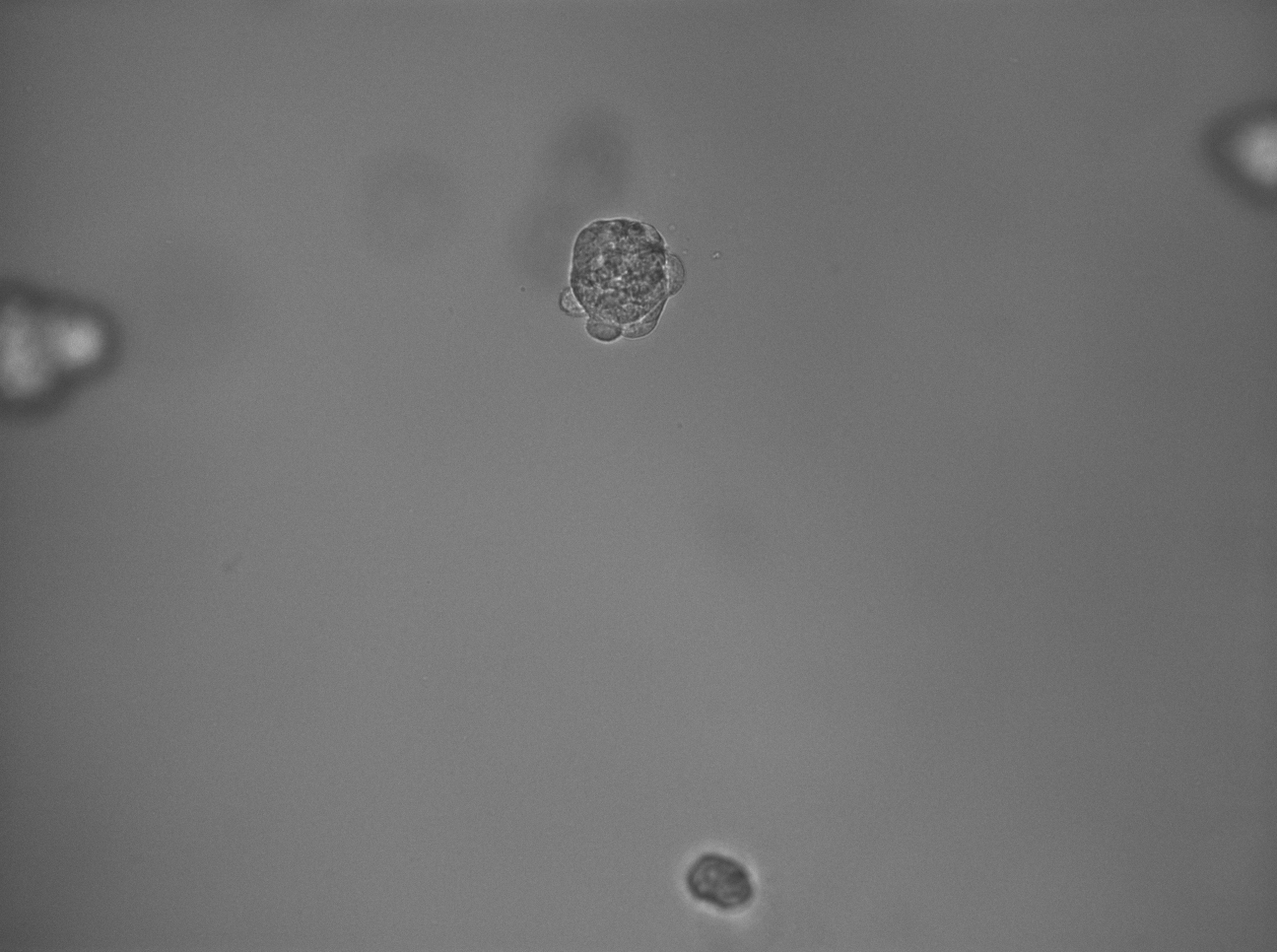

Supplement: Supplementary file 4 — Source Data Fig. 4 [file 41586_2026_10187_MOESM4_ESM.zip › HCEC1CT/HCEC1CT-KRAS_D10_Dox-00125_C02c_20x_ch00.jpg]

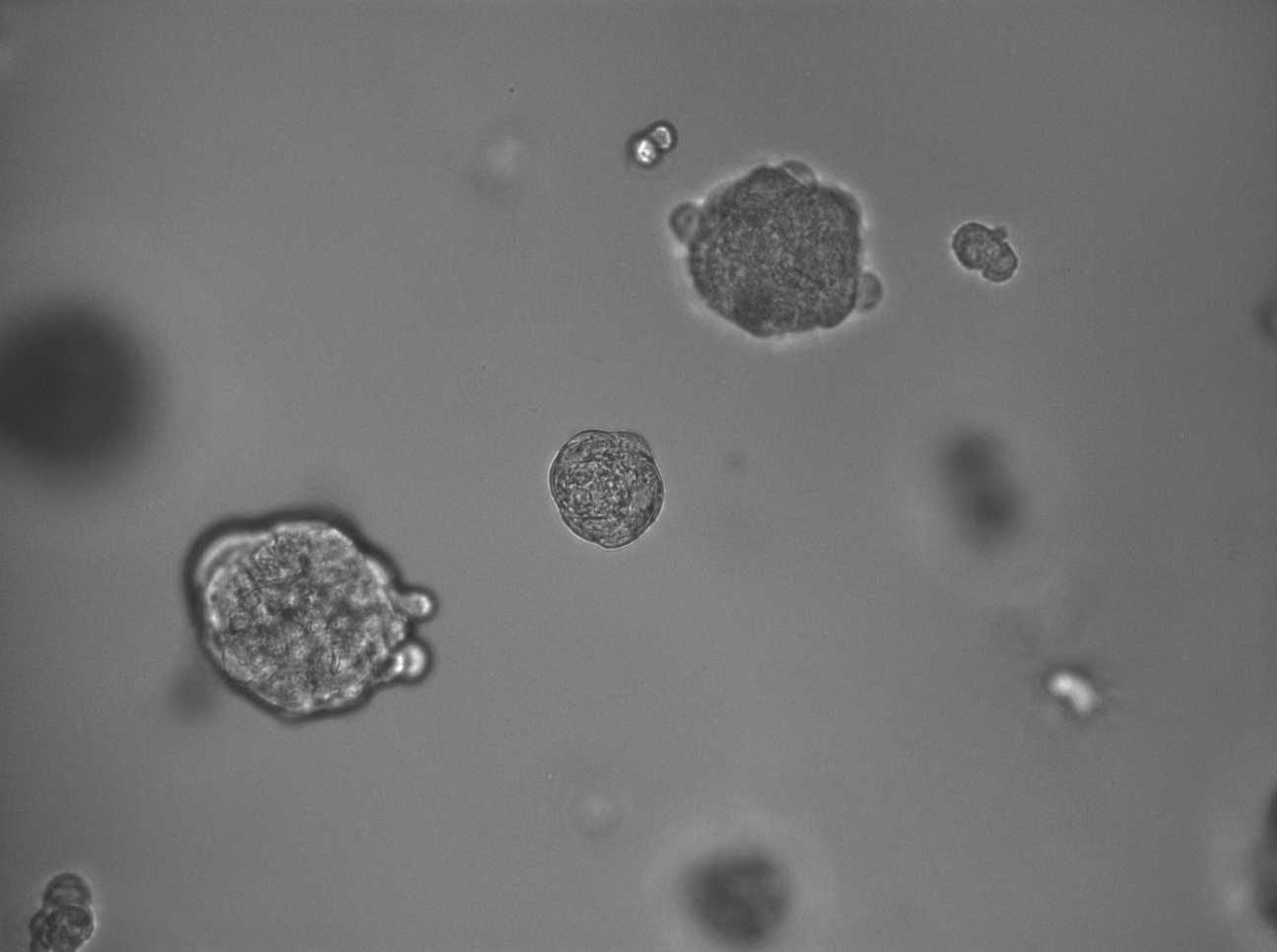

Supplement: Supplementary file 4 — Source Data Fig. 4 [file 41586_2026_10187_MOESM4_ESM.zip › HCEC1CT/HCEC1CT-KRAS_D10_Dox-00125_C02d_20x_ch00.jpg]

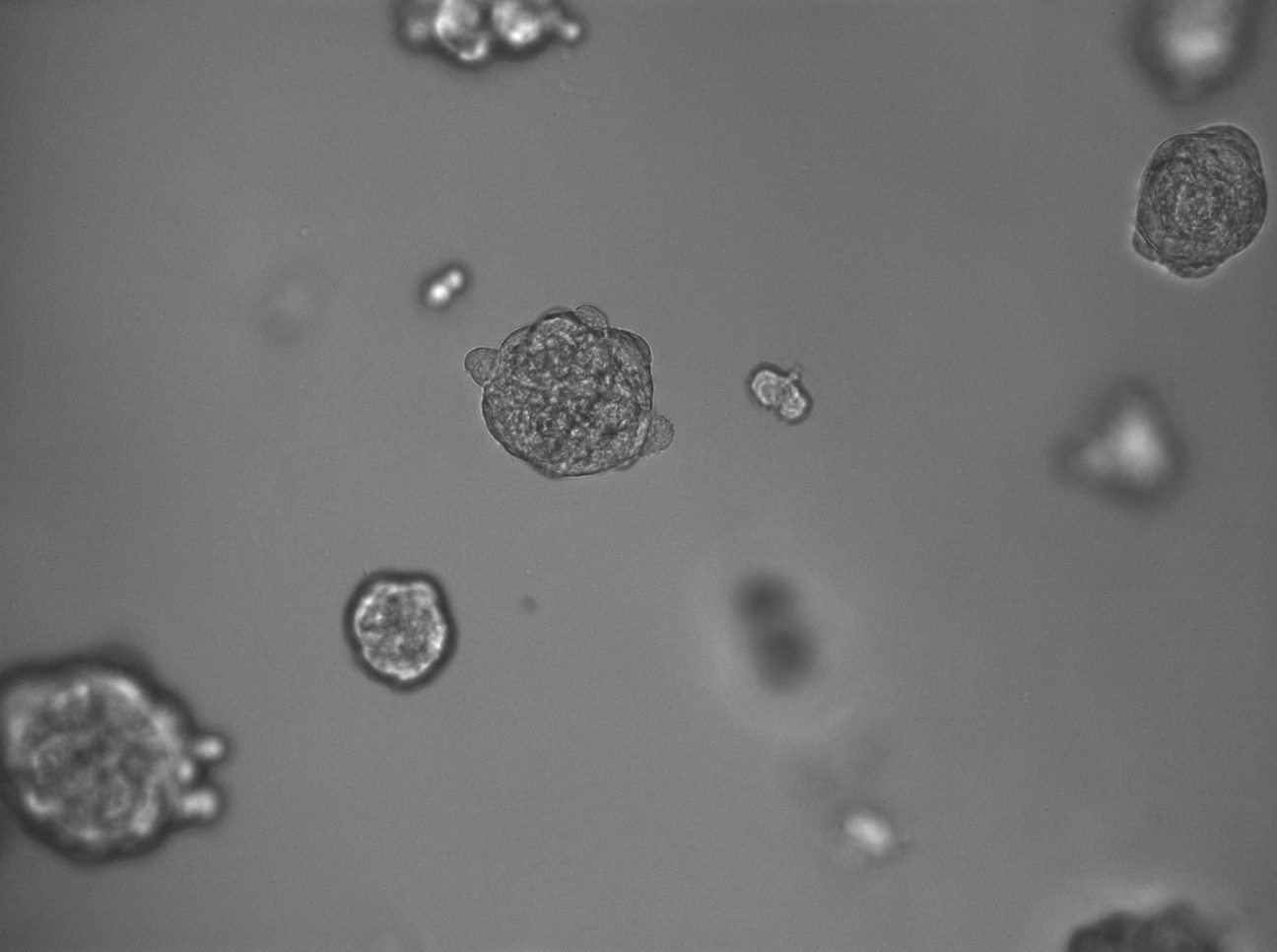

Supplement: Supplementary file 4 — Source Data Fig. 4 [file 41586_2026_10187_MOESM4_ESM.zip › HCEC1CT/HCEC1CT-KRAS_D10_Dox-00125_C02e_20x_ch00.jpg]

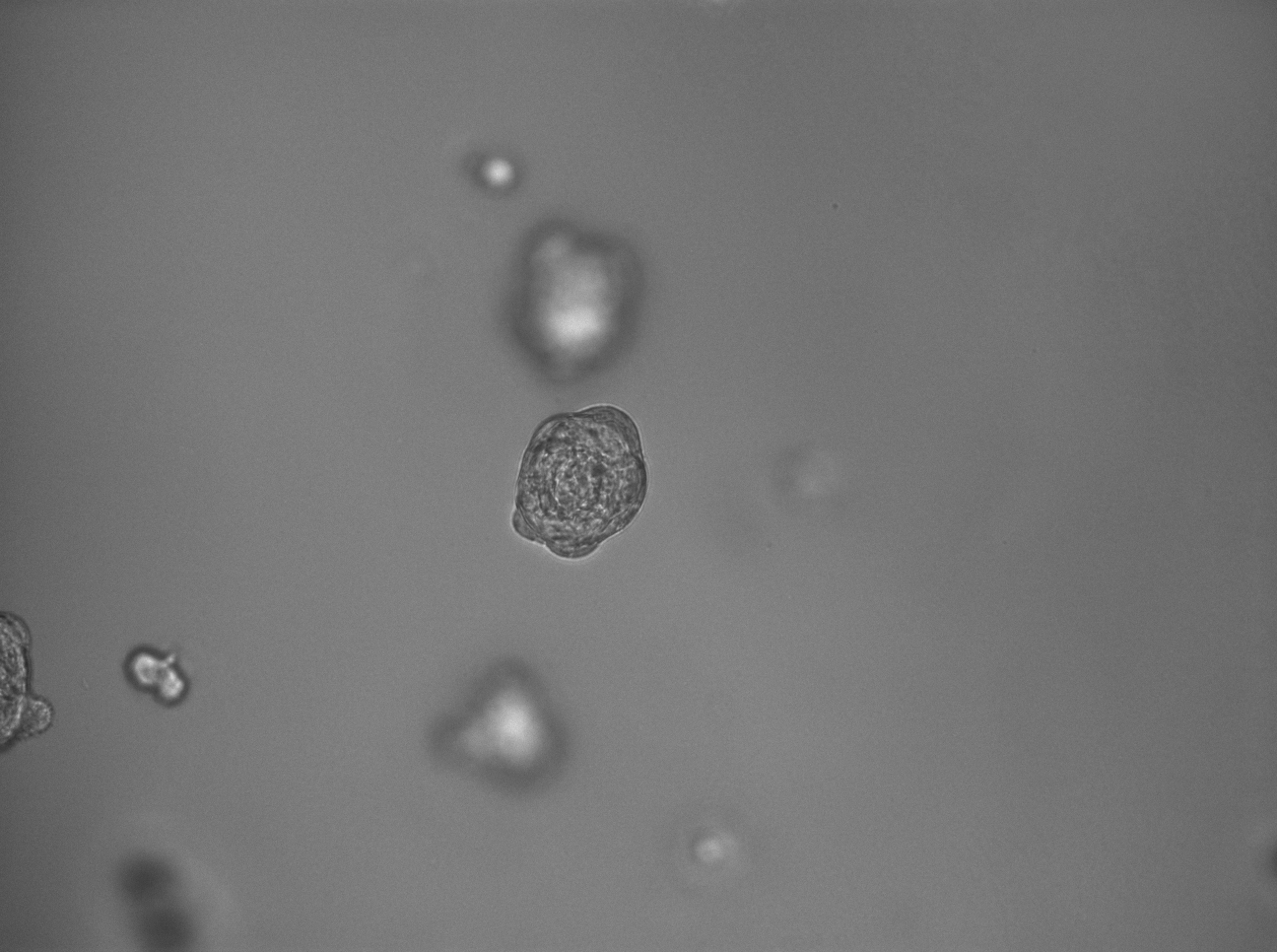

Supplement: Supplementary file 4 — Source Data Fig. 4 [file 41586_2026_10187_MOESM4_ESM.zip › HCEC1CT/HCEC1CT-KRAS_D10_Dox-00125_C02f_20x_ch00.jpg]

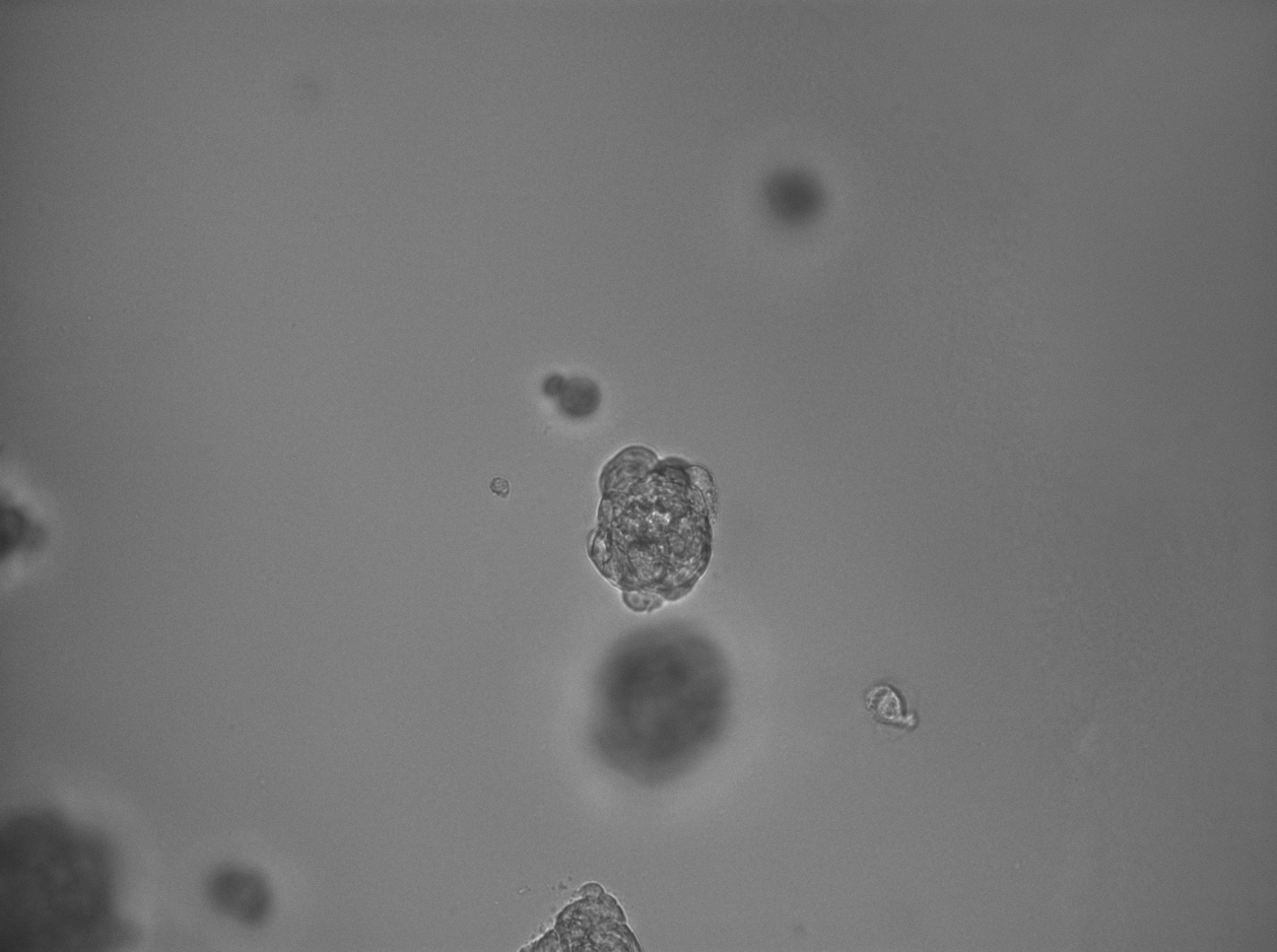

Supplement: Supplementary file 4 — Source Data Fig. 4 [file 41586_2026_10187_MOESM4_ESM.zip › HCEC1CT/HCEC1CT-KRAS_D10_Dox-00125_C02g_20x_ch00.jpg]

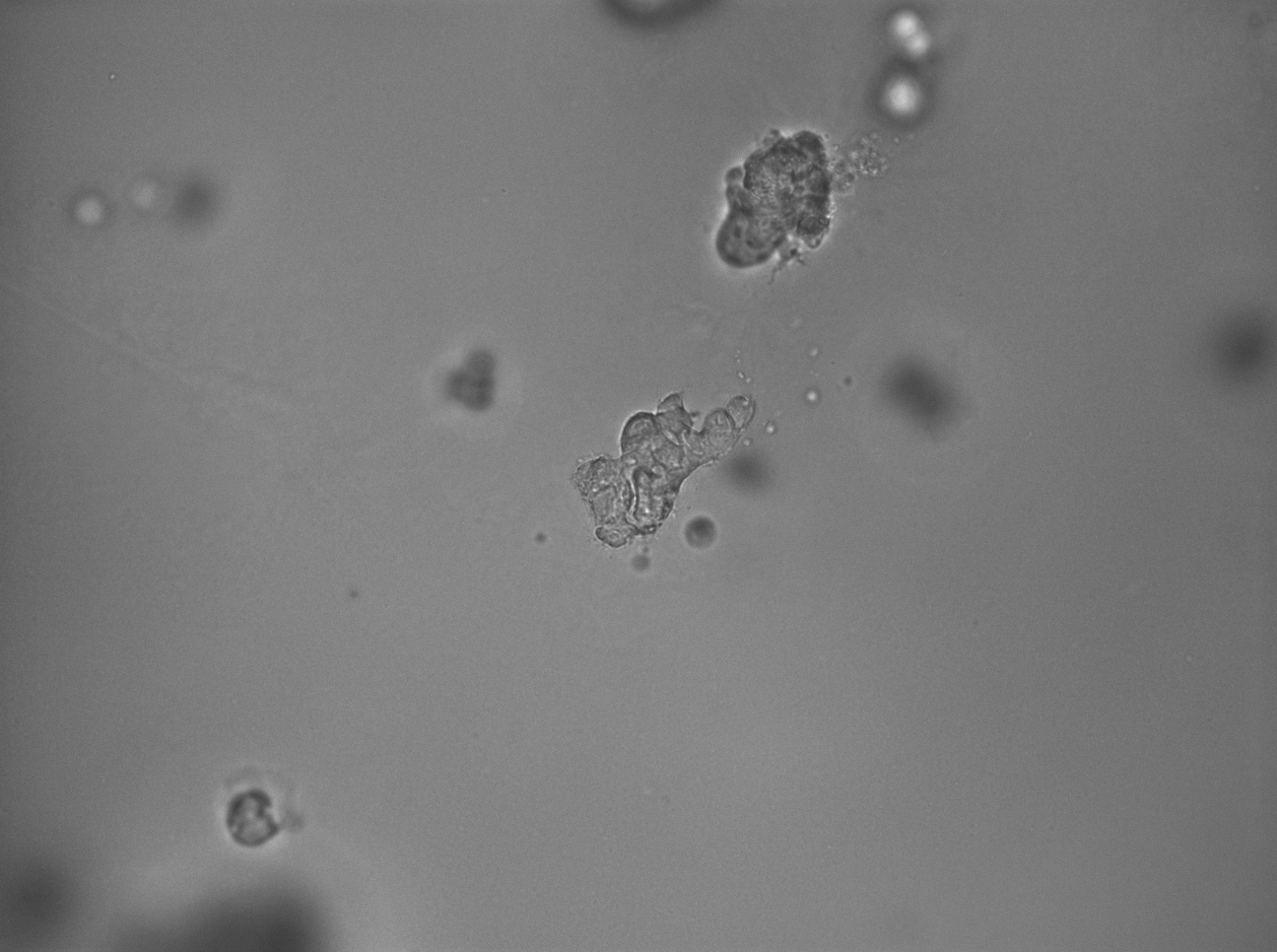

Supplement: Supplementary file 4 — Source Data Fig. 4 [file 41586_2026_10187_MOESM4_ESM.zip › HCEC1CT/HCEC1CT-KRAS_D10_Dox-00125_C03a_20x_ch00.jpg]

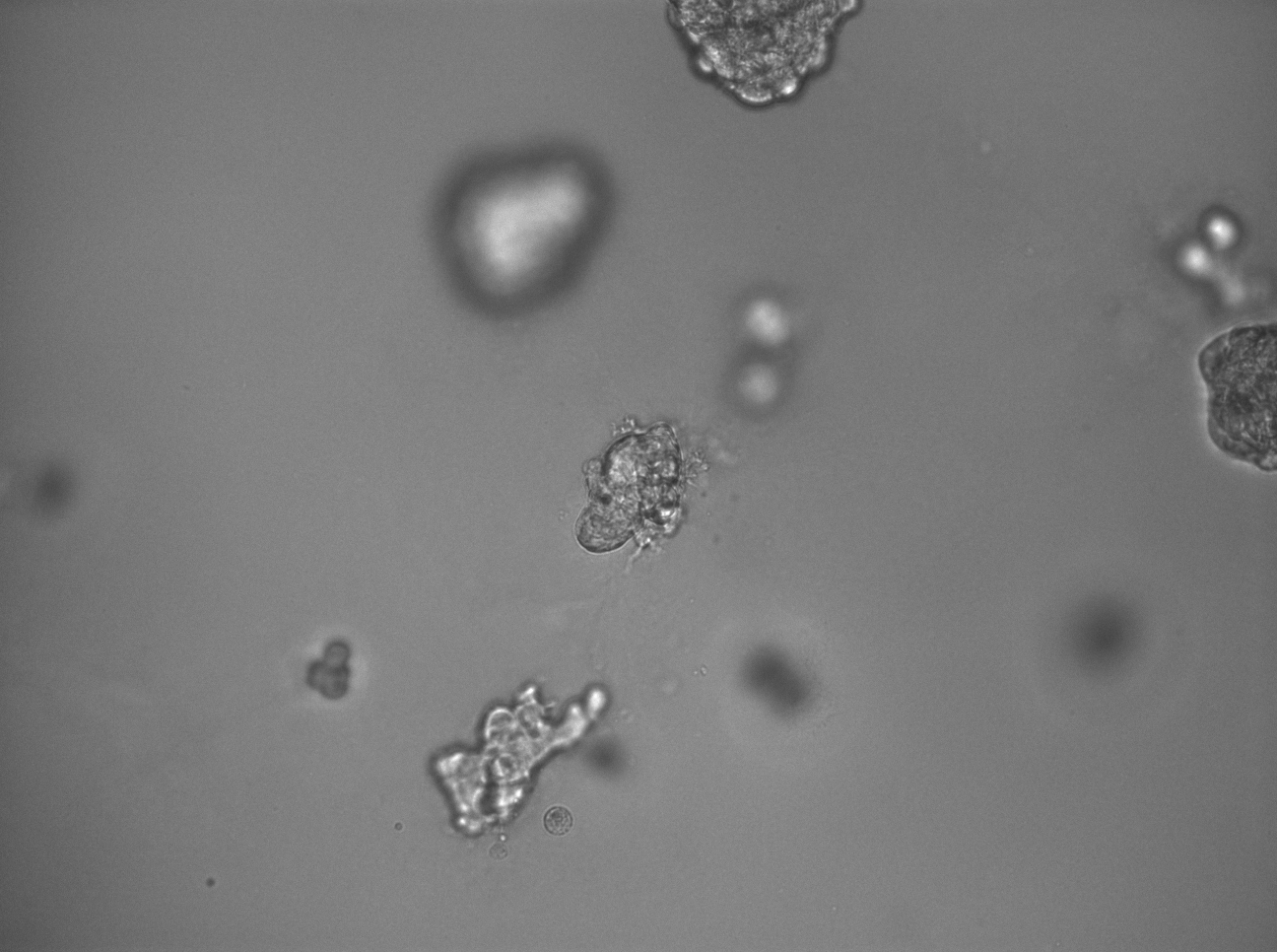

Supplement: Supplementary file 4 — Source Data Fig. 4 [file 41586_2026_10187_MOESM4_ESM.zip › HCEC1CT/HCEC1CT-KRAS_D10_Dox-00125_C03b_20x_ch00.jpg]

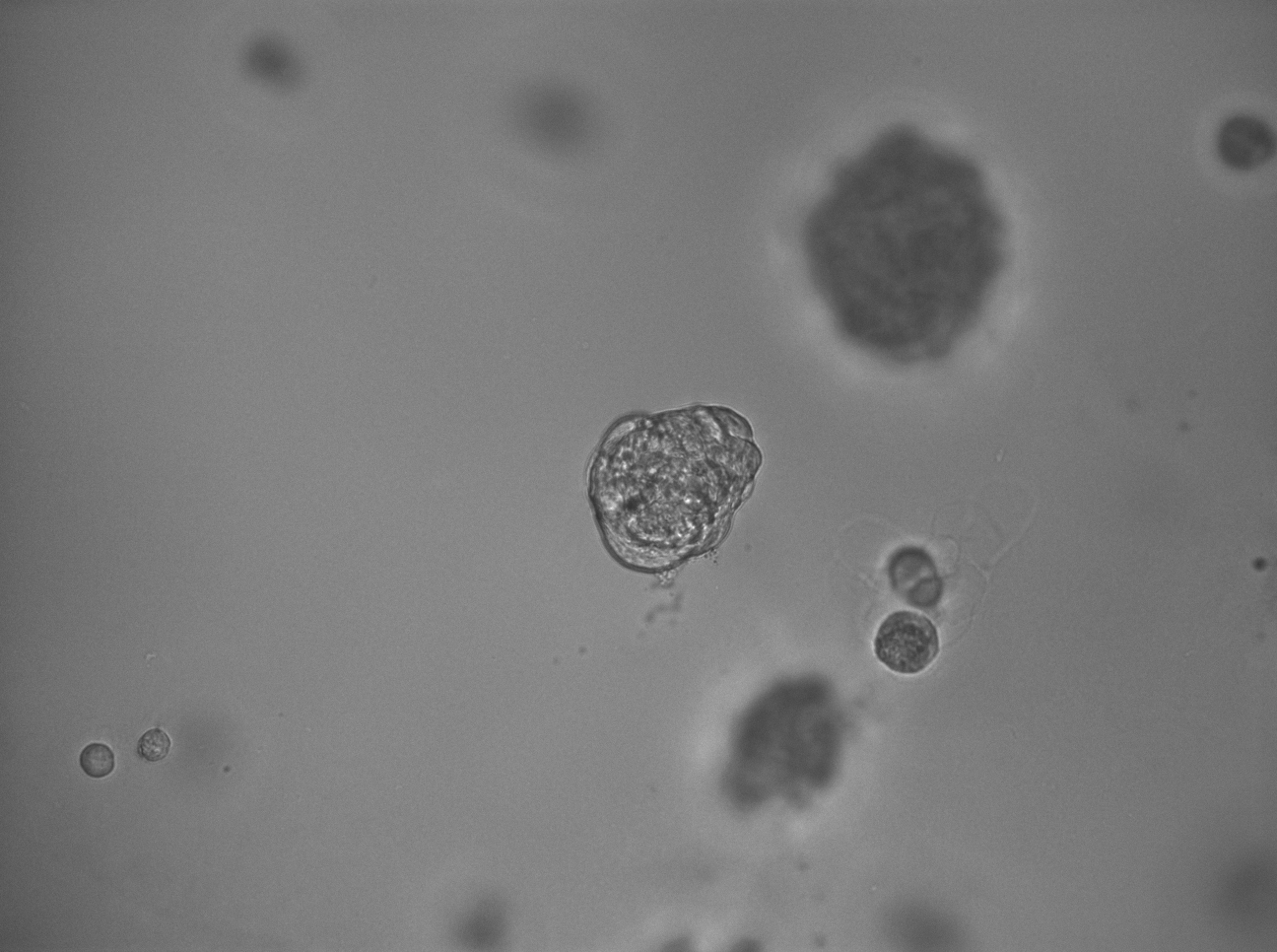

Supplement: Supplementary file 4 — Source Data Fig. 4 [file 41586_2026_10187_MOESM4_ESM.zip › HCEC1CT/HCEC1CT-KRAS_D10_Dox-00125_C03c_20x_ch00.jpg]

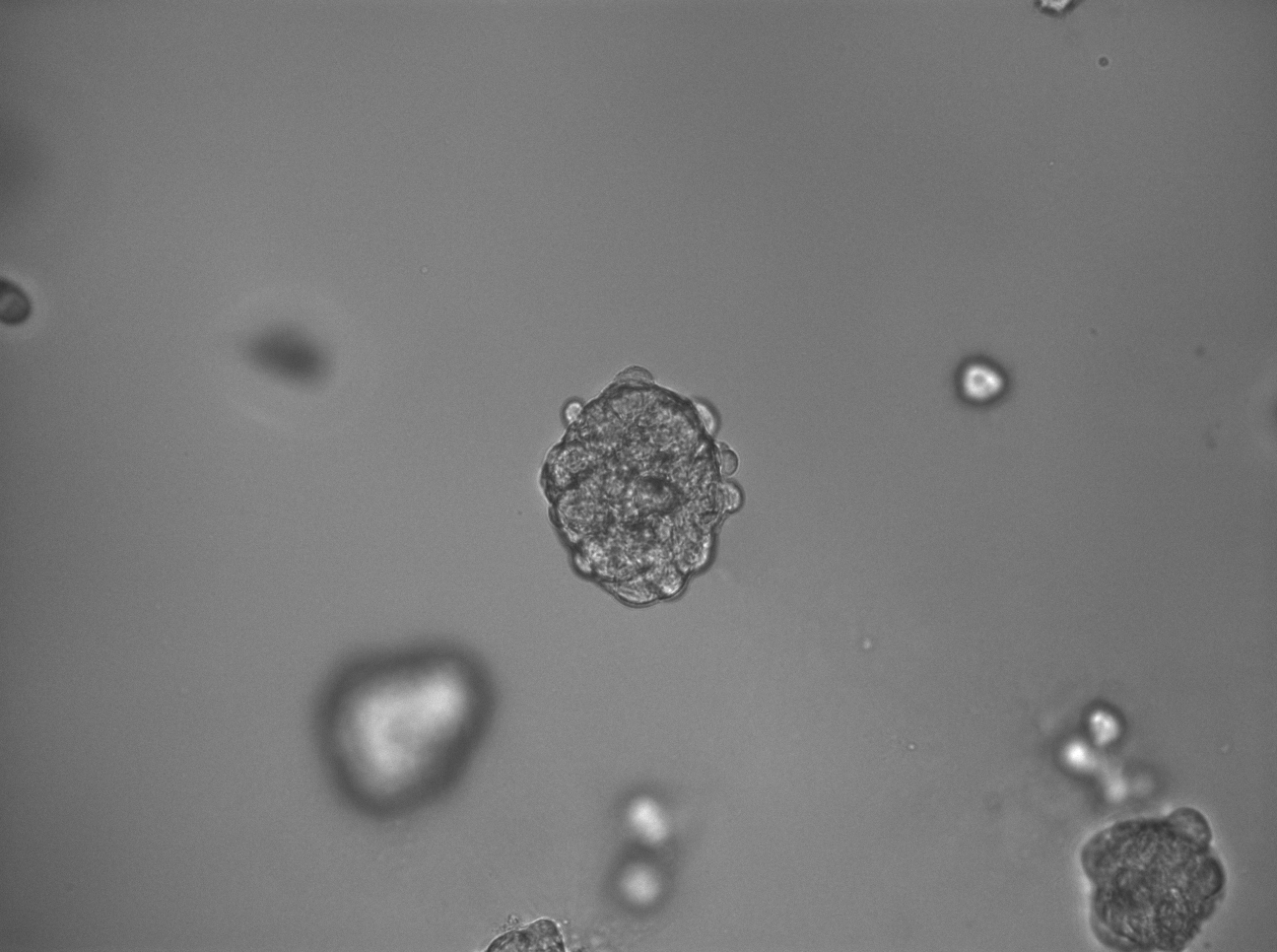

Supplement: Supplementary file 4 — Source Data Fig. 4 [file 41586_2026_10187_MOESM4_ESM.zip › HCEC1CT/HCEC1CT-KRAS_D10_Dox-00125_C03d_20x_ch00.jpg]
