# Supplementary figures and images for: A disease model resource reveals core principles of tissue-specific cancer evolution (part 3 of 3)
Source: Nature. 2026 Feb 25;653(8113):57. doi: 10.1038/s41586-026-10187-2 (PMC13149333; doi:10.1038/s41586-026-10187-2)

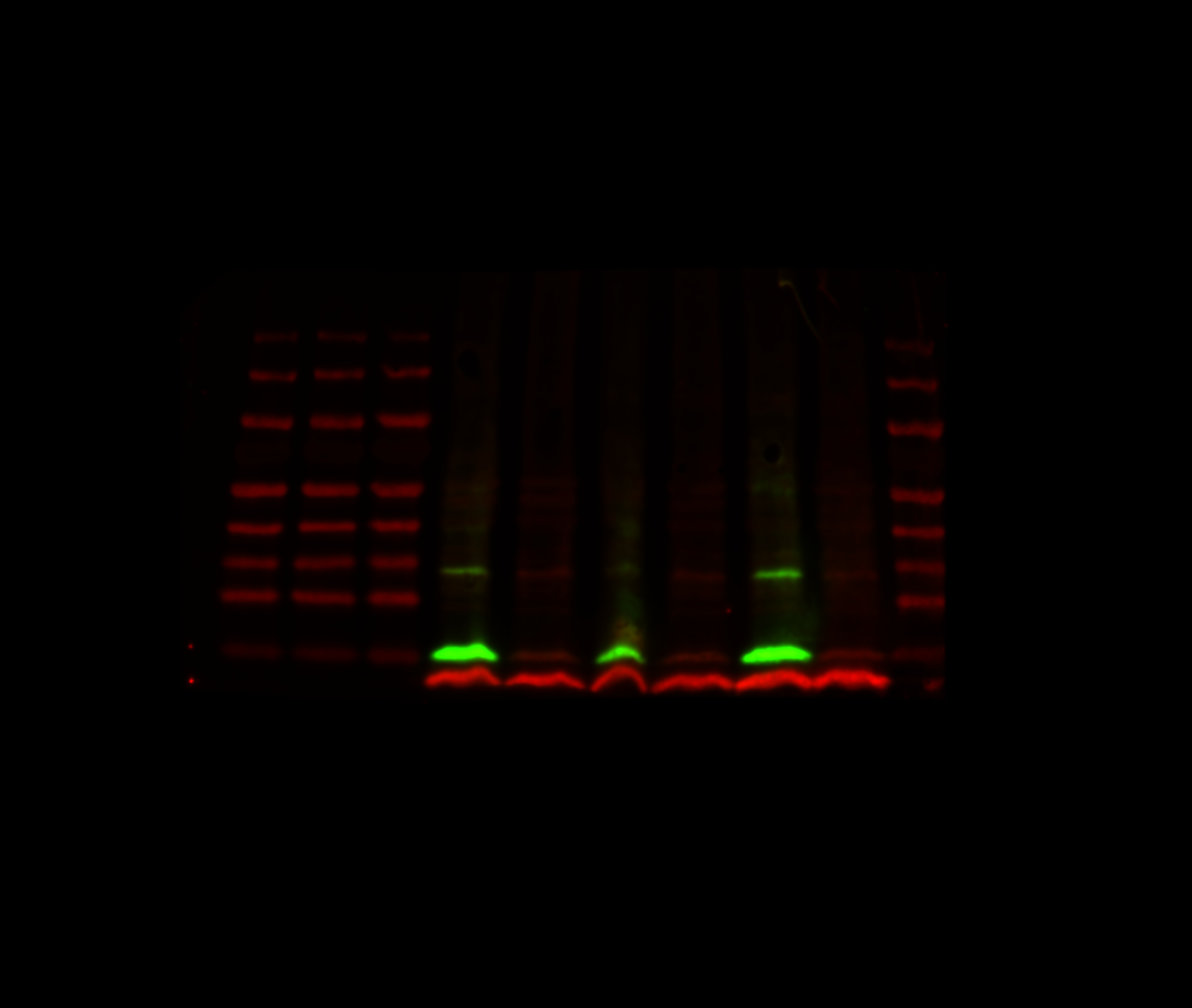

Supplement: Supplementary file 5 — Source Data Extended Data Fig. 11 [file 41586_2026_10187_MOESM5_ESM.zip › WB_120625_organoids_color.tif.tif]

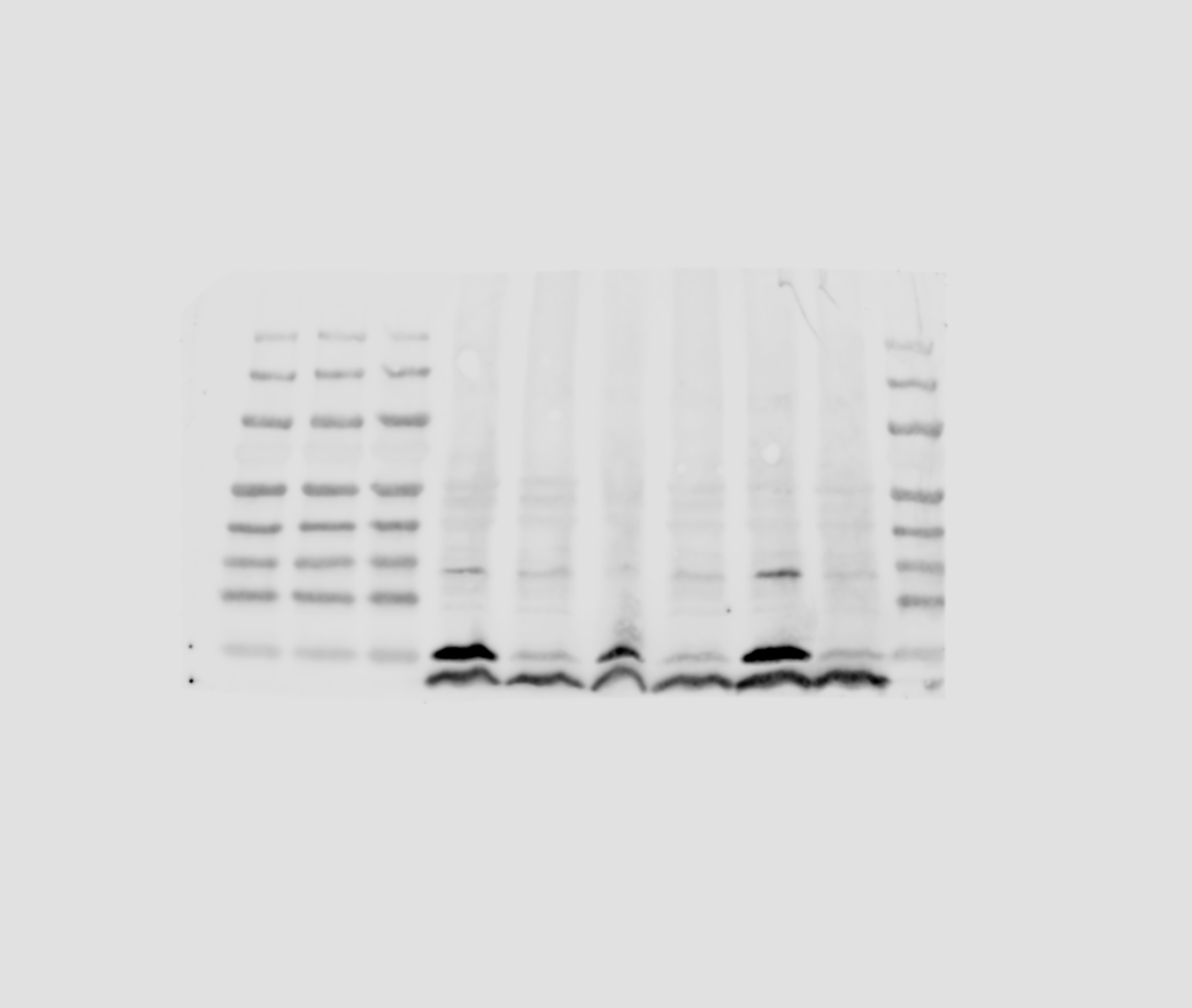

Supplement: Supplementary file 5 — Source Data Extended Data Fig. 11 [file 41586_2026_10187_MOESM5_ESM.zip › WB_120625_organoids_bw.tif.tif]
